# Bibliometric Study of Sodium Glucose Cotransporter 2 Inhibitors in Cardiovascular Research

**DOI:** 10.3389/fphar.2020.561494

**Published:** 2020-09-15

**Authors:** Lu Chen, Siyuan Ma, Donghong Hu, Hairuo Lin, Yingqi Zhu, Kaitong Chen, Lin Chen, Cankun Zheng, Jichen Liu, Yulin Liao

**Affiliations:** Department of Cardiology, State Key Laboratory of Organ Failure Research, Nanfang Hospital, Southern Medical University, Guangzhou, China

**Keywords:** sodium glucose co-transporter 2 inhibitors, bibliometrics, cardiovascular research, heart failure, publication trend, co-occurrence analysis, co-citation analysis

## Abstract

**Background:**

An increasing number of studies have shown that sodium glucose cotransporter 2 (SGLT2) inhibitors, initially used as antidiabetic agents, have cardiovascular (CV) benefits. However, few bibliometric analyses have examined this field systematically. Our study aimed to visualize the publications to determine the trends and hotspots in CV research on SGLT2 inhibitors.

**Methods:**

Publications on SGLT2 inhibitors in cardiovascular research were retrieved from the Web of Science Core Collection. Microsoft Excel 2019, VOSviewer, and CiteSpace V were used to analyze and plot the references.

**Results:**

On July 3, 2020, 1509 records of CV research on SGLT2 inhibitors published from 2013 to 2020 were retrieved. Nearly half were authored by American scholars, and most were published in *Diabetes Obesity Metabolism*, *Cardiovascular Diabetology*, and *Diabetes Therapy*. The USA was the leading driving force, with a strong academic reputation in this area. Inzucchi SE published the most related articles, while Neal B was cited the most frequently. All the top 10 co-cited references were in the leading co-cited journal, *The New England Journal of Medicine*. “Atherosclerotic cardiovascular event” was the leading research hotspot. The keywords “cardiac metabolism,” “heart failure hospitalization,” and “heart failure with preserved ejection fraction” appeared most recently as research frontiers.

**Conclusion:**

Most studies focused on clinical trial outcomes, such as cardiovascular death and heart failure (HF) hospitalization. The mechanisms of SGLT2 inhibitors, especially those related to cardiac metabolism, may soon become hotspots and should be closely monitored.

## Introduction

Sodium glucose cotransporter 2 (SGLT2) inhibitors were initially identified for their glycemic effects in type 2 diabetes mellitus (T2DM). By inhibiting the protein SGLT2, which is expressed in the proximal convoluted tubule, SGLT2 inhibitors lower glucose levels independent of insulin action. Currently, SGLT2 inhibitors are recognized to possess a considerably broader scope of benefits in addition to simply hypoglycemic effects, including the induction of modest weight loss, natriuresis, and diuresis and reducing blood pressure (Zelniker and Braunwald, 2018; Park et al., 2020). Traditional antidiabetic drugs do not always reduce the cardiovascular (CV) complications of diabetes; however, the potential applications of SGLT2 inhibitors in CV disease (Docherty et al., 2020; Heerspink et al., 2020; Slomski, 2020) have attracted increasing intense research interest.

Phlorizin, a naturally occurring nonselective SGLT2 inhibitor, was first extracted from apple tree bark by von Chr. Petersen in 1835. However, not until 1987—when Rossetti et al. (1987) at Yale University unprecedently demonstrated that phlorizin completely normalized insulin sensitivity in diabetic rats without hypoglycemia—was a connection made between SGLT2 inhibitors and the treatment of diabetes. To date, seven types of specific SGLT2 inhibitors have been developed worldwide (canagliflozin, dapagliflozin, empagliflozin, ertugliflozin, ipragliflozin, luseogliflozin, and tofogliflozin), the first four of which are widely approved by the U.S. Food and Drug Administration (FDA) as antihyperglycemics.

Unexpected CV benefits of SGLT2 inhibitors were gradually identified, as initially shown in the landmark EMPA-REG OUTCOME trial (Zinman et al., 2015) and later in the clinical trials CANVAS (Neal et al., 2017) and DECLARE (Wiviott et al., 2019). The results of these clinical trials indicated that SGLT2 inhibitors, including empagliflozin, canagliflozin, and dapagliflozin, lead to a significant reduction in the composite outcomes of myocardial infarction, stroke, and CV death and/or hospitalization for heart failure (HF) in patients with T2DM. Recently, the striking results of the DAPA-HF (Mcmurray et al., 2019) trial indicated that dapagliflozin is equally effective independent of the presence of diabetes in patients with HF and reduced ejection fraction (HFrEF), reducing the risk of worsening HF and CV death by 26% and the rate of all-cause mortality by 17%. Additionally, SGLT2 inhibitors have relatively few side effects and rarely cause symptomatic hypoglycemia, even when given to patients without diabetes (AI-Jobori et al., 2017)). Considering the different phenotypes of HF at baseline, two parallel trials, EMPEROR-Preserved (NCT03057951 and NCT03619213) and EMPEROR-Reduced (NCT03057977), were conducted to clarify whether the benefits of SGLT2 inhibitors extend to each subset of HF. The former trial observed patients with HF and preserved ejection fraction (HFpEF), a syndrome with no conclusively demonstrated therapeutic benefit (Paulus and van Ballegoij, 2010).

Conversely, numerous studies in preclinical models have been conducted to confirm the CV benefits of SGLT2 inhibitors, including reducing infarct size in reperfused ischemic hearts (Andreadou et al., 2020), attenuating cardiac fibrosis (Lee et al., 2019) and coronary microvascular dysfunction (Adingupu et al., 2019), reducing cardiac inflammation (Byrne et al., 2020), ameliorating adverse ventricular remodeling by affecting cardiac metabolism (Santos-Gallego et al., 2019; Yurista et al., 2019), and improving cardiac function. However, the precise molecular mechanisms underlying those processes remain unclear. With the rapid development of SGLT2 inhibitors in CV research, staying abreast of emerging trends and key milestones in the development of relevant knowledge is highly important. However, few systematic analyses of these publications have been performed.

Bibliometric analysis has been widely used to organize the knowledge structure and explore developmental trends in many research fields *via* quantitative analysis of patterns in the scientific literature (Chen, 2004; Chen et al., 2014). This method enables researchers to understand the range of research topics and predict future directions. To our knowledge, no previous bibliometric analysis of SGLT2 inhibitors in CV research has been performed. We thus aimed to describe the scientific outputs of CV research on SGLT2 inhibitors to determine trends and hotspots to guide investigators’ future work.

## Methods

### Search Strategies

Data were downloaded from the Science Citation Index-Expanded database of the Web of Science Core Collection (WoSCC) on a single day, July 3, 2020. The search terms used were the following: (“SGLT2 inhibit*” OR “sodium glucose cotransporter 2 inhibit*” OR “sodium glucose transporter 2 inhibit*” OR “gliflozin*” OR “canagliflozin” OR “dapagliflozin” OR “empagliflozin” OR “ertugliflozin” OR “ipragliflozin” OR “luseogliflozin” OR “tofogliflozin”) AND (“heart” OR “*cardi*”). Only original articles and reviews written in English and published between 2013 and 2020 were included. This query resulted in 1509 records, which were obtained for this study.

### Data Collection and Analysis

All records retrieved from the WoSCC were downloaded independently by two authors (LC, SM) and included the number of annual publication output; outputs of countries/regions, institutions, journals, and authors; citation frequency; and Hirsch index (H-index). The H-index, which indicates that an academic journal or scholar/country/region published H papers, each of which was cited at least H times, was used to evaluate the scientific impact of an author or a country. Journal Citation Reports (JCR) 2019 was used to obtain the impact factor (IF) and quartile of a journal category. Any disagreements were resolved by consensus. Then, the data were converted to Microsoft Excel 2019 (Redmond, Washington, USA), VOSviewer (Leiden University, Leiden, the Netherlands) and CiteSpace V (Drexel University, Philadelphia, PA, USA) for analysis of basic metrics.

Microsoft Excel (v. 2019) was applied to analyze and plot the annual publication output, H-index, total and mean IF, citations per article, and total number of citations for every country/region and to organize data on the basic characteristics of publications and citations.

VOSviewer (van Eck and Waltman, 2010) was used to create network visualization maps to analyze the collaborative relationships between countries/regions, institutions, and authors of highly cocited references. In addition, VOSviewer can classify keywords with high co-occurrence frequencies into several clusters and simultaneously color them by time course. Co-occurrence analysis identifies research hotspots and trends. We selected “author keywords” as the unit of analysis.

We used CiteSpace V to conduct cocitation analysis of the journals, references, and clusters and further constructed a timeline view of cocited references, by which we could clarify the rise and period of certain clustering fields. Furthermore, CiteSpace can capture keywords with strong citation bursts and construct visualization maps of all items. A citation burst is a key indicator for identifying emerging trends (Chen et al., 2014). We set the “years per slice” and “top N per slice” values as 1 and 50, respectively; thus, the network map was extracted from the top 50 cited papers in one year per slice.

## Results

### Publication Output and Temporal Trend

**Figure 1:**
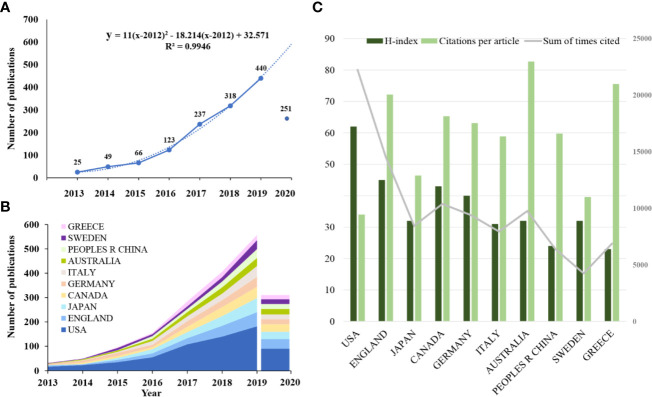
Trends in the number of publications and analysis of country/regions in SGLT2 inhibitors in cardiovascular research. **(A)** The annual worldwide publication output. **(B)** Growth trends in the publication output from the top 10 countries. **(C)** H-index, citations per article, and total number of citations for the top 10 country/regions.

A total of 1,509 publications met the inclusion criteria—813 articles and 696 reviews. The annual publication output increased steadily before 2015, increased sharply in the following 4 years and increased to 440 in 2019 to more than five times the annual publication output in 2015(66) (**Figure 1A**). A total of 251 articles have been published in 2020 to date, but this count does not reflect the number of publications during the whole year. The development trend predicts that the number of studies being carried out on SGLT2 inhibitors in CV will increase to 591.

### Distribution by Country/Region and Institution

All publications were distributed among 76 countries/regions and 2757 institutions (**Supplement Tables 1**, **2**). The USA had the highest production, with 654 (43.34%) documents, followed by England (201, 13.32%), Japan (182, 12.061%), Canada (159, 10.537%), and Germany (149, 9.874%) (**Table 1**). We further identified the annual national output of the 10 most productive countries/regions (**Figure 1B**). The USA has ranked first in the number of annual publications since 2013 and had the highest cumulative impact factor (3706.047) from 2013 to 2019. However, the mean IF in the USA was the lowest among the five most productive countries (**Supplementary Figure 1A**). England and Japan showed a similar trend in the growth rate of the annual publication output, while the annual publication output of Canada grew rapidly from 2017 and has even surpassed that of Japan to date in 2020. Additionally, Canada had the highest mean IF of 11.102 among the top 5 productive countries, although the number of cumulative publications of Canada from 2013 to 2019 was ranked fifth worldwide (127) (**Supplementary Figures 1B, C**).

**Table 1:** Top 10 productive country/regions and institutions related to SGLT2 inhibitors in cardiovascular research.

Rank	Countries/regions	Articles (N)	Percentage (N/1509)	H-index	Citations per article	Sum of times cited	Rank	Institutes	Articles (N)	Percentage (N/1509)	Location
**1**	USA	654	43.34	62	34	22233	**1**	UNIV TORONTO	118	7.82	Canada
**2**	ENGLAND	201	13.32	45	72.19	14510	**2**	ASTRAZENECA	70	4.639	England
**3**	JAPAN	182	12.061	32	46.42	8449	**3**	UNIV GRONINGEN	57	3.777	Netherlands
**4**	CANADA	159	10.537	43	65.29	10381	**4**	HARVARD MED SCH	55	3.645	USA
**5**	GERMANY	149	9.874	40	63.11	9403	**5**	BOEHRINGER INGELHEIM PHARMA GMBH CO KG	52	3.446	Germany
**6**	ITALY	135	8.946	31	58.88	7949	**6**	BRIGHAM WOMENS HOSP	45	2.982	USA
**7**	AUSTRALIA	118	7.82	32	82.66	9754	**7**	ARISTOTLE UNIV THESSALONIKI	40	2.651	Greece
**8**	PEOPLES R CHINA	107	7.091	24	59.75	6393	**8**	UNIV SYDNEY	37	2.452	Australia
**9**	SWEDEN	107	7.091	32	39.62	4239	**9**	UNIV TEXAS SOUTHWESTERN MED CTR DALLAS	37	2.452	USA
**10**	GREECE	91	6.03	23	75.56	6876	**10**	JANSSEN RES DEV LLC	36	2.386	Canada

The overall trend in the annual number of papers published by those countries exhibited a sharp increase beginning in 2016. The USA had 22,233 citations and an H-index of 62, both of which ranked first among all included countries/regions, but its citation/article ratio (34) was far less than that of Australia (82.66) and other listed countries, even the lowest-ranked country. However, Australia had a relatively low H-index (32) and number of citations (9,754) (**Figure 1C**, **Table 1**).

**Figure 2:**
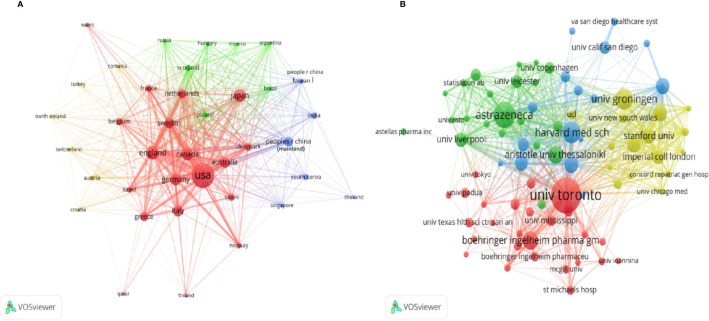
VOSviewer network visualization map of country/regions and institutions involved in SGLT2 inhibitors in cardiovascular research. **(A)** Collaboration analysis of countries/regions. Of the 76 countries/regions, 38 had at least 10 publications. **(B)** Collaboration analysis of institutions. Of the 2,757 institutions, 81 had at least 10 publications.

To investigate international collaborations, we used VOSviewer to construct a network visualization map for publications on CV research of SGLT2 inhibitors. **Figure 2A** shows collaborations among country/regions publishing more than 10 documents (38 of the 76). Country/regions with higher cooccurrence are classified as the same color. Country/regions with similar colors, which we identified as country/regions with closer cooperative relationships, formed clusters. The width of the lines represents the magnitude of the collaboration. The USA (792) had the highest total link strength, indicating that it participated in the most collaborations with other countries worldwide (**Supplement Table 3**). The countries/regions that collaborated the most with the USA were England, Sweden, Canada, Germany, Japan and Australia. The cluster indicated in blue was led by the People’s Republic of China collaborating with India, South Korea, Thailand, Australia, England, and the USA.

The 10 most productive institutions in relevant research are shown in **Table 1**. The leading institutions were the University of Toronto (118, 7.82%), Astrazeneca (70, 4.639%), University of Groningen (57, 3.777%), Harvard Medical School (55, 6.645%), and Boehringer Ingelheim Pharma GmbH & Co.KG (52, 3.446%). To reveal potential collaborations among institutions, we used VOSviewer to conduct a co-authorship analysis in terms of institutions (**Figure 2B**, **Supplement Table 4**).

### Distribution by Journal

The 1,509 publications on SGLT2 inhibitors in CV research were published in 364 academic journals. The 10 most productive and co-cited journals (at least 10 citations) are listed in **Table 2** (**Supplement Tables 5**, **6**). *Diabetes Obesity Metabolism* (112 publications, 7.422%), which had an IF of 5.9 in 2019, published the most studies in this field, followed by *Cardiovascular Diabetology* (87 publications, 5.765%), *Diabetes Therapy* (51 publications, 3.38%), *Diabetes Care* (36 publications, 2.386%), and *Circulation* (27, 1.789%), which had the highest IF in 2019 (23.603) among the 10 most productive journals. *Diabetes Care* had the highest H-index (269). More than half of the 10 most productive journals were classified in Q1 (the top 25% of the IF distribution), three were in Q2 (between the 50th percentile and 25th percentile), and only one was in Q3 (between the 75th percentile and 50th percentile). The most frequently co-cited journal in Q1 was *The New England Journal of Medicine *(8400 citations), with the highest IF in 2019 (74.699) and H-index (352). The next most frequently cocited journals were *Diabetes Care* (7534 citations), *Diabetes Obesity Metabolism* (5105 citations), *Circulation* (3469 citations), and *The Lancet* (2977 citations). All the aforementioned co-cited journals were in Q1.

**Table 2:** Top 10 productive journals and co-cited journals of SGLT2 inhibitors in CV research.

Rank	Productive Journal	Count (N)	Percentage (N/1509)	IF (2019)	H-index	quartile in category	Rank	Co-cited journal	Count (N)	IF (2019)	H-index	Best quartile
**1**	Diabetes obesity metabolism	112	7.422	5.9	116	Q1*	**1**	NEW ENGL J MED	8400	74.699	352	Q1***
**2**	Cardiovascular diabetology	87	5.765	7.332	69	Q1**	**2**	DIABETES CARE	7534	16.019	269	Q1*
**3**	Diabetes therapy	51	3.38	3.179	29	Q3*	**3**	DIABETES OBESITY METABOLISM	5105	5.9	116	Q1*
**4**	Diabetes care	36	2.386	16.019	269	Q1*	**4**	CIRCULATION	3469	23.603	165	Q1**
**5**	Circulation	27	1.789	23.603	165	Q1**	**5**	LANCET	2977	60.392	282	Q1***
**6**	Diabetologia	26	1.723	7.518	79	Q1*	**6**	DIABETOLOGIA	2285	7.518	79	Q1*
**7**	Postgraduate medicine	24	1.59	2.464	45	Q2***	**7**	DIABETES	1872	7.72	94	Q1*
**8**	Current diabetes reports	23	1.524	3.686	61	Q2*	**8**	LANCET DIABETES ENDO	1852	25.34	103	Q1*
**9**	Diabetes research and clinical practice	22	1.458	4.234	92	Q1*	**9**	CARDIOVASCULAR DIABETOLOGY	1848	7.332	47	Q1*
**10**	Expert opinion on pharmacotherapy	21	1.392	2.878	65	Q2****	**10**	JOURNAL OF THE AMERICAN COLLEGE OF CARDIOLOGY	1364	20.589	167	Q1**

*Endocrinology & Metabolism; **Cardiology & Cardiovascular System; ***Medicine, General & Internal; ****Pharmacology & Pharmacy.

### Distribution by Author

**Figure 3:**
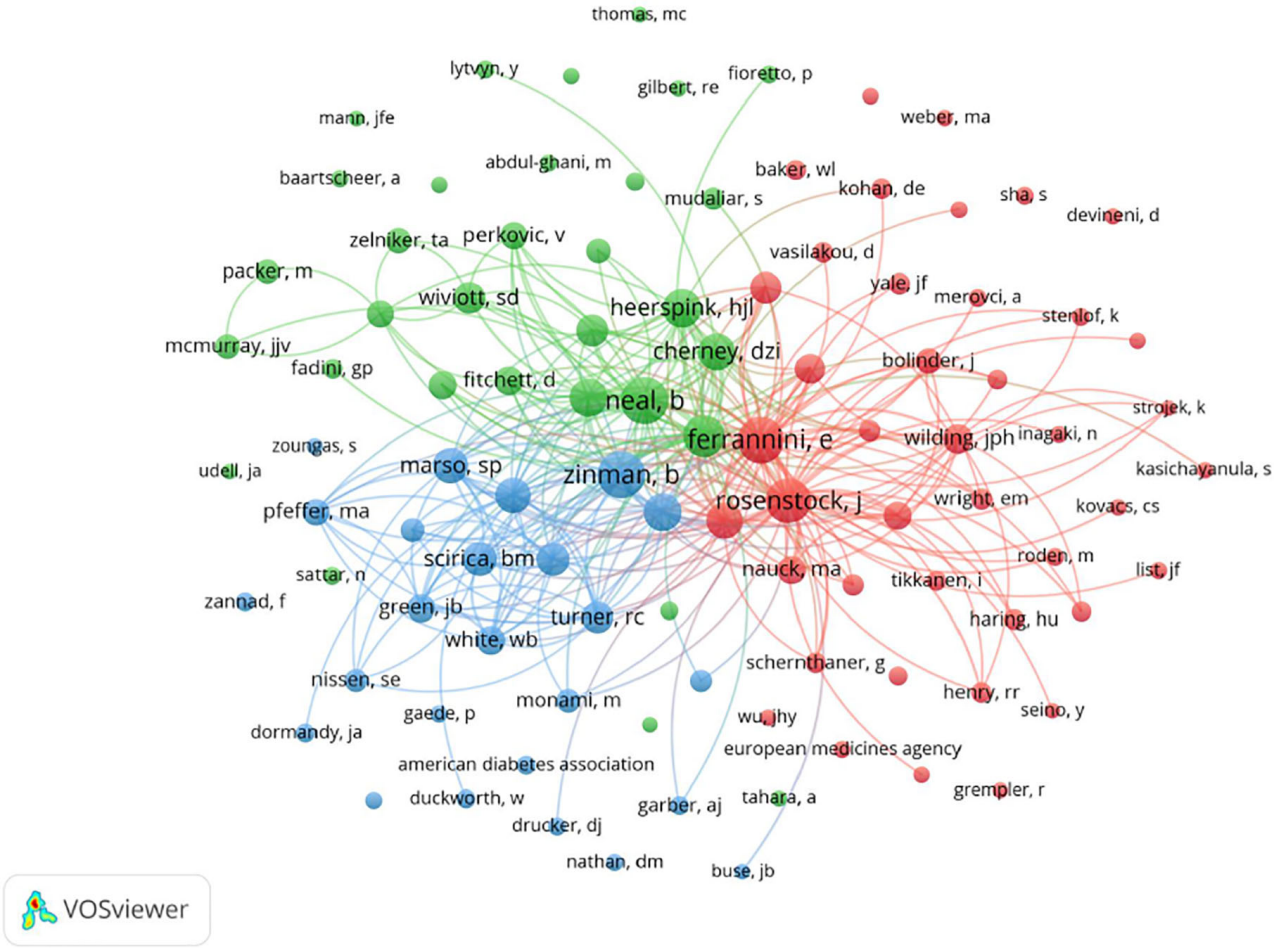
VOSviewer network visualization map of cocited authors of the articles related to SGLT2 inhibitors in cardiovascular research. Of the 23,266 cocited authors, 99 had at least 100 citations.

A total of 7357 authors contributed to all output in our study (**Supplement Table 7**). The 10 most productive authors are shown in **Table 3**. Inzucchi SE published 42 articles, ranking first in the number of publications, followed by Zinman B (34 articles), Scheen AJ (32), and Heerspink HJL (31). Mcguire DK (30), Perkovic V (30), and Verma S (30) shared fifth place. The network visualization map of the cocited authors is shown in **Figure 3**. The largest nodes are associated with the most frequently co-cited authors, including Neal B (907 citations), Zinman B (882 citations), Ferrannini E (861 citations), Rosenstock J (787 citations), and Scheen AJ (683 citations) (**Table 3**, **Supplement Table 8**). Six of the 10 most productive authors (Inzucchi SE, Zinman B, Scheen AJ, Heerspink HJL, Neal B, Wanner C) were also among the most frequently co-cited authors.

**Table 3:** Top 11 productive authors and co-cited authors in SGLT2 inhibitors in CV research.

Rank	Author	Count	Rank	Co-cited author	Citation
**1**	INZUCCHI SE	42	**1**	NEAL B	907
**2**	ZINMAN B	34	**2**	ZINMAN B	882
**3**	SCHEEN AJ	32	**3**	FERRANNINI E	861
**4**	HEERSPINK HJL	31	**4**	ROSENSTOCK J	787
**5**	MCGUIRE DK	30	**5**	SCHEEN AJ	683
**5**	PERKOVIC V	30	**6**	HEERSPINK HJL	610
**5**	VERMA S	30	**7**	WANNER C	593
**8**	WOERLE HJ	28	**8**	INZUCCHI SE	582
**9**	MAHAFFEY KW	27	**9**	CHERNEY DZI	569
**9**	NEAL B	27	**10**	DEFRONZO RA	528
**9**	WANNER C	27	**11**	MARSO SP	505

### Analysis of Co-Cited References

**Figure 4:**
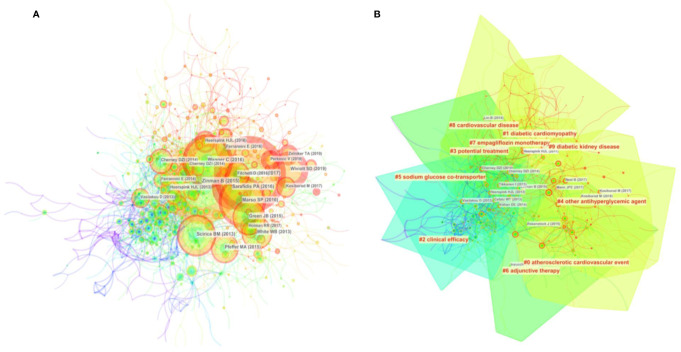
Analysis of references to SGLT2 inhibitors in cardiovascular research. **(A)** Network map of co-cited references. **(B)** Network map of co-cited clusters.

The network map of cocited references consisted of 938 references from the 50 most frequently cited references, with the time slice set as 1 year and the time span set as 2013 to 2020 (**Figure 4A**, **Supplement Table 9**). As shown in **Table 4**, all of the top 10 co-cited references were published by *The New England Journal of Medicine* (IF 2019, 74.699 and H-index, 352), including the highest-ranked publication, which was authored by Zinman et al. (2015) , with 665 citations and classified into cluster #0.

**Table 4:** Top 10 co-cited references in SGLT2 inhibitors in CV research.

Rank	co-citation counts	Centrality	Author	Year	Title	Source	Vol	page	DOI	Cluster	
1	665	0.04	Zinman B	2015	Empagliflozin, Cardiovascular Outcomes, and Mortality in Type 2 Diabetes.	NEW ENGL J MED	373	2117	10.1056/NEJMoa1504720	# 0
2	622	0.03	Neal B	2017	Canagliflozin and Cardiovascular and Renal Events in Type 2 Diabetes.	NEW ENGL J MED	377	644	10.1056/NEJMoa1611925	# 0
3	437	0.06	Wanner C	2016	Empagliflozin and Progression of Kidney Disease in Type 2 Diabetes.	NEW ENGL J MED	375	1801	10.1056/NEJMc1611290	# 0
4	416	0.04	Marso SP	2016	Liraglutide and Cardiovascular Outcomes in Type 2 Diabetes.	NEW ENGL J MED	375	311	10.1056/NEJMoa1603827	# 4
5	351	0.01	Sarafidis PA	2016	Empagliflozin, Cardiovascular Outcomes, and Mortality in Type 2 Diabetes.	NEW ENGL J MED	374	1092	10.1056/NEJMc1600827	# 0
6	345	0.03	Scirica BM	2013	Saxagliptin and cardiovascular outcomes in patients with type 2 diabetes mellitus.	NEW ENGL J MED	369	1317	10.1056/NEJMoa1307684	# 4
7	310	0.02	Wiviott SD	2019	Dapagliflozin and Cardiovascular Outcomes in Type 2 Diabetes.	NEW ENGL J MED	380	347	10.1056/NEJMoa1812389	# 0
8	293	0.01	Green JB	2015	Effect of Sitagliptin on Cardiovascular Outcomes in Type 2 Diabetes.	NEW ENGL J MED	373	232	10.1056/NEJMoa1501352	# 4
9	277	0	White WB	2013	Alogliptin after acute coronary syndrome in patients with type 2 diabetes.	NEW ENGL J MED	369	1327	10.1056/NEJMoa1305889	# 0
10	249	0.05	Pfeffer MA	2015	Lixisenatide in Patients With Type 2 Diabetes and Acute Coronary Syndrome	NEW ENGL J MED	373	2247	DOI 10.1056/NEJMoa1509225	# 0

We also constructed a network map to visualize the key clusters of cocited references (**Figure 4B**). The modularity Q score of the clustering map was 0.692, indicating the stable network structure of the map. Although the mean silhouette value of 0.2551 was relatively low, the values of the major clusters were sufficiently high (**Table 5**, **Supplement Table 10**). Cluster #0, labeling the “atherosclerotic cardiovascular event,” was the largest cluster, consisting of 91 references with a silhouette value of 0.692, followed by “diabetic cardiomyopathy” (cluster #1), “clinical efficacy” (cluster #2), and “potential treatment” (cluster #3). More than half of the top 10 co-cited references were classified into cluster #0; the rest were in cluster #4.

**Table 5:** Top 10 largest clusters of co-cited references in SGLT2 inhibitors in CV research.

Cluster ID	Size	Silhouette	Year	Top terms
# 0	91	0.692	2015	Atherosclerotic cardiovascular event
# 1	88	0.717	2017	Diabetic cardiomyopathy
# 2	75	0.634	2013	Clinical efficacy
# 3	74	0.682	2014	Potential treatment
# 4	52	0.796	2017	Other antihyperglycemic agent
# 5	51	0.638	2013	Sodium glucose co-transporter
# 6	43	0.751	2016	Adjunctive therapy
# 7	41	0.752	2015	Empagliflozin monotherapy
# 8	41	0.813	2016	Cardiovascular disease
# 9	40	0.826	2015	Diabetic kidney disease

**Figure 5:**
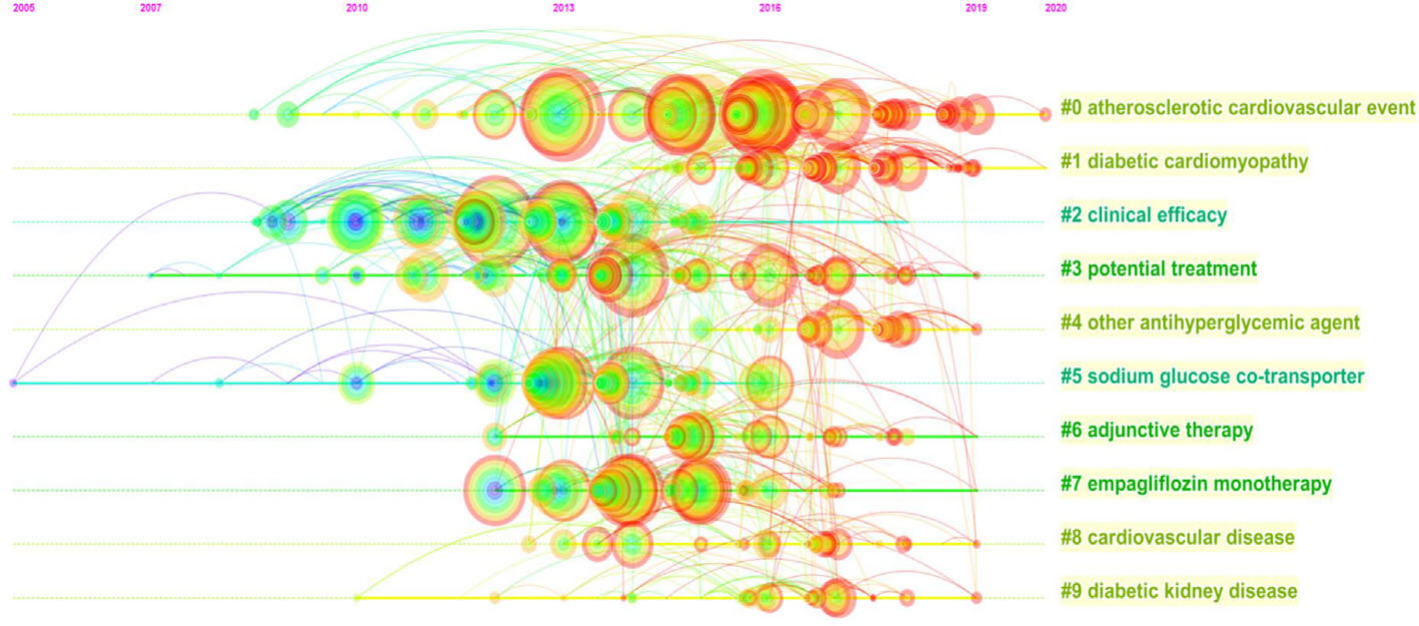
Timeline view of co-cited references related to SGLT2 inhibitors in cardiovascular research.

Furthermore, we performed a temporal cocitation analysis (**Figure 5**). Most articles were published after 2013, and the number suddenly increased in 2015, consistent with the results shown in **Figure 1A**. Clinical efficacy (cluster #2) was a relatively early research hotspot. Cluster #0 (atherosclerotic cardiovascular event), with the warmest color and the largest nodes, contained the most publications, indicating that this clustering issue is currently the primary hotspot and direction in CV research on SGLT2 inhibitors.

### Analysis of Keyword Co-Occurrence Clusters

**Figure 6:**
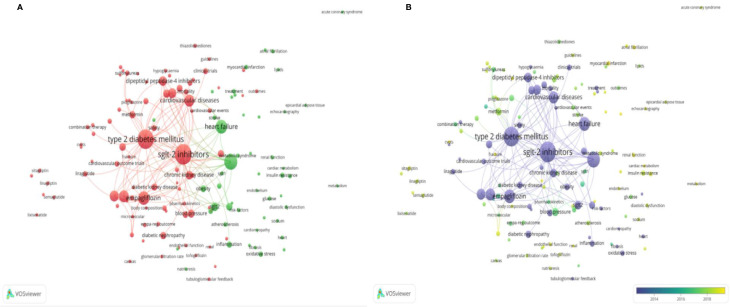
Analysis of author keywords in publications related to SGLT2 inhibitors in cardiovascular research from 2013 to 2020. **(A)** VOSviewer network visualization map of co-occurring author keywords. Of the 1,893 keywords, 141 had at least five co-occurrences. **(B)** VOSviewer overlay visualization of co-occurring author keywords by time.

We used VOSviewer to create a knowledge map of keyword co-occurrence with 141 terms (defined as terms that occurred more than 5 times) after combining synonyms into 2 clusters (**Figure 6A**, **Supplement Tables 11**,**12**): “mechanism-related research” (green) and “clinical trial-related research” (red). The size of the circles represents the keyword occurrence frequency; the larger a circle is, the more frequently do the keywords occur. **Figure 6B** shows an overlay visualization of the keywords by time. The purple, blue, green, and yellow colors on the time course correspond to the appearance of keywords over the average time, from early years to recent years. During the early stage of research on “type 2 diabetes mellitus,” “heart failure,” and “SGLT2 inhibitors” were the major topics in this field.

### Analysis of Burst Keywords

**Figure 7:**
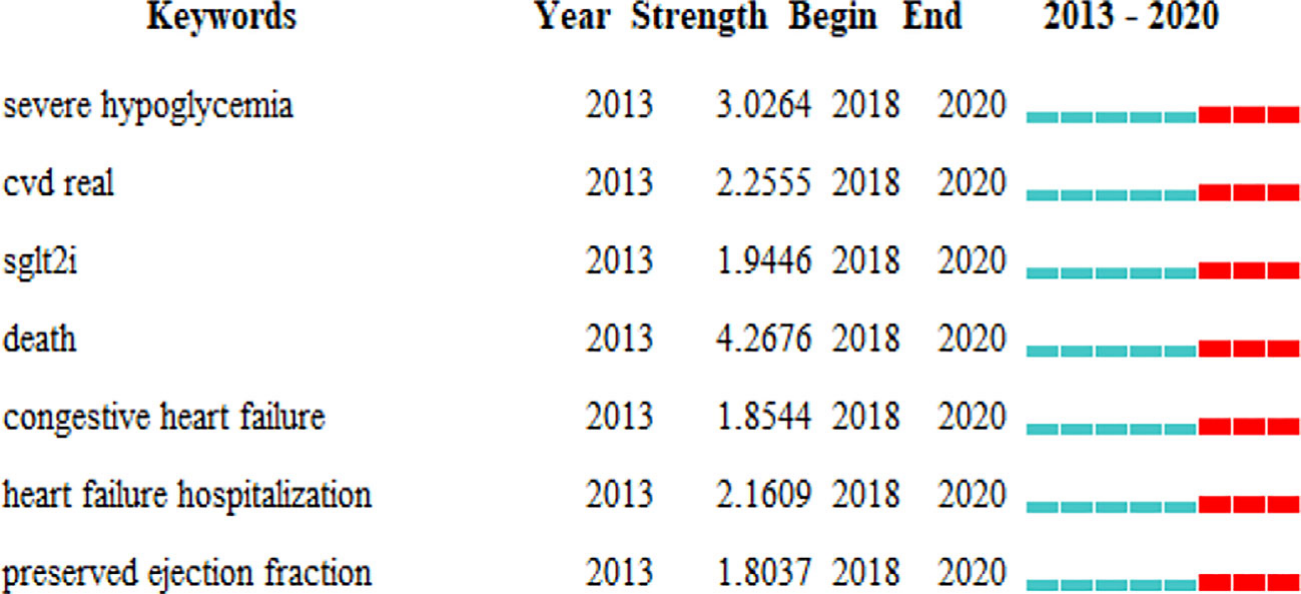
Keywords with periods of burst from 2018 onward among the top 100 burst keywords in articles related to SGLT2 inhibitors in cardiovascular research.

We used CiteSpace to detect burst keywords to determine the hotspots and research frontiers over time. Among the top 100 keywords with the strongest citation bursts in this field, we were particularly interested in those keywords that started to burst from 2018 onward (**Figure 7**), including “severe hypoglycemia” (with a burst strength of 3.0264), “CVD real” (with a burst strength of 2.2555), “death” (with a burst strength of 4.2676), “congestive heart failure” (with a burst strength of 1.8544), “heart failure hospitalization” (with a burst strength of 2.1609), and “preserved ejection fraction” (with a burst strength of 1.8037).

## Discussion

### General Information

Given that the SGLT2 inhibitor canagliflozin was first approved by the FDA in 2013 for glycemic control in patients with T2DM, we retrieved data beginning in 2013. The annual publication output increased steadily until 2015, and it then suddenly increased quickly, which was associated with the EMPA-REG OUTCOME (Zinman et al., 2015) clinical trial published in 2015, with the highest citation frequency. This trial was considered the most fundamental and important study and greatly advanced this research field. Then, the CANVAS trials (Neal et al., 2017) authored by Neal B in 2017, with the second highest citation frequency, continued to strongly drive the annual output. Furthermore, the clinical benefits of dapagliflozin in nondiabetic patients with HF were first observed in the DAPA-HF (Slomski, 2020; Vieira and Mehra, 2020) trial, which further promoted the development of this field. Thus, the publication output is expected to increase in 2020.

The earliest developments and research on selective SGLT2 inhibitors were initially conducted by American scholars (Meng et al., 2008; Dhillon, 2019), who focused on related research earlier than researchers from other regions of the world, and SGLT2 inhibitors first went public in the United States. Therefore, the USA was the main driving force and had a high academic reputation in SGLT2 inhibitor research, as evidenced by the following characteristics: number of publications, H-index value, total IF, and total number of citations. Although other countries, namely, England, Japan, Canada, and Germany, had relatively low total research production, the recent increases in their annual output reflect the considerable progress made by these countries in this field, consistent with their strong collaborations with the USA. Surprisingly, the total publications of Canada from 2013 to 2019 was the lowest among the top 5 countries, but it still had the highest mean IF, indicating that Canada performed high quality research and showed a potential important influence on future developments in this field, which merits attention. Furthermore, the University of Toronto in Canada was the most productive institution worldwide and collaborated closely with many other agencies. In addition, active collaborations were observed between American institutions, 3 of which ranked among the 10 most productive institutions, maximizing the regional advantages and further strengthening the academic impact of the USA on CV research of SGLT2 inhibitors.

Among the 10 most active journals, *Circulation* (23.603) and *Diabetes Care* (16.019) had IFs higher than 10. The aforementioned two journals were also among the leading co-cited journals, ranking fourth and second, respectively, reflecting that they have been key information resources. The core literature focused mainly on three categories—namely, cardiac & cardiovascular systems; endocrinology & metabolism; medicine, general & internal—indicating that these categories are highly recommended for tracking knowledge about SGLT2 inhibitors in CV disease. Notably, *The New England Journal of Medicine*, with the highest IF (74.699) and H-index (352), ranked first among co-cited journals, a position related to contributions of landmark clinical trials (Zinman et al., 2015; Neal et al., 2017; Vieira and Mehra, 2020); this journal was thus recognized as a basic research resource and played an important role in this research field.

### Author and Cited Reference Analysis

Four of the main members of the EMPA-REG OUTCOME trial group—Inzucchi S E (42), Zinman B (34), Woerle H J (28) and Wanner C (27)—were among the 10 most productive authors, contributing to 8.681% of the total publications in this field. Moreover, three of these prolific authors (Zinman B, Wanner C, and Inzucchi SE) were among the top 10 co-cited authors, suggesting that they considered not only the quality but also the quantity of their articles.

Among the top 10 cocited references, only one article was a comment, while nine were randomized controlled trials of SGLT2 inhibitors, glucagon-like peptide 1-receptor (GLP-1) agonists, and dipeptidyl peptidase 4 (DPP-4) inhibitors; in addition, these studies have been regarded as reliable reference resources for later related research. Notably, the EMPA-REG OUTCOME trial by Zinman B (Zinman et al., 2015) in 2015 was the most influential article, indicating striking reductions in the relative risk for CV mortality (38%), hospitalization for HF (35%), and death from any cause (32%); this trial was recognized as a critical milestone and laid a significant research foundation for the development of this domain. Wanner et al. found that empagliflozin had a good cardiorenal effect (Wanner et al., 2016). In addition, research on other kinds of SGLT2 inhibitors has been conducted: for example, the CANVAS program, conducted by Neal B et al. in 2017, showed the possible benefits of canagliflozin with respect to a lower risk of CV events and progression of albuminuria in patients with T2DM (Neal et al., 2017). In 2019, Wiviott et al. found that treatment of type 2 diabetic patients with dapagliflozin resulted in a lower rate of CV death or hospitalization for HF (Wiviott et al., 2019). However, DPP-4 inhibitors and GLP-1 were observed to be noninferior to placebo for primary composite CV outcomes and hospitalization for HF (Scirica et al., 2013; White et al., 2013; Marso et al., 2016).

As shown in the timeline view of cocited references, most studies were published after 2013 and increased in 2015, consistent with the data shown in **Figure 1A**. Cluster #0 (atherosclerotic cardiovascular event), with the warmest color and largest nodes scattered on the timeline, contained 7 of the 10 most frequently cited references, namely articles by Zinman B (2015) (Zinman et al., 2015), Sarafidis PA (Sarafidis and Tsapas, 2016), Neal B (2017) (Neal et al., 2017), Wanner C (2016) (Wanner et al., 2016), White WB (White et al., 2013), Pfeffer MA (Pfeffer et al., 2015) and Wiviott SD (2019) (Wiviott et al., 2019), who assessed the effects of SGLT2 inhibitors and GLP-1 on atherosclerotic CV outcomes in patients with T2DM. Cluster #1 (diabetic cardiomyopathy), cluster #3 (potential treatment) and cluster #4 (other antihyperglycemic agent) contained numerous hotspot nodes with red rings, meaning that they were the most recently formed clusters and the most popular research hotspots and directions. Therefore, future research should focus on the potential treatment effect of SGLT2 inhibitors and the application of SGLT2 inhibitors in diabetic cardiomyopathy; in addition, the CV effects of other antihyperglycemic agents have also been assessed, with more high-quality basic research conducted to further clarify the potential related mechanisms.

### Keyword Analysis

We used VOSviewer to analyze author keywords and visualized the two clusters, which included terms related to mechanisms and terms related to clinical trials. The potential mechanisms of SGLT2 inhibitors in DM or CV disease have been studied, Combined with the results of the overlay visualization of cooccurring author keywords and the burst keywords, the application of SGLT2 inhibitors on cardiac diastolic dysfunction and, especially, on the HFpEF was the newly evident research direction. SGLT2 inhibitors were shown to significantly improve diastolic function in the human heart, as confirmed in the myocardium of mice with or without DM and further in HFpEF models (Habibi et al., 2017; Pabel et al., 2018; Zhang et al., 2019). Substantial evidence has suggested that SGLT2 inhibitors ameliorate worsening cardiac dysfunction by reducing cardiac inflammation and oxidative stress (Ye et al., 2017; Li et al., 2019; Arow et al., 2020; Byrne et al., 2020). Although an increasing number of studies on HFpEF have been conducted (Anker et al., 2019), more in-depth research on SGLT2 inhibitors in HFpEF is expected to be conducted in the future.

In addition, SGLT2 inhibitors have significant therapeutic effects on hypertension (Park et al., 2020) and atherosclerotic disease (Nasiri-Ansari et al., 2018). Furthermore, recent studies have indicated that SGLT2 inhibitors enhance cardiac systolic function by improving myocardial energy production by directing myocardial fuel metabolism away from glucose synthesis toward ketone body (KB) formation (Ferrannini et al., 2016; Santos-Gallego et al., 2019). As the overlay visualization of co-occurring author keywords demonstrates, “cardiac metabolism” was noted as a new research hotspot and should be closely monitored.

Clinical trials of SGLT2 inhibitors have also been performed. Empagliflozin, canagliflozin, and dapagliflozin are the most extensively studied glucose-lowering drugs in CV clinical trials, becoming an active area of research in 2018. Research hotspots related to these drugs have focused on adverse CV outcomes, mainly including the risk of hypoglycemia, hospitalization for HF and CV death, consistent with the top 10 co-cited references. SGLT2 inhibitors are associated with minimal risk of hypoglycemia, particularly severe hypoglycemia, as a result of its insulin-independent mechanism of action, enabling it to be used as monotherapy or as a component of combination therapy with other antihyperglycemic agents (Frampton, 2018). The CVD-REAL trial found that, compared with therapy using other antihyperglycemic drugs, initiation of an SGLT2 inhibitor was associated with a lower risk of death and HF in patients with and without CV disease (Cavender et al., 2018). The DECLARE–TIMI 58 trial showed that treatment with dapagliflozin results in a lower rate of CV death or hospitalization for HF for patients with diabetes (Wiviott et al., 2019); the subsequent DAPA-HF trial further supplemented that the risk of worsening HF or death from CV causes was lower among patients with HFrEF than those who received dapagliflozin, regardless of the presence or absence of diabetes (McMurray et al., 2019; Slomski, 2020). The clinical evidence provides an important basis for the addition of SGLT2 inhibitors to the guideline-recommended therapies for HF.

### Limitations

To our knowledge, this bibliometric analysis is the first to explore the development and trends in CV research on SGLT2 inhibitors. However, this study had some limitations. First, the data in this study were extracted only from the WoSCC database because we consider this database a reliable service for publications and citations, although it may include fewer documents and journals than other databases, such as Google Scholar or Scopus. Second, non-English articles were excluded from the database and analysis, possibly leading to source bias. Additionally, we selectively analyzed the characteristics of the information; thus, some crucial points and details may have been overlooked.

## Conclusion

To our knowledge, our study is the first bibliometric analysis of articles focused on SGLT2 inhibitors in CV research. An increasing number of studies have been conducted to confirm the medical value of SGLT2 inhibitors in CV disease, especially since 2015. The USA has made the largest contribution to this field. The University of Toronto was the most productive institution. The most productive journal was *Diabetes Obesity Metabolism*, and the most productive author was Inzucchi Se. *The New England Journal of Medicine* was the most co-cited journal. “Atherosclerotic cardiovascular event” may be the most recent research frontier. Most studies have focused on clinical trial outcomes, such as CV death and HF hospitalization. The mechanisms of SGLT2 inhibitors, especially those related to cardiac metabolism, may soon become research hotspots and should be closely monitored.

## Data Availability Statement

All datasets presented in this study are included in the article/**Supplementary Material**.

## Author Contributions

LuC, SM, and YL designed the study. LuC and SM collected the data. LuC, DH, HL, YZ, KC, LiC, CZ, and JL analyzed the data and drafted the manuscript. YL revised and approved the final version of the manuscript.

## Funding

This work was supported by grants from the Joint Funds of the National Natural Science Foundation of China(U1908205), the Key program of Natural Science Foundation of Guangdong Province (2018B0303110008 to YL), and the Municipal Planning Project of Scientific Technology of Guangzhou (201804020083), Guangdong Province, China.

## Conflict of Interest

The authors declare that the research was conducted in the absence of any commercial or financial relationships that could be construed as a potential conflict of interest.
