# Supplementary material for: Bibliometric Study of Sodium Glucose Cotransporter 2 Inhibitors in Cardiovascular Research
Source: Front Pharmacol. 2020 Sep 15;11:561494. doi: 10.3389/fphar.2020.561494 (PMC7522576; doi:10.3389/fphar.2020.561494)
Supplement: Supplementary file 1 [file Table_1.docx]

Supplementary Material

**
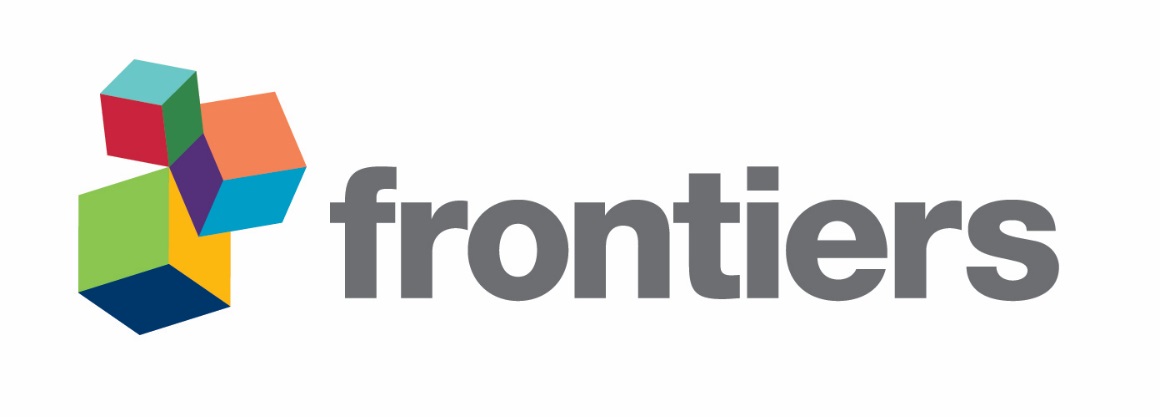
**

**Supplementary Table 1.** The country/regions that published articles of SGLT2 inhibitors in CV research.

| **Rank** | **Country/Regions** | **Count** | **% of 1509** |
| --- | --- | --- | --- |
| 1 | USA | 654 | 43.34 |
| 2 | ENGLAND | 201 | 13.32 |
| 3 | JAPAN | 182 | 12.061 |
| 4 | CANADA | 159 | 10.537 |
| 5 | GERMANY | 149 | 9.874 |
| 6 | ITALY | 135 | 8.946 |
| 7 | AUSTRALIA | 118 | 7.82 |
| 8 | PEOPLES R CHINA (MAINLAND) | 107 | 7.091 |
| 9 | SWEDEN | 107 | 7.091 |
| 10 | GREECE | 91 | 6.03 |
| 11 | NETHERLANDS | 90 | 5.964 |
| 12 | BELGIUM | 65 | 4.307 |
| 13 | DENMARK | 65 | 4.307 |
| 14 | SPAIN | 60 | 3.976 |
| 15 | FRANCE | 58 | 3.844 |
| 16 | SCOTLAND | 48 | 3.181 |
| 17 | INDIA | 47 | 3.115 |
| 18 | SOUTH KOREA | 45 | 2.982 |
| 19 | ISRAEL | 43 | 2.85 |
| 20 | NORWAY | 43 | 2.85 |
| 21 | POLAND | 35 | 2.319 |
| 22 | PEOPLES R CHINA (TAIWAN) | 35 | 2.319 |
| 23 | BRAZIL | 34 | 2.253 |
| 24 | AUSTRIA | 28 | 1.856 |
| 25 | SINGAPORE | 25 | 1.657 |
| 26 | RUSSIA | 23 | 1.524 |
| 27 | HUNGARY | 22 | 1.458 |
| 28 | ARGENTINA | 21 | 1.392 |
| 29 | MEXICO | 21 | 1.392 |
| 30 | ROMANIA | 20 | 1.325 |
| 31 | CROATIA | 19 | 1.259 |
| 32 | THAILAND | 19 | 1.259 |
| 33 | CZECH REPUBLIC | 17 | 1.127 |
| 34 | NEW ZEALAND | 17 | 1.127 |
| 35 | WALES | 16 | 1.06 |
| 36 | MALAYSIA | 15 | 0.994 |
| 37 | PORTUGAL | 15 | 0.994 |
| 38 | SWITZERLAND | 15 | 0.994 |
| 39 | FINLAND | 13 | 0.861 |
| 40 | PHILIPPINES | 13 | 0.861 |
| 41 | SOUTH AFRICA | 13 | 0.861 |
| 42 | UKRAINE | 12 | 0.795 |
| 43 | INDONESIA | 11 | 0.729 |
| 44 | COLOMBIA | 10 | 0.663 |
| 45 | NORTH IRELAND | 10 | 0.663 |
| 46 | QATAR | 10 | 0.663 |
| 47 | TURKEY | 10 | 0.663 |
| 48 | ESTONIA | 9 | 0.596 |
| 49 | GEORGIA | 9 | 0.596 |
| 50 | PERU | 9 | 0.596 |
| 51 | SRI LANKA | 9 | 0.596 |
| 52 | IRAN | 8 | 0.53 |
| 53 | IRELAND | 8 | 0.53 |
| 54 | U ARAB EMIRATES | 8 | 0.53 |
| 55 | BULGARIA | 6 | 0.398 |
| 56 | EGYPT | 6 | 0.398 |
| 57 | LEBANON | 6 | 0.398 |
| 58 | SAUDI ARABIA | 6 | 0.398 |
| 59 | SERBIA | 6 | 0.398 |
| 60 | SLOVENIA | 6 | 0.398 |
| 61 | VIETNAM | 6 | 0.398 |
| 62 | CYPRUS | 5 | 0.331 |
| 63 | SLOVAKIA | 5 | 0.331 |
| 64 | IRAQ | 3 | 0.199 |
| 65 | PAKISTAN | 3 | 0.199 |
| 66 | LATVIA | 2 | 0.133 |
| 67 | LIECHTENSTEIN | 2 | 0.133 |
| 68 | CAMEROON | 1 | 0.066 |
| 69 | CHILE | 1 | 0.066 |
| 70 | ECUADOR | 1 | 0.066 |
| 71 | JORDAN | 1 | 0.066 |
| 72 | KAZAKHSTAN | 1 | 0.066 |
| 73 | LIBYA | 1 | 0.066 |
| 74 | MYANMAR | 1 | 0.066 |
| 75 | SENEGAL | 1 | 0.066 |
| 76 | TANZANIA | 1 | 0.066 |

**Note:** SGLT2: Sodium Glucose Cotransporter 2. CV: cardiovascular
