# Supplementary material for: Bibliometric Study of Sodium Glucose Cotransporter 2 Inhibitors in Cardiovascular Research
Source: Front Pharmacol. 2020 Sep 15;11:561494. doi: 10.3389/fphar.2020.561494 (PMC7522576; doi:10.3389/fphar.2020.561494)
Supplement: Supplementary file 2 [file Table_2.docx]

Supplementary Material

**
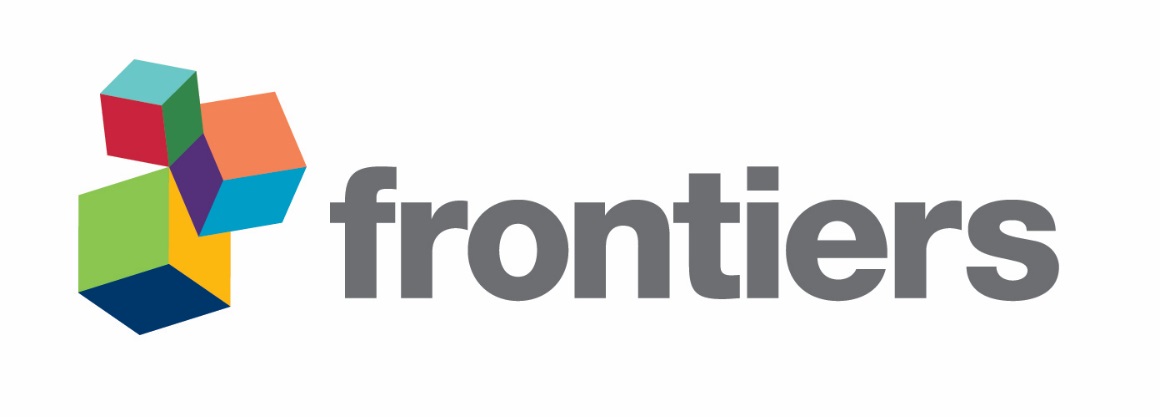
**

**Supplementary Table 2.** The institutions that published articles of SGLT2 inhibitors in CV research.

| **Rank** | **Institutions** | **count** | **% of 1509** |
| --- | --- | --- | --- |
| 1 | UNIV TORONTO | 118 | 7.82 |
| 2 | ASTRAZENECA | 70 | 4.639 |
| 3 | UNIV GRONINGEN | 57 | 3.777 |
| 4 | HARVARD MED SCH | 55 | 3.645 |
| 5 | BOEHRINGER INGELHEIM PHARMA GMBH CO KG | 52 | 3.446 |
| 6 | BRIGHAM WOMENS HOSP | 45 | 2.982 |
| 7 | ARISTOTLE UNIV THESSALONIKI | 40 | 2.651 |
| 8 | UNIV SYDNEY | 37 | 2.452 |
| 9 | UNIV TEXAS SOUTHWESTERN MED CTR DALLAS | 37 | 2.452 |
| 10 | JANSSEN RES DEV LLC | 36 | 2.386 |
| 11 | STANFORD UNIV | 34 | 2.253 |
| 12 | UNIV OXFORD | 34 | 2.253 |
| 13 | UNIV LIEGE | 32 | 2.121 |
| 14 | YALE UNIV | 32 | 2.121 |
| 15 | IMPERIAL COLL LONDON | 31 | 2.054 |
| 16 | UNIV LIVERPOOL | 30 | 1.988 |
| 17 | UNIV MISSOURI | 30 | 1.988 |
| 18 | UNIV LEICESTER | 27 | 1.789 |
| 19 | KAROLINSKA INST | 26 | 1.723 |
| 20 | UNIV CALIF SAN DIEGO | 26 | 1.723 |
| 21 | ST MICHAELS HOSP | 25 | 1.657 |
| 22 | UNIV GLASGOW | 25 | 1.657 |
| 23 | GEORGE INST GLOBAL HLTH | 24 | 1.59 |
| 24 | UNIV COLORADO | 24 | 1.59 |
| 25 | DUKE UNIV | 23 | 1.524 |
| 26 | GEORGE WASHINGTON UNIV | 23 | 1.524 |
| 27 | ST LUKES MID AMER HEART INST | 23 | 1.524 |
| 28 | UNIV COPENHAGEN | 23 | 1.524 |
| 29 | MONASH UNIV | 22 | 1.458 |
| 30 | BAYLOR UNIV | 21 | 1.392 |
| 31 | UNIV MISSISSIPPI | 21 | 1.392 |
| 32 | BOEHRINGER INGELHEIM PHARMACEUT INC | 20 | 1.325 |
| 33 | MASSACHUSETTS GEN HOSP | 20 | 1.325 |
| 34 | UNIV PADUA | 20 | 1.325 |
| 35 | BOEHRINGER INGELHEIM INT GMBH | 18 | 1.193 |
| 36 | CATHOLIC UNIV KOREA | 18 | 1.193 |
| 37 | MT SINAI HOSP | 18 | 1.193 |
| 38 | PEKING UNIV | 18 | 1.193 |
| 39 | SEMMELWEIS UNIV | 18 | 1.193 |
| 40 | UNIV TEXAS HLTH SCI CTR SAN ANTONIO | 18 | 1.193 |
| 41 | BOEHRINGER INGELHEIM NORWAY KS | 17 | 1.127 |
| 42 | STENO DIABET CTR | 17 | 1.127 |
| 43 | UNIV ATHENS | 17 | 1.127 |
| 44 | UNIV MARYLAND | 17 | 1.127 |
| 45 | UNIV NEW SOUTH WALES | 17 | 1.127 |
| 46 | MCGILL UNIV | 16 | 1.06 |
| 47 | ROYAL NORTH SHORE HOSP | 16 | 1.06 |
| 48 | TAIPEI VET GEN HOSP | 16 | 1.06 |
| 49 | UCL | 16 | 1.06 |
| 50 | WURZBURG UNIV CLIN | 16 | 1.06 |
| 51 | UNIV ALBERTA | 15 | 0.994 |
| 52 | YALE SCH MED | 15 | 0.994 |
| 53 | CHU LIEGE | 14 | 0.928 |
| 54 | MED UNIV GRAZ | 14 | 0.928 |
| 55 | SEOUL NATL UNIV | 14 | 0.928 |
| 56 | UNIV N CAROLINA | 14 | 0.928 |
| 57 | VA SAN DIEGO HEALTHCARE SYST | 14 | 0.928 |
| 58 | ASTRAZENECA GOTHENBURG | 13 | 0.861 |
| 59 | JANSSEN SCI AFFAIRS LLC | 13 | 0.861 |
| 60 | NYU | 13 | 0.861 |
| 61 | STATISTICON AB | 13 | 0.861 |
| 62 | ASTELLAS PHARMA INC | 12 | 0.795 |
| 63 | GEORGETOWN UNIV | 12 | 0.795 |
| 64 | HADASSAH HEBREW UNIV HOSP | 12 | 0.795 |
| 65 | JIKEI UNIV | 12 | 0.795 |
| 66 | LOUISIANA STATE UNIV | 12 | 0.795 |
| 67 | NATL HEART CTR | 12 | 0.795 |
| 68 | OSLO UNIV HOSP | 12 | 0.795 |
| 69 | SEOUL NATL UNIV HOSP | 12 | 0.795 |
| 70 | SWANSEA UNIV | 12 | 0.795 |
| 71 | TORONTO GEN HOSP | 12 | 0.795 |
| 72 | UNIV MIAMI | 12 | 0.795 |
| 73 | UNIV PISA | 12 | 0.795 |
| 74 | UNIV TOKYO | 12 | 0.795 |
| 75 | UNIV UTAH | 12 | 0.795 |
| 76 | WROCLAW MED UNIV | 12 | 0.795 |
| 77 | APEX MED RES | 11 | 0.729 |
| 78 | BOEHRINGER INGELHEIM GMBH CO KG | 11 | 0.729 |
| 79 | CONCORD REPATRIAT GEN HOSP | 11 | 0.729 |
| 80 | EMORY UNIV | 11 | 0.729 |
| 81 | HOSP 12 OCTUBRE | 11 | 0.729 |
| 82 | HOSP CLIN BARCELONA | 11 | 0.729 |
| 83 | HOSP VALLE DE HEBRON | 11 | 0.729 |
| 84 | KAWASAKI MED SCH | 11 | 0.729 |
| 85 | KYOTO UNIV | 11 | 0.729 |
| 86 | LMC DIABET ENDOCRINOL | 11 | 0.729 |
| 87 | MANIPAL HOSP | 11 | 0.729 |
| 88 | MEDSTAR HLTH RES INST | 11 | 0.729 |
| 89 | NATL CHENG KUNG UNIV HOSP | 11 | 0.729 |
| 90 | NATL TAIWAN UNIV HOSP | 11 | 0.729 |
| 91 | ROYAL BRISBANE WOMENS HOSP | 11 | 0.729 |
| 92 | SINGAPORE GEN HOSP | 11 | 0.729 |
| 93 | TULANE UNIV | 11 | 0.729 |
| 94 | UNIV BRITISH COLUMBIA | 11 | 0.729 |
| 95 | UNIV CHICAGO MED | 11 | 0.729 |
| 96 | UNIV IOANNINA | 11 | 0.729 |
| 97 | UNIV MED CTR GRONINGEN | 11 | 0.729 |
| 98 | UNIV OSLO | 11 | 0.729 |
| 99 | UPPSALA UNIV | 11 | 0.729 |
| 100 | BAKER HEART DIABET INST | 10 | 0.663 |
| 101 | BAYLOR HEART VASC INST | 10 | 0.663 |
| 102 | BNAI ZION MED CTR | 10 | 0.663 |
| 103 | CHANG GUNG MEM HOSP | 10 | 0.663 |
| 104 | CHARITE UNIV MED BERLIN | 10 | 0.663 |
| 105 | CHINA MED UNIV HOSP | 10 | 0.663 |
| 106 | CHONNAM NATL UNIV HOSP | 10 | 0.663 |
| 107 | CHU SART TILMAN B35 | 10 | 0.663 |
| 108 | CHUNG SHAN MED UNIV HOSP | 10 | 0.663 |
| 109 | CLIN HOSP CTR OSIJEK | 10 | 0.663 |
| 110 | CLIN UNIV ST LUC | 10 | 0.663 |
| 111 | CRU HUNGARY KFT | 10 | 0.663 |
| 112 | CTR HOSP LISBOA OCIDENTAL | 10 | 0.663 |
| 113 | CTR HOSP VILA NOVA DE GAIA ESPINHO | 10 | 0.663 |
| 114 | CTR SALUD LAVAPIES | 10 | 0.663 |
| 115 | DIABET AMER | 10 | 0.663 |
| 116 | FOUR RIVERS CLIN RES | 10 | 0.663 |
| 117 | HEBREW UNIV JERUSALEM | 10 | 0.663 |
| 118 | HOP HOTEL DIEU | 10 | 0.663 |
| 119 | HOSP GEN SEGOVIA | 10 | 0.663 |
| 120 | HOSP UNIV COIMBRA | 10 | 0.663 |
| 121 | HOSP UNIV RAMON Y CAJAL | 10 | 0.663 |
| 122 | IMPERIAL COLL | 10 | 0.663 |
| 123 | KAOHSIUNG MED UNIV HOSP | 10 | 0.663 |
| 124 | KHOO TECK PUAT HOSP | 10 | 0.663 |
| 125 | KINGS COLL LONDON | 10 | 0.663 |
| 126 | KRANKENANSTALT RUDOLFSTIFTUNG WIEN | 10 | 0.663 |
| 127 | MAINE RES ASSOCIATES | 10 | 0.663 |
| 128 | NATL CEREBRAL CARDIOVASC CTR | 10 | 0.663 |
| 129 | NATL HLTH INSURANCE SERV ILSAN HOSP | 10 | 0.663 |
| 130 | NHS TAYSIDE | 10 | 0.663 |
| 131 | QEII HLTH SCI CTR | 10 | 0.663 |
| 132 | QUEEN ELIZABETH HOSP | 10 | 0.663 |
| 133 | QUEEN MARY HOSP | 10 | 0.663 |
| 134 | RAJAVITHI HOSP | 10 | 0.663 |
| 135 | ROYAL PRINCE ALFRED HOSP | 10 | 0.663 |
| 136 | STENO DIABET CTR COPENHAGEN | 10 | 0.663 |
| 137 | UNION MEM HOSP | 10 | 0.663 |
| 138 | UNIV CONNECTICUT | 10 | 0.663 |
| 139 | UNIV HELSINKI | 10 | 0.663 |
| 140 | UNIV HOSP CTR SESTRE MILOSRDNICE | 10 | 0.663 |
| 141 | UNIV SAO PAULO | 10 | 0.663 |
| 142 | UNIV TURIN | 10 | 0.663 |
| 143 | UNIV WASHINGTON | 10 | 0.663 |
| 144 | UNSW SYDNEY | 10 | 0.663 |
| 145 | ZIEKENHUIS OOST LIMBURG | 10 | 0.663 |
| 146 | A O CAREGGI | 9 | 0.596 |
| 147 | A O MATER DOMINI | 9 | 0.596 |
| 148 | A R RES GRP LLC | 9 | 0.596 |
| 149 | ADELAIDE MED RES | 9 | 0.596 |
| 150 | ADV RES INST | 9 | 0.596 |
| 151 | AJOU UNIV HOSP | 9 | 0.596 |
| 152 | ALABAMA CLIN THERAPEUT LLC | 9 | 0.596 |
| 153 | ALBUQUERQUE CLIN TRIALS | 9 | 0.596 |
| 154 | ALICE HO MIU LING NETHERSOLE HOSP | 9 | 0.596 |
| 155 | ALL MED RES LLC | 9 | 0.596 |
| 156 | ALTAI STATE MED UNIV | 9 | 0.596 |
| 157 | ALZOHAILI MED CONSULTANTS | 9 | 0.596 |
| 158 | AMANG RODRIGUEZ MED CTR | 9 | 0.596 |
| 159 | AP BADIA DEL VALLES | 9 | 0.596 |
| 160 | APDP | 9 | 0.596 |
| 161 | AREA ENDOCRINOL | 9 | 0.596 |
| 162 | ARKANSAS PRIMARY CARE CLIN | 9 | 0.596 |
| 163 | ASAN MED CTR | 9 | 0.596 |
| 164 | ASSAF HAROFEH MED CTR | 9 | 0.596 |
| 165 | ATLANTA CLIN RES CTR | 9 | 0.596 |
| 166 | ATLANTIC CLIN TRIALS LLC | 9 | 0.596 |
| 167 | ATRIUM MED CTR | 9 | 0.596 |
| 168 | AUCKLAND CITY HOSP | 9 | 0.596 |
| 169 | AUTONOMOUS NONPROFIT ORG | 9 | 0.596 |
| 170 | AVINGTON MEM HOSP | 9 | 0.596 |
| 171 | AZIENDA OSPED PADOVA | 9 | 0.596 |
| 172 | BANGALORE CLINISEARCH | 9 | 0.596 |
| 173 | BAYLOR COLL MED | 9 | 0.596 |
| 174 | BAYSTATE MED CTR | 9 | 0.596 |
| 175 | BEKES CTY PANDY KALMAN HOSP | 9 | 0.596 |
| 176 | BENEFIS HLTH GRP | 9 | 0.596 |
| 177 | BERMA RES GRP | 9 | 0.596 |
| 178 | BETHESDA ZIEKENHUIS | 9 | 0.596 |
| 179 | BISPEBJERG HOSP | 9 | 0.596 |
| 180 | BLUMENAU SERV MED SC LTDA | 9 | 0.596 |
| 181 | BUDDHIST TZU CHI GEN HOSP | 9 | 0.596 |
| 182 | BURKE INTERNAL MED RES | 9 | 0.596 |
| 183 | BUYNAK CLIN RES | 9 | 0.596 |
| 184 | CABINET MED | 9 | 0.596 |
| 185 | CALABASH MED CTR | 9 | 0.596 |
| 186 | CALIF RES FDN | 9 | 0.596 |
| 187 | CARDINAL SANTOS MED CTR | 9 | 0.596 |
| 188 | CARDIOL CLIN RES | 9 | 0.596 |
| 189 | CARDIOL RES CLIN | 9 | 0.596 |
| 190 | CARDIOLAB LTDA | 9 | 0.596 |
| 191 | CARL EDYTH LINDER CTR RES EDUC | 9 | 0.596 |
| 192 | CAROLINA HLTH SPECIALISTS | 9 | 0.596 |
| 193 | CASA DIABET NUTR | 9 | 0.596 |
| 194 | CASE WESTERN RESERVE UNIV | 9 | 0.596 |
| 195 | CCBR CZECH PRAGUE SRO | 9 | 0.596 |
| 196 | CEBU DOCTORS UNIV HOSP | 9 | 0.596 |
| 197 | CED CTR ENDOCRINOL DIABET | 9 | 0.596 |
| 198 | CEE RES KFT | 9 | 0.596 |
| 199 | CEMDE | 9 | 0.596 |
| 200 | CENT CLIN HOSP 2 | 9 | 0.596 |
| 201 | CEQUIN | 9 | 0.596 |
| 202 | CH NARBONNE | 9 | 0.596 |
| 203 | CHARLESTON INTERNAL MED RES INST | 9 | 0.596 |
| 204 | CHASE MED RES LLC | 9 | 0.596 |
| 205 | CHATSWORTH UNIT 10 | 9 | 0.596 |
| 206 | CHEMOTHERAPY IMMUNOTHERAPY CLIN MEDULLA | 9 | 0.596 |
| 207 | CHIBANISHI GEN HOSP | 9 | 0.596 |
| 208 | CHR CITADELLE | 9 | 0.596 |
| 209 | CHR HUY | 9 | 0.596 |
| 210 | CHRISTCHURCH HOSP CAMPUS | 9 | 0.596 |
| 211 | CHU GRENOBLE | 9 | 0.596 |
| 212 | CHU SART TILMAN | 9 | 0.596 |
| 213 | CHU SUD | 9 | 0.596 |
| 214 | CHU TIVOLI | 9 | 0.596 |
| 215 | CHU TOURS | 9 | 0.596 |
| 216 | CHUNGNAM NATL UNIV HOSP | 9 | 0.596 |
| 217 | CINERE HOSP | 9 | 0.596 |
| 218 | CIPTO MANGUNKUSUMO HOSP | 9 | 0.596 |
| 219 | CITOMED SP ZOO | 9 | 0.596 |
| 220 | CITY ALEXANDER HOSP ST PETERSBURG | 9 | 0.596 |
| 221 | CITY CLIN HOSP 1 | 9 | 0.596 |
| 222 | CITY CLIN HOSP 19 | 9 | 0.596 |
| 223 | CITY CLIN HOSP 4 | 9 | 0.596 |
| 224 | CITY CLIN HOSP 67 | 9 | 0.596 |
| 225 | CITY CLIN HOSP 8 | 9 | 0.596 |
| 226 | CITY CLIN HOSP EMERGENCY CARE | 9 | 0.596 |
| 227 | CITY HOSP 17 | 9 | 0.596 |
| 228 | CITY POLYCLIN 120 | 9 | 0.596 |
| 229 | CLEVELAND CLIN | 9 | 0.596 |
| 230 | CLIN CARDIOL SERTSE I SUDYNY LTD | 9 | 0.596 |
| 231 | CLIN HORIKAWA | 9 | 0.596 |
| 232 | CLIN HOSP 5 | 9 | 0.596 |
| 233 | CLIN INMACULADA CONCEPC | 9 | 0.596 |
| 234 | CLIN MED SAN MARTIN | 9 | 0.596 |
| 235 | CLIN PRIVADA COLOMBO | 9 | 0.596 |
| 236 | CLIN PROJECTS RES | 9 | 0.596 |
| 237 | CLIN RES ADV INC | 9 | 0.596 |
| 238 | CLIN RES CONSULTANTS LLC | 9 | 0.596 |
| 239 | CLIN RES INC | 9 | 0.596 |
| 240 | CLIN RES INST | 9 | 0.596 |
| 241 | CLINFAN SMO LTD | 9 | 0.596 |
| 242 | CLINRESCO CTR PTY LTD | 9 | 0.596 |
| 243 | COASTAL CAROLINA RES CTR | 9 | 0.596 |
| 244 | COASTAL NEPHROL ASSOCIATES RES CTR LLC | 9 | 0.596 |
| 245 | COLOMBO NORTH TEACHING HOSP | 9 | 0.596 |
| 246 | COMMONWEALTH BIOMED RES LLC | 9 | 0.596 |
| 247 | COMMUNITY RES SOUTH FLORIDA | 9 | 0.596 |
| 248 | COMPASS RES | 9 | 0.596 |
| 249 | CONSANO CLIN RES | 9 | 0.596 |
| 250 | CONSULTORIOS ASOCIADOS ENDOCRINOL INVEST CLIN | 9 | 0.596 |
| 251 | COOK CTY HOSP | 9 | 0.596 |
| 252 | CORP SANITARIA PARC TAULI | 9 | 0.596 |
| 253 | CREIGHTON UNIV | 9 | 0.596 |
| 254 | CTR DIABET ENDOCRINE CARE | 9 | 0.596 |
| 255 | CTR DIABET ENDOCRINOL | 9 | 0.596 |
| 256 | CTR DIABETOL DR WAITMAN | 9 | 0.596 |
| 257 | CTR ESPECIALIZADO DIABET | 9 | 0.596 |
| 258 | CTR HLTH | 9 | 0.596 |
| 259 | CTR HOSP GEN SUD FRANCILIEN | 9 | 0.596 |
| 260 | CTR HOSP LISBOA CENT | 9 | 0.596 |
| 261 | CTR HOSP UNIV LIEGE | 9 | 0.596 |
| 262 | CTR INVEST HEART HELP | 9 | 0.596 |
| 263 | CTR MED ALTA COMPLEJIDAD | 9 | 0.596 |
| 264 | CTR MED COLON | 9 | 0.596 |
| 265 | CTR MED VIAMONTE | 9 | 0.596 |
| 266 | CTR PESQUISA CLIN BRASIL | 9 | 0.596 |
| 267 | CTR PESQUISAS CLIN LTDA | 9 | 0.596 |
| 268 | CTR PESQUISAS DIABET | 9 | 0.596 |
| 269 | CTR PESQUISAS DIABET DOENCAS | 9 | 0.596 |
| 270 | CTR SALUD LA ALAMEDILLA | 9 | 0.596 |
| 271 | CTR SALUD MARIA JESUS HEREZA | 9 | 0.596 |
| 272 | CTR SALUD PEDRO LAIN ENTRALGO | 9 | 0.596 |
| 273 | CTR SCOMPENSO CARDIACO | 9 | 0.596 |
| 274 | CTY CLIN HOSP | 9 | 0.596 |
| 275 | CU PHARMACEUT RES | 9 | 0.596 |
| 276 | DAISHINKAI MED CORP OOKUMA HOSP | 9 | 0.596 |
| 277 | DALLAS DIABET ENDOCRINE CTR | 9 | 0.596 |
| 278 | DAVID METREVELI MED CTR LTD | 9 | 0.596 |
| 279 | DAYTON CLIN RES | 9 | 0.596 |
| 280 | DE LA SALLE UNIV MED CTR | 9 | 0.596 |
| 281 | DEENDAYAL MEM HOSP | 9 | 0.596 |
| 282 | DELAWARE RES | 9 | 0.596 |
| 283 | DELTA WAVES INC | 9 | 0.596 |
| 284 | DESERT OASIS HEALTHCARE | 9 | 0.596 |
| 285 | DEXA DIAB IPS | 9 | 0.596 |
| 286 | DIABET CARE N RES CTR | 9 | 0.596 |
| 287 | DIABET CLIN | 9 | 0.596 |
| 288 | DIABET ENDOCRINE SPECIALISTS INC | 9 | 0.596 |
| 289 | DIABET ENDOCRINE UNIT | 9 | 0.596 |
| 290 | DIACON HOSP RES CTR | 9 | 0.596 |
| 291 | DIACONESSENHUIS MEPPEL | 9 | 0.596 |
| 292 | DIPARTIMENTO CLIN TERAPIA MED | 9 | 0.596 |
| 293 | DIPARTIMENTO MED INTERNA | 9 | 0.596 |
| 294 | DIPARTIMENTO SCI MED | 9 | 0.596 |
| 295 | DIV CARDIOL | 9 | 0.596 |
| 296 | DJW NAVORSING | 9 | 0.596 |
| 297 | DORMIR CLIN TRIALS INC | 9 | 0.596 |
| 298 | DR MOHANS DIABET SPECIAL | 9 | 0.596 |
| 299 | DR MOODLEY DR SARVAN | 9 | 0.596 |
| 300 | DR RAMESH CARDIAC MULTISPECIAL HOSP LTD | 9 | 0.596 |
| 301 | DR U GOVIND | 9 | 0.596 |
| 302 | E DA HOSP | 9 | 0.596 |
| 303 | E WOLFSON MED CTR | 9 | 0.596 |
| 304 | EAST TALLINN CENT HOSP | 9 | 0.596 |
| 305 | EAST WEST MED RES | 9 | 0.596 |
| 306 | ENDOCRINE ASSOCIATES LONG ISL PC | 9 | 0.596 |
| 307 | ENDOCRINE DIABET CARE RESOURCE CTR | 9 | 0.596 |
| 308 | ENDOCRINE RES SOLUT INC | 9 | 0.596 |
| 309 | ENDOCRINOL ASSOCIATES | 9 | 0.596 |
| 310 | EPE | 9 | 0.596 |
| 311 | ETTRICK HLTH CTR PA | 9 | 0.596 |
| 312 | EULJI UNIV HOSP | 9 | 0.596 |
| 313 | FAMILY MED SAYEBROOK | 9 | 0.596 |
| 314 | FAY WEST FAMILY PRACTICE | 9 | 0.596 |
| 315 | FDN CARIBE INVESTIGAC BIOMED | 9 | 0.596 |
| 316 | FDN OFTALMOLOG SANTANDER | 9 | 0.596 |
| 317 | FDN UNIV G DANNUNZIO | 9 | 0.596 |
| 318 | FEPREVA | 9 | 0.596 |
| 319 | FLORIDA INST CLIN RES | 9 | 0.596 |
| 320 | FMUSP | 9 | 0.596 |
| 321 | FOCUS CLIN RES | 9 | 0.596 |
| 322 | FORSKNINGSCTR NORDSJAELLANDS HOSP | 9 | 0.596 |
| 323 | FOWEY RIVER PRACTICE | 9 | 0.596 |
| 324 | FREDERIKSBERG UNIV HOSP | 9 | 0.596 |
| 325 | FRESNO INC | 9 | 0.596 |
| 326 | FUJIKOSHI HOSP | 9 | 0.596 |
| 327 | FUJITA HLTH UNIV HOSP | 9 | 0.596 |
| 328 | GACHON UNIV GIL MED CTR | 9 | 0.596 |
| 329 | GELRE ZIEKENHUIZEN APELDOORN | 9 | 0.596 |
| 330 | GELRE ZIEKENHUIZEN LOCATIE ZUTPHEN | 9 | 0.596 |
| 331 | GEMINI SCI LLC | 9 | 0.596 |
| 332 | GEMINI ZIEKENHUIS | 9 | 0.596 |
| 333 | GEN HOSP ATHENS G GENNIMATAS | 9 | 0.596 |
| 334 | GEN HOSP ATHENS LAIKO | 9 | 0.596 |
| 335 | GEN HOSP ATHENS POLIKLINIKI | 9 | 0.596 |
| 336 | GEN HOSP KARLOVAC | 9 | 0.596 |
| 337 | GEN HOSP LARISSA | 9 | 0.596 |
| 338 | GEN HOSP NIKAIA | 9 | 0.596 |
| 339 | GEN HOSP PAPAGEORGIOU | 9 | 0.596 |
| 340 | GEN HOSP SVETI DUH | 9 | 0.596 |
| 341 | GEN HOSP THESSALONIKI G PAPANIKOLAOU | 9 | 0.596 |
| 342 | GENESIS CLIN RES | 9 | 0.596 |
| 343 | GENESIS RES INT | 9 | 0.596 |
| 344 | GIFU HEART CTR | 9 | 0.596 |
| 345 | GREAT FALLS CLIN LLP | 9 | 0.596 |
| 346 | GREEN CLIN PC | 9 | 0.596 |
| 347 | GREEN SEIDNER FAMILY PRACTICE | 9 | 0.596 |
| 348 | GREENACRES HOSP | 9 | 0.596 |
| 349 | GULFCOAST ENDOCRINE DIABET CTR | 9 | 0.596 |
| 350 | HAEMEK MED CTR | 9 | 0.596 |
| 351 | HANYU GEN HOSP | 9 | 0.596 |
| 352 | HARAPAN KITA NATL CARDIOVASC CTR | 9 | 0.596 |
| 353 | HARRISBURG FAMILY MED CTR | 9 | 0.596 |
| 354 | HEART VASC INST | 9 | 0.596 |
| 355 | HEART VESSELS DIS COMPLEX PROBLEMS | 9 | 0.596 |
| 356 | HERITAGE HOSP LTD | 9 | 0.596 |
| 357 | HIGH DESERT MED GRP | 9 | 0.596 |
| 358 | HIGHLAND DIABET INST | 9 | 0.596 |
| 359 | HILL CTY MED ASSOCIATES | 9 | 0.596 |
| 360 | HILLEL YAFFE MED CTR | 9 | 0.596 |
| 361 | HOLSTON MED GRP | 9 | 0.596 |
| 362 | HOP CARDIOL HAUT LEVEQUE | 9 | 0.596 |
| 363 | HOP NORD | 9 | 0.596 |
| 364 | HOP POITIERS | 9 | 0.596 |
| 365 | HOP ST LOUIS | 9 | 0.596 |
| 366 | HOP UNIV ERASME | 9 | 0.596 |
| 367 | HORIZAN RES GRP INC | 9 | 0.596 |
| 368 | HORIZON CLIN RES ASSOCIATES | 9 | 0.596 |
| 369 | HOSP ALBERTO SABOGAL SOLOGUREN | 9 | 0.596 |
| 370 | HOSP CLIN | 9 | 0.596 |
| 371 | HOSP CLIN UNIV SAN CARLOS | 9 | 0.596 |
| 372 | HOSP DR ANGEL LEANO | 9 | 0.596 |
| 373 | HOSP FERNANDO FONSECA | 9 | 0.596 |
| 374 | HOSP GEN UNIV | 9 | 0.596 |
| 375 | HOSP GERAL GOIANIA | 9 | 0.596 |
| 376 | HOSP GUILHERME ALVARO | 9 | 0.596 |
| 377 | HOSP INFANTA LEONOR | 9 | 0.596 |
| 378 | HOSP JINDRICHUV HRADEC | 9 | 0.596 |
| 379 | HOSP LUZ | 9 | 0.596 |
| 380 | HOSP MAE DEUS | 9 | 0.596 |
| 381 | HOSP NACL ARZOBISPO LOAYZA | 9 | 0.596 |
| 382 | HOSP NACL DANIEL ALCIDES CARRION | 9 | 0.596 |
| 383 | HOSP NACL EDGARDO REBAGLIATI MARTINS | 9 | 0.596 |
| 384 | HOSP NACL HIPOLITO UNANUE | 9 | 0.596 |
| 385 | HOSP NUESTRA SENORA DE LA CANDELARIA | 9 | 0.596 |
| 386 | HOSP NUESTRA SENORA DE SONSOLES | 9 | 0.596 |
| 387 | HOSP PRIVADO CTR MED CORDOBA SA | 9 | 0.596 |
| 388 | HOSP RIBERA | 9 | 0.596 |
| 389 | HOSP RIM HIPERTENSAO | 9 | 0.596 |
| 390 | HOSP SAO JOAO | 9 | 0.596 |
| 391 | HOSP SAO PAULO UNIFESP | 9 | 0.596 |
| 392 | HOSP SELAYANG | 9 | 0.596 |
| 393 | HOSP SON ESPASES | 9 | 0.596 |
| 394 | HOSP SULTANAH AMINAH | 9 | 0.596 |
| 395 | HOSP SULTANAH BAHIYAH | 9 | 0.596 |
| 396 | HOSP TENGKU AMPUAN AFZAN | 9 | 0.596 |
| 397 | HOSP UNIV JOAO BARROS BARRETO | 9 | 0.596 |
| 398 | HOSP UNIV LA PRINCESA | 9 | 0.596 |
| 399 | HOSP UNIV NUEVO LEON | 9 | 0.596 |
| 400 | HOSP UNIV PUERTA DE HIERRO | 9 | 0.596 |
| 401 | HOSP UNIV SAINS MALAYSIA | 9 | 0.596 |
| 402 | HOWARD KERSTEIN MD | 9 | 0.596 |
| 403 | HUISARTSENMAATSCHAP LSV | 9 | 0.596 |
| 404 | HUISARTSENPRAKTIJK DEKELVER | 9 | 0.596 |
| 405 | HUISARTSENPRAKTIJK HOOGE BOOM | 9 | 0.596 |
| 406 | HUISARTSENPRAKTIJK HYGEIA | 9 | 0.596 |
| 407 | HUISARTSENPRAKTIJK WILDERVANK | 9 | 0.596 |
| 408 | ICAHN SCH MED MT SINAI | 9 | 0.596 |
| 409 | ICLE SC | 9 | 0.596 |
| 410 | ILAIMCEOM | 9 | 0.596 |
| 411 | ILUMINA CLIN ASSOCIATES | 9 | 0.596 |
| 412 | IMC | 9 | 0.596 |
| 413 | IMELDA ZH BONHEIDEN | 9 | 0.596 |
| 414 | INJE UNIV HAEUNDAE PAIK HOSP | 9 | 0.596 |
| 415 | INNOVAT CLIN RES INC | 9 | 0.596 |
| 416 | INST ADULT DIS | 9 | 0.596 |
| 417 | INST CIENCIAS MED | 9 | 0.596 |
| 418 | INST CORACAO | 9 | 0.596 |
| 419 | INST DANTE PAZZANESE CARDIOL | 9 | 0.596 |
| 420 | INST DELGADO INVEST MED | 9 | 0.596 |
| 421 | INST DIABET NUTR METAB DIS | 9 | 0.596 |
| 422 | INST ENDOCRINOL FARFAN | 9 | 0.596 |
| 423 | INST INVEST CLIN | 9 | 0.596 |
| 424 | INST MED MIRAFLORES | 9 | 0.596 |
| 425 | INST NACL CIENCIAS MED NUTR SALVADOR ZUBIRAN | 9 | 0.596 |
| 426 | INST PESQUISA CLIN MED AVANCADA | 9 | 0.596 |
| 427 | INT DIABET CTR | 9 | 0.596 |
| 428 | INT RES ASSOCIATES LLC | 9 | 0.596 |
| 429 | INTEGRATED MED GRP PC | 9 | 0.596 |
| 430 | INTEGRATED RES CTR | 9 | 0.596 |
| 431 | INTEGRATED RES GRP INC | 9 | 0.596 |
| 432 | INTERN MED | 9 | 0.596 |
| 433 | INTERNAL MED ASSOCIATES ANDERSON PA | 9 | 0.596 |
| 434 | IRMANDADE SANTA CASA MISERICORDIA PORTO ALEGRE | 9 | 0.596 |
| 435 | IRMANDADE SANTA CASA MISERICORDIA SAO PAULO | 9 | 0.596 |
| 436 | IRYOUHOUIJNEIWAKAI MINAMIAKATSUKA CLIN | 9 | 0.596 |
| 437 | JEFFERSON CARDIOL ASSOC | 9 | 0.596 |
| 438 | JEFFRY LINDENBAUM DO PC | 9 | 0.596 |
| 439 | JINNOUCHI DIABET CTR | 9 | 0.596 |
| 440 | JOSHA RES CTR | 9 | 0.596 |
| 441 | KERNODLE CLIN | 9 | 0.596 |
| 442 | KHARKIV CITY HOSP 3 | 9 | 0.596 |
| 443 | KING CHULALONGKORN MEM HOSP | 9 | 0.596 |
| 444 | KISHIWADA TOKUSHUKAI HOSP | 9 | 0.596 |
| 445 | KOBARI GEN HOSP | 9 | 0.596 |
| 446 | KOREA UNIV ANAM HOSP | 9 | 0.596 |
| 447 | KOREA UNIV GURO HOSP | 9 | 0.596 |
| 448 | KORGIALENIO BENAKIO HELLEN RED CROSS HOSP | 9 | 0.596 |
| 449 | KRANKENHAUS HIETZING NZR | 9 | 0.596 |
| 450 | KRISHNA INST MED SCI | 9 | 0.596 |
| 451 | KYUNGPOOK NATL UNIV HOSP | 9 | 0.596 |
| 452 | L J CLIN | 9 | 0.596 |
| 453 | LAKSHMI HOSP | 9 | 0.596 |
| 454 | LANDESKRANKENHAUS FELDKIRCH | 9 | 0.596 |
| 455 | LANGEBERG CLIN TRIALS | 9 | 0.596 |
| 456 | LEADING EDGE RES PA | 9 | 0.596 |
| 457 | LEADING EDGE RES PA INOVA | 9 | 0.596 |
| 458 | LEXINGTON LLC | 9 | 0.596 |
| 459 | LION RES | 9 | 0.596 |
| 460 | LKH SALZBURG | 9 | 0.596 |
| 461 | LLC INT MED CTR SOGAZ | 9 | 0.596 |
| 462 | LLC MEDINET | 9 | 0.596 |
| 463 | LUBBOCK DIAGNOST CLIN | 9 | 0.596 |
| 464 | LYNN INST | 9 | 0.596 |
| 465 | MAASTRICHT UMC | 9 | 0.596 |
| 466 | MACKAY MEM HOSP | 9 | 0.596 |
| 467 | MAHARAJ NAKORN CHIANGMAI HOSP | 9 | 0.596 |
| 468 | MAHARAT NAKHON RATCHASIMA HOSP | 9 | 0.596 |
| 469 | MAIN DEPT INTERNAL AFFAIRS | 9 | 0.596 |
| 470 | MASARYK HOSP | 9 | 0.596 |
| 471 | MEANDER MED CTR | 9 | 0.596 |
| 472 | MED AFFILIATED RES CTR INC | 9 | 0.596 |
| 473 | MED CTR GORECHT | 9 | 0.596 |
| 474 | MED CTR HOSP SWIETEJ RODZINY | 9 | 0.596 |
| 475 | MED CTR MEDELITE LTD | 9 | 0.596 |
| 476 | MED GRP ENCINO | 9 | 0.596 |
| 477 | MED RES HLTH EDUC FDN INC | 9 | 0.596 |
| 478 | MED RES SOUTH LLC | 9 | 0.596 |
| 479 | MED SENTER FORNEBU | 9 | 0.596 |
| 480 | MEDIAB SRL | 9 | 0.596 |
| 481 | MEDISPHERE MED RES CTR LLC | 9 | 0.596 |
| 482 | MEDSTAR RES INST | 9 | 0.596 |
| 483 | MERCK CO INC | 9 | 0.596 |
| 484 | MERELAHE FAMILY DOCTORS CTR | 9 | 0.596 |
| 485 | MERIDEN RES | 9 | 0.596 |
| 486 | MERIDIEN RES | 9 | 0.596 |
| 487 | METROPOLITAN MED CTR | 9 | 0.596 |
| 488 | MICHAEL L REEVES MD | 9 | 0.596 |
| 489 | MICHELLE ZANIEWSKI MD PA | 9 | 0.596 |
| 490 | MILEGROUND FAMILY PRACTICE | 9 | 0.596 |
| 491 | MINNESOTA CTR OBES METAB EDOCRINOL PA | 9 | 0.596 |
| 492 | MONASH UNIV SUNWAY CAMPUS | 9 | 0.596 |
| 493 | MOSCOW GUZ CITY CLIN HOSP | 9 | 0.596 |
| 494 | MOSCOW GUZ CITY CLIN HOSP 68 | 9 | 0.596 |
| 495 | MT ROYAL FAMILY PHYSICIANS | 9 | 0.596 |
| 496 | MT VIEW CLIN RES | 9 | 0.596 |
| 497 | MULTICARE SPECIALTIES RES | 9 | 0.596 |
| 498 | MUZ CLIN HOSP 2 | 9 | 0.596 |
| 499 | MUZ NOVOSIBIRSK MUNICIPAL CLIN HOSP EMERGENCY 2 | 9 | 0.596 |
| 500 | NAIDU CLIN | 9 | 0.596 |
| 501 | NATL CTR DIABET RES LTD | 9 | 0.596 |
| 502 | NATL HOSP | 9 | 0.596 |
| 503 | NATL HOSP ORG HOKKAIDO MED CTR | 9 | 0.596 |
| 504 | NATL INST ENDOCRINOL LTD | 9 | 0.596 |
| 505 | NATL KAPODISTRIAN UNIV ATHENS | 9 | 0.596 |
| 506 | NEW HORIZONS CLIN RES | 9 | 0.596 |
| 507 | NEWTOWN CLIN RES CTR | 9 | 0.596 |
| 508 | NHS DUMFRIES GALLOWAY | 9 | 0.596 |
| 509 | NHS LANARKSHIRE | 9 | 0.596 |
| 510 | NISUS RES | 9 | 0.596 |
| 511 | NONPUBL HEALTHCARE CTR NASZ LEKARZ | 9 | 0.596 |
| 512 | NORTH ESTONIA MED CTR FDN | 9 | 0.596 |
| 513 | NORTH MYRTLE BEACH FAMILY PRACTICE | 9 | 0.596 |
| 514 | NORTH TEXAS ENDOCRINE CTR | 9 | 0.596 |
| 515 | NORTHSIDE INTERNAL MED | 9 | 0.596 |
| 516 | NORWALK MED | 9 | 0.596 |
| 517 | NOVOSIBIRSK REG CLIN | 9 | 0.596 |
| 518 | NZOZ ALL MED MED CTR | 9 | 0.596 |
| 519 | NZOZ PRZYCHODNIA SPECJALISTYCZNA MED | 9 | 0.596 |
| 520 | ODESSA STATE MED UNIV | 9 | 0.596 |
| 521 | OKINAWA TOKUSHUKAI MED CORP | 9 | 0.596 |
| 522 | ORANGE CTY RES INST | 9 | 0.596 |
| 523 | ORBIS MED CTR | 9 | 0.596 |
| 524 | OREGON MED GRP | 9 | 0.596 |
| 525 | OSLO UNIV SYKEHUS HF | 9 | 0.596 |
| 526 | OSVALDO BRUSCO MD | 9 | 0.596 |
| 527 | OSWIECIMSKIE CTR BADAN KLINICZNYCH MEDICOME SP ZO | 9 | 0.596 |
| 528 | PAARL RES CTR | 9 | 0.596 |
| 529 | PANACEA CLIN RES | 9 | 0.596 |
| 530 | PARQUE VELEZ SARFIELD | 9 | 0.596 |
| 531 | PARTNERS NEPHROL ENDOCRINOL | 9 | 0.596 |
| 532 | PELICAN IMPEX SRL | 9 | 0.596 |
| 533 | PHARMACOTHERAPY RES ASSOCIATES INC | 9 | 0.596 |
| 534 | PHILADELPHIA HLTH ASSOCIATES | 9 | 0.596 |
| 535 | PHILIPPINE NIKKEI JIN KAI POLYCLIN DIAGNOST CTR | 9 | 0.596 |
| 536 | PHOENIX MED GRP PC | 9 | 0.596 |
| 537 | PHYS EAST PA | 9 | 0.596 |
| 538 | PIONEER RES SOLUT INC | 9 | 0.596 |
| 539 | PISH MED ASSOCIATES | 9 | 0.596 |
| 540 | PREFERRED PRIMARY CARE PHYS INC | 9 | 0.596 |
| 541 | PRIME MED GRP | 9 | 0.596 |
| 542 | PRINCE WALES HOSP | 9 | 0.596 |
| 543 | PUCCAMP | 9 | 0.596 |
| 544 | PUSAN NATL UNIV HOSP | 9 | 0.596 |
| 545 | PUSAT PERUBATAN UNIV | 9 | 0.596 |
| 546 | Q T RES OUTAOUAIS | 9 | 0.596 |
| 547 | RADIANT RES INC | 9 | 0.596 |
| 548 | REG HOSP RANDERS | 9 | 0.596 |
| 549 | REINIER DE GRAAF GASTHUIS | 9 | 0.596 |
| 550 | RES INST DALLAS | 9 | 0.596 |
| 551 | RES UNIT | 9 | 0.596 |
| 552 | RICHARD CHERLIN MD | 9 | 0.596 |
| 553 | RISER MED RES | 9 | 0.596 |
| 554 | RITCHKEN FIRST MDS | 9 | 0.596 |
| 555 | ROBLEY REX VA MED CTR | 9 | 0.596 |
| 556 | ROCKWOOD DIABET METAB HLTH CTR | 9 | 0.596 |
| 557 | ROPCKE ZWEERS ZIEKENHUIS | 9 | 0.596 |
| 558 | ROPHEKA MED CTR | 9 | 0.596 |
| 559 | ROTTERDAM RES INST | 9 | 0.596 |
| 560 | RUMAH SAKIT FK UKI | 9 | 0.596 |
| 561 | S R KALLA GASTROENTOROL GEN HOSP | 9 | 0.596 |
| 562 | SAIFUL ANWAR HOSP | 9 | 0.596 |
| 563 | SAMSUNG MED CTR | 9 | 0.596 |
| 564 | SANATORIO GUEMES HOSP PRIVADO | 9 | 0.596 |
| 565 | SANGLAH HOSP | 9 | 0.596 |
| 566 | SANTA CASA MISERICORDIA BELO HORIZONTE | 9 | 0.596 |
| 567 | SARDJITO HOSP | 9 | 0.596 |
| 568 | SC CARDIOCTR DR ISTRATOAIE SRL | 9 | 0.596 |
| 569 | SC CARDIOMED SRL | 9 | 0.596 |
| 570 | SC DIANA BARBONTA SRL | 9 | 0.596 |
| 571 | SCI CTR RADIAT | 9 | 0.596 |
| 572 | SEIDMAN CLIN TRIALS | 9 | 0.596 |
| 573 | SENOR SANTO NINO HOSP | 9 | 0.596 |
| 574 | SEVERANCE HOSP | 9 | 0.596 |
| 575 | SHANGHAI JIAO TONG UNIV | 9 | 0.596 |
| 576 | SHATABDI SUPER SPECIAL HOSP | 9 | 0.596 |
| 577 | SHREE KRISHNA HOSP HEART CARE CTR | 9 | 0.596 |
| 578 | SKDS RES INC | 9 | 0.596 |
| 579 | SLAGELSE SYGEHUS | 9 | 0.596 |
| 580 | SOETOMO HOSP | 9 | 0.596 |
| 581 | SOUTH BROWARD RES | 9 | 0.596 |
| 582 | SOUTH CAROLINA PHARMACEUT RES | 9 | 0.596 |
| 583 | SOUTHWEST CLIN RES CTR LLC | 9 | 0.596 |
| 584 | SOUTHWEST CLIN TRIAL | 9 | 0.596 |
| 585 | SPANDAN HEART INST RES CTR | 9 | 0.596 |
| 586 | SPECIALIZED HOSP MED REHABIL | 9 | 0.596 |
| 587 | SPECIALIZED PHYS OFF KO MED | 9 | 0.596 |
| 588 | SRI JAYEWARDENEPURA GEN HOSP PGMI | 9 | 0.596 |
| 589 | ST ANNA HOSP | 9 | 0.596 |
| 590 | ST ANTONIUS HOSP | 9 | 0.596 |
| 591 | ST FRANCISCUS GASTHUIS | 9 | 0.596 |
| 592 | ST JOHNS MED COLL HOSP | 9 | 0.596 |
| 593 | ST JOSEPHS MED ASSOCIATES | 9 | 0.596 |
| 594 | ST ORSOLA MARCELLO MALPIGHI HOSP | 9 | 0.596 |
| 595 | ST PETERSBURG GUZ CITY CLIN HOSP 40 | 9 | 0.596 |
| 596 | ST PETERSBURG GUZ CITY CLIN HOSP ST PETERSBURG | 9 | 0.596 |
| 597 | ST PETERSBURG STATE HEALTHCARE INST | 9 | 0.596 |
| 598 | ST PETERSBURG STATE MED UNIV | 9 | 0.596 |
| 599 | STANWELL RD SURG | 9 | 0.596 |
| 600 | STEDMAN CLIN TRIALS | 9 | 0.596 |
| 601 | STRELITZ DIABET CTR | 9 | 0.596 |
| 602 | SUMANA HOSP | 9 | 0.596 |
| 603 | SWAN LANE MED CTR | 9 | 0.596 |
| 604 | SYKEHUSET INNLANDET HF | 9 | 0.596 |
| 605 | SYNEXUS HUNGARY LTD | 9 | 0.596 |
| 606 | SYNOPSIS RES | 9 | 0.596 |
| 607 | SZENT PANTALEON HOSP | 9 | 0.596 |
| 608 | TAGORE HOSP HEART CARE CTR | 9 | 0.596 |
| 609 | TARTU ENDOCRINOL CTR | 9 | 0.596 |
| 610 | TARTU UNIV HOSP | 9 | 0.596 |
| 611 | TEACHING HOSP PERADENIYA | 9 | 0.596 |
| 612 | TEL AVIV UNIV | 9 | 0.596 |
| 613 | TEXAS HLTH PHYSICIANS GRP | 9 | 0.596 |
| 614 | THAMMASAT UNIV HOSP | 9 | 0.596 |
| 615 | THOMAS JEFFERSON UNIV | 9 | 0.596 |
| 616 | TIERVLEI TRIAL CTR | 9 | 0.596 |
| 617 | TIPTON MED DIAGNOST CTR | 9 | 0.596 |
| 618 | TOTAL DIABET HORMONE INST | 9 | 0.596 |
| 619 | TOWER POINTE RES CTR | 9 | 0.596 |
| 620 | TREAD RES | 9 | 0.596 |
| 621 | TRY RES | 9 | 0.596 |
| 622 | TUNG WAH EASTERN HOSP | 9 | 0.596 |
| 623 | UDONTHANI HOSP | 9 | 0.596 |
| 624 | UERM INST STUDIES DIABET FDN INC | 9 | 0.596 |
| 625 | UF ENDOCRINOL DIABET | 9 | 0.596 |
| 626 | UNIDAD INVEST CLIN CARDIOMETAB | 9 | 0.596 |
| 627 | UNIDADE LOCAL SAUDE ALTO MINHO | 9 | 0.596 |
| 628 | UNIENDO UNIDAD INTEGRAL ENDOCRINOL | 9 | 0.596 |
| 629 | UNIMED AJARA LLC | 9 | 0.596 |
| 630 | UNITED MED ASSOCIATES | 9 | 0.596 |
| 631 | UNIV BIRMINGHAM | 9 | 0.596 |
| 632 | UNIV CALIF LOS ANGELES | 9 | 0.596 |
| 633 | UNIV CLIN CTR | 9 | 0.596 |
| 634 | UNIV DIABET ENDOCRINE CONSULTANTS INC | 9 | 0.596 |
| 635 | UNIV ESTADUAL MARINGA | 9 | 0.596 |
| 636 | UNIV FED PARANA | 9 | 0.596 |
| 637 | UNIV HLTH NETWORK | 9 | 0.596 |
| 638 | UNIV HOSP | 9 | 0.596 |
| 639 | UNIV HOSP CTR RIJEKA | 9 | 0.596 |
| 640 | UNIV HOSP GASTHUISBERG | 9 | 0.596 |
| 641 | UNIV KLIN INNERE MED II | 9 | 0.596 |
| 642 | UNIV KLIN INNERE MED III | 9 | 0.596 |
| 643 | UNIV KLIN INNSBRUCK | 9 | 0.596 |
| 644 | UNIV MALAYA MED CTR | 9 | 0.596 |
| 645 | UNIV MANITOBA | 9 | 0.596 |
| 646 | UNIV MELBOURNE | 9 | 0.596 |
| 647 | UNIV PHYS GRP | 9 | 0.596 |
| 648 | UNIV QUEENSLAND | 9 | 0.596 |
| 649 | UNIV RIBEIRAO PRETO | 9 | 0.596 |
| 650 | UNIV TEKNOL MARA | 9 | 0.596 |
| 651 | UNIV WESTERN AUSTRALIA | 9 | 0.596 |
| 652 | UNIV WISCONSIN | 9 | 0.596 |
| 653 | UNIV WURZBURG | 9 | 0.596 |
| 654 | UO MED INTERNA | 9 | 0.596 |
| 655 | UOC CARDIOL | 9 | 0.596 |
| 656 | UOC ENDOCRINOL | 9 | 0.596 |
| 657 | UOC ENDOCRINOL DIABETOL MALATTIE METABOL | 9 | 0.596 |
| 658 | UTAH CLIN TRIALS LLC | 9 | 0.596 |
| 659 | UZ BRUSSELS | 9 | 0.596 |
| 660 | UZA | 9 | 0.596 |
| 661 | VALLEY ENDOCRINE DIABET CONSULTANTS INC | 9 | 0.596 |
| 662 | VERGELEGEN MEDICLIN | 9 | 0.596 |
| 663 | VIA CHRISTI CLIN PA | 9 | 0.596 |
| 664 | VIACAR RECH CLINIQUE INC | 9 | 0.596 |
| 665 | VLIETLAND ZIEKENHUIS | 9 | 0.596 |
| 666 | VSEVOLOZHSK CENT REG HOSP | 9 | 0.596 |
| 667 | WALLA WALLA CLIN | 9 | 0.596 |
| 668 | WATERLOO MED CTR | 9 | 0.596 |
| 669 | WELLINGTON HOSP | 9 | 0.596 |
| 670 | WELLS INST HLTH AWARENESS | 9 | 0.596 |
| 671 | WEST VISAYAS STATE UNIV MED CTR | 9 | 0.596 |
| 672 | WESTCOTT MED CTR | 9 | 0.596 |
| 673 | WESTERN GALILEE HOSP | 9 | 0.596 |
| 674 | WESTERN GEN HOSP | 9 | 0.596 |
| 675 | WILHELMINENSPITAL WIEN | 9 | 0.596 |
| 676 | WINDSOR MED CLIN | 9 | 0.596 |
| 677 | WONJU SEVERANCE CHRISTIAN HOSP | 9 | 0.596 |
| 678 | ZHORDANIA INST HUMAN REPROD | 9 | 0.596 |
| 679 | ZIV MED CTR | 9 | 0.596 |
| 680 | BRISTOL MYERS SQUIBB CO | 8 | 0.53 |
| 681 | CHINESE UNIV HONG KONG | 8 | 0.53 |
| 682 | FUDAN UNIV | 8 | 0.53 |
| 683 | HEIDELBERG UNIV | 8 | 0.53 |
| 684 | JANSSEN RES DEV | 8 | 0.53 |
| 685 | JICHI MED UNIV | 8 | 0.53 |
| 686 | JOHNS HOPKINS UNIV | 8 | 0.53 |
| 687 | MED UNIV VIENNA | 8 | 0.53 |
| 688 | NATL YANG MING UNIV | 8 | 0.53 |
| 689 | UNIV AMSTERDAM | 8 | 0.53 |
| 690 | UNIV CATTOLICA SACRO CUORE | 8 | 0.53 |
| 691 | UNIV FLORENCE | 8 | 0.53 |
| 692 | UNIV FLORIDA | 8 | 0.53 |
| 693 | UNIV PENN | 8 | 0.53 |
| 694 | ASTON UNIV | 7 | 0.464 |
| 695 | BAIM INST CLIN RES | 7 | 0.464 |
| 696 | CHIBA UNIV | 7 | 0.464 |
| 697 | COPENHAGEN UNIV HOSP | 7 | 0.464 |
| 698 | DUKE CLIN RES INST | 7 | 0.464 |
| 699 | HANNOVER MED SCH | 7 | 0.464 |
| 700 | IRCCS MULTIMED | 7 | 0.464 |
| 701 | JUNTENDO UNIV | 7 | 0.464 |
| 702 | KEIO UNIV | 7 | 0.464 |
| 703 | LEICESTER GEN HOSP | 7 | 0.464 |
| 704 | LONDON SCH HYG TROP MED | 7 | 0.464 |
| 705 | MASHHAD UNIV MED SCI | 7 | 0.464 |
| 706 | MED UNIV LODZ | 7 | 0.464 |
| 707 | OSAKA UNIV | 7 | 0.464 |
| 708 | RHEIN WESTFAL TH AACHEN | 7 | 0.464 |
| 709 | UNIV CAMPANIA LUIGI VANVITELLI | 7 | 0.464 |
| 710 | UNIV MINNESOTA | 7 | 0.464 |
| 711 | UNIV WARWICK | 7 | 0.464 |
| 712 | VAMC | 7 | 0.464 |
| 713 | VANDERBILT UNIV | 7 | 0.464 |
| 714 | YOKOHAMA CITY UNIV | 7 | 0.464 |
| 715 | CAPIO ST GORANS HOSP | 6 | 0.398 |
| 716 | CEDARS SINAI MED CTR | 6 | 0.398 |
| 717 | CHARLES UNIV PRAGUE | 6 | 0.398 |
| 718 | CHINA MED UNIV | 6 | 0.398 |
| 719 | DALLAS DIABET RES CTR MED CITY | 6 | 0.398 |
| 720 | DREXEL UNIV | 6 | 0.398 |
| 721 | DUKE NATL UNIV SINGAPORE | 6 | 0.398 |
| 722 | INDIANA UNIV SCH MED | 6 | 0.398 |
| 723 | KAROLINSKA UNIV HOSP | 6 | 0.398 |
| 724 | KITASATO UNIV | 6 | 0.398 |
| 725 | KYUSHU UNIV | 6 | 0.398 |
| 726 | MAGNA GRAECIA UNIV CATANZARO | 6 | 0.398 |
| 727 | NANJING MED UNIV | 6 | 0.398 |
| 728 | NEW YORK MED COLL | 6 | 0.398 |
| 729 | NORTHWESTERN UNIV | 6 | 0.398 |
| 730 | OHIO STATE UNIV | 6 | 0.398 |
| 731 | RUHR UNIV BOCHUM | 6 | 0.398 |
| 732 | SAGA UNIV | 6 | 0.398 |
| 733 | ST VINCENTS HOSP | 6 | 0.398 |
| 734 | UNIV BOLOGNA | 6 | 0.398 |
| 735 | UNIV CALIF SAN FRANCISCO | 6 | 0.398 |
| 736 | UNIV HOSP LLANDOUGH | 6 | 0.398 |
| 737 | UNIV LORRAINE | 6 | 0.398 |
| 738 | UNIV NANTES | 6 | 0.398 |
| 739 | UNIV TEXAS SW MED CTR DALLAS | 6 | 0.398 |
| 740 | UNIV VALENCIA | 6 | 0.398 |
| 741 | AALBORG UNIV | 5 | 0.331 |
| 742 | AARHUS UNIV HOSP | 5 | 0.331 |
| 743 | ASTRAZENECA NORD BALT | 5 | 0.331 |
| 744 | AUSTIN HLTH | 5 | 0.331 |
| 745 | BRISTOL MYERS SQUIBB | 5 | 0.331 |
| 746 | CAPITAL MED UNIV | 5 | 0.331 |
| 747 | CHARITE | 5 | 0.331 |
| 748 | CNR | 5 | 0.331 |
| 749 | HARRY S TRUMAN MEM VET HOSP | 5 | 0.331 |
| 750 | HLTH ECON OUTCOMES RES LTD | 5 | 0.331 |
| 751 | IRCCS SAN RAFFAELE PISANA | 5 | 0.331 |
| 752 | KAGAWA UNIV | 5 | 0.331 |
| 753 | KATHOLIEKE UNIV LEUVEN | 5 | 0.331 |
| 754 | KYUNG HEE UNIV | 5 | 0.331 |
| 755 | MAYO CLIN | 5 | 0.331 |
| 756 | MITSUBISHI TANABE PHARMA CORP | 5 | 0.331 |
| 757 | MONTREAL HEART INST | 5 | 0.331 |
| 758 | NIPPON BOEHRINGER INGELHEIM CO LTD | 5 | 0.331 |
| 759 | NOVO NORDISK AS | 5 | 0.331 |
| 760 | PFIZER INC | 5 | 0.331 |
| 761 | RIGSHOSP | 5 | 0.331 |
| 762 | SICHUAN UNIV | 5 | 0.331 |
| 763 | SUNY STONY BROOK | 5 | 0.331 |
| 764 | TAICHUNG VET GEN HOSP | 5 | 0.331 |
| 765 | TOHO UNIV | 5 | 0.331 |
| 766 | TOKYO MED DENT UNIV | 5 | 0.331 |
| 767 | TOKYO MED UNIV | 5 | 0.331 |
| 768 | UNIV ALABAMA BIRMINGHAM | 5 | 0.331 |
| 769 | UNIV AUTONOMA BARCELONA | 5 | 0.331 |
| 770 | UNIV BELGRADE | 5 | 0.331 |
| 771 | UNIV CALGARY | 5 | 0.331 |
| 772 | UNIV CAMPANIA L VANVITELLI | 5 | 0.331 |
| 773 | UNIV GOTHENBURG | 5 | 0.331 |
| 774 | UNIV HONG KONG | 5 | 0.331 |
| 775 | UNIV ILLINOIS | 5 | 0.331 |
| 776 | UNIV MALAYA | 5 | 0.331 |
| 777 | UNIV MICHIGAN | 5 | 0.331 |
| 778 | UNIV PORTO | 5 | 0.331 |
| 779 | UNIV SAARLAND | 5 | 0.331 |
| 780 | UNIV SURREY | 5 | 0.331 |
| 781 | UNIV ZAGREB | 5 | 0.331 |
| 782 | VA MED CTR | 5 | 0.331 |
| 783 | VET AFFAIRS MED CTR | 5 | 0.331 |
| 784 | VIRGINIA COMMONWEALTH UNIV | 5 | 0.331 |
| 785 | VRIJE UNIV AMSTERDAM MED CTR | 5 | 0.331 |
| 786 | WASHINGTON UNIV | 5 | 0.331 |
| 787 | WEILL CORNELL MED QATAR | 5 | 0.331 |
| 788 | YALE NEW HAVEN MED CTR | 5 | 0.331 |
| 789 | YONSEI UNIV | 5 | 0.331 |
| 790 | AJOU UNIV | 4 | 0.265 |
| 791 | AMER UNIV BEIRUT | 4 | 0.265 |
| 792 | AMRI HOSP | 4 | 0.265 |
| 793 | ASAHIKAWA MED UNIV | 4 | 0.265 |
| 794 | BAYLOR HEART VASC HOSP | 4 | 0.265 |
| 795 | BAYLOR SCOTT WHITE RES INST | 4 | 0.265 |
| 796 | BETH ISRAEL DEACONESS MED CTR | 4 | 0.265 |
| 797 | BHARTI HOSP | 4 | 0.265 |
| 798 | BOEHRINGER INGELHEIM INT | 4 | 0.265 |
| 799 | CAREGGI HOSP | 4 | 0.265 |
| 800 | CHANG GUNG UNIV | 4 | 0.265 |
| 801 | CHIANG MAI UNIV | 4 | 0.265 |
| 802 | CHINESE ACAD MED SCI | 4 | 0.265 |
| 803 | CHU | 4 | 0.265 |
| 804 | COLUMBIA UNIV | 4 | 0.265 |
| 805 | DEMOCRITUS UNIV THRACE | 4 | 0.265 |
| 806 | DOKKYO MED UNIV | 4 | 0.265 |
| 807 | EINSTEIN MED CTR | 4 | 0.265 |
| 808 | GARVAN INST MED RES | 4 | 0.265 |
| 809 | HELSINKI UNIV HOSP | 4 | 0.265 |
| 810 | HERLEV GENTOFTE HOSP | 4 | 0.265 |
| 811 | HOKKAIDO UNIV | 4 | 0.265 |
| 812 | INSERM | 4 | 0.265 |
| 813 | KANAZAWA UNIV | 4 | 0.265 |
| 814 | KINGS COLL HOSP LONDON | 4 | 0.265 |
| 815 | KOBE UNIV | 4 | 0.265 |
| 816 | KOREA UNIV | 4 | 0.265 |
| 817 | KURUME UNIV | 4 | 0.265 |
| 818 | LINKOPING UNIV | 4 | 0.265 |
| 819 | MCMASTER UNIV | 4 | 0.265 |
| 820 | MCPHS UNIV | 4 | 0.265 |
| 821 | MED UNIV | 4 | 0.265 |
| 822 | MED UNIV SOUTH CAROLINA | 4 | 0.265 |
| 823 | MINERVA FDN | 4 | 0.265 |
| 824 | NATL CHENG KUNG UNIV | 4 | 0.265 |
| 825 | NATL HEART CTR SINGAPORE | 4 | 0.265 |
| 826 | NATL UNIV CORDOBA | 4 | 0.265 |
| 827 | NIDDK | 4 | 0.265 |
| 828 | NIGHTINGALE HOSP | 4 | 0.265 |
| 829 | NIHON UNIV | 4 | 0.265 |
| 830 | OREGON HLTH SCI UNIV | 4 | 0.265 |
| 831 | ROYAL N SHORE HOSP | 4 | 0.265 |
| 832 | ROYAL PERTH HOSP | 4 | 0.265 |
| 833 | RUTGERS STATE UNIV | 4 | 0.265 |
| 834 | SAPPORO MED UNIV | 4 | 0.265 |
| 835 | SPRINGER | 4 | 0.265 |
| 836 | SUNGKYUNKWAN UNIV | 4 | 0.265 |
| 837 | TAIPEI MED UNIV | 4 | 0.265 |
| 838 | TEMPLE UNIV | 4 | 0.265 |
| 839 | TEXAS A M COLL MED | 4 | 0.265 |
| 840 | TIANJIN MED UNIV | 4 | 0.265 |
| 841 | TOKAI UNIV | 4 | 0.265 |
| 842 | UNIV ARIZONA | 4 | 0.265 |
| 843 | UNIV AUTONOMA MADRID | 4 | 0.265 |
| 844 | UNIV BRESCIA | 4 | 0.265 |
| 845 | UNIV CYPRUS | 4 | 0.265 |
| 846 | UNIV DUNDEE | 4 | 0.265 |
| 847 | UNIV EDINBURGH | 4 | 0.265 |
| 848 | UNIV ESTADUAL CAMPINAS | 4 | 0.265 |
| 849 | UNIV FREIBURG | 4 | 0.265 |
| 850 | UNIV GENOA | 4 | 0.265 |
| 851 | UNIV GEORGIA | 4 | 0.265 |
| 852 | UNIV HOSP AACHEN | 4 | 0.265 |
| 853 | UNIV HOSP LEICESTER NHS TRUST | 4 | 0.265 |
| 854 | UNIV LILLE | 4 | 0.265 |
| 855 | UNIV NACL CORDOBA | 4 | 0.265 |
| 856 | UNIV NEW SOUTH WALES SYDNEY | 4 | 0.265 |
| 857 | UNIV RYUKYUS | 4 | 0.265 |
| 858 | UNIV S FLORIDA | 4 | 0.265 |
| 859 | UNIV SKOVDE | 4 | 0.265 |
| 860 | UNIV TENNESSEE | 4 | 0.265 |
| 861 | UNIV TEXAS MED BRANCH | 4 | 0.265 |
| 862 | UNIV THESSALY | 4 | 0.265 |
| 863 | UNIV ULM | 4 | 0.265 |
| 864 | UT SOUTHWESTERN MED CTR | 4 | 0.265 |
| 865 | WESTERN UNIV | 4 | 0.265 |
| 866 | ZHEJIANG UNIV | 4 | 0.265 |
| 867 | AARHUS UNIV | 3 | 0.199 |
| 868 | AICHI MED UNIV | 3 | 0.199 |
| 869 | ALFRED HOSP | 3 | 0.199 |
| 870 | AZIENDA OSPED UNIV PARMA | 3 | 0.199 |
| 871 | BANTING BEST DIABET CTR | 3 | 0.199 |
| 872 | BOEHRINGER INGELHEIM AB | 3 | 0.199 |
| 873 | BOEHRINGER INGELHEIM CANADA LTD | 3 | 0.199 |
| 874 | BOEHRINGER INGELHEIM LTD | 3 | 0.199 |
| 875 | BOGIER CLIN IT SOLUT | 3 | 0.199 |
| 876 | CAROL DAVILA UNIV MED PHARM | 3 | 0.199 |
| 877 | CHANG GUNG MED FDN | 3 | 0.199 |
| 878 | CHOSUN UNIV | 3 | 0.199 |
| 879 | CLEVELAND CLIN FDN | 3 | 0.199 |
| 880 | CLIN HOSP DUBRAVA | 3 | 0.199 |
| 881 | COLORADO STATE UNIV | 3 | 0.199 |
| 882 | COMENIUS UNIV | 3 | 0.199 |
| 883 | CTR INVEST BIOMED RED DIABET ENFERMEDADES METAB | 3 | 0.199 |
| 884 | EAST CAROLINA UNIV | 3 | 0.199 |
| 885 | ELI LILLY CO | 3 | 0.199 |
| 886 | EVIDERA | 3 | 0.199 |
| 887 | FORSCHERGRP DIABET EV | 3 | 0.199 |
| 888 | FUKUOKA UNIV | 3 | 0.199 |
| 889 | GD HOSP DIABET INST | 3 | 0.199 |
| 890 | GENTOFTE UNIV HOSP | 3 | 0.199 |
| 891 | GERMAN CTR CARDIOVASC RES DZHK | 3 | 0.199 |
| 892 | GLAXOSMITHKLINE | 3 | 0.199 |
| 893 | GWANGMYEONG SUNGAE HOSP | 3 | 0.199 |
| 894 | HARVARD TH CHAN SCH PUBL HLTH | 3 | 0.199 |
| 895 | HEART ENGLAND NHS FDN TRUST | 3 | 0.199 |
| 896 | HELMHOLTZ CTR | 3 | 0.199 |
| 897 | HIROSHIMA UNIV | 3 | 0.199 |
| 898 | HOKKAIDO UNIV HOSP | 3 | 0.199 |
| 899 | INDIANA UNIV | 3 | 0.199 |
| 900 | INJE UNIV | 3 | 0.199 |
| 901 | INST CARDIOVASC BUENOS AIRES | 3 | 0.199 |
| 902 | INST INVEST BIOMED AUGUST PI I SUNYER IDIBAPS | 3 | 0.199 |
| 903 | INST SALUD CARLOS III | 3 | 0.199 |
| 904 | INT UNIV HLTH WELF | 3 | 0.199 |
| 905 | JANSSEN GLOBAL SERV LLC | 3 | 0.199 |
| 906 | JEWISH GEN HOSP | 3 | 0.199 |
| 907 | JOHANNES GUTENBERG UNIV MAINZ | 3 | 0.199 |
| 908 | JOHNS HOPKINS BLOOMBERG SCH PUBL HLTH | 3 | 0.199 |
| 909 | JOSLIN DIABET CTR | 3 | 0.199 |
| 910 | KANSAI ELECT POWER HOSP | 3 | 0.199 |
| 911 | KEELUNG CHANG GUNG MEM HOSP | 3 | 0.199 |
| 912 | KING ABDULAZIZ UNIV | 3 | 0.199 |
| 913 | KOWA CO LTD | 3 | 0.199 |
| 914 | KUMAMOTO UNIV | 3 | 0.199 |
| 915 | KUSATSU GEN HOSP | 3 | 0.199 |
| 916 | LINKOU CHANG GUNG MEM HOSP | 3 | 0.199 |
| 917 | MACCABI HEALTHCARE SERV | 3 | 0.199 |
| 918 | MED UNIV SILESIA | 3 | 0.199 |
| 919 | MEDANTA | 3 | 0.199 |
| 920 | METAB INST AMER | 3 | 0.199 |
| 921 | MICHAEL E DEBAKEY VA MED CTR | 3 | 0.199 |
| 922 | NAGOYA UNIV | 3 | 0.199 |
| 923 | NATL MED RES CTR CARDIOL | 3 | 0.199 |
| 924 | NATL RES INST | 3 | 0.199 |
| 925 | NATL TAIWAN UNIV | 3 | 0.199 |
| 926 | OKAYAMA UNIV | 3 | 0.199 |
| 927 | PAPAGEORGIOU HOSP | 3 | 0.199 |
| 928 | QUEENS UNIV | 3 | 0.199 |
| 929 | REDINREN | 3 | 0.199 |
| 930 | ROYAL BROMPTON HOSP | 3 | 0.199 |
| 931 | SAITAMA MED UNIV | 3 | 0.199 |
| 932 | SERBIAN ACAD ARTS SCI | 3 | 0.199 |
| 933 | SHAHID BEHESHTI UNIV MED SCI | 3 | 0.199 |
| 934 | SLAGELSE HOSP | 3 | 0.199 |
| 935 | SOUTHERN DENMARK UNIV | 3 | 0.199 |
| 936 | SOUTHSIDE ENDOCRINOL | 3 | 0.199 |
| 937 | ST GEORGES UNIV LONDON | 3 | 0.199 |
| 938 | STATE UNIV CAMPINAS UNICAMP | 3 | 0.199 |
| 939 | SUN YAT SEN UNIV | 3 | 0.199 |
| 940 | SUNY BUFFALO | 3 | 0.199 |
| 941 | TAISHO PHARMACEUT CO LTD | 3 | 0.199 |
| 942 | TAN TAO UNIV | 3 | 0.199 |
| 943 | TECHNION ISRAEL INST TECHNOL | 3 | 0.199 |
| 944 | TOKUSHIMA UNIV | 3 | 0.199 |
| 945 | TONGJI UNIV | 3 | 0.199 |
| 946 | TUFTS UNIV | 3 | 0.199 |
| 947 | UHN | 3 | 0.199 |
| 948 | UIC ADVOCATE CHRIST MED CTR | 3 | 0.199 |
| 949 | UNITED ARAB EMIRATES UNIV | 3 | 0.199 |
| 950 | UNIV ADELAIDE | 3 | 0.199 |
| 951 | UNIV BARI ALDO MORO | 3 | 0.199 |
| 952 | UNIV COLL DUBLIN | 3 | 0.199 |
| 953 | UNIV COLOGNE | 3 | 0.199 |
| 954 | UNIV COLORADO HLTH | 3 | 0.199 |
| 955 | UNIV HOSP REGENSBURG | 3 | 0.199 |
| 956 | UNIV HOSP WURZBURG | 3 | 0.199 |
| 957 | UNIV KLINIKUM SAARLANDES | 3 | 0.199 |
| 958 | UNIV LA LAGUNA | 3 | 0.199 |
| 959 | UNIV MED CTR | 3 | 0.199 |
| 960 | UNIV MED CTR GOTTINGEN | 3 | 0.199 |
| 961 | UNIV MILAN | 3 | 0.199 |
| 962 | UNIV MISSOURI KANSAS CITY | 3 | 0.199 |
| 963 | UNIV MONTREAL | 3 | 0.199 |
| 964 | UNIV NAPLES FEDERICO II | 3 | 0.199 |
| 965 | UNIV NEBRASKA MED CTR | 3 | 0.199 |
| 966 | UNIV OCCUPAT ENVIRONM HLTH | 3 | 0.199 |
| 967 | UNIV PALERMO | 3 | 0.199 |
| 968 | UNIV SOUTHERN CALIF | 3 | 0.199 |
| 969 | UNIV SOUTHERN DENMARK | 3 | 0.199 |
| 970 | UNIV TURKU | 3 | 0.199 |
| 971 | UNIV WATERLOO | 3 | 0.199 |
| 972 | UNIV WUERZBURG HOSP | 3 | 0.199 |
| 973 | UNIV WYOMING | 3 | 0.199 |
| 974 | VA BOSTON HEALTHCARE SYST | 3 | 0.199 |
| 975 | VRIJE UNIV AMSTERDAM | 3 | 0.199 |
| 976 | WASHINGTON STATE UNIV | 3 | 0.199 |
| 977 | WESTCHESTER MED CTR | 3 | 0.199 |
| 978 | ZIEKENHUISGRP TWENTE | 3 | 0.199 |
| 979 | AIJINKAI TAKATSUKI HOSP | 2 | 0.133 |
| 980 | AINTREE UNIV HOSP NHS FDN TRUST | 2 | 0.133 |
| 981 | ALBANY MED COLL | 2 | 0.133 |
| 982 | ALMA MATER STUDIORUM UNIV BOLOGNA | 2 | 0.133 |
| 983 | ANAL GRP INC | 2 | 0.133 |
| 984 | ANKARA UNIV | 2 | 0.133 |
| 985 | ANTAROS MED AB | 2 | 0.133 |
| 986 | ANTWERP UNIV HOSP | 2 | 0.133 |
| 987 | AOKI CLIN | 2 | 0.133 |
| 988 | ASAHI GEN HOSP | 2 | 0.133 |
| 989 | ASAHI LIFE FDN | 2 | 0.133 |
| 990 | ASTELLAS US LLC | 2 | 0.133 |
| 991 | ASTRA ZENECA | 2 | 0.133 |
| 992 | ASTRAZENECA EUROPE CANADA | 2 | 0.133 |
| 993 | ASTRAZENECA R D | 2 | 0.133 |
| 994 | AUTONOMOUS UNIV BARCELONA | 2 | 0.133 |
| 995 | AZIENDA OSPED UNIV L VANVITELLI | 2 | 0.133 |
| 996 | AZIENDA OSPED UNIV PISANA | 2 | 0.133 |
| 997 | BAKER IDI HEART DIABET INST | 2 | 0.133 |
| 998 | BAYLOR JACK JANE HAMILTON HEART VASC HOSP | 2 | 0.133 |
| 999 | BEIJING AIRPORT HOSP | 2 | 0.133 |
| 1000 | BEIRUT ARAB UNIV | 2 | 0.133 |
| 1001 | BELFAST HLTH SOCIAL CARE TRUST | 2 | 0.133 |
| 1002 | BELLVITGE UNIV HOSP | 2 | 0.133 |
| 1003 | BERLIN INST HLTH CTR REGENERAT THERAPIES BCRT | 2 | 0.133 |
| 1004 | BHARTI HOSP BRIDE | 2 | 0.133 |
| 1005 | BISPEBJERG FREDERIKSBERG HOSP | 2 | 0.133 |
| 1006 | BOEHRINGER INGELHEIM CANADA LTD LTEE | 2 | 0.133 |
| 1007 | BOEHRINGER INGELHEIM FRANCE | 2 | 0.133 |
| 1008 | BOEHRINGER INGELHEIM NORWAY | 2 | 0.133 |
| 1009 | BOEHRINGER INGELHEIM PHARMACEUT | 2 | 0.133 |
| 1010 | BOGIER CLIN IT SOLUT INC | 2 | 0.133 |
| 1011 | BROWN UNIV | 2 | 0.133 |
| 1012 | CAIRO UNIV | 2 | 0.133 |
| 1013 | CANADIAN HEART RES CTR | 2 | 0.133 |
| 1014 | CARDIFF UNIV | 2 | 0.133 |
| 1015 | CARDIOVASC IMAGING CLIN | 2 | 0.133 |
| 1016 | CATHOLIC UNIV | 2 | 0.133 |
| 1017 | CHA UNIV | 2 | 0.133 |
| 1018 | CHAPMAN UNIV | 2 | 0.133 |
| 1019 | CHELSEA WESTMINSTER HOSP | 2 | 0.133 |
| 1020 | CHINA PHARMACEUT UNIV | 2 | 0.133 |
| 1021 | CHINESE ACAD SCI | 2 | 0.133 |
| 1022 | CHU LIEGE ULG CHU | 2 | 0.133 |
| 1023 | CHUNGBUK NATL UNIV | 2 | 0.133 |
| 1024 | CHUNGBUK NATL UNIV HOSP | 2 | 0.133 |
| 1025 | CHUNGNAM NATL UNIV | 2 | 0.133 |
| 1026 | CHURCHILL HOSP | 2 | 0.133 |
| 1027 | CIBER CARDIOVASC | 2 | 0.133 |
| 1028 | CIBERCV | 2 | 0.133 |
| 1029 | CINCINNATI CHILDRENS HOSP MED CTR | 2 | 0.133 |
| 1030 | CITY HOSP | 2 | 0.133 |
| 1031 | CLALIT HLTH SERV HEADQUARTERS | 2 | 0.133 |
| 1032 | CLIN UNIV HOSP VALENCIA | 2 | 0.133 |
| 1033 | CLIN UNIV NAVARRA | 2 | 0.133 |
| 1034 | COMPLEJO HOSP UNIV SANTIAGO DE COMPOSTELA | 2 | 0.133 |
| 1035 | COVENTRY UNIV | 2 | 0.133 |
| 1036 | CRAIGAVON AREA HOSP | 2 | 0.133 |
| 1037 | CTR OUTCOMES RES CLIN EPIDEMIOL | 2 | 0.133 |
| 1038 | DALLAS DIABET ENDOCRINE CTR MED CITY | 2 | 0.133 |
| 1039 | DEPT CARDIOL CVK | 2 | 0.133 |
| 1040 | DEPT MED | 2 | 0.133 |
| 1041 | DIABET THYROID ASSOCIATES | 2 | 0.133 |
| 1042 | DR MOHANS DIABET SPECIAL CTR | 2 | 0.133 |
| 1043 | EDOGAWA HOSP | 2 | 0.133 |
| 1044 | ENDOCRINOL RES CTR | 2 | 0.133 |
| 1045 | ENZYMOICS | 2 | 0.133 |
| 1046 | FDA | 2 | 0.133 |
| 1047 | FDN POLICLIN UNIV A GEMELLI IRCCS | 2 | 0.133 |
| 1048 | FIONA STANLEY HOSP | 2 | 0.133 |
| 1049 | FRIST CLIN | 2 | 0.133 |
| 1050 | FUKUSHIMA MED UNIV | 2 | 0.133 |
| 1051 | GALWAY UNIV HOSP | 2 | 0.133 |
| 1052 | GEN HOSP MURSKA SOBOTA | 2 | 0.133 |
| 1053 | GEN HOSP SIBENIK | 2 | 0.133 |
| 1054 | GERMAN CTR CARDIOVASC RES DZHK PARTNER SITE | 2 | 0.133 |
| 1055 | GERMAN CTR DIABET RES DZD | 2 | 0.133 |
| 1056 | GERMAN CTR DIABET RES DZD EV | 2 | 0.133 |
| 1057 | GUANGZHOU MED UNIV | 2 | 0.133 |
| 1058 | GUGLIELMO DA SALICETO HOSP | 2 | 0.133 |
| 1059 | GWANGJU INST SCI TECHNOL | 2 | 0.133 |
| 1060 | HADASSAH HEBREW UNIV | 2 | 0.133 |
| 1061 | HAMILTON HLTH SCI | 2 | 0.133 |
| 1062 | HARBIN MED UNIV | 2 | 0.133 |
| 1063 | HARVARD UNIV | 2 | 0.133 |
| 1064 | HASSELT UNIV | 2 | 0.133 |
| 1065 | HEART DIABET CTR | 2 | 0.133 |
| 1066 | HEINRICH HEINE UNIV | 2 | 0.133 |
| 1067 | HEINRICH HEINE UNIV DUSSELDORF | 2 | 0.133 |
| 1068 | HENRY FORD HLTH SYST | 2 | 0.133 |
| 1069 | HENRY FORD HOSP | 2 | 0.133 |
| 1070 | HEOR | 2 | 0.133 |
| 1071 | HLTH RES INST SANTIAGO DE COMPOSTELA | 2 | 0.133 |
| 1072 | HOSP BADALONA GERMANS TRIAS PUJOL | 2 | 0.133 |
| 1073 | HOSP UNIV BELLVITGE | 2 | 0.133 |
| 1074 | HOUSTON METHODIST HOSP | 2 | 0.133 |
| 1075 | HUMBER RIVER HOSP | 2 | 0.133 |
| 1076 | HUMBOLDT UNIV | 2 | 0.133 |
| 1077 | INOVA HEART VASC INST | 2 | 0.133 |
| 1078 | INST NACL CARDIOL IGNACIO CHAVEZ | 2 | 0.133 |
| 1079 | INST PASTEUR | 2 | 0.133 |
| 1080 | INST STAT MATH | 2 | 0.133 |
| 1081 | INST UNIV CARDIOL PNEUMOL QUEBEC | 2 | 0.133 |
| 1082 | IONIS PHARMACEUT | 2 | 0.133 |
| 1083 | IPGMER | 2 | 0.133 |
| 1084 | IQVIA INC | 2 | 0.133 |
| 1085 | IRCCS CASA SOLLIEVO SOFFERENZA | 2 | 0.133 |
| 1086 | IRCCS OSPED POLICLIN SAN MARTINO | 2 | 0.133 |
| 1087 | IRCCS SDN | 2 | 0.133 |
| 1088 | IST DIAGNOST VARELLI | 2 | 0.133 |
| 1089 | JAMIA HAMDARD | 2 | 0.133 |
| 1090 | JANSSEN INC | 2 | 0.133 |
| 1091 | JEAN VERDIER HOSP | 2 | 0.133 |
| 1092 | JINAN UNIV | 2 | 0.133 |
| 1093 | JINNOUCHI HOSP | 2 | 0.133 |
| 1094 | JOHN H STROGER JR HOSP COOK CTY | 2 | 0.133 |
| 1095 | JOHNS HOPKINS SCH MED | 2 | 0.133 |
| 1096 | JOHNSON JOHNSON | 2 | 0.133 |
| 1097 | JOHNSON JOHNSON PRIVATE LTD | 2 | 0.133 |
| 1098 | JR HIROSHIMA HOSP | 2 | 0.133 |
| 1099 | JUNTENDO TOKYO KOTO GERIATR MED CTR | 2 | 0.133 |
| 1100 | KAMEDA MED CTR | 2 | 0.133 |
| 1101 | KANAGAWA PHYS ASSOC | 2 | 0.133 |
| 1102 | KANAZAWA UNIV HOSP | 2 | 0.133 |
| 1103 | KANSAI ELECT POWER MED RES INST | 2 | 0.133 |
| 1104 | KANSAI MED UNIV | 2 | 0.133 |
| 1105 | KANSAI ROSAI HOSP | 2 | 0.133 |
| 1106 | KAOHSIUNG MED UNIV | 2 | 0.133 |
| 1107 | KAPODESTRIAN UNIV | 2 | 0.133 |
| 1108 | KAWASAKI HOSP | 2 | 0.133 |
| 1109 | KING SAUD UNIV | 2 | 0.133 |
| 1110 | KOBE RED CROSS HOSP | 2 | 0.133 |
| 1111 | KOC UNIV | 2 | 0.133 |
| 1112 | KOMAGOME HOSP | 2 | 0.133 |
| 1113 | KURIHARA CLIN | 2 | 0.133 |
| 1114 | KYOTO UNIV HOSP | 2 | 0.133 |
| 1115 | LEXICON PHARMACEUT INC | 2 | 0.133 |
| 1116 | LILAVATI HOSP RES CTR | 2 | 0.133 |
| 1117 | LISTER HOSP | 2 | 0.133 |
| 1118 | LORRAINE UNIV | 2 | 0.133 |
| 1119 | MAASTRICHT UNIV | 2 | 0.133 |
| 1120 | MADRAS DIABET RES FDN | 2 | 0.133 |
| 1121 | MAHIDOL UNIV | 2 | 0.133 |
| 1122 | MAIN LINE HLTH SYST | 2 | 0.133 |
| 1123 | MALCOM RANDALL VET ADM MED CTR | 2 | 0.133 |
| 1124 | MED INVEST INC | 2 | 0.133 |
| 1125 | MED UNIV S CAROLINA | 2 | 0.133 |
| 1126 | MEM SLOAN KETTERING CANC CTR | 2 | 0.133 |
| 1127 | MT SINAI SCH MED | 2 | 0.133 |
| 1128 | NAGASAKI UNIV | 2 | 0.133 |
| 1129 | NAGOYA CITY UNIV | 2 | 0.133 |
| 1130 | NANCHANG UNIV | 2 | 0.133 |
| 1131 | NATL CARDIOL HOSP | 2 | 0.133 |
| 1132 | NATL DEF MED CTR | 2 | 0.133 |
| 1133 | NATL HOSP ORG | 2 | 0.133 |
| 1134 | NATL SCH PUBL HLTH | 2 | 0.133 |
| 1135 | NATL UNIV IRELAND | 2 | 0.133 |
| 1136 | NATL UNIV SINGAPORE | 2 | 0.133 |
| 1137 | NEW YORK UNIV LANGONE HLTH | 2 | 0.133 |
| 1138 | NEWCASTLE UNIV | 2 | 0.133 |
| 1139 | NINEWELLS HOSP MED SCH | 2 | 0.133 |
| 1140 | NIPPON MED SCH | 2 | 0.133 |
| 1141 | NORTH BERWICK HLTH CTR | 2 | 0.133 |
| 1142 | NORWEGIAN UNIV SCI TECHNOL | 2 | 0.133 |
| 1143 | NOVEL GLOBAL COMMUNITY EDUC FDN | 2 | 0.133 |
| 1144 | OAKENHURST MED PRACTICE | 2 | 0.133 |
| 1145 | OCHSNER MED CTR | 2 | 0.133 |
| 1146 | ODENSE UNIV HOSP | 2 | 0.133 |
| 1147 | OHIO UNIV | 2 | 0.133 |
| 1148 | OSAKA POLICE HOSP | 2 | 0.133 |
| 1149 | OSAKA SAISEIKAI NAKATSU HOSP | 2 | 0.133 |
| 1150 | OSMANIA MED COLL HOSP | 2 | 0.133 |
| 1151 | OSPED MAGGIORE POLICLIN MILANO | 2 | 0.133 |
| 1152 | OTTO VON GUERICKE UNIV | 2 | 0.133 |
| 1153 | OXFORD CTR DIABET ENDOCRINOL METAB | 2 | 0.133 |
| 1154 | PALM BEACH ATLANTIC UNIV | 2 | 0.133 |
| 1155 | PARIS DESCARTES UNIV | 2 | 0.133 |
| 1156 | PARIS UNIV | 2 | 0.133 |
| 1157 | PENNINGTON BIOMED RES CTR | 2 | 0.133 |
| 1158 | PFIZER PHARMA GMBH | 2 | 0.133 |
| 1159 | PFIZER R D UK LTD | 2 | 0.133 |
| 1160 | PHARMERIT INT | 2 | 0.133 |
| 1161 | PRECIS HLTH ECON | 2 | 0.133 |
| 1162 | PRIVATE UNIV PRINCIPAL LIECHTENSTEIN | 2 | 0.133 |
| 1163 | PROFIL | 2 | 0.133 |
| 1164 | PROVIDENCE HLTH CARE | 2 | 0.133 |
| 1165 | PURDUE UNIV | 2 | 0.133 |
| 1166 | QUEEN MARY UNIV LONDON | 2 | 0.133 |
| 1167 | QUEENS UNIV BELFAST | 2 | 0.133 |
| 1168 | QUEENSLAND UNIV TECHNOL | 2 | 0.133 |
| 1169 | RANGSIT UNIV | 2 | 0.133 |
| 1170 | ROBERTSON CTR BIOSTAT CLIN TRIALS | 2 | 0.133 |
| 1171 | RONALD REAGAN UCLA MED CTR | 2 | 0.133 |
| 1172 | ROYAL MELBOURNE HOSP | 2 | 0.133 |
| 1173 | RUDOLFSTIFTUNG HOSP | 2 | 0.133 |
| 1174 | RUDOLFSTITUNG HOSP | 2 | 0.133 |
| 1175 | SAARLAND UNIV HOSP | 2 | 0.133 |
| 1176 | SAHLGRENS UNIV HOSP | 2 | 0.133 |
| 1177 | SALFORD ROYAL HOSP | 2 | 0.133 |
| 1178 | SAPIENZA UNIV ROME | 2 | 0.133 |
| 1179 | SAPIR COLL | 2 | 0.133 |
| 1180 | SCRIPPS CLIN | 2 | 0.133 |
| 1181 | SEMNAN UNIV MED SCI | 2 | 0.133 |
| 1182 | SHEBA MED CTR | 2 | 0.133 |
| 1183 | SHIGA UNIV MED SCI | 2 | 0.133 |
| 1184 | SHOWA UNIV | 2 | 0.133 |
| 1185 | SIMON FRASER UNIV | 2 | 0.133 |
| 1186 | SODER SJUKHUSET | 2 | 0.133 |
| 1187 | SOOCHOW UNIV | 2 | 0.133 |
| 1188 | SOONCHUNHYANG UNIV | 2 | 0.133 |
| 1189 | SOUTH TEXAS VET HLTH CARE SYST | 2 | 0.133 |
| 1190 | SOUTHEAST UNIV | 2 | 0.133 |
| 1191 | SOUTHERN TRUST | 2 | 0.133 |
| 1192 | ST MARYS HOSP | 2 | 0.133 |
| 1193 | ST VINCENTS UNIV HOSP | 2 | 0.133 |
| 1194 | STATENS SERUM INST | 2 | 0.133 |
| 1195 | SUNNYBROOK HLTH SCI CTR | 2 | 0.133 |
| 1196 | TATSUMI CLIN | 2 | 0.133 |
| 1197 | TEAM GESUNDHEIT GMBH | 2 | 0.133 |
| 1198 | TECH UNIV DRESDEN | 2 | 0.133 |
| 1199 | TECH UNIV MUNICH | 2 | 0.133 |
| 1200 | TEIKYO UNIV | 2 | 0.133 |
| 1201 | TEXAS TECH UNIV | 2 | 0.133 |
| 1202 | TIANJIN INST ENDOCRINOL | 2 | 0.133 |
| 1203 | TIMI STUDY GRP | 2 | 0.133 |
| 1204 | TOHOKU UNIV | 2 | 0.133 |
| 1205 | TOKUSHIMA UNIV HOSP | 2 | 0.133 |
| 1206 | TOKYO WOMENS MED UNIV | 2 | 0.133 |
| 1207 | TUNGHAI UNIV | 2 | 0.133 |
| 1208 | TURKU UNIV HOSP | 2 | 0.133 |
| 1209 | UAE UNIV | 2 | 0.133 |
| 1210 | UAM | 2 | 0.133 |
| 1211 | UCL MED SCH | 2 | 0.133 |
| 1212 | UDAYANA UNIV | 2 | 0.133 |
| 1213 | UMG | 2 | 0.133 |
| 1214 | UNIV ANTWERP | 2 | 0.133 |
| 1215 | UNIV ARKANSAS MED SCI | 2 | 0.133 |
| 1216 | UNIV AUCKLAND | 2 | 0.133 |
| 1217 | UNIV BORDEAUX | 2 | 0.133 |
| 1218 | UNIV CAPE TOWN | 2 | 0.133 |
| 1219 | UNIV CHINESE ACAD SCI | 2 | 0.133 |
| 1220 | UNIV CINCINNATI | 2 | 0.133 |
| 1221 | UNIV CLIN | 2 | 0.133 |
| 1222 | UNIV CLIN HOSP SANTIAGO DE COMPOSTELA | 2 | 0.133 |
| 1223 | UNIV COLORADO DENVER | 2 | 0.133 |
| 1224 | UNIV CRETE | 2 | 0.133 |
| 1225 | UNIV FED BAHIA | 2 | 0.133 |
| 1226 | UNIV G DANNUNZIO | 2 | 0.133 |
| 1227 | UNIV HOSP BERNE | 2 | 0.133 |
| 1228 | UNIV HOSP BIRMINGHAM NHS FDN TRUST | 2 | 0.133 |
| 1229 | UNIV HOSP HERAKLION | 2 | 0.133 |
| 1230 | UNIV HOSP SOUTHAMPTON NHS FDN TRUST | 2 | 0.133 |
| 1231 | UNIV HOSP ZURICH | 2 | 0.133 |
| 1232 | UNIV HULL | 2 | 0.133 |
| 1233 | UNIV IOWA | 2 | 0.133 |
| 1234 | UNIV IOWA HOSP CLIN | 2 | 0.133 |
| 1235 | UNIV KANSAS | 2 | 0.133 |
| 1236 | UNIV KIEL | 2 | 0.133 |
| 1237 | UNIV KLINIKUM LEIPZIG | 2 | 0.133 |
| 1238 | UNIV LAVAL | 2 | 0.133 |
| 1239 | UNIV LEEDS | 2 | 0.133 |
| 1240 | UNIV MAGNA GRECIA | 2 | 0.133 |
| 1241 | UNIV MANCHESTER | 2 | 0.133 |
| 1242 | UNIV MED BERLIN | 2 | 0.133 |
| 1243 | UNIV MED CTR LJUBLJANA | 2 | 0.133 |
| 1244 | UNIV MED CTR REGENSBURG | 2 | 0.133 |
| 1245 | UNIV MED PHARM CAROL DAVILA | 2 | 0.133 |
| 1246 | UNIV MESSINA | 2 | 0.133 |
| 1247 | UNIV MILANO BICOCCA | 2 | 0.133 |
| 1248 | UNIV NACL AUTONOMA MEXICO | 2 | 0.133 |
| 1249 | UNIV NOTTINGHAM | 2 | 0.133 |
| 1250 | UNIV PARMA | 2 | 0.133 |
| 1251 | UNIV PAVIA | 2 | 0.133 |
| 1252 | UNIV PENN HLTH SYST | 2 | 0.133 |
| 1253 | UNIV PERUGIA | 2 | 0.133 |
| 1254 | UNIV RENNES 1 | 2 | 0.133 |
| 1255 | UNIV ROME SAPIENZA | 2 | 0.133 |
| 1256 | UNIV SANTIAGO DE COMPOSTELA | 2 | 0.133 |
| 1257 | UNIV SIENA | 2 | 0.133 |
| 1258 | UNIV SOUTH CAROLINA | 2 | 0.133 |
| 1259 | UNIV TEXAS SAN ANTONIO | 2 | 0.133 |
| 1260 | UNIV TOKUSHIMA | 2 | 0.133 |
| 1261 | UNIV TOYAMA | 2 | 0.133 |
| 1262 | UNIV TSUKUBA | 2 | 0.133 |
| 1263 | UNIV TUBINGEN | 2 | 0.133 |
| 1264 | UNIV ZURICH | 2 | 0.133 |
| 1265 | US FDA | 2 | 0.133 |
| 1266 | UT HLTH SAN ANTONIO | 2 | 0.133 |
| 1267 | VALL DHEBRON RES INST VHIR | 2 | 0.133 |
| 1268 | VASDHS | 2 | 0.133 |
| 1269 | VASTRA GOTALANDSREGIONEN | 2 | 0.133 |
| 1270 | VET AFFAIRS SAN DIEGO HEALTHCARE SYST | 2 | 0.133 |
| 1271 | VIVIT | 2 | 0.133 |
| 1272 | WAKE FOREST SCH MED | 2 | 0.133 |
| 1273 | WARSAW MED UNIV | 2 | 0.133 |
| 1274 | WAYNE STATE UNIV | 2 | 0.133 |
| 1275 | WEIFANG MED UNIV | 2 | 0.133 |
| 1276 | WEILL CORNELL MED | 2 | 0.133 |
| 1277 | WEILL CORNELL MED COLL | 2 | 0.133 |
| 1278 | WELCH CTR PREVENT EPIDEMIOL CLIN RES | 2 | 0.133 |
| 1279 | WEST HERTS HOSP NHS TRUST | 2 | 0.133 |
| 1280 | WESTERN SYDNEY UNIV | 2 | 0.133 |
| 1281 | WESTMEAD HOSP | 2 | 0.133 |
| 1282 | WOMENS COLL HOSP | 2 | 0.133 |
| 1283 | YACHIYO HOSP | 2 | 0.133 |
| 1284 | 2KMM SP ZOO | 1 | 0.066 |
| 1285 | 424 GEN MIL HOSP | 1 | 0.066 |
| 1286 | AALBORG UNIV HOSP | 1 | 0.066 |
| 1287 | ABC FDN | 1 | 0.066 |
| 1288 | ABINGTON JEFFERSON HLTH | 1 | 0.066 |
| 1289 | ACAD ATHENS | 1 | 0.066 |
| 1290 | ACAD HLTH SYST | 1 | 0.066 |
| 1291 | ACAD SCI INNOVAT RES ACSIR | 1 | 0.066 |
| 1292 | ACAD TEACHING HOSP FELDKIRCH | 1 | 0.066 |
| 1293 | ACERRA | 1 | 0.066 |
| 1294 | ADIS | 1 | 0.066 |
| 1295 | ADMIRALTY MED CTR | 1 | 0.066 |
| 1296 | ADV CTR DIABET ENDOCRINE CARE | 1 | 0.066 |
| 1297 | ADVENTHLTH TRANSLAT RES INST | 1 | 0.066 |
| 1298 | AETION | 1 | 0.066 |
| 1299 | AFFILIATED HOSP SW MED UNIV | 1 | 0.066 |
| 1300 | AFFILIATED HOSP ZUNYI MED COLL | 1 | 0.066 |
| 1301 | AGCY SCI TECHNOL RES | 1 | 0.066 |
| 1302 | AGHIA SOPHIA CHILDRENS HOSP | 1 | 0.066 |
| 1303 | AHEPA UNIV HOSP | 1 | 0.066 |
| 1304 | AIIMS | 1 | 0.066 |
| 1305 | AIM SPECIALTY HLTH | 1 | 0.066 |
| 1306 | AL AZHAR UNIV | 1 | 0.066 |
| 1307 | ALABAMA COLL OSTEOPATH MED | 1 | 0.066 |
| 1308 | ALBANY COLL PHARM HLTH SCI | 1 | 0.066 |
| 1309 | ALBANY MED CTR | 1 | 0.066 |
| 1310 | ALBERT EINSTEIN MED CTR | 1 | 0.066 |
| 1311 | ALBERTA COLL FAMILY PHYS | 1 | 0.066 |
| 1312 | ALEXANDRIA UNIV | 1 | 0.066 |
| 1313 | ALFRED HLTH | 1 | 0.066 |
| 1314 | ALL INDIA INST MED SCI | 1 | 0.066 |
| 1315 | ALMAZOV NATL MED RES CTR | 1 | 0.066 |
| 1316 | AMER COLL PHYSICIANS | 1 | 0.066 |
| 1317 | AMER DIABET ASSOC | 1 | 0.066 |
| 1318 | AMER HEART ASSOC | 1 | 0.066 |
| 1319 | AMER UNIV BEIRUT MED CTR | 1 | 0.066 |
| 1320 | AMGEN BULGARIA EOOD | 1 | 0.066 |
| 1321 | AMGEN INC | 1 | 0.066 |
| 1322 | AMITY UNIV | 1 | 0.066 |
| 1323 | AMRITA VISWA VIDYAPEETHAM UNIV | 1 | 0.066 |
| 1324 | AMSTERDAM UMC | 1 | 0.066 |
| 1325 | AMSTERDAM UNIV MED CTR | 1 | 0.066 |
| 1326 | ANAL IATRIKI SA | 1 | 0.066 |
| 1327 | ANKARA YILDIRIM BEYAZIT UNIV | 1 | 0.066 |
| 1328 | ANMCO RES CTR | 1 | 0.066 |
| 1329 | ANTAKYA STATE HOSP | 1 | 0.066 |
| 1330 | ANTHEM INC | 1 | 0.066 |
| 1331 | ANTI DOPING LAB QATAR | 1 | 0.066 |
| 1332 | ANTIDOPING LAB QATAR | 1 | 0.066 |
| 1333 | ANTONIUS ZIEKENHUIS SNEEK | 1 | 0.066 |
| 1334 | AOKI INTERNAL MED DIGEST CLIN | 1 | 0.066 |
| 1335 | AORN ST ANNA SAN SEBASTIANO HOSP | 1 | 0.066 |
| 1336 | APOLLO GLENEAGLES HOSP | 1 | 0.066 |
| 1337 | APOLLO HOSP | 1 | 0.066 |
| 1338 | ARCHIMEDES INC | 1 | 0.066 |
| 1339 | ARIEL UNIV | 1 | 0.066 |
| 1340 | ARIZONA GRAND MED CTR | 1 | 0.066 |
| 1341 | ARMY MIL MED UNIV | 1 | 0.066 |
| 1342 | ARROWHEAD REG MED CTR | 1 | 0.066 |
| 1343 | ARTHUR ASIRVATHAM HOSP | 1 | 0.066 |
| 1344 | ASAHI ROSAI HOSP | 1 | 0.066 |
| 1345 | ASANO KANAMACHI CLIN | 1 | 0.066 |
| 1346 | ASCENS MED GRP | 1 | 0.066 |
| 1347 | ASIA UNIV | 1 | 0.066 |
| 1348 | ASST NORD MILANO | 1 | 0.066 |
| 1349 | ASST PAPA GIOVANNI XXIII | 1 | 0.066 |
| 1350 | ASST SPEDALI CIVILI | 1 | 0.066 |
| 1351 | ASSUTA ASHDOD ACAD MED CTR | 1 | 0.066 |
| 1352 | ASTELLAS PHARMA EUROPE BV | 1 | 0.066 |
| 1353 | ASTELLAS PHARMA GLOBAL DEV | 1 | 0.066 |
| 1354 | ASTELLAS PHARMA GLOBAL DEV INC | 1 | 0.066 |
| 1355 | ASTELLAS PHARRNA INC | 1 | 0.066 |
| 1356 | ASTRAZENECA BRAZIL | 1 | 0.066 |
| 1357 | ASTRAZENECA BV | 1 | 0.066 |
| 1358 | ASTRAZENECA FARMACEUT SPAIN | 1 | 0.066 |
| 1359 | ASTRAZENECA KK | 1 | 0.066 |
| 1360 | ASTRAZENECA NORD BALTIC | 1 | 0.066 |
| 1361 | ASTRAZENECA NORDIC BALT | 1 | 0.066 |
| 1362 | ASTRAZENECA NORDIC BALTIC | 1 | 0.066 |
| 1363 | ASTRAZENECA UK | 1 | 0.066 |
| 1364 | ASTRAZENECA UK LTD | 1 | 0.066 |
| 1365 | ATASAM HOSP | 1 | 0.066 |
| 1366 | ATHENS MED SCH | 1 | 0.066 |
| 1367 | ATHENS NAVAL HOSP | 1 | 0.066 |
| 1368 | ATHENS UNIV HOSP ATTIKON | 1 | 0.066 |
| 1369 | ATTIKON UNIV HOSP | 1 | 0.066 |
| 1370 | AUCKLAND HOSP | 1 | 0.066 |
| 1371 | AUGUSTA UNIV | 1 | 0.066 |
| 1372 | AULSS 4 VENETO | 1 | 0.066 |
| 1373 | AUSL | 1 | 0.066 |
| 1374 | AZIENDA ASSISTENZA SANITARIA N 5 FRIULI OCCIDENTA | 1 | 0.066 |
| 1375 | AZIENDA OSPED RILIEVO NAZL ALTA SPECIALIZZA GAR | 1 | 0.066 |
| 1376 | AZIENDA OSPED UNIV | 1 | 0.066 |
| 1377 | AZIENDA OSPED UNIV CAREGGI | 1 | 0.066 |
| 1378 | AZIENDA OSPED UNIV CATANZARO | 1 | 0.066 |
| 1379 | AZIENDA OSPED UNIV FERRARA | 1 | 0.066 |
| 1380 | AZIENDA OSPED UNIV INTEGRATA VERONA | 1 | 0.066 |
| 1381 | AZIENDA OSPED UNIV LUIGI VANVITELLI | 1 | 0.066 |
| 1382 | AZIENDA OSPED UNIV MAGGIORE CARITA NOVARA | 1 | 0.066 |
| 1383 | AZIENDA OSPED UNIV MATER DOMINI | 1 | 0.066 |
| 1384 | AZIENDA OSPED UNIV OSPED RIUNITI ANCONA | 1 | 0.066 |
| 1385 | AZIENDA OSPED UNIV POLICLIN G MARTINO MESSINA | 1 | 0.066 |
| 1386 | AZIENDA OSPED UNIV POLICLIN UMBERTO I | 1 | 0.066 |
| 1387 | AZIENDA OSPED UNIV SAN LUIGI GONZAGA | 1 | 0.066 |
| 1388 | AZIENDA SANIT LOCALE FOGGIA | 1 | 0.066 |
| 1389 | AZIENDA SANIT LOCALE LECCE | 1 | 0.066 |
| 1390 | AZIENDA SANIT LOCALE NAPOLI 1 CTR | 1 | 0.066 |
| 1391 | AZIENDA SANIT LOCALE NAPOLI 2 NORD | 1 | 0.066 |
| 1392 | AZIENDA SANIT LOCALE TARANTO | 1 | 0.066 |
| 1393 | AZIENDA SANIT LOCALE VERBANO CUSIO OSSOLA | 1 | 0.066 |
| 1394 | AZIENDA SANIT PROV AUTONOMA BOLZANO | 1 | 0.066 |
| 1395 | AZIENDA SANIT PROV COSENZA | 1 | 0.066 |
| 1396 | AZIENDA SANIT PROV SIRACUSA | 1 | 0.066 |
| 1397 | AZIENDA SANIT UNIV INTEGRATA TRIESTE | 1 | 0.066 |
| 1398 | AZIENDA SANITARIA LOCALE LATINA | 1 | 0.066 |
| 1399 | AZIENDA SANITARIA LOCALE ROMA 2 | 1 | 0.066 |
| 1400 | AZIENDA SOCIO SANIT TERR BERGAMO EST | 1 | 0.066 |
| 1401 | AZIENDA SOCIO SANIT TERR BERGAMO OVEST | 1 | 0.066 |
| 1402 | AZIENDA SOCIO SANIT TERR FATEBENEFRATELLI SACCO | 1 | 0.066 |
| 1403 | AZIENDA SOCIO SANIT TERR PAPA GIOVANNI XXIII | 1 | 0.066 |
| 1404 | AZIENDA SOCIO SANIT TERR SETTE LAGHI | 1 | 0.066 |
| 1405 | AZIENDA SOCIO SANIT TERR VALLE OLONA | 1 | 0.066 |
| 1406 | AZIENDA ULSS N 4 VENETO ORIENTALE | 1 | 0.066 |
| 1407 | AZIENDA ULSS N 6 EUGANEA | 1 | 0.066 |
| 1408 | AZIENDA UNIV POLICLIN PAOLO GIACCONE PALERMO | 1 | 0.066 |
| 1409 | AZIENDA USL LATINA | 1 | 0.066 |
| 1410 | AZIENDA USL TOSCANA CTR | 1 | 0.066 |
| 1411 | BADR UNIV CAIRO | 1 | 0.066 |
| 1412 | BAERUM HOSP VESTRE VIKEN HF | 1 | 0.066 |
| 1413 | BAJCSY ZSILINSZKY TEACHING HOSP | 1 | 0.066 |
| 1414 | BAKER GILMOUR CARDIOVASC INST | 1 | 0.066 |
| 1415 | BAMET GEN HOSP | 1 | 0.066 |
| 1416 | BANGALORE DIABET DIAGNOST CTR | 1 | 0.066 |
| 1417 | BAQIYATALLAH UNIV MED SCI | 1 | 0.066 |
| 1418 | BAR ILAN UNIV | 1 | 0.066 |
| 1419 | BARCELONA AUTONOMOUS UNIV UAB | 1 | 0.066 |
| 1420 | BARNET ENFIELD HARINGEY MENTAL HLTH TRUST | 1 | 0.066 |
| 1421 | BASSETT MED CTR | 1 | 0.066 |
| 1422 | BAYLOR SCOTT WHITE HLTH | 1 | 0.066 |
| 1423 | BAYLOR SCOTT WHITE MED CTR | 1 | 0.066 |
| 1424 | BEAUMONT HOSP | 1 | 0.066 |
| 1425 | BEIJING YANQING HOSP | 1 | 0.066 |
| 1426 | BEN GURION UNIV NEGEV | 1 | 0.066 |
| 1427 | BERLIN BRANDENBURG CTR REGENERAT THERAPIES | 1 | 0.066 |
| 1428 | BERLIN BRANDENBURG CTR REGENERAT THERAPIES BCRT | 1 | 0.066 |
| 1429 | BERLIN BRANDENBURG INST HLTH | 1 | 0.066 |
| 1430 | BERLIN INST HLTH | 1 | 0.066 |
| 1431 | BETHEL CLIN EVKB | 1 | 0.066 |
| 1432 | BETHESDA DIABET RES CTR | 1 | 0.066 |
| 1433 | BHATIA HOSP | 1 | 0.066 |
| 1434 | BHF GLASGOW CARDIOVASC RES CTR | 1 | 0.066 |
| 1435 | BICESTER HLTH CTR | 1 | 0.066 |
| 1436 | BIOACT FOOD GMBH | 1 | 0.066 |
| 1437 | BIOACT FOOD GMBH HV | 1 | 0.066 |
| 1438 | BIOINNOVAT CTR | 1 | 0.066 |
| 1439 | BIOMED HELSINKI | 1 | 0.066 |
| 1440 | BIOMEDICUM HELSINKI | 1 | 0.066 |
| 1441 | BIOPHARMACEUT R D | 1 | 0.066 |
| 1442 | BIOPHARMACEUTICALS R D | 1 | 0.066 |
| 1443 | BIRLA INST TECHNOL SCI BITS PILANI | 1 | 0.066 |
| 1444 | BIRMINGHAM HEARTLANDS HOSP | 1 | 0.066 |
| 1445 | BIRMINGHAM VA MED CTR | 1 | 0.066 |
| 1446 | BJORKNES COLL | 1 | 0.066 |
| 1447 | BLACKTOWN HOSP | 1 | 0.066 |
| 1448 | BLUE CROSS BLUE SHIELD LOUISIANA | 1 | 0.066 |
| 1449 | BMC HOSP DIABET CTR | 1 | 0.066 |
| 1450 | BOEHRINGER INGELHEIM CHINA INVEST CO LTD | 1 | 0.066 |
| 1451 | BOEHRINGER INGELHEIM CHINA INVESTMENT CO LTD | 1 | 0.066 |
| 1452 | BOEHRINGER INGELHEIM DANMARK AS | 1 | 0.066 |
| 1453 | BOEHRINGER INGELHEIM ESPANA | 1 | 0.066 |
| 1454 | BOEHRINGER INGELHEIM ESPANA SA | 1 | 0.066 |
| 1455 | BOEHRINGER INGELHEIM FINLAND KY | 1 | 0.066 |
| 1456 | BOEHRINGER INGELHEIM FRANCE SAS | 1 | 0.066 |
| 1457 | BOEHRINGER INGELHEIM HELLAS | 1 | 0.066 |
| 1458 | BOEHRINGER INGELHEIM KS | 1 | 0.066 |
| 1459 | BOEHRINGER INGELHEIM PVT LTD | 1 | 0.066 |
| 1460 | BOGOMOLETS NATL MED UNIV | 1 | 0.066 |
| 1461 | BOGOMOLETZ INST PHYSIOL | 1 | 0.066 |
| 1462 | BON SECOURS HEART VASC INST | 1 | 0.066 |
| 1463 | BON SECOURS HOSP | 1 | 0.066 |
| 1464 | BOSTON MCPHS UNIV | 1 | 0.066 |
| 1465 | BRIGHAM WOMANS HOSP | 1 | 0.066 |
| 1466 | BRISTOL MYERS SQUIBB PHARMACEUT | 1 | 0.066 |
| 1467 | BRISTOL MYERS SQUIBB PHARMACEUT LTD | 1 | 0.066 |
| 1468 | BRITISH UNIV EGYPT | 1 | 0.066 |
| 1469 | BUTLER UNIV | 1 | 0.066 |
| 1470 | CAEN UNIV HOSP | 1 | 0.066 |
| 1471 | CALIF STATE UNIV MONTEREY BAY | 1 | 0.066 |
| 1472 | CALVIN COLL | 1 | 0.066 |
| 1473 | CAMPBELL UNIV | 1 | 0.066 |
| 1474 | CAMPBELLTOWN HOSP | 1 | 0.066 |
| 1475 | CAMPUS BIOMED UNIV | 1 | 0.066 |
| 1476 | CANBERRA HOSP | 1 | 0.066 |
| 1477 | CARDIOL CLIN INTERVENCIONISTA TARASCOS | 1 | 0.066 |
| 1478 | CARDIOL CONSULTANTS | 1 | 0.066 |
| 1479 | CARDIOMETAB INST | 1 | 0.066 |
| 1480 | CARDIOVASC RES UCSF FRESNO CENT SAN JOAQUIN VALLE | 1 | 0.066 |
| 1481 | CARILION CLIN | 1 | 0.066 |
| 1482 | CARLOS III RES INST | 1 | 0.066 |
| 1483 | CAROL DAVILA UNIV | 1 | 0.066 |
| 1484 | CATHOLIC KWANDONG UNIV | 1 | 0.066 |
| 1485 | CATHOLIC UNIV LOUVAIN | 1 | 0.066 |
| 1486 | CEDARVILLE UNIV | 1 | 0.066 |
| 1487 | CENT FINLAND CENT HOSP | 1 | 0.066 |
| 1488 | CENT HOSP LINYI CITY | 1 | 0.066 |
| 1489 | CENT HOSP YAOUNDE | 1 | 0.066 |
| 1490 | CENT OHIO POISON CTR | 1 | 0.066 |
| 1491 | CENT S UNIV | 1 | 0.066 |
| 1492 | CENT UNIV JAMMU | 1 | 0.066 |
| 1493 | CHAIM SHEBA MED CTR | 1 | 0.066 |
| 1494 | CHALEUR REG HOSP | 1 | 0.066 |
| 1495 | CHALMERS UNIV TECHNOL | 1 | 0.066 |
| 1496 | CHARITE CAMPUS BENJAMIN FRANKLIN | 1 | 0.066 |
| 1497 | CHARITE CAMPUS CVK | 1 | 0.066 |
| 1498 | CHARITE UNIV MED BERLIN CVK | 1 | 0.066 |
| 1499 | CHARLES PERKINS CTR | 1 | 0.066 |
| 1500 | CHARLOTTE HEART GRP RES CTR | 1 | 0.066 |
| 1501 | CHEIKH ANTA DIOP UNIV | 1 | 0.066 |
| 1502 | CHELLARAM DIABET INST | 1 | 0.066 |
| 1503 | CHENG HSIN GEN HOSP | 1 | 0.066 |
| 1504 | CHENGDU UNIV TRADIT CHINESE MED | 1 | 0.066 |
| 1505 | CHIBA UNIV HOSP | 1 | 0.066 |
| 1506 | CHICAGO ADVOCATE HLTH HOSP | 1 | 0.066 |
| 1507 | CHILDRENS HOSP BULT | 1 | 0.066 |
| 1508 | CHILLIWACK GEN HOSP | 1 | 0.066 |
| 1509 | CHINA ACAD CHINESE MED SCI | 1 | 0.066 |
| 1510 | CHINESE ACAD MED SCI PEKING UNION MED COLL | 1 | 0.066 |
| 1511 | CHINESE PEOPLES LIBERAT ARMY GEN HOSP | 1 | 0.066 |
| 1512 | CHINESE PLA MED COLL | 1 | 0.066 |
| 1513 | CHONGQING MED UNIV | 1 | 0.066 |
| 1514 | CHRISTUS SPOHN HOSP CORPUS CHRISTI | 1 | 0.066 |
| 1515 | CHRU | 1 | 0.066 |
| 1516 | CHU CAEN | 1 | 0.066 |
| 1517 | CHU DUMONT UHC | 1 | 0.066 |
| 1518 | CHU LIEGE SART TILMAN B35 | 1 | 0.066 |
| 1519 | CHU LILLE | 1 | 0.066 |
| 1520 | CHU VAUDOIS | 1 | 0.066 |
| 1521 | CHUF | 1 | 0.066 |
| 1522 | CHULABHORN HOSP | 1 | 0.066 |
| 1523 | CHULALONGKORN UNIV | 1 | 0.066 |
| 1524 | CHUNG SHAN MED UNIV | 1 | 0.066 |
| 1525 | CI PARHON UNIV HOSP | 1 | 0.066 |
| 1526 | CIBER DIABET ENFERMEDADES METAB ASOCIADAS CIBER | 1 | 0.066 |
| 1527 | CIBERDEM DIABET ASSOCIATED METAB DIS NETWORKING | 1 | 0.066 |
| 1528 | CIC BORDEAUX CICI1401 | 1 | 0.066 |
| 1529 | CINTESIS CARDIOCARE | 1 | 0.066 |
| 1530 | CIT HUIZHOU HOSP | 1 | 0.066 |
| 1531 | CITY HOPE NATL MED CTR | 1 | 0.066 |
| 1532 | CIUDAD REAL GEN UNIV HOSP | 1 | 0.066 |
| 1533 | CIVIL HOSP | 1 | 0.066 |
| 1534 | CLALIT HLTH SERV | 1 | 0.066 |
| 1535 | CLEMENT J ZABLOCKI VET AFFAIRS MED CTR | 1 | 0.066 |
| 1536 | CLEVELAND CLIN ABU DHABI | 1 | 0.066 |
| 1537 | CLIN CTR | 1 | 0.066 |
| 1538 | CLIN CTR SERBIA | 1 | 0.066 |
| 1539 | CLIN ENDOCRINOL EDUC RES ACEER | 1 | 0.066 |
| 1540 | CLIN HOSP | 1 | 0.066 |
| 1541 | CLIN HOSP MERKUR | 1 | 0.066 |
| 1542 | CLIN HOSP SISTERS MERCY | 1 | 0.066 |
| 1543 | CLIN LA ESPERANZA | 1 | 0.066 |
| 1544 | CLIN ST MARIEN | 1 | 0.066 |
| 1545 | CLIN TRIAL SERV UNIT | 1 | 0.066 |
| 1546 | CLIN UNIV BRUXELLES | 1 | 0.066 |
| 1547 | CNRS | 1 | 0.066 |
| 1548 | CODES COMMUNITY DIABET EDUC SUPPORT | 1 | 0.066 |
| 1549 | COLL MED VET LIFE SCI | 1 | 0.066 |
| 1550 | COLORADO SCH PUBL HLTH | 1 | 0.066 |
| 1551 | COLUMBIA UNIV COLL PHYS SURG | 1 | 0.066 |
| 1552 | COMPLEJO HOSP UNIV DE SANTIAGO | 1 | 0.066 |
| 1553 | COMPLEJO HOSP UNIV LEON | 1 | 0.066 |
| 1554 | COMPLEJO HOSP UNIV SANTIAGO | 1 | 0.066 |
| 1555 | CONNCORD REPATRIAT GEN HOSP | 1 | 0.066 |
| 1556 | CONSORCIO HOSP GEN VALENCIA | 1 | 0.066 |
| 1557 | CONSUMER REPRESENTAT | 1 | 0.066 |
| 1558 | COOK CTY HLTH HOSP SYST | 1 | 0.066 |
| 1559 | COPENHAGEN MED UNIV | 1 | 0.066 |
| 1560 | CORDOBA UNIV | 1 | 0.066 |
| 1561 | CORESEARCH | 1 | 0.066 |
| 1562 | CPC CLIN RES | 1 | 0.066 |
| 1563 | CRECON MED ASSESSMENT INC | 1 | 0.066 |
| 1564 | CREDIT VALLEY HOSP | 1 | 0.066 |
| 1565 | CREST | 1 | 0.066 |
| 1566 | CRIN INI CRCT CARDIOVASC RENAL CLIN TRIALISTS | 1 | 0.066 |
| 1567 | CRUCES UNIV HOSP | 1 | 0.066 |
| 1568 | CSIR CENT DRUG RES INST | 1 | 0.066 |
| 1569 | CTR ESPANOL INVEST FARMACOEPIDEMIOL | 1 | 0.066 |
| 1570 | CTR ESTUDOS SUPER MACEIO | 1 | 0.066 |
| 1571 | CTR HOSP LANAUDIERE | 1 | 0.066 |
| 1572 | CTR HOSP REG UNIV BESANCON | 1 | 0.066 |
| 1573 | CTR HOSP ST JOSEPH ST LUC | 1 | 0.066 |
| 1574 | CTR HOSP UNIV COIMBRA | 1 | 0.066 |
| 1575 | CTR HOSP UNIV LISBOA NORTE | 1 | 0.066 |
| 1576 | CTR HOSP UNIV SAO JOAO | 1 | 0.066 |
| 1577 | CTR HOSP VILA NOVA GAIA ESPINHO | 1 | 0.066 |
| 1578 | CTR INVEST BIOMED DIABET ENFERMEDADES METAB ASO | 1 | 0.066 |
| 1579 | CTR INVEST BIOMED RED DIABET ENFERMEDADES CIBER | 1 | 0.066 |
| 1580 | CTR INVEST BIOMED RED ENFERMEDADES CARDIOVASC | 1 | 0.066 |
| 1581 | CTR OUTCOMES RES EVALUAT RI MUHC | 1 | 0.066 |
| 1582 | CTR RECH CORDELIERS | 1 | 0.066 |
| 1583 | CTR REGISTERS | 1 | 0.066 |
| 1584 | CTR STROKE RES | 1 | 0.066 |
| 1585 | CTR UNIV SANTE MCGILL | 1 | 0.066 |
| 1586 | CURTIN UNIV | 1 | 0.066 |
| 1587 | CVS PHARM | 1 | 0.066 |
| 1588 | CYGNUS HOSP | 1 | 0.066 |
| 1589 | CYPRUS UNIV TECHNOL | 1 | 0.066 |
| 1590 | DALTON CARDIOVASC RES CTR | 1 | 0.066 |
| 1591 | DANKOOK UNIV | 1 | 0.066 |
| 1592 | DARTMOUTH HITCHCOCK MED CTR | 1 | 0.066 |
| 1593 | DEAKIN UNIV | 1 | 0.066 |
| 1594 | DEF HLTH AGCY | 1 | 0.066 |
| 1595 | DELTA UNIV SCI TECHNOL | 1 | 0.066 |
| 1596 | DEPT BIOMED SCI BIOTECHNOL | 1 | 0.066 |
| 1597 | DEPT CARDIOMETABOL DIS RES | 1 | 0.066 |
| 1598 | DEPT CLIN EXPT MED | 1 | 0.066 |
| 1599 | DEPT CLIN SCI EDUC | 1 | 0.066 |
| 1600 | DEPT MED AGING SCI | 1 | 0.066 |
| 1601 | DEPT SURG MED MOL CRIT AREA PATHOL | 1 | 0.066 |
| 1602 | DEPT VET AFFAIRS EASTERN KANSAS HLTH CARE SYST | 1 | 0.066 |
| 1603 | DEUTSCHES ZENTRUM HERZ KREISLAUF FORSCH DZHK | 1 | 0.066 |
| 1604 | DIABET CARE RES CTR | 1 | 0.066 |
| 1605 | DIABET CTR CHILDREN ADOLESCENTS | 1 | 0.066 |
| 1606 | DIABET DIV | 1 | 0.066 |
| 1607 | DIABET HEART RES CTR | 1 | 0.066 |
| 1608 | DIABET INST | 1 | 0.066 |
| 1609 | DIABET NIERENZENTRUM | 1 | 0.066 |
| 1610 | DIABET SCHWERPUNKTPRAXIS ROSENHEIM | 1 | 0.066 |
| 1611 | DIABET ZENTRUM BAD LAUTERBERG | 1 | 0.066 |
| 1612 | DIABETES360 HLTH CTR | 1 | 0.066 |
| 1613 | DIACARE DIABET CARE HORMONE CLIN | 1 | 0.066 |
| 1614 | DIACON HOSP | 1 | 0.066 |
| 1615 | DIAMEL | 1 | 0.066 |
| 1616 | DIPARTIMENTO MED | 1 | 0.066 |
| 1617 | DIV ENDOCRINOL METAB GENET | 1 | 0.066 |
| 1618 | DMC SPECIALTY CTR HEART HOSP | 1 | 0.066 |
| 1619 | DONGGUK UNIV SEOUL | 1 | 0.066 |
| 1620 | DOW UNIV HLTH SCI | 1 | 0.066 |
| 1621 | DUBAI HOSP | 1 | 0.066 |
| 1622 | DUBRAVA UNIV HOSP | 1 | 0.066 |
| 1623 | DUHS | 1 | 0.066 |
| 1624 | DUKE NUS MED SCH | 1 | 0.066 |
| 1625 | DUKE SR AHEC | 1 | 0.066 |
| 1626 | DUKE UNIV HOSP | 1 | 0.066 |
| 1627 | DUPUYTREN UNIV HOSP | 1 | 0.066 |
| 1628 | DUZCE UNIV | 1 | 0.066 |
| 1629 | DUZCE UNIV TIP | 1 | 0.066 |
| 1630 | EAST COAST INST RES | 1 | 0.066 |
| 1631 | EASTERN NEPHROL ASSOCIATES | 1 | 0.066 |
| 1632 | EASTERN VIRGINIA MED SCH | 1 | 0.066 |
| 1633 | EBERHARD KARLS UNIV TUBINGEN | 1 | 0.066 |
| 1634 | EDC CTR DIABET EDUC | 1 | 0.066 |
| 1635 | EFFECT EVIDENCE | 1 | 0.066 |
| 1636 | ELI LILLY CO ESPANA | 1 | 0.066 |
| 1637 | ELI LILLY JAPAN KK | 1 | 0.066 |
| 1638 | ELI LILLY SUISSE SA | 1 | 0.066 |
| 1639 | ELISABETH HOSP | 1 | 0.066 |
| 1640 | ELSEVIER | 1 | 0.066 |
| 1641 | EMERGENCY CTY CLIN HOSP | 1 | 0.066 |
| 1642 | EMERGENCY INST CARDIOVASC DIS | 1 | 0.066 |
| 1643 | EMERGENCY INST CARDIOVASC DIS PROF CC ILIESCU | 1 | 0.066 |
| 1644 | EMORY HEART DIS PREVENT CTR | 1 | 0.066 |
| 1645 | ENDOCRINE METAB CONSULTANTS | 1 | 0.066 |
| 1646 | ENDOCRINE METAB DISORDERS INST | 1 | 0.066 |
| 1647 | ENDONET | 1 | 0.066 |
| 1648 | ENGLEWOOD HOSP MED CTR | 1 | 0.066 |
| 1649 | EPIDEMIOL STUDIES UNIT | 1 | 0.066 |
| 1650 | EPS CORP | 1 | 0.066 |
| 1651 | EPSOM ST HELIER UNIV HOSP NHS TRUST | 1 | 0.066 |
| 1652 | EQUATION AB | 1 | 0.066 |
| 1653 | ERASMUS MC | 1 | 0.066 |
| 1654 | ES HLTH SCI FDN | 1 | 0.066 |
| 1655 | ESKENAZI HLTH | 1 | 0.066 |
| 1656 | ESTABLISHED INVESTIGATORS CONICET | 1 | 0.066 |
| 1657 | ESTUDIOS CLIN LATINO AMER | 1 | 0.066 |
| 1658 | EUROPEAN GENOM INST DIABET | 1 | 0.066 |
| 1659 | EUROPEAN SOC CARDIOL | 1 | 0.066 |
| 1660 | EVAGGELISMOS GEN HOSP ATHENS | 1 | 0.066 |
| 1661 | EVANGELISMOS MED CTR | 1 | 0.066 |
| 1662 | EVROSTON LP | 1 | 0.066 |
| 1663 | F CRIN NETWORK | 1 | 0.066 |
| 1664 | F HOFFMANN LA ROCHE LTD | 1 | 0.066 |
| 1665 | FAC FARM | 1 | 0.066 |
| 1666 | FAC MED | 1 | 0.066 |
| 1667 | FAIRLEIGH DICKINSON UNIV | 1 | 0.066 |
| 1668 | FARRER PK HOSP | 1 | 0.066 |
| 1669 | FAVALORO FDN | 1 | 0.066 |
| 1670 | FCRIN INI CRCTC | 1 | 0.066 |
| 1671 | FDN BIOMED RES INNOVAT KOBE | 1 | 0.066 |
| 1672 | FDN FAVALORO | 1 | 0.066 |
| 1673 | FDN IRCCS CA GRANDA OSPED MAGGIORE POLICLIN MILAN | 1 | 0.066 |
| 1674 | FDN IRCCS POLICLIN SAN MATTEO | 1 | 0.066 |
| 1675 | FDN JIMENEZ DIAZ UAM | 1 | 0.066 |
| 1676 | FDN POLICLIN UNIV A GEMELLI | 1 | 0.066 |
| 1677 | FDN POLICLIN UNIV A GERELLI IRCCS | 1 | 0.066 |
| 1678 | FDN POLICLIN UNIV AGOSTINO GEMELLI IRCCS | 1 | 0.066 |
| 1679 | FDN RENAL INIGO DE ALVAREZ TOLEDO IRSIN C JOSE AB | 1 | 0.066 |
| 1680 | FDN STUDY HYPERTENS CARDIOVASC RISK | 1 | 0.066 |
| 1681 | FED INST DRUGS MED DEVICES | 1 | 0.066 |
| 1682 | FED INST EDUC SCI TECHNOL IFES | 1 | 0.066 |
| 1683 | FEDER II UNIV NAPLES | 1 | 0.066 |
| 1684 | FEDERICO II UNIV NAPLES | 1 | 0.066 |
| 1685 | FEINSTEIN INST MED RES | 1 | 0.066 |
| 1686 | FERRIS STATE UNIV | 1 | 0.066 |
| 1687 | FIRST COAST CARDIOVASC INST | 1 | 0.066 |
| 1688 | FLENI FDN | 1 | 0.066 |
| 1689 | FLINDERS UNIV S AUSTRALIA | 1 | 0.066 |
| 1690 | FLORIDA HOSP | 1 | 0.066 |
| 1691 | FLORIDA HOSP TRANSLAT RES INST METAB DIABET | 1 | 0.066 |
| 1692 | FLORIDA INT UNIV | 1 | 0.066 |
| 1693 | FLORIDA POISON INFORMAT CTR | 1 | 0.066 |
| 1694 | FMTS | 1 | 0.066 |
| 1695 | FOLKHALSAN RES CTR | 1 | 0.066 |
| 1696 | FONDAT BORDEAUX UNIV | 1 | 0.066 |
| 1697 | FORTIS HOSP | 1 | 0.066 |
| 1698 | FOSCAL | 1 | 0.066 |
| 1699 | FOURTH MIL MED UNIV | 1 | 0.066 |
| 1700 | FRANCISCUS GASTHUIS | 1 | 0.066 |
| 1701 | FREE UNIV BERLIN | 1 | 0.066 |
| 1702 | FREEMAN MED CTR | 1 | 0.066 |
| 1703 | FREIBURG UNIV HOSP | 1 | 0.066 |
| 1704 | FREMANTLE HOSP | 1 | 0.066 |
| 1705 | FRENCH CLIN RES INFRASTRUCT NETWORK | 1 | 0.066 |
| 1706 | FRIEDRICH ALEXANDER UNIV ERLANGEN | 1 | 0.066 |
| 1707 | FRIEDRICH ALEXANDER UNIV ERLANGEN NURNBERG FAU | 1 | 0.066 |
| 1708 | FU JEN CATHOLIC UNIV | 1 | 0.066 |
| 1709 | FUDAN UNIV ZHONGSHAN HOSP | 1 | 0.066 |
| 1710 | FUNDACIO INST UNIV RECERCA ATENCIO PRIMARIA SALUT | 1 | 0.066 |
| 1711 | FUTATA TETSUHIRO CLIN | 1 | 0.066 |
| 1712 | FV HOSP | 1 | 0.066 |
| 1713 | G PAPANIKOLAOU GEN HOSP | 1 | 0.066 |
| 1714 | G SALICETO HOSP | 1 | 0.066 |
| 1715 | GACHON CARDIOVASC RES INST | 1 | 0.066 |
| 1716 | GACHON UNIV | 1 | 0.066 |
| 1717 | GALAXY SPECIAL CTR | 1 | 0.066 |
| 1718 | GALILEE MED CTR | 1 | 0.066 |
| 1719 | GEELONG HOSP | 1 | 0.066 |
| 1720 | GEENDIAB | 1 | 0.066 |
| 1721 | GEISEL SCH MED DARTMOUTH | 1 | 0.066 |
| 1722 | GEISINGER CMC HOSP | 1 | 0.066 |
| 1723 | GEISINGER MED CTR | 1 | 0.066 |
| 1724 | GEN FAC HOSP | 1 | 0.066 |
| 1725 | GEN HOSP CHINESE PEOPLES ARMED POLICE FORCES | 1 | 0.066 |
| 1726 | GENEVA UNIV HOSP | 1 | 0.066 |
| 1727 | GENTOFTE UNIV | 1 | 0.066 |
| 1728 | GEORG AUGUST UNIV GOETTINGEN | 1 | 0.066 |
| 1729 | GEORG AUGUST UNIV GOTTINGEN | 1 | 0.066 |
| 1730 | GEORGE INST | 1 | 0.066 |
| 1731 | GEORGETOWN UNIV HOSP | 1 | 0.066 |
| 1732 | GERMAN AEROSP CTR DLR | 1 | 0.066 |
| 1733 | GERMAN CTR CARDIOVASCULAR RES DZHK | 1 | 0.066 |
| 1734 | GERMAN CTR DIABET RES | 1 | 0.066 |
| 1735 | GETWELL HOSP RES INST | 1 | 0.066 |
| 1736 | GHM PORTES SUD | 1 | 0.066 |
| 1737 | GIP CYCERON | 1 | 0.066 |
| 1738 | GLENFIELD HOSP | 1 | 0.066 |
| 1739 | GLENMARK PHARMACEUT LTD | 1 | 0.066 |
| 1740 | GLOBAL MARKET ACCESS PRICING | 1 | 0.066 |
| 1741 | GLOBAL MED DEV ASTRAZENECA | 1 | 0.066 |
| 1742 | GOLDA MEIR HOSP | 1 | 0.066 |
| 1743 | GREEN CLIN | 1 | 0.066 |
| 1744 | GRENOBLE ALPS UNIV | 1 | 0.066 |
| 1745 | GRIFFIN HOSP | 1 | 0.066 |
| 1746 | GRIGORE T POPA UNIV MED | 1 | 0.066 |
| 1747 | GROCHOWSKI HOSP | 1 | 0.066 |
| 1748 | GUANGDONG PHARMACEUT UNIV | 1 | 0.066 |
| 1749 | GUANGDONG PROV PHARMACEUT ASSOC | 1 | 0.066 |
| 1750 | GUANGXI MED UNIV | 1 | 0.066 |
| 1751 | GUANGZHOU GEN PHARMACEUT RES INST CO LTD | 1 | 0.066 |
| 1752 | GUANGZHOU HUI AI HOSP | 1 | 0.066 |
| 1753 | GUYS ST THOMAS NHS FDN TRUST | 1 | 0.066 |
| 1754 | HACETTEPE UNIV | 1 | 0.066 |
| 1755 | HADASSAH MED CTR | 1 | 0.066 |
| 1756 | HAMAD GEN HOSP | 1 | 0.066 |
| 1757 | HAMASAKI CLIN | 1 | 0.066 |
| 1758 | HANUSCH HOSP | 1 | 0.066 |
| 1759 | HANWA MEM HOSP | 1 | 0.066 |
| 1760 | HANYANG UNIV | 1 | 0.066 |
| 1761 | HARBOR UCLA MED CTR | 1 | 0.066 |
| 1762 | HARRY PERKINS INST MED RES | 1 | 0.066 |
| 1763 | HARTFORD HOSP | 1 | 0.066 |
| 1764 | HARVARD CLIN RES INST | 1 | 0.066 |
| 1765 | HARVARD SCH DENT MED | 1 | 0.066 |
| 1766 | HARVARD SCH PUBL HLTH | 1 | 0.066 |
| 1767 | HAYASHI CLIN | 1 | 0.066 |
| 1768 | HEALTHCARE HOMELESS | 1 | 0.066 |
| 1769 | HEALTHCARE INST COMMUNITY PHARM SLAVONSKI BROD | 1 | 0.066 |
| 1770 | HEALTHCORE INC | 1 | 0.066 |
| 1771 | HEART GRP EASTERN SHORE | 1 | 0.066 |
| 1772 | HEDON GRP PRACTICE | 1 | 0.066 |
| 1773 | HELEN SCHNEIDER WOMENS HOSP | 1 | 0.066 |
| 1774 | HELLEN NATL CTR RES PREVENT TREATMENT DIABET ME | 1 | 0.066 |
| 1775 | HELMHOLTZ DIABET CTR | 1 | 0.066 |
| 1776 | HENAN UNIV SCI TECHNOL | 1 | 0.066 |
| 1777 | HEOR LTD | 1 | 0.066 |
| 1778 | HERLEV HOSP | 1 | 0.066 |
| 1779 | HIGASHIAGATSUMA NATL HLTH INSURANCE CLIN | 1 | 0.066 |
| 1780 | HIGH SPECIALTY REG HOSP IXTAPALUCA | 1 | 0.066 |
| 1781 | HINATA CLIN | 1 | 0.066 |
| 1782 | HIPPOKRATEION HOSP | 1 | 0.066 |
| 1783 | HLTH ECON OUTCOMES RES ASTRAZENECA | 1 | 0.066 |
| 1784 | HLTH RESEARCHTX LLC | 1 | 0.066 |
| 1785 | HLTH SCI RES INST | 1 | 0.066 |
| 1786 | HLTH SCI UNIV | 1 | 0.066 |
| 1787 | HLTH SCI UNIV HOKKAIDO | 1 | 0.066 |
| 1788 | HOAG MEM HOSP | 1 | 0.066 |
| 1789 | HOFSTRA NORTHWELL SCH MED | 1 | 0.066 |
| 1790 | HOKUSHO UNIV | 1 | 0.066 |
| 1791 | HOLBAEK CENT HOSP | 1 | 0.066 |
| 1792 | HOMMA INTERNAL MED CARDIOVASC CLIN | 1 | 0.066 |
| 1793 | HOP EUROPEEN GEORGES POMPIDOU | 1 | 0.066 |
| 1794 | HOP RAYMOND POINCARE | 1 | 0.066 |
| 1795 | HOP ST JOSEPH | 1 | 0.066 |
| 1796 | HOP UNIV STRASBOURG | 1 | 0.066 |
| 1797 | HOP XAVIER BICHAT | 1 | 0.066 |
| 1798 | HOPKINS CONSULTING LLC | 1 | 0.066 |
| 1799 | HORIZON BLUE CROSS BLUE SHIELD NEW JERSEY | 1 | 0.066 |
| 1800 | HORMOZGAN UNIV MED SCI | 1 | 0.066 |
| 1801 | HORUS UNIV | 1 | 0.066 |
| 1802 | HORUS UNIV EGYPT | 1 | 0.066 |
| 1803 | HOSP ALBERT EINSTEIN | 1 | 0.066 |
| 1804 | HOSP ALEMAO OSWALDO CRUZ | 1 | 0.066 |
| 1805 | HOSP BARMHERZIGE BRUDER LINZ | 1 | 0.066 |
| 1806 | HOSP CLIN PORTO ALEGRE | 1 | 0.066 |
| 1807 | HOSP CLIN UNIV | 1 | 0.066 |
| 1808 | HOSP CUF PORTO | 1 | 0.066 |
| 1809 | HOSP EISENSTADT | 1 | 0.066 |
| 1810 | HOSP ESPECIALIDADES CTR MED LA RAZA | 1 | 0.066 |
| 1811 | HOSP FORCAS ARMADAS POLO PORTO | 1 | 0.066 |
| 1812 | HOSP GEN UNIV CIUDAD REAL | 1 | 0.066 |
| 1813 | HOSP GEN UNIV GREGORIO MARANON | 1 | 0.066 |
| 1814 | HOSP GEN UNIV VALENCIA | 1 | 0.066 |
| 1815 | HOSP GRAZ II SITE WEST | 1 | 0.066 |
| 1816 | HOSP GRP TWENTE | 1 | 0.066 |
| 1817 | HOSP LA CORUNA | 1 | 0.066 |
| 1818 | HOSP LA PAZ | 1 | 0.066 |
| 1819 | HOSP MARCIDE | 1 | 0.066 |
| 1820 | HOSP PORTUGUES | 1 | 0.066 |
| 1821 | HOSP RIM | 1 | 0.066 |
| 1822 | HOSP SANTA CREU SANT PAU | 1 | 0.066 |
| 1823 | HOSP SANTA CRUZ | 1 | 0.066 |
| 1824 | HOSP SPECIAL SURG | 1 | 0.066 |
| 1825 | HOSP TROP DIS | 1 | 0.066 |
| 1826 | HOSP UNIV 12 OCTUBRE RES INST I 12 | 1 | 0.066 |
| 1827 | HOSP UNIV CENT ASTURIAS | 1 | 0.066 |
| 1828 | HOSP UNIV DOCTOR PESET | 1 | 0.066 |
| 1829 | HOSP UNIV FDN ALCORCON | 1 | 0.066 |
| 1830 | HOSP UNIV NS CANDELARIA | 1 | 0.066 |
| 1831 | HOSP UNIV NUESTRA SENORA CANDELARIA | 1 | 0.066 |
| 1832 | HOSP UNIV NUESTRA SENORA DE CANDELARIA | 1 | 0.066 |
| 1833 | HOSP UNIV PUERTA DEL MAR | 1 | 0.066 |
| 1834 | HOSP UNIV PUERTA HIERRO MAJADAHONDA | 1 | 0.066 |
| 1835 | HOSP UNIV REINA SOFIA | 1 | 0.066 |
| 1836 | HOSP UNIV RIBERA | 1 | 0.066 |
| 1837 | HOSP UNIV SALAMANCA | 1 | 0.066 |
| 1838 | HOSP UNIV SAN JUAN | 1 | 0.066 |
| 1839 | HOSP UNIV SANTIAGO COMPOSTELA | 1 | 0.066 |
| 1840 | HOSP UNIV SANTIAGO DE COMPOSTELA | 1 | 0.066 |
| 1841 | HOSP UNIV VALL DHEBRON | 1 | 0.066 |
| 1842 | HOSP UNIV VALL HEBRON | 1 | 0.066 |
| 1843 | HOSP UNIV VIRGEN DE LA VICTORIA | 1 | 0.066 |
| 1844 | HOSP UNIV VIRGEN MACARENA | 1 | 0.066 |
| 1845 | HOSP VIRGEN MACARENA | 1 | 0.066 |
| 1846 | HOSP WUERZBURG | 1 | 0.066 |
| 1847 | HOSPICES CIVILS LYON | 1 | 0.066 |
| 1848 | HOTARUNO CENT NAIKA | 1 | 0.066 |
| 1849 | HOUJU MEM HOSP | 1 | 0.066 |
| 1850 | HR PATEL INST PHARMACEUT EDUC RES | 1 | 0.066 |
| 1851 | HUAZHONG UNIV SCI TECHNOL | 1 | 0.066 |
| 1852 | HUMANITAS UNIV | 1 | 0.066 |
| 1853 | HUNTER NEW ENGLAND LOCAL HLTH DIST | 1 | 0.066 |
| 1854 | HYOGO COLL MED | 1 | 0.066 |
| 1855 | IATRIKO PALEOU FALIROU MED CTR | 1 | 0.066 |
| 1856 | IBARAKI SEINAN MED CTR HOSP | 1 | 0.066 |
| 1857 | ICAHN SCH MED MT SINAI HOSP | 1 | 0.066 |
| 1858 | ICMR NATL ANIM RESOURCE FACIL BIOMED RES NARFBR | 1 | 0.066 |
| 1859 | ICMR NATL INST EPIDEMIOL | 1 | 0.066 |
| 1860 | ICMR NATL INST VIROL | 1 | 0.066 |
| 1861 | IDAHO STATE UNIV | 1 | 0.066 |
| 1862 | IKEDA MUNICIPAL HOSP | 1 | 0.066 |
| 1863 | ILLINOIS POISON CTR | 1 | 0.066 |
| 1864 | IMPERIAL COLL HEALTHCARE NHS FDN TRUST | 1 | 0.066 |
| 1865 | INADA MED CLIN | 1 | 0.066 |
| 1866 | INAMDAR MULTISPECIALTY HOSP | 1 | 0.066 |
| 1867 | INCHEON MED CTR | 1 | 0.066 |
| 1868 | INFRAFRONTIER GMBH | 1 | 0.066 |
| 1869 | INHA UNIV | 1 | 0.066 |
| 1870 | INNERE MED ALLGEMEINMED LEVERKUSEN | 1 | 0.066 |
| 1871 | INNLANDET HOSP TRUST | 1 | 0.066 |
| 1872 | INOKUCHI CLIN | 1 | 0.066 |
| 1873 | INSERM CRI 866 | 1 | 0.066 |
| 1874 | INSERM U1018 | 1 | 0.066 |
| 1875 | INSERM U657 | 1 | 0.066 |
| 1876 | INST BIOMED RES | 1 | 0.066 |
| 1877 | INST CARDIOL | 1 | 0.066 |
| 1878 | INST CARLOS IIIFEDER | 1 | 0.066 |
| 1879 | INST CATALA SALUT | 1 | 0.066 |
| 1880 | INST CLIN CHEM PATHOBIOCHEM | 1 | 0.066 |
| 1881 | INST CLIN PHARMACOL TOXICOL | 1 | 0.066 |
| 1882 | INST DESARROLLO INTEGRAT SALUD | 1 | 0.066 |
| 1883 | INST DIABET MELLITUS | 1 | 0.066 |
| 1884 | INST ECUATORIANO CORAZON IECOR | 1 | 0.066 |
| 1885 | INST HLTH CARLOS III | 1 | 0.066 |
| 1886 | INST HLTH RES INCLIVA | 1 | 0.066 |
| 1887 | INST INVEST BIOMED AUGUST PI SUNYER IDIBAPS | 1 | 0.066 |
| 1888 | INST INVEST BIOMED MALAGA IBIMA | 1 | 0.066 |
| 1889 | INST INVEST INNOVAC CIENCIAS BIOMED PROV CADIZ | 1 | 0.066 |
| 1890 | INST INVEST SANITARIA PRINCIPADO ASTURIAS ISPA | 1 | 0.066 |
| 1891 | INST INVEST SANITARIAS SANTIAGO COMPOSTELA IDIS | 1 | 0.066 |
| 1892 | INST LORRAIN COEUR VAISSEAUX | 1 | 0.066 |
| 1893 | INST MED RES OCCUPAT HLTH | 1 | 0.066 |
| 1894 | INST MEXICANO SEGURO SOCIAL | 1 | 0.066 |
| 1895 | INST NACL CARDIOL DR IGNACIO CHAVEZ RIERA | 1 | 0.066 |
| 1896 | INST PHARMACOL PREVENT MED | 1 | 0.066 |
| 1897 | INST RECERCA BIOMED LLEIDA | 1 | 0.066 |
| 1898 | INST SCI TECHNOL MED | 1 | 0.066 |
| 1899 | INST TRANSLAT HLTH SCI | 1 | 0.066 |
| 1900 | INT MED UNIV | 1 | 0.066 |
| 1901 | INTERIOR HLTH AUTHOR DIABET STRATEGY | 1 | 0.066 |
| 1902 | INVENT HLTH CLIN | 1 | 0.066 |
| 1903 | IQVIA REAL WORLD EVIDENCE SOLUT | 1 | 0.066 |
| 1904 | IRBLLEIDA | 1 | 0.066 |
| 1905 | IRCCS MULTIMED SESTO SAN GIOVANNI | 1 | 0.066 |
| 1906 | IRCCS NEUROMED | 1 | 0.066 |
| 1907 | ISALA | 1 | 0.066 |
| 1908 | ISAR KLINIKEN GMBH MUNICH | 1 | 0.066 |
| 1909 | ISCIII | 1 | 0.066 |
| 1910 | ISRAEL MINIST HLTH | 1 | 0.066 |
| 1911 | ISTANBUL UNIV | 1 | 0.066 |
| 1912 | ITABASHI DIABET MED DERMATOL CLIN | 1 | 0.066 |
| 1913 | ITALIAN ASSOC HOSP CARDIOLOGISTS | 1 | 0.066 |
| 1914 | IULIU HATIEGANU UNIV MED PHARM | 1 | 0.066 |
| 1915 | IVAN FRANKO NATL UNIV LVIV | 1 | 0.066 |
| 1916 | IWAKUNI CLIN CTR | 1 | 0.066 |
| 1917 | IWATE MED UNIV | 1 | 0.066 |
| 1918 | JA HIROSHIMA GEN HOSP | 1 | 0.066 |
| 1919 | JACKSON LAB | 1 | 0.066 |
| 1920 | JAGIELLONIAN UNIV | 1 | 0.066 |
| 1921 | JAPAN AGCY MED RES DEV | 1 | 0.066 |
| 1922 | JAPAN AGCY MEDI CAL RES DEV | 1 | 0.066 |
| 1923 | JAPAN COMMUNITY HLTH CARE ORG TOKYO KAMATA MED CT | 1 | 0.066 |
| 1924 | JAPAN LABOUR HLTH WELF ORG | 1 | 0.066 |
| 1925 | JAPAN SELF DEF FORCES CENT HOSP | 1 | 0.066 |
| 1926 | JAPAN SOC PROMOT SCI | 1 | 0.066 |
| 1927 | JAPANESE RED CROSS MED CTR | 1 | 0.066 |
| 1928 | JAPANESE RED CROSS NAGOYA DAINI HOSP | 1 | 0.066 |
| 1929 | JAPANESE RED CROSS NARITA HOSP | 1 | 0.066 |
| 1930 | JAZAN UNIV | 1 | 0.066 |
| 1931 | JDRF | 1 | 0.066 |
| 1932 | JEFFERSON COLL PHARM | 1 | 0.066 |
| 1933 | JEFFERSON UNIV HOSPITAL | 1 | 0.066 |
| 1934 | JEROEN BOSCH ZIEKENHUIS | 1 | 0.066 |
| 1935 | JERSEY SHORE UNIV | 1 | 0.066 |
| 1936 | JIANNREN HOSP | 1 | 0.066 |
| 1937 | JILIN UNIV | 1 | 0.066 |
| 1938 | JIYUGAOKA MED CLIN | 1 | 0.066 |
| 1939 | JOHN H STROGER JR HOSP | 1 | 0.066 |
| 1940 | JOHN HUNTER HOSP | 1 | 0.066 |
| 1941 | JOHNS HOPKINS CICCARONE CTR PREVENT CARDIOVASC DI | 1 | 0.066 |
| 1942 | JOHNS HOPKINS CICCARONE CTR PREVENT HEART DIS | 1 | 0.066 |
| 1943 | JOHNS HOPKINS OUTPATIENT CTR | 1 | 0.066 |
| 1944 | JR SAPPORO HOSP | 1 | 0.066 |
| 1945 | KAGOSHIMA UNIV | 1 | 0.066 |
| 1946 | KAN ETSU CHUOH HOSP | 1 | 0.066 |
| 1947 | KANAGAWA DENT UNIV | 1 | 0.066 |
| 1948 | KANAGAWA UNIV HUMAN SERV | 1 | 0.066 |
| 1949 | KANDA NAIKA CLIN | 1 | 0.066 |
| 1950 | KANTAR HLTH GMBH | 1 | 0.066 |
| 1951 | KAO CORP | 1 | 0.066 |
| 1952 | KAOHSIUNG CHANG GUNG MEM HOSP | 1 | 0.066 |
| 1953 | KARDINAL SCHWARZENBERGSCHES KRANKENHAUS SCHWARZAC | 1 | 0.066 |
| 1954 | KAROLINSKA INST KAROLINSKA UNIV HOSP | 1 | 0.066 |
| 1955 | KAROLINSKA UNIV | 1 | 0.066 |
| 1956 | KAROLINSKA UNIV HOSP SOLNA | 1 | 0.066 |
| 1957 | KASHIWA CITY HOSP | 1 | 0.066 |
| 1958 | KAT GEN HOSP | 1 | 0.066 |
| 1959 | KATO CLIN INTERNAL MED | 1 | 0.066 |
| 1960 | KATSUYA CLIN | 1 | 0.066 |
| 1961 | KBR CLIN PHARMACOL SERV | 1 | 0.066 |
| 1962 | KEELE UNIV | 1 | 0.066 |
| 1963 | KEENAN RES CTR BIOMED SCI | 1 | 0.066 |
| 1964 | KEPLERUNIKLINIKUM LINZ | 1 | 0.066 |
| 1965 | KFH KIDNEY CTR | 1 | 0.066 |
| 1966 | KFH NIERENZENTRUM MUNCHEN SCHWABING | 1 | 0.066 |
| 1967 | KG JEBSEN CTR CARDIAC RES | 1 | 0.066 |
| 1968 | KHON KAEN UNIV | 1 | 0.066 |
| 1969 | KIKUMA CLIN | 1 | 0.066 |
| 1970 | KIMITSU CHUO HOSP | 1 | 0.066 |
| 1971 | KINDAI UNIV | 1 | 0.066 |
| 1972 | KINDERKRANKENHAUS BULT | 1 | 0.066 |
| 1973 | KINDRED HOSP | 1 | 0.066 |
| 1974 | KING SAUD BIN ABDULAZIZ UNIV HLTH SCI | 1 | 0.066 |
| 1975 | KING SAUD UNIV LI KA SHING COLLABORAT RES PROGRAM | 1 | 0.066 |
| 1976 | KINGSTON GEN HOSP | 1 | 0.066 |
| 1977 | KISSEI PHARMACEUT CO | 1 | 0.066 |
| 1978 | KITASENRI MAEDA CLIN | 1 | 0.066 |
| 1979 | KLINIKUM FRANKFURT HOCHST | 1 | 0.066 |
| 1980 | KLINIKUM KLAGENFURT | 1 | 0.066 |
| 1981 | KLINIKUM LUDWIGSHAFEN | 1 | 0.066 |
| 1982 | KOCAELI UNIV | 1 | 0.066 |
| 1983 | KONSTANTOPOULEIO HOSP | 1 | 0.066 |
| 1984 | KONYA TRAINING RES HOSP | 1 | 0.066 |
| 1985 | KOREA ADV INST SCI TECHNOL | 1 | 0.066 |
| 1986 | KOSUGI MED CLIN | 1 | 0.066 |
| 1987 | KOVAI DIABET SPECIAL CTR HOSP | 1 | 0.066 |
| 1988 | KOWA PHARMACEUT CO LTD | 1 | 0.066 |
| 1989 | KS RANGASAMY COLL ARTS SCI AUTONOMOUS | 1 | 0.066 |
| 1990 | KUMAMOTO UNIV HOSP | 1 | 0.066 |
| 1991 | KUMASHIRO HOSP | 1 | 0.066 |
| 1992 | KUNSHAN REHABIL HOSP | 1 | 0.066 |
| 1993 | KURATORIUM DIALYSE KIDNEY CTR | 1 | 0.066 |
| 1994 | KURUME CHUO HOSP | 1 | 0.066 |
| 1995 | KURUME INTERNAL MED ASSOC | 1 | 0.066 |
| 1996 | KYOTO PREFECTURAL UNIV MED | 1 | 0.066 |
| 1997 | KYUNGPOOK NATL UNIV | 1 | 0.066 |
| 1998 | L MARC | 1 | 0.066 |
| 1999 | L VANVITELLI UNIV | 1 | 0.066 |
| 2000 | LA FE UNIV HOSP | 1 | 0.066 |
| 2001 | LA TROBE UNIV | 1 | 0.066 |
| 2002 | LADY DAVIS INST | 1 | 0.066 |
| 2003 | LADY HARDINGE MED COLL HOSP | 1 | 0.066 |
| 2004 | LAIKON GEN HOSP | 1 | 0.066 |
| 2005 | LARISSA UNIV | 1 | 0.066 |
| 2006 | LATVIAN INST ORGAN SYNTH | 1 | 0.066 |
| 2007 | LAWSON HLTH RES INST | 1 | 0.066 |
| 2008 | LEADERSHIP SINAI CTR DIABET | 1 | 0.066 |
| 2009 | LEBANESE UNIV | 1 | 0.066 |
| 2010 | LEEDS TEACHING HOSP NHS TRUST | 1 | 0.066 |
| 2011 | LEIBNIZ CTR DIABET RES | 1 | 0.066 |
| 2012 | LEIGHTON HOSP | 1 | 0.066 |
| 2013 | LI KA SHING INST HLTH SCI | 1 | 0.066 |
| 2014 | LIMOGES UNIV | 1 | 0.066 |
| 2015 | LINA DIABET CTR | 1 | 0.066 |
| 2016 | LIONS EYE INST | 1 | 0.066 |
| 2017 | LIVERPOOL HOSP | 1 | 0.066 |
| 2018 | LMC | 1 | 0.066 |
| 2019 | LOCAT VU UNIV | 1 | 0.066 |
| 2020 | LONDON SCH ECON | 1 | 0.066 |
| 2021 | LOS ANGELES BIOMED RES INST | 1 | 0.066 |
| 2022 | LOUGHBOROUGH UNIV | 1 | 0.066 |
| 2023 | LOUISIANA POISON CTR | 1 | 0.066 |
| 2024 | LOUISIANA PUBL HLTH INST | 1 | 0.066 |
| 2025 | LOUISIANA STATE UNIV SYST | 1 | 0.066 |
| 2026 | LOUISVILLE METAB ATHEROSCLEROSIS RES CTR | 1 | 0.066 |
| 2027 | LOUISVILLE METAB ATHEROSCLEROSIS RES CTR INC | 1 | 0.066 |
| 2028 | LOVELY PROFESS UNIV | 1 | 0.066 |
| 2029 | LUND UNIV | 1 | 0.066 |
| 2030 | LUNENFELD TANENBAUM RES INST | 1 | 0.066 |
| 2031 | LUZHOU MED COLL | 1 | 0.066 |
| 2032 | M S DECIS | 1 | 0.066 |
| 2033 | MACAU UNIV SCI | 1 | 0.066 |
| 2034 | MACKAY MED COLL | 1 | 0.066 |
| 2035 | MAE FAH LUANG UNIV | 1 | 0.066 |
| 2036 | MAGDALENA CLIN CARDIOVASC DIS | 1 | 0.066 |
| 2037 | MANCHESTER UNIV NHS FDN TRUST | 1 | 0.066 |
| 2038 | MANDA MEM HOSP | 1 | 0.066 |
| 2039 | MANHES HOSP | 1 | 0.066 |
| 2040 | MANSOURA FAC MED | 1 | 0.066 |
| 2041 | MANSOURA UNIV | 1 | 0.066 |
| 2042 | MARATHA MANDAL RES CTR | 1 | 0.066 |
| 2043 | MARIENHOSPITAL | 1 | 0.066 |
| 2044 | MARMARA UNIV | 1 | 0.066 |
| 2045 | MASARYK UNIV HOSP | 1 | 0.066 |
| 2046 | MASSACHUSETTS COLL PHARM ALLIED HLTH SCI | 1 | 0.066 |
| 2047 | MASSACHUSETTS COLL PHARM HLTH SCI | 1 | 0.066 |
| 2048 | MASSACHUSETTS COLL PHARM HLTH SCI UNIV | 1 | 0.066 |
| 2049 | MASTER SCI PA PROGRAM | 1 | 0.066 |
| 2050 | MAX SUPER SPECIALTY HOSP | 1 | 0.066 |
| 2051 | MAYO CLIN FLORIDA | 1 | 0.066 |
| 2052 | MAZANKOWSKI ALBERTA HEART INST | 1 | 0.066 |
| 2053 | MCMDC | 1 | 0.066 |
| 2054 | MED CENT LABS | 1 | 0.066 |
| 2055 | MED COLL WISCONSIN | 1 | 0.066 |
| 2056 | MED CORP | 1 | 0.066 |
| 2057 | MED CORP KYOUSOUKAI | 1 | 0.066 |
| 2058 | MED CORPS | 1 | 0.066 |
| 2059 | MED CTR VARNA | 1 | 0.066 |
| 2060 | MED HLTH SCI UNIV | 1 | 0.066 |
| 2061 | MED INFORMAT METROHLTH HEART VASC | 1 | 0.066 |
| 2062 | MED PK HOSP | 1 | 0.066 |
| 2063 | MED SCH | 1 | 0.066 |
| 2064 | MED UNIV LUBLIN | 1 | 0.066 |
| 2065 | MED UNIV WARSAW | 1 | 0.066 |
| 2066 | MEDANTA THE MEDICITY | 1 | 0.066 |
| 2067 | MEDEMERGE FAMILY PRACTICE CTR | 1 | 0.066 |
| 2068 | MEDICITY | 1 | 0.066 |
| 2069 | MEDICUS HLTH PARTNERS | 1 | 0.066 |
| 2070 | MEDIMMUNE | 1 | 0.066 |
| 2071 | MEDITERRANEA CARDIOCENTRO | 1 | 0.066 |
| 2072 | MEDIWELL MED CLIN | 1 | 0.066 |
| 2073 | MEDSTAR HLTH | 1 | 0.066 |
| 2074 | MEDSTAR UNION MEM HOSP | 1 | 0.066 |
| 2075 | MEDSTAR WASHINGTON HOSP CTR | 1 | 0.066 |
| 2076 | MEM HEALTHCARE SYST | 1 | 0.066 |
| 2077 | MEM UNIV | 1 | 0.066 |
| 2078 | MEMPHIS VET AFFAIRS MED CTR | 1 | 0.066 |
| 2079 | MENDED HEARTS | 1 | 0.066 |
| 2080 | MENIA UNIV | 1 | 0.066 |
| 2081 | MENZIES SCH HLTH RES | 1 | 0.066 |
| 2082 | MERCK KGAA | 1 | 0.066 |
| 2083 | MERCK SHARP DOHME CORP | 1 | 0.066 |
| 2084 | MERCK SHARP DOHME LTD | 1 | 0.066 |
| 2085 | MERCY HLTH OSTEOPOROSIS BONE HLTH SERV | 1 | 0.066 |
| 2086 | MERKUR UNIV HOSP | 1 | 0.066 |
| 2087 | MET TEST | 1 | 0.066 |
| 2088 | METAXA GEN HOSP | 1 | 0.066 |
| 2089 | METHODIST UNIV HOSP | 1 | 0.066 |
| 2090 | METROHEALTH | 1 | 0.066 |
| 2091 | METROHLTH MED CTR | 1 | 0.066 |
| 2092 | MEXICAN SOCIAL SECUR INST | 1 | 0.066 |
| 2093 | MI SECHENOV FIRST MOSCOW STATE MED UNIV | 1 | 0.066 |
| 2094 | MIAMI VA HEALTHCARE SYST | 1 | 0.066 |
| 2095 | MIDAMER DIABET ASSOCIATES | 1 | 0.066 |
| 2096 | MIHO CLIN | 1 | 0.066 |
| 2097 | MIL HOSP | 1 | 0.066 |
| 2098 | MILANO BICOCCA UNIV | 1 | 0.066 |
| 2099 | MILLIMAN INC | 1 | 0.066 |
| 2100 | MINAMISANRIKU HOSP | 1 | 0.066 |
| 2101 | MINIST HLTH | 1 | 0.066 |
| 2102 | MINIST INTERIOR | 1 | 0.066 |
| 2103 | MINNEAPOLIS HEART INST | 1 | 0.066 |
| 2104 | MINNEAPOLIS HEART INST FDN | 1 | 0.066 |
| 2105 | MINNEAPOLIS VA CTR CARE DELIVERY OUTCOMES RES | 1 | 0.066 |
| 2106 | MINNEAPOLIS VA HLTH CARE SYST | 1 | 0.066 |
| 2107 | MISAKI NAIKA CLIN | 1 | 0.066 |
| 2108 | MISR SCI TECHNOL UNIV | 1 | 0.066 |
| 2109 | MITOCHON PHARMACEUT INC | 1 | 0.066 |
| 2110 | MITOYO GEN HOSP | 1 | 0.066 |
| 2111 | MIURA CENT CLIN | 1 | 0.066 |
| 2112 | MONASH HLTH | 1 | 0.066 |
| 2113 | MONASH UNIV CENT | 1 | 0.066 |
| 2114 | MRC | 1 | 0.066 |
| 2115 | MS RAMAIAH MEM HOSP | 1 | 0.066 |
| 2116 | MSD LTD | 1 | 0.066 |
| 2117 | MT AUBURN HOSP | 1 | 0.066 |
| 2118 | MT SINAI CLIN DIABET INST | 1 | 0.066 |
| 2119 | MT SINAI ST LUKES ROOSEVELT | 1 | 0.066 |
| 2120 | MT SINAI ST LUKES ROOSEVELT HOSP | 1 | 0.066 |
| 2121 | MUHIMBILI UNIV HLTH ALLIED SCI | 1 | 0.066 |
| 2122 | MUKAE INTERNAL MED CLIN | 1 | 0.066 |
| 2123 | MULTIMED IRCCS | 1 | 0.066 |
| 2124 | MUNICH HELMHOLTZ CTR | 1 | 0.066 |
| 2125 | MUSASHINO TOKUSHUKAI HOSP | 1 | 0.066 |
| 2126 | MUSTAFA KEMAL UNIV | 1 | 0.066 |
| 2127 | N PAULESCU NATL INST DIABET NUTR METAB DIS | 1 | 0.066 |
| 2128 | N ZERO 1 LTD | 1 | 0.066 |
| 2129 | NAGANO COLL NURSING | 1 | 0.066 |
| 2130 | NAGASAKI UNIV HOSP | 1 | 0.066 |
| 2131 | NAKAKINEN CLIN | 1 | 0.066 |
| 2132 | NANJING UNIV | 1 | 0.066 |
| 2133 | NANYANG TECHNOL UNIV | 1 | 0.066 |
| 2134 | NARA MED UNIV | 1 | 0.066 |
| 2135 | NARA MED UNIV HOSP | 1 | 0.066 |
| 2136 | NASU MINAMI HOSP | 1 | 0.066 |
| 2137 | NATL BUR ECON RES | 1 | 0.066 |
| 2138 | NATL CTR GLOBAL HLTH MED | 1 | 0.066 |
| 2139 | NATL DEF MED COLL | 1 | 0.066 |
| 2140 | NATL DIABET AUDIT | 1 | 0.066 |
| 2141 | NATL HEART FDN AUSTRALIA | 1 | 0.066 |
| 2142 | NATL HOSP ORG CHIBA MED CTR | 1 | 0.066 |
| 2143 | NATL HOSP ORG KYOTO MED CTR | 1 | 0.066 |
| 2144 | NATL INST ENDOCRINOL DIABETOL | 1 | 0.066 |
| 2145 | NATL INST HLTH WELF | 1 | 0.066 |
| 2146 | NATL INST MED SCI NUTR SALVADOR ZUBIRAN | 1 | 0.066 |
| 2147 | NATL INST NUTR ICMR | 1 | 0.066 |
| 2148 | NATL LIB MED | 1 | 0.066 |
| 2149 | NATL MED RES CTR CARDIOL RUSSIA | 1 | 0.066 |
| 2150 | NATL MED SCI NUTR INST SALVADOR ZUBIRAN | 1 | 0.066 |
| 2151 | NATL RES COUNCIL CNR | 1 | 0.066 |
| 2152 | NATL RES COUNCIL IFC CNR | 1 | 0.066 |
| 2153 | NATL SUN YAT SEN UNIV | 1 | 0.066 |
| 2154 | NATL UNIV HLTH SYST | 1 | 0.066 |
| 2155 | NATL UNIV IRELAND GALWAY | 1 | 0.066 |
| 2156 | NAVAL MED CTR | 1 | 0.066 |
| 2157 | NEPHROPATHY CLIN MED RES CTR SICHUAN PROV | 1 | 0.066 |
| 2158 | NEWCASTLE UPON TYNE HOSP NHS FDN TRUST | 1 | 0.066 |
| 2159 | NEWYORK UNIV LANGONE HLTH | 1 | 0.066 |
| 2160 | NHLBI | 1 | 0.066 |
| 2161 | NIAID | 1 | 0.066 |
| 2162 | NICHHD | 1 | 0.066 |
| 2163 | NIDCR | 1 | 0.066 |
| 2164 | NIEHS | 1 | 0.066 |
| 2165 | NIH | 1 | 0.066 |
| 2166 | NIHON UNIV HOSP | 1 | 0.066 |
| 2167 | NIHR APPL RES COLLABORAT EAST MIDLANDS | 1 | 0.066 |
| 2168 | NIHR COLLABORAT LEADERSHIP APPL HLTH RES CARE C | 1 | 0.066 |
| 2169 | NIHR EXETER CLIN RES FACIL | 1 | 0.066 |
| 2170 | NIHR LEICESTER BIOMED RES CTR | 1 | 0.066 |
| 2171 | NINGBO UNIV | 1 | 0.066 |
| 2172 | NINGBO UROL NEPHROL HOSP | 1 | 0.066 |
| 2173 | NIPPON MED COLL HOSP | 1 | 0.066 |
| 2174 | NISHIDA HOSP | 1 | 0.066 |
| 2175 | NISSAY HOSP | 1 | 0.066 |
| 2176 | NORFOLK NORWICH UNIV HOSP NHS FDN TRUST | 1 | 0.066 |
| 2177 | NORTH WEST UNIV | 1 | 0.066 |
| 2178 | NORTHEAST FLORIDA ENDOCRINE DIABET ASSOCIATES | 1 | 0.066 |
| 2179 | NORTHENDEN GRP PRACTICE | 1 | 0.066 |
| 2180 | NORTHSHORE UNIV | 1 | 0.066 |
| 2181 | NORTHWELL HLTH | 1 | 0.066 |
| 2182 | NORWEGIAN INST PUBL HLTH | 1 | 0.066 |
| 2183 | NORWOOD SURG | 1 | 0.066 |
| 2184 | NOVA SOUTHEASTERN UNIV | 1 | 0.066 |
| 2185 | NOVANT HLTH PRESBYTERIAN MED CTR | 1 | 0.066 |
| 2186 | NOVARTIS PHARMA GMBH | 1 | 0.066 |
| 2187 | NOVARTIS PHARMACEUT | 1 | 0.066 |
| 2188 | NOVO NORD FDN | 1 | 0.066 |
| 2189 | NOVO NORDISK INC | 1 | 0.066 |
| 2190 | NOVO NORDISK PHARMA | 1 | 0.066 |
| 2191 | NOVO NORDISK PHARMA SA | 1 | 0.066 |
| 2192 | NTT EAST CORP | 1 | 0.066 |
| 2193 | NUIG | 1 | 0.066 |
| 2194 | NYU LANGONE MED CTR | 1 | 0.066 |
| 2195 | NYU SCH MED | 1 | 0.066 |
| 2196 | OAK ST HLTH | 1 | 0.066 |
| 2197 | OCHSNER HLTH SYST CTR OUTCOMES HLTH SERV RES | 1 | 0.066 |
| 2198 | ODAYAMA CLIN | 1 | 0.066 |
| 2199 | OITA UNIV | 1 | 0.066 |
| 2200 | OKAYAMA MED CTR | 1 | 0.066 |
| 2201 | OKAYAMA ROSAI HOSP | 1 | 0.066 |
| 2202 | OKLAHOMA CTR POISON DRUG INFORMAT | 1 | 0.066 |
| 2203 | OLD DOMINION UNIV | 1 | 0.066 |
| 2204 | OMAHA VET AFFAIRS MED CTR | 1 | 0.066 |
| 2205 | ONASSIS CARDIAC SURG CTR | 1 | 0.066 |
| 2206 | ONGA HOSP | 1 | 0.066 |
| 2207 | ONO PHARMACEUT CO LTD | 1 | 0.066 |
| 2208 | OPTUM | 1 | 0.066 |
| 2209 | OPTUMLABS | 1 | 0.066 |
| 2210 | OSAKA CITY UNIV | 1 | 0.066 |
| 2211 | OSAKA GEN MED CTR | 1 | 0.066 |
| 2212 | OSAKA ROSAI HOSP | 1 | 0.066 |
| 2213 | OSPED ACCREDITATO VILLA FIORI | 1 | 0.066 |
| 2214 | OSPED POLICLIN SAN MARTINO | 1 | 0.066 |
| 2215 | OSPED RIUNITI REGGIO CALABRIA | 1 | 0.066 |
| 2216 | OSSIAN HLTH ECON COMMUN | 1 | 0.066 |
| 2217 | OSSIAN HLTH ECON COMMUN GMBH | 1 | 0.066 |
| 2218 | OTOSHI MED CLIN | 1 | 0.066 |
| 2219 | OULU UNIV HOSP | 1 | 0.066 |
| 2220 | OXFORD PHARMAGENESIS | 1 | 0.066 |
| 2221 | OYAMA EAST CLIN | 1 | 0.066 |
| 2222 | PACE UNIV | 1 | 0.066 |
| 2223 | PAKISTAN KIDNEY LIVER INST RES CTR | 1 | 0.066 |
| 2224 | PARACELSUS MED SCH NURNBERG | 1 | 0.066 |
| 2225 | PARACELSUS MED UNIV SALZBURG | 1 | 0.066 |
| 2226 | PARIS 13 UNIV | 1 | 0.066 |
| 2227 | PARIS ILE DE FRANCE OUEST UVSQ UNIV | 1 | 0.066 |
| 2228 | PARK NICOLLET | 1 | 0.066 |
| 2229 | PAVLOV FIRST ST PETERSBURG STATE MED UNIV | 1 | 0.066 |
| 2230 | PEERLESS HOSP | 1 | 0.066 |
| 2231 | PEKING UNION MED COLL | 1 | 0.066 |
| 2232 | PELVIPHARM | 1 | 0.066 |
| 2233 | PEOPLES FRIENDSHIP UNIV RUSSIA | 1 | 0.066 |
| 2234 | PEOPLES LIBERAT ARMY GEN HOSP | 1 | 0.066 |
| 2235 | PETER MUNK CARDIAC CTR | 1 | 0.066 |
| 2236 | PFIZER | 1 | 0.066 |
| 2237 | PGIMER | 1 | 0.066 |
| 2238 | PHARMATELLIGENCE | 1 | 0.066 |
| 2239 | PHARMO INST DRUG OUTCOMES RES | 1 | 0.066 |
| 2240 | PHARMO INST DRUG OUTCOMES RES CRS | 1 | 0.066 |
| 2241 | PHILADELPHIA COLL PHARM | 1 | 0.066 |
| 2242 | PHILIPPINE GEN HOSP | 1 | 0.066 |
| 2243 | PIERRE MARIE CURIE UNIV | 1 | 0.066 |
| 2244 | PIERRE MARIE CURIE UNIV PARIS VI | 1 | 0.066 |
| 2245 | PISA UNIV | 1 | 0.066 |
| 2246 | PMMHRI | 1 | 0.066 |
| 2247 | POLICE MED CTR THESSALONIKI | 1 | 0.066 |
| 2248 | POLICE OUTPATIENT CLIN | 1 | 0.066 |
| 2249 | POLICLIN GIPUZKOA | 1 | 0.066 |
| 2250 | POLICLIN MONZA | 1 | 0.066 |
| 2251 | POLISH ACAD SCI | 1 | 0.066 |
| 2252 | PONDICHERRY INST MED SCI | 1 | 0.066 |
| 2253 | PONTIFICIA UNIV CATOLICA PARANA | 1 | 0.066 |
| 2254 | POPULAT HLTH RES INST | 1 | 0.066 |
| 2255 | PORT SHEPSTONE REG HOSP | 1 | 0.066 |
| 2256 | POZNAN UNIV MED SCI | 1 | 0.066 |
| 2257 | PRESBYTERIAN COLL SCH PHARM | 1 | 0.066 |
| 2258 | PRESIDIO OSPED RIC INRCA IRCCS | 1 | 0.066 |
| 2259 | PRIMA CARE PC | 1 | 0.066 |
| 2260 | PRIMARY CARE METAB GRP | 1 | 0.066 |
| 2261 | PRINCE HAMZAH HOSP | 1 | 0.066 |
| 2262 | PRINCESS ALEXANDRA HOSP | 1 | 0.066 |
| 2263 | PRINCESS MARGARET HOSP | 1 | 0.066 |
| 2264 | PRINCESS ROYAL UNIV HOSP | 1 | 0.066 |
| 2265 | PROFIL INST CLIN RES | 1 | 0.066 |
| 2266 | PROFIL INST STOFFWECHSELFORSCH GMBH | 1 | 0.066 |
| 2267 | PUSAN NATL UNIV | 1 | 0.066 |
| 2268 | QASSIM UNIV | 1 | 0.066 |
| 2269 | QATAR FDN | 1 | 0.066 |
| 2270 | QATAR UNIV | 1 | 0.066 |
| 2271 | QE2 HOSP HOWLANDS WELWYN GARDEN CITY | 1 | 0.066 |
| 2272 | QINGDAO UNIV | 1 | 0.066 |
| 2273 | QUEEN ELIZABETH UNIV HOSP | 1 | 0.066 |
| 2274 | QUEENS DIABET ENDOCRINOL ASSOCIATES | 1 | 0.066 |
| 2275 | QUINTILESIMS | 1 | 0.066 |
| 2276 | R D JAPAN ASTRAZENECA | 1 | 0.066 |
| 2277 | RABIN MED CTR | 1 | 0.066 |
| 2278 | RAJENDRA INST MED SCI | 1 | 0.066 |
| 2279 | RAMBAM HLTH CAMPUS | 1 | 0.066 |
| 2280 | RAMBAM MED CTR | 1 | 0.066 |
| 2281 | RC PATEL INST PHARMACEUT EDUC RES | 1 | 0.066 |
| 2282 | RCGP | 1 | 0.066 |
| 2283 | REAL WORLD EVIDENCE | 1 | 0.066 |
| 2284 | REGINA MONTIS REGALIS HOSP | 1 | 0.066 |
| 2285 | RES CONSORTIUM | 1 | 0.066 |
| 2286 | RICHARD L ROUDEBUSH VA MED CTR | 1 | 0.066 |
| 2287 | RICHFORD GATE MED PRACTICE | 1 | 0.066 |
| 2288 | RIGA STRADINS UNIV | 1 | 0.066 |
| 2289 | ROBARTS RES INST | 1 | 0.066 |
| 2290 | ROBERT KOCH MED CTR | 1 | 0.066 |
| 2291 | ROBERT WOOD JOHNSON UNIV HOSP | 1 | 0.066 |
| 2292 | ROBERTO SANTOS GEN HOSP SESAB | 1 | 0.066 |
| 2293 | RONALD REGAN UNIV CALIF LOS ANGELES | 1 | 0.066 |
| 2294 | ROUEN UNIV HOSP | 1 | 0.066 |
| 2295 | ROYAL ADELAIDE HOSP | 1 | 0.066 |
| 2296 | ROYAL ALEXANDRA HOSP | 1 | 0.066 |
| 2297 | ROYAL BROMPTON HAREFIELD NHS FDN TRUST | 1 | 0.066 |
| 2298 | ROYAL COLL GEN PRACTITIONERS | 1 | 0.066 |
| 2299 | ROYAL COMMISS HLTH SERV PROGRAM | 1 | 0.066 |
| 2300 | ROYAL DEVON EXETER HOSP | 1 | 0.066 |
| 2301 | ROYAL FREE LONDON NHS FDN TRUST | 1 | 0.066 |
| 2302 | ROYAL FREE NHS FDN TRUST | 1 | 0.066 |
| 2303 | ROYAL GLAMORGAN HOSP | 1 | 0.066 |
| 2304 | ROYAL HOBART HOSP | 1 | 0.066 |
| 2305 | ROYAL JUBILEE HOSP | 1 | 0.066 |
| 2306 | ROYAL LIVERPOOL BROADGREEN UNIV NHS HOSP TRUST | 1 | 0.066 |
| 2307 | ROYAL LIVERPOOL UNIV HOSP | 1 | 0.066 |
| 2308 | ROYAL LONDON HOSP | 1 | 0.066 |
| 2309 | ROYAL PERTH HOSP UNIT | 1 | 0.066 |
| 2310 | ROYAL WOLVERHAMPTON HOSP NHS TRUST | 1 | 0.066 |
| 2311 | RUSH UNIV | 1 | 0.066 |
| 2312 | RUSSIAN ACAD SCI | 1 | 0.066 |
| 2313 | RUTGERS CANC INST NEW JERSEY | 1 | 0.066 |
| 2314 | RUTGERS SCH PUBL HLTH | 1 | 0.066 |
| 2315 | RUTTONJEE TANG SHIU KIN HOSP | 1 | 0.066 |
| 2316 | S MARIA PIETA HOSP | 1 | 0.066 |
| 2317 | SAARLAND UNIV | 1 | 0.066 |
| 2318 | SAFETY PHARMACOL SOC | 1 | 0.066 |
| 2319 | SAGA UNIV HOSP | 1 | 0.066 |
| 2320 | SAHLGRENS ACAD | 1 | 0.066 |
| 2321 | SAIFEE HOSP | 1 | 0.066 |
| 2322 | SAISEIKAI FUTSUKAICHI HOSP | 1 | 0.066 |
| 2323 | SAISEIKAI SUITA HOSP | 1 | 0.066 |
| 2324 | SAISEIKAI YOKOHAMA SOUTH HOSP | 1 | 0.066 |
| 2325 | SAISEIKAI YOKOHAMASHI TOBU HOSP | 1 | 0.066 |
| 2326 | SALAHADDIN UNIV ERBIL | 1 | 0.066 |
| 2327 | SALFORD ROYAL NHS FDN TRUST | 1 | 0.066 |
| 2328 | SALUS INFIRMORUM UNIV CADIZ | 1 | 0.066 |
| 2329 | SAN ANTONIO ABATE HOSP | 1 | 0.066 |
| 2330 | SAN BORTOLO HOSP | 1 | 0.066 |
| 2331 | SAN FILIPPO NERI HOSP | 1 | 0.066 |
| 2332 | SAN FRANCISCO VA MED CTR | 1 | 0.066 |
| 2333 | SANDRO PERTINI HOSP | 1 | 0.066 |
| 2334 | SANDWELL WEST BIRMINGHAM HOSP NATL HLTH SERV TR | 1 | 0.066 |
| 2335 | SANNOU HOSP | 1 | 0.066 |
| 2336 | SANOFI | 1 | 0.066 |
| 2337 | SANOFI KK | 1 | 0.066 |
| 2338 | SANTA CASA SAO PAULO | 1 | 0.066 |
| 2339 | SAPIENZA UNIV | 1 | 0.066 |
| 2340 | SAPPORO DIABET THYROID CLIN | 1 | 0.066 |
| 2341 | SASAZUKA INOUE CLIN | 1 | 0.066 |
| 2342 | SASKATOON HLTH REG | 1 | 0.066 |
| 2343 | SCHNEIDER CHILDRENS MED CTR ISRAEL | 1 | 0.066 |
| 2344 | SCHRAMM | 1 | 0.066 |
| 2345 | SCI UNIV JOSEPH FOURIER | 1 | 0.066 |
| 2346 | SCOTTISH UNIV ENVIRONM RES CTR | 1 | 0.066 |
| 2347 | SCRIPPS WHITTIER DIABET INST | 1 | 0.066 |
| 2348 | SECOND MIL MED UNIV | 1 | 0.066 |
| 2349 | SEGOVIA GEN HOSP | 1 | 0.066 |
| 2350 | SEINO INTERNAL MED CLIN | 1 | 0.066 |
| 2351 | SEJONG ST MARYS DIABET ENDOCRINE CLIN | 1 | 0.066 |
| 2352 | SEKINO HOSP | 1 | 0.066 |
| 2353 | SELCUK UNIV | 1 | 0.066 |
| 2354 | SELF REG HEALTHCARE FAMILY MED RESIDENCY PROGRAM | 1 | 0.066 |
| 2355 | SERV GALLEGO SATUD XXIS SERGAS | 1 | 0.066 |
| 2356 | SERV INST MED SCI | 1 | 0.066 |
| 2357 | SESTRE MILOSRDNICE UNIV HOSP CTR | 1 | 0.066 |
| 2358 | SEVENTH DOCTOR CONSULTING | 1 | 0.066 |
| 2359 | SHANDONG UNIV | 1 | 0.066 |
| 2360 | SHANGHAI INST CARDIOVASC DIS | 1 | 0.066 |
| 2361 | SHANGHAI JIAO TONG UNIV AFFILIATED PEOPLES HOSP 6 | 1 | 0.066 |
| 2362 | SHANGHAI JIAYUE PHARMATECH | 1 | 0.066 |
| 2363 | SHANGHAI KEY LAB VISUAL IMPAIRMENT RESTORAT | 1 | 0.066 |
| 2364 | SHAWNEE MISSION HLTH ENDOCRINE CLIN | 1 | 0.066 |
| 2365 | SHEFFIELD TEACHING HOSP NHS FDN TRUST | 1 | 0.066 |
| 2366 | SHIKOKU CENT HOSP | 1 | 0.066 |
| 2367 | SHIN KOGA HOSP | 1 | 0.066 |
| 2368 | SHIRAIWA MED CLIN | 1 | 0.066 |
| 2369 | SHIZUOKA MED CTR | 1 | 0.066 |
| 2370 | SHIZUOKA PREFECTURAL GEN HOSP | 1 | 0.066 |
| 2371 | SHREE HINDU MANDAL HOSP | 1 | 0.066 |
| 2372 | SHRINERS HOSP CHILDREN | 1 | 0.066 |
| 2373 | SHSCT | 1 | 0.066 |
| 2374 | SICHUAN MENTAL HLTH CTR | 1 | 0.066 |
| 2375 | SIDRA MED RES CTR | 1 | 0.066 |
| 2376 | SIJHIH CATHAY GEN HOSP | 1 | 0.066 |
| 2377 | SINAI HLTH SYST | 1 | 0.066 |
| 2378 | SINAI HOSP | 1 | 0.066 |
| 2379 | SINGHLTH DUKE NUS | 1 | 0.066 |
| 2380 | SINGLETON HOSP | 1 | 0.066 |
| 2381 | SIR CHARLES GAIRDNER HOSP | 1 | 0.066 |
| 2382 | SISTERS CHAR UNIV HOSP | 1 | 0.066 |
| 2383 | SKANE UNIV HOSP | 1 | 0.066 |
| 2384 | SOOKMYUNG WOMENS UNIV | 1 | 0.066 |
| 2385 | SOUTH AFRICAN MED RES COUNCIL | 1 | 0.066 |
| 2386 | SOUTH AUSTRALIAN HLTH MED RES INST | 1 | 0.066 |
| 2387 | SOUTH LONDON MAUDSLEY NHS FDN TRUST | 1 | 0.066 |
| 2388 | SOUTH OSTROBOTHNIA HOSP DIST | 1 | 0.066 |
| 2389 | SOUTHEAST LOUISIANA VET HLTH CARE SYST | 1 | 0.066 |
| 2390 | SOUTHERN MED UNIV | 1 | 0.066 |
| 2391 | SOUTHSIDE ENDOCRINOL DIABET THYROID ASSOCIATE | 1 | 0.066 |
| 2392 | SPANISH SOC NEPHROL | 1 | 0.066 |
| 2393 | SPAULDING CLIN RES | 1 | 0.066 |
| 2394 | SPRINGER NAT | 1 | 0.066 |
| 2395 | SRINAKHARINWIROT UNIV | 1 | 0.066 |
| 2396 | SRINATH INST PHARMACEUT EDUC RES | 1 | 0.066 |
| 2397 | ST BONIFACE GEN HOSP | 1 | 0.066 |
| 2398 | ST CHARLES HOSP | 1 | 0.066 |
| 2399 | ST COLUMCILLES HOSP | 1 | 0.066 |
| 2400 | ST FRANCIS HOSP | 1 | 0.066 |
| 2401 | ST GEORGE HOSP | 1 | 0.066 |
| 2402 | ST GEORGES HOSP NHS TRUST UNIV LONDON | 1 | 0.066 |
| 2403 | ST GEORGES UNIV HOSP NHS | 1 | 0.066 |
| 2404 | ST GORANS UNIV HOSP | 1 | 0.066 |
| 2405 | ST JOSEPH ST LUC HOSP | 1 | 0.066 |
| 2406 | ST JOSEPHS HLTH CARE | 1 | 0.066 |
| 2407 | ST KLIMENT OHRIDSKY UNIV | 1 | 0.066 |
| 2408 | ST MARYS HOSP LUODONG | 1 | 0.066 |
| 2409 | ST PAULS HOSP | 1 | 0.066 |
| 2410 | ST PETERSBURG CHEM PHARMACEUT UNIV | 1 | 0.066 |
| 2411 | ST THOMAS HOSP | 1 | 0.066 |
| 2412 | ST VINCENTS HLTH | 1 | 0.066 |
| 2413 | ST VINCENTS HOSP MELBOURNE | 1 | 0.066 |
| 2414 | STANDORT BERLIN CHARITE | 1 | 0.066 |
| 2415 | STANFORD STROKE CTR | 1 | 0.066 |
| 2416 | STATISTICON | 1 | 0.066 |
| 2417 | STEEL MEM MURORAN HOSP | 1 | 0.066 |
| 2418 | STENO DIABET CTR AS | 1 | 0.066 |
| 2419 | STEPHENS INSURANCE | 1 | 0.066 |
| 2420 | SUITA MUNICIPAL HOSP | 1 | 0.066 |
| 2421 | SULEYMAN DEMIREL UNIV | 1 | 0.066 |
| 2422 | SUNCHON NATL UNIV | 1 | 0.066 |
| 2423 | SUNY BINGHAMTON | 1 | 0.066 |
| 2424 | SUNY DOWNSTATE COLL MED | 1 | 0.066 |
| 2425 | SUNY DOWNSTATE MED CTR | 1 | 0.066 |
| 2426 | SWEDISH INST HLTH ECON | 1 | 0.066 |
| 2427 | SWEDISH RES COUNCIL | 1 | 0.066 |
| 2428 | SWINBURNE UNIV TECHNOL | 1 | 0.066 |
| 2429 | TACHIKAWA GEN HOSP | 1 | 0.066 |
| 2430 | TAIBAH UNIV | 1 | 0.066 |
| 2431 | TAIPEI MED UNIV HOSP | 1 | 0.066 |
| 2432 | TAKEDA PHARMACEUT CO LTD | 1 | 0.066 |
| 2433 | TAKING CONTROL YOUR DIABET | 1 | 0.066 |
| 2434 | TALWALKAR DIABET CLIN | 1 | 0.066 |
| 2435 | TAN TOCK SENG HOSP | 1 | 0.066 |
| 2436 | TANAKA CLIN | 1 | 0.066 |
| 2437 | TARAS SHEVCHENKO NATL UNIV KYIV | 1 | 0.066 |
| 2438 | TAWAM HOSP | 1 | 0.066 |
| 2439 | TED ROGERS CTR HEART RES | 1 | 0.066 |
| 2440 | TEL AVIV SOURASKY MED CTR | 1 | 0.066 |
| 2441 | TEL AVIV UNIV MACCABI HEALTHCARE ISRAEL | 1 | 0.066 |
| 2442 | TERVEYSTALO | 1 | 0.066 |
| 2443 | TEXAS DIABET ENDOCRINOL | 1 | 0.066 |
| 2444 | TEXAS DIABET INST | 1 | 0.066 |
| 2445 | THE MEDICITY | 1 | 0.066 |
| 2446 | THERESIENKRANKENHAUS | 1 | 0.066 |
| 2447 | THIRD MIL MED UNIV | 1 | 0.066 |
| 2448 | THOMAS JEFFERSON UNIV HOSP | 1 | 0.066 |
| 2449 | THROMBOLYSIS MYOCARDIAL INFARCT STUDY OFF | 1 | 0.066 |
| 2450 | TIANJIN UNIV SPORT | 1 | 0.066 |
| 2451 | TIMI STUDY OFF | 1 | 0.066 |
| 2452 | TOHNO CHUO CLIN | 1 | 0.066 |
| 2453 | TOHTO CLIN | 1 | 0.066 |
| 2454 | TOKYO KYOSAI HOSP | 1 | 0.066 |
| 2455 | TOKYO METROPOLITAN INST MED SCI | 1 | 0.066 |
| 2456 | TOKYO UNIV PHARM LIFE SCI | 1 | 0.066 |
| 2457 | TOKYO UNIV SCI RIKADAI | 1 | 0.066 |
| 2458 | TOLLEY HLTH ECON | 1 | 0.066 |
| 2459 | TOLLEY HLTH ECON LTD | 1 | 0.066 |
| 2460 | TOR VERGATA UNIV | 1 | 0.066 |
| 2461 | TORANOMON GEN HOSP | 1 | 0.066 |
| 2462 | TORONTO EAST GEN HOSP | 1 | 0.066 |
| 2463 | TOTALCARDIOL RES NETWORK | 1 | 0.066 |
| 2464 | TRANSILVANIA UNIV | 1 | 0.066 |
| 2465 | TRANSYLVANIA UNIV BRASOV | 1 | 0.066 |
| 2466 | TREANT ZORGGRP | 1 | 0.066 |
| 2467 | TRINETX INC | 1 | 0.066 |
| 2468 | TRUMAN MED CTR | 1 | 0.066 |
| 2469 | TSUMURA CLIN | 1 | 0.066 |
| 2470 | TSURUOKA KYORITSU HOSP | 1 | 0.066 |
| 2471 | TUEN MUN HOSP | 1 | 0.066 |
| 2472 | TULANE SCH MED | 1 | 0.066 |
| 2473 | TZU CHI FDN | 1 | 0.066 |
| 2474 | TZU CHI UNIV HOSP | 1 | 0.066 |
| 2475 | UC RIVERSIDE SCH MED | 1 | 0.066 |
| 2476 | UC SAN DIEGO MED CTR | 1 | 0.066 |
| 2477 | UCHIYAMA CLIN | 1 | 0.066 |
| 2478 | UCLA | 1 | 0.066 |
| 2479 | UCSD VA SAN DIEGO HEALTHCARE | 1 | 0.066 |
| 2480 | UCSF PROGRAM | 1 | 0.066 |
| 2481 | UCSF SCH MED | 1 | 0.066 |
| 2482 | UHL NHS TRUST | 1 | 0.066 |
| 2483 | ULM UNIV | 1 | 0.066 |
| 2484 | ULSS N 6 EUGANEA | 1 | 0.066 |
| 2485 | UMASS MED SCH | 1 | 0.066 |
| 2486 | UMBERTO HOSP | 1 | 0.066 |
| 2487 | UMP MED CTR | 1 | 0.066 |
| 2488 | UMR CNRS 7021 LAB BIOIMAGERIE PATHOL | 1 | 0.066 |
| 2489 | UNC GILLING GLOBAL SCH PUBL HLTH | 1 | 0.066 |
| 2490 | UNGER CONCIERGE PRIMARY CARE MED GRP | 1 | 0.066 |
| 2491 | UNICAEN | 1 | 0.066 |
| 2492 | UNITED CHRISTIAN HOSP | 1 | 0.066 |
| 2493 | UNITEDHLTH GRP | 1 | 0.066 |
| 2494 | UNITY HLTH NETWORK | 1 | 0.066 |
| 2495 | UNIV A CORUNA | 1 | 0.066 |
| 2496 | UNIV ALBERTA EDMONTON | 1 | 0.066 |
| 2497 | UNIV ALCALA DE HENARES | 1 | 0.066 |
| 2498 | UNIV APPL SCI KAISERSLAUTERN | 1 | 0.066 |
| 2499 | UNIV AUTONOMA NUEVO LEON | 1 | 0.066 |
| 2500 | UNIV AUTONOMA TAMAULIPAS | 1 | 0.066 |
| 2501 | UNIV BAGHDAD | 1 | 0.066 |
| 2502 | UNIV BARCELONA | 1 | 0.066 |
| 2503 | UNIV BASEL | 1 | 0.066 |
| 2504 | UNIV BERGEN | 1 | 0.066 |
| 2505 | UNIV BERN | 1 | 0.066 |
| 2506 | UNIV BIRMINGHAM EDGBASTON | 1 | 0.066 |
| 2507 | UNIV BORDEAUX SEGALEN | 1 | 0.066 |
| 2508 | UNIV BRADFORD | 1 | 0.066 |
| 2509 | UNIV BUFFALO | 1 | 0.066 |
| 2510 | UNIV CADIZ | 1 | 0.066 |
| 2511 | UNIV CALIF DAVIS | 1 | 0.066 |
| 2512 | UNIV CALIF IRVINE | 1 | 0.066 |
| 2513 | UNIV CAMBRIDGE | 1 | 0.066 |
| 2514 | UNIV CAMPUS BIOMED | 1 | 0.066 |
| 2515 | UNIV CARLO BO URBINO | 1 | 0.066 |
| 2516 | UNIV CATANZARO | 1 | 0.066 |
| 2517 | UNIV CENT LANCASHIRE | 1 | 0.066 |
| 2518 | UNIV CHICAGO | 1 | 0.066 |
| 2519 | UNIV CLIN HOSP VALENCIA | 1 | 0.066 |
| 2520 | UNIV CLIN WURZBURG | 1 | 0.066 |
| 2521 | UNIV COLL LONDON HOSP | 1 | 0.066 |
| 2522 | UNIV COLL MED SCH | 1 | 0.066 |
| 2523 | UNIV COLORADO ANSCHUTZ MED CAMPUS | 1 | 0.066 |
| 2524 | UNIV COLORADO HOSP | 1 | 0.066 |
| 2525 | UNIV COMPLUTENSE MADRID | 1 | 0.066 |
| 2526 | UNIV DEBRECEN | 1 | 0.066 |
| 2527 | UNIV DENIS DIDEROT PARIS 7 | 1 | 0.066 |
| 2528 | UNIV DIYALA | 1 | 0.066 |
| 2529 | UNIV DORTMUND | 1 | 0.066 |
| 2530 | UNIV DUSSELDORF | 1 | 0.066 |
| 2531 | UNIV ESTADO RIO DE JANEIRO | 1 | 0.066 |
| 2532 | UNIV ESTADUAL CIENCIAS SAUDE ALAGOAS UNCISAL | 1 | 0.066 |
| 2533 | UNIV EUROPEA MADRID | 1 | 0.066 |
| 2534 | UNIV EXETER | 1 | 0.066 |
| 2535 | UNIV FED ALAGOAS | 1 | 0.066 |
| 2536 | UNIV FED ALAGOAS UFAL | 1 | 0.066 |
| 2537 | UNIV FED CEARA | 1 | 0.066 |
| 2538 | UNIV FED UBERLANDIA | 1 | 0.066 |
| 2539 | UNIV FEDERICO II | 1 | 0.066 |
| 2540 | UNIV FERRARA | 1 | 0.066 |
| 2541 | UNIV FLORIDA HLTH | 1 | 0.066 |
| 2542 | UNIV FUKUI | 1 | 0.066 |
| 2543 | UNIV G DANNUNZIO CHIETI | 1 | 0.066 |
| 2544 | UNIV GENEVA HOSP | 1 | 0.066 |
| 2545 | UNIV GHENT | 1 | 0.066 |
| 2546 | UNIV GOTHENBERG | 1 | 0.066 |
| 2547 | UNIV GRANADA | 1 | 0.066 |
| 2548 | UNIV GREGORIO MARANON | 1 | 0.066 |
| 2549 | UNIV GUADALAJARA | 1 | 0.066 |
| 2550 | UNIV HAIFA | 1 | 0.066 |
| 2551 | UNIV HALLE | 1 | 0.066 |
| 2552 | UNIV HASSELT | 1 | 0.066 |
| 2553 | UNIV HEIDELBERG HOSP | 1 | 0.066 |
| 2554 | UNIV HLTH BOARD | 1 | 0.066 |
| 2555 | UNIV HOSP ALEXANDROUPOLIS | 1 | 0.066 |
| 2556 | UNIV HOSP BASEL | 1 | 0.066 |
| 2557 | UNIV HOSP BIRMINGHAM | 1 | 0.066 |
| 2558 | UNIV HOSP CIUDAD REAL | 1 | 0.066 |
| 2559 | UNIV HOSP COVENTRY | 1 | 0.066 |
| 2560 | UNIV HOSP COVENTRY WARWICKSHIRE NHS TRUST | 1 | 0.066 |
| 2561 | UNIV HOSP CTR ZAGREB | 1 | 0.066 |
| 2562 | UNIV HOSP DIJON | 1 | 0.066 |
| 2563 | UNIV HOSP DOCTOR PESET | 1 | 0.066 |
| 2564 | UNIV HOSP ERLANGEN | 1 | 0.066 |
| 2565 | UNIV HOSP ESSEN | 1 | 0.066 |
| 2566 | UNIV HOSP FERRARA | 1 | 0.066 |
| 2567 | UNIV HOSP GERMANS TRIAS PUJOL | 1 | 0.066 |
| 2568 | UNIV HOSP GRENOBLE | 1 | 0.066 |
| 2569 | UNIV HOSP HALLE | 1 | 0.066 |
| 2570 | UNIV HOSP HEIDELBERG | 1 | 0.066 |
| 2571 | UNIV HOSP LAUSANNE | 1 | 0.066 |
| 2572 | UNIV HOSP MORECAMBE NHS TRUST | 1 | 0.066 |
| 2573 | UNIV HOSP MOTOL | 1 | 0.066 |
| 2574 | UNIV HOSP NORTH NORWAY | 1 | 0.066 |
| 2575 | UNIV HOSP SISTERS MERCY | 1 | 0.066 |
| 2576 | UNIV HOSP ST LOUIS LARIBOISIERE | 1 | 0.066 |
| 2577 | UNIV HOSP ST POLTEN | 1 | 0.066 |
| 2578 | UNIV HOSP SVETI DUH | 1 | 0.066 |
| 2579 | UNIV HOSP TUBINGEN | 1 | 0.066 |
| 2580 | UNIV HOSP VERONA | 1 | 0.066 |
| 2581 | UNIV HOUSTON | 1 | 0.066 |
| 2582 | UNIV HYPERTENS CTR | 1 | 0.066 |
| 2583 | UNIV JAEN | 1 | 0.066 |
| 2584 | UNIV JAUME 1 | 1 | 0.066 |
| 2585 | UNIV JJ STROSSMAYER OSIJEK | 1 | 0.066 |
| 2586 | UNIV KENTUCKY | 1 | 0.066 |
| 2587 | UNIV KLINIKUM | 1 | 0.066 |
| 2588 | UNIV KLINIKUM AACHEN | 1 | 0.066 |
| 2589 | UNIV KLINIKUM REGENSBURG | 1 | 0.066 |
| 2590 | UNIV KWAZULU NATAL | 1 | 0.066 |
| 2591 | UNIV LA FRONTERA | 1 | 0.066 |
| 2592 | UNIV LATVIA | 1 | 0.066 |
| 2593 | UNIV LEUVEN | 1 | 0.066 |
| 2594 | UNIV LINKOPING | 1 | 0.066 |
| 2595 | UNIV LISBON | 1 | 0.066 |
| 2596 | UNIV LJUBLJANA | 1 | 0.066 |
| 2597 | UNIV LLEIDA | 1 | 0.066 |
| 2598 | UNIV LONDON IMPERIAL COLL SCI TECHNOL MED | 1 | 0.066 |
| 2599 | UNIV LOUISVILLE | 1 | 0.066 |
| 2600 | UNIV LYON | 1 | 0.066 |
| 2601 | UNIV LYON 1 | 1 | 0.066 |
| 2602 | UNIV MARIBOR | 1 | 0.066 |
| 2603 | UNIV MED 2 | 1 | 0.066 |
| 2604 | UNIV MED CTR GOETTINGEN | 1 | 0.066 |
| 2605 | UNIV MED CTR GROININGEN | 1 | 0.066 |
| 2606 | UNIV MED CTR UTRECHT | 1 | 0.066 |
| 2607 | UNIV MED GOTTINGEN | 1 | 0.066 |
| 2608 | UNIV MED PHARM | 1 | 0.066 |
| 2609 | UNIV MIYAZAKI | 1 | 0.066 |
| 2610 | UNIV MONTANA | 1 | 0.066 |
| 2611 | UNIV MUNICH | 1 | 0.066 |
| 2612 | UNIV NACL LA PLATA | 1 | 0.066 |
| 2613 | UNIV NEWCASTLE | 1 | 0.066 |
| 2614 | UNIV NORTH CAROLINA CHAPEL HILL | 1 | 0.066 |
| 2615 | UNIV NORTH TEXAS SYST | 1 | 0.066 |
| 2616 | UNIV NOVA LISBOA | 1 | 0.066 |
| 2617 | UNIV NSW | 1 | 0.066 |
| 2618 | UNIV OKLAHOMA | 1 | 0.066 |
| 2619 | UNIV OTTAWA | 1 | 0.066 |
| 2620 | UNIV OTTAWA HEART INST | 1 | 0.066 |
| 2621 | UNIV OULU | 1 | 0.066 |
| 2622 | UNIV OVIEDO | 1 | 0.066 |
| 2623 | UNIV PARIS | 1 | 0.066 |
| 2624 | UNIV PARIS DIDEROT | 1 | 0.066 |
| 2625 | UNIV PATRAS | 1 | 0.066 |
| 2626 | UNIV PHILIPPINES | 1 | 0.066 |
| 2627 | UNIV PITTSBURGH | 1 | 0.066 |
| 2628 | UNIV QUEENSLAND BIOL RESOURCES | 1 | 0.066 |
| 2629 | UNIV REGENSBURG | 1 | 0.066 |
| 2630 | UNIV RIJEKA | 1 | 0.066 |
| 2631 | UNIV ROMA LA SAPIENZA | 1 | 0.066 |
| 2632 | UNIV ROMA TOR VERGATA | 1 | 0.066 |
| 2633 | UNIV RYUKYUS HOSP | 1 | 0.066 |
| 2634 | UNIV S AUSTRALIA | 1 | 0.066 |
| 2635 | UNIV SANTANDER UDES | 1 | 0.066 |
| 2636 | UNIV SANTIAGO COMPOSTELA | 1 | 0.066 |
| 2637 | UNIV SANTO | 1 | 0.066 |
| 2638 | UNIV SANTO TOMAS HOSP | 1 | 0.066 |
| 2639 | UNIV SAO JOAO | 1 | 0.066 |
| 2640 | UNIV SAO PAOLO | 1 | 0.066 |
| 2641 | UNIV SASKATCHEWAN | 1 | 0.066 |
| 2642 | UNIV SHEFFIELD | 1 | 0.066 |
| 2643 | UNIV SHERBROOKE | 1 | 0.066 |
| 2644 | UNIV SOUTH AUSTRALIA | 1 | 0.066 |
| 2645 | UNIV SOUTH DAKOTA | 1 | 0.066 |
| 2646 | UNIV SPITAL BASEL | 1 | 0.066 |
| 2647 | UNIV ST ANDREWS | 1 | 0.066 |
| 2648 | UNIV ST JOSEPH | 1 | 0.066 |
| 2649 | UNIV STRASBOURG | 1 | 0.066 |
| 2650 | UNIV SULAIMANI | 1 | 0.066 |
| 2651 | UNIV TAMPERE | 1 | 0.066 |
| 2652 | UNIV TAOYUAN | 1 | 0.066 |
| 2653 | UNIV TASMANIA | 1 | 0.066 |
| 2654 | UNIV TEXAS AUSTIN | 1 | 0.066 |
| 2655 | UNIV TEXAS EL PASO | 1 | 0.066 |
| 2656 | UNIV TEXAS HLTH SCI CTR HOUSTON | 1 | 0.066 |
| 2657 | UNIV TEXAS RIO GRANDE VALLEY | 1 | 0.066 |
| 2658 | UNIV THESSALONIKI | 1 | 0.066 |
| 2659 | UNIV TOLEDO | 1 | 0.066 |
| 2660 | UNIV TOR VERGATA | 1 | 0.066 |
| 2661 | UNIV VERONA | 1 | 0.066 |
| 2662 | UNIV VERSAILLES ST QUENTIN YVELINES | 1 | 0.066 |
| 2663 | UNIV VET MED VIENNA | 1 | 0.066 |
| 2664 | UNIV VIRGINIA | 1 | 0.066 |
| 2665 | UNIV WARMIA MAZURY | 1 | 0.066 |
| 2666 | UNIV WEST SCOTLAND | 1 | 0.066 |
| 2667 | UNIV WESTERN SYDNEY | 1 | 0.066 |
| 2668 | UNIV WITWATERSRAND | 1 | 0.066 |
| 2669 | UNIV YAMANASHI | 1 | 0.066 |
| 2670 | UNIV YAOUNDE I | 1 | 0.066 |
| 2671 | UNIV ZAWIA | 1 | 0.066 |
| 2672 | UNIV ZIEKENHUIS LEUVEN | 1 | 0.066 |
| 2673 | UNIV ZURICH HOSP | 1 | 0.066 |
| 2674 | UNIWERSYTET JAGIELLONSKI | 1 | 0.066 |
| 2675 | UNIWERSYTET WARMINSKO MAZURSKI OLSZTYNIE | 1 | 0.066 |
| 2676 | UNLP | 1 | 0.066 |
| 2677 | UNSW | 1 | 0.066 |
| 2678 | UO DIABETOL ASUR AV4 | 1 | 0.066 |
| 2679 | UPMC UNIV PARIS 06 | 1 | 0.066 |
| 2680 | UPPSALA CLIN RES CTR | 1 | 0.066 |
| 2681 | US MED AFFAIRS | 1 | 0.066 |
| 2682 | UTHLTH SCH PUBL HLTH | 1 | 0.066 |
| 2683 | UTSUNOMIYA HIGASHI HOSP | 1 | 0.066 |
| 2684 | VA MED CTR LA JOLLA | 1 | 0.066 |
| 2685 | VA NEBRASKA WESTERN IOWA HLTH CARE SYST | 1 | 0.066 |
| 2686 | VA TENNESSEE VALLEY HEALTHCARE SYST | 1 | 0.066 |
| 2687 | VALL DHEBRON RES INST | 1 | 0.066 |
| 2688 | VAMC CINCINNATI | 1 | 0.066 |
| 2689 | VANDERBILT HEART VASC INST | 1 | 0.066 |
| 2690 | VANDERBILT UNIV SCH MED | 1 | 0.066 |
| 2691 | VENETIAN INST MOL MED | 1 | 0.066 |
| 2692 | VENETO INST MOL MED | 1 | 0.066 |
| 2693 | VERONA UNIV HOSP | 1 | 0.066 |
| 2694 | VESTRE VIKEN HOSP TRUST | 1 | 0.066 |
| 2695 | VET AFFAIRS ANN ARBOR HEALTHCARE SYST | 1 | 0.066 |
| 2696 | VET AFFAIRS MED CTR WASHINGTON | 1 | 0.066 |
| 2697 | VET AFFAIRS PUGET SOUND HLTH CARE SYST S123 PCC | 1 | 0.066 |
| 2698 | VETERANS AFFAIRS PALO ALTO HLTH CARE SYST | 1 | 0.066 |
| 2699 | VICTOR BABES UNIV MED PHARM | 1 | 0.066 |
| 2700 | VIENNA GEN HOSP | 1 | 0.066 |
| 2701 | VILLANOVA UNIV | 1 | 0.066 |
| 2702 | VINAYA HOSP RES CTR | 1 | 0.066 |
| 2703 | VIRGEN MACARENA HOSP | 1 | 0.066 |
| 2704 | VIRGEN MACARENA UNIV HOSP | 1 | 0.066 |
| 2705 | VM BIOPHARMA | 1 | 0.066 |
| 2706 | VOLGOGRAD MED UNIV | 1 | 0.066 |
| 2707 | VOLGOGRAD STATE MED UNIV | 1 | 0.066 |
| 2708 | VORARLBERG INST VASC INVEST TREATMENT | 1 | 0.066 |
| 2709 | VORARLBERG INST VASC INVEST TREATMENT VIVIT | 1 | 0.066 |
| 2710 | WAKAGI CLIN | 1 | 0.066 |
| 2711 | WAKISAKA NAIKA WAKISAKA INTERNAL MED CLIN | 1 | 0.066 |
| 2712 | WALSALL MANOR HOSP | 1 | 0.066 |
| 2713 | WALTER REED NATL MIL MED CTR | 1 | 0.066 |
| 2714 | WARNEFORD HOSP | 1 | 0.066 |
| 2715 | WARWICKSHIRE NHS TRUST | 1 | 0.066 |
| 2716 | WASHINGTON VET AFFAIRS MED CTR | 1 | 0.066 |
| 2717 | WEILL CORNELL MED COLL QATAR | 1 | 0.066 |
| 2718 | WEILL CORNELL MED CTR | 1 | 0.066 |
| 2719 | WEST KAZAKHSTAN MARAT OSPANOV STATE MED UNIV | 1 | 0.066 |
| 2720 | WEST TEXAS REG POISON CTR | 1 | 0.066 |
| 2721 | WEST VIRGINIA UNIV | 1 | 0.066 |
| 2722 | WESTERN INFIRM ASSOCIATED HOSP | 1 | 0.066 |
| 2723 | WESTERN MELBOURNE HOSP | 1 | 0.066 |
| 2724 | WESTERN SYDNEY UNIV BLACKTOWN CAMPUS | 1 | 0.066 |
| 2725 | WESTERN UNIV CANADA | 1 | 0.066 |
| 2726 | WESTMEAD PRIVATE HOSP | 1 | 0.066 |
| 2727 | WHITBY CARDIOVASC INST | 1 | 0.066 |
| 2728 | WILHELMINENSPITAL STADT WIEN | 1 | 0.066 |
| 2729 | WINGATE INST PHYS EDUC SPORTS | 1 | 0.066 |
| 2730 | WINGATE UNIV | 1 | 0.066 |
| 2731 | WOMENS HEART CARE | 1 | 0.066 |
| 2732 | WREXHAM MAELOR HOSP | 1 | 0.066 |
| 2733 | WRIGHT CTR GRAD MED EDUC | 1 | 0.066 |
| 2734 | WUHAN POLYTECH UNIV | 1 | 0.066 |
| 2735 | XERENCIA XEST INTEGRADA SANTIAGO XXIS SERGAS | 1 | 0.066 |
| 2736 | XI AN JIAO TONG UNIV | 1 | 0.066 |
| 2737 | XUZHOU CTR HOSP | 1 | 0.066 |
| 2738 | XUZHOU INST MED SCI | 1 | 0.066 |
| 2739 | XUZHOU MED UNIV | 1 | 0.066 |
| 2740 | YALE ENDOCRINOL | 1 | 0.066 |
| 2741 | YALE SHOOL MED | 1 | 0.066 |
| 2742 | YAN CHAI HOSP | 1 | 0.066 |
| 2743 | YASHODEEP INST PHARM | 1 | 0.066 |
| 2744 | YB CHAVAN COLL PHARM | 1 | 0.066 |
| 2745 | YOKOHAMA CHUO HOSP | 1 | 0.066 |
| 2746 | YOKOHAMA MINAMI KYOUSAI HOSP | 1 | 0.066 |
| 2747 | YOKOHAMA SAKAE KYOSAI HOSP | 1 | 0.066 |
| 2748 | YONG LOO LIN SCH MED | 1 | 0.066 |
| 2749 | YORK UNIV | 1 | 0.066 |
| 2750 | YUKARIGAOKA TOKUYAMA MED CLIN | 1 | 0.066 |
| 2751 | YUYAO PEOPLES HOSP | 1 | 0.066 |
| 2752 | ZAKYNTHOS HOSP | 1 | 0.066 |
| 2753 | ZEALAND UNIV HOSP | 1 | 0.066 |
| 2754 | ZHENGZHOU UNIV | 1 | 0.066 |
| 2755 | ZIEKENHUIS OOST | 1 | 0.066 |
| 2756 | ZINMAN MT SINAI HOSP | 1 | 0.066 |
| 2757 | ZUCKER HILLSIDE HOSP | 1 | 0.066 |

**Note:** SGLT2: Sodium Glucose Cotransporter 2. CV: cardiovascular
