# Supplementary material for: Bibliometric Study of Sodium Glucose Cotransporter 2 Inhibitors in Cardiovascular Research
Source: Front Pharmacol. 2020 Sep 15;11:561494. doi: 10.3389/fphar.2020.561494 (PMC7522576; doi:10.3389/fphar.2020.561494)
Supplement: Supplementary file 3 [file Table_3.docx]

Supplementary Material

**
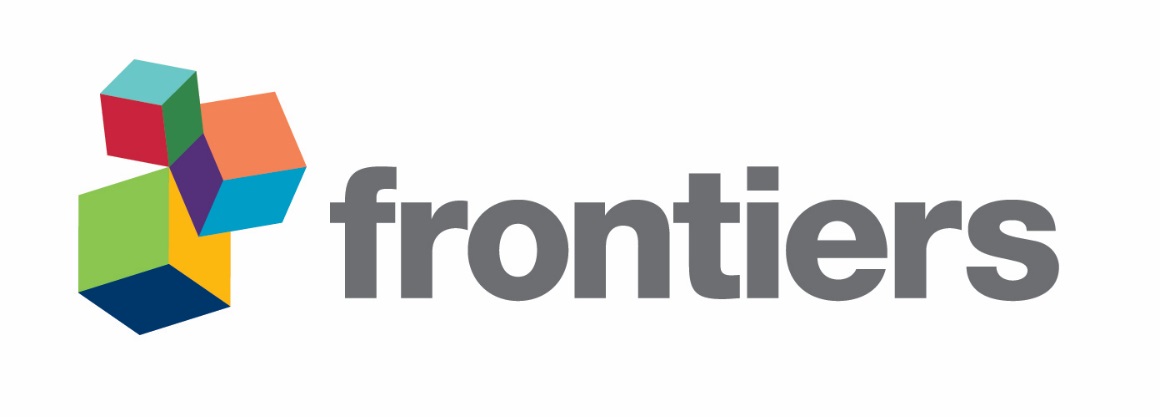
**

**Supplementary Table 3.** The collaborative country/regions publishing more than 10 documents.

| **Rank** | **Country/regions** | **Total link strength** | **Documents** | **citations** |
| --- | --- | --- | --- | --- |
| 1 | usa | 792 | 644 | 17711 |
| 2 | england | 508 | 192 | 9990 |
| 3 | sweden | 405 | 107 | 4239 |
| 4 | canada | 361 | 150 | 5861 |
| 5 | germany | 361 | 140 | 4883 |
| 6 | netherlands | 294 | 81 | 5579 |
| 7 | australia | 273 | 109 | 5234 |
| 8 | denmark | 233 | 56 | 2605 |
| 9 | italy | 220 | 126 | 3429 |
| 10 | japan | 199 | 173 | 3929 |
| 11 | scotland | 191 | 39 | 1245 |
| 12 | poland | 171 | 26 | 632 |
| 13 | peoples r china (mainland) | 167 | 98 | 1873 |
| 14 | france | 159 | 49 | 1194 |
| 15 | greece | 155 | 82 | 2356 |
| 16 | israel | 140 | 34 | 2053 |
| 17 | belgium | 127 | 56 | 2256 |
| 18 | brazil | 124 | 25 | 402 |
| 19 | norway | 120 | 34 | 1520 |
| 20 | spain | 114 | 51 | 698 |
| 21 | hungary | 111 | 13 | 317 |
| 22 | argentina | 108 | 12 | 353 |
| 23 | russia | 102 | 14 | 419 |
| 24 | peoples r china (taiwan) | 83 | 26 | 466 |
| 25 | india | 80 | 38 | 560 |
| 26 | austria | 73 | 19 | 383 |
| 27 | singapore | 66 | 16 | 320 |
| 28 | mexico | 63 | 12 | 40 |
| 29 | romania | 54 | 11 | 274 |
| 30 | south korea | 52 | 36 | 472 |
| 31 | switzerland | 43 | 15 | 293 |
| 32 | croatia | 34 | 10 | 179 |
| 33 | north ireland | 34 | 10 | 114 |
| 34 | finland | 33 | 12 | 745 |
| 35 | wales | 29 | 16 | 186 |
| 36 | turkey | 22 | 10 | 37 |
| 37 | thailand | 19 | 10 | 98 |
| 38 | qatar | 12 | 10 | 290 |
