# Supplementary material for: Bibliometric Study of Sodium Glucose Cotransporter 2 Inhibitors in Cardiovascular Research
Source: Front Pharmacol. 2020 Sep 15;11:561494. doi: 10.3389/fphar.2020.561494 (PMC7522576; doi:10.3389/fphar.2020.561494)
Supplement: Supplementary file 4 [file Table_4.docx]

Supplementary Material

**
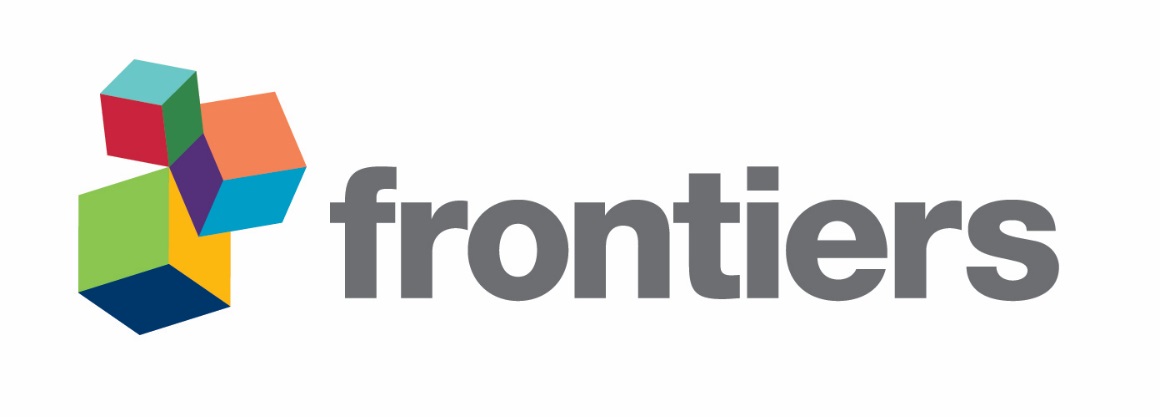
**

**Supplementary Table 4.** The collaborative organizations publishing more than 10 documents.

| **Rank** | **Organization** | **Total link strength** | **Document** | **Citation** |
| --- | --- | --- | --- | --- |
| 1 | univ toronto | 307 | 104 | 4839 |
| 2 | univ groningen | 233 | 51 | 4434 |
| 3 | brigham & womens hosp | 218 | 37 | 2719 |
| 4 | stanford univ | 205 | 34 | 3413 |
| 5 | harvard med sch | 194 | 52 | 2385 |
| 6 | astrazeneca | 189 | 68 | 2298 |
| 7 | univ sydney | 188 | 37 | 3884 |
| 8 | imperial coll london | 167 | 29 | 3276 |
| 9 | janssen res & dev llc | 151 | 36 | 1869 |
| 10 | univ oxford | 130 | 34 | 3649 |
| 11 | univ liverpool | 123 | 30 | 2171 |
| 12 | univ texas southwestern med ctr dallas | 122 | 34 | 1996 |
| 13 | royal north shore hosp | 117 | 15 | 2746 |
| 14 | univ missouri | 105 | 28 | 792 |
| 15 | st lukes mid amer heart inst | 100 | 21 | 861 |
| 16 | univ new south wales | 97 | 16 | 2243 |
| 17 | boehringer ingelheim pharma gmbh & co kg | 91 | 43 | 2262 |
| 18 | univ leicester | 91 | 27 | 1643 |
| 19 | concord repatriat gen hosp | 81 | 10 | 969 |
| 20 | univ n carolina | 81 | 14 | 1373 |
| 21 | univ utah | 75 | 10 | 706 |
| 22 | karolinska inst | 74 | 24 | 939 |
| 23 | univ british columbia | 73 | 10 | 742 |
| 24 | yale univ | 71 | 21 | 615 |
| 25 | duke univ | 69 | 21 | 1015 |
| 26 | peking univ | 69 | 18 | 742 |
| 27 | statisticon ab | 69 | 13 | 814 |
| 28 | univ chicago med | 67 | 11 | 852 |
| 29 | massachusetts gen hosp | 66 | 16 | 1966 |
| 30 | unsw sydney | 66 | 10 | 2208 |
| 31 | ucl | 65 | 16 | 661 |
| 32 | royal prince alfred hosp | 63 | 10 | 797 |
| 33 | univ copenhagen | 62 | 23 | 1089 |
| 34 | boehringer ingelheim int gmbh | 60 | 16 | 183 |
| 35 | george inst global hlth | 60 | 15 | 390 |
| 36 | aristotle univ thessaloniki | 59 | 40 | 1675 |
| 37 | hebrew univ jerusalem | 58 | 10 | 763 |
| 38 | oslo univ hosp | 58 | 11 | 661 |
| 39 | steno diabet ctr copenhagen | 58 | 10 | 1052 |
| 40 | yale sch med | 58 | 15 | 1200 |
| 41 | univ oslo | 56 | 11 | 652 |
| 42 | mt sinai hosp | 55 | 13 | 188 |
| 43 | univ glasgow | 53 | 19 | 578 |
| 44 | wurzburg univ clin | 51 | 12 | 393 |
| 45 | univ colorado | 50 | 22 | 527 |
| 46 | boehringer ingelheim norway ks | 47 | 13 | 805 |
| 47 | astrazeneca gothenburg | 44 | 13 | 1471 |
| 48 | nyu | 44 | 13 | 670 |
| 49 | kyoto univ | 40 | 11 | 1557 |
| 50 | univ mississippi | 40 | 20 | 324 |
| 51 | kings coll london | 36 | 10 | 1037 |
| 52 | georgetown univ | 35 | 12 | 445 |
| 53 | boehringer ingelheim pharmaceut inc | 34 | 17 | 696 |
| 54 | st michaels hosp | 34 | 16 | 209 |
| 55 | lmc diabet & endocrinol | 33 | 11 | 56 |
| 56 | george washington univ | 29 | 17 | 172 |
| 57 | uppsala univ | 27 | 10 | 296 |
| 58 | mcgill univ | 25 | 15 | 149 |
| 59 | univ alberta | 25 | 14 | 67 |
| 60 | univ calif san diego | 25 | 26 | 1040 |
| 61 | monash univ | 24 | 20 | 229 |
| 62 | emory univ | 21 | 10 | 174 |
| 63 | baylor univ | 19 | 20 | 249 |
| 64 | boehringer ingelheim gmbh & co kg | 18 | 11 | 176 |
| 65 | va san diego healthcare syst | 16 | 14 | 641 |
| 66 | chu liege | 14 | 14 | 319 |
| 67 | janssen sci affairs llc | 14 | 13 | 242 |
| 68 | univ athens | 14 | 14 | 209 |
| 69 | univ liege | 14 | 32 | 887 |
| 70 | univ texas hlth sci ctr san antonio | 13 | 18 | 772 |
| 71 | univ tokyo | 12 | 12 | 109 |
| 72 | swansea univ | 11 | 12 | 182 |
| 73 | tulane univ | 8 | 11 | 76 |
| 74 | jikei univ | 7 | 12 | 185 |
| 75 | univ maryland | 7 | 17 | 149 |
| 76 | kawasaki med sch | 6 | 10 | 97 |
| 77 | univ padua | 6 | 19 | 263 |
| 78 | univ pisa | 6 | 12 | 390 |
| 79 | seoul natl univ | 3 | 13 | 182 |
| 80 | astellas pharma inc | 1 | 12 | 72 |
| 81 | univ ioannina | 1 | 11 | 89 |
