# Supplementary material for: Bibliometric Study of Sodium Glucose Cotransporter 2 Inhibitors in Cardiovascular Research
Source: Front Pharmacol. 2020 Sep 15;11:561494. doi: 10.3389/fphar.2020.561494 (PMC7522576; doi:10.3389/fphar.2020.561494)
Supplement: Supplementary file 5 [file Table_5.docx]

Supplementary Material

**
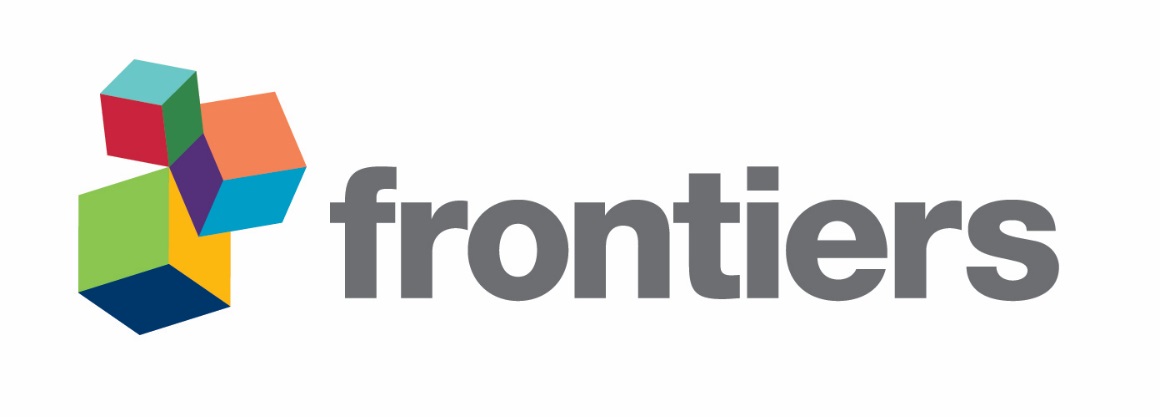
**

**Supplementary Table 5.** The journals publishing articles of SGLT2 inhibitors in CV research.

| **Rank** | **Journal title** | **Count (N)** | **Percentage (N/1509)** |
| --- | --- | --- | --- |
| 1 | DIABETES OBESITY METABOLISM | 112 | 7.422 |
| 2 | CARDIOVASCULAR DIABETOLOGY | 87 | 5.765 |
| 3 | DIABETES THERAPY | 51 | 3.38 |
| 4 | DIABETES CARE | 36 | 2.386 |
| 5 | CIRCULATION | 27 | 1.789 |
| 6 | DIABETOLOGIA | 26 | 1.723 |
| 7 | POSTGRADUATE MEDICINE | 24 | 1.59 |
| 8 | CURRENT DIABETES REPORTS | 23 | 1.524 |
| 9 | DIABETES RESEARCH AND CLINICAL PRACTICE | 22 | 1.458 |
| 10 | EXPERT OPINION ON PHARMACOTHERAPY | 21 | 1.392 |
| 11 | EUROPEAN JOURNAL OF HEART FAILURE | 18 | 1.193 |
| 12 | EXPERT OPINION ON DRUG SAFETY | 17 | 1.127 |
| 13 | AMERICAN JOURNAL OF MEDICINE | 16 | 1.06 |
| 14 | HEART FAILURE REVIEWS | 16 | 1.06 |
| 15 | LANCET DIABETES ENDOCRINOLOGY | 16 | 1.06 |
| 16 | AMERICAN JOURNAL OF CARDIOLOGY | 15 | 0.994 |
| 17 | DIABETES METABOLISM | 15 | 0.994 |
| 18 | DIABETIC MEDICINE | 15 | 0.994 |
| 19 | CARDIOVASCULAR DRUGS AND THERAPY | 14 | 0.928 |
| 20 | CURRENT MEDICAL RESEARCH AND OPINION | 14 | 0.928 |
| 21 | INTERNATIONAL JOURNAL OF MOLECULAR SCIENCES | 14 | 0.928 |
| 22 | ADVANCES IN THERAPY | 13 | 0.861 |
| 23 | DIABETES METABOLIC SYNDROME AND OBESITY TARGETS AND THERAPY | 13 | 0.861 |
| 24 | JOURNAL OF THE AMERICAN COLLEGE OF CARDIOLOGY | 13 | 0.861 |
| 25 | SCIENTIFIC REPORTS | 13 | 0.861 |
| 26 | CURRENT CARDIOLOGY REPORTS | 12 | 0.795 |
| 27 | DRUGS | 12 | 0.795 |
| 28 | EXPERT OPINION ON DRUG METABOLISM TOXICOLOGY | 12 | 0.795 |
| 29 | EXPERT REVIEW OF CLINICAL PHARMACOLOGY | 12 | 0.795 |
| 30 | JOURNAL OF DIABETES INVESTIGATION | 12 | 0.795 |
| 31 | NEPHROLOGY DIALYSIS TRANSPLANTATION | 12 | 0.795 |
| 32 | DIABETES VASCULAR DISEASE RESEARCH | 11 | 0.729 |
| 33 | JOURNAL OF DIABETES AND ITS COMPLICATIONS | 11 | 0.729 |
| 34 | JOURNAL OF THE AMERICAN HEART ASSOCIATION | 10 | 0.663 |
| 35 | PLOS ONE | 10 | 0.663 |
| 36 | ANNALS OF PHARMACOTHERAPY | 9 | 0.596 |
| 37 | CLINICAL DRUG INVESTIGATION | 9 | 0.596 |
| 38 | CURRENT OPINION IN CARDIOLOGY | 9 | 0.596 |
| 39 | DIABETOLOGY METABOLIC SYNDROME | 9 | 0.596 |
| 40 | CANADIAN JOURNAL OF DIABETES | 8 | 0.53 |
| 41 | CLINICAL THERAPEUTICS | 8 | 0.53 |
| 42 | CURRENT VASCULAR PHARMACOLOGY | 8 | 0.53 |
| 43 | DIABETES METABOLISM RESEARCH AND REVIEWS | 8 | 0.53 |
| 44 | CURRENT OPINION IN NEPHROLOGY AND HYPERTENSION | 7 | 0.464 |
| 45 | ESC HEART FAILURE | 7 | 0.464 |
| 46 | EUROPEAN HEART JOURNAL | 7 | 0.464 |
| 47 | JOURNAL OF CLINICAL ENDOCRINOLOGY METABOLISM | 7 | 0.464 |
| 48 | JOURNAL OF CLINICAL MEDICINE | 7 | 0.464 |
| 49 | JOURNAL OF THE AMERICAN SOCIETY OF NEPHROLOGY | 7 | 0.464 |
| 50 | WORLD JOURNAL OF DIABETES | 7 | 0.464 |
| 51 | AMERICAN HEART JOURNAL | 6 | 0.398 |
| 52 | AMERICAN JOURNAL OF PHYSIOLOGY RENAL PHYSIOLOGY | 6 | 0.398 |
| 53 | BMJ OPEN | 6 | 0.398 |
| 54 | CARDIOVASCULAR RESEARCH | 6 | 0.398 |
| 55 | CLINICAL SCIENCE | 6 | 0.398 |
| 56 | CURRENT PHARMACEUTICAL DESIGN | 6 | 0.398 |
| 57 | HYPERTENSION RESEARCH | 6 | 0.398 |
| 58 | INTERNATIONAL JOURNAL OF CLINICAL PRACTICE | 6 | 0.398 |
| 59 | JACC HEART FAILURE | 6 | 0.398 |
| 60 | KIDNEY INTERNATIONAL | 6 | 0.398 |
| 61 | NEW ENGLAND JOURNAL OF MEDICINE | 6 | 0.398 |
| 62 | NUTRITION METABOLISM AND CARDIOVASCULAR DISEASES | 6 | 0.398 |
| 63 | REVISTA DA ASSOCIACAO MEDICA BRASILEIRA | 6 | 0.398 |
| 64 | THERAPEUTICS AND CLINICAL RISK MANAGEMENT | 6 | 0.398 |
| 65 | AMERICAN JOURNAL OF CARDIOVASCULAR DRUGS | 5 | 0.331 |
| 66 | ARTERIOSCLEROSIS THROMBOSIS AND VASCULAR BIOLOGY | 5 | 0.331 |
| 67 | CLINICAL CARDIOLOGY | 5 | 0.331 |
| 68 | DIABETES METABOLISM JOURNAL | 5 | 0.331 |
| 69 | ENDOCRINE JOURNAL | 5 | 0.331 |
| 70 | EUROPEAN JOURNAL OF PREVENTIVE CARDIOLOGY | 5 | 0.331 |
| 71 | FRONTIERS IN ENDOCRINOLOGY | 5 | 0.331 |
| 72 | FRONTIERS IN PHYSIOLOGY | 5 | 0.331 |
| 73 | INTERNATIONAL JOURNAL OF CARDIOLOGY | 5 | 0.331 |
| 74 | JOURNAL OF THE AMERICAN SOCIETY OF HYPERTENSION | 5 | 0.331 |
| 75 | THERAPEUTIC ADVANCES IN ENDOCRINOLOGY AND METABOLISM | 5 | 0.331 |
| 76 | AMERICAN JOURNAL OF HYPERTENSION | 4 | 0.265 |
| 77 | BIOMEDICINE PHARMACOTHERAPY | 4 | 0.265 |
| 78 | BMJ BRITISH MEDICAL JOURNAL | 4 | 0.265 |
| 79 | BMJ OPEN DIABETES RESEARCH CARE | 4 | 0.265 |
| 80 | CLINICAL KIDNEY JOURNAL | 4 | 0.265 |
| 81 | CURRENT DRUG TARGETS | 4 | 0.265 |
| 82 | CURRENT HYPERTENSION REPORTS | 4 | 0.265 |
| 83 | CURRENT MEDICINAL CHEMISTRY | 4 | 0.265 |
| 84 | DIABETES | 4 | 0.265 |
| 85 | DIABETES TECHNOLOGY THERAPEUTICS | 4 | 0.265 |
| 86 | DRUGS OF TODAY | 4 | 0.265 |
| 87 | ENDOCRINE | 4 | 0.265 |
| 88 | ENDOCRINE PRACTICE | 4 | 0.265 |
| 89 | EUROPEAN JOURNAL OF PHARMACOLOGY | 4 | 0.265 |
| 90 | EXPERT OPINION ON INVESTIGATIONAL DRUGS | 4 | 0.265 |
| 91 | INTERNAL MEDICINE | 4 | 0.265 |
| 92 | JOURNAL OF FAMILY PRACTICE | 4 | 0.265 |
| 93 | JOURNAL OF MEDICAL ECONOMICS | 4 | 0.265 |
| 94 | PROGRESS IN CARDIOVASCULAR DISEASES | 4 | 0.265 |
| 95 | ACTA DIABETOLOGICA | 3 | 0.199 |
| 96 | AMERICAN JOURNAL OF MANAGED CARE | 3 | 0.199 |
| 97 | AMERICAN JOURNAL OF NEPHROLOGY | 3 | 0.199 |
| 98 | ANNALS OF INTERNAL MEDICINE | 3 | 0.199 |
| 99 | ANNALS OF MEDICINE | 3 | 0.199 |
| 100 | ATHEROSCLEROSIS | 3 | 0.199 |
| 101 | CANADIAN FAMILY PHYSICIAN | 3 | 0.199 |
| 102 | CIRCULATION JOURNAL | 3 | 0.199 |
| 103 | CLINICAL JOURNAL OF THE AMERICAN SOCIETY OF NEPHROLOGY | 3 | 0.199 |
| 104 | CLINICAL PHARMACOKINETICS | 3 | 0.199 |
| 105 | CURRENT ATHEROSCLEROSIS REPORTS | 3 | 0.199 |
| 106 | CURRENT OPINION IN ENDOCRINOLOGY DIABETES AND OBESITY | 3 | 0.199 |
| 107 | DRUG DESIGN DEVELOPMENT AND THERAPY | 3 | 0.199 |
| 108 | ENDOCRINE METABOLIC IMMUNE DISORDERS DRUG TARGETS | 3 | 0.199 |
| 109 | ENDOCRINOLOGY AND METABOLISM | 3 | 0.199 |
| 110 | ENDOKRYNOLOGIA POLSKA | 3 | 0.199 |
| 111 | EUROPEAN JOURNAL OF CLINICAL PHARMACOLOGY | 3 | 0.199 |
| 112 | FRONTIERS IN PHARMACOLOGY | 3 | 0.199 |
| 113 | FUTURE MEDICINAL CHEMISTRY | 3 | 0.199 |
| 114 | HEART FAILURE CLINICS | 3 | 0.199 |
| 115 | INTERNATIONAL HEART JOURNAL | 3 | 0.199 |
| 116 | INTERNATIONAL JOURNAL OF ENDOCRINOLOGY | 3 | 0.199 |
| 117 | JOURNAL OF CELLULAR PHYSIOLOGY | 3 | 0.199 |
| 118 | JOURNAL OF DIABETES | 3 | 0.199 |
| 119 | JOURNAL OF DIABETES RESEARCH | 3 | 0.199 |
| 120 | JOURNAL OF HYPERTENSION | 3 | 0.199 |
| 121 | JOURNAL OF MANAGED CARE SPECIALTY PHARMACY | 3 | 0.199 |
| 122 | JOURNAL OF PHARMACOLOGY AND EXPERIMENTAL THERAPEUTICS | 3 | 0.199 |
| 123 | KIDNEY BLOOD PRESSURE RESEARCH | 3 | 0.199 |
| 124 | MEDICAL HYPOTHESES | 3 | 0.199 |
| 125 | MEDICINA LITHUANIA | 3 | 0.199 |
| 126 | METABOLISM CLINICAL AND EXPERIMENTAL | 3 | 0.199 |
| 127 | MOLECULAR AND CELLULAR BIOCHEMISTRY | 3 | 0.199 |
| 128 | NAUNYN SCHMIEDEBERGS ARCHIVES OF PHARMACOLOGY | 3 | 0.199 |
| 129 | PHARMACOTHERAPY | 3 | 0.199 |
| 130 | PRIMARY CARE DIABETES | 3 | 0.199 |
| 131 | REDOX BIOLOGY | 3 | 0.199 |
| 132 | TRIALS | 3 | 0.199 |
| 133 | ADVANCES IN EXPERIMENTAL MEDICINE AND BIOLOGY | 2 | 0.133 |
| 134 | AMERICAN JOURNAL OF HEALTH SYSTEM PHARMACY | 2 | 0.133 |
| 135 | AMERICAN JOURNAL OF KIDNEY DISEASES | 2 | 0.133 |
| 136 | AMERICAN JOURNAL OF PHYSIOLOGY ENDOCRINOLOGY AND METABOLISM | 2 | 0.133 |
| 137 | ANNALS OF TRANSLATIONAL MEDICINE | 2 | 0.133 |
| 138 | ANNUAL REVIEW OF MEDICINE | 2 | 0.133 |
| 139 | ARCHIVES OF PHARMACAL RESEARCH | 2 | 0.133 |
| 140 | BIOCHIMICA ET BIOPHYSICA ACTA MOLECULAR BASIS OF DISEASE | 2 | 0.133 |
| 141 | BIOMEDICINES | 2 | 0.133 |
| 142 | BIOORGANIC MEDICINAL CHEMISTRY | 2 | 0.133 |
| 143 | BIOSCIENCE REPORTS | 2 | 0.133 |
| 144 | BMC CARDIOVASCULAR DISORDERS | 2 | 0.133 |
| 145 | BRITISH JOURNAL OF CLINICAL PHARMACOLOGY | 2 | 0.133 |
| 146 | CANADIAN JOURNAL OF CARDIOLOGY | 2 | 0.133 |
| 147 | CANADIAN JOURNAL OF PHYSIOLOGY AND PHARMACOLOGY | 2 | 0.133 |
| 148 | CARDIOLOGY | 2 | 0.133 |
| 149 | CARDIOLOGY IN REVIEW | 2 | 0.133 |
| 150 | CELL METABOLISM | 2 | 0.133 |
| 151 | CIRCULATION HEART FAILURE | 2 | 0.133 |
| 152 | CIRCULATION RESEARCH | 2 | 0.133 |
| 153 | CLEVELAND CLINIC JOURNAL OF MEDICINE | 2 | 0.133 |
| 154 | CLINICAL AND EXPERIMENTAL PHARMACOLOGY AND PHYSIOLOGY | 2 | 0.133 |
| 155 | CLINICAL PHARMACOLOGY IN DRUG DEVELOPMENT | 2 | 0.133 |
| 156 | CLINICAL PHARMACOLOGY THERAPEUTICS | 2 | 0.133 |
| 157 | CLINICAL RESEARCH IN CARDIOLOGY | 2 | 0.133 |
| 158 | COCHRANE DATABASE OF SYSTEMATIC REVIEWS | 2 | 0.133 |
| 159 | CURRENT OPINION IN ANESTHESIOLOGY | 2 | 0.133 |
| 160 | CURRENT PROBLEMS IN CARDIOLOGY | 2 | 0.133 |
| 161 | DRUG SAFETY | 2 | 0.133 |
| 162 | DRUGS AGING | 2 | 0.133 |
| 163 | EXPERT OPINION ON DRUG DISCOVERY | 2 | 0.133 |
| 164 | EXPERT OPINION ON THERAPEUTIC PATENTS | 2 | 0.133 |
| 165 | FUNDAMENTAL CLINICAL PHARMACOLOGY | 2 | 0.133 |
| 166 | HEART FAILURE FROM RESEARCH TO CLINICAL PRACTICE VOL 3 | 2 | 0.133 |
| 167 | HEART LUNG AND CIRCULATION | 2 | 0.133 |
| 168 | HEPATOLOGY RESEARCH | 2 | 0.133 |
| 169 | HERZ | 2 | 0.133 |
| 170 | HORMONES INTERNATIONAL JOURNAL OF ENDOCRINOLOGY AND METABOLISM | 2 | 0.133 |
| 171 | HYPERTENSION | 2 | 0.133 |
| 172 | INTERNAL MEDICINE JOURNAL | 2 | 0.133 |
| 173 | INTERNATIONAL JOURNAL OF ENVIRONMENTAL RESEARCH AND PUBLIC HEALTH | 2 | 0.133 |
| 174 | JAAPA JOURNAL OF THE AMERICAN ACADEMY OF PHYSICIAN ASSISTANTS | 2 | 0.133 |
| 175 | JAMA JOURNAL OF THE AMERICAN MEDICAL ASSOCIATION | 2 | 0.133 |
| 176 | JAMA NETWORK OPEN | 2 | 0.133 |
| 177 | JCI INSIGHT | 2 | 0.133 |
| 178 | JOURNAL OF CARDIOLOGY | 2 | 0.133 |
| 179 | JOURNAL OF CLINICAL HYPERTENSION | 2 | 0.133 |
| 180 | JOURNAL OF CLINICAL LIPIDOLOGY | 2 | 0.133 |
| 181 | JOURNAL OF CLINICAL PHARMACOLOGY | 2 | 0.133 |
| 182 | JOURNAL OF ENDOCRINOLOGICAL INVESTIGATION | 2 | 0.133 |
| 183 | JOURNAL OF ENDOCRINOLOGY | 2 | 0.133 |
| 184 | JOURNAL OF NEPHROLOGY | 2 | 0.133 |
| 185 | JOURNAL OF THE CHINESE MEDICAL ASSOCIATION | 2 | 0.133 |
| 186 | JOURNAL OF THE PAKISTAN MEDICAL ASSOCIATION | 2 | 0.133 |
| 187 | KIDNEY INTERNATIONAL SUPPLEMENTS | 2 | 0.133 |
| 188 | KOREAN JOURNAL OF INTERNAL MEDICINE | 2 | 0.133 |
| 189 | MEDICAL DECISION MAKING | 2 | 0.133 |
| 190 | MEDICAL JOURNAL OF AUSTRALIA | 2 | 0.133 |
| 191 | MOLECULAR AND CELLULAR ENDOCRINOLOGY | 2 | 0.133 |
| 192 | MOLECULAR NUTRITION FOOD RESEARCH | 2 | 0.133 |
| 193 | NATURE REVIEWS ENDOCRINOLOGY | 2 | 0.133 |
| 194 | NUTRIENTS | 2 | 0.133 |
| 195 | PHARMACOLOGY THERAPEUTICS | 2 | 0.133 |
| 196 | REVIEWS IN CARDIOVASCULAR MEDICINE | 2 | 0.133 |
| 197 | REVIEWS IN ENDOCRINE METABOLIC DISORDERS | 2 | 0.133 |
| 198 | REVISTA ESPANOLA DE CARDIOLOGIA | 2 | 0.133 |
| 199 | SAUDI PHARMACEUTICAL JOURNAL | 2 | 0.133 |
| 200 | STROKE | 2 | 0.133 |
| 201 | TRENDS IN CARDIOVASCULAR MEDICINE | 2 | 0.133 |
| 202 | TRENDS IN ENDOCRINOLOGY AND METABOLISM | 2 | 0.133 |
| 203 | ACTA CARDIOLOGICA | 1 | 0.066 |
| 204 | ACTA PHARMACOLOGICA SINICA | 1 | 0.066 |
| 205 | ADVANCES IN CHRONIC KIDNEY DISEASE | 1 | 0.066 |
| 206 | AGE AND AGEING | 1 | 0.066 |
| 207 | AGING MALE | 1 | 0.066 |
| 208 | ALZHEIMERS RESEARCH THERAPY | 1 | 0.066 |
| 209 | AMERICAN FAMILY PHYSICIAN | 1 | 0.066 |
| 210 | AMERICAN JOURNAL OF PHYSIOLOGY CELL PHYSIOLOGY | 1 | 0.066 |
| 211 | AMERICAN JOURNAL OF PHYSIOLOGY HEART AND CIRCULATORY PHYSIOLOGY | 1 | 0.066 |
| 212 | AMERICAN JOURNAL OF PHYSIOLOGY LUNG CELLULAR AND MOLECULAR PHYSIOLOGY | 1 | 0.066 |
| 213 | AMERICAN JOURNAL OF THE MEDICAL SCIENCES | 1 | 0.066 |
| 214 | AMERICAN JOURNAL OF THERAPEUTICS | 1 | 0.066 |
| 215 | ANNALS OF VASCULAR SURGERY | 1 | 0.066 |
| 216 | ANNUAL REVIEW OF MEDICINE VOL 66 | 1 | 0.066 |
| 217 | ANNUAL REVIEW OF MEDICINE VOL 70 | 1 | 0.066 |
| 218 | ARCHIVES OF MEDICAL RESEARCH | 1 | 0.066 |
| 219 | BASIC CLINICAL PHARMACOLOGY TOXICOLOGY | 1 | 0.066 |
| 220 | BASIC RESEARCH IN CARDIOLOGY | 1 | 0.066 |
| 221 | BEST PRACTICE RESEARCH CLINICAL ENDOCRINOLOGY METABOLISM | 1 | 0.066 |
| 222 | BIOCHEMICAL AND BIOPHYSICAL RESEARCH COMMUNICATIONS | 1 | 0.066 |
| 223 | BIOCHEMICAL PHARMACOLOGY | 1 | 0.066 |
| 224 | BIOCHIMIE | 1 | 0.066 |
| 225 | BIOLOGICAL CYBERNETICS | 1 | 0.066 |
| 226 | BIOLOGICAL PHARMACEUTICAL BULLETIN | 1 | 0.066 |
| 227 | BIOMED RESEARCH INTERNATIONAL | 1 | 0.066 |
| 228 | BLOOD PRESSURE | 1 | 0.066 |
| 229 | BMC FAMILY PRACTICE | 1 | 0.066 |
| 230 | BMC GERIATRICS | 1 | 0.066 |
| 231 | BMC HEALTH SERVICES RESEARCH | 1 | 0.066 |
| 232 | BMC MEDICINE | 1 | 0.066 |
| 233 | BONE | 1 | 0.066 |
| 234 | BRITISH MEDICAL BULLETIN | 1 | 0.066 |
| 235 | CANADIAN JOURNAL OF ANESTHESIA JOURNAL CANADIEN D ANESTHESIE | 1 | 0.066 |
| 236 | CARDIOLOGY CLINICS | 1 | 0.066 |
| 237 | CARDIORENAL MEDICINE | 1 | 0.066 |
| 238 | CARDIOVASCULAR DIAGNOSIS AND THERAPY | 1 | 0.066 |
| 239 | CARDIOVASCULAR JOURNAL OF AFRICA | 1 | 0.066 |
| 240 | CARDIOVASCULAR ULTRASOUND | 1 | 0.066 |
| 241 | CELLS | 1 | 0.066 |
| 242 | CELLULAR PHYSIOLOGY AND BIOCHEMISTRY | 1 | 0.066 |
| 243 | CELLULAR SIGNALLING | 1 | 0.066 |
| 244 | CLINICAL AND EXPERIMENTAL HYPERTENSION | 1 | 0.066 |
| 245 | CLINICAL AND EXPERIMENTAL NEPHROLOGY | 1 | 0.066 |
| 246 | CLINICAL AND INVESTIGATIVE MEDICINE | 1 | 0.066 |
| 247 | CLINICAL ENDOCRINOLOGY | 1 | 0.066 |
| 248 | CLINICAL EPIDEMIOLOGY | 1 | 0.066 |
| 249 | CLINICAL PHYSIOLOGY AND FUNCTIONAL IMAGING | 1 | 0.066 |
| 250 | CLINICAL TOXICOLOGY | 1 | 0.066 |
| 251 | CRITICAL CARE CLINICS | 1 | 0.066 |
| 252 | CURRENT DRUG METABOLISM | 1 | 0.066 |
| 253 | CURRENT OPINION IN LIPIDOLOGY | 1 | 0.066 |
| 254 | CURRENT OPINION IN PHARMACOLOGY | 1 | 0.066 |
| 255 | CURRENT OSTEOPOROSIS REPORTS | 1 | 0.066 |
| 256 | CURRENT TOPICS IN MEDICINAL CHEMISTRY | 1 | 0.066 |
| 257 | EBIOMEDICINE | 1 | 0.066 |
| 258 | EMBO MOLECULAR MEDICINE | 1 | 0.066 |
| 259 | ENDOCRINOLOGY AND METABOLISM CLINICS OF NORTH AMERICA | 1 | 0.066 |
| 260 | EUROPEAN HEART JOURNAL CARDIOVASCULAR PHARMACOTHERAPY | 1 | 0.066 |
| 261 | EUROPEAN HEART JOURNAL SUPPLEMENTS | 1 | 0.066 |
| 262 | EUROPEAN JOURNAL OF CLINICAL INVESTIGATION | 1 | 0.066 |
| 263 | EUROPEAN JOURNAL OF ENDOCRINOLOGY | 1 | 0.066 |
| 264 | EUROPEAN JOURNAL OF INTERNAL MEDICINE | 1 | 0.066 |
| 265 | EUROPEAN JOURNAL OF MEDICINAL CHEMISTRY | 1 | 0.066 |
| 266 | EUROPEAN JOURNAL OF PHARMACEUTICAL SCIENCES | 1 | 0.066 |
| 267 | EXPERT OPINION ON EMERGING DRUGS | 1 | 0.066 |
| 268 | EXPERT OPINION ON THERAPEUTIC TARGETS | 1 | 0.066 |
| 269 | EXPERT REVIEW OF PHARMACOECONOMICS OUTCOMES RESEARCH | 1 | 0.066 |
| 270 | FREE RADICAL BIOLOGY AND MEDICINE | 1 | 0.066 |
| 271 | FRONTIERS IN CARDIOVASCULAR MEDICINE | 1 | 0.066 |
| 272 | FRONTIERS IN NEUROSCIENCE | 1 | 0.066 |
| 273 | HEALTH TECHNOLOGY ASSESSMENT | 1 | 0.066 |
| 274 | HEART | 1 | 0.066 |
| 275 | HEPATOLOGY | 1 | 0.066 |
| 276 | HONG KONG MEDICAL JOURNAL | 1 | 0.066 |
| 277 | HORMONE AND METABOLIC RESEARCH | 1 | 0.066 |
| 278 | INTERNAL AND EMERGENCY MEDICINE | 1 | 0.066 |
| 279 | INTERNATIONAL JOURNAL OF BIOCHEMISTRY CELL BIOLOGY | 1 | 0.066 |
| 280 | INTERNATIONAL JOURNAL OF CLINICAL PHARMACOLOGY AND THERAPEUTICS | 1 | 0.066 |
| 281 | INTERNATIONAL JOURNAL OF GENERAL MEDICINE | 1 | 0.066 |
| 282 | INTERNATIONAL JOURNAL OF TOXICOLOGY | 1 | 0.066 |
| 283 | INTERNATIONAL UROLOGY AND NEPHROLOGY | 1 | 0.066 |
| 284 | ISRAEL MEDICAL ASSOCIATION JOURNAL | 1 | 0.066 |
| 285 | JAMA CARDIOLOGY | 1 | 0.066 |
| 286 | JOURNAL OF BACTERIOLOGY | 1 | 0.066 |
| 287 | JOURNAL OF BONE AND MINERAL RESEARCH | 1 | 0.066 |
| 288 | JOURNAL OF CARDIAC FAILURE | 1 | 0.066 |
| 289 | JOURNAL OF CARDIOVASCULAR MEDICINE | 1 | 0.066 |
| 290 | JOURNAL OF CARDIOVASCULAR NURSING | 1 | 0.066 |
| 291 | JOURNAL OF CARDIOVASCULAR PHARMACOLOGY AND THERAPEUTICS | 1 | 0.066 |
| 292 | JOURNAL OF CELLULAR AND MOLECULAR MEDICINE | 1 | 0.066 |
| 293 | JOURNAL OF CLINICAL INVESTIGATION | 1 | 0.066 |
| 294 | JOURNAL OF CLINICAL PHARMACY AND THERAPEUTICS | 1 | 0.066 |
| 295 | JOURNAL OF EMERGENCY MEDICINE | 1 | 0.066 |
| 296 | JOURNAL OF EVALUATION IN CLINICAL PRACTICE | 1 | 0.066 |
| 297 | JOURNAL OF FUNCTIONAL FOODS | 1 | 0.066 |
| 298 | JOURNAL OF GASTROENTEROLOGY | 1 | 0.066 |
| 299 | JOURNAL OF GENERAL INTERNAL MEDICINE | 1 | 0.066 |
| 300 | JOURNAL OF HUMAN HYPERTENSION | 1 | 0.066 |
| 301 | JOURNAL OF INTERNAL MEDICINE | 1 | 0.066 |
| 302 | JOURNAL OF MOLECULAR AND CELLULAR CARDIOLOGY | 1 | 0.066 |
| 303 | JOURNAL OF NATURAL PRODUCTS | 1 | 0.066 |
| 304 | JOURNAL OF NEUROLOGY NEUROSURGERY AND PSYCHIATRY | 1 | 0.066 |
| 305 | JOURNAL OF PHARMACOLOGICAL AND TOXICOLOGICAL METHODS | 1 | 0.066 |
| 306 | JOURNAL OF PHARMACOLOGICAL SCIENCES | 1 | 0.066 |
| 307 | JOURNAL OF SEXUAL MEDICINE | 1 | 0.066 |
| 308 | JOURNAL OF STROKE | 1 | 0.066 |
| 309 | JOURNAL OF THE AMERICAN GERIATRICS SOCIETY | 1 | 0.066 |
| 310 | JOURNAL OF THORACIC DISEASE | 1 | 0.066 |
| 311 | JOURNAL OF TRANSLATIONAL MEDICINE | 1 | 0.066 |
| 312 | JOURNAL OF VASCULAR SURGERY | 1 | 0.066 |
| 313 | KARDIOLOGIA POLSKA | 1 | 0.066 |
| 314 | KARDIOLOGIYA | 1 | 0.066 |
| 315 | KOREAN CIRCULATION JOURNAL | 1 | 0.066 |
| 316 | KOREAN JOURNAL OF PHYSIOLOGY PHARMACOLOGY | 1 | 0.066 |
| 317 | LANCET | 1 | 0.066 |
| 318 | LIFE SCIENCES | 1 | 0.066 |
| 319 | LIPIDS IN HEALTH AND DISEASE | 1 | 0.066 |
| 320 | LIVER INTERNATIONAL | 1 | 0.066 |
| 321 | MATURITAS | 1 | 0.066 |
| 322 | MEDICAL CLINICS OF NORTH AMERICA | 1 | 0.066 |
| 323 | MEDICAL LETTER ON DRUGS AND THERAPEUTICS | 1 | 0.066 |
| 324 | MEDICAL SCIENCE MONITOR | 1 | 0.066 |
| 325 | MEDICINE | 1 | 0.066 |
| 326 | METABOLIC SYNDROME AND RELATED DISORDERS | 1 | 0.066 |
| 327 | MINERVA ENDOCRINOLOGICA | 1 | 0.066 |
| 328 | MOLECULAR BIOSYSTEMS | 1 | 0.066 |
| 329 | MOLECULAR MEDICINE REPORTS | 1 | 0.066 |
| 330 | NATURAL PRODUCT REPORTS | 1 | 0.066 |
| 331 | NATURE COMMUNICATIONS | 1 | 0.066 |
| 332 | NATURE REVIEWS CARDIOLOGY | 1 | 0.066 |
| 333 | NATURE REVIEWS NEPHROLOGY | 1 | 0.066 |
| 334 | NEPHRON | 1 | 0.066 |
| 335 | NUTRITION | 1 | 0.066 |
| 336 | NUTRITION DIABETES | 1 | 0.066 |
| 337 | OBESITY | 1 | 0.066 |
| 338 | OXIDATIVE MEDICINE AND CELLULAR LONGEVITY | 1 | 0.066 |
| 339 | PATIENT PREFERENCE AND ADHERENCE | 1 | 0.066 |
| 340 | PFLUGERS ARCHIV EUROPEAN JOURNAL OF PHYSIOLOGY | 1 | 0.066 |
| 341 | PHARMACOECONOMICS | 1 | 0.066 |
| 342 | PHARMACOLOGICAL RESEARCH | 1 | 0.066 |
| 343 | PHYSICIAN AND SPORTSMEDICINE | 1 | 0.066 |
| 344 | PHYSIOLOGICAL RESEARCH | 1 | 0.066 |
| 345 | PHYSIOLOGY | 1 | 0.066 |
| 346 | PHYTOTHERAPY RESEARCH | 1 | 0.066 |
| 347 | PLOS MEDICINE | 1 | 0.066 |
| 348 | POLISH ARCHIVES OF INTERNAL MEDICINE POLSKIE ARCHIWUM MEDYCYNY WEWNETRZNEJ | 1 | 0.066 |
| 349 | POSTGRADUATE MEDICAL JOURNAL | 1 | 0.066 |
| 350 | PROCEEDINGS OF THE NUTRITION SOCIETY | 1 | 0.066 |
| 351 | REJUVENATION RESEARCH | 1 | 0.066 |
| 352 | RHEUMATOLOGY | 1 | 0.066 |
| 353 | RSC ADVANCES | 1 | 0.066 |
| 354 | SEMINARS IN THROMBOSIS AND HEMOSTASIS | 1 | 0.066 |
| 355 | SINGAPORE MEDICAL JOURNAL | 1 | 0.066 |
| 356 | SPECTROCHIMICA ACTA PART A MOLECULAR AND BIOMOLECULAR SPECTROSCOPY | 1 | 0.066 |
| 357 | SURGERY FOR OBESITY AND RELATED DISEASES | 1 | 0.066 |
| 358 | SWISS MEDICAL WEEKLY | 1 | 0.066 |
| 359 | THERAPEUTIC ADVANCES IN CHRONIC DISEASE | 1 | 0.066 |
| 360 | TRANSPLANTATION PROCEEDINGS | 1 | 0.066 |
| 361 | UPSALA JOURNAL OF MEDICAL SCIENCES | 1 | 0.066 |
| 362 | VALUE IN HEALTH | 1 | 0.066 |
| 363 | VASCULAR PHARMACOLOGY | 1 | 0.066 |
| 364 | WORLD JOURNAL OF GASTROENTEROLOGY | 1 | 0.066 |

**Note:** SGLT2: Sodium Glucose Cotransporter 2. CV: cardiovascular.
