# Supplementary material for: Bibliometric Study of Sodium Glucose Cotransporter 2 Inhibitors in Cardiovascular Research
Source: Front Pharmacol. 2020 Sep 15;11:561494. doi: 10.3389/fphar.2020.561494 (PMC7522576; doi:10.3389/fphar.2020.561494)
Supplement: Supplementary file 6 [file Table_6.docx]

Supplementary Material

**
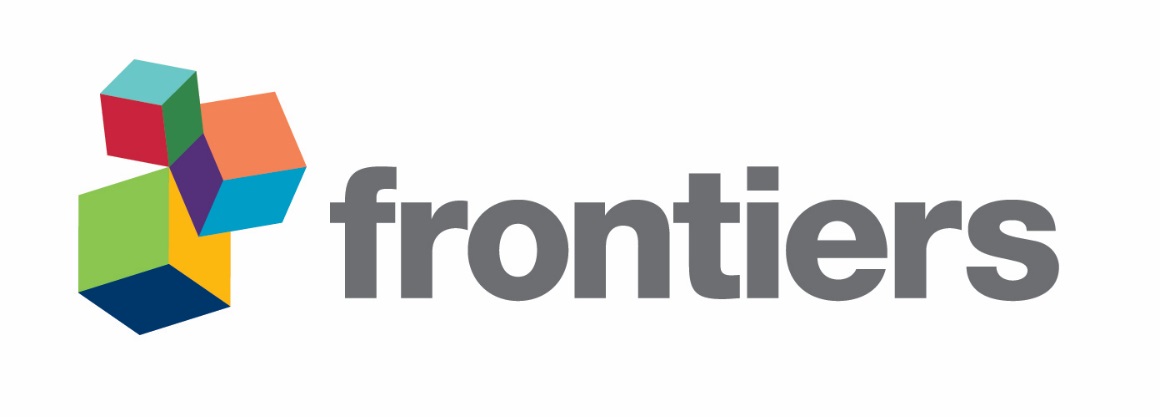
**

**Supplementary Table 6.** The cocited journals related to SGLT2 inhibitors in CV research.

| **Rank** | **Journal** | **Count** |
| --- | --- | --- |
| 1 | new engl j med | 8400 |
| 2 | diabetes care | 7534 |
| 3 | diabetes obes metab | 5105 |
| 4 | circulation | 3469 |
| 5 | lancet | 2977 |
| 6 | diabetologia | 2285 |
| 7 | diabetes | 1872 |
| 8 | lancet diabetes endo | 1852 |
| 9 | cardiovasc diabetol | 1848 |
| 10 | j am coll cardiol | 1364 |
| 11 | eur heart j | 1299 |
| 12 | jama-j am med assoc | 1268 |
| 13 | kidney int | 1101 |
| 14 | j clin endocr metab | 944 |
| 15 | plos one | 940 |
| 16 | j clin invest | 882 |
| 17 | eur j heart fail | 858 |
| 18 | j am soc nephrol | 845 |
| 19 | am j physiol-renal | 785 |
| 20 | ann intern med | 774 |
| 21 | bmj-brit med j | 726 |
| 22 | diabetes res clin pr | 714 |
| 23 | diabetic med | 643 |
| 24 | hypertension | 531 |
| 25 | diabetes ther | 522 |
| 26 | diabetes vasc dis re | 511 |
| 27 | circ res | 502 |
| 28 | am j cardiol | 486 |
| 29 | curr med res opin | 486 |
| 30 | j diabetes complicat | 474 |
| 31 | am heart j | 473 |
| 32 | clin ther | 454 |
| 33 | j hypertens | 402 |
| 34 | diabetes metab | 397 |
| 35 | int j cardiol | 394 |
| 36 | am j kidney dis | 378 |
| 37 | j biol chem | 371 |
| 38 | j diabetes invest | 366 |
| 39 | diabetes-metab res | 349 |
| 40 | cardiovasc res | 339 |
| 41 | metabolism | 336 |
| 42 | endocr pract | 335 |
| 43 | drugs | 325 |
| 44 | postgrad med | 323 |
| 45 | j am heart assoc | 316 |
| 46 | nephrol dial transpl | 302 |
| 47 | clin j am soc nephro | 289 |
| 48 | int j clin pract | 288 |
| 49 | arch intern med | 287 |
| 50 | cell metab | 265 |
| 51 | expert opin pharmaco | 264 |
| 52 | j am soc hypertens | 259 |
| 53 | am j physiol-heart c | 255 |
| 54 | bmj open | 250 |
| 55 | arterioscl throm vas | 248 |
| 56 | atherosclerosis | 248 |
| 57 | am j med | 240 |
| 58 | nat rev endocrinol | 239 |
| 59 | sci rep-uk | 239 |
| 60 | j pharmacol exp ther | 235 |
| 61 | circ-heart fail | 232 |
| 62 | adv ther | 229 |
| 63 | p natl acad sci usa | 219 |
| 64 | physiol rev | 219 |
| 65 | nature | 208 |
| 66 | am j physiol-endoc m | 207 |
| 67 | curr opin nephrol hy | 206 |
| 68 | nat med | 204 |
| 69 | eur j pharmacol | 198 |
| 70 | cochrane db syst rev | 196 |
| 71 | nat rev nephrol | 193 |
| 72 | cardiovasc drug ther | 190 |
| 73 | jacc-heart fail | 188 |
| 74 | endocrinology | 185 |
| 75 | clin pharmacokinet | 180 |
| 76 | am j nephrol | 178 |
| 77 | clin pharmacol ther | 178 |
| 78 | jama cardiol | 178 |
| 79 | j mol cell cardiol | 170 |
| 80 | brit j pharmacol | 167 |
| 81 | curr diabetes rep | 167 |
| 82 | can j diabetes | 162 |
| 83 | diabetes technol the | 158 |
| 84 | obesity | 157 |
| 85 | am j physiol-reg i | 156 |
| 86 | value health | 150 |
| 87 | nutr metab cardiovas | 146 |
| 88 | diabetol metab syndr | 145 |
| 89 | endocr rev | 143 |
| 90 | am j physiol-cell ph | 140 |
| 91 | biochem bioph res co | 139 |
| 92 | stroke | 138 |
| 93 | am j hypertens | 137 |
| 94 | acta diabetol | 132 |
| 95 | expert opin drug met | 132 |
| 96 | expert opin drug saf | 132 |
| 97 | jacc basic transl sci | 132 |
| 98 | brit med j | 131 |
| 99 | hepatology | 131 |
| 100 | j med chem | 130 |
| 101 | am j physiol | 129 |
| 102 | j card fail | 127 |
| 103 | j clin hypertens | 125 |
| 104 | circ j | 124 |
| 105 | free radical bio med | 124 |
| 106 | clin sci | 123 |
| 107 | horm metab res | 121 |
| 108 | hypertens res | 120 |
| 109 | j clin med res | 120 |
| 110 | j diabetes | 119 |
| 111 | j intern med | 118 |
| 112 | guid ind diab mell e | 117 |
| 113 | world j diabetes | 114 |
| 114 | int j mol sci | 112 |
| 115 | brit j clin pharmaco | 111 |
| 116 | expert opin inv drug | 107 |
| 117 | nat rev drug discov | 106 |
| 118 | j clin pharmacol | 105 |
| 119 | endocrine | 104 |
| 120 | am j clin nutr | 102 |
| 121 | diabetes metab syndr | 102 |
| 122 | heart fail rev | 102 |
| 123 | jama intern med | 102 |
| 124 | drug safety | 99 |
| 125 | j diabetes res | 99 |
| 126 | heart | 98 |
| 127 | int j obesity | 96 |
| 128 | nat rev cardiol | 96 |
| 129 | faseb j | 95 |
| 130 | curr pharm design | 94 |
| 131 | endocr j | 94 |
| 132 | pflug arch eur j phy | 94 |
| 133 | j am geriatr soc | 93 |
| 134 | diabetes metab j | 92 |
| 135 | fda drug saf comm fd | 92 |
| 136 | clin drug invest | 91 |
| 137 | gastroenterology | 91 |
| 138 | science | 91 |
| 139 | bmc med | 86 |
| 140 | curr vasc pharmacol | 86 |
| 141 | pharmacoepidem dr s | 86 |
| 142 | idf diabetes atlas | 85 |
| 143 | j hepatol | 85 |
| 144 | redox biol | 85 |
| 145 | drug des dev ther | 84 |
| 146 | ther adv endocrinol | 84 |
| 147 | j physiol-london | 81 |
| 148 | medicine | 81 |
| 149 | plos med | 80 |
| 150 | stat med | 80 |
| 151 | bmc endocr disord | 79 |
| 152 | ann pharmacother | 77 |
| 153 | life sci | 77 |
| 154 | esc heart fail | 76 |
| 155 | front physiol | 75 |
| 156 | j endocrinol | 74 |
| 157 | pharmacotherapy | 74 |
| 158 | indian j endocrinol metab | 72 |
| 159 | j clin epidemiol | 71 |
| 160 | mol cell biochem | 70 |
| 161 | pharmacol therapeut | 70 |
| 162 | prim care diabetes | 69 |
| 163 | vasc health risk man | 69 |
| 164 | clin diabetes | 68 |
| 165 | curr hypertens rep | 66 |
| 166 | eur j prev cardiol | 66 |
| 167 | bioorg med chem lett | 65 |
| 168 | can med assoc j | 65 |
| 169 | pharmacol res | 64 |
| 170 | j cardiovasc pharm | 63 |
| 171 | drug metab dispos | 62 |
| 172 | eur j clin pharmacol | 62 |
| 173 | front endocrinol | 62 |
| 174 | ann med | 60 |
| 175 | cell | 60 |
| 176 | clin exp pharmacol p | 60 |
| 177 | diabet metab synd ob | 60 |
| 178 | basic res cardiol | 59 |
| 179 | hepatol res | 59 |
| 180 | biopharm drug dispos | 58 |
| 181 | eur j endocrinol | 58 |
| 182 | biochem j | 57 |
| 183 | bmc cardiovasc disor | 56 |
| 184 | obes rev | 56 |
| 185 | oxid med cell longev | 56 |
| 186 | ann ny acad sci | 55 |
| 187 | clin kidney j | 55 |
| 188 | curr cardiol rep | 55 |
| 189 | curr diabetes rev | 55 |
| 190 | diabetes s1 | 55 |
| 191 | mol cell endocrinol | 55 |
| 192 | antioxid redox sign | 54 |
| 193 | eur j clin invest | 53 |
| 194 | hosp pract (1995) | 53 |
| 195 | int j endocrinol | 53 |
| 196 | internal med | 53 |
| 197 | j bone miner res | 53 |
| 198 | can j cardiol | 52 |
| 199 | nat commun | 52 |
| 200 | scand j clin lab inv | 52 |
| 201 | blood pressure | 51 |
| 202 | bmj open diab res ca | 51 |
| 203 | j nephrol | 51 |
| 204 | trends endocrin met | 51 |
| 205 | annu rev med | 50 |
| 206 | bmc nephrol | 50 |
| 207 | diabetol int | 50 |
| 208 | j cell biochem | 50 |
| 209 | am j physiol-gastr l | 49 |
| 210 | j am soc echocardiog | 49 |
| 211 | physiol rep | 49 |
| 212 | bba-mol basis dis | 48 |
| 213 | bone | 48 |
| 214 | n engl j med | 48 |
| 215 | vasc pharmacol | 48 |
| 216 | biochem pharmacol | 47 |
| 217 | cardiovasc ther | 47 |
| 218 | j clin med | 47 |
| 219 | jci insight | 47 |
| 220 | nat genet | 46 |
| 221 | world j gastroentero | 46 |
| 222 | cell physiol biochem | 45 |
| 223 | j gastroenterol | 45 |
| 224 | mol metab | 45 |
| 225 | prog cardiovasc dis | 45 |
| 226 | am j epidemiol | 44 |
| 227 | am j manag care | 44 |
| 228 | j hum hypertens | 44 |
| 229 | j membrane biol | 44 |
| 230 | mediat inflamm | 44 |
| 231 | antimicrob agents ch | 43 |
| 232 | brit j nutr | 43 |
| 233 | febs lett | 43 |
| 234 | j agr food chem | 43 |
| 235 | j cardiol | 43 |
| 236 | annu rev physiol | 42 |
| 237 | bioorgan med chem | 42 |
| 238 | diabetes care s1 | 42 |
| 239 | j atheroscler thromb | 42 |
| 240 | exp diabetes res | 41 |
| 241 | expert rev clin phar | 41 |
| 242 | j cell physiol | 41 |
| 243 | semin nephrol | 41 |
| 244 | adv chronic kidney d | 40 |
| 245 | am j pathol | 40 |
| 246 | ebiomedicine | 40 |
| 247 | kidney blood press r | 39 |
| 248 | endocrin metab clin | 38 |
| 249 | exp clin endocr diab | 38 |
| 250 | j gen intern med | 38 |
| 251 | j pharmacol sci | 38 |
| 252 | regul peptides | 38 |
| 253 | blood | 37 |
| 254 | front pharmacol | 37 |
| 255 | heart fail clin | 37 |
| 256 | j diabetes sci technol | 37 |
| 257 | j nutr | 37 |
| 258 | mol med rep | 37 |
| 259 | control clin trials | 36 |
| 260 | curr med chem | 36 |
| 261 | j appl physiol | 36 |
| 262 | circ-cardiovasc imag | 35 |
| 263 | guid clin inv med pr | 35 |
| 264 | int j epidemiol | 35 |
| 265 | aliment pharm ther | 34 |
| 266 | clin pharm drug dev | 34 |
| 267 | j pharm pharmacol | 34 |
| 268 | n-s arch pharmacol | 34 |
| 269 | nephrology | 34 |
| 270 | nutr diabetes | 34 |
| 271 | peptides | 34 |
| 272 | clin cardiol | 33 |
| 273 | int j clin pharm th | 33 |
| 274 | j clin lipidol | 33 |
| 275 | nephron | 33 |
| 276 | can j physiol pharm | 32 |
| 277 | clin endocrinol | 32 |
| 278 | curr heart fail rep | 32 |
| 279 | diabetes metab synd | 32 |
| 280 | j med econ | 32 |
| 281 | mayo clin proc | 32 |
| 282 | med clin n am | 32 |
| 283 | res synth methods | 32 |
| 284 | trends pharmacol sci | 32 |
| 285 | am j cardiovasc drug | 31 |
| 286 | bmc pharmacol toxico | 31 |
| 287 | j lipid res | 31 |
| 288 | patient prefer adher | 31 |
| 289 | pharmacol rev | 31 |
| 290 | renal failure | 31 |
| 291 | biomed pharmacother | 30 |
| 292 | curr opin endocrinol | 30 |
| 293 | lipids health dis | 30 |
| 294 | osteoporosis int | 30 |
| 295 | clin chem | 29 |
| 296 | curr atheroscler rep | 29 |
| 297 | eur heart j-card pha | 29 |
| 298 | j immunol | 29 |
| 299 | j renin-angio-aldo s | 29 |
| 300 | oncotarget | 29 |
| 301 | adv exp med biol | 28 |
| 302 | jacc-cardiovasc imag | 28 |
| 303 | biomed res int | 27 |
| 304 | clin res cardiol | 27 |
| 305 | expert opin ther tar | 27 |
| 306 | gut | 27 |
| 307 | j antibiot | 27 |
| 308 | pharmacoeconomics | 27 |
| 309 | acta physiol | 26 |
| 310 | arch med sci | 26 |
| 311 | curr treat options cardiovasc med | 26 |
| 312 | drugs context | 26 |
| 313 | eur j intern med | 26 |
| 314 | glob rep diab | 26 |
| 315 | j endocrinol invest | 26 |
| 316 | nutrients | 26 |
| 317 | obes res | 26 |
| 318 | rev diabet stud | 26 |
| 319 | angiology | 25 |
| 320 | curr opin cardiol | 25 |
| 321 | eur heart j-card img | 25 |
| 322 | hum mol genet | 25 |
| 323 | j nucl med | 25 |
| 324 | j nutr biochem | 25 |
| 325 | mol cell biol | 25 |
| 326 | neurology | 25 |
| 327 | postgrad med j | 25 |
| 328 | rev endocr metab dis | 25 |
| 329 | rev esp cardiol | 25 |
| 330 | am j med sci | 24 |
| 331 | bmc health serv res | 24 |
| 332 | j transl med | 24 |
| 333 | biochemistry-us | 23 |
| 334 | biochim biophys acta | 23 |
| 335 | biometrics | 23 |
| 336 | bmc res notes | 23 |
| 337 | chest | 23 |
| 338 | endocrinol metab | 23 |
| 339 | eur j clin nutr | 23 |
| 340 | front cardiovasc med | 23 |
| 341 | j cardiovasc pharm t | 23 |
| 342 | j invest med | 23 |
| 343 | mol aspects med | 23 |
| 344 | mol nutr food res | 23 |
| 345 | nat diab stat rep | 23 |
| 346 | sci transl med | 23 |
| 347 | autophagy | 22 |
| 348 | bmc public health | 22 |
| 349 | clin exp nephrol | 22 |
| 350 | diabetologia s1 | 22 |
| 351 | j am chem soc | 22 |
| 352 | j ethnopharmacol | 22 |
| 353 | j vasc surg | 22 |
| 354 | med decis making | 22 |
| 355 | mol pharmacol | 22 |
| 356 | am heart ass ann sci | 21 |
| 357 | am j physiol-lung c | 21 |
| 358 | am j transplant | 21 |
| 359 | cell signal | 21 |
| 360 | clin gastroenterol h | 21 |
| 361 | exp physiol | 21 |
| 362 | heart vessels | 21 |
| 363 | int heart j | 21 |
| 364 | j clin transl endocr | 21 |
| 365 | mult trial ev eff da | 21 |
| 366 | nat diab fact sheet | 21 |
| 367 | pediatr diabetes | 21 |
| 368 | ther clin risk manag | 21 |
| 369 | ageing res rev | 20 |
| 370 | cardiol rev | 20 |
| 371 | curr drug saf | 20 |
| 372 | curr opin lipidol | 20 |
| 373 | drug aging | 20 |
| 374 | drug metab pharmacok | 20 |
| 375 | health technol asses | 20 |
| 376 | int j mol med | 20 |
| 377 | invest ophth vis sci | 20 |
| 378 | j cell mol med | 20 |
| 379 | j gerontol a-biol | 20 |
| 380 | j nat prod | 20 |
| 381 | nat rev dis primers | 20 |
| 382 | prostag leukotr ess | 20 |
| 383 | xenobiotica | 20 |
| 384 | am j gastroenterol | 19 |
| 385 | am j health-syst ph | 19 |
| 386 | bmc med res methodol | 19 |
| 387 | cardiology | 19 |
| 388 | cell death dis | 19 |
| 389 | cmaj open | 19 |
| 390 | curr opin pharmacol | 19 |
| 391 | eur j cardiov prev r | 19 |
| 392 | eur j pharm sci | 19 |
| 393 | int j biochem cell b | 19 |
| 394 | j cell sci | 19 |
| 395 | med care | 19 |
| 396 | med j australia | 19 |
| 397 | pharmacol res perspe | 19 |
| 398 | biol pharm bull | 18 |
| 399 | clin chim acta | 18 |
| 400 | compr physiol | 18 |
| 401 | j exp med | 18 |
| 402 | med sci sport exer | 18 |
| 403 | p t | 18 |
| 404 | qual life res | 18 |
| 405 | trends cardiovas med | 18 |
| 406 | trials | 18 |
| 407 | basic clin pharmacol | 17 |
| 408 | bba-mol cell biol l | 17 |
| 409 | best pract res cl en | 17 |
| 410 | biochem soc t | 17 |
| 411 | biochimie | 17 |
| 412 | cell mol life sci | 17 |
| 413 | circ-cardiovasc inte | 17 |
| 414 | clin exp hypertens | 17 |
| 415 | clin nephrol | 17 |
| 416 | clin pharmacol-adv a | 17 |
| 417 | crit care | 17 |
| 418 | diab mell ev card ri | 17 |
| 419 | eur rev med pharmaco | 17 |
| 420 | heart rhythm | 17 |
| 421 | int j med sci | 17 |
| 422 | j amer med assoc | 17 |
| 423 | j cell biol | 17 |
| 424 | j neurochem | 17 |
| 425 | liver int | 17 |
| 426 | molecules | 17 |
| 427 | nephron physiol | 17 |
| 428 | nutrition | 17 |
| 429 | ppar res | 17 |
| 430 | transplantation | 17 |
| 431 | ann rheum dis | 16 |
| 432 | arch biochem biophys | 16 |
| 433 | brit heart j | 16 |
| 434 | drug today | 16 |
| 435 | expert opin ther pat | 16 |
| 436 | expert rev cardiovas | 16 |
| 437 | front immunol | 16 |
| 438 | int j surg | 16 |
| 439 | j diabetes metab dis | 16 |
| 440 | j fam practice | 16 |
| 441 | med sci monitor | 16 |
| 442 | nat diab stat rep 20 | 16 |
| 443 | nat immunol | 16 |
| 444 | nat rev immunol | 16 |
| 445 | bba-biomembranes | 15 |
| 446 | bba-mol cell res | 15 |
| 447 | cell rep | 15 |
| 448 | clin biochem | 15 |
| 449 | cochrane collaborati | 15 |
| 450 | curr ther res clin e | 15 |
| 451 | deut med wochenschr | 15 |
| 452 | exp cell res | 15 |
| 453 | health econ | 15 |
| 454 | histochem cell biol | 15 |
| 455 | int urol nephrol | 15 |
| 456 | intern med j | 15 |
| 457 | j magn reson imaging | 15 |
| 458 | j mol endocrinol | 15 |
| 459 | jacc-cardiovasc inte | 15 |
| 460 | nat clin pract endoc | 15 |
| 461 | nat diab stat rep es | 15 |
| 462 | sci world j | 15 |
| 463 | ther adv drug saf | 15 |
| 464 | thromb haemostasis | 15 |
| 465 | toxicol appl pharm | 15 |
| 466 | am j ther | 14 |
| 467 | annu rev nutr | 14 |
| 468 | curr osteoporos rep | 14 |
| 469 | diabetes educator | 14 |
| 470 | diabetes s1a | 14 |
| 471 | embo j | 14 |
| 472 | handb exp pharmacol | 14 |
| 473 | int j hypertens | 14 |
| 474 | invokana can tabl or | 14 |
| 475 | j assoc physicians india | 14 |
| 476 | j korean med sci | 14 |
| 477 | j mol med | 14 |
| 478 | j nucl cardiol | 14 |
| 479 | metab syndr relat d | 14 |
| 480 | rev cardiovasc med | 14 |
| 481 | tex heart i j | 14 |
| 482 | aging male | 13 |
| 483 | am j resp crit care | 13 |
| 484 | card fail rev | 13 |
| 485 | cardiovasc pathol | 13 |
| 486 | chem-biol interact | 13 |
| 487 | circ-cardiovasc qual | 13 |
| 488 | clin nutr | 13 |
| 489 | curr drug targets | 13 |
| 490 | diab med cont sax al | 13 |
| 491 | diabetol stoffwechs | 13 |
| 492 | eur j epidemiol | 13 |
| 493 | eur j nutr | 13 |
| 494 | exp neurol | 13 |
| 495 | exp ther med | 13 |
| 496 | fda appr jard red ca | 13 |
| 497 | iubmb life | 13 |
| 498 | j clin pharm ther | 13 |
| 499 | j gastroen hepatol | 13 |
| 500 | j int med res | 13 |
| 501 | kidney int suppl | 13 |
| 502 | lab invest | 13 |
| 503 | med lett drugs ther | 13 |
| 504 | nat rev mol cell bio | 13 |
| 505 | open heart | 13 |
| 506 | semin thromb hemost | 13 |
| 507 | surg obes relat dis | 13 |
| 508 | toxicol pathol | 13 |
| 509 | am coll card ann sci | 12 |
| 510 | am j transl res | 12 |
| 511 | ann thorac surg | 12 |
| 512 | ann transl med | 12 |
| 513 | brain res | 12 |
| 514 | cancer res | 12 |
| 515 | card outc foll ert t | 12 |
| 516 | clev clin j med | 12 |
| 517 | clin cancer res | 12 |
| 518 | clin invest med | 12 |
| 519 | cochrane hdb systema | 12 |
| 520 | curr cardiol rev | 12 |
| 521 | diabetes res clin pract | 12 |
| 522 | drug saf comm fda wa | 12 |
| 523 | eur endocrinol | 12 |
| 524 | eur j med chem | 12 |
| 525 | expert opin biol th | 12 |
| 526 | fda warns sglt2 inh | 12 |
| 527 | heart lung circ | 12 |
| 528 | int j cancer | 12 |
| 529 | int j gen med | 12 |
| 530 | j am med dir assoc | 12 |
| 531 | j cardiovasc med | 12 |
| 532 | j clin hypertens (greenwich) | 12 |
| 533 | j heart lung transpl | 12 |
| 534 | j lab clin med | 12 |
| 535 | j neurol neurosur ps | 12 |
| 536 | j pineal res | 12 |
| 537 | j stat softw | 12 |
| 538 | j thorac cardiov sur | 12 |
| 539 | med hypotheses | 12 |
| 540 | mol endocrinol | 12 |
| 541 | nat cell biol | 12 |
| 542 | nat clin pract card | 12 |
| 543 | nat prod rep | 12 |
| 544 | nat rev gastro hepat | 12 |
| 545 | physiology | 12 |
| 546 | regul toxicol pharm | 12 |
| 547 | rev diab med call sg | 12 |
| 548 | stem cells | 12 |
| 549 | world psychiatry | 12 |
| 550 | zhonghua jie he he h | 12 |
| 551 | am diab ass 73 sci s | 11 |
| 552 | am fam physician | 11 |
| 553 | am health drug benefits | 11 |
| 554 | braz j med biol res | 11 |
| 555 | clin trials | 11 |
| 556 | crit care med | 11 |
| 557 | curr diab rep | 11 |
| 558 | curr hypertens rev | 11 |
| 559 | diabetes spectr | 11 |
| 560 | drug discov today | 11 |
| 561 | eur j echocardiogr | 11 |
| 562 | europace | 11 |
| 563 | ev eff can ren card | 11 |
| 564 | fda rev lab sglt2 in | 11 |
| 565 | guid ind diab mell c | 11 |
| 566 | j cardiovasc transl | 11 |
| 567 | j manage care pharm | 11 |
| 568 | j org chem | 11 |
| 569 | j physiol biochem | 11 |
| 570 | j sex med | 11 |
| 571 | lipids | 11 |
| 572 | obes sci pract | 11 |
| 573 | obes surg | 11 |
| 574 | pediatr nephrol | 11 |
| 575 | pharm stat | 11 |
| 576 | qjm-int j med | 11 |
| 577 | r lang env stat comp | 11 |
| 578 | radiology | 11 |
| 579 | tetrahedron lett | 11 |
| 580 | typ 2 diab ad man | 11 |
| 581 | world j cardiol | 11 |
| 582 | world j hepatol | 11 |
| 583 | acta med scand | 10 |
| 584 | acta pharmacol sin | 10 |
| 585 | acta physiol scand | 10 |
| 586 | aging cell | 10 |
| 587 | aging-us | 10 |
| 588 | amino acids | 10 |
| 589 | ann acad sci francai | 10 |
| 590 | auton neurosci-basic | 10 |
| 591 | bba-gen subjects | 10 |
| 592 | br j diabetes vasc d | 10 |
| 593 | calcified tissue int | 10 |
| 594 | can fam physician | 10 |
| 595 | chem pharm bull | 10 |
| 596 | chinese med j-peking | 10 |
| 597 | clin chem lab med | 10 |
| 598 | contrib nephrol | 10 |
| 599 | cpt-pharmacomet syst | 10 |
| 600 | dis model mech | 10 |
| 601 | drug res | 10 |
| 602 | drugs r&d | 10 |
| 603 | end met drugs adv co | 10 |
| 604 | epidemiology | 10 |
| 605 | eur j biochem | 10 |
| 606 | eur j gastroen hepat | 10 |
| 607 | eurointervention | 10 |
| 608 | fda conf incr risk l | 10 |
| 609 | food chem toxicol | 10 |
| 610 | front endocrinol (lausanne) | 10 |
| 611 | gene dev | 10 |
| 612 | health affair | 10 |
| 613 | horm-int j endocrino | 10 |
| 614 | infect immun | 10 |
| 615 | int j antimicrob ag | 10 |
| 616 | int j clin exp patho | 10 |
| 617 | int j toxicol | 10 |
| 618 | inv can | 10 |
| 619 | j am stat assoc | 10 |
| 620 | j diabetes metab | 10 |
| 621 | j epidemiol commun h | 10 |
| 622 | j pediatr-us | 10 |
| 623 | j pharmacol tox met | 10 |
| 624 | j stroke cerebrovasc | 10 |
| 625 | jama netw open | 10 |
| 626 | kardiol pol | 10 |
| 627 | kidney int s | 10 |
| 628 | maturitas | 10 |
| 629 | nephron clin pract | 10 |
| 630 | open med | 10 |
| 631 | ophthalmology | 10 |
| 632 | pharmacology | 10 |
| 633 | schizophr res | 10 |
| 634 | stata j | 10 |
| 635 | thromb res | 10 |
| 636 | tohoku j exp med | 10 |
| 637 | toxicol sci | 10 |
| 638 | vasc med | 10 |
| 639 | world j nephrol | 10 |

**Note:** SGLT2: Sodium Glucose Cotransporter 2. CV: cardiovascular
