# Supplementary material for: Bibliometric Study of Sodium Glucose Cotransporter 2 Inhibitors in Cardiovascular Research
Source: Front Pharmacol. 2020 Sep 15;11:561494. doi: 10.3389/fphar.2020.561494 (PMC7522576; doi:10.3389/fphar.2020.561494)
Supplement: Supplementary file 7 [file Table_7.docx]

Supplementary Material

**
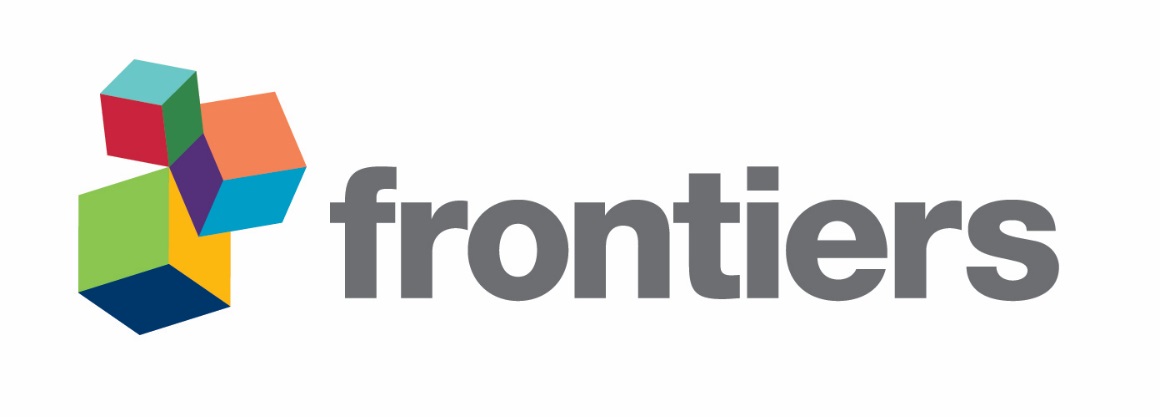
**

**Supplementary Table 7.** Authors publishing the articles of SGLT2 inhibitors in CV research.

| **Rank** | **Productive author** | **Articles** | **% of 1509** |
| --- | --- | --- | --- |
| 1 | INZUCCHI SE | 42 | 2.783 |
| 2 | ZINMAN B | 34 | 2.253 |
| 3 | SCHEEN AJ | 32 | 2.121 |
| 4 | HEERSPINK HJL | 31 | 2.054 |
| 5 | MCGUIRE DK | 30 | 1.988 |
| 6 | PERKOVIC V | 30 | 1.988 |
| 7 | VERMA S | 30 | 1.988 |
| 8 | WOERLE HJ | 28 | 1.856 |
| 9 | MAHAFFEY KW | 27 | 1.789 |
| 10 | NEAL B | 27 | 1.789 |
| 11 | WANNER C | 27 | 1.789 |
| 12 | BROEDL UC | 25 | 1.657 |
| 13 | DAVIES MJ | 24 | 1.59 |
| 14 | FITCHETT D | 24 | 1.59 |
| 15 | DE ZEEUW D | 23 | 1.524 |
| 16 | LEITER LA | 22 | 1.458 |
| 17 | JOHANSEN OE | 21 | 1.392 |
| 18 | KHUNTI K | 21 | 1.392 |
| 19 | SABATINE MS | 21 | 1.392 |
| 20 | WILDING JPH | 21 | 1.392 |
| 21 | BUTLER J | 20 | 1.325 |
| 22 | DESAI M | 20 | 1.325 |
| 23 | LANGKILDE AM | 19 | 1.259 |
| 24 | BHATT DL | 18 | 1.193 |
| 25 | CHERNEY DZI | 18 | 1.193 |
| 26 | GEORGE JT | 18 | 1.193 |
| 27 | KOSIBOROD M | 18 | 1.193 |
| 28 | ROSENSTOCK J | 18 | 1.193 |
| 29 | FULCHER G | 17 | 1.127 |
| 30 | MATTHEWS DR | 17 | 1.127 |
| 31 | FIORETTO P | 16 | 1.06 |
| 32 | RAZ I | 16 | 1.06 |
| 33 | TENTOLOURIS N | 16 | 1.06 |
| 34 | VALLON V | 16 | 1.06 |
| 35 | MATHIEU C | 15 | 0.994 |
| 36 | PACKER M | 15 | 0.994 |
| 37 | WOO V | 15 | 0.994 |
| 38 | AVOGARO A | 14 | 0.928 |
| 39 | MOSENZON O | 14 | 0.928 |
| 40 | SCHERNTHANER G | 14 | 0.928 |
| 41 | THURESSON M | 14 | 0.928 |
| 42 | CAHN A | 13 | 0.861 |
| 43 | HANTEL S | 13 | 0.861 |
| 44 | SHAW W | 13 | 0.861 |
| 45 | SJOSTROM CD | 13 | 0.861 |
| 46 | TADDEI S | 13 | 0.861 |
| 47 | TAKAHASHI H | 13 | 0.861 |
| 48 | WIVIOTT SD | 13 | 0.861 |
| 49 | DEEROCHANAWONG C | 12 | 0.795 |
| 50 | DOUMAS M | 12 | 0.795 |
| 51 | DREXEL H | 12 | 0.795 |
| 52 | FENICI P | 12 | 0.795 |
| 53 | GUPTA M | 12 | 0.795 |
| 54 | KIM H | 12 | 0.795 |
| 55 | MEININGER G | 12 | 0.795 |
| 56 | PARK H | 12 | 0.795 |
| 57 | PONIKOWSKI P | 12 | 0.795 |
| 58 | STAVROPOULOS K | 12 | 0.795 |
| 59 | AGAFYINA A | 11 | 0.729 |
| 60 | AIZENBERG D | 11 | 0.729 |
| 61 | AQUITANIA G | 11 | 0.729 |
| 62 | ARIF A | 11 | 0.729 |
| 63 | AWAD A | 11 | 0.729 |
| 64 | BARBARASH O | 11 | 0.729 |
| 65 | CHEN J | 11 | 0.729 |
| 66 | COLOMBO H | 11 | 0.729 |
| 67 | COSMA D | 11 | 0.729 |
| 68 | CRISAN C | 11 | 0.729 |
| 69 | DISTILLER L | 11 | 0.729 |
| 70 | DUDAS M | 11 | 0.729 |
| 71 | FADINI GP | 11 | 0.729 |
| 72 | GHANI RA | 11 | 0.729 |
| 73 | GNASSO A | 11 | 0.729 |
| 74 | GODLEVSKA O | 11 | 0.729 |
| 75 | HADJADJ S | 11 | 0.729 |
| 76 | HENEIN S | 11 | 0.729 |
| 77 | JOHNSSON E | 11 | 0.729 |
| 78 | JURGENS J | 11 | 0.729 |
| 79 | KADIR KA | 11 | 0.729 |
| 80 | KIM Y | 11 | 0.729 |
| 81 | KONDO Y | 11 | 0.729 |
| 82 | LASTUVKA J | 11 | 0.729 |
| 83 | MARX N | 11 | 0.729 |
| 84 | MOHAN V | 11 | 0.729 |
| 85 | MOOKADAM M | 11 | 0.729 |
| 86 | NETO BG | 11 | 0.729 |
| 87 | NORTJE H | 11 | 0.729 |
| 88 | PARK S | 11 | 0.729 |
| 89 | POZZILLI P | 11 | 0.729 |
| 90 | RIDDERSTRALE M | 11 | 0.729 |
| 91 | SALAZAR MA | 11 | 0.729 |
| 92 | SAMOYLOV O | 11 | 0.729 |
| 93 | SCOTT R | 11 | 0.729 |
| 94 | SUASTIKA K | 11 | 0.729 |
| 95 | TAN G | 11 | 0.729 |
| 96 | TSAPAS A | 11 | 0.729 |
| 97 | TSELUYKO V | 11 | 0.729 |
| 98 | VAN GAAL L | 11 | 0.729 |
| 99 | WAKIDA Y | 11 | 0.729 |
| 100 | WANG J | 11 | 0.729 |
| 101 | WEINER P | 11 | 0.729 |
| 102 | ZAOUI P | 11 | 0.729 |
| 103 | ANKER SD | 10 | 0.663 |
| 104 | AUSTIN B | 10 | 0.663 |
| 105 | AVRAMIDIS I | 10 | 0.663 |
| 106 | BODEGARD J | 10 | 0.663 |
| 107 | BRANDON D | 10 | 0.663 |
| 108 | BRUSCO O | 10 | 0.663 |
| 109 | CHILTON R | 10 | 0.663 |
| 110 | CHRISTENSEN T | 10 | 0.663 |
| 111 | COLIVICCHI F | 10 | 0.663 |
| 112 | CUDDIHY R | 10 | 0.663 |
| 113 | D'EMDEN M | 10 | 0.663 |
| 114 | EDER F | 10 | 0.663 |
| 115 | FASCHING P | 10 | 0.663 |
| 116 | GUPTA S | 10 | 0.663 |
| 117 | HIESHIMA K | 10 | 0.663 |
| 118 | HOFFMAN J | 10 | 0.663 |
| 119 | ITO M | 10 | 0.663 |
| 120 | JARDINE MJ | 10 | 0.663 |
| 121 | JINNOUCHI H | 10 | 0.663 |
| 122 | JURISIC-ERZEN D | 10 | 0.663 |
| 123 | KOOY A | 10 | 0.663 |
| 124 | LANGSLET G | 10 | 0.663 |
| 125 | LEE S | 10 | 0.663 |
| 126 | LEWIS D | 10 | 0.663 |
| 127 | MATTHEUS M | 10 | 0.663 |
| 128 | MAYOUX E | 10 | 0.663 |
| 129 | MCMURRAY JJV | 10 | 0.663 |
| 130 | MESA J | 10 | 0.663 |
| 131 | MONTEIRO P | 10 | 0.663 |
| 132 | NORHAMMAR A | 10 | 0.663 |
| 133 | OMAR M | 10 | 0.663 |
| 134 | OZAKI R | 10 | 0.663 |
| 135 | PATEL A | 10 | 0.663 |
| 136 | PATEL N | 10 | 0.663 |
| 137 | RENDELL M | 10 | 0.663 |
| 138 | SALSALI A | 10 | 0.663 |
| 139 | SATTAR N | 10 | 0.663 |
| 140 | SHANIK M | 10 | 0.663 |
| 141 | SIMPSON R | 10 | 0.663 |
| 142 | SOLOMON SD | 10 | 0.663 |
| 143 | STEDMAN M | 10 | 0.663 |
| 144 | TAKAHASHI M | 10 | 0.663 |
| 145 | TAKENAKA T | 10 | 0.663 |
| 146 | TAN K | 10 | 0.663 |
| 147 | TOURAL E | 10 | 0.663 |
| 148 | VON EYNATTEN M | 10 | 0.663 |
| 149 | WARREN M | 10 | 0.663 |
| 150 | WEISS R | 10 | 0.663 |
| 151 | WOJNOWSKI L | 10 | 0.663 |
| 152 | WYSHAM C | 10 | 0.663 |
| 153 | YANG J | 10 | 0.663 |
| 154 | YEH H | 10 | 0.663 |
| 155 | ZANIEWSKI-SINGH M | 10 | 0.663 |
| 156 | ZELNIKER TA | 10 | 0.663 |
| 157 | ABU HASSAN M | 9 | 0.596 |
| 158 | ADAWI F | 9 | 0.596 |
| 159 | ADOJAAN B | 9 | 0.596 |
| 160 | AGUILAR-SALINAS C | 9 | 0.596 |
| 161 | AGUNG P | 9 | 0.596 |
| 162 | AHMAD NN | 9 | 0.596 |
| 163 | AIELLO A | 9 | 0.596 |
| 164 | AJANI D | 9 | 0.596 |
| 165 | AL-WINDY N | 9 | 0.596 |
| 166 | ALOI J | 9 | 0.596 |
| 167 | ALZOHAILI O | 9 | 0.596 |
| 168 | AMOD A | 9 | 0.596 |
| 169 | ANDERSEN J | 9 | 0.596 |
| 170 | ANDERSON M | 9 | 0.596 |
| 171 | ANDRADE L | 9 | 0.596 |
| 172 | ANSPACH R | 9 | 0.596 |
| 173 | ANTOLI A | 9 | 0.596 |
| 174 | ANTONYPILLAI C | 9 | 0.596 |
| 175 | ARAVIND S | 9 | 0.596 |
| 176 | ARCA M | 9 | 0.596 |
| 177 | ARENA C | 9 | 0.596 |
| 178 | ARONOFF S | 9 | 0.596 |
| 179 | ARTOLA S | 9 | 0.596 |
| 180 | ARUTYUNOV G | 9 | 0.596 |
| 181 | AURE P | 9 | 0.596 |
| 182 | AYOUB J | 9 | 0.596 |
| 183 | BADGANDI M | 9 | 0.596 |
| 184 | BALASKO A | 9 | 0.596 |
| 185 | BALIC S | 9 | 0.596 |
| 186 | BALL E | 9 | 0.596 |
| 187 | BANTWAL G | 9 | 0.596 |
| 188 | BARAGER W | 9 | 0.596 |
| 189 | BARANOWSKI M | 9 | 0.596 |
| 190 | BARBARICH V | 9 | 0.596 |
| 191 | BARBONTA D | 9 | 0.596 |
| 192 | BARKER B | 9 | 0.596 |
| 193 | BASHKIN A | 9 | 0.596 |
| 194 | BAYLY K | 9 | 0.596 |
| 195 | BELENKIY D | 9 | 0.596 |
| 196 | BENATAR J | 9 | 0.596 |
| 197 | BENHALIMA K | 9 | 0.596 |
| 198 | BENROUBI M | 9 | 0.596 |
| 199 | BERGENSTAL R | 9 | 0.596 |
| 200 | BERGMAN B | 9 | 0.596 |
| 201 | BERLINGIERI J | 9 | 0.596 |
| 202 | BERTOLAMI M | 9 | 0.596 |
| 203 | BERZ A | 9 | 0.596 |
| 204 | BETTI R | 9 | 0.596 |
| 205 | BITTAR N | 9 | 0.596 |
| 206 | BLAKNEY E | 9 | 0.596 |
| 207 | BLAZE K | 9 | 0.596 |
| 208 | BLIGNAUT S | 9 | 0.596 |
| 209 | BOESGAARD TW | 9 | 0.596 |
| 210 | BONONI P | 9 | 0.596 |
| 211 | BORGES J | 9 | 0.596 |
| 212 | BOSCIA J | 9 | 0.596 |
| 213 | BOUCHER P | 9 | 0.596 |
| 214 | BOUSBOULAS S | 9 | 0.596 |
| 215 | BOWDEN R | 9 | 0.596 |
| 216 | BOYARKIN M | 9 | 0.596 |
| 217 | BRATCHER C | 9 | 0.596 |
| 218 | BRAVO LG | 9 | 0.596 |
| 219 | BRAZAO M | 9 | 0.596 |
| 220 | BRESSLER P | 9 | 0.596 |
| 221 | BRETON C | 9 | 0.596 |
| 222 | BRETTON E | 9 | 0.596 |
| 223 | BRITO M | 9 | 0.596 |
| 224 | BROCKMYRE A | 9 | 0.596 |
| 225 | BUCCI M | 9 | 0.596 |
| 226 | BULUGAHAPITIYA D | 9 | 0.596 |
| 227 | BURGESS L | 9 | 0.596 |
| 228 | BURR R | 9 | 0.596 |
| 229 | BURTCHULADZE T | 9 | 0.596 |
| 230 | BUTLER M | 9 | 0.596 |
| 231 | BUYNAK R | 9 | 0.596 |
| 232 | BYSTROVA A | 9 | 0.596 |
| 233 | CAHILL T | 9 | 0.596 |
| 234 | CALABRO P | 9 | 0.596 |
| 235 | CALDERON JU | 9 | 0.596 |
| 236 | CALDWELL I | 9 | 0.596 |
| 237 | CAMAFORT M | 9 | 0.596 |
| 238 | CAPIAU L | 9 | 0.596 |
| 239 | CASTRO M | 9 | 0.596 |
| 240 | CATRINOIU D | 9 | 0.596 |
| 241 | CEFALU W | 9 | 0.596 |
| 242 | CHACHATI A | 9 | 0.596 |
| 243 | CHACRA A | 9 | 0.596 |
| 244 | CHAN Y | 9 | 0.596 |
| 245 | CHANDRAMOULI A | 9 | 0.596 |
| 246 | CHANG H | 9 | 0.596 |
| 247 | CHANG K | 9 | 0.596 |
| 248 | CHAPPELL D | 9 | 0.596 |
| 249 | CHAUHAN A | 9 | 0.596 |
| 250 | CHAYKIN L | 9 | 0.596 |
| 251 | CHEE K | 9 | 0.596 |
| 252 | CHEHAYEB R | 9 | 0.596 |
| 253 | CHERLIN R | 9 | 0.596 |
| 254 | CHIANG C | 9 | 0.596 |
| 255 | CHOKSI M | 9 | 0.596 |
| 256 | CHOUKROUN G | 9 | 0.596 |
| 257 | CHRISTENSEN P | 9 | 0.596 |
| 258 | CHUMAKOVA G | 9 | 0.596 |
| 259 | CIGNARELLI M | 9 | 0.596 |
| 260 | CLAVEL S | 9 | 0.596 |
| 261 | CLAYTON D | 9 | 0.596 |
| 262 | COLFER H | 9 | 0.596 |
| 263 | CONCHA YR | 9 | 0.596 |
| 264 | CONNERY L | 9 | 0.596 |
| 265 | COUFFINHAL T | 9 | 0.596 |
| 266 | COURREGES J | 9 | 0.596 |
| 267 | CRUZ H | 9 | 0.596 |
| 268 | CURTIS C | 9 | 0.596 |
| 269 | DAUGENET C | 9 | 0.596 |
| 270 | DE JONG A | 9 | 0.596 |
| 271 | DE LA ROSA R | 9 | 0.596 |
| 272 | DE LOREDO L | 9 | 0.596 |
| 273 | DE LOS SANTOS G | 9 | 0.596 |
| 274 | DE TERESA L | 9 | 0.596 |
| 275 | DEFRONZO RA | 9 | 0.596 |
| 276 | DEKELVER P | 9 | 0.596 |
| 277 | DEL CANIZO F | 9 | 0.596 |
| 278 | DELA LLANA A | 9 | 0.596 |
| 279 | DENKER P | 9 | 0.596 |
| 280 | DESHPANDE N | 9 | 0.596 |
| 281 | DEVERS M | 9 | 0.596 |
| 282 | DIAZ J | 9 | 0.596 |
| 283 | DINATO M | 9 | 0.596 |
| 284 | DISANTO L | 9 | 0.596 |
| 285 | DO ROSARIO FS | 9 | 0.596 |
| 286 | DRVODELIC-SUNIC E | 9 | 0.596 |
| 287 | DUARTE R | 9 | 0.596 |
| 288 | DUARTE S | 9 | 0.596 |
| 289 | DULGEROFF A | 9 | 0.596 |
| 290 | DUNMYER S | 9 | 0.596 |
| 291 | EAGERTON D | 9 | 0.596 |
| 292 | EFRATI S | 9 | 0.596 |
| 293 | ELIASCHEWITZ F | 9 | 0.596 |
| 294 | EMANUEL S | 9 | 0.596 |
| 295 | ENGELBRECHT J | 9 | 0.596 |
| 296 | ESCALANTE DM | 9 | 0.596 |
| 297 | ESCUDERO AR | 9 | 0.596 |
| 298 | ESIP V | 9 | 0.596 |
| 299 | ESPOSITO K | 9 | 0.596 |
| 300 | ESTOUR B | 9 | 0.596 |
| 301 | FALUDI A | 9 | 0.596 |
| 302 | FARFAN J | 9 | 0.596 |
| 303 | FARIAS J | 9 | 0.596 |
| 304 | FARMER I | 9 | 0.596 |
| 305 | FARRIS N | 9 | 0.596 |
| 306 | FELD L | 9 | 0.596 |
| 307 | FELDMAN G | 9 | 0.596 |
| 308 | FELICIO J | 9 | 0.596 |
| 309 | FERNANDEZ-CRUZ A | 9 | 0.596 |
| 310 | FIDELEFF H | 9 | 0.596 |
| 311 | FILLIPOVA E | 9 | 0.596 |
| 312 | FIRST B | 9 | 0.596 |
| 313 | FISHMAN N | 9 | 0.596 |
| 314 | FITZ-PATRICK D | 9 | 0.596 |
| 315 | FLIESSER-GORZER E | 9 | 0.596 |
| 316 | FLIPPO G | 9 | 0.596 |
| 317 | FLORES F | 9 | 0.596 |
| 318 | FOGELFELD L | 9 | 0.596 |
| 319 | FOO S | 9 | 0.596 |
| 320 | FORMAGNE L | 9 | 0.596 |
| 321 | FOSSUM C | 9 | 0.596 |
| 322 | FOWLER W | 9 | 0.596 |
| 323 | FREEDMAN Z | 9 | 0.596 |
| 324 | FUCILI A | 9 | 0.596 |
| 325 | FUNG Y | 9 | 0.596 |
| 326 | FURUI K | 9 | 0.596 |
| 327 | GABRA N | 9 | 0.596 |
| 328 | GARCIA-ORTIZ L | 9 | 0.596 |
| 329 | GARDNER T | 9 | 0.596 |
| 330 | GARG N | 9 | 0.596 |
| 331 | GEROO L | 9 | 0.596 |
| 332 | GHAISAS N | 9 | 0.596 |
| 333 | GIORGADZE E | 9 | 0.596 |
| 334 | GIUGLIANO D | 9 | 0.596 |
| 335 | GIUGLIANO G | 9 | 0.596 |
| 336 | GLONTI S | 9 | 0.596 |
| 337 | GOLDONI V | 9 | 0.596 |
| 338 | GONKEL F | 9 | 0.596 |
| 339 | GONZALEZ-CAMPOY J | 9 | 0.596 |
| 340 | GOTTSCHLICH G | 9 | 0.596 |
| 341 | GOUET D | 9 | 0.596 |
| 342 | GOVIND U | 9 | 0.596 |
| 343 | GOYTIA-LEOS D | 9 | 0.596 |
| 344 | GRAF R | 9 | 0.596 |
| 345 | GREEN C | 9 | 0.596 |
| 346 | GREEN F | 9 | 0.596 |
| 347 | GROSS J | 9 | 0.596 |
| 348 | GUPTA J | 9 | 0.596 |
| 349 | GUTLAPALLI K | 9 | 0.596 |
| 350 | GWON H | 9 | 0.596 |
| 351 | HALIMI J | 9 | 0.596 |
| 352 | HALPERN A | 9 | 0.596 |
| 353 | HALPERN S | 9 | 0.596 |
| 354 | HAMMOND G | 9 | 0.596 |
| 355 | HANSEN V | 9 | 0.596 |
| 356 | HANSEN VR | 9 | 0.596 |
| 357 | HASSANI F | 9 | 0.596 |
| 358 | HAYASHIDA C | 9 | 0.596 |
| 359 | HEGGEN E | 9 | 0.596 |
| 360 | HERMANS M | 9 | 0.596 |
| 361 | HERNANDEZ M | 9 | 0.596 |
| 362 | HERNANDEZ PG | 9 | 0.596 |
| 363 | HERSKOVITS T | 9 | 0.596 |
| 364 | HEURICH E | 9 | 0.596 |
| 365 | HIGASHIUE S | 9 | 0.596 |
| 366 | HIPPERT R | 9 | 0.596 |
| 367 | HIRAMATSU N | 9 | 0.596 |
| 368 | HISSA M | 9 | 0.596 |
| 369 | HOFFMAN K | 9 | 0.596 |
| 370 | HOJO F | 9 | 0.596 |
| 371 | HOLLANDERS G | 9 | 0.596 |
| 372 | HONG T | 9 | 0.596 |
| 373 | HOOGSLAG P | 9 | 0.596 |
| 374 | HOUCHIN V | 9 | 0.596 |
| 375 | HOWARD T | 9 | 0.596 |
| 376 | HUANG C | 9 | 0.596 |
| 377 | HUFFMAN D | 9 | 0.596 |
| 378 | HUNTLEY R | 9 | 0.596 |
| 379 | HURLEY D | 9 | 0.596 |
| 380 | HURTIG U | 9 | 0.596 |
| 381 | HWANG J | 9 | 0.596 |
| 382 | IABLUCHANSKYI M | 9 | 0.596 |
| 383 | IGLESIAS M | 9 | 0.596 |
| 384 | IGLESIAS R | 9 | 0.596 |
| 385 | IMRAN SA | 9 | 0.596 |
| 386 | IP T | 9 | 0.596 |
| 387 | ISTRATOAIE O | 9 | 0.596 |
| 388 | IZUMINO K | 9 | 0.596 |
| 389 | JACKS R | 9 | 0.596 |
| 390 | JAFFE A | 9 | 0.596 |
| 391 | JAIN M | 9 | 0.596 |
| 392 | JAIN S | 9 | 0.596 |
| 393 | JAKOVLEV U | 9 | 0.596 |
| 394 | JARAMILLO N | 9 | 0.596 |
| 395 | JARAMILLO PL | 9 | 0.596 |
| 396 | JARDULA M | 9 | 0.596 |
| 397 | JAVASHVILI L | 9 | 0.596 |
| 398 | JEONG J | 9 | 0.596 |
| 399 | JEONG M | 9 | 0.596 |
| 400 | JI LN | 9 | 0.596 |
| 401 | JOHANSSON PA | 9 | 0.596 |
| 402 | JUAREZ M | 9 | 0.596 |
| 403 | JUHASZ F | 9 | 0.596 |
| 404 | JUHL H | 9 | 0.596 |
| 405 | JUNCHAYA JG | 9 | 0.596 |
| 406 | JUWANA Y | 9 | 0.596 |
| 407 | KAEWSUWANNA P | 9 | 0.596 |
| 408 | KANG K | 9 | 0.596 |
| 409 | KARAMITSOS K | 9 | 0.596 |
| 410 | KARIM S | 9 | 0.596 |
| 411 | KARLSSON T | 9 | 0.596 |
| 412 | KASER S | 9 | 0.596 |
| 413 | KASPER J | 9 | 0.596 |
| 414 | KAVEH K | 9 | 0.596 |
| 415 | KAWAMITSU K | 9 | 0.596 |
| 416 | KAYNE D | 9 | 0.596 |
| 417 | KEREIAKES D | 9 | 0.596 |
| 418 | KERSTEIN H | 9 | 0.596 |
| 419 | KEYMEULEN B | 9 | 0.596 |
| 420 | KHAN B | 9 | 0.596 |
| 421 | KHETAGUROVA F | 9 | 0.596 |
| 422 | KHOKHLOV A | 9 | 0.596 |
| 423 | KHOMASURIDZE A | 9 | 0.596 |
| 424 | KIM D | 9 | 0.596 |
| 425 | KNOPKE C | 9 | 0.596 |
| 426 | KOCKAERTS Y | 9 | 0.596 |
| 427 | KONIECZNY M | 9 | 0.596 |
| 428 | KOPPEL W | 9 | 0.596 |
| 429 | KOVALYOVA O | 9 | 0.596 |
| 430 | KOWALOFF E | 9 | 0.596 |
| 431 | KOWALYK S | 9 | 0.596 |
| 432 | KRAGTEN J | 9 | 0.596 |
| 433 | KRARUP T | 9 | 0.596 |
| 434 | KRASILNIKOVA E | 9 | 0.596 |
| 435 | KREBS J | 9 | 0.596 |
| 436 | KREKELS M | 9 | 0.596 |
| 437 | KRIANGSAK P | 9 | 0.596 |
| 438 | KRZYZAGORSKA E | 9 | 0.596 |
| 439 | KULKARNI A | 9 | 0.596 |
| 440 | KURASHVILI G | 9 | 0.596 |
| 441 | KURASHVILI R | 9 | 0.596 |
| 442 | KUS W | 9 | 0.596 |
| 443 | LAGRUTTA M | 9 | 0.596 |
| 444 | LAI W | 9 | 0.596 |
| 445 | LALIOTIS A | 9 | 0.596 |
| 446 | LAMKANFI F | 9 | 0.596 |
| 447 | LANNO R | 9 | 0.596 |
| 448 | LANTSEVA O | 9 | 0.596 |
| 449 | LEHMAN R | 9 | 0.596 |
| 450 | LEITAO A | 9 | 0.596 |
| 451 | LEMARIE B | 9 | 0.596 |
| 452 | LEMIS P | 9 | 0.596 |
| 453 | LERMAN S | 9 | 0.596 |
| 454 | LEVINS P | 9 | 0.596 |
| 455 | LEVINSON L | 9 | 0.596 |
| 456 | LEWY-ALTERBAUM L | 9 | 0.596 |
| 457 | LIBOV I | 9 | 0.596 |
| 458 | LICHIARDOPOL R | 9 | 0.596 |
| 459 | LIENART F | 9 | 0.596 |
| 460 | LIHN AS | 9 | 0.596 |
| 461 | LIM D | 9 | 0.596 |
| 462 | LIMA M | 9 | 0.596 |
| 463 | LINARES J | 9 | 0.596 |
| 464 | LINDENBAUM J | 9 | 0.596 |
| 465 | LING K | 9 | 0.596 |
| 466 | LISS J | 9 | 0.596 |
| 467 | LISSON R | 9 | 0.596 |
| 468 | LIU J | 9 | 0.596 |
| 469 | LIWAG A | 9 | 0.596 |
| 470 | LLORENTE I | 9 | 0.596 |
| 471 | LOCHNAN H | 9 | 0.596 |
| 472 | LOCHORN G | 9 | 0.596 |
| 473 | LOMBAARD J | 9 | 0.596 |
| 474 | LOMINADZE Z | 9 | 0.596 |
| 475 | LONGSHAW K | 9 | 0.596 |
| 476 | LOZANO MT | 9 | 0.596 |
| 477 | LOZNO HY | 9 | 0.596 |
| 478 | LU Y | 9 | 0.596 |
| 479 | LUBI M | 9 | 0.596 |
| 480 | LUGER A | 9 | 0.596 |
| 481 | LUND P | 9 | 0.596 |
| 482 | MABIRE P | 9 | 0.596 |
| 483 | MACADAMS M | 9 | 0.596 |
| 484 | MACH M | 9 | 0.596 |
| 485 | MACHKOVA M | 9 | 0.596 |
| 486 | MACRURY S | 9 | 0.596 |
| 487 | MAFFEI L | 9 | 0.596 |
| 488 | MAGEE M | 9 | 0.596 |
| 489 | MAHOOD K | 9 | 0.596 |
| 490 | MALANO J | 9 | 0.596 |
| 491 | MALDONADO AA | 9 | 0.596 |
| 492 | MALDONADO N | 9 | 0.596 |
| 493 | MANENTI E | 9 | 0.596 |
| 494 | MANES C | 9 | 0.596 |
| 495 | MANGKLABRUKS A | 9 | 0.596 |
| 496 | MANNUCCI E | 9 | 0.596 |
| 497 | MARANDI T | 9 | 0.596 |
| 498 | MARCHIONNI N | 9 | 0.596 |
| 499 | MARIANO H | 9 | 0.596 |
| 500 | MARSILII A | 9 | 0.596 |
| 501 | MATFIN G | 9 | 0.596 |
| 502 | MAW K | 9 | 0.596 |
| 503 | MAYFIELD R | 9 | 0.596 |
| 504 | MAYNARD B | 9 | 0.596 |
| 505 | MAZEN E | 9 | 0.596 |
| 506 | MCKNIGHT J | 9 | 0.596 |
| 507 | MEDAGAMA U | 9 | 0.596 |
| 508 | MELESHKEVICH T | 9 | 0.596 |
| 509 | METREVELI D | 9 | 0.596 |
| 510 | MEVISSEN H | 9 | 0.596 |
| 511 | MEZZETTI A | 9 | 0.596 |
| 512 | MIDDLETON A | 9 | 0.596 |
| 513 | MILITARU C | 9 | 0.596 |
| 514 | MIRANDA F | 9 | 0.596 |
| 515 | MIRANDA-PALMA B | 9 | 0.596 |
| 516 | MIROSEVIC G | 9 | 0.596 |
| 517 | MITHA E | 9 | 0.596 |
| 518 | MOHAMED WW | 9 | 0.596 |
| 519 | MOKSHAGUNDAM S | 9 | 0.596 |
| 520 | MOLTER D | 9 | 0.596 |
| 521 | MONNO S | 9 | 0.596 |
| 522 | MONTE O | 9 | 0.596 |
| 523 | MOODLEY R | 9 | 0.596 |
| 524 | MORAWSKI E | 9 | 0.596 |
| 525 | MORENO B | 9 | 0.596 |
| 526 | MORO E | 9 | 0.596 |
| 527 | MORTELMANS J | 9 | 0.596 |
| 528 | MOSELY J | 9 | 0.596 |
| 529 | MULDER H | 9 | 0.596 |
| 530 | MUNOZ EC | 9 | 0.596 |
| 531 | MURPHY SA | 9 | 0.596 |
| 532 | MUSTAFA N | 9 | 0.596 |
| 533 | NADAREISHVILI L | 9 | 0.596 |
| 534 | NAGY K | 9 | 0.596 |
| 535 | NAIDOO D | 9 | 0.596 |
| 536 | NAIDOO V | 9 | 0.596 |
| 537 | NAIDU J | 9 | 0.596 |
| 538 | NASSIM O | 9 | 0.596 |
| 539 | NAVARRO J | 9 | 0.596 |
| 540 | NICASIO J | 9 | 0.596 |
| 541 | NIEROP P | 9 | 0.596 |
| 542 | NIKOLAEV K | 9 | 0.596 |
| 543 | O'BRIEN I | 9 | 0.596 |
| 544 | OBIEKWE O | 9 | 0.596 |
| 545 | OBREZAN A | 9 | 0.596 |
| 546 | OCICKA-KOZAKIEWICZ A | 9 | 0.596 |
| 547 | OGOREK M | 9 | 0.596 |
| 548 | OKUBO M | 9 | 0.596 |
| 549 | OLDENBURG-LIGTENBERG P | 9 | 0.596 |
| 550 | OLSOVSKY J | 9 | 0.596 |
| 551 | ONISHI Y | 9 | 0.596 |
| 552 | ORLOWSKA-KUNIKOWSKA E | 9 | 0.596 |
| 553 | ORR R | 9 | 0.596 |
| 554 | OSEA E | 9 | 0.596 |
| 555 | OSKIN T | 9 | 0.596 |
| 556 | OSORES JL | 9 | 0.596 |
| 557 | OUELLETT A | 9 | 0.596 |
| 558 | PACORA FF | 9 | 0.596 |
| 559 | PAINE W | 9 | 0.596 |
| 560 | PANDEY A | 9 | 0.596 |
| 561 | PANELO A | 9 | 0.596 |
| 562 | PAPPAS S | 9 | 0.596 |
| 563 | PARMAR P | 9 | 0.596 |
| 564 | PATKAY J | 9 | 0.596 |
| 565 | PATRICK J | 9 | 0.596 |
| 566 | PAULWEBER B | 9 | 0.596 |
| 567 | PEARSON E | 9 | 0.596 |
| 568 | PENISTON J | 9 | 0.596 |
| 569 | PERALTA FG | 9 | 0.596 |
| 570 | PEREG V | 9 | 0.596 |
| 571 | PEREZ G | 9 | 0.596 |
| 572 | PERSU A | 9 | 0.596 |
| 573 | PETIT C | 9 | 0.596 |
| 574 | PETROV A | 9 | 0.596 |
| 575 | PETUNINA N | 9 | 0.596 |
| 576 | PIADITIS G | 9 | 0.596 |
| 577 | PIESIEWICZ W | 9 | 0.596 |
| 578 | PILLAI M | 9 | 0.596 |
| 579 | PINTO L | 9 | 0.596 |
| 580 | PISH R | 9 | 0.596 |
| 581 | PIYAYOTAI D | 9 | 0.596 |
| 582 | PLUTO T | 9 | 0.596 |
| 583 | PODGORSKI G | 9 | 0.596 |
| 584 | POP L | 9 | 0.596 |
| 585 | POTEMKIN V | 9 | 0.596 |
| 586 | PRAGER R | 9 | 0.596 |
| 587 | PRAMONO B | 9 | 0.596 |
| 588 | PRAWER J | 9 | 0.596 |
| 589 | PREMCHAND R | 9 | 0.596 |
| 590 | PRETORIUS M | 9 | 0.596 |
| 591 | PURISCH S | 9 | 0.596 |
| 592 | QUEIROS J | 9 | 0.596 |
| 593 | RAMONCITO H | 9 | 0.596 |
| 594 | RASSI N | 9 | 0.596 |
| 595 | RAWLS R | 9 | 0.596 |
| 596 | REA R | 9 | 0.596 |
| 597 | REDDY S | 9 | 0.596 |
| 598 | REED J | 9 | 0.596 |
| 599 | REEVES M | 9 | 0.596 |
| 600 | REGGIANI GM | 9 | 0.596 |
| 601 | REMAUD P | 9 | 0.596 |
| 602 | RHA S | 9 | 0.596 |
| 603 | RICHARDS R | 9 | 0.596 |
| 604 | RICHWINE R | 9 | 0.596 |
| 605 | RIGLA M | 9 | 0.596 |
| 606 | RILEY E | 9 | 0.596 |
| 607 | RISER J | 9 | 0.596 |
| 608 | RITA H | 9 | 0.596 |
| 609 | ROBERSON K | 9 | 0.596 |
| 610 | RODEROS O | 9 | 0.596 |
| 611 | ROGADO C | 9 | 0.596 |
| 612 | ROJAS WG | 9 | 0.596 |
| 613 | RONNER E | 9 | 0.596 |
| 614 | ROODT A | 9 | 0.596 |
| 615 | ROSALES R | 9 | 0.596 |
| 616 | ROSENFELD J | 9 | 0.596 |
| 617 | ROSSING P | 9 | 0.596 |
| 618 | ROSSOLKO L | 9 | 0.596 |
| 619 | ROTHMAN J | 9 | 0.596 |
| 620 | SABAN J | 9 | 0.596 |
| 621 | SADHU N | 9 | 0.596 |
| 622 | SAKSONO HD | 9 | 0.596 |
| 623 | SALAMON C | 9 | 0.596 |
| 624 | SALGADO V | 9 | 0.596 |
| 625 | SALLES JN | 9 | 0.596 |
| 626 | SANCHEZ G | 9 | 0.596 |
| 627 | SANDBERG M | 9 | 0.596 |
| 628 | SANDERS D | 9 | 0.596 |
| 629 | SANTINI M | 9 | 0.596 |
| 630 | SARAIVA J | 9 | 0.596 |
| 631 | SATARASINGHE R | 9 | 0.596 |
| 632 | SAXMAN K | 9 | 0.596 |
| 633 | SCHAPER N | 9 | 0.596 |
| 634 | SCHEAR M | 9 | 0.596 |
| 635 | SCHEEN A | 9 | 0.596 |
| 636 | SCHIFF E | 9 | 0.596 |
| 637 | SCHMID H | 9 | 0.596 |
| 638 | SCHNACK C | 9 | 0.596 |
| 639 | SEGURA J | 9 | 0.596 |
| 640 | SEIDMAN B | 9 | 0.596 |
| 641 | SEIDNER M | 9 | 0.596 |
| 642 | SELVANAYAGAM J | 9 | 0.596 |
| 643 | SEMPLICINI A | 9 | 0.596 |
| 644 | SEUNG K | 9 | 0.596 |
| 645 | SGARBI J | 9 | 0.596 |
| 646 | SHAH S | 9 | 0.596 |
| 647 | SHAMANNA P | 9 | 0.596 |
| 648 | SHANDILYA L | 9 | 0.596 |
| 649 | SHAPIRO J | 9 | 0.596 |
| 650 | SHEPARD M | 9 | 0.596 |
| 651 | SHIN M | 9 | 0.596 |
| 652 | SHOMALI M | 9 | 0.596 |
| 653 | SILVA A | 9 | 0.596 |
| 654 | SISWANTO B | 9 | 0.596 |
| 655 | SITAR S | 9 | 0.596 |
| 656 | SIU S | 9 | 0.596 |
| 657 | SIYAMBALAPITIYA S | 9 | 0.596 |
| 658 | SKOKOWSKA E | 9 | 0.596 |
| 659 | SOFLEY C | 9 | 0.596 |
| 660 | SOLDYSHEV R | 9 | 0.596 |
| 661 | SOMASUNDARAM N | 9 | 0.596 |
| 662 | SORIA MG | 9 | 0.596 |
| 663 | SOUFER J | 9 | 0.596 |
| 664 | SPARBY J | 9 | 0.596 |
| 665 | SPYRA J | 9 | 0.596 |
| 666 | ST AMOUR E | 9 | 0.596 |
| 667 | STORMS G | 9 | 0.596 |
| 668 | STRIVAY M | 9 | 0.596 |
| 669 | STROGER JH | 9 | 0.596 |
| 670 | STROUT C | 9 | 0.596 |
| 671 | SUAREZ C | 9 | 0.596 |
| 672 | SUM C | 9 | 0.596 |
| 673 | SUWANWALAIKORN S | 9 | 0.596 |
| 674 | SUZUKI A | 9 | 0.596 |
| 675 | SY R | 9 | 0.596 |
| 676 | SYED M | 9 | 0.596 |
| 677 | SZENTPETERI I | 9 | 0.596 |
| 678 | TABAK A | 9 | 0.596 |
| 679 | TAHK S | 9 | 0.596 |
| 680 | TAMAS G | 9 | 0.596 |
| 681 | TAMAYO R | 9 | 0.596 |
| 682 | TAN R | 9 | 0.596 |
| 683 | TANGGO Y | 9 | 0.596 |
| 684 | TARASOV N | 9 | 0.596 |
| 685 | TARNOW L | 9 | 0.596 |
| 686 | TEIXEIRA J | 9 | 0.596 |
| 687 | TELTSER M | 9 | 0.596 |
| 688 | TIBURCIO A | 9 | 0.596 |
| 689 | TOLEDO R | 9 | 0.596 |
| 690 | TOPLAK H | 9 | 0.596 |
| 691 | TORRES CD | 9 | 0.596 |
| 692 | TOSCANO V | 9 | 0.596 |
| 693 | TRESCOLI C | 9 | 0.596 |
| 694 | TSANG C | 9 | 0.596 |
| 695 | TUSEK S | 9 | 0.596 |
| 696 | UBANI A | 9 | 0.596 |
| 697 | ULLA M | 9 | 0.596 |
| 698 | ULLAL J | 9 | 0.596 |
| 699 | UR E | 9 | 0.596 |
| 700 | URHAMMER S | 9 | 0.596 |
| 701 | URINA M | 9 | 0.596 |
| 702 | UWAIFO G | 9 | 0.596 |
| 703 | VAN BEMMEL T | 9 | 0.596 |
| 704 | VAN DE BORNE P | 9 | 0.596 |
| 705 | VAN SOEST J | 9 | 0.596 |
| 706 | VAN ZYL L | 9 | 0.596 |
| 707 | VANTROYEN D | 9 | 0.596 |
| 708 | VARZIC SC | 9 | 0.596 |
| 709 | VAWDA H | 9 | 0.596 |
| 710 | VELAZQUEZ F | 9 | 0.596 |
| 711 | VELAZQUEZ MV | 9 | 0.596 |
| 712 | VELEZ M | 9 | 0.596 |
| 713 | VENTER T | 9 | 0.596 |
| 714 | VERCAMMEN C | 9 | 0.596 |
| 715 | VEREECKEN G | 9 | 0.596 |
| 716 | VERHOEVEN R | 9 | 0.596 |
| 717 | VIDAL J | 9 | 0.596 |
| 718 | VIERGEVER P | 9 | 0.596 |
| 719 | VIJAPURKAR U | 9 | 0.596 |
| 720 | VIRSALADZE D | 9 | 0.596 |
| 721 | VOLOSHYNA O | 9 | 0.596 |
| 722 | VOROKHOBINA N | 9 | 0.596 |
| 723 | VRYONIDOU A | 9 | 0.596 |
| 724 | VYKHOVANYUK I | 9 | 0.596 |
| 725 | WAHLEN J | 9 | 0.596 |
| 726 | WAHONO SD | 9 | 0.596 |
| 727 | WAINSTEIN J | 9 | 0.596 |
| 728 | WAITMAN J | 9 | 0.596 |
| 729 | WALDRON M | 9 | 0.596 |
| 730 | WASSERMAN A | 9 | 0.596 |
| 731 | WEHMEIER K | 9 | 0.596 |
| 732 | WEINRAUCH L | 9 | 0.596 |
| 733 | WELCH M | 9 | 0.596 |
| 734 | WEN M | 9 | 0.596 |
| 735 | WHEELER DC | 9 | 0.596 |
| 736 | WILKS K | 9 | 0.596 |
| 737 | WITTMER B | 9 | 0.596 |
| 738 | WOOD J | 9 | 0.596 |
| 739 | WYATT N | 9 | 0.596 |
| 740 | YAKHONTOVA P | 9 | 0.596 |
| 741 | YOON J | 9 | 0.596 |
| 742 | ZALEVSKAYA A | 9 | 0.596 |
| 743 | ZANELLA M | 9 | 0.596 |
| 744 | ZAPATA L | 9 | 0.596 |
| 745 | ZATEYSHCHIKOV D | 9 | 0.596 |
| 746 | ZAYOUR D | 9 | 0.596 |
| 747 | ZETU C | 9 | 0.596 |
| 748 | ZMUDA W | 9 | 0.596 |
| 749 | ZOTOV S | 9 | 0.596 |
| 750 | ZWIERS G | 9 | 0.596 |
| 751 | BONACA MP | 8 | 0.53 |
| 752 | BOULTON DW | 8 | 0.53 |
| 753 | BUSE JB | 8 | 0.53 |
| 754 | CANNON CP | 8 | 0.53 |
| 755 | CERIELLO A | 8 | 0.53 |
| 756 | DE BOER RA | 8 | 0.53 |
| 757 | ERIKSSON JW | 8 | 0.53 |
| 758 | GAUSE-NILSSON I | 8 | 0.53 |
| 759 | JANUZZI JL | 8 | 0.53 |
| 760 | KAKU K | 8 | 0.53 |
| 761 | KITAKAZE M | 8 | 0.53 |
| 762 | KOBER L | 8 | 0.53 |
| 763 | LACHIN JM | 8 | 0.53 |
| 764 | LI Q | 8 | 0.53 |
| 765 | PETRIE MC | 8 | 0.53 |
| 766 | POLLOCK C | 8 | 0.53 |
| 767 | SHARMA A | 8 | 0.53 |
| 768 | VAN RAALTE DH | 8 | 0.53 |
| 769 | ATHYROS VG | 7 | 0.464 |
| 770 | BELLASTELLA G | 7 | 0.464 |
| 771 | BENGTSSON O | 7 | 0.464 |
| 772 | BERGENHEIM K | 7 | 0.464 |
| 773 | BIRKELAND KI | 7 | 0.464 |
| 774 | BJORNSTAD P | 7 | 0.464 |
| 775 | BRUECKMANN M | 7 | 0.464 |
| 776 | CAPUANO G | 7 | 0.464 |
| 777 | EVANS M | 7 | 0.464 |
| 778 | GAUSE-NILSSON IAM | 7 | 0.464 |
| 779 | GEORGE J | 7 | 0.464 |
| 780 | GILBERT RE | 7 | 0.464 |
| 781 | HAMMAR N | 7 | 0.464 |
| 782 | HERNANDEZ AF | 7 | 0.464 |
| 783 | IMPRIALOS K | 7 | 0.464 |
| 784 | JHUND PS | 7 | 0.464 |
| 785 | KARAGIANNIS A | 7 | 0.464 |
| 786 | KASPERS S | 7 | 0.464 |
| 787 | KATO ET | 7 | 0.464 |
| 788 | KATSIKI N | 7 | 0.464 |
| 789 | KOSIBOROD MN | 7 | 0.464 |
| 790 | LAM CSP | 7 | 0.464 |
| 791 | LIM S | 7 | 0.464 |
| 792 | LYTVYN Y | 7 | 0.464 |
| 793 | MAIORINO MI | 7 | 0.464 |
| 794 | MARTINEZ FA | 7 | 0.464 |
| 795 | MCEWAN P | 7 | 0.464 |
| 796 | SJOSTRAND M | 7 | 0.464 |
| 797 | SOLINI A | 7 | 0.464 |
| 798 | ZELLER C | 7 | 0.464 |
| 799 | ABDUL-GHANI M | 6 | 0.398 |
| 800 | BADIMON JJ | 6 | 0.398 |
| 801 | BAJAJ HS | 6 | 0.398 |
| 802 | BAKRIS GL | 6 | 0.398 |
| 803 | BOHM M | 6 | 0.398 |
| 804 | CEFALU WT | 6 | 0.398 |
| 805 | CONNELLY KA | 6 | 0.398 |
| 806 | COOPER ME | 6 | 0.398 |
| 807 | DE NICOLA L | 6 | 0.398 |
| 808 | DEMETS DL | 6 | 0.398 |
| 809 | GREEN JB | 6 | 0.398 |
| 810 | KALRA S | 6 | 0.398 |
| 811 | LIAKOS A | 6 | 0.398 |
| 812 | MAZER CD | 6 | 0.398 |
| 813 | MCCULLOUGH PA | 6 | 0.398 |
| 814 | MERKELY B | 6 | 0.398 |
| 815 | NEUEN BL | 6 | 0.398 |
| 816 | PERSSON F | 6 | 0.398 |
| 817 | PTASZYNSKA A | 6 | 0.398 |
| 818 | ROSENTHAL N | 6 | 0.398 |
| 819 | RYDEN L | 6 | 0.398 |
| 820 | SAHEBKAR A | 6 | 0.398 |
| 821 | SANTOS-GALLEGO CG | 6 | 0.398 |
| 822 | SATO Y | 6 | 0.398 |
| 823 | TAKASU T | 6 | 0.398 |
| 824 | TAKEDA Y | 6 | 0.398 |
| 825 | TANG FM | 6 | 0.398 |
| 826 | UDELL JA | 6 | 0.398 |
| 827 | UTSUNOMIYA K | 6 | 0.398 |
| 828 | VADUGANATHAN M | 6 | 0.398 |
| 829 | VERCRUYSSE F | 6 | 0.398 |
| 830 | XU J | 6 | 0.398 |
| 831 | ZACCARDI F | 6 | 0.398 |
| 832 | ZANNAD F | 6 | 0.398 |
| 833 | AGARWAL R | 5 | 0.331 |
| 834 | ATKIN SL | 5 | 0.331 |
| 835 | BAILEY CJ | 5 | 0.331 |
| 836 | BAKRIS G | 5 | 0.331 |
| 837 | BEKIARI E | 5 | 0.331 |
| 838 | BONORA BM | 5 | 0.331 |
| 839 | CARBONE S | 5 | 0.331 |
| 840 | CARIOU B | 5 | 0.331 |
| 841 | CHAN SP | 5 | 0.331 |
| 842 | CHARYTAN DM | 5 | 0.331 |
| 843 | CHERNEY D | 5 | 0.331 |
| 844 | CHIANG CE | 5 | 0.331 |
| 845 | DE PONTI F | 5 | 0.331 |
| 846 | DEKKERS CCJ | 5 | 0.331 |
| 847 | DEL PRATO S | 5 | 0.331 |
| 848 | DIXON DL | 5 | 0.331 |
| 849 | ECKEL RH | 5 | 0.331 |
| 850 | ELISAF MS | 5 | 0.331 |
| 851 | ERONDU N | 5 | 0.331 |
| 852 | FERRANNINI E | 5 | 0.331 |
| 853 | FILIPPATOS G | 5 | 0.331 |
| 854 | FILIPPATOS TD | 5 | 0.331 |
| 855 | FONAROW GC | 5 | 0.331 |
| 856 | FREDRIKSSON M | 5 | 0.331 |
| 857 | FURTADO RHM | 5 | 0.331 |
| 858 | GARCIA-ROPERO A | 5 | 0.331 |
| 859 | GOLDBERG IJ | 5 | 0.331 |
| 860 | GOLDENBERG RM | 5 | 0.331 |
| 861 | GONZALEZ-JUANATEY JR | 5 | 0.331 |
| 862 | GORRIZ JL | 5 | 0.331 |
| 863 | GREASLEY PJ | 5 | 0.331 |
| 864 | GREENE T | 5 | 0.331 |
| 865 | HARDY E | 5 | 0.331 |
| 866 | ITO H | 5 | 0.331 |
| 867 | ITO Y | 5 | 0.331 |
| 868 | JORDAN J | 5 | 0.331 |
| 869 | JORGENSEN ME | 5 | 0.331 |
| 870 | KO SH | 5 | 0.331 |
| 871 | KOITKA-WEBER A | 5 | 0.331 |
| 872 | LEE BW | 5 | 0.331 |
| 873 | LEE J | 5 | 0.331 |
| 874 | LEVIN A | 5 | 0.331 |
| 875 | LI J | 5 | 0.331 |
| 876 | LINGVAY I | 5 | 0.331 |
| 877 | LOVSHIN JA | 5 | 0.331 |
| 878 | LUND SS | 5 | 0.331 |
| 879 | MACHA S | 5 | 0.331 |
| 880 | MENTZ RJ | 5 | 0.331 |
| 881 | NATHANSON D | 5 | 0.331 |
| 882 | NEELAND IJ | 5 | 0.331 |
| 883 | NICOLAU JC | 5 | 0.331 |
| 884 | NISHIYAMA A | 5 | 0.331 |
| 885 | NODE K | 5 | 0.331 |
| 886 | NYSTROM T | 5 | 0.331 |
| 887 | ORTIZ A | 5 | 0.331 |
| 888 | PARIKH S | 5 | 0.331 |
| 889 | PERKINS BA | 5 | 0.331 |
| 890 | PFARR E | 5 | 0.331 |
| 891 | RANGASWAMI J | 5 | 0.331 |
| 892 | RASCHI E | 5 | 0.331 |
| 893 | SATA M | 5 | 0.331 |
| 894 | SCHNELL O | 5 | 0.331 |
| 895 | SCHOU M | 5 | 0.331 |
| 896 | SHEU WHH | 5 | 0.331 |
| 897 | SPERLING LS | 5 | 0.331 |
| 898 | STANDL E | 5 | 0.331 |
| 899 | TANAKA A | 5 | 0.331 |
| 900 | TERAUCHI Y | 5 | 0.331 |
| 901 | TERRA SG | 5 | 0.331 |
| 902 | TIKKANEN I | 5 | 0.331 |
| 903 | UEDA S | 5 | 0.331 |
| 904 | WANG Y | 5 | 0.331 |
| 905 | WATADA H | 5 | 0.331 |
| 906 | WEXLER DJ | 5 | 0.331 |
| 907 | XIE J | 5 | 0.331 |
| 908 | YARIBEYGI H | 5 | 0.331 |
| 909 | YOON KH | 5 | 0.331 |
| 910 | ZUURBIER CJ | 5 | 0.331 |
| 911 | ABBATE A | 4 | 0.265 |
| 912 | AL-OMRAN M | 4 | 0.265 |
| 913 | ARNOLD SV | 4 | 0.265 |
| 914 | ARODA VR | 4 | 0.265 |
| 915 | ARONOW WS | 4 | 0.265 |
| 916 | AROOR AR | 4 | 0.265 |
| 917 | BAARTSCHEER A | 4 | 0.265 |
| 918 | BAIN S | 4 | 0.265 |
| 919 | BAKER WL | 4 | 0.265 |
| 920 | BARNETT AH | 4 | 0.265 |
| 921 | BIRNBAUM Y | 4 | 0.265 |
| 922 | BRAUNWALD E | 4 | 0.265 |
| 923 | BULL S | 4 | 0.265 |
| 924 | CAIN V | 4 | 0.265 |
| 925 | CAVENDER MA | 4 | 0.265 |
| 926 | CHATTERJEE S | 4 | 0.265 |
| 927 | CHATTIPAKORN N | 4 | 0.265 |
| 928 | CHEN H | 4 | 0.265 |
| 929 | CHILTON RJ | 4 | 0.265 |
| 930 | CHIODINI P | 4 | 0.265 |
| 931 | CORONEL R | 4 | 0.265 |
| 932 | COSENTINO F | 4 | 0.265 |
| 933 | D'ALESSIO DA | 4 | 0.265 |
| 934 | DAVIES M | 4 | 0.265 |
| 935 | DAVIS SN | 4 | 0.265 |
| 936 | DE COSMO S | 4 | 0.265 |
| 937 | DE LUSIGNAN S | 4 | 0.265 |
| 938 | DEEDWANIA P | 4 | 0.265 |
| 939 | DEMARCO VG | 4 | 0.265 |
| 940 | DICEMBRINI I | 4 | 0.265 |
| 941 | DIMITRIADIS G | 4 | 0.265 |
| 942 | EDWARDS R | 4 | 0.265 |
| 943 | EKINCI EI | 4 | 0.265 |
| 944 | ELIASSON B | 4 | 0.265 |
| 945 | ELISAF M | 4 | 0.265 |
| 946 | FERDINAND KC | 4 | 0.265 |
| 947 | FUNG A | 4 | 0.265 |
| 948 | GHOSAL S | 4 | 0.265 |
| 949 | GIACCARI A | 4 | 0.265 |
| 950 | GOODRICH EL | 4 | 0.265 |
| 951 | HALLER H | 4 | 0.265 |
| 952 | HANEDA M | 4 | 0.265 |
| 953 | HIRATA K | 4 | 0.265 |
| 954 | HIROSE T | 4 | 0.265 |
| 955 | INAGAKI N | 4 | 0.265 |
| 956 | INOUE T | 4 | 0.265 |
| 957 | KADOWAKI T | 4 | 0.265 |
| 958 | KARAGIANNIS T | 4 | 0.265 |
| 959 | KAUL S | 4 | 0.265 |
| 960 | KIM DJ | 4 | 0.265 |
| 961 | KIM JH | 4 | 0.265 |
| 962 | KIM NH | 4 | 0.265 |
| 963 | KIMURA T | 4 | 0.265 |
| 964 | KOEPSELL H | 4 | 0.265 |
| 965 | KOMAJDA M | 4 | 0.265 |
| 966 | KUMASHIRO N | 4 | 0.265 |
| 967 | LAMBADIARI V | 4 | 0.265 |
| 968 | LAMOS EM | 4 | 0.265 |
| 969 | LIU XY | 4 | 0.265 |
| 970 | MARCHESINI G | 4 | 0.265 |
| 971 | MENOWN IBA | 4 | 0.265 |
| 972 | METRA M | 4 | 0.265 |
| 973 | MIKHAILIDIS DP | 4 | 0.265 |
| 974 | MINGRONE G | 4 | 0.265 |
| 975 | MONAMI M | 4 | 0.265 |
| 976 | MUNRO N | 4 | 0.265 |
| 977 | O'MEARA E | 4 | 0.265 |
| 978 | OFSTAD AP | 4 | 0.265 |
| 979 | OHKUMA T | 4 | 0.265 |
| 980 | OYAMA J | 4 | 0.265 |
| 981 | PAPANAS N | 4 | 0.265 |
| 982 | PARK SH | 4 | 0.265 |
| 983 | PERRONE-FILARDI P | 4 | 0.265 |
| 984 | POLUZZI E | 4 | 0.265 |
| 985 | RADHOLM K | 4 | 0.265 |
| 986 | RAHMAN A | 4 | 0.265 |
| 987 | ROZENBERG A | 4 | 0.265 |
| 988 | SARTIPY P | 4 | 0.265 |
| 989 | SATO T | 4 | 0.265 |
| 990 | SCHNEEWEISS S | 4 | 0.265 |
| 991 | SCIRICA BM | 4 | 0.265 |
| 992 | SEFEROVIC PM | 4 | 0.265 |
| 993 | SESTI G | 4 | 0.265 |
| 994 | SHESTAKOVA MV | 4 | 0.265 |
| 995 | SHIGIYAMA F | 4 | 0.265 |
| 996 | SILVERMAN MG | 4 | 0.265 |
| 997 | SINGH R | 4 | 0.265 |
| 998 | SINHA B | 4 | 0.265 |
| 999 | SOLER MJ | 4 | 0.265 |
| 1000 | SPOSITO AC | 4 | 0.265 |
| 1001 | STEFANSSON BV | 4 | 0.265 |
| 1002 | TAGUCHI I | 4 | 0.265 |
| 1003 | TANAKA H | 4 | 0.265 |
| 1004 | THOMSON SC | 4 | 0.265 |
| 1005 | TOMIYAMA H | 4 | 0.265 |
| 1006 | TOTO RD | 4 | 0.265 |
| 1007 | TSCHOPE C | 4 | 0.265 |
| 1008 | TSIMIHODIMOS V | 4 | 0.265 |
| 1009 | USTYUGOVA A | 4 | 0.265 |
| 1010 | WILDING J | 4 | 0.265 |
| 1011 | WITTBRODT E | 4 | 0.265 |
| 1012 | YAMADA H | 4 | 0.265 |
| 1013 | YAMADA M | 4 | 0.265 |
| 1014 | YAMAMOTO H | 4 | 0.265 |
| 1015 | YANUV I | 4 | 0.265 |
| 1016 | YAVIN Y | 4 | 0.265 |
| 1017 | YE YM | 4 | 0.265 |
| 1018 | ZHANG H | 4 | 0.265 |
| 1019 | ZHANG Y | 4 | 0.265 |
| 1020 | ZHOU Z | 4 | 0.265 |
| 1021 | AHMED F | 3 | 0.199 |
| 1022 | ALBIERO M | 3 | 0.199 |
| 1023 | ALICIC RZ | 3 | 0.199 |
| 1024 | ANAGNOSTIS P | 3 | 0.199 |
| 1025 | ANDREADOU I | 3 | 0.199 |
| 1026 | ARONSON R | 3 | 0.199 |
| 1027 | ATSUMI T | 3 | 0.199 |
| 1028 | BAJAJ M | 3 | 0.199 |
| 1029 | BAKER J | 3 | 0.199 |
| 1030 | BANDYOPADHYAY D | 3 | 0.199 |
| 1031 | BAUERSACHS J | 3 | 0.199 |
| 1032 | BELL DSH | 3 | 0.199 |
| 1033 | BELOHLAVEK J | 3 | 0.199 |
| 1034 | BENNETT H | 3 | 0.199 |
| 1035 | BLUHMKI E | 3 | 0.199 |
| 1036 | BOMPOINT S | 3 | 0.199 |
| 1037 | BONADONNA R | 3 | 0.199 |
| 1038 | BONORA E | 3 | 0.199 |
| 1039 | BOOKHART B | 3 | 0.199 |
| 1040 | BOUTARI C | 3 | 0.199 |
| 1041 | BOWES CD | 3 | 0.199 |
| 1042 | BRENNER BM | 3 | 0.199 |
| 1043 | CAI XL | 3 | 0.199 |
| 1044 | CANANI LH | 3 | 0.199 |
| 1045 | CHAN YY | 3 | 0.199 |
| 1046 | CHANG KC | 3 | 0.199 |
| 1047 | CHEN LM | 3 | 0.199 |
| 1048 | CHENG Y | 3 | 0.199 |
| 1049 | CHERNEY DZ | 3 | 0.199 |
| 1050 | CHERTOW GM | 3 | 0.199 |
| 1051 | CHOPRA VK | 3 | 0.199 |
| 1052 | CONSOLI A | 3 | 0.199 |
| 1053 | CUMMINGS DM | 3 | 0.199 |
| 1054 | DE BRUIN TWA | 3 | 0.199 |
| 1055 | DELANAYE P | 3 | 0.199 |
| 1056 | DENG HW | 3 | 0.199 |
| 1057 | DEROSA G | 3 | 0.199 |
| 1058 | DESAI AS | 3 | 0.199 |
| 1059 | DEVORE AD | 3 | 0.199 |
| 1060 | DHALWANI NN | 3 | 0.199 |
| 1061 | DIEZ M | 3 | 0.199 |
| 1062 | DOCHERTY KF | 3 | 0.199 |
| 1063 | DUKAT A | 3 | 0.199 |
| 1064 | DURKIN M | 3 | 0.199 |
| 1065 | ELEFTHERIADOU I | 3 | 0.199 |
| 1066 | EZEKOWITZ JA | 3 | 0.199 |
| 1067 | FABER J | 3 | 0.199 |
| 1068 | FARKOUH ME | 3 | 0.199 |
| 1069 | FEHER M | 3 | 0.199 |
| 1070 | FERRANNINI G | 3 | 0.199 |
| 1071 | FILIPPAS-NTEKOUAN S | 3 | 0.199 |
| 1072 | FILIPPATOS GS | 3 | 0.199 |
| 1073 | FIOLET JWT | 3 | 0.199 |
| 1074 | FITCHETT DH | 3 | 0.199 |
| 1075 | FRIAS JP | 3 | 0.199 |
| 1076 | FU AZ | 3 | 0.199 |
| 1077 | FUKUDA T | 3 | 0.199 |
| 1078 | GALLO S | 3 | 0.199 |
| 1079 | GANSEVOORT RT | 3 | 0.199 |
| 1080 | GHOSH RK | 3 | 0.199 |
| 1081 | GIANNETTI N | 3 | 0.199 |
| 1082 | GIORGINO F | 3 | 0.199 |
| 1083 | GNUDI L | 3 | 0.199 |
| 1084 | GOLDENBERG R | 3 | 0.199 |
| 1085 | GONCALVES E | 3 | 0.199 |
| 1086 | GOYAL A | 3 | 0.199 |
| 1087 | GRAY LJ | 3 | 0.199 |
| 1088 | GROOP PH | 3 | 0.199 |
| 1089 | GUPTA A | 3 | 0.199 |
| 1090 | HALLOW KM | 3 | 0.199 |
| 1091 | HANCU N | 3 | 0.199 |
| 1092 | HANDELSMAN Y | 3 | 0.199 |
| 1093 | HANIF W | 3 | 0.199 |
| 1094 | HASEGAWA Y | 3 | 0.199 |
| 1095 | HITOMI H | 3 | 0.199 |
| 1096 | HOLL RW | 3 | 0.199 |
| 1097 | HOLLMANN MW | 3 | 0.199 |
| 1098 | HOLMAN RR | 3 | 0.199 |
| 1099 | HUNG MJ | 3 | 0.199 |
| 1100 | HUR KY | 3 | 0.199 |
| 1101 | HUSAIN M | 3 | 0.199 |
| 1102 | HUSSEIN H | 3 | 0.199 |
| 1103 | HWANG SJ | 3 | 0.199 |
| 1104 | IIJIMA H | 3 | 0.199 |
| 1105 | IMPRIALOS KP | 3 | 0.199 |
| 1106 | INZUCCHI S | 3 | 0.199 |
| 1107 | ITO S | 3 | 0.199 |
| 1108 | JACOBS-CACHA C | 3 | 0.199 |
| 1109 | JAIN D | 3 | 0.199 |
| 1110 | JAMAL W | 3 | 0.199 |
| 1111 | JARDINE M | 3 | 0.199 |
| 1112 | JIA XM | 3 | 0.199 |
| 1113 | JOHANSON P | 3 | 0.199 |
| 1114 | JOHNSON A | 3 | 0.199 |
| 1115 | KANEKO M | 3 | 0.199 |
| 1116 | KANG S | 3 | 0.199 |
| 1117 | KARASIK A | 3 | 0.199 |
| 1118 | KARIO K | 3 | 0.199 |
| 1119 | KASHIWAGI A | 3 | 0.199 |
| 1120 | KATZ A | 3 | 0.199 |
| 1121 | KIM G | 3 | 0.199 |
| 1122 | KIM HJ | 3 | 0.199 |
| 1123 | KIM M | 3 | 0.199 |
| 1124 | KIM S | 3 | 0.199 |
| 1125 | KIM SG | 3 | 0.199 |
| 1126 | KIM-MITSUYAMA S | 3 | 0.199 |
| 1127 | KIMURA K | 3 | 0.199 |
| 1128 | KLUGER AY | 3 | 0.199 |
| 1129 | KNOP FK | 3 | 0.199 |
| 1130 | KOBAYASHI K | 3 | 0.199 |
| 1131 | KOHSAKA S | 3 | 0.199 |
| 1132 | KOIBUCHI N | 3 | 0.199 |
| 1133 | KURIHARA Y | 3 | 0.199 |
| 1134 | KWON HS | 3 | 0.199 |
| 1135 | KWON MJ | 3 | 0.199 |
| 1136 | LAI ECC | 3 | 0.199 |
| 1137 | LANG CC | 3 | 0.199 |
| 1138 | LAUFS U | 3 | 0.199 |
| 1139 | LAVERMAN GD | 3 | 0.199 |
| 1140 | LAVIE CJ | 3 | 0.199 |
| 1141 | LAW G | 3 | 0.199 |
| 1142 | LEE AY | 3 | 0.199 |
| 1143 | LEE MK | 3 | 0.199 |
| 1144 | LERMA EV | 3 | 0.199 |
| 1145 | LEWIS J | 3 | 0.199 |
| 1146 | LI T | 3 | 0.199 |
| 1147 | LI X | 3 | 0.199 |
| 1148 | LI XY | 3 | 0.199 |
| 1149 | LI Z | 3 | 0.199 |
| 1150 | LIEN LF | 3 | 0.199 |
| 1151 | LIEW D | 3 | 0.199 |
| 1152 | LINDENFELD J | 3 | 0.199 |
| 1153 | LIP GYH | 3 | 0.199 |
| 1154 | LIST J | 3 | 0.199 |
| 1155 | LONGO M | 3 | 0.199 |
| 1156 | LU QG | 3 | 0.199 |
| 1157 | LU YH | 3 | 0.199 |
| 1158 | MACISAAC RJ | 3 | 0.199 |
| 1159 | MAIER LS | 3 | 0.199 |
| 1160 | MANSFIELD TA | 3 | 0.199 |
| 1161 | MARTINEZ-CASTELAO A | 3 | 0.199 |
| 1162 | MARTINKA E | 3 | 0.199 |
| 1163 | MATOBA K | 3 | 0.199 |
| 1164 | MATTHEWS D | 3 | 0.199 |
| 1165 | MATTHEWS VB | 3 | 0.199 |
| 1166 | MCCRIMMON RJ | 3 | 0.199 |
| 1167 | MCQUILLAN C | 3 | 0.199 |
| 1168 | MIKI T | 3 | 0.199 |
| 1169 | MISHRIKY BM | 3 | 0.199 |
| 1170 | MITA T | 3 | 0.199 |
| 1171 | MITHAL A | 3 | 0.199 |
| 1172 | MIURA T | 3 | 0.199 |
| 1173 | MIYOSHI H | 3 | 0.199 |
| 1174 | MOHAN M | 3 | 0.199 |
| 1175 | MOON MK | 3 | 0.199 |
| 1176 | MULLENS W | 3 | 0.199 |
| 1177 | MUNTEANU M | 3 | 0.199 |
| 1178 | MUROHARA T | 3 | 0.199 |
| 1179 | MUSKIET MHA | 3 | 0.199 |
| 1180 | MUSTROPH J | 3 | 0.199 |
| 1181 | NAGAI Y | 3 | 0.199 |
| 1182 | NAKAMURA A | 3 | 0.199 |
| 1183 | NAKAMURA I | 3 | 0.199 |
| 1184 | NAKANO D | 3 | 0.199 |
| 1185 | NARUKAWA M | 3 | 0.199 |
| 1186 | NASSIF ME | 3 | 0.199 |
| 1187 | NAVARRO-GONZALEZ JF | 3 | 0.199 |
| 1188 | NEWMAN JD | 3 | 0.199 |
| 1189 | NICOLUCCI A | 3 | 0.199 |
| 1190 | NIEUWDORP M | 3 | 0.199 |
| 1191 | NISHIMURA R | 3 | 0.199 |
| 1192 | NISHIO S | 3 | 0.199 |
| 1193 | NORTON L | 3 | 0.199 |
| 1194 | OFORI-ASENSO R | 3 | 0.199 |
| 1195 | OGAWA Y | 3 | 0.199 |
| 1196 | OH R | 3 | 0.199 |
| 1197 | OKADA Y | 3 | 0.199 |
| 1198 | OKAMURA T | 3 | 0.199 |
| 1199 | ORSI E | 3 | 0.199 |
| 1200 | OSCARSSON J | 3 | 0.199 |
| 1201 | PANCHAPAKESAN U | 3 | 0.199 |
| 1202 | PAPADEMETRIOU V | 3 | 0.199 |
| 1203 | PAPADOPOULOS C | 3 | 0.199 |
| 1204 | PARAGH G | 3 | 0.199 |
| 1205 | PARIKH SJ | 3 | 0.199 |
| 1206 | PARK SO | 3 | 0.199 |
| 1207 | PATORNO E | 3 | 0.199 |
| 1208 | PATOULIAS D | 3 | 0.199 |
| 1209 | PEARSON ER | 3 | 0.199 |
| 1210 | PECOITS R | 3 | 0.199 |
| 1211 | PENLAND RC | 3 | 0.199 |
| 1212 | PERLMAN A | 3 | 0.199 |
| 1213 | PHAM SV | 3 | 0.199 |
| 1214 | PIEPOLI MF | 3 | 0.199 |
| 1215 | PINNETTI S | 3 | 0.199 |
| 1216 | PONG A | 3 | 0.199 |
| 1217 | PORTILLO CM | 3 | 0.199 |
| 1218 | PRAZNY M | 3 | 0.199 |
| 1219 | PRIEUR X | 3 | 0.199 |
| 1220 | QIU R | 3 | 0.199 |
| 1221 | QUAN A | 3 | 0.199 |
| 1222 | QUINAGLIA T | 3 | 0.199 |
| 1223 | REN D | 3 | 0.199 |
| 1224 | REN J | 3 | 0.199 |
| 1225 | RHEE SY | 3 | 0.199 |
| 1226 | RIZZO M | 3 | 0.199 |
| 1227 | ROSANO GMC | 3 | 0.199 |
| 1228 | ROUDAUT M | 3 | 0.199 |
| 1229 | ROUSSEL R | 3 | 0.199 |
| 1230 | RUFF CT | 3 | 0.199 |
| 1231 | SAKAI S | 3 | 0.199 |
| 1232 | SANO M | 3 | 0.199 |
| 1233 | SASAKI T | 3 | 0.199 |
| 1234 | SASSO FC | 3 | 0.199 |
| 1235 | SCHLAICH MP | 3 | 0.199 |
| 1236 | SCHOLTES RA | 3 | 0.199 |
| 1237 | SCHUMACHER CA | 3 | 0.199 |
| 1238 | SEINO Y | 3 | 0.199 |
| 1239 | SEMAN L | 3 | 0.199 |
| 1240 | SEUFERT J | 3 | 0.199 |
| 1241 | SHAO SC | 3 | 0.199 |
| 1242 | SHI LZ | 3 | 0.199 |
| 1243 | SHIMABUKURO M | 3 | 0.199 |
| 1244 | SHIMOMURA I | 3 | 0.199 |
| 1245 | SINGH AK | 3 | 0.199 |
| 1246 | SMEETH L | 3 | 0.199 |
| 1247 | SORICE GP | 3 | 0.199 |
| 1248 | SOURIJ H | 3 | 0.199 |
| 1249 | STAELS B | 3 | 0.199 |
| 1250 | STORGAARD H | 3 | 0.199 |
| 1251 | STRUTHERS AD | 3 | 0.199 |
| 1252 | SUGG J | 3 | 0.199 |
| 1253 | SUMIDA Y | 3 | 0.199 |
| 1254 | SUN B | 3 | 0.199 |
| 1255 | SUN J | 3 | 0.199 |
| 1256 | SUN T | 3 | 0.199 |
| 1257 | SUN XD | 3 | 0.199 |
| 1258 | SURMONT F | 3 | 0.199 |
| 1259 | SUZUKI D | 3 | 0.199 |
| 1260 | SVENSSON AM | 3 | 0.199 |
| 1261 | TAHARA A | 3 | 0.199 |
| 1262 | TAKAKURA S | 3 | 0.199 |
| 1263 | TAKIHATA M | 3 | 0.199 |
| 1264 | TANAKA Y | 3 | 0.199 |
| 1265 | TANG HL | 3 | 0.199 |
| 1266 | TANGRI N | 3 | 0.199 |
| 1267 | TANNO M | 3 | 0.199 |
| 1268 | TAYLOR SI | 3 | 0.199 |
| 1269 | TECSON KM | 3 | 0.199 |
| 1270 | TEOH H | 3 | 0.199 |
| 1271 | TERESHCHENKO S | 3 | 0.199 |
| 1272 | TOMLINSON B | 3 | 0.199 |
| 1273 | TONG NW | 3 | 0.199 |
| 1274 | TOUSOULIS D | 3 | 0.199 |
| 1275 | TOWNSEND R | 3 | 0.199 |
| 1276 | TOYAMA T | 3 | 0.199 |
| 1277 | TOYODA M | 3 | 0.199 |
| 1278 | TSUTSUI H | 3 | 0.199 |
| 1279 | TUTTLE KR | 3 | 0.199 |
| 1280 | USISKIN K | 3 | 0.199 |
| 1281 | UTHMAN L | 3 | 0.199 |
| 1282 | VERGARA A | 3 | 0.199 |
| 1283 | VILSBOLL T | 3 | 0.199 |
| 1284 | VINH PN | 3 | 0.199 |
| 1285 | VON LEWINSKI D | 3 | 0.199 |
| 1286 | VOORS AA | 3 | 0.199 |
| 1287 | VORA J | 3 | 0.199 |
| 1288 | WADA T | 3 | 0.199 |
| 1289 | WAGNER S | 3 | 0.199 |
| 1290 | WATANABE T | 3 | 0.199 |
| 1291 | WEBB DR | 3 | 0.199 |
| 1292 | WEBER NC | 3 | 0.199 |
| 1293 | WEIR MR | 3 | 0.199 |
| 1294 | WHITE WB | 3 | 0.199 |
| 1295 | WILDING JP | 3 | 0.199 |
| 1296 | WINOCOUR P | 3 | 0.199 |
| 1297 | XU LX | 3 | 0.199 |
| 1298 | XUE M | 3 | 0.199 |
| 1299 | YAMADA T | 3 | 0.199 |
| 1300 | YAMAMOTO T | 3 | 0.199 |
| 1301 | YANASE T | 3 | 0.199 |
| 1302 | YANDRAPALLI S | 3 | 0.199 |
| 1303 | YANG YHK | 3 | 0.199 |
| 1304 | YANO T | 3 | 0.199 |
| 1305 | YASUI A | 3 | 0.199 |
| 1306 | YOKOTA T | 3 | 0.199 |
| 1307 | YONEDA M | 3 | 0.199 |
| 1308 | YU XC | 3 | 0.199 |
| 1309 | ZHAI SD | 3 | 0.199 |
| 1310 | ZOUNGAS S | 3 | 0.199 |
| 1311 | ZWIENER I | 3 | 0.199 |
| 1312 | ABASSI Z | 2 | 0.133 |
| 1313 | ABDALLAH J | 2 | 0.133 |
| 1314 | ABDULLAH R | 2 | 0.133 |
| 1315 | ABE K | 2 | 0.133 |
| 1316 | ABEJUELA Z | 2 | 0.133 |
| 1317 | ABOUGLILA K | 2 | 0.133 |
| 1318 | ABRAHAM O | 2 | 0.133 |
| 1319 | ABRAMOWITZ M | 2 | 0.133 |
| 1320 | ABUSNANA S | 2 | 0.133 |
| 1321 | ACHARYA T | 2 | 0.133 |
| 1322 | ACOSTA I | 2 | 0.133 |
| 1323 | AGGARWAL N | 2 | 0.133 |
| 1324 | AGRA J | 2 | 0.133 |
| 1325 | AGROGIANNIS G | 2 | 0.133 |
| 1326 | AHMAD NNFN | 2 | 0.133 |
| 1327 | AHMADIEH H | 2 | 0.133 |
| 1328 | AHMED A | 2 | 0.133 |
| 1329 | AIELLO J | 2 | 0.133 |
| 1330 | AINSWORTH P | 2 | 0.133 |
| 1331 | AKHTAR M | 2 | 0.133 |
| 1332 | AKIYAMA H | 2 | 0.133 |
| 1333 | AKRIGHT L | 2 | 0.133 |
| 1334 | AKYEA-DJAMSON A | 2 | 0.133 |
| 1335 | AL DHAYBI O | 2 | 0.133 |
| 1336 | AL-KARADSHEH A | 2 | 0.133 |
| 1337 | AL-SHAREA A | 2 | 0.133 |
| 1338 | ALAMARTINE E | 2 | 0.133 |
| 1339 | ALAPPAN R | 2 | 0.133 |
| 1340 | ALBA M | 2 | 0.133 |
| 1341 | ALBISU JP | 2 | 0.133 |
| 1342 | ALI A | 2 | 0.133 |
| 1343 | ALI N | 2 | 0.133 |
| 1344 | ALI Z | 2 | 0.133 |
| 1345 | ALICIC R | 2 | 0.133 |
| 1346 | ALKAABI JM | 2 | 0.133 |
| 1347 | ALLISON DC | 2 | 0.133 |
| 1348 | ALONSO RDV | 2 | 0.133 |
| 1349 | ALVARISQUETA A | 2 | 0.133 |
| 1350 | AMAR A | 2 | 0.133 |
| 1351 | AMBROSIO G | 2 | 0.133 |
| 1352 | AMERENA J | 2 | 0.133 |
| 1353 | AMODEO C | 2 | 0.133 |
| 1354 | AMPUDIA-BLASCO FJ | 2 | 0.133 |
| 1355 | ANAND I | 2 | 0.133 |
| 1356 | ANAND IS | 2 | 0.133 |
| 1357 | ANDREEVA V | 2 | 0.133 |
| 1358 | ANEJA A | 2 | 0.133 |
| 1359 | ANGELESCU LM | 2 | 0.133 |
| 1360 | ANGELOVA A | 2 | 0.133 |
| 1361 | ANGHEL V | 2 | 0.133 |
| 1362 | ANKER S | 2 | 0.133 |
| 1363 | ANSARY TM | 2 | 0.133 |
| 1364 | ANTIC S | 2 | 0.133 |
| 1365 | ANZAI T | 2 | 0.133 |
| 1366 | AOKI H | 2 | 0.133 |
| 1367 | AOKI S | 2 | 0.133 |
| 1368 | AOYAMA K | 2 | 0.133 |
| 1369 | AOYAMA T | 2 | 0.133 |
| 1370 | ARAUZ-PACHECO C | 2 | 0.133 |
| 1371 | ARBEL R | 2 | 0.133 |
| 1372 | ARCOS E | 2 | 0.133 |
| 1373 | ARENA R | 2 | 0.133 |
| 1374 | ARENALES JAS | 2 | 0.133 |
| 1375 | ARFEEN S | 2 | 0.133 |
| 1376 | ARNOTT C | 2 | 0.133 |
| 1377 | ARUTCHELVAM V | 2 | 0.133 |
| 1378 | ARVIND M | 2 | 0.133 |
| 1379 | ASAKAWA M | 2 | 0.133 |
| 1380 | ASAKURA M | 2 | 0.133 |
| 1381 | ASANO A | 2 | 0.133 |
| 1382 | ASANO T | 2 | 0.133 |
| 1383 | ATAR D | 2 | 0.133 |
| 1384 | ATHANASIADOU E | 2 | 0.133 |
| 1385 | ATHYROS V | 2 | 0.133 |
| 1386 | ATRAY N | 2 | 0.133 |
| 1387 | AUGER C | 2 | 0.133 |
| 1388 | AVENDANO J | 2 | 0.133 |
| 1389 | AVGERINOS I | 2 | 0.133 |
| 1390 | AVRAM RI | 2 | 0.133 |
| 1391 | AZAR S | 2 | 0.133 |
| 1392 | AZAR ST | 2 | 0.133 |
| 1393 | AZURI J | 2 | 0.133 |
| 1394 | BABIKOVA J | 2 | 0.133 |
| 1395 | BACHINA S | 2 | 0.133 |
| 1396 | BADAT A | 2 | 0.133 |
| 1397 | BAIGENT C | 2 | 0.133 |
| 1398 | BAJAJ H | 2 | 0.133 |
| 1399 | BALIJEPALLI C | 2 | 0.133 |
| 1400 | BALLESTEROS CG | 2 | 0.133 |
| 1401 | BANACH M | 2 | 0.133 |
| 1402 | BANGERT A | 2 | 0.133 |
| 1403 | BARBIN CM | 2 | 0.133 |
| 1404 | BARGIOTA A | 2 | 0.133 |
| 1405 | BARNA O | 2 | 0.133 |
| 1406 | BARNARD M | 2 | 0.133 |
| 1407 | BARNHILL P | 2 | 0.133 |
| 1408 | BARON PF | 2 | 0.133 |
| 1409 | BARRANCO E | 2 | 0.133 |
| 1410 | BARRERA C | 2 | 0.133 |
| 1411 | BARRERA-CHIMAL J | 2 | 0.133 |
| 1412 | BARRETO J | 2 | 0.133 |
| 1413 | BARRETT TD | 2 | 0.133 |
| 1414 | BARRIOS C | 2 | 0.133 |
| 1415 | BARTASKOVA D | 2 | 0.133 |
| 1416 | BARTOLACCI I | 2 | 0.133 |
| 1417 | BARTON P | 2 | 0.133 |
| 1418 | BARYSHEVA O | 2 | 0.133 |
| 1419 | BASU D | 2 | 0.133 |
| 1420 | BATTSON ML | 2 | 0.133 |
| 1421 | BEACOM M | 2 | 0.133 |
| 1422 | BEHARA V | 2 | 0.133 |
| 1423 | BEHNAMMANESH G | 2 | 0.133 |
| 1424 | BEITELSHEES AL | 2 | 0.133 |
| 1425 | BELL C | 2 | 0.133 |
| 1426 | BELLARY S | 2 | 0.133 |
| 1427 | BELLIDO D | 2 | 0.133 |
| 1428 | BELO D | 2 | 0.133 |
| 1429 | BELOBRADKOVA J | 2 | 0.133 |
| 1430 | BENTLEY-LEWIS R | 2 | 0.133 |
| 1431 | BENUSOVA O | 2 | 0.133 |
| 1432 | BERGER JS | 2 | 0.133 |
| 1433 | BERKOVIC MC | 2 | 0.133 |
| 1434 | BERLI MA | 2 | 0.133 |
| 1435 | BHATTACHARYYA A | 2 | 0.133 |
| 1436 | BIELA LM | 2 | 0.133 |
| 1437 | BIJATA-BRONISZ R | 2 | 0.133 |
| 1438 | BILIR SP | 2 | 0.133 |
| 1439 | BILLINGSLEY HE | 2 | 0.133 |
| 1440 | BILYK SD | 2 | 0.133 |
| 1441 | BIN SHUDIM SS | 2 | 0.133 |
| 1442 | BLASCHEK W | 2 | 0.133 |
| 1443 | BLAU JE | 2 | 0.133 |
| 1444 | BLONDE L | 2 | 0.133 |
| 1445 | BONADONNA RC | 2 | 0.133 |
| 1446 | BONINO B | 2 | 0.133 |
| 1447 | BONNET F | 2 | 0.133 |
| 1448 | BOOZ GW | 2 | 0.133 |
| 1449 | BORDONAVA A | 2 | 0.133 |
| 1450 | BOROT S | 2 | 0.133 |
| 1451 | BOSCH A | 2 | 0.133 |
| 1452 | BOSE M | 2 | 0.133 |
| 1453 | BOTKER HE | 2 | 0.133 |
| 1454 | BOTSYURKO V | 2 | 0.133 |
| 1455 | BOUCHI R | 2 | 0.133 |
| 1456 | BOURA P | 2 | 0.133 |
| 1457 | BOWMAN-STROUD C | 2 | 0.133 |
| 1458 | BOYLE LD | 2 | 0.133 |
| 1459 | BREEDT J | 2 | 0.133 |
| 1460 | BREGMAN R | 2 | 0.133 |
| 1461 | BRIASOULIS A | 2 | 0.133 |
| 1462 | BRUNO RM | 2 | 0.133 |
| 1463 | BUCKLEY LF | 2 | 0.133 |
| 1464 | BUGANOVA I | 2 | 0.133 |
| 1465 | BURNIER M | 2 | 0.133 |
| 1466 | BURST V | 2 | 0.133 |
| 1467 | BUSEGEANU MM | 2 | 0.133 |
| 1468 | BUTLER AE | 2 | 0.133 |
| 1469 | BUZZETTI R | 2 | 0.133 |
| 1470 | CAIN VA | 2 | 0.133 |
| 1471 | CALCANEO JP | 2 | 0.133 |
| 1472 | CALELLA P | 2 | 0.133 |
| 1473 | CALLAN L | 2 | 0.133 |
| 1474 | CANADA JM | 2 | 0.133 |
| 1475 | CANONGE RS | 2 | 0.133 |
| 1476 | CANTERO MC | 2 | 0.133 |
| 1477 | CAPPELLARI R | 2 | 0.133 |
| 1478 | CARINGAL C | 2 | 0.133 |
| 1479 | CARLSON CJ | 2 | 0.133 |
| 1480 | CARNAGARIN R | 2 | 0.133 |
| 1481 | CARPENTER AJ | 2 | 0.133 |
| 1482 | CARRILLO LV | 2 | 0.133 |
| 1483 | CARSON P | 2 | 0.133 |
| 1484 | CARSTENSEN B | 2 | 0.133 |
| 1485 | CARTASEGNA LR | 2 | 0.133 |
| 1486 | CASABELLA TS | 2 | 0.133 |
| 1487 | CASCIARO F | 2 | 0.133 |
| 1488 | CASSADER M | 2 | 0.133 |
| 1489 | CASTELLANOS RO | 2 | 0.133 |
| 1490 | CASTELLINO P | 2 | 0.133 |
| 1491 | CASTRO FC | 2 | 0.133 |
| 1492 | CEBALLOS RIL | 2 | 0.133 |
| 1493 | CEFALO CMA | 2 | 0.133 |
| 1494 | CEPEDA AV | 2 | 0.133 |
| 1495 | CERCOS E | 2 | 0.133 |
| 1496 | CERVERA LFF | 2 | 0.133 |
| 1497 | CHACRA AR | 2 | 0.133 |
| 1498 | CHAE DW | 2 | 0.133 |
| 1499 | CHAN J | 2 | 0.133 |
| 1500 | CHAN M | 2 | 0.133 |
| 1501 | CHAN P | 2 | 0.133 |
| 1502 | CHANDRASEKAR B | 2 | 0.133 |
| 1503 | CHANG CT | 2 | 0.133 |
| 1504 | CHARBONNEL B | 2 | 0.133 |
| 1505 | CHAROKOPOU M | 2 | 0.133 |
| 1506 | CHATTIPAKORN SC | 2 | 0.133 |
| 1507 | CHAVEZ ET | 2 | 0.133 |
| 1508 | CHEN CH | 2 | 0.133 |
| 1509 | CHEN F | 2 | 0.133 |
| 1510 | CHEN HY | 2 | 0.133 |
| 1511 | CHEN N | 2 | 0.133 |
| 1512 | CHEN QK | 2 | 0.133 |
| 1513 | CHEN XZ | 2 | 0.133 |
| 1514 | CHENG HM | 2 | 0.133 |
| 1515 | CHENG JWM | 2 | 0.133 |
| 1516 | CHEUNG AK | 2 | 0.133 |
| 1517 | CHEUNG NW | 2 | 0.133 |
| 1518 | CHIN K | 2 | 0.133 |
| 1519 | CHIN KL | 2 | 0.133 |
| 1520 | CHIONCEL O | 2 | 0.133 |
| 1521 | CHIOVATO L | 2 | 0.133 |
| 1522 | CHIZHOV D | 2 | 0.133 |
| 1523 | CHO KY | 2 | 0.133 |
| 1524 | CHO YM | 2 | 0.133 |
| 1525 | CHOI KM | 2 | 0.133 |
| 1526 | CHOUINARD G | 2 | 0.133 |
| 1527 | CHRISTENSEN MB | 2 | 0.133 |
| 1528 | CHUANG LM | 2 | 0.133 |
| 1529 | CHUN S | 2 | 0.133 |
| 1530 | CIF A | 2 | 0.133 |
| 1531 | CINTI F | 2 | 0.133 |
| 1532 | CLAYTON T | 2 | 0.133 |
| 1533 | CLEGG LE | 2 | 0.133 |
| 1534 | CLELAND JGF | 2 | 0.133 |
| 1535 | CLEMENT M | 2 | 0.133 |
| 1536 | COATS AJS | 2 | 0.133 |
| 1537 | COBBLE ME | 2 | 0.133 |
| 1538 | COLASO PDG | 2 | 0.133 |
| 1539 | COLEMAN CI | 2 | 0.133 |
| 1540 | COLHOUN HM | 2 | 0.133 |
| 1541 | COLOMA GC | 2 | 0.133 |
| 1542 | COMETA LV | 2 | 0.133 |
| 1543 | COMIA RS | 2 | 0.133 |
| 1544 | COMMENDATORE V | 2 | 0.133 |
| 1545 | CONNELLY K | 2 | 0.133 |
| 1546 | CONTIERI FLD | 2 | 0.133 |
| 1547 | CONWAY J | 2 | 0.133 |
| 1548 | COOPER LB | 2 | 0.133 |
| 1549 | COPPINI R | 2 | 0.133 |
| 1550 | CORREA-ROTTER R | 2 | 0.133 |
| 1551 | COURNOYER S | 2 | 0.133 |
| 1552 | CREA F | 2 | 0.133 |
| 1553 | CROWE S | 2 | 0.133 |
| 1554 | CRUZ JB | 2 | 0.133 |
| 1555 | CSECSEI G | 2 | 0.133 |
| 1556 | CSIKY B | 2 | 0.133 |
| 1557 | CUADRADO J | 2 | 0.133 |
| 1558 | CULAK J | 2 | 0.133 |
| 1559 | CUNEO CA | 2 | 0.133 |
| 1560 | CUSI K | 2 | 0.133 |
| 1561 | CUSTODIO JS | 2 | 0.133 |
| 1562 | CUSUMANO AM | 2 | 0.133 |
| 1563 | CUTHBERTSON DJ | 2 | 0.133 |
| 1564 | CUTRUZZOLA A | 2 | 0.133 |
| 1565 | CYPRYK K | 2 | 0.133 |
| 1566 | D'AMARIO D | 2 | 0.133 |
| 1567 | D'AVILA D | 2 | 0.133 |
| 1568 | DA COSTA FAA | 2 | 0.133 |
| 1569 | DAIBER A | 2 | 0.133 |
| 1570 | DANDONA P | 2 | 0.133 |
| 1571 | DANIEL H | 2 | 0.133 |
| 1572 | DANNE T | 2 | 0.133 |
| 1573 | DANOS P | 2 | 0.133 |
| 1574 | DAREKAR A | 2 | 0.133 |
| 1575 | DAROZA G | 2 | 0.133 |
| 1576 | DAS NA | 2 | 0.133 |
| 1577 | DAVE N | 2 | 0.133 |
| 1578 | DAVIES E | 2 | 0.133 |
| 1579 | DAVIES G | 2 | 0.133 |
| 1580 | DAVIES S | 2 | 0.133 |
| 1581 | DAVIS TME | 2 | 0.133 |
| 1582 | DAWSON A | 2 | 0.133 |
| 1583 | DAWSON J | 2 | 0.133 |
| 1584 | DAWWAS GK | 2 | 0.133 |
| 1585 | DE ALMEIDA RS | 2 | 0.133 |
| 1586 | DE BRITO CM | 2 | 0.133 |
| 1587 | DE CHAZAL HM | 2 | 0.133 |
| 1588 | DE LA FUENTE RAL | 2 | 0.133 |
| 1589 | DE LOS RIOS JE | 2 | 0.133 |
| 1590 | DE MARTINO F | 2 | 0.133 |
| 1591 | DE SALAZAR DIM | 2 | 0.133 |
| 1592 | DE SERRES S | 2 | 0.133 |
| 1593 | DE SOUZA P | 2 | 0.133 |
| 1594 | DEAK L | 2 | 0.133 |
| 1595 | DEBONI LM | 2 | 0.133 |
| 1596 | DEEKS ED | 2 | 0.133 |
| 1597 | DEEPAK D | 2 | 0.133 |
| 1598 | DEGAWA H | 2 | 0.133 |
| 1599 | DELLEGROTTAGLIE S | 2 | 0.133 |
| 1600 | DELOS SANTOS L | 2 | 0.133 |
| 1601 | DEMIAN LD | 2 | 0.133 |
| 1602 | DESJARDINS JF | 2 | 0.133 |
| 1603 | DESOUZA C | 2 | 0.133 |
| 1604 | DI CARLO A | 2 | 0.133 |
| 1605 | DI CIANNI G | 2 | 0.133 |
| 1606 | DI FRANCO A | 2 | 0.133 |
| 1607 | DIAZ JPM | 2 | 0.133 |
| 1608 | DIMITRIADIS GK | 2 | 0.133 |
| 1609 | DIMITROV S | 2 | 0.133 |
| 1610 | DING H | 2 | 0.133 |
| 1611 | DJORDJEVIC M | 2 | 0.133 |
| 1612 | DOBRONRAVOV V | 2 | 0.133 |
| 1613 | DOEHNER W | 2 | 0.133 |
| 1614 | DOHNALOVA L | 2 | 0.133 |
| 1615 | DOI T | 2 | 0.133 |
| 1616 | DOUGLAS IJ | 2 | 0.133 |
| 1617 | DOUTHAT WG | 2 | 0.133 |
| 1618 | DRAN RD | 2 | 0.133 |
| 1619 | DRASNAR T | 2 | 0.133 |
| 1620 | DREVAL A | 2 | 0.133 |
| 1621 | DROSTE C | 2 | 0.133 |
| 1622 | DROZDZ J | 2 | 0.133 |
| 1623 | DRUYTS E | 2 | 0.133 |
| 1624 | DUBE F | 2 | 0.133 |
| 1625 | DUDAR I | 2 | 0.133 |
| 1626 | DURANTE W | 2 | 0.133 |
| 1627 | DUSSOL B | 2 | 0.133 |
| 1628 | DYCK JRB | 2 | 0.133 |
| 1629 | DZUPINA A | 2 | 0.133 |
| 1630 | DZUPONOVA J | 2 | 0.133 |
| 1631 | EDWIN FM | 2 | 0.133 |
| 1632 | EGUCHI K | 2 | 0.133 |
| 1633 | EICKHOFF MK | 2 | 0.133 |
| 1634 | EID A | 2 | 0.133 |
| 1635 | EIRAS S | 2 | 0.133 |
| 1636 | EL KOSSI M | 2 | 0.133 |
| 1637 | ELDOR R | 2 | 0.133 |
| 1638 | ELGENDY IY | 2 | 0.133 |
| 1639 | ELLIS SL | 2 | 0.133 |
| 1640 | ENGELI S | 2 | 0.133 |
| 1641 | ENGLISH P | 2 | 0.133 |
| 1642 | ESCALANTE TD | 2 | 0.133 |
| 1643 | ESCOBEDO RZ | 2 | 0.133 |
| 1644 | ESPOSITO I | 2 | 0.133 |
| 1645 | EVERETT BM | 2 | 0.133 |
| 1646 | FABBRINI E | 2 | 0.133 |
| 1647 | FACILA L | 2 | 0.133 |
| 1648 | FAGHIH M | 2 | 0.133 |
| 1649 | FARIAS E | 2 | 0.133 |
| 1650 | FARINA MM | 2 | 0.133 |
| 1651 | FARMAKIS D | 2 | 0.133 |
| 1652 | FATHI A | 2 | 0.133 |
| 1653 | FAULMANN G | 2 | 0.133 |
| 1654 | FAUVEL JP | 2 | 0.133 |
| 1655 | FEDAK PWM | 2 | 0.133 |
| 1656 | FEGAN PG | 2 | 0.133 |
| 1657 | FELICIO JS | 2 | 0.133 |
| 1658 | FERARIU IE | 2 | 0.133 |
| 1659 | FERKL R | 2 | 0.133 |
| 1660 | FERNANDEZ AL | 2 | 0.133 |
| 1661 | FERNANDEZ MF | 2 | 0.133 |
| 1662 | FERNANDEZ-FERNANDEZ B | 2 | 0.133 |
| 1663 | FERNANDO K | 2 | 0.133 |
| 1664 | FERRARI R | 2 | 0.133 |
| 1665 | FERRINI M | 2 | 0.133 |
| 1666 | FILION KB | 2 | 0.133 |
| 1667 | FILIPPATOS T | 2 | 0.133 |
| 1668 | FINKELSTEIN H | 2 | 0.133 |
| 1669 | FIUZAT M | 2 | 0.133 |
| 1670 | FLORES E | 2 | 0.133 |
| 1671 | FLORES FJ | 2 | 0.133 |
| 1672 | FLORY JH | 2 | 0.133 |
| 1673 | FOGARTY D | 2 | 0.133 |
| 1674 | FONSECA V | 2 | 0.133 |
| 1675 | FONTES-CARVALHO R | 2 | 0.133 |
| 1676 | FOOTE C | 2 | 0.133 |
| 1677 | FORBES JM | 2 | 0.133 |
| 1678 | FORMOSO G | 2 | 0.133 |
| 1679 | FRADKIN J | 2 | 0.133 |
| 1680 | FRAGALE G | 2 | 0.133 |
| 1681 | FRAIGE F | 2 | 0.133 |
| 1682 | FRANCO GM | 2 | 0.133 |
| 1683 | FRANCO RJD | 2 | 0.133 |
| 1684 | FRANZEN S | 2 | 0.133 |
| 1685 | FRASCA G | 2 | 0.133 |
| 1686 | FRASER D | 2 | 0.133 |
| 1687 | FRASER I | 2 | 0.133 |
| 1688 | FRESNEDO GF | 2 | 0.133 |
| 1689 | FRESTON J | 2 | 0.133 |
| 1690 | FRETES JO | 2 | 0.133 |
| 1691 | FRIMODT-MOLLER M | 2 | 0.133 |
| 1692 | FRITSCHE A | 2 | 0.133 |
| 1693 | FUCHIGAMI A | 2 | 0.133 |
| 1694 | FUIANO G | 2 | 0.133 |
| 1695 | FUJISAWA Y | 2 | 0.133 |
| 1696 | FUJISHIRO M | 2 | 0.133 |
| 1697 | FUKATSU A | 2 | 0.133 |
| 1698 | FUKUDA D | 2 | 0.133 |
| 1699 | FULOP P | 2 | 0.133 |
| 1700 | FURUKI T | 2 | 0.133 |
| 1701 | FUSHTEY I | 2 | 0.133 |
| 1702 | FUSTER V | 2 | 0.133 |
| 1703 | GAEDE P | 2 | 0.133 |
| 1704 | GAJARDO VS | 2 | 0.133 |
| 1705 | GALLEGOS KM | 2 | 0.133 |
| 1706 | GALLO LA | 2 | 0.133 |
| 1707 | GAMBARO G | 2 | 0.133 |
| 1708 | GAMBINO R | 2 | 0.133 |
| 1709 | GAN CL | 2 | 0.133 |
| 1710 | GAN SL | 2 | 0.133 |
| 1711 | GAO YM | 2 | 0.133 |
| 1712 | GARCIA I | 2 | 0.133 |
| 1713 | GARCIA NH | 2 | 0.133 |
| 1714 | GARCIA PA | 2 | 0.133 |
| 1715 | GARCIA-CABALLERO T | 2 | 0.133 |
| 1716 | GARG A | 2 | 0.133 |
| 1717 | GARG V | 2 | 0.133 |
| 1718 | GARGIULO P | 2 | 0.133 |
| 1719 | GARIBOTTO G | 2 | 0.133 |
| 1720 | GAROFALO C | 2 | 0.133 |
| 1721 | GASTALDI A | 2 | 0.133 |
| 1722 | GAUPSIENE E | 2 | 0.133 |
| 1723 | GELERSZTEIN E | 2 | 0.133 |
| 1724 | GENADIEVA V | 2 | 0.133 |
| 1725 | GENOVA-HRISTOVA G | 2 | 0.133 |
| 1726 | GENOVESE S | 2 | 0.133 |
| 1727 | GEORGIANOS PI | 2 | 0.133 |
| 1728 | GHAZI A | 2 | 0.133 |
| 1729 | GHIADONI L | 2 | 0.133 |
| 1730 | GIANNINI L | 2 | 0.133 |
| 1731 | GIGLIO RV | 2 | 0.133 |
| 1732 | GIMENO JEJ | 2 | 0.133 |
| 1733 | GIORDA C | 2 | 0.133 |
| 1734 | GLENNY JA | 2 | 0.133 |
| 1735 | GLINKINA I | 2 | 0.133 |
| 1736 | GLUUD LL | 2 | 0.133 |
| 1737 | GODOY LC | 2 | 0.133 |
| 1738 | GOLOVCHENKO O | 2 | 0.133 |
| 1739 | GOMES M | 2 | 0.133 |
| 1740 | GOMEZ O | 2 | 0.133 |
| 1741 | GOMEZ-PERALTA F | 2 | 0.133 |
| 1742 | GONZALEZ A | 2 | 0.133 |
| 1743 | GONZALEZ AS | 2 | 0.133 |
| 1744 | GONZALEZ C | 2 | 0.133 |
| 1745 | GONZALEZ JP | 2 | 0.133 |
| 1746 | GOODMAN SG | 2 | 0.133 |
| 1747 | GOPALAKRISHNAN N | 2 | 0.133 |
| 1748 | GOURDY P | 2 | 0.133 |
| 1749 | GOVENDER R | 2 | 0.133 |
| 1750 | GOVENDER V | 2 | 0.133 |
| 1751 | GOYCOA C | 2 | 0.133 |
| 1752 | GRACIOUS N | 2 | 0.133 |
| 1753 | GRAY A | 2 | 0.133 |
| 1754 | GREEN A | 2 | 0.133 |
| 1755 | GREENBERG B | 2 | 0.133 |
| 1756 | GREENE SJ | 2 | 0.133 |
| 1757 | GREENFIELD JR | 2 | 0.133 |
| 1758 | GRELONI GC | 2 | 0.133 |
| 1759 | GRIFFEN SC | 2 | 0.133 |
| 1760 | GRINEVA E | 2 | 0.133 |
| 1761 | GRONDA E | 2 | 0.133 |
| 1762 | GU SY | 2 | 0.133 |
| 1763 | GUDBJORNSDOTTIR S | 2 | 0.133 |
| 1764 | GUERRERO RAA | 2 | 0.133 |
| 1765 | GUINSBURG A | 2 | 0.133 |
| 1766 | GUJA C | 2 | 0.133 |
| 1767 | GULDRIS SC | 2 | 0.133 |
| 1768 | GULSETH HL | 2 | 0.133 |
| 1769 | GUNSTONE A | 2 | 0.133 |
| 1770 | GUNTON JE | 2 | 0.133 |
| 1771 | GUO Y | 2 | 0.133 |
| 1772 | GUPTA N | 2 | 0.133 |
| 1773 | GUSTAFSSON F | 2 | 0.133 |
| 1774 | GUTHRIE R | 2 | 0.133 |
| 1775 | GUZMAN JR | 2 | 0.133 |
| 1776 | GYRINA O | 2 | 0.133 |
| 1777 | HAASE FP | 2 | 0.133 |
| 1778 | HABIBI J | 2 | 0.133 |
| 1779 | HACHMANN JCT | 2 | 0.133 |
| 1780 | HALCIAKOVA K | 2 | 0.133 |
| 1781 | HALL T | 2 | 0.133 |
| 1782 | HALMAGYI I | 2 | 0.133 |
| 1783 | HAMMERMAN A | 2 | 0.133 |
| 1784 | HAMOUDA NN | 2 | 0.133 |
| 1785 | HAN F | 2 | 0.133 |
| 1786 | HAN Y | 2 | 0.133 |
| 1787 | HANSRAJ A | 2 | 0.133 |
| 1788 | HARASHIMA S | 2 | 0.133 |
| 1789 | HARCSA E | 2 | 0.133 |
| 1790 | HASENFUSS G | 2 | 0.133 |
| 1791 | HASHIMOTO K | 2 | 0.133 |
| 1792 | HATORI N | 2 | 0.133 |
| 1793 | HAYASHIDA CY | 2 | 0.133 |
| 1794 | HAYDEN MR | 2 | 0.133 |
| 1795 | HAYNES R | 2 | 0.133 |
| 1796 | HE HB | 2 | 0.133 |
| 1797 | HE JG | 2 | 0.133 |
| 1798 | HE X | 2 | 0.133 |
| 1799 | HEERSPINK HL | 2 | 0.133 |
| 1800 | HEHNKE U | 2 | 0.133 |
| 1801 | HELMLINGER G | 2 | 0.133 |
| 1802 | HERAT LY | 2 | 0.133 |
| 1803 | HERMAN WH | 2 | 0.133 |
| 1804 | HERMIDA S | 2 | 0.133 |
| 1805 | HERRINGTON WG | 2 | 0.133 |
| 1806 | HEYMAN SN | 2 | 0.133 |
| 1807 | HEYNE N | 2 | 0.133 |
| 1808 | HICKMAN A | 2 | 0.133 |
| 1809 | HICKS D | 2 | 0.133 |
| 1810 | HIGASHI Y | 2 | 0.133 |
| 1811 | HIGUERA JD | 2 | 0.133 |
| 1812 | HILL L | 2 | 0.133 |
| 1813 | HILLEBRANDS JL | 2 | 0.133 |
| 1814 | HINNEN D | 2 | 0.133 |
| 1815 | HINTON W | 2 | 0.133 |
| 1816 | HIRANO T | 2 | 0.133 |
| 1817 | HISHIKI T | 2 | 0.133 |
| 1818 | HISSA MN | 2 | 0.133 |
| 1819 | HOME P | 2 | 0.133 |
| 1820 | HOPPER I | 2 | 0.133 |
| 1821 | HOSODA K | 2 | 0.133 |
| 1822 | HOTLOS L | 2 | 0.133 |
| 1823 | HOUSTON JG | 2 | 0.133 |
| 1824 | HOWARTH FC | 2 | 0.133 |
| 1825 | HOWLETT J | 2 | 0.133 |
| 1826 | HOWLETT JG | 2 | 0.133 |
| 1827 | HRISTOZOV K | 2 | 0.133 |
| 1828 | HSIA TL | 2 | 0.133 |
| 1829 | HSIEH CH | 2 | 0.133 |
| 1830 | HU JB | 2 | 0.133 |
| 1831 | HU M | 2 | 0.133 |
| 1832 | HUGGINS LA | 2 | 0.133 |
| 1833 | HUYCK S | 2 | 0.133 |
| 1834 | HVEEM K | 2 | 0.133 |
| 1835 | IACOBELLIS G | 2 | 0.133 |
| 1836 | IGLESIAS JN | 2 | 0.133 |
| 1837 | IITSUKA T | 2 | 0.133 |
| 1838 | IKONOMIDIS I | 2 | 0.133 |
| 1839 | ILAVSKA A | 2 | 0.133 |
| 1840 | IM K | 2 | 0.133 |
| 1841 | IMAMURA M | 2 | 0.133 |
| 1842 | IMPRONTA F | 2 | 0.133 |
| 1843 | INOUE K | 2 | 0.133 |
| 1844 | IRACE C | 2 | 0.133 |
| 1845 | ISHIKAWA K | 2 | 0.133 |
| 1846 | ISHIZU T | 2 | 0.133 |
| 1847 | ISMAIL M | 2 | 0.133 |
| 1848 | ISMAIL-BEIGI F | 2 | 0.133 |
| 1849 | ITO K | 2 | 0.133 |
| 1850 | IZUMIYAMA H | 2 | 0.133 |
| 1851 | JABBOUR S | 2 | 0.133 |
| 1852 | JABBOUR SA | 2 | 0.133 |
| 1853 | JACKSON A | 2 | 0.133 |
| 1854 | JAISSER F | 2 | 0.133 |
| 1855 | JAMES J | 2 | 0.133 |
| 1856 | JANEZ A | 2 | 0.133 |
| 1857 | JANG H | 2 | 0.133 |
| 1858 | JANIC M | 2 | 0.133 |
| 1859 | JANUSZEWICZ A | 2 | 0.133 |
| 1860 | JANUZZI J | 2 | 0.133 |
| 1861 | JAVOR E | 2 | 0.133 |
| 1862 | JEONG IK | 2 | 0.133 |
| 1863 | JERUMS G | 2 | 0.133 |
| 1864 | JERWAN-KEIM R | 2 | 0.133 |
| 1865 | JIA GH | 2 | 0.133 |
| 1866 | JIANG J | 2 | 0.133 |
| 1867 | JIMENEZ LEH | 2 | 0.133 |
| 1868 | JIMENO C | 2 | 0.133 |
| 1869 | JOERG IER | 2 | 0.133 |
| 1870 | JOHANSSON L | 2 | 0.133 |
| 1871 | JOHNSON D | 2 | 0.133 |
| 1872 | JOHNSON EJ | 2 | 0.133 |
| 1873 | JONASSON C | 2 | 0.133 |
| 1874 | JORQUERA JGG | 2 | 0.133 |
| 1875 | JOSEPH F | 2 | 0.133 |
| 1876 | JOSHI M | 2 | 0.133 |
| 1877 | JOSHI S | 2 | 0.133 |
| 1878 | JOUBERT M | 2 | 0.133 |
| 1879 | JUAREZ ET | 2 | 0.133 |
| 1880 | JUN M | 2 | 0.133 |
| 1881 | JUNCOS LI | 2 | 0.133 |
| 1882 | JUNI P | 2 | 0.133 |
| 1883 | KACZMAREK B | 2 | 0.133 |
| 1884 | KADARIYA D | 2 | 0.133 |
| 1885 | KAJIYAMA S | 2 | 0.133 |
| 1886 | KALRA PA | 2 | 0.133 |
| 1887 | KALTSAS G | 2 | 0.133 |
| 1888 | KALYANI RR | 2 | 0.133 |
| 1889 | KAMAL MA | 2 | 0.133 |
| 1890 | KAMENOV Z | 2 | 0.133 |
| 1891 | KAMINSKA A | 2 | 0.133 |
| 1892 | KANAMORI A | 2 | 0.133 |
| 1893 | KANBAY M | 2 | 0.133 |
| 1894 | KANDA E | 2 | 0.133 |
| 1895 | KANDASWAMY P | 2 | 0.133 |
| 1896 | KANEGAE H | 2 | 0.133 |
| 1897 | KANETO H | 2 | 0.133 |
| 1898 | KANG ES | 2 | 0.133 |
| 1899 | KANNENKERIL D | 2 | 0.133 |
| 1900 | KANSAL A | 2 | 0.133 |
| 1901 | KAPLOWITZ N | 2 | 0.133 |
| 1902 | KARAM SL | 2 | 0.133 |
| 1903 | KARG MV | 2 | 0.133 |
| 1904 | KASHINE S | 2 | 0.133 |
| 1905 | KASSI E | 2 | 0.133 |
| 1906 | KATAKAMI N | 2 | 0.133 |
| 1907 | KATOVA T | 2 | 0.133 |
| 1908 | KATSIMARDOU A | 2 | 0.133 |
| 1909 | KAWADA T | 2 | 0.133 |
| 1910 | KAWAGUCHI T | 2 | 0.133 |
| 1911 | KAWANAMI D | 2 | 0.133 |
| 1912 | KAZMIRCHUK A | 2 | 0.133 |
| 1913 | KEARNEY A | 2 | 0.133 |
| 1914 | KEITEL E | 2 | 0.133 |
| 1915 | KELLY MS | 2 | 0.133 |
| 1916 | KELTAI K | 2 | 0.133 |
| 1917 | KENDERESKI A | 2 | 0.133 |
| 1918 | KERESZTESI S | 2 | 0.133 |
| 1919 | KERNAN WN | 2 | 0.133 |
| 1920 | KHANDWALA H | 2 | 0.133 |
| 1921 | KHIRMANOV V | 2 | 0.133 |
| 1922 | KHOO CM | 2 | 0.133 |
| 1923 | KHULLAR D | 2 | 0.133 |
| 1924 | KIM BY | 2 | 0.133 |
| 1925 | KIM HS | 2 | 0.133 |
| 1926 | KIM J | 2 | 0.133 |
| 1927 | KIM SH | 2 | 0.133 |
| 1928 | KIM YH | 2 | 0.133 |
| 1929 | KIMURA G | 2 | 0.133 |
| 1930 | KIMURA M | 2 | 0.133 |
| 1931 | KIMURA Y | 2 | 0.133 |
| 1932 | KINGHORN AD | 2 | 0.133 |
| 1933 | KISS K | 2 | 0.133 |
| 1934 | KITAZAWA T | 2 | 0.133 |
| 1935 | KLEIN A | 2 | 0.133 |
| 1936 | KLOKOCNIKOVA V | 2 | 0.133 |
| 1937 | KLYVER MI | 2 | 0.133 |
| 1938 | KOBARA H | 2 | 0.133 |
| 1939 | KODERA T | 2 | 0.133 |
| 1940 | KOHARA SK | 2 | 0.133 |
| 1941 | KOHLER S | 2 | 0.133 |
| 1942 | KOLESNIK E | 2 | 0.133 |
| 1943 | KOLESNYK M | 2 | 0.133 |
| 1944 | KOLMAKOVA E | 2 | 0.133 |
| 1945 | KOMISARENKO I | 2 | 0.133 |
| 1946 | KONG WY | 2 | 0.133 |
| 1947 | KONG YZ | 2 | 0.133 |
| 1948 | KONO H | 2 | 0.133 |
| 1949 | KONYVES L | 2 | 0.133 |
| 1950 | KOROLEVA T | 2 | 0.133 |
| 1951 | KORZH O | 2 | 0.133 |
| 1952 | KOSHMAN SL | 2 | 0.133 |
| 1953 | KOSTAKIS ID | 2 | 0.133 |
| 1954 | KOTSA K | 2 | 0.133 |
| 1955 | KOUNDURDJIEV A | 2 | 0.133 |
| 1956 | KOURLABA G | 2 | 0.133 |
| 1957 | KOVAR R | 2 | 0.133 |
| 1958 | KOWAL S | 2 | 0.133 |
| 1959 | KOYAMA K | 2 | 0.133 |
| 1960 | KOZLOVIENE D | 2 | 0.133 |
| 1961 | KRAFT F | 2 | 0.133 |
| 1962 | KRAMER CK | 2 | 0.133 |
| 1963 | KRAVCHUN N | 2 | 0.133 |
| 1964 | KRISHNAN S | 2 | 0.133 |
| 1965 | KROLLER-SCHON S | 2 | 0.133 |
| 1966 | KRYNSKI F | 2 | 0.133 |
| 1967 | KU EJ | 2 | 0.133 |
| 1968 | KUDER J | 2 | 0.133 |
| 1969 | KUDER JF | 2 | 0.133 |
| 1970 | KUMAR A | 2 | 0.133 |
| 1971 | KUMAR N | 2 | 0.133 |
| 1972 | KUMEDA Y | 2 | 0.133 |
| 1973 | KUMWENDA M | 2 | 0.133 |
| 1974 | KUNO A | 2 | 0.133 |
| 1975 | KURIYAMA S | 2 | 0.133 |
| 1976 | KUSAKA H | 2 | 0.133 |
| 1977 | KUSAKABE T | 2 | 0.133 |
| 1978 | KUSUNOSE K | 2 | 0.133 |
| 1979 | KVITKOVA L | 2 | 0.133 |
| 1980 | LA SALA L | 2 | 0.133 |
| 1981 | LAFEUILLE MH | 2 | 0.133 |
| 1982 | LAINSCAK M | 2 | 0.133 |
| 1983 | LAJARA R | 2 | 0.133 |
| 1984 | LALIC K | 2 | 0.133 |
| 1985 | LALIC N | 2 | 0.133 |
| 1986 | LAM SW | 2 | 0.133 |
| 1987 | LAN NSR | 2 | 0.133 |
| 1988 | LANCHIOTTI PV | 2 | 0.133 |
| 1989 | LATIFF G | 2 | 0.133 |
| 1990 | LATVA-RASKU A | 2 | 0.133 |
| 1991 | LAYTON AT | 2 | 0.133 |
| 1992 | LAZUKA L | 2 | 0.133 |
| 1993 | LE MEUR Y | 2 | 0.133 |
| 1994 | LEE D | 2 | 0.133 |
| 1995 | LEE DH | 2 | 0.133 |
| 1996 | LEE G | 2 | 0.133 |
| 1997 | LEE H | 2 | 0.133 |
| 1998 | LEE KK | 2 | 0.133 |
| 1999 | LEE KW | 2 | 0.133 |
| 2000 | LEE LY | 2 | 0.133 |
| 2001 | LEE M | 2 | 0.133 |
| 2002 | LEE SH | 2 | 0.133 |
| 2003 | LEFEBVRE P | 2 | 0.133 |
| 2004 | LEGUN O | 2 | 0.133 |
| 2005 | LEHRKE M | 2 | 0.133 |
| 2006 | LEITER L | 2 | 0.133 |
| 2007 | LEONCINI G | 2 | 0.133 |
| 2008 | LEPOR NE | 2 | 0.133 |
| 2009 | LESLIE BR | 2 | 0.133 |
| 2010 | LI D | 2 | 0.133 |
| 2011 | LI DT | 2 | 0.133 |
| 2012 | LI GP | 2 | 0.133 |
| 2013 | LI WG | 2 | 0.133 |
| 2014 | LI XM | 2 | 0.133 |
| 2015 | LIANG J | 2 | 0.133 |
| 2016 | LIAO XX | 2 | 0.133 |
| 2017 | LIBIANTO R | 2 | 0.133 |
| 2018 | LIM SK | 2 | 0.133 |
| 2019 | LIN B | 2 | 0.133 |
| 2020 | LIN CC | 2 | 0.133 |
| 2021 | LIN HL | 2 | 0.133 |
| 2022 | LIN P | 2 | 0.133 |
| 2023 | LINDEN K | 2 | 0.133 |
| 2024 | LISBOA HRK | 2 | 0.133 |
| 2025 | LISTER S | 2 | 0.133 |
| 2026 | LIU B | 2 | 0.133 |
| 2027 | LIU H | 2 | 0.133 |
| 2028 | LIU ZM | 2 | 0.133 |
| 2029 | LJUNGMAN CEA | 2 | 0.133 |
| 2030 | LOH CL | 2 | 0.133 |
| 2031 | LOMBARD L | 2 | 0.133 |
| 2032 | LOMBARDI CM | 2 | 0.133 |
| 2033 | LOPATIN Y | 2 | 0.133 |
| 2034 | LOPEZ MD | 2 | 0.133 |
| 2035 | LOTAN C | 2 | 0.133 |
| 2036 | LOZA OTM | 2 | 0.133 |
| 2037 | LOZANO FF | 2 | 0.133 |
| 2038 | LOZANO MAT | 2 | 0.133 |
| 2039 | LOZANOV L | 2 | 0.133 |
| 2040 | LU WP | 2 | 0.133 |
| 2041 | LU YC | 2 | 0.133 |
| 2042 | LUCIJANIC T | 2 | 0.133 |
| 2043 | LUCKASEN GJ | 2 | 0.133 |
| 2044 | LUCONI M | 2 | 0.133 |
| 2045 | LUK A | 2 | 0.133 |
| 2046 | LUKAC M | 2 | 0.133 |
| 2047 | LUND LH | 2 | 0.133 |
| 2048 | LUPSA BC | 2 | 0.133 |
| 2049 | LUSCHER TF | 2 | 0.133 |
| 2050 | MA J | 2 | 0.133 |
| 2051 | MA M | 2 | 0.133 |
| 2052 | MA RCW | 2 | 0.133 |
| 2053 | MAACK C | 2 | 0.133 |
| 2054 | MACDOUGALL I | 2 | 0.133 |
| 2055 | MACHIMURA H | 2 | 0.133 |
| 2056 | MADAAN T | 2 | 0.133 |
| 2057 | MADDOX TM | 2 | 0.133 |
| 2058 | MADEJ A | 2 | 0.133 |
| 2059 | MADORE F | 2 | 0.133 |
| 2060 | MADUENO FJT | 2 | 0.133 |
| 2061 | MAEGAWA H | 2 | 0.133 |
| 2062 | MAGGIONI AP | 2 | 0.133 |
| 2063 | MAGLIANO DJ | 2 | 0.133 |
| 2064 | MAGRE J | 2 | 0.133 |
| 2065 | MAGWIRE ML | 2 | 0.133 |
| 2066 | MAH PM | 2 | 0.133 |
| 2067 | MAINOU M | 2 | 0.133 |
| 2068 | MAJOR L | 2 | 0.133 |
| 2069 | MALAVER N | 2 | 0.133 |
| 2070 | MALBERTI F | 2 | 0.133 |
| 2071 | MANCEUR AM | 2 | 0.133 |
| 2072 | MANCUSO JP | 2 | 0.133 |
| 2073 | MANDREOLI M | 2 | 0.133 |
| 2074 | MANIADAKIS N | 2 | 0.133 |
| 2075 | MANISHA S | 2 | 0.133 |
| 2076 | MANKOVSKYY B | 2 | 0.133 |
| 2077 | MANN J | 2 | 0.133 |
| 2078 | MANOCHA AB | 2 | 0.133 |
| 2079 | MANRIQUE A | 2 | 0.133 |
| 2080 | MAO H | 2 | 0.133 |
| 2081 | MARASAEV V | 2 | 0.133 |
| 2082 | MARCHETTA N | 2 | 0.133 |
| 2083 | MARCIANO C | 2 | 0.133 |
| 2084 | MARGARITOV V | 2 | 0.133 |
| 2085 | MARIN TM | 2 | 0.133 |
| 2086 | MARKLEY R | 2 | 0.133 |
| 2087 | MARSICO F | 2 | 0.133 |
| 2088 | MARTINEZ FJG | 2 | 0.133 |
| 2089 | MARTYNYUK L | 2 | 0.133 |
| 2090 | MARUYAMA N | 2 | 0.133 |
| 2091 | MASAKI T | 2 | 0.133 |
| 2092 | MASOUDI FA | 2 | 0.133 |
| 2093 | MASUDA T | 2 | 0.133 |
| 2094 | MATAWARAN BJ | 2 | 0.133 |
| 2095 | MATSUI T | 2 | 0.133 |
| 2096 | MATSUMOTO K | 2 | 0.133 |
| 2097 | MATSUMURA K | 2 | 0.133 |
| 2098 | MAURICIO D | 2 | 0.133 |
| 2099 | MAXEINER S | 2 | 0.133 |
| 2100 | MAZIDI M | 2 | 0.133 |
| 2101 | MAZUR S | 2 | 0.133 |
| 2102 | MCCULLOUGH P | 2 | 0.133 |
| 2103 | MCDONAGH T | 2 | 0.133 |
| 2104 | MCGILL JB | 2 | 0.133 |
| 2105 | MCGOVERN A | 2 | 0.133 |
| 2106 | MCMAHON A | 2 | 0.133 |
| 2107 | MEDINA J | 2 | 0.133 |
| 2108 | MEHTA SN | 2 | 0.133 |
| 2109 | MEIER JJ | 2 | 0.133 |
| 2110 | MELBY CL | 2 | 0.133 |
| 2111 | MELBYE M | 2 | 0.133 |
| 2112 | MELE P | 2 | 0.133 |
| 2113 | MELLBIN L | 2 | 0.133 |
| 2114 | MENDE C | 2 | 0.133 |
| 2115 | MENDOZA JLA | 2 | 0.133 |
| 2116 | MENG L | 2 | 0.133 |
| 2117 | MERCADAL LA | 2 | 0.133 |
| 2118 | MERTENS PR | 2 | 0.133 |
| 2119 | MERTON K | 2 | 0.133 |
| 2120 | MESA JV | 2 | 0.133 |
| 2121 | MEZZA T | 2 | 0.133 |
| 2122 | MIJANGOS JHS | 2 | 0.133 |
| 2123 | MILAGRES R | 2 | 0.133 |
| 2124 | MILEDER M | 2 | 0.133 |
| 2125 | MILNE N | 2 | 0.133 |
| 2126 | MINAMI I | 2 | 0.133 |
| 2127 | MISRA-HEBERT AD | 2 | 0.133 |
| 2128 | MIYAKAWA M | 2 | 0.133 |
| 2129 | MIYAMOTO Y | 2 | 0.133 |
| 2130 | MIYAUCHI K | 2 | 0.133 |
| 2131 | MIYAUCHI S | 2 | 0.133 |
| 2132 | MIYOSHI T | 2 | 0.133 |
| 2133 | MIZUYAMA K | 2 | 0.133 |
| 2134 | MKRTUMYAN A | 2 | 0.133 |
| 2135 | MLODAWSKA-CHOLUJ D | 2 | 0.133 |
| 2136 | MOCHIZUKI Y | 2 | 0.133 |
| 2137 | MOFFA S | 2 | 0.133 |
| 2138 | MOKUBO A | 2 | 0.133 |
| 2139 | MOLINA HL | 2 | 0.133 |
| 2140 | MOLNAR M | 2 | 0.133 |
| 2141 | MONTENEGRO MAF | 2 | 0.133 |
| 2142 | MONTENEGRO R | 2 | 0.133 |
| 2143 | MONTORI VM | 2 | 0.133 |
| 2144 | MORDI IR | 2 | 0.133 |
| 2145 | MORI K | 2 | 0.133 |
| 2146 | MORUGOVA T | 2 | 0.133 |
| 2147 | MOSTOVOY Y | 2 | 0.133 |
| 2148 | MUCSI J | 2 | 0.133 |
| 2149 | MUDALIAR S | 2 | 0.133 |
| 2150 | MUIRHEAD N | 2 | 0.133 |
| 2151 | MUNIR KM | 2 | 0.133 |
| 2152 | MUNOZ LDA | 2 | 0.133 |
| 2153 | MUNZEL T | 2 | 0.133 |
| 2154 | MURAKAMI M | 2 | 0.133 |
| 2155 | MURPHY AJ | 2 | 0.133 |
| 2156 | MUSSO G | 2 | 0.133 |
| 2157 | MUSZKAT M | 2 | 0.133 |
| 2158 | NAGAREDDY PR | 2 | 0.133 |
| 2159 | NAGATA D | 2 | 0.133 |
| 2160 | NAGIBOVICH G | 2 | 0.133 |
| 2161 | NAGIBOVICH O | 2 | 0.133 |
| 2162 | NAJMI AK | 2 | 0.133 |
| 2163 | NAKAGAWA T | 2 | 0.133 |
| 2164 | NAKAJIMA S | 2 | 0.133 |
| 2165 | NAKAMURA K | 2 | 0.133 |
| 2166 | NAKAMURA S | 2 | 0.133 |
| 2167 | NAKAMURA T | 2 | 0.133 |
| 2168 | NAKANO Y | 2 | 0.133 |
| 2169 | NAKATA K | 2 | 0.133 |
| 2170 | NAM MS | 2 | 0.133 |
| 2171 | NASIRI-ANSARI N | 2 | 0.133 |
| 2172 | NAUCK MA | 2 | 0.133 |
| 2173 | NAVICKAS A | 2 | 0.133 |
| 2174 | NEDOGODA S | 2 | 0.133 |
| 2175 | NEGRU D | 2 | 0.133 |
| 2176 | NESPOUX J | 2 | 0.133 |
| 2177 | NEUMILLER JJ | 2 | 0.133 |
| 2178 | NEWMAN AA | 2 | 0.133 |
| 2179 | NEWMAN J | 2 | 0.133 |
| 2180 | NG KS | 2 | 0.133 |
| 2181 | NGCAKANI N | 2 | 0.133 |
| 2182 | NICHOLLS K | 2 | 0.133 |
| 2183 | NICOLAI S | 2 | 0.133 |
| 2184 | NIE L | 2 | 0.133 |
| 2185 | NIIYA T | 2 | 0.133 |
| 2186 | NILSSON PM | 2 | 0.133 |
| 2187 | NISHIMURA T | 2 | 0.133 |
| 2188 | NIXON P | 2 | 0.133 |
| 2189 | NOMA H | 2 | 0.133 |
| 2190 | NOMOTO H | 2 | 0.133 |
| 2191 | NONCHEV B | 2 | 0.133 |
| 2192 | NOONAN P | 2 | 0.133 |
| 2193 | NORONHA I | 2 | 0.133 |
| 2194 | NOVIKOV A | 2 | 0.133 |
| 2195 | NOVOA PA | 2 | 0.133 |
| 2196 | NOWICKI M | 2 | 0.133 |
| 2197 | NUFFER WA | 2 | 0.133 |
| 2198 | NUNEZ J | 2 | 0.133 |
| 2199 | NUUTILA P | 2 | 0.133 |
| 2200 | O'HARE J | 2 | 0.133 |
| 2201 | OBANA M | 2 | 0.133 |
| 2202 | OBERDORF-MAASS SU | 2 | 0.133 |
| 2203 | OCHIAI H | 2 | 0.133 |
| 2204 | OCHODNICKA Z | 2 | 0.133 |
| 2205 | OELZE M | 2 | 0.133 |
| 2206 | OH CM | 2 | 0.133 |
| 2207 | OH KH | 2 | 0.133 |
| 2208 | OH TK | 2 | 0.133 |
| 2209 | OHMAN P | 2 | 0.133 |
| 2210 | OHNO K | 2 | 0.133 |
| 2211 | OHNO Y | 2 | 0.133 |
| 2212 | OISHI H | 2 | 0.133 |
| 2213 | OLGAR Y | 2 | 0.133 |
| 2214 | OLIVEIRA DC | 2 | 0.133 |
| 2215 | OMORI K | 2 | 0.133 |
| 2216 | ONACA AG | 2 | 0.133 |
| 2217 | ONO S | 2 | 0.133 |
| 2218 | OOSTHUIZEN H | 2 | 0.133 |
| 2219 | ORELLANA RAG | 2 | 0.133 |
| 2220 | ORIO SI | 2 | 0.133 |
| 2221 | ORLOWSKA-KOWALIK G | 2 | 0.133 |
| 2222 | OROSZLAN T | 2 | 0.133 |
| 2223 | OROZCO JATY | 2 | 0.133 |
| 2224 | ORTIZ LHG | 2 | 0.133 |
| 2225 | ORY I | 2 | 0.133 |
| 2226 | OSIPOVA I | 2 | 0.133 |
| 2227 | OTA S | 2 | 0.133 |
| 2228 | OTRERAS F | 2 | 0.133 |
| 2229 | OTT C | 2 | 0.133 |
| 2230 | OVIEDO A | 2 | 0.133 |
| 2231 | OZ M | 2 | 0.133 |
| 2232 | PABEL S | 2 | 0.133 |
| 2233 | PACKHAM D | 2 | 0.133 |
| 2234 | PAFILI K | 2 | 0.133 |
| 2235 | PAGIDIPATI N | 2 | 0.133 |
| 2236 | PALAZZUOLI A | 2 | 0.133 |
| 2237 | PALMER M | 2 | 0.133 |
| 2238 | PANAROTTO D | 2 | 0.133 |
| 2239 | PANENI F | 2 | 0.133 |
| 2240 | PAOLILLO S | 2 | 0.133 |
| 2241 | PAPAVASSILIOU AG | 2 | 0.133 |
| 2242 | PAPINI NR | 2 | 0.133 |
| 2243 | PARISSIS J | 2 | 0.133 |
| 2244 | PARK CY | 2 | 0.133 |
| 2245 | PARK JH | 2 | 0.133 |
| 2246 | PASCHEN B | 2 | 0.133 |
| 2247 | PASCHOU SA | 2 | 0.133 |
| 2248 | PASHKOVSKA N | 2 | 0.133 |
| 2249 | PASTERNAK B | 2 | 0.133 |
| 2250 | PATEL D | 2 | 0.133 |
| 2251 | PATEL DK | 2 | 0.133 |
| 2252 | PATEL S | 2 | 0.133 |
| 2253 | PATTI AM | 2 | 0.133 |
| 2254 | PATTZI HR | 2 | 0.133 |
| 2255 | PAWASKAR M | 2 | 0.133 |
| 2256 | PELLA D | 2 | 0.133 |
| 2257 | PENFORNIS A | 2 | 0.133 |
| 2258 | PEREIRA MA | 2 | 0.133 |
| 2259 | PEREIRA MJ | 2 | 0.133 |
| 2260 | PERERVA L | 2 | 0.133 |
| 2261 | PEREZ A | 2 | 0.133 |
| 2262 | PEREZ JMP | 2 | 0.133 |
| 2263 | PEREZ JP | 2 | 0.133 |
| 2264 | PERINGAT J | 2 | 0.133 |
| 2265 | PERREAULT L | 2 | 0.133 |
| 2266 | PERSEGHIN G | 2 | 0.133 |
| 2267 | PERTSEVA T | 2 | 0.133 |
| 2268 | PESICKOVA S | 2 | 0.133 |
| 2269 | PETERFAI E | 2 | 0.133 |
| 2270 | PETERKA K | 2 | 0.133 |
| 2271 | PETERSON E | 2 | 0.133 |
| 2272 | PETRICA L | 2 | 0.133 |
| 2273 | PETRIE JR | 2 | 0.133 |
| 2274 | PETRO G | 2 | 0.133 |
| 2275 | PETRY TBZ | 2 | 0.133 |
| 2276 | PETRYKIV S | 2 | 0.133 |
| 2277 | PEYTON KJ | 2 | 0.133 |
| 2278 | PHAM S | 2 | 0.133 |
| 2279 | PHILIP S | 2 | 0.133 |
| 2280 | PIATTI P | 2 | 0.133 |
| 2281 | PICHETTE V | 2 | 0.133 |
| 2282 | PILLAY-RAMAYA L | 2 | 0.133 |
| 2283 | PILON D | 2 | 0.133 |
| 2284 | PLASSMANN G | 2 | 0.133 |
| 2285 | PLAZA MV | 2 | 0.133 |
| 2286 | POCOCK S | 2 | 0.133 |
| 2287 | POCOCK SJ | 2 | 0.133 |
| 2288 | POKRAJAC A | 2 | 0.133 |
| 2289 | POLO JVS | 2 | 0.133 |
| 2290 | PONTREMOLI R | 2 | 0.133 |
| 2291 | POPA AR | 2 | 0.133 |
| 2292 | POPENDA G | 2 | 0.133 |
| 2293 | POPOVIC-RADINOVIC V | 2 | 0.133 |
| 2294 | PORRINI E | 2 | 0.133 |
| 2295 | PORTES ED | 2 | 0.133 |
| 2296 | POSTEMA R | 2 | 0.133 |
| 2297 | POWELL DR | 2 | 0.133 |
| 2298 | POWELL JR | 2 | 0.133 |
| 2299 | PRASAD N | 2 | 0.133 |
| 2300 | PRATLEY R | 2 | 0.133 |
| 2301 | PRATTICHIZZO F | 2 | 0.133 |
| 2302 | PREISS D | 2 | 0.133 |
| 2303 | PROIETTO J | 2 | 0.133 |
| 2304 | PROVENZANO M | 2 | 0.133 |
| 2305 | PROZESKY H | 2 | 0.133 |
| 2306 | PUMPRLA J | 2 | 0.133 |
| 2307 | QU XH | 2 | 0.133 |
| 2308 | QURESHI MA | 2 | 0.133 |
| 2309 | RABASA-LHORET R | 2 | 0.133 |
| 2310 | RADUAN RA | 2 | 0.133 |
| 2311 | RAFFAELE P | 2 | 0.133 |
| 2312 | RAHELIC D | 2 | 0.133 |
| 2313 | RAILEY M | 2 | 0.133 |
| 2314 | RAIMONDI L | 2 | 0.133 |
| 2315 | RAJANI A | 2 | 0.133 |
| 2316 | RAJEEV SP | 2 | 0.133 |
| 2317 | RAJPATHAK S | 2 | 0.133 |
| 2318 | RAMOS CS | 2 | 0.133 |
| 2319 | RAMOS JC | 2 | 0.133 |
| 2320 | RAMTOOLA S | 2 | 0.133 |
| 2321 | RANDEVA HS | 2 | 0.133 |
| 2322 | RANETTI AE | 2 | 0.133 |
| 2323 | RANGEL JDG | 2 | 0.133 |
| 2324 | RANGELOV R | 2 | 0.133 |
| 2325 | RASKINA T | 2 | 0.133 |
| 2326 | RATNASINGAM V | 2 | 0.133 |
| 2327 | REDDY J | 2 | 0.133 |
| 2328 | REN Y | 2 | 0.133 |
| 2329 | REN YL | 2 | 0.133 |
| 2330 | RENGA F | 2 | 0.133 |
| 2331 | RESK JH | 2 | 0.133 |
| 2332 | REVESZ K | 2 | 0.133 |
| 2333 | REWERSKA B | 2 | 0.133 |
| 2334 | RHEEDER P | 2 | 0.133 |
| 2335 | RICHE DM | 2 | 0.133 |
| 2336 | RIDDLE MC | 2 | 0.133 |
| 2337 | RIEG T | 2 | 0.133 |
| 2338 | RIERA MT | 2 | 0.133 |
| 2339 | RISTA L | 2 | 0.133 |
| 2340 | RIZVI AA | 2 | 0.133 |
| 2341 | ROBERTS A | 2 | 0.133 |
| 2342 | ROCA LAR | 2 | 0.133 |
| 2343 | ROCHA CM | 2 | 0.133 |
| 2344 | ROCHA NA | 2 | 0.133 |
| 2345 | ROCHA ND | 2 | 0.133 |
| 2346 | ROGER S | 2 | 0.133 |
| 2347 | ROSANO G | 2 | 0.133 |
| 2348 | ROSANO MAR | 2 | 0.133 |
| 2349 | ROSE L | 2 | 0.133 |
| 2350 | ROSERO R | 2 | 0.133 |
| 2351 | ROSOL ZP | 2 | 0.133 |
| 2352 | ROTHER KI | 2 | 0.133 |
| 2353 | ROTTER JRC | 2 | 0.133 |
| 2354 | RUILOPE LM | 2 | 0.133 |
| 2355 | RUIZ JEAD | 2 | 0.133 |
| 2356 | RUNGBY J | 2 | 0.133 |
| 2357 | RUOCCO G | 2 | 0.133 |
| 2358 | RUSCHITZKA F | 2 | 0.133 |
| 2359 | RYCHLIK I | 2 | 0.133 |
| 2360 | RYD N L | 2 | 0.133 |
| 2361 | RYDER REJ | 2 | 0.133 |
| 2362 | SAAD M | 2 | 0.133 |
| 2363 | SABA F | 2 | 0.133 |
| 2364 | SABBAG ARN | 2 | 0.133 |
| 2365 | SABONGUI S | 2 | 0.133 |
| 2366 | SABOVIC M | 2 | 0.133 |
| 2367 | SACHINIDIS A | 2 | 0.133 |
| 2368 | SAELY CH | 2 | 0.133 |
| 2369 | SAHAY M | 2 | 0.133 |
| 2370 | SAITO Y | 2 | 0.133 |
| 2371 | SAKAI H | 2 | 0.133 |
| 2372 | SAKAKIBARA T | 2 | 0.133 |
| 2373 | SALA J | 2 | 0.133 |
| 2374 | SAMBEVSKI S | 2 | 0.133 |
| 2375 | SAMOYLOVA Y | 2 | 0.133 |
| 2376 | SAMUKAWA Y | 2 | 0.133 |
| 2377 | SANCHES FCC | 2 | 0.133 |
| 2378 | SANCHEZ SBS | 2 | 0.133 |
| 2379 | SANO H | 2 | 0.133 |
| 2380 | SANSON PS | 2 | 0.133 |
| 2381 | SANTANA SSI | 2 | 0.133 |
| 2382 | SANTORO D | 2 | 0.133 |
| 2383 | SANTOS F | 2 | 0.133 |
| 2384 | SANTOS JC | 2 | 0.133 |
| 2385 | SANZ J | 2 | 0.133 |
| 2386 | SAPORITO W | 2 | 0.133 |
| 2387 | SARAFIDIS P | 2 | 0.133 |
| 2388 | SARAFIDIS PA | 2 | 0.133 |
| 2389 | SARAIVA JFK | 2 | 0.133 |
| 2390 | SARANGDHAR M | 2 | 0.133 |
| 2391 | SARGEANT JA | 2 | 0.133 |
| 2392 | SARIGIANNI M | 2 | 0.133 |
| 2393 | SASAHARA Y | 2 | 0.133 |
| 2394 | SATO K | 2 | 0.133 |
| 2395 | SATOH-ASAHARA N | 2 | 0.133 |
| 2396 | SATYANARAYANA RK | 2 | 0.133 |
| 2397 | SAUDEK F | 2 | 0.133 |
| 2398 | SAULNIER PJ | 2 | 0.133 |
| 2399 | SAXENA M | 2 | 0.133 |
| 2400 | SAZONOVA O | 2 | 0.133 |
| 2401 | SCHIAVI LB | 2 | 0.133 |
| 2402 | SCHINI-KERTH VB | 2 | 0.133 |
| 2403 | SCHMIEDER RE | 2 | 0.133 |
| 2404 | SCHNEE J | 2 | 0.133 |
| 2405 | SCHOENBERG HM | 2 | 0.133 |
| 2406 | SCHORK A | 2 | 0.133 |
| 2407 | SCHUCH T | 2 | 0.133 |
| 2408 | SCHULZE C | 2 | 0.133 |
| 2409 | SCHWANCK B | 2 | 0.133 |
| 2410 | SCHWARTZ SS | 2 | 0.133 |
| 2411 | SCHWARTZBARD AZ | 2 | 0.133 |
| 2412 | SCOTTON AS | 2 | 0.133 |
| 2413 | SEEBER M | 2 | 0.133 |
| 2414 | SEGHIERI M | 2 | 0.133 |
| 2415 | SEIDU S | 2 | 0.133 |
| 2416 | SENNIK D | 2 | 0.133 |
| 2417 | SERAFINCEANU C | 2 | 0.133 |
| 2418 | SERRANO AG | 2 | 0.133 |
| 2419 | SERVIDDIO G | 2 | 0.133 |
| 2420 | SESSA H | 2 | 0.133 |
| 2421 | SEWELL KA | 2 | 0.133 |
| 2422 | SEZAI A | 2 | 0.133 |
| 2423 | SHA S | 2 | 0.133 |
| 2424 | SHAMKHALOVA M | 2 | 0.133 |
| 2425 | SHEN Y | 2 | 0.133 |
| 2426 | SHIBATA S | 2 | 0.133 |
| 2427 | SHIMIZU W | 2 | 0.133 |
| 2428 | SHIMURA H | 2 | 0.133 |
| 2429 | SHINKOV A | 2 | 0.133 |
| 2430 | SHITE J | 2 | 0.133 |
| 2431 | SHOJIMA N | 2 | 0.133 |
| 2432 | SHRIWAS P | 2 | 0.133 |
| 2433 | SHUTEMOVA E | 2 | 0.133 |
| 2434 | SHWARTZ Y | 2 | 0.133 |
| 2435 | SIASOS G | 2 | 0.133 |
| 2436 | SILLJE HHW | 2 | 0.133 |
| 2437 | SILVA-CARDOSO J | 2 | 0.133 |
| 2438 | SIMON G | 2 | 0.133 |
| 2439 | SIMON MAQ | 2 | 0.133 |
| 2440 | SIMONS G | 2 | 0.133 |
| 2441 | SINGH B | 2 | 0.133 |
| 2442 | SINGH JSS | 2 | 0.133 |
| 2443 | SKELIN M | 2 | 0.133 |
| 2444 | SMATANOVA I | 2 | 0.133 |
| 2445 | SMIRNOV I | 2 | 0.133 |
| 2446 | SMITH SM | 2 | 0.133 |
| 2447 | SOEKI T | 2 | 0.133 |
| 2448 | SOGA F | 2 | 0.133 |
| 2449 | SOLEYMANLOU N | 2 | 0.133 |
| 2450 | SON C | 2 | 0.133 |
| 2451 | SONESSON C | 2 | 0.133 |
| 2452 | SONG WH | 2 | 0.133 |
| 2453 | SOSA FEE | 2 | 0.133 |
| 2454 | SOTO MB | 2 | 0.133 |
| 2455 | SOWERS JR | 2 | 0.133 |
| 2456 | SOWINSKI D | 2 | 0.133 |
| 2457 | SREEDHAR R | 2 | 0.133 |
| 2458 | SREELATHA M | 2 | 0.133 |
| 2459 | STEELE A | 2 | 0.133 |
| 2460 | STELLA A | 2 | 0.133 |
| 2461 | STEPHENS J | 2 | 0.133 |
| 2462 | STEVEN S | 2 | 0.133 |
| 2463 | STRIEPE K | 2 | 0.133 |
| 2464 | STRZELECKA A | 2 | 0.133 |
| 2465 | SUBRAMANIAN S | 2 | 0.133 |
| 2466 | SUDHAKAR B | 2 | 0.133 |
| 2467 | SUGAWARA H | 2 | 0.133 |
| 2468 | SUGITANI T | 2 | 0.133 |
| 2469 | SUGIURA T | 2 | 0.133 |
| 2470 | SUGIYAMA S | 2 | 0.133 |
| 2471 | SUISSA S | 2 | 0.133 |
| 2472 | SUN F | 2 | 0.133 |
| 2473 | SUN L | 2 | 0.133 |
| 2474 | SUNG SH | 2 | 0.133 |
| 2475 | SUZUKI M | 2 | 0.133 |
| 2476 | SVANSTROM H | 2 | 0.133 |
| 2477 | SVYSHCHENKO Y | 2 | 0.133 |
| 2478 | SYMONDS R | 2 | 0.133 |
| 2479 | SZALAT A | 2 | 0.133 |
| 2480 | TAHRANI AA | 2 | 0.133 |
| 2481 | TAKACS R | 2 | 0.133 |
| 2482 | TAKAHASHI S | 2 | 0.133 |
| 2483 | TAKAI M | 2 | 0.133 |
| 2484 | TAKAOKA H | 2 | 0.133 |
| 2485 | TAKEDA H | 2 | 0.133 |
| 2486 | TAKEUCHI T | 2 | 0.133 |
| 2487 | TANAKA M | 2 | 0.133 |
| 2488 | TANAKA T | 2 | 0.133 |
| 2489 | TANG WF | 2 | 0.133 |
| 2490 | TANKOVA T | 2 | 0.133 |
| 2491 | TATSUMI K | 2 | 0.133 |
| 2492 | TAUB PR | 2 | 0.133 |
| 2493 | TEMELKOVA M | 2 | 0.133 |
| 2494 | TENTOLOURIS A | 2 | 0.133 |
| 2495 | TERAGAWA H | 2 | 0.133 |
| 2496 | TERASHIMA M | 2 | 0.133 |
| 2497 | TESAR V | 2 | 0.133 |
| 2498 | THAI K | 2 | 0.133 |
| 2499 | THEODORAKIS MJ | 2 | 0.133 |
| 2500 | THERVET E | 2 | 0.133 |
| 2501 | THOMAS MC | 2 | 0.133 |
| 2502 | THOME F | 2 | 0.133 |
| 2503 | THRASHER J | 2 | 0.133 |
| 2504 | TOARBA C | 2 | 0.133 |
| 2505 | TOKI H | 2 | 0.133 |
| 2506 | TOLENTINO M | 2 | 0.133 |
| 2507 | TOLLEY K | 2 | 0.133 |
| 2508 | TOMASHKEVYCH H | 2 | 0.133 |
| 2509 | TOMITANI N | 2 | 0.133 |
| 2510 | TOMLINSON LA | 2 | 0.133 |
| 2511 | TOMONAGA O | 2 | 0.133 |
| 2512 | TOPCHII I | 2 | 0.133 |
| 2513 | TORRES MM | 2 | 0.133 |
| 2514 | TORSHIZI A | 2 | 0.133 |
| 2515 | TOWNSEND RR | 2 | 0.133 |
| 2516 | TOYAMA K | 2 | 0.133 |
| 2517 | TRANKLE CR | 2 | 0.133 |
| 2518 | TREVISAN R | 2 | 0.133 |
| 2519 | TRIANA EH | 2 | 0.133 |
| 2520 | TRIGGLE CR | 2 | 0.133 |
| 2521 | TRUJILLO JM | 2 | 0.133 |
| 2522 | TRUM M | 2 | 0.133 |
| 2523 | TRYSHCHUK N | 2 | 0.133 |
| 2524 | TSAI SF | 2 | 0.133 |
| 2525 | TSANG MW | 2 | 0.133 |
| 2526 | TSANG V | 2 | 0.133 |
| 2527 | TSIOUFIS C | 2 | 0.133 |
| 2528 | TSIOUFIS K | 2 | 0.133 |
| 2529 | TSUJIMOTO M | 2 | 0.133 |
| 2530 | TU P | 2 | 0.133 |
| 2531 | TURAN B | 2 | 0.133 |
| 2532 | TURATTI LAA | 2 | 0.133 |
| 2533 | TURNER W | 2 | 0.133 |
| 2534 | TZANETAKOS C | 2 | 0.133 |
| 2535 | TZERAVINI E | 2 | 0.133 |
| 2536 | TZIOMALOS K | 2 | 0.133 |
| 2537 | UEDA P | 2 | 0.133 |
| 2538 | UEKAWA K | 2 | 0.133 |
| 2539 | ULLA MR | 2 | 0.133 |
| 2540 | UMEZAWA S | 2 | 0.133 |
| 2541 | UMEZONO T | 2 | 0.133 |
| 2542 | UMPIERREZ G | 2 | 0.133 |
| 2543 | URBANAVICIENE E | 2 | 0.133 |
| 2544 | URIASYEV O | 2 | 0.133 |
| 2545 | VALDEZ M | 2 | 0.133 |
| 2546 | VALENSI P | 2 | 0.133 |
| 2547 | VALENZUELA A | 2 | 0.133 |
| 2548 | VALIS M | 2 | 0.133 |
| 2549 | VALLEJOS A | 2 | 0.133 |
| 2550 | VAN BAAR MJB | 2 | 0.133 |
| 2551 | VAN BOMMEL EJM | 2 | 0.133 |
| 2552 | VAN GOOR H | 2 | 0.133 |
| 2553 | VAN SCHYNDLE J | 2 | 0.133 |
| 2554 | VAN TASSELL BW | 2 | 0.133 |
| 2555 | VAN VELDHUISEN DJ | 2 | 0.133 |
| 2556 | VANGEL S | 2 | 0.133 |
| 2557 | VARDENY O | 2 | 0.133 |
| 2558 | VASAS S | 2 | 0.133 |
| 2559 | VEJAR M | 2 | 0.133 |
| 2560 | VELICHKOVA E | 2 | 0.133 |
| 2561 | VENCIO SAC | 2 | 0.133 |
| 2562 | VENGADASALAM P | 2 | 0.133 |
| 2563 | VERA GO | 2 | 0.133 |
| 2564 | VERA MP | 2 | 0.133 |
| 2565 | VERONELLI AM | 2 | 0.133 |
| 2566 | VETTOR R | 2 | 0.133 |
| 2567 | VIAZZI F | 2 | 0.133 |
| 2568 | VICKNESON K | 2 | 0.133 |
| 2569 | VILA MAM | 2 | 0.133 |
| 2570 | VILJOEN A | 2 | 0.133 |
| 2571 | VILLARINO A | 2 | 0.133 |
| 2572 | VILLEGAS I | 2 | 0.133 |
| 2573 | VIRANI SS | 2 | 0.133 |
| 2574 | VISCO VE | 2 | 0.133 |
| 2575 | VIVIAN EM | 2 | 0.133 |
| 2576 | VIZIR V | 2 | 0.133 |
| 2577 | VLASENKO M | 2 | 0.133 |
| 2578 | VOLLERT H | 2 | 0.133 |
| 2579 | VOLTERRANI M | 2 | 0.133 |
| 2580 | VOROBYEV S | 2 | 0.133 |
| 2581 | VYASAM RC | 2 | 0.133 |
| 2582 | WAHBA M | 2 | 0.133 |
| 2583 | WAKASUGI M | 2 | 0.133 |
| 2584 | WAKELING J | 2 | 0.133 |
| 2585 | WALKER R | 2 | 0.133 |
| 2586 | WALLNER M | 2 | 0.133 |
| 2587 | WAN NN | 2 | 0.133 |
| 2588 | WANG B | 2 | 0.133 |
| 2589 | WANG CY | 2 | 0.133 |
| 2590 | WANG GX | 2 | 0.133 |
| 2591 | WANG TS | 2 | 0.133 |
| 2592 | WARD T | 2 | 0.133 |
| 2593 | WASSERMANN A | 2 | 0.133 |
| 2594 | WATSON E | 2 | 0.133 |
| 2595 | WATTS NB | 2 | 0.133 |
| 2596 | WAYS K | 2 | 0.133 |
| 2597 | WEEDA ER | 2 | 0.133 |
| 2598 | WESTENBRINK BD | 2 | 0.133 |
| 2599 | WHALEY-CONNELL A | 2 | 0.133 |
| 2600 | WHEELER D | 2 | 0.133 |
| 2601 | WIECEK A | 2 | 0.133 |
| 2602 | WIGGERS H | 2 | 0.133 |
| 2603 | WILBURN JR | 2 | 0.133 |
| 2604 | WILCOX CS | 2 | 0.133 |
| 2605 | WILKINSON MJ | 2 | 0.133 |
| 2606 | WILKINSON S | 2 | 0.133 |
| 2607 | WILLIAMS M | 2 | 0.133 |
| 2608 | WILLIAMSON E | 2 | 0.133 |
| 2609 | WINTZELL V | 2 | 0.133 |
| 2610 | WITTBRODT ET | 2 | 0.133 |
| 2611 | WONG AC | 2 | 0.133 |
| 2612 | WONG MG | 2 | 0.133 |
| 2613 | WOODWARD M | 2 | 0.133 |
| 2614 | WRIGHT EM | 2 | 0.133 |
| 2615 | WU JHY | 2 | 0.133 |
| 2616 | XU GS | 2 | 0.133 |
| 2617 | XU L | 2 | 0.133 |
| 2618 | XU Y | 2 | 0.133 |
| 2619 | YABE D | 2 | 0.133 |
| 2620 | YAGI S | 2 | 0.133 |
| 2621 | YAJIMA T | 2 | 0.133 |
| 2622 | YAKOV A | 2 | 0.133 |
| 2623 | YAMADA S | 2 | 0.133 |
| 2624 | YANAGIDA K | 2 | 0.133 |
| 2625 | YANG DY | 2 | 0.133 |
| 2626 | YANG HC | 2 | 0.133 |
| 2627 | YANG JK | 2 | 0.133 |
| 2628 | YANG WJ | 2 | 0.133 |
| 2629 | YANG YQ | 2 | 0.133 |
| 2630 | YAO C | 2 | 0.133 |
| 2631 | YAP YE | 2 | 0.133 |
| 2632 | YASUDA S | 2 | 0.133 |
| 2633 | YE W | 2 | 0.133 |
| 2634 | YE YL | 2 | 0.133 |
| 2635 | YEAP BB | 2 | 0.133 |
| 2636 | YEE J | 2 | 0.133 |
| 2637 | YGPUARA MDL | 2 | 0.133 |
| 2638 | YIN AP | 2 | 0.133 |
| 2639 | YOKOTE K | 2 | 0.133 |
| 2640 | YOSHIDA A | 2 | 0.133 |
| 2641 | YOSHIDA M | 2 | 0.133 |
| 2642 | YOSHIDA Y | 2 | 0.133 |
| 2643 | YOSHII H | 2 | 0.133 |
| 2644 | YOSHIMOTO T | 2 | 0.133 |
| 2645 | YOUNG S | 2 | 0.133 |
| 2646 | YOUNK LM | 2 | 0.133 |
| 2647 | YU MX | 2 | 0.133 |
| 2648 | YU XQ | 2 | 0.133 |
| 2649 | YUAN Z | 2 | 0.133 |
| 2650 | YUMITA W | 2 | 0.133 |
| 2651 | YUPANQUI H | 2 | 0.133 |
| 2652 | YURISTA SR | 2 | 0.133 |
| 2653 | YUSOF MDC | 2 | 0.133 |
| 2654 | ZADOURIAN A | 2 | 0.133 |
| 2655 | ZAIDMAN CJ | 2 | 0.133 |
| 2656 | ZALUNARDO N | 2 | 0.133 |
| 2657 | ZANELLA MT | 2 | 0.133 |
| 2658 | ZANOLI L | 2 | 0.133 |
| 2659 | ZATEYSHCHIKOVA A | 2 | 0.133 |
| 2660 | ZATEYSHSHIKOV D | 2 | 0.133 |
| 2661 | ZEMEK S | 2 | 0.133 |
| 2662 | ZHANG JW | 2 | 0.133 |
| 2663 | ZHANG SM | 2 | 0.133 |
| 2664 | ZHANG YL | 2 | 0.133 |
| 2665 | ZHAO CH | 2 | 0.133 |
| 2666 | ZHAO D | 2 | 0.133 |
| 2667 | ZHAO MH | 2 | 0.133 |
| 2668 | ZHAO Y | 2 | 0.133 |
| 2669 | ZHENG HG | 2 | 0.133 |
| 2670 | ZHOU Y | 2 | 0.133 |
| 2671 | ZHUANG XD | 2 | 0.133 |
| 2672 | ZIEROTH S | 2 | 0.133 |
| 2673 | ZLOVA T | 2 | 0.133 |
| 2674 | ZOGRAFOU I | 2 | 0.133 |
| 2675 | ZSOM M | 2 | 0.133 |
| 2676 | ZUB L | 2 | 0.133 |
| 2677 | ZYKOVA T | 2 | 0.133 |
| 2678 | ABAD-JIMENEZ Z | 1 | 0.066 |
| 2679 | ABASCAL V | 1 | 0.066 |
| 2680 | ABBAS J | 1 | 0.066 |
| 2681 | ABBAS M | 1 | 0.066 |
| 2682 | ABDELGADIR E | 1 | 0.066 |
| 2683 | ABDULLAH AM | 1 | 0.066 |
| 2684 | ABDURRACHIM D | 1 | 0.066 |
| 2685 | ABE M | 1 | 0.066 |
| 2686 | ABEYRATNE A | 1 | 0.066 |
| 2687 | ABHAYARATNA WP | 1 | 0.066 |
| 2688 | ABIDI E | 1 | 0.066 |
| 2689 | ABIKO A | 1 | 0.066 |
| 2690 | ABIRU N | 1 | 0.066 |
| 2691 | ABLASSER K | 1 | 0.066 |
| 2692 | ABOALHASAN E | 1 | 0.066 |
| 2693 | ABOU-JOKH C | 1 | 0.066 |
| 2694 | ABOYANS V | 1 | 0.066 |
| 2695 | ABRAHAM NG | 1 | 0.066 |
| 2696 | ABRAHAM W | 1 | 0.066 |
| 2697 | ABRAHAM WT | 1 | 0.066 |
| 2698 | ABRAHAMSEN I | 1 | 0.066 |
| 2699 | ABRAMS H | 1 | 0.066 |
| 2700 | ABREU C | 1 | 0.066 |
| 2701 | ABREU M | 1 | 0.066 |
| 2702 | ABUZAID A | 1 | 0.066 |
| 2703 | ACKERMANN RT | 1 | 0.066 |
| 2704 | ACKERMANS MT | 1 | 0.066 |
| 2705 | ADAMCZAK M | 1 | 0.066 |
| 2706 | ADAMO M | 1 | 0.066 |
| 2707 | ADEGHATE E | 1 | 0.066 |
| 2708 | ADEGHATE EA | 1 | 0.066 |
| 2709 | ADEMI Z | 1 | 0.066 |
| 2710 | ADI F | 1 | 0.066 |
| 2711 | ADINGUPU DD | 1 | 0.066 |
| 2712 | ADLER S | 1 | 0.066 |
| 2713 | ADRIO B | 1 | 0.066 |
| 2714 | ADVANI A | 1 | 0.066 |
| 2715 | AFSAR B | 1 | 0.066 |
| 2716 | AGEWALL S | 1 | 0.066 |
| 2717 | AGHAR-JAFFAR R | 1 | 0.066 |
| 2718 | AGIRO A | 1 | 0.066 |
| 2719 | AGOSTONI P | 1 | 0.066 |
| 2720 | AGRA RM | 1 | 0.066 |
| 2721 | AGRA-BERMEJO RM | 1 | 0.066 |
| 2722 | AGRAWAL A | 1 | 0.066 |
| 2723 | AGRAWAL V | 1 | 0.066 |
| 2724 | AGRAWAL YO | 1 | 0.066 |
| 2725 | AGRAZ I | 1 | 0.066 |
| 2726 | AHMAD F | 1 | 0.066 |
| 2727 | AHMAD T | 1 | 0.066 |
| 2728 | AHMED HM | 1 | 0.066 |
| 2729 | AHMED I | 1 | 0.066 |
| 2730 | AHMED M | 1 | 0.066 |
| 2731 | AHN CH | 1 | 0.066 |
| 2732 | AHN YB | 1 | 0.066 |
| 2733 | AHN YK | 1 | 0.066 |
| 2734 | AHUJA T | 1 | 0.066 |
| 2735 | AI M | 1 | 0.066 |
| 2736 | AIHARA K | 1 | 0.066 |
| 2737 | AIKAWA T | 1 | 0.066 |
| 2738 | AIMARETTI G | 1 | 0.066 |
| 2739 | AIZAWA Y | 1 | 0.066 |
| 2740 | AKAGI S | 1 | 0.066 |
| 2741 | AKAIKE M | 1 | 0.066 |
| 2742 | AKAIKE T | 1 | 0.066 |
| 2743 | AKAMINE T | 1 | 0.066 |
| 2744 | AKAZAWA K | 1 | 0.066 |
| 2745 | AKERBLOM A | 1 | 0.066 |
| 2746 | AKHTAR T | 1 | 0.066 |
| 2747 | AKIMOTO T | 1 | 0.066 |
| 2748 | AKIYAMA N | 1 | 0.066 |
| 2749 | AKKUS E | 1 | 0.066 |
| 2750 | AKKUS G | 1 | 0.066 |
| 2751 | AKKUS O | 1 | 0.066 |
| 2752 | AKO J | 1 | 0.066 |
| 2753 | AKTURK HK | 1 | 0.066 |
| 2754 | AL HAKIM M | 1 | 0.066 |
| 2755 | AL KHUSHAYM NM | 1 | 0.066 |
| 2756 | AL RIFAI M | 1 | 0.066 |
| 2757 | AL YAMI MS | 1 | 0.066 |
| 2758 | AL-ANI M | 1 | 0.066 |
| 2759 | AL-HESAYEN A | 1 | 0.066 |
| 2760 | AL-MALLAH MH | 1 | 0.066 |
| 2761 | AL-OMARY HL | 1 | 0.066 |
| 2762 | ALABI OD | 1 | 0.066 |
| 2763 | ALAM M | 1 | 0.066 |
| 2764 | ALAM MF | 1 | 0.066 |
| 2765 | ALAM U | 1 | 0.066 |
| 2766 | ALATAKI S | 1 | 0.066 |
| 2767 | ALAWADI F | 1 | 0.066 |
| 2768 | ALBAI A | 1 | 0.066 |
| 2769 | ALBER H | 1 | 0.066 |
| 2770 | ALBERS GW | 1 | 0.066 |
| 2771 | ALBERT NM | 1 | 0.066 |
| 2772 | ALEGRIA E | 1 | 0.066 |
| 2773 | ALEGUAS A | 1 | 0.066 |
| 2774 | ALEKSIC I | 1 | 0.066 |
| 2775 | ALEMAN JO | 1 | 0.066 |
| 2776 | ALEMU R | 1 | 0.066 |
| 2777 | ALEPPO G | 1 | 0.066 |
| 2778 | ALESSI F | 1 | 0.066 |
| 2779 | ALEXANDRE L | 1 | 0.066 |
| 2780 | ALEXANDROU A | 1 | 0.066 |
| 2781 | ALEXANDROU ME | 1 | 0.066 |
| 2782 | ALFAYEZ OM | 1 | 0.066 |
| 2783 | ALI H | 1 | 0.066 |
| 2784 | ALI S | 1 | 0.066 |
| 2785 | ALJABRI BA | 1 | 0.066 |
| 2786 | ALKHATIB N | 1 | 0.066 |
| 2787 | ALKINDI F | 1 | 0.066 |
| 2788 | ALLAN GM | 1 | 0.066 |
| 2789 | ALLEGRETTI AS | 1 | 0.066 |
| 2790 | ALLEN L | 1 | 0.066 |
| 2791 | ALLOJU S | 1 | 0.066 |
| 2792 | ALMABROUK TA | 1 | 0.066 |
| 2793 | ALONSO N | 1 | 0.066 |
| 2794 | ALPERIN P | 1 | 0.066 |
| 2795 | ALSHEIKH R | 1 | 0.066 |
| 2796 | ALSHIBANI M | 1 | 0.066 |
| 2797 | ALSTON LA | 1 | 0.066 |
| 2798 | ALTARA R | 1 | 0.066 |
| 2799 | ALTUCCI L | 1 | 0.066 |
| 2800 | ALVA H | 1 | 0.066 |
| 2801 | ALVAREZ B | 1 | 0.066 |
| 2802 | ALVAREZ BV | 1 | 0.066 |
| 2803 | ALVES-MARTINEZ P | 1 | 0.066 |
| 2804 | AMAMOTO M | 1 | 0.066 |
| 2805 | AMBERNTSSON R | 1 | 0.066 |
| 2806 | AMBROSIO A | 1 | 0.066 |
| 2807 | AMERI P | 1 | 0.066 |
| 2808 | AMIGO A | 1 | 0.066 |
| 2809 | AMIN KFM | 1 | 0.066 |
| 2810 | AMOROSI A | 1 | 0.066 |
| 2811 | AMOURA L | 1 | 0.066 |
| 2812 | ANABTAWI A | 1 | 0.066 |
| 2813 | ANAI M | 1 | 0.066 |
| 2814 | ANDERSON A | 1 | 0.066 |
| 2815 | ANDERSON J | 1 | 0.066 |
| 2816 | ANDERSON JE | 1 | 0.066 |
| 2817 | ANDERSON SG | 1 | 0.066 |
| 2818 | ANDO S | 1 | 0.066 |
| 2819 | ANDREOZZI F | 1 | 0.066 |
| 2820 | ANDREUCCI M | 1 | 0.066 |
| 2821 | ANDREW CA | 1 | 0.066 |
| 2822 | ANDREWS D | 1 | 0.066 |
| 2823 | ANDRIANESIS V | 1 | 0.066 |
| 2824 | ANDRIKOU E | 1 | 0.066 |
| 2825 | ANDROULAKIS ES | 1 | 0.066 |
| 2826 | ANDRUKHOVA O | 1 | 0.066 |
| 2827 | ANICHINI R | 1 | 0.066 |
| 2828 | ANKER MS | 1 | 0.066 |
| 2829 | ANONYMOUS | 1 | 0.066 |
| 2830 | ANOTHAISINTAWEE T | 1 | 0.066 |
| 2831 | ANSARI SA | 1 | 0.066 |
| 2832 | ANSTROM KJ | 1 | 0.066 |
| 2833 | ANTO-MICHEL N | 1 | 0.066 |
| 2834 | ANTOKU S | 1 | 0.066 |
| 2835 | ANTONIO RS | 1 | 0.066 |
| 2836 | ANTONOPOULOS AS | 1 | 0.066 |
| 2837 | ANTZA C | 1 | 0.066 |
| 2838 | ANWER T | 1 | 0.066 |
| 2839 | ANZAI K | 1 | 0.066 |
| 2840 | AOKI K | 1 | 0.066 |
| 2841 | AOKI T | 1 | 0.066 |
| 2842 | AONUMA K | 1 | 0.066 |
| 2843 | ARAD M | 1 | 0.066 |
| 2844 | ARAGON-HERRERA A | 1 | 0.066 |
| 2845 | ARAKAWA K | 1 | 0.066 |
| 2846 | ARAKI A | 1 | 0.066 |
| 2847 | ARASE K | 1 | 0.066 |
| 2848 | ARASE Y | 1 | 0.066 |
| 2849 | ARAVIND SR | 1 | 0.066 |
| 2850 | ARAVOT D | 1 | 0.066 |
| 2851 | ARENA S | 1 | 0.066 |
| 2852 | ARIETA CEL | 1 | 0.066 |
| 2853 | ARIKAN AA | 1 | 0.066 |
| 2854 | ARINO B | 1 | 0.066 |
| 2855 | ARNAOUT A | 1 | 0.066 |
| 2856 | ARNOLDS S | 1 | 0.066 |
| 2857 | ARONNE LJ | 1 | 0.066 |
| 2858 | AROW M | 1 | 0.066 |
| 2859 | ARSENAULT BJ | 1 | 0.066 |
| 2860 | ARTUNC F | 1 | 0.066 |
| 2861 | ARYA N | 1 | 0.066 |
| 2862 | ASAI K | 1 | 0.066 |
| 2863 | ASBERG A | 1 | 0.066 |
| 2864 | ASGAR AW | 1 | 0.066 |
| 2865 | ASHRAF GM | 1 | 0.066 |
| 2866 | ASHRAFZADEH S | 1 | 0.066 |
| 2867 | ASICO LD | 1 | 0.066 |
| 2868 | ASIRVATHAM A | 1 | 0.066 |
| 2869 | ASLEH R | 1 | 0.066 |
| 2870 | ASPROMONTE N | 1 | 0.066 |
| 2871 | ASSAF N | 1 | 0.066 |
| 2872 | ASSALY R | 1 | 0.066 |
| 2873 | ASSEBURG C | 1 | 0.066 |
| 2874 | ATAGELDIYEVA K | 1 | 0.066 |
| 2875 | ATALLAH-LAJAM F | 1 | 0.066 |
| 2876 | ATHERTON JJ | 1 | 0.066 |
| 2877 | ATISSO CM | 1 | 0.066 |
| 2878 | AUDEHM R | 1 | 0.066 |
| 2879 | AUGUST P | 1 | 0.066 |
| 2880 | AVEZUM A | 1 | 0.066 |
| 2881 | AVGERINOS K | 1 | 0.066 |
| 2882 | AWAL H | 1 | 0.066 |
| 2883 | AYER A | 1 | 0.066 |
| 2884 | AYESHA FNU | 1 | 0.066 |
| 2885 | AYLSWORTH A | 1 | 0.066 |
| 2886 | AYON R | 1 | 0.066 |
| 2887 | AYUB MT | 1 | 0.066 |
| 2888 | AZIM S | 1 | 0.066 |
| 2889 | AZIZI M | 1 | 0.066 |
| 2890 | AZOULAY L | 1 | 0.066 |
| 2891 | AZUMA K | 1 | 0.066 |
| 2892 | AZUSHIMA K | 1 | 0.066 |
| 2893 | BABA Y | 1 | 0.066 |
| 2894 | BABSKY AM | 1 | 0.066 |
| 2895 | BABU A | 1 | 0.066 |
| 2896 | BACCHUS S | 1 | 0.066 |
| 2897 | BACH LA | 1 | 0.066 |
| 2898 | BACHARAKI D | 1 | 0.066 |
| 2899 | BADARAU S | 1 | 0.066 |
| 2900 | BADRELDIN HA | 1 | 0.066 |
| 2901 | BADVE SV | 1 | 0.066 |
| 2902 | BAE JH | 1 | 0.066 |
| 2903 | BAEZA S | 1 | 0.066 |
| 2904 | BAGEPALLY BS | 1 | 0.066 |
| 2905 | BAGGER JI | 1 | 0.066 |
| 2906 | BAGIAS C | 1 | 0.066 |
| 2907 | BAIG F | 1 | 0.066 |
| 2908 | BAIK SH | 1 | 0.066 |
| 2909 | BAIN SC | 1 | 0.066 |
| 2910 | BAJESTANI SN | 1 | 0.066 |
| 2911 | BAKER AH | 1 | 0.066 |
| 2912 | BAKER HE | 1 | 0.066 |
| 2913 | BAKER ML | 1 | 0.066 |
| 2914 | BAKHAI A | 1 | 0.066 |
| 2915 | BAKKER SJL | 1 | 0.066 |
| 2916 | BAKOGIANNIS K | 1 | 0.066 |
| 2917 | BALAFAS E | 1 | 0.066 |
| 2918 | BALAMPANIS K | 1 | 0.066 |
| 2919 | BALAMURUGAN R | 1 | 0.066 |
| 2920 | BALDASSARRE MPA | 1 | 0.066 |
| 2921 | BALDERESCHI GI | 1 | 0.066 |
| 2922 | BALFOUR PC | 1 | 0.066 |
| 2923 | BALIS D | 1 | 0.066 |
| 2924 | BALIS DA | 1 | 0.066 |
| 2925 | BALOGH E | 1 | 0.066 |
| 2926 | BALTEAU M | 1 | 0.066 |
| 2927 | BAMIAS A | 1 | 0.066 |
| 2928 | BAN HZ | 1 | 0.066 |
| 2929 | BANERJEE A | 1 | 0.066 |
| 2930 | BANSILAL S | 1 | 0.066 |
| 2931 | BANULS C | 1 | 0.066 |
| 2932 | BARAKAT AF | 1 | 0.066 |
| 2933 | BARBARO G | 1 | 0.066 |
| 2934 | BARBETSEAS J | 1 | 0.066 |
| 2935 | BARBOSA FT | 1 | 0.066 |
| 2936 | BARDIA A | 1 | 0.066 |
| 2937 | BARKATE H | 1 | 0.066 |
| 2938 | BARRAL L | 1 | 0.066 |
| 2939 | BARRETT TJ | 1 | 0.066 |
| 2940 | BARRIO JR | 1 | 0.066 |
| 2941 | BARRON B | 1 | 0.066 |
| 2942 | BARRON J | 1 | 0.066 |
| 2943 | BARRY AR | 1 | 0.066 |
| 2944 | BARRY MJ | 1 | 0.066 |
| 2945 | BARUTTA F | 1 | 0.066 |
| 2946 | BARZEL B | 1 | 0.066 |
| 2947 | BASHIER A | 1 | 0.066 |
| 2948 | BASILE J | 1 | 0.066 |
| 2949 | BASILE JN | 1 | 0.066 |
| 2950 | BASSI N | 1 | 0.066 |
| 2951 | BASU S | 1 | 0.066 |
| 2952 | BATCHU SN | 1 | 0.066 |
| 2953 | BATZIAS K | 1 | 0.066 |
| 2954 | BAUD G | 1 | 0.066 |
| 2955 | BAUMAN J | 1 | 0.066 |
| 2956 | BAUMAN V | 1 | 0.066 |
| 2957 | BAVRY AA | 1 | 0.066 |
| 2958 | BAWEJA P | 1 | 0.066 |
| 2959 | BAXTER CA | 1 | 0.066 |
| 2960 | BAYCELEBI G | 1 | 0.066 |
| 2961 | BAYES-GENIS A | 1 | 0.066 |
| 2962 | BAYS H | 1 | 0.066 |
| 2963 | BAYS HE | 1 | 0.066 |
| 2964 | BAZZANO AN | 1 | 0.066 |
| 2965 | BEAULOYE C | 1 | 0.066 |
| 2966 | BEDIR RF | 1 | 0.066 |
| 2967 | BEEKMAN-HENDRIKS WL | 1 | 0.066 |
| 2968 | BEHETS-WYDEMANS G | 1 | 0.066 |
| 2969 | BEHR-ROUSSEL D | 1 | 0.066 |
| 2970 | BEHRENDT M | 1 | 0.066 |
| 2971 | BEKKI M | 1 | 0.066 |
| 2972 | BELCASTRO E | 1 | 0.066 |
| 2973 | BELENCHIA A | 1 | 0.066 |
| 2974 | BELKE DD | 1 | 0.066 |
| 2975 | BELL A | 1 | 0.066 |
| 2976 | BELL DA | 1 | 0.066 |
| 2977 | BELL JS | 1 | 0.066 |
| 2978 | BELL KF | 1 | 0.066 |
| 2979 | BELL M | 1 | 0.066 |
| 2980 | BELL RM | 1 | 0.066 |
| 2981 | BELLO F | 1 | 0.066 |
| 2982 | BEMBERG GB | 1 | 0.066 |
| 2983 | BEN GAL T | 1 | 0.066 |
| 2984 | BENDER SB | 1 | 0.066 |
| 2985 | BENETTI E | 1 | 0.066 |
| 2986 | BENGEL P | 1 | 0.066 |
| 2987 | BENKE K | 1 | 0.066 |
| 2988 | BENKO R | 1 | 0.066 |
| 2989 | BENNETT C | 1 | 0.066 |
| 2990 | BENRAHLA D | 1 | 0.066 |
| 2991 | BENSIMHON HF | 1 | 0.066 |
| 2992 | BERALL M | 1 | 0.066 |
| 2993 | BERCHIALLA P | 1 | 0.066 |
| 2994 | BERENGUER R | 1 | 0.066 |
| 2995 | BERG DD | 1 | 0.066 |
| 2996 | BERGENSTAL RM | 1 | 0.066 |
| 2997 | BERGER R | 1 | 0.066 |
| 2998 | BERGER Z | 1 | 0.066 |
| 2999 | BERKOWITZ SA | 1 | 0.066 |
| 3000 | BERLIN JA | 1 | 0.066 |
| 3001 | BERMUDEZ L | 1 | 0.066 |
| 3002 | BERNABE J | 1 | 0.066 |
| 3003 | BERNARDI S | 1 | 0.066 |
| 3004 | BERNARDO M | 1 | 0.066 |
| 3005 | BERNSTEIN R | 1 | 0.066 |
| 3006 | BERNSTEIN RA | 1 | 0.066 |
| 3007 | BERNSTEIN S | 1 | 0.066 |
| 3008 | BERRINO L | 1 | 0.066 |
| 3009 | BERTERO E | 1 | 0.066 |
| 3010 | BERTHOLD HK | 1 | 0.066 |
| 3011 | BERTOMEU-MARTINEZ V | 1 | 0.066 |
| 3012 | BERTRAND L | 1 | 0.066 |
| 3013 | BESSETTE LG | 1 | 0.066 |
| 3014 | BETTENCOURT P | 1 | 0.066 |
| 3015 | BEUSEKAMP JC | 1 | 0.066 |
| 3016 | BEYDOUN MF | 1 | 0.066 |
| 3017 | BGATOVA NP | 1 | 0.066 |
| 3018 | BHANSALI A | 1 | 0.066 |
| 3019 | BHARDWAJ A | 1 | 0.066 |
| 3020 | BHAT A | 1 | 0.066 |
| 3021 | BHATT SH | 1 | 0.066 |
| 3022 | BHUSHAN R | 1 | 0.066 |
| 3023 | BHUSHAN RS | 1 | 0.066 |
| 3024 | BIANCALANA E | 1 | 0.066 |
| 3025 | BIESTER T | 1 | 0.066 |
| 3026 | BILEZIKIAN JP | 1 | 0.066 |
| 3027 | BILIC-CURCIC I | 1 | 0.066 |
| 3028 | BILLOT L | 1 | 0.066 |
| 3029 | BIN HUSSAIN A | 1 | 0.066 |
| 3030 | BIOMATH D | 1 | 0.066 |
| 3031 | BIRBA D | 1 | 0.066 |
| 3032 | BIRKENFELD AL | 1 | 0.066 |
| 3033 | BIRNER C | 1 | 0.066 |
| 3034 | BIRTCHER KK | 1 | 0.066 |
| 3035 | BISCOVEANU M | 1 | 0.066 |
| 3036 | BISHAY RH | 1 | 0.066 |
| 3037 | BISWAS M | 1 | 0.066 |
| 3038 | BLACKBOURN LAK | 1 | 0.066 |
| 3039 | BLAIR RA | 1 | 0.066 |
| 3040 | BLAK BT | 1 | 0.066 |
| 3041 | BLAKE L | 1 | 0.066 |
| 3042 | BLANCHARD K | 1 | 0.066 |
| 3043 | BLANCO BMD | 1 | 0.066 |
| 3044 | BLANCO BMDC | 1 | 0.066 |
| 3045 | BLANCO PG | 1 | 0.066 |
| 3046 | BLEIJLEVENS B | 1 | 0.066 |
| 3047 | BLETSA E | 1 | 0.066 |
| 3048 | BLEVINS TC | 1 | 0.066 |
| 3049 | BLOMSTER J | 1 | 0.066 |
| 3050 | BLUMENTHAL RS | 1 | 0.066 |
| 3051 | BO S | 1 | 0.066 |
| 3052 | BOCCHI E | 1 | 0.066 |
| 3053 | BODE BW | 1 | 0.066 |
| 3054 | BODE C | 1 | 0.066 |
| 3055 | BOEDER S | 1 | 0.066 |
| 3056 | BOEHM M | 1 | 0.066 |
| 3057 | BOEMI M | 1 | 0.066 |
| 3058 | BOEMKE-ZELCH F | 1 | 0.066 |
| 3059 | BOERWINKLE E | 1 | 0.066 |
| 3060 | BOGAEV R | 1 | 0.066 |
| 3061 | BOGDANFFY MS | 1 | 0.066 |
| 3062 | BOHOVYK R | 1 | 0.066 |
| 3063 | BOJER AS | 1 | 0.066 |
| 3064 | BOLEN S | 1 | 0.066 |
| 3065 | BOLLENBERG H | 1 | 0.066 |
| 3066 | BOLLERSLEV J | 1 | 0.066 |
| 3067 | BOLTON W | 1 | 0.066 |
| 3068 | BONACA M | 1 | 0.066 |
| 3069 | BONAVENTURA A | 1 | 0.066 |
| 3070 | BONDUGULAPATI LNR | 1 | 0.066 |
| 3071 | BONILHA I | 1 | 0.066 |
| 3072 | BONNER C | 1 | 0.066 |
| 3073 | BONNES H | 1 | 0.066 |
| 3074 | BONNY O | 1 | 0.066 |
| 3075 | BOOBES Y | 1 | 0.066 |
| 3076 | BOORSMA EM | 1 | 0.066 |
| 3077 | BORG DJ | 1 | 0.066 |
| 3078 | BORGES FA | 1 | 0.066 |
| 3079 | BORGES JLC | 1 | 0.066 |
| 3080 | BORGHETTI G | 1 | 0.066 |
| 3081 | BORGHI C | 1 | 0.066 |
| 3082 | BORISSOVA AM | 1 | 0.066 |
| 3083 | BORNPOINT S | 1 | 0.066 |
| 3084 | BORRELLI S | 1 | 0.066 |
| 3085 | BOSSI AC | 1 | 0.066 |
| 3086 | BOTROS D | 1 | 0.066 |
| 3087 | BOTROS FT | 1 | 0.066 |
| 3088 | BOTTARI SP | 1 | 0.066 |
| 3089 | BOTTIGLIENGO D | 1 | 0.066 |
| 3090 | BOULOUKOU S | 1 | 0.066 |
| 3091 | BOULTON D | 1 | 0.066 |
| 3092 | BOURRET EM | 1 | 0.066 |
| 3093 | BOUTER KEC | 1 | 0.066 |
| 3094 | BOYD D | 1 | 0.066 |
| 3095 | BOYKO EJ | 1 | 0.066 |
| 3096 | BOZKURT B | 1 | 0.066 |
| 3097 | BOZOVIC A | 1 | 0.066 |
| 3098 | BRAAM B | 1 | 0.066 |
| 3099 | BRADY EM | 1 | 0.066 |
| 3100 | BRADY JA | 1 | 0.066 |
| 3101 | BRAHA A | 1 | 0.066 |
| 3102 | BRAMLAGE P | 1 | 0.066 |
| 3103 | BRANAGAN M | 1 | 0.066 |
| 3104 | BRAND T | 1 | 0.066 |
| 3105 | BRAND-ARZAMENDI K | 1 | 0.066 |
| 3106 | BRANDT NH | 1 | 0.066 |
| 3107 | BRANDT S | 1 | 0.066 |
| 3108 | BRANDTNER EM | 1 | 0.066 |
| 3109 | BREDER I | 1 | 0.066 |
| 3110 | BREDER JC | 1 | 0.066 |
| 3111 | BREITHAUPT-GROEGLER K | 1 | 0.066 |
| 3112 | BRELJAK D | 1 | 0.066 |
| 3113 | BRENNAN EP | 1 | 0.066 |
| 3114 | BRENNAN P | 1 | 0.066 |
| 3115 | BRESCIA F | 1 | 0.066 |
| 3116 | BRESSI E | 1 | 0.066 |
| 3117 | BREYER MD | 1 | 0.066 |
| 3118 | BRIDGEMAN MB | 1 | 0.066 |
| 3119 | BRIERLEY L | 1 | 0.066 |
| 3120 | BRIETZKE SA | 1 | 0.066 |
| 3121 | BRIFFA TG | 1 | 0.066 |
| 3122 | BRIGANTI EM | 1 | 0.066 |
| 3123 | BRIL F | 1 | 0.066 |
| 3124 | BRILL G | 1 | 0.066 |
| 3125 | BRINTON DL | 1 | 0.066 |
| 3126 | BRION M | 1 | 0.066 |
| 3127 | BRITO D | 1 | 0.066 |
| 3128 | BRITO JP | 1 | 0.066 |
| 3129 | BRITO-SANFIEL M | 1 | 0.066 |
| 3130 | BRIZZI MF | 1 | 0.066 |
| 3131 | BROADBENT DA | 1 | 0.066 |
| 3132 | BROCHU B | 1 | 0.066 |
| 3133 | BRODOVICZ KG | 1 | 0.066 |
| 3134 | BROMAGE DI | 1 | 0.066 |
| 3135 | BROOKS LK | 1 | 0.066 |
| 3136 | BROWN AJM | 1 | 0.066 |
| 3137 | BROWN E | 1 | 0.066 |
| 3138 | BROWN JM | 1 | 0.066 |
| 3139 | BROWN KE | 1 | 0.066 |
| 3140 | BROWN R | 1 | 0.066 |
| 3141 | BROWN SM | 1 | 0.066 |
| 3142 | BROX J | 1 | 0.066 |
| 3143 | BRUCE KE | 1 | 0.066 |
| 3144 | BRUCE S | 1 | 0.066 |
| 3145 | BRUCKERT C | 1 | 0.066 |
| 3146 | BRUDER O | 1 | 0.066 |
| 3147 | BRUN M | 1 | 0.066 |
| 3148 | BRUNE M | 1 | 0.066 |
| 3149 | BRUNTON SA | 1 | 0.066 |
| 3150 | BRUUN NE | 1 | 0.066 |
| 3151 | BUATOIS EM | 1 | 0.066 |
| 3152 | BUCH A | 1 | 0.066 |
| 3153 | BUCHEIT JD | 1 | 0.066 |
| 3154 | BUCHHOLTZ N | 1 | 0.066 |
| 3155 | BUDAJ A | 1 | 0.066 |
| 3156 | BUDOFF MJ | 1 | 0.066 |
| 3157 | BUKOWY JD | 1 | 0.066 |
| 3158 | BULBUL MC | 1 | 0.066 |
| 3159 | BULJ N | 1 | 0.066 |
| 3160 | BUMDELGER B | 1 | 0.066 |
| 3161 | BUNN RC | 1 | 0.066 |
| 3162 | BURDETTE JE | 1 | 0.066 |
| 3163 | BURGER C | 1 | 0.066 |
| 3164 | BURGESS D | 1 | 0.066 |
| 3165 | BURGGRAAF B | 1 | 0.066 |
| 3166 | BURGUERA B | 1 | 0.066 |
| 3167 | BURKE SL | 1 | 0.066 |
| 3168 | BURNSTOCK G | 1 | 0.066 |
| 3169 | BUSCH R | 1 | 0.066 |
| 3170 | BUSCH RS | 1 | 0.066 |
| 3171 | BUSETTO L | 1 | 0.066 |
| 3172 | BUTKOW N | 1 | 0.066 |
| 3173 | BUTLER MS | 1 | 0.066 |
| 3174 | BUYSMAN EK | 1 | 0.066 |
| 3175 | BYRNE NJ | 1 | 0.066 |
| 3176 | CABEZAS MC | 1 | 0.066 |
| 3177 | CADENA D | 1 | 0.066 |
| 3178 | CAI J | 1 | 0.066 |
| 3179 | CAI T | 1 | 0.066 |
| 3180 | CAI ZJ | 1 | 0.066 |
| 3181 | CAIAZZO R | 1 | 0.066 |
| 3182 | CALLE RA | 1 | 0.066 |
| 3183 | CALOGERO E | 1 | 0.066 |
| 3184 | CAMACHO F | 1 | 0.066 |
| 3185 | CAMPBELL IW | 1 | 0.066 |
| 3186 | CAMPBELL S | 1 | 0.066 |
| 3187 | CAMPEAU MA | 1 | 0.066 |
| 3188 | CAMPOS-TOIMIL M | 1 | 0.066 |
| 3189 | CANAAN Y | 1 | 0.066 |
| 3190 | CANDIDO R | 1 | 0.066 |
| 3191 | CANIVELL S | 1 | 0.066 |
| 3192 | CANNISTRACI R | 1 | 0.066 |
| 3193 | CANOVATCHEL W | 1 | 0.066 |
| 3194 | CANTINI G | 1 | 0.066 |
| 3195 | CAPARROTTA TM | 1 | 0.066 |
| 3196 | CAPOBIANCO G | 1 | 0.066 |
| 3197 | CAPPETTA D | 1 | 0.066 |
| 3198 | CAPUANO A | 1 | 0.066 |
| 3199 | CARAMELLI B | 1 | 0.066 |
| 3200 | CARBALLO D | 1 | 0.066 |
| 3201 | CARBALLO S | 1 | 0.066 |
| 3202 | CARBONE A | 1 | 0.066 |
| 3203 | CARFORA G | 1 | 0.066 |
| 3204 | CARNOVALE A | 1 | 0.066 |
| 3205 | CARPINO PA | 1 | 0.066 |
| 3206 | CARRANZA-NAVAL MJ | 1 | 0.066 |
| 3207 | CARRION C | 1 | 0.066 |
| 3208 | CARSON PE | 1 | 0.066 |
| 3209 | CARTER R | 1 | 0.066 |
| 3210 | CARUBBI C | 1 | 0.066 |
| 3211 | CARVALHO D | 1 | 0.066 |
| 3212 | CAS AD | 1 | 0.066 |
| 3213 | CASELLA R | 1 | 0.066 |
| 3214 | CASELLINI C | 1 | 0.066 |
| 3215 | CASIRAGHI C | 1 | 0.066 |
| 3216 | CASS A | 1 | 0.066 |
| 3217 | CASSARLY C | 1 | 0.066 |
| 3218 | CASTANARES-ZAPATERO D | 1 | 0.066 |
| 3219 | CASTANEDA VB | 1 | 0.066 |
| 3220 | CASTELLANA M | 1 | 0.066 |
| 3221 | CASTRO RD | 1 | 0.066 |
| 3222 | CASTRO-ACOSTA ML | 1 | 0.066 |
| 3223 | CATALIOTTI A | 1 | 0.066 |
| 3224 | CATARIG AM | 1 | 0.066 |
| 3225 | CATTADORI G | 1 | 0.066 |
| 3226 | CAVAIOLA TS | 1 | 0.066 |
| 3227 | CAVALCANTE P | 1 | 0.066 |
| 3228 | CAVALCANTI CAJ | 1 | 0.066 |
| 3229 | CAVALOT F | 1 | 0.066 |
| 3230 | CAZZANIGA A | 1 | 0.066 |
| 3231 | CAZZETTA G | 1 | 0.066 |
| 3232 | CEA-SORIANO L | 1 | 0.066 |
| 3233 | CEBADA AB | 1 | 0.066 |
| 3234 | CECCARELLI E | 1 | 0.066 |
| 3235 | CEJVANOVIC V | 1 | 0.066 |
| 3236 | CELI FS | 1 | 0.066 |
| 3237 | CERNEA S | 1 | 0.066 |
| 3238 | CERVONE S | 1 | 0.066 |
| 3239 | CESCUTTI J | 1 | 0.066 |
| 3240 | CEYLAN AF | 1 | 0.066 |
| 3241 | CHA BS | 1 | 0.066 |
| 3242 | CHA SA | 1 | 0.066 |
| 3243 | CHAABAN A | 1 | 0.066 |
| 3244 | CHAI SB | 1 | 0.066 |
| 3245 | CHAIKLEDKAEW U | 1 | 0.066 |
| 3246 | CHAITOFF A | 1 | 0.066 |
| 3247 | CHAMBERLAIN D | 1 | 0.066 |
| 3248 | CHAMBERLIN KW | 1 | 0.066 |
| 3249 | CHAMBERS ES | 1 | 0.066 |
| 3250 | CHAN CTM | 1 | 0.066 |
| 3251 | CHAN KW | 1 | 0.066 |
| 3252 | CHAN N | 1 | 0.066 |
| 3253 | CHAN TM | 1 | 0.066 |
| 3254 | CHAN WX | 1 | 0.066 |
| 3255 | CHANDRAKUMAR D | 1 | 0.066 |
| 3256 | CHANG A | 1 | 0.066 |
| 3257 | CHANG CH | 1 | 0.066 |
| 3258 | CHANG CY | 1 | 0.066 |
| 3259 | CHANG HC | 1 | 0.066 |
| 3260 | CHANG HW | 1 | 0.066 |
| 3261 | CHANG HY | 1 | 0.066 |
| 3262 | CHANG J | 1 | 0.066 |
| 3263 | CHANG LS | 1 | 0.066 |
| 3264 | CHANG NC | 1 | 0.066 |
| 3265 | CHANG T | 1 | 0.066 |
| 3266 | CHANG TI | 1 | 0.066 |
| 3267 | CHANG TJ | 1 | 0.066 |
| 3268 | CHANG YP | 1 | 0.066 |
| 3269 | CHAO TF | 1 | 0.066 |
| 3270 | CHAPIN L | 1 | 0.066 |
| 3271 | CHARALAMBOUS G | 1 | 0.066 |
| 3272 | CHARPENTIER F | 1 | 0.066 |
| 3273 | CHATSUDTHIPONG V | 1 | 0.066 |
| 3274 | CHATTIPAKORN S | 1 | 0.066 |
| 3275 | CHAU A | 1 | 0.066 |
| 3276 | CHAUDHARI S | 1 | 0.066 |
| 3277 | CHAUDHRY S | 1 | 0.066 |
| 3278 | CHAUDHURI A | 1 | 0.066 |
| 3279 | CHAUHAN K | 1 | 0.066 |
| 3280 | CHAVES FRP | 1 | 0.066 |
| 3281 | CHAWLA H | 1 | 0.066 |
| 3282 | CHAWLA M | 1 | 0.066 |
| 3283 | CHEEMA AN | 1 | 0.066 |
| 3284 | CHEEVER E | 1 | 0.066 |
| 3285 | CHEIFETZ A | 1 | 0.066 |
| 3286 | CHEN BC | 1 | 0.066 |
| 3287 | CHEN CX | 1 | 0.066 |
| 3288 | CHEN E | 1 | 0.066 |
| 3289 | CHEN HC | 1 | 0.066 |
| 3290 | CHEN HH | 1 | 0.066 |
| 3291 | CHEN KC | 1 | 0.066 |
| 3292 | CHEN KH | 1 | 0.066 |
| 3293 | CHEN Q | 1 | 0.066 |
| 3294 | CHEN SB | 1 | 0.066 |
| 3295 | CHEN WY | 1 | 0.066 |
| 3296 | CHEN YC | 1 | 0.066 |
| 3297 | CHEN YD | 1 | 0.066 |
| 3298 | CHEN YF | 1 | 0.066 |
| 3299 | CHEN YH | 1 | 0.066 |
| 3300 | CHEN YJ | 1 | 0.066 |
| 3301 | CHEN YT | 1 | 0.066 |
| 3302 | CHEN YW | 1 | 0.066 |
| 3303 | CHEN YY | 1 | 0.066 |
| 3304 | CHENG A | 1 | 0.066 |
| 3305 | CHENG AYY | 1 | 0.066 |
| 3306 | CHENG G | 1 | 0.066 |
| 3307 | CHENG KK | 1 | 0.066 |
| 3308 | CHENG SS | 1 | 0.066 |
| 3309 | CHENG XL | 1 | 0.066 |
| 3310 | CHERTOW G | 1 | 0.066 |
| 3311 | CHEUNG A | 1 | 0.066 |
| 3312 | CHEUNG BMY | 1 | 0.066 |
| 3313 | CHEWCHARAT A | 1 | 0.066 |
| 3314 | CHI C | 1 | 0.066 |
| 3315 | CHIABRANDO JG | 1 | 0.066 |
| 3316 | CHIANETTA R | 1 | 0.066 |
| 3317 | CHIANG HP | 1 | 0.066 |
| 3318 | CHIAZZA F | 1 | 0.066 |
| 3319 | CHIBA K | 1 | 0.066 |
| 3320 | CHIEN FJ | 1 | 0.066 |
| 3321 | CHIEN RN | 1 | 0.066 |
| 3322 | CHILELLI NC | 1 | 0.066 |
| 3323 | CHILITO A | 1 | 0.066 |
| 3324 | CHIMENE-WEISS J | 1 | 0.066 |
| 3325 | CHIN RN | 1 | 0.066 |
| 3326 | CHIN-DUSTING J | 1 | 0.066 |
| 3327 | CHIU YW | 1 | 0.066 |
| 3328 | CHO GY | 1 | 0.066 |
| 3329 | CHO H | 1 | 0.066 |
| 3330 | CHO HM | 1 | 0.066 |
| 3331 | CHO JH | 1 | 0.066 |
| 3332 | CHO JW | 1 | 0.066 |
| 3333 | CHO L | 1 | 0.066 |
| 3334 | CHO S | 1 | 0.066 |
| 3335 | CHO W | 1 | 0.066 |
| 3336 | CHO YE | 1 | 0.066 |
| 3337 | CHODICK G | 1 | 0.066 |
| 3338 | CHOI CH | 1 | 0.066 |
| 3339 | CHOI CI | 1 | 0.066 |
| 3340 | CHOI DH | 1 | 0.066 |
| 3341 | CHOI DJ | 1 | 0.066 |
| 3342 | CHOI E | 1 | 0.066 |
| 3343 | CHOI M | 1 | 0.066 |
| 3344 | CHOI S | 1 | 0.066 |
| 3345 | CHOI SE | 1 | 0.066 |
| 3346 | CHOKSI RR | 1 | 0.066 |
| 3347 | CHON S | 1 | 0.066 |
| 3348 | CHONG CR | 1 | 0.066 |
| 3349 | CHONG E | 1 | 0.066 |
| 3350 | CHOPRA V | 1 | 0.066 |
| 3351 | CHOUTHE RS | 1 | 0.066 |
| 3352 | CHOW CC | 1 | 0.066 |
| 3353 | CHOW WS | 1 | 0.066 |
| 3354 | CHOWDHURY B | 1 | 0.066 |
| 3355 | CHOWDHURY TA | 1 | 0.066 |
| 3356 | CHOY AM | 1 | 0.066 |
| 3357 | CHOY AMJ | 1 | 0.066 |
| 3358 | CHRISTENSEN N | 1 | 0.066 |
| 3359 | CHRISTIANO C | 1 | 0.066 |
| 3360 | CHRISTIANSEN AV | 1 | 0.066 |
| 3361 | CHRISTOFIDES E | 1 | 0.066 |
| 3362 | CHRISTOPHER S | 1 | 0.066 |
| 3363 | CHRISTOU K | 1 | 0.066 |
| 3364 | CHRYSANT SG | 1 | 0.066 |
| 3365 | CHU C | 1 | 0.066 |
| 3366 | CHU JX | 1 | 0.066 |
| 3367 | CHU LL | 1 | 0.066 |
| 3368 | CHU PL | 1 | 0.066 |
| 3369 | CHU S | 1 | 0.066 |
| 3370 | CHU Y | 1 | 0.066 |
| 3371 | CHUATECO C | 1 | 0.066 |
| 3372 | CHUDLEIGH RA | 1 | 0.066 |
| 3373 | CHUEAKULA N | 1 | 0.066 |
| 3374 | CHUNG CC | 1 | 0.066 |
| 3375 | CHUNG LT | 1 | 0.066 |
| 3376 | CHUNG S | 1 | 0.066 |
| 3377 | CHUNG SH | 1 | 0.066 |
| 3378 | CHUNG YR | 1 | 0.066 |
| 3379 | CHUQUIURE E | 1 | 0.066 |
| 3380 | CIANFLONE E | 1 | 0.066 |
| 3381 | CIARDULLO S | 1 | 0.066 |
| 3382 | CID-RAZUFA J | 1 | 0.066 |
| 3383 | CIGNARELLI A | 1 | 0.066 |
| 3384 | CIGOLINI M | 1 | 0.066 |
| 3385 | CINTRA R | 1 | 0.066 |
| 3386 | CINTRA RM | 1 | 0.066 |
| 3387 | CINTRA RMR | 1 | 0.066 |
| 3388 | CIPRIANI A | 1 | 0.066 |
| 3389 | CIPU D | 1 | 0.066 |
| 3390 | CIRULE H | 1 | 0.066 |
| 3391 | CIUFFREDA LP | 1 | 0.066 |
| 3392 | CLAGGETT B | 1 | 0.066 |
| 3393 | CLAR C | 1 | 0.066 |
| 3394 | CLARK AL | 1 | 0.066 |
| 3395 | CLARKE G | 1 | 0.066 |
| 3396 | CLARKE K | 1 | 0.066 |
| 3397 | CLARKE SL | 1 | 0.066 |
| 3398 | CLAUSON P | 1 | 0.066 |
| 3399 | CLEGG A | 1 | 0.066 |
| 3400 | CLEMENS K | 1 | 0.066 |
| 3401 | CLIFTON PM | 1 | 0.066 |
| 3402 | COCA SG | 1 | 0.066 |
| 3403 | COCH RW | 1 | 0.066 |
| 3404 | COELHO OR | 1 | 0.066 |
| 3405 | COFFEE L | 1 | 0.066 |
| 3406 | COHEN G | 1 | 0.066 |
| 3407 | COHEN K | 1 | 0.066 |
| 3408 | COHEN M | 1 | 0.066 |
| 3409 | COHEN ND | 1 | 0.066 |
| 3410 | COHEN R | 1 | 0.066 |
| 3411 | COHEN S | 1 | 0.066 |
| 3412 | COHEN-SOLAL A | 1 | 0.066 |
| 3413 | COHEN-STEIN D | 1 | 0.066 |
| 3414 | COHNEY S | 1 | 0.066 |
| 3415 | COLAGIURI S | 1 | 0.066 |
| 3416 | COLBERG SR | 1 | 0.066 |
| 3417 | COLEMAN RL | 1 | 0.066 |
| 3418 | COLLADO A | 1 | 0.066 |
| 3419 | COLLINO M | 1 | 0.066 |
| 3420 | COLLINS MT | 1 | 0.066 |
| 3421 | COLLINS S | 1 | 0.066 |
| 3422 | COLOMBO G | 1 | 0.066 |
| 3423 | COLQUITT J | 1 | 0.066 |
| 3424 | COLUCCI VJ | 1 | 0.066 |
| 3425 | COMOGLIO RH | 1 | 0.066 |
| 3426 | COMPAGNIE S | 1 | 0.066 |
| 3427 | CONGET I | 1 | 0.066 |
| 3428 | CONNELL C | 1 | 0.066 |
| 3429 | CONNOLLY DL | 1 | 0.066 |
| 3430 | CONTE G | 1 | 0.066 |
| 3431 | CONTRERAS JP | 1 | 0.066 |
| 3432 | CONWAY EM | 1 | 0.066 |
| 3433 | COOK C | 1 | 0.066 |
| 3434 | COOKE R | 1 | 0.066 |
| 3435 | COOPER AJ | 1 | 0.066 |
| 3436 | COOPER MA | 1 | 0.066 |
| 3437 | COOPER S | 1 | 0.066 |
| 3438 | COPPENRATH VA | 1 | 0.066 |
| 3439 | COPUR S | 1 | 0.066 |
| 3440 | CORDERO A | 1 | 0.066 |
| 3441 | CORESH J | 1 | 0.066 |
| 3442 | CORNU C | 1 | 0.066 |
| 3443 | CORPE CP | 1 | 0.066 |
| 3444 | CORRELL CU | 1 | 0.066 |
| 3445 | CORTESE C | 1 | 0.066 |
| 3446 | COSTANZO MR | 1 | 0.066 |
| 3447 | COURT R | 1 | 0.066 |
| 3448 | COURVILLE AB | 1 | 0.066 |
| 3449 | COUSELO-SEIJAS M | 1 | 0.066 |
| 3450 | COVIC A | 1 | 0.066 |
| 3451 | COVINO J | 1 | 0.066 |
| 3452 | COWLEY AW | 1 | 0.066 |
| 3453 | COX JM | 1 | 0.066 |
| 3454 | COYNE D | 1 | 0.066 |
| 3455 | COZZOLINO A | 1 | 0.066 |
| 3456 | CRAWFORD M | 1 | 0.066 |
| 3457 | CROWLEY RK | 1 | 0.066 |
| 3458 | CRUZ JE | 1 | 0.066 |
| 3459 | CSOMOS K | 1 | 0.066 |
| 3460 | CUCHERAT M | 1 | 0.066 |
| 3461 | CUCINOTTA D | 1 | 0.066 |
| 3462 | CUI L | 1 | 0.066 |
| 3463 | CUI LJ | 1 | 0.066 |
| 3464 | CUI W | 1 | 0.066 |
| 3465 | CUI YT | 1 | 0.066 |
| 3466 | CULHAM MD | 1 | 0.066 |
| 3467 | CUMMINS E | 1 | 0.066 |
| 3468 | CUNHA BORGES JL | 1 | 0.066 |
| 3469 | CUNNINGTON RH | 1 | 0.066 |
| 3470 | CURRIE CJ | 1 | 0.066 |
| 3471 | CUSHMAN WC | 1 | 0.066 |
| 3472 | CUTLER DL | 1 | 0.066 |
| 3473 | CUTRIN JC | 1 | 0.066 |
| 3474 | CUTSHALL BT | 1 | 0.066 |
| 3475 | CZUPRYNIAK L | 1 | 0.066 |
| 3476 | D'AMORE C | 1 | 0.066 |
| 3477 | D'ANGELO F | 1 | 0.066 |
| 3478 | D'ANGELO P | 1 | 0.066 |
| 3479 | D'ANNA M | 1 | 0.066 |
| 3480 | D'ASTOUS M | 1 | 0.066 |
| 3481 | D'AURIA S | 1 | 0.066 |
| 3482 | D'SOUZA J | 1 | 0.066 |
| 3483 | DABOUL N | 1 | 0.066 |
| 3484 | DAGOGO-JACK S | 1 | 0.066 |
| 3485 | DAHAGAM C | 1 | 0.066 |
| 3486 | DAHLQVIST U | 1 | 0.066 |
| 3487 | DAI AZ | 1 | 0.066 |
| 3488 | DAI X | 1 | 0.066 |
| 3489 | DAI Y | 1 | 0.066 |
| 3490 | DAI ZK | 1 | 0.066 |
| 3491 | DAIDA H | 1 | 0.066 |
| 3492 | DAILEY G | 1 | 0.066 |
| 3493 | DAILEY GE | 1 | 0.066 |
| 3494 | DALAMA B | 1 | 0.066 |
| 3495 | DALAN R | 1 | 0.066 |
| 3496 | DALES J | 1 | 0.066 |
| 3497 | DALLAPELLEGRINA L | 1 | 0.066 |
| 3498 | DALLINGA-THIE GM | 1 | 0.066 |
| 3499 | DAMARAJU CV | 1 | 0.066 |
| 3500 | DAMBROVA M | 1 | 0.066 |
| 3501 | DAMMAN K | 1 | 0.066 |
| 3502 | DANG S | 1 | 0.066 |
| 3503 | DANIELE G | 1 | 0.066 |
| 3504 | DANSER AHJ | 1 | 0.066 |
| 3505 | DAOUDI M | 1 | 0.066 |
| 3506 | DAPHNIS EK | 1 | 0.066 |
| 3507 | DARDANO A | 1 | 0.066 |
| 3508 | DARMON D | 1 | 0.066 |
| 3509 | DARWESH AM | 1 | 0.066 |
| 3510 | DARWISH R | 1 | 0.066 |
| 3511 | DAS AK | 1 | 0.066 |
| 3512 | DAS R | 1 | 0.066 |
| 3513 | DAS SR | 1 | 0.066 |
| 3514 | DASARI D | 1 | 0.066 |
| 3515 | DASGUPTA K | 1 | 0.066 |
| 3516 | DASHKIN MV | 1 | 0.066 |
| 3517 | DASWANI A | 1 | 0.066 |
| 3518 | DAVE CV | 1 | 0.066 |
| 3519 | DAVID C | 1 | 0.066 |
| 3520 | DAVIDSON JA | 1 | 0.066 |
| 3521 | DAVIS D | 1 | 0.066 |
| 3522 | DAVIS M | 1 | 0.066 |
| 3523 | DAVOS CH | 1 | 0.066 |
| 3524 | DAY C | 1 | 0.066 |
| 3525 | DAY RO | 1 | 0.066 |
| 3526 | DE ANGELIS A | 1 | 0.066 |
| 3527 | DE BACKER G | 1 | 0.066 |
| 3528 | DE BACQUER D | 1 | 0.066 |
| 3529 | DE BLOCK C | 1 | 0.066 |
| 3530 | DE BOBADILLA JF | 1 | 0.066 |
| 3531 | DE BORST MH | 1 | 0.066 |
| 3532 | DE BROUWER K | 1 | 0.066 |
| 3533 | DE CARVALHO LSF | 1 | 0.066 |
| 3534 | DE GIUSTI CJ | 1 | 0.066 |
| 3535 | DE KREUTZENBERG SV | 1 | 0.066 |
| 3536 | DE LEEUW AE | 1 | 0.066 |
| 3537 | DE LIMA FJC | 1 | 0.066 |
| 3538 | DE LIMA JC | 1 | 0.066 |
| 3539 | DE MARANON AM | 1 | 0.066 |
| 3540 | DE MELO RT | 1 | 0.066 |
| 3541 | DE MESTRAL C | 1 | 0.066 |
| 3542 | DE MICHIELI F | 1 | 0.066 |
| 3543 | DE NIGRIS V | 1 | 0.066 |
| 3544 | DE PASQUALE CG | 1 | 0.066 |
| 3545 | DE ROSA S | 1 | 0.066 |
| 3546 | DE S | 1 | 0.066 |
| 3547 | DE SOUSA-RODRIGUES CF | 1 | 0.066 |
| 3548 | DE VOS LC | 1 | 0.066 |
| 3549 | DEA L | 1 | 0.066 |
| 3550 | DEAN Z | 1 | 0.066 |
| 3551 | DEAR AE | 1 | 0.066 |
| 3552 | DECK K | 1 | 0.066 |
| 3553 | DEED G | 1 | 0.066 |
| 3554 | DEFALCO F | 1 | 0.066 |
| 3555 | DEGIRMENCI S | 1 | 0.066 |
| 3556 | DEGUCHI R | 1 | 0.066 |
| 3557 | DEI CAS A | 1 | 0.066 |
| 3558 | DEL MARCO A | 1 | 0.066 |
| 3559 | DELGADO DH | 1 | 0.066 |
| 3560 | DELGADO E | 1 | 0.066 |
| 3561 | DELL M | 1 | 0.066 |
| 3562 | DELL'AVERSANA C | 1 | 0.066 |
| 3563 | DELL'AVERSANA S | 1 | 0.066 |
| 3564 | DEMAREST K | 1 | 0.066 |
| 3565 | DEMURA M | 1 | 0.066 |
| 3566 | DENAXAS S | 1 | 0.066 |
| 3567 | DENG AP | 1 | 0.066 |
| 3568 | DENG LM | 1 | 0.066 |
| 3569 | DERAZNE E | 1 | 0.066 |
| 3570 | DERUAZ-LUYET A | 1 | 0.066 |
| 3571 | DESAI A | 1 | 0.066 |
| 3572 | DESHMUKH H | 1 | 0.066 |
| 3573 | DESHPANDE M | 1 | 0.066 |
| 3574 | DESLAURIERS C | 1 | 0.066 |
| 3575 | DESOUZA CV | 1 | 0.066 |
| 3576 | DESPLANTIE O | 1 | 0.066 |
| 3577 | DESSY C | 1 | 0.066 |
| 3578 | DESTREE M | 1 | 0.066 |
| 3579 | DEV D | 1 | 0.066 |
| 3580 | DEVANGELIO E | 1 | 0.066 |
| 3581 | DEVINENI D | 1 | 0.066 |
| 3582 | DEVINS T | 1 | 0.066 |
| 3583 | DEWAN P | 1 | 0.066 |
| 3584 | DEY AK | 1 | 0.066 |
| 3585 | DEY J | 1 | 0.066 |
| 3586 | DHANDHANIA V | 1 | 0.066 |
| 3587 | DHAR A | 1 | 0.066 |
| 3588 | DHAR I | 1 | 0.066 |
| 3589 | DHARMALINGAM M | 1 | 0.066 |
| 3590 | DHATARIYA K | 1 | 0.066 |
| 3591 | DHILLON M | 1 | 0.066 |
| 3592 | DHILLON S | 1 | 0.066 |
| 3593 | DHINDSA DS | 1 | 0.066 |
| 3594 | DI BELLO V | 1 | 0.066 |
| 3595 | DI BENEDETTO A | 1 | 0.066 |
| 3596 | DI NAPOLI P | 1 | 0.066 |
| 3597 | DI TANNA GL | 1 | 0.066 |
| 3598 | DIACONU L | 1 | 0.066 |
| 3599 | DIAZ LJ | 1 | 0.066 |
| 3600 | DIAZ R | 1 | 0.066 |
| 3601 | DIAZ SA | 1 | 0.066 |
| 3602 | DIAZ-MEJIA N | 1 | 0.066 |
| 3603 | DIAZ-MORALES N | 1 | 0.066 |
| 3604 | DIAZ-RODRIGUEZ E | 1 | 0.066 |
| 3605 | DIENEMANN T | 1 | 0.066 |
| 3606 | DIENER HC | 1 | 0.066 |
| 3607 | DIETER B | 1 | 0.066 |
| 3608 | DIETZ A | 1 | 0.066 |
| 3609 | DIMA L | 1 | 0.066 |
| 3610 | DIMITRIADIS GD | 1 | 0.066 |
| 3611 | DIMITRIOU CA | 1 | 0.066 |
| 3612 | DIMITRIOU T | 1 | 0.066 |
| 3613 | DIMITROPOULOS G | 1 | 0.066 |
| 3614 | DIMOVA R | 1 | 0.066 |
| 3615 | DING FH | 1 | 0.066 |
| 3616 | DINKHA LR | 1 | 0.066 |
| 3617 | DIPP S | 1 | 0.066 |
| 3618 | DIVANI M | 1 | 0.066 |
| 3619 | DIWAN PV | 1 | 0.066 |
| 3620 | DOGGRELL SA | 1 | 0.066 |
| 3621 | DOLIBA NM | 1 | 0.066 |
| 3622 | DOLLET L | 1 | 0.066 |
| 3623 | DOMECQ JP | 1 | 0.066 |
| 3624 | DOMINGUEZ E | 1 | 0.066 |
| 3625 | DONG BZ | 1 | 0.066 |
| 3626 | DONG F | 1 | 0.066 |
| 3627 | DONG HJ | 1 | 0.066 |
| 3628 | DONG PS | 1 | 0.066 |
| 3629 | DONG TC | 1 | 0.066 |
| 3630 | DONNAN PT | 1 | 0.066 |
| 3631 | DONNELLY R | 1 | 0.066 |
| 3632 | DOS SANTOS DS | 1 | 0.066 |
| 3633 | DOUPIS J | 1 | 0.066 |
| 3634 | DRAGOLJEVIC D | 1 | 0.066 |
| 3635 | DRAZNER MH | 1 | 0.066 |
| 3636 | DREYFUSS JM | 1 | 0.066 |
| 3637 | DRISCOLL A | 1 | 0.066 |
| 3638 | DRONAMRAJU N | 1 | 0.066 |
| 3639 | DRUCKER DJ | 1 | 0.066 |
| 3640 | DU ZM | 1 | 0.066 |
| 3641 | DUA S | 1 | 0.066 |
| 3642 | DUARTE J | 1 | 0.066 |
| 3643 | DUCENA K | 1 | 0.066 |
| 3644 | DUCHARME A | 1 | 0.066 |
| 3645 | DUCHEIX S | 1 | 0.066 |
| 3646 | DUDUM R | 1 | 0.066 |
| 3647 | DUFF HJ | 1 | 0.066 |
| 3648 | DUFFY CI | 1 | 0.066 |
| 3649 | DUGI K | 1 | 0.066 |
| 3650 | DUMANN E | 1 | 0.066 |
| 3651 | DUNDAS JA | 1 | 0.066 |
| 3652 | DUNHAM MW | 1 | 0.066 |
| 3653 | DUNSKY A | 1 | 0.066 |
| 3654 | DUONG J | 1 | 0.066 |
| 3655 | DUPONT M | 1 | 0.066 |
| 3656 | DURAES AR | 1 | 0.066 |
| 3657 | DURAK A | 1 | 0.066 |
| 3658 | DURANTE C | 1 | 0.066 |
| 3659 | DURANTE GL | 1 | 0.066 |
| 3660 | DURANTE ZE | 1 | 0.066 |
| 3661 | DURATORRE E | 1 | 0.066 |
| 3662 | DURAZZO M | 1 | 0.066 |
| 3663 | DUTA C | 1 | 0.066 |
| 3664 | DUTKIEWICZ-PIASECKA M | 1 | 0.066 |
| 3665 | DUTTA D | 1 | 0.066 |
| 3666 | DUVNJAK LS | 1 | 0.066 |
| 3667 | DWIVEDI G | 1 | 0.066 |
| 3668 | DWORACKA M | 1 | 0.066 |
| 3669 | DWORACKI G | 1 | 0.066 |
| 3670 | DWORAK M | 1 | 0.066 |
| 3671 | DWYER JP | 1 | 0.066 |
| 3672 | DYAL L | 1 | 0.066 |
| 3673 | DYKHOFF HJ | 1 | 0.066 |
| 3674 | DZIUBA J | 1 | 0.066 |
| 3675 | EAPEN S | 1 | 0.066 |
| 3676 | EARL CC | 1 | 0.066 |
| 3677 | EATON DM | 1 | 0.066 |
| 3678 | EBELING T | 1 | 0.066 |
| 3679 | ECHOUFFO-TCHEUGUI JB | 1 | 0.066 |
| 3680 | ECKSTEIN N | 1 | 0.066 |
| 3681 | ECTON KE | 1 | 0.066 |
| 3682 | EDELMAN SV | 1 | 0.066 |
| 3683 | EDI R | 1 | 0.066 |
| 3684 | EDMONSTON D | 1 | 0.066 |
| 3685 | EERBEEK O | 1 | 0.066 |
| 3686 | EFENTAKIS P | 1 | 0.066 |
| 3687 | EGGER A | 1 | 0.066 |
| 3688 | EGUCHI Y | 1 | 0.066 |
| 3689 | EIAM-ONG S | 1 | 0.066 |
| 3690 | EID EA | 1 | 0.066 |
| 3691 | EIKELIS N | 1 | 0.066 |
| 3692 | EILBRACHT J | 1 | 0.066 |
| 3693 | EINHORN D | 1 | 0.066 |
| 3694 | EISEN HJ | 1 | 0.066 |
| 3695 | EISENGA MF | 1 | 0.066 |
| 3696 | EJIRI K | 1 | 0.066 |
| 3697 | EKART R | 1 | 0.066 |
| 3698 | EKELUND J | 1 | 0.066 |
| 3699 | EKMAN M | 1 | 0.066 |
| 3700 | EL ESSAWY B | 1 | 0.066 |
| 3701 | EL MOUHAYYAR C | 1 | 0.066 |
| 3702 | EL SAYED NA | 1 | 0.066 |
| 3703 | EL-DALY M | 1 | 0.066 |
| 3704 | EL-KADI AOS | 1 | 0.066 |
| 3705 | EL-SHAHAWY M | 1 | 0.066 |
| 3706 | EL-YAMANY MF | 1 | 0.066 |
| 3707 | EL-YAZBI A | 1 | 0.066 |
| 3708 | ELEBRASHY IN | 1 | 0.066 |
| 3709 | ELEFTHERIADIS T | 1 | 0.066 |
| 3710 | ELEFTHERIADOU A | 1 | 0.066 |
| 3711 | ELENKOVA A | 1 | 0.066 |
| 3712 | ELGENDY AY | 1 | 0.066 |
| 3713 | ELLIOT RH | 1 | 0.066 |
| 3714 | ELOSEGUI AM | 1 | 0.066 |
| 3715 | ELRAIYAH T | 1 | 0.066 |
| 3716 | ELSEAIDY T | 1 | 0.066 |
| 3717 | ELSHAZLI RM | 1 | 0.066 |
| 3718 | ELVAN A | 1 | 0.066 |
| 3719 | ENDO J | 1 | 0.066 |
| 3720 | ENHOFFER DM | 1 | 0.066 |
| 3721 | ERBEN RG | 1 | 0.066 |
| 3722 | EROR DB | 1 | 0.066 |
| 3723 | ERVIN J | 1 | 0.066 |
| 3724 | ERYTHROPOULOU-KALTSIDOU A | 1 | 0.066 |
| 3725 | ESCALADA J | 1 | 0.066 |
| 3726 | ESCOBAR-MORREALE HF | 1 | 0.066 |
| 3727 | ESPADERO RM | 1 | 0.066 |
| 3728 | ESPINEL E | 1 | 0.066 |
| 3729 | ESPINOSA E | 1 | 0.066 |
| 3730 | ESPOSITO L | 1 | 0.066 |
| 3731 | ESQUENAZI A | 1 | 0.066 |
| 3732 | ESQUIVEL MA | 1 | 0.066 |
| 3733 | ESSER N | 1 | 0.066 |
| 3734 | ESTERLINE RL | 1 | 0.066 |
| 3735 | ESTRELLA-HOLDER E | 1 | 0.066 |
| 3736 | EUDICONE JM | 1 | 0.066 |
| 3737 | EVANS J | 1 | 0.066 |
| 3738 | EWELL TR | 1 | 0.066 |
| 3739 | EZEKOWITZ J | 1 | 0.066 |
| 3740 | FABIANI I | 1 | 0.066 |
| 3741 | FADLO F | 1 | 0.066 |
| 3742 | FAGAN N | 1 | 0.066 |
| 3743 | FAGBOTE CO | 1 | 0.066 |
| 3744 | FAKHRE NA | 1 | 0.066 |
| 3745 | FAKIH I | 1 | 0.066 |
| 3746 | FALALYEYEVA T | 1 | 0.066 |
| 3747 | FALCON R | 1 | 0.066 |
| 3748 | FALLATAH SB | 1 | 0.066 |
| 3749 | FAMULLA S | 1 | 0.066 |
| 3750 | FANELLI F | 1 | 0.066 |
| 3751 | FANG Y | 1 | 0.066 |
| 3752 | FANG ZW | 1 | 0.066 |
| 3753 | FANTOZZI R | 1 | 0.066 |
| 3754 | FANTUZZI F | 1 | 0.066 |
| 3755 | FARAHANI P | 1 | 0.066 |
| 3756 | FARHAT A | 1 | 0.066 |
| 3757 | FARHOUMAND PD | 1 | 0.066 |
| 3758 | FARKOUH M | 1 | 0.066 |
| 3759 | FAROOKI A | 1 | 0.066 |
| 3760 | FAROOQ MA | 1 | 0.066 |
| 3761 | FASELIS C | 1 | 0.066 |
| 3762 | FATIMA S | 1 | 0.066 |
| 3763 | FATTAH H | 1 | 0.066 |
| 3764 | FATTOR B | 1 | 0.066 |
| 3765 | FEBBRAIO MA | 1 | 0.066 |
| 3766 | FEBO F | 1 | 0.066 |
| 3767 | FEDERICI M | 1 | 0.066 |
| 3768 | FEDORIUK M | 1 | 0.066 |
| 3769 | FEHER MD | 1 | 0.066 |
| 3770 | FEI Y | 1 | 0.066 |
| 3771 | FEIJOO-BANDIN S | 1 | 0.066 |
| 3772 | FELACE G | 1 | 0.066 |
| 3773 | FELKER GM | 1 | 0.066 |
| 3774 | FENG AN | 1 | 0.066 |
| 3775 | FENG B | 1 | 0.066 |
| 3776 | FENG X | 1 | 0.066 |
| 3777 | FENG YM | 1 | 0.066 |
| 3778 | FEOFANOVA E | 1 | 0.066 |
| 3779 | FERDAOUSSI M | 1 | 0.066 |
| 3780 | FERGUS I | 1 | 0.066 |
| 3781 | FERKET B | 1 | 0.066 |
| 3782 | FERNANDES VHR | 1 | 0.066 |
| 3783 | FERNANDEZ-GARCIA JC | 1 | 0.066 |
| 3784 | FERNANDEZ-PRADO R | 1 | 0.066 |
| 3785 | FERRANDINO R | 1 | 0.066 |
| 3786 | FERRARO RA | 1 | 0.066 |
| 3787 | FERRAZZANO F | 1 | 0.066 |
| 3788 | FERREIRA J | 1 | 0.066 |
| 3789 | FERREIRA JP | 1 | 0.066 |
| 3790 | FERREIRA-HERMOSILLO A | 1 | 0.066 |
| 3791 | FERRO CJ | 1 | 0.066 |
| 3792 | FEUERSTEIN J | 1 | 0.066 |
| 3793 | FIGTREE G | 1 | 0.066 |
| 3794 | FIGTREE GA | 1 | 0.066 |
| 3795 | FILETTI S | 1 | 0.066 |
| 3796 | FINE N | 1 | 0.066 |
| 3797 | FINK JC | 1 | 0.066 |
| 3798 | FINUCANE F | 1 | 0.066 |
| 3799 | FIORENTINO R | 1 | 0.066 |
| 3800 | FIROZ CK | 1 | 0.066 |
| 3801 | FISCHER T | 1 | 0.066 |
| 3802 | FISCHL AH | 1 | 0.066 |
| 3803 | FISHBANE S | 1 | 0.066 |
| 3804 | FISHER EA | 1 | 0.066 |
| 3805 | FISHMAN B | 1 | 0.066 |
| 3806 | FISMAN EZ | 1 | 0.066 |
| 3807 | FITRIDGE R | 1 | 0.066 |
| 3808 | FLEMING J | 1 | 0.066 |
| 3809 | FLEMMING NB | 1 | 0.066 |
| 3810 | FLOYD JS | 1 | 0.066 |
| 3811 | FLYNN M | 1 | 0.066 |
| 3812 | FONG MC | 1 | 0.066 |
| 3813 | FONG MW | 1 | 0.066 |
| 3814 | FONGTROW GC | 1 | 0.066 |
| 3815 | FONSECA C | 1 | 0.066 |
| 3816 | FONSECA VA | 1 | 0.066 |
| 3817 | FONTES ML | 1 | 0.066 |
| 3818 | FOOS V | 1 | 0.066 |
| 3819 | FORCIEA MA | 1 | 0.066 |
| 3820 | FOREST CP | 1 | 0.066 |
| 3821 | FORESTO RD | 1 | 0.066 |
| 3822 | FOSBOL E | 1 | 0.066 |
| 3823 | FOTHERINGHAM AK | 1 | 0.066 |
| 3824 | FOUQUE D | 1 | 0.066 |
| 3825 | FOUSHEE JA | 1 | 0.066 |
| 3826 | FOWLKES JL | 1 | 0.066 |
| 3827 | FOX R | 1 | 0.066 |
| 3828 | FOX RD | 1 | 0.066 |
| 3829 | FRAMPTON JE | 1 | 0.066 |
| 3830 | FRANCESCHINI N | 1 | 0.066 |
| 3831 | FRANCH-NADAL J | 1 | 0.066 |
| 3832 | FRANCIS D | 1 | 0.066 |
| 3833 | FRANCO F | 1 | 0.066 |
| 3834 | FRANKENSTEIN L | 1 | 0.066 |
| 3835 | FRANKLIN JM | 1 | 0.066 |
| 3836 | FRANTZ S | 1 | 0.066 |
| 3837 | FRANZETTI I | 1 | 0.066 |
| 3838 | FRAUNBERGER P | 1 | 0.066 |
| 3839 | FRAZIER KS | 1 | 0.066 |
| 3840 | FRAZZETTO M | 1 | 0.066 |
| 3841 | FREDERICH R | 1 | 0.066 |
| 3842 | FREDERIKSEN PH | 1 | 0.066 |
| 3843 | FREEMAN MW | 1 | 0.066 |
| 3844 | FREIMARK D | 1 | 0.066 |
| 3845 | FRENIS K | 1 | 0.066 |
| 3846 | FRIAS J | 1 | 0.066 |
| 3847 | FRISHMAN WH | 1 | 0.066 |
| 3848 | FRISON V | 1 | 0.066 |
| 3849 | FRITTITTA L | 1 | 0.066 |
| 3850 | FROHLICH H | 1 | 0.066 |
| 3851 | FROST G | 1 | 0.066 |
| 3852 | FRYDRYCHOWICZ M | 1 | 0.066 |
| 3853 | FRYER AA | 1 | 0.066 |
| 3854 | FU Y | 1 | 0.066 |
| 3855 | FUJIGAKI Y | 1 | 0.066 |
| 3856 | FUJIHARA Y | 1 | 0.066 |
| 3857 | FUJII H | 1 | 0.066 |
| 3858 | FUJII K | 1 | 0.066 |
| 3859 | FUJII S | 1 | 0.066 |
| 3860 | FUJII W | 1 | 0.066 |
| 3861 | FUJIMIYA M | 1 | 0.066 |
| 3862 | FUJIMOTO A | 1 | 0.066 |
| 3863 | FUJIMURA Y | 1 | 0.066 |
| 3864 | FUJITA S | 1 | 0.066 |
| 3865 | FUJITA T | 1 | 0.066 |
| 3866 | FUJITA Y | 1 | 0.066 |
| 3867 | FUJITANI Y | 1 | 0.066 |
| 3868 | FUJIWARA T | 1 | 0.066 |
| 3869 | FUKUDA Y | 1 | 0.066 |
| 3870 | FUKUI T | 1 | 0.066 |
| 3871 | FUKUMOTO Y | 1 | 0.066 |
| 3872 | FUKUSHIMA A | 1 | 0.066 |
| 3873 | FUNAZAKI S | 1 | 0.066 |
| 3874 | FUNER L | 1 | 0.066 |
| 3875 | FURIHATA T | 1 | 0.066 |
| 3876 | FURLER J | 1 | 0.066 |
| 3877 | FUSE K | 1 | 0.066 |
| 3878 | FUSHIMI N | 1 | 0.066 |
| 3879 | FUSHIMI Y | 1 | 0.066 |
| 3880 | FUTATA T | 1 | 0.066 |
| 3881 | GADSBY R | 1 | 0.066 |
| 3882 | GAEDE PH | 1 | 0.066 |
| 3883 | GAERTNER S | 1 | 0.066 |
| 3884 | GAGLIA J | 1 | 0.066 |
| 3885 | GAIKWAD R | 1 | 0.066 |
| 3886 | GAJOS G | 1 | 0.066 |
| 3887 | GALE SE | 1 | 0.066 |
| 3888 | GALINDO-RAMOS E | 1 | 0.066 |
| 3889 | GALITZ M | 1 | 0.066 |
| 3890 | GALLI M | 1 | 0.066 |
| 3891 | GALLWITZ B | 1 | 0.066 |
| 3892 | GALPHIN C | 1 | 0.066 |
| 3893 | GALVE E | 1 | 0.066 |
| 3894 | GAMBINO D | 1 | 0.066 |
| 3895 | GAN LM | 1 | 0.066 |
| 3896 | GANBAATAR B | 1 | 0.066 |
| 3897 | GANDA OP | 1 | 0.066 |
| 3898 | GANDHI P | 1 | 0.066 |
| 3899 | GANDY S | 1 | 0.066 |
| 3900 | GANGOPADHYAY KK | 1 | 0.066 |
| 3901 | GANGULY R | 1 | 0.066 |
| 3902 | GANOTAKIS ES | 1 | 0.066 |
| 3903 | GANTZ I | 1 | 0.066 |
| 3904 | GAO G | 1 | 0.066 |
| 3905 | GAO HK | 1 | 0.066 |
| 3906 | GAO P | 1 | 0.066 |
| 3907 | GARAL-PANTALER E | 1 | 0.066 |
| 3908 | GARCEAU C | 1 | 0.066 |
| 3909 | GARCIA-ALLOZA M | 1 | 0.066 |
| 3910 | GARCIA-CARRO C | 1 | 0.066 |
| 3911 | GARCIA-PORRERO E | 1 | 0.066 |
| 3912 | GARCIA-SANCHEZ R | 1 | 0.066 |
| 3913 | GARCIA-SECO F | 1 | 0.066 |
| 3914 | GARCIA-SECO JA | 1 | 0.066 |
| 3915 | GARG M | 1 | 0.066 |
| 3916 | GARG SK | 1 | 0.066 |
| 3917 | GARGIULO G | 1 | 0.066 |
| 3918 | GARIN N | 1 | 0.066 |
| 3919 | GARNICA P | 1 | 0.066 |
| 3920 | GARRETT K | 1 | 0.066 |
| 3921 | GARRIDO-MENDOZA AP | 1 | 0.066 |
| 3922 | GARRO M | 1 | 0.066 |
| 3923 | GARRY EM | 1 | 0.066 |
| 3924 | GARVEY WT | 1 | 0.066 |
| 3925 | GASPARI T | 1 | 0.066 |
| 3926 | GATTI A | 1 | 0.066 |
| 3927 | GAUGHRAN F | 1 | 0.066 |
| 3928 | GAUSE-NILSON IAM | 1 | 0.066 |
| 3929 | GAUTAM S | 1 | 0.066 |
| 3930 | GE J | 1 | 0.066 |
| 3931 | GE JB | 1 | 0.066 |
| 3932 | GEBHARD C | 1 | 0.066 |
| 3933 | GEE HY | 1 | 0.066 |
| 3934 | GEIGER K | 1 | 0.066 |
| 3935 | GEILLINGER KE | 1 | 0.066 |
| 3936 | GEISLER JG | 1 | 0.066 |
| 3937 | GELADARI E | 1 | 0.066 |
| 3938 | GENTILE CL | 1 | 0.066 |
| 3939 | GENUARDI MV | 1 | 0.066 |
| 3940 | GEORGE P | 1 | 0.066 |
| 3941 | GEORGE RE | 1 | 0.066 |
| 3942 | GEORGE S | 1 | 0.066 |
| 3943 | GEORGIADO E | 1 | 0.066 |
| 3944 | GEORGIJEV N | 1 | 0.066 |
| 3945 | GEORGIOPOULOS G | 1 | 0.066 |
| 3946 | GERASIMIDIS K | 1 | 0.066 |
| 3947 | GEROU S | 1 | 0.066 |
| 3948 | GERSTEIN HC | 1 | 0.066 |
| 3949 | GERSZTEN R | 1 | 0.066 |
| 3950 | GHAZAL N | 1 | 0.066 |
| 3951 | GHEZZI C | 1 | 0.066 |
| 3952 | GHONEIM HA | 1 | 0.066 |
| 3953 | GHOSH A | 1 | 0.066 |
| 3954 | GHOSH GC | 1 | 0.066 |
| 3955 | GHOSH S | 1 | 0.066 |
| 3956 | GHOSH-SWABY OR | 1 | 0.066 |
| 3957 | GIANDALIA A | 1 | 0.066 |
| 3958 | GIANI MGP | 1 | 0.066 |
| 3959 | GICCHINO M | 1 | 0.066 |
| 3960 | GIFFORD L | 1 | 0.066 |
| 3961 | GIL-LONGO J | 1 | 0.066 |
| 3962 | GILBERT JD | 1 | 0.066 |
| 3963 | GILON P | 1 | 0.066 |
| 3964 | GILSTRAP LG | 1 | 0.066 |
| 3965 | GIORDANO C | 1 | 0.066 |
| 3966 | GIORDANO M | 1 | 0.066 |
| 3967 | GIRARDI ACC | 1 | 0.066 |
| 3968 | GIRERD S | 1 | 0.066 |
| 3969 | GISSLER MC | 1 | 0.066 |
| 3970 | GIULIANO F | 1 | 0.066 |
| 3971 | GIVERTZ MM | 1 | 0.066 |
| 3972 | GKEKAS NK | 1 | 0.066 |
| 3973 | GLAZER SA | 1 | 0.066 |
| 3974 | GLYKOFRIDI S | 1 | 0.066 |
| 3975 | GLYNN RJ | 1 | 0.066 |
| 3976 | GNASSO C | 1 | 0.066 |
| 3977 | GNESIN F | 1 | 0.066 |
| 3978 | GOBBI G | 1 | 0.066 |
| 3979 | GODA M | 1 | 0.066 |
| 3980 | GODEC T | 1 | 0.066 |
| 3981 | GODEC TR | 1 | 0.066 |
| 3982 | GODLEY PJ | 1 | 0.066 |
| 3983 | GODTEL-ARMBRUST U | 1 | 0.066 |
| 3984 | GODWIN EC | 1 | 0.066 |
| 3985 | GOH SY | 1 | 0.066 |
| 3986 | GOKHALE K | 1 | 0.066 |
| 3987 | GOKOSMANOGLU F | 1 | 0.066 |
| 3988 | GOLDBERG M | 1 | 0.066 |
| 3989 | GOLDFADEN RF | 1 | 0.066 |
| 3990 | GOLDFINE AB | 1 | 0.066 |
| 3991 | GOLDMAN B | 1 | 0.066 |
| 3992 | GOLDMAN-LEVINE JD | 1 | 0.066 |
| 3993 | GOLDSMITH M | 1 | 0.066 |
| 3994 | GOLM G | 1 | 0.066 |
| 3995 | GOLZAR Y | 1 | 0.066 |
| 3996 | GOMES MB | 1 | 0.066 |
| 3997 | GOMIS R | 1 | 0.066 |
| 3998 | GONCALVES-TEIXEIRA P | 1 | 0.066 |
| 3999 | GONG MQ | 1 | 0.066 |
| 4000 | GONG SQ | 1 | 0.066 |
| 4001 | GONZALEZ DE | 1 | 0.066 |
| 4002 | GONZALEZ E | 1 | 0.066 |
| 4003 | GONZALEZ JPM | 1 | 0.066 |
| 4004 | GONZALEZ-IZQUIERDO A | 1 | 0.066 |
| 4005 | GONZALEZ-NAVARRO H | 1 | 0.066 |
| 4006 | GONZALVO JD | 1 | 0.066 |
| 4007 | GOODBAR NH | 1 | 0.066 |
| 4008 | GOODWILL AG | 1 | 0.066 |
| 4009 | GOPEL SO | 1 | 0.066 |
| 4010 | GORDON J | 1 | 0.066 |
| 4011 | GORDON RA | 1 | 0.066 |
| 4012 | GORELICK P | 1 | 0.066 |
| 4013 | GORGOJO-MARTINEZ JJ | 1 | 0.066 |
| 4014 | GORMSEN LC | 1 | 0.066 |
| 4015 | GORNY D | 1 | 0.066 |
| 4016 | GORSON D | 1 | 0.066 |
| 4017 | GOSWAMI D | 1 | 0.066 |
| 4018 | GOTFRIED R | 1 | 0.066 |
| 4019 | GOTO S | 1 | 0.066 |
| 4020 | GOUD A | 1 | 0.066 |
| 4021 | GOULIS DG | 1 | 0.066 |
| 4022 | GOUNI-BERTHOLD I | 1 | 0.066 |
| 4023 | GOURGARI E | 1 | 0.066 |
| 4024 | GOURZOULIDIS G | 1 | 0.066 |
| 4025 | GOWDA A | 1 | 0.066 |
| 4026 | GOWER EW | 1 | 0.066 |
| 4027 | GOYAL R | 1 | 0.066 |
| 4028 | GOYAL S | 1 | 0.066 |
| 4029 | GOYAL SN | 1 | 0.066 |
| 4030 | GOYAT R | 1 | 0.066 |
| 4031 | GOYKHMAN S | 1 | 0.066 |
| 4032 | GRA-MENENDEZ S | 1 | 0.066 |
| 4033 | GRABARCZYK TR | 1 | 0.066 |
| 4034 | GRAHAM-BROWN MPM | 1 | 0.066 |
| 4035 | GRAMS ME | 1 | 0.066 |
| 4036 | GRANSTROM O | 1 | 0.066 |
| 4037 | GRASSI G | 1 | 0.066 |
| 4038 | GRASSOS C | 1 | 0.066 |
| 4039 | GRATACOS M | 1 | 0.066 |
| 4040 | GRAY AM | 1 | 0.066 |
| 4041 | GRAZIANO G | 1 | 0.066 |
| 4042 | GRECO B | 1 | 0.066 |
| 4043 | GRECO E | 1 | 0.066 |
| 4044 | GREEN J | 1 | 0.066 |
| 4045 | GREEN L | 1 | 0.066 |
| 4046 | GREGG EW | 1 | 0.066 |
| 4047 | GREITER-WILKE A | 1 | 0.066 |
| 4048 | GRENET G | 1 | 0.066 |
| 4049 | GRIEVE DJ | 1 | 0.066 |
| 4050 | GRIFFIN M | 1 | 0.066 |
| 4051 | GRIFFIN MD | 1 | 0.066 |
| 4052 | GRIFFIN TP | 1 | 0.066 |
| 4053 | GRIGORIAN-SHAMAGIAN L | 1 | 0.066 |
| 4054 | GRIGOROPOULOU P | 1 | 0.066 |
| 4055 | GRIMM NC | 1 | 0.066 |
| 4056 | GROBLER AF | 1 | 0.066 |
| 4057 | GROENENDYK J | 1 | 0.066 |
| 4058 | GRONDAHL MF | 1 | 0.066 |
| 4059 | GRONROS J | 1 | 0.066 |
| 4060 | GROOTHOF D | 1 | 0.066 |
| 4061 | GROSSI G | 1 | 0.066 |
| 4062 | GROSSMAN E | 1 | 0.066 |
| 4063 | GROSSMAN HL | 1 | 0.066 |
| 4064 | GROVE O | 1 | 0.066 |
| 4065 | GRUBB S | 1 | 0.066 |
| 4066 | GRUDEN G | 1 | 0.066 |
| 4067 | GRUNDTVIG M | 1 | 0.066 |
| 4068 | GRZESLO A | 1 | 0.066 |
| 4069 | GRZESZCZAK S | 1 | 0.066 |
| 4070 | GU JL | 1 | 0.066 |
| 4071 | GU JQ | 1 | 0.066 |
| 4072 | GU ZC | 1 | 0.066 |
| 4073 | GUALANDRO DM | 1 | 0.066 |
| 4074 | GUALILLO O | 1 | 0.066 |
| 4075 | GUEGUEN C | 1 | 0.066 |
| 4076 | GUERANDEL A | 1 | 0.066 |
| 4077 | GUERRA G | 1 | 0.066 |
| 4078 | GUERRA LC | 1 | 0.066 |
| 4079 | GUEYFFIER F | 1 | 0.066 |
| 4080 | GUITART XV | 1 | 0.066 |
| 4081 | GUL F | 1 | 0.066 |
| 4082 | GULATI R | 1 | 0.066 |
| 4083 | GULLESTAD L | 1 | 0.066 |
| 4084 | GULSIN GS | 1 | 0.066 |
| 4085 | GUMMERT J | 1 | 0.066 |
| 4086 | GUMPRECHT J | 1 | 0.066 |
| 4087 | GUNAPARN S | 1 | 0.066 |
| 4088 | GUNJI R | 1 | 0.066 |
| 4089 | GUNNINK SM | 1 | 0.066 |
| 4090 | GUO HY | 1 | 0.066 |
| 4091 | GUO JD | 1 | 0.066 |
| 4092 | GUO LX | 1 | 0.066 |
| 4093 | GUO M | 1 | 0.066 |
| 4094 | GUO R | 1 | 0.066 |
| 4095 | GUPTA C | 1 | 0.066 |
| 4096 | GUPTA P | 1 | 0.066 |
| 4097 | GUPTA Y | 1 | 0.066 |
| 4098 | GURAV YK | 1 | 0.066 |
| 4099 | GURMU Y | 1 | 0.066 |
| 4100 | GURSES KM | 1 | 0.066 |
| 4101 | GUSTAFSSON I | 1 | 0.066 |
| 4102 | GUSTAVSON S | 1 | 0.066 |
| 4103 | GUTH BD | 1 | 0.066 |
| 4104 | GUTHOFF M | 1 | 0.066 |
| 4105 | GUTHRIE RM | 1 | 0.066 |
| 4106 | GUTIERREZ AD | 1 | 0.066 |
| 4107 | GUTMAN SJ | 1 | 0.066 |
| 4108 | GUZZARDI DG | 1 | 0.066 |
| 4109 | HA HH | 1 | 0.066 |
| 4110 | HA KH | 1 | 0.066 |
| 4111 | HAAS B | 1 | 0.066 |
| 4112 | HAAS M | 1 | 0.066 |
| 4113 | HACH T | 1 | 0.066 |
| 4114 | HADDAD AN | 1 | 0.066 |
| 4115 | HADDAD H | 1 | 0.066 |
| 4116 | HADDAD JA | 1 | 0.066 |
| 4117 | HAEITMANN R | 1 | 0.066 |
| 4118 | HAEUSLER RA | 1 | 0.066 |
| 4119 | HAHN S | 1 | 0.066 |
| 4120 | HAJJAR R | 1 | 0.066 |
| 4121 | HAJJAR RJ | 1 | 0.066 |
| 4122 | HAJRA A | 1 | 0.066 |
| 4123 | HALDEN TAS | 1 | 0.066 |
| 4124 | HALIMI S | 1 | 0.066 |
| 4125 | HALL S | 1 | 0.066 |
| 4126 | HALL WL | 1 | 0.066 |
| 4127 | HALLEN N | 1 | 0.066 |
| 4128 | HALLOW MK | 1 | 0.066 |
| 4129 | HALVORSEN YD | 1 | 0.066 |
| 4130 | HAMA S | 1 | 0.066 |
| 4131 | HAMANOUE N | 1 | 0.066 |
| 4132 | HAMASAKI H | 1 | 0.066 |
| 4133 | HAMASAKI T | 1 | 0.066 |
| 4134 | HAMDANI N | 1 | 0.066 |
| 4135 | HAMDY O | 1 | 0.066 |
| 4136 | HAMMER KP | 1 | 0.066 |
| 4137 | HAMMOUD J | 1 | 0.066 |
| 4138 | HAMMOUDI N | 1 | 0.066 |
| 4139 | HAMO CE | 1 | 0.066 |
| 4140 | HAN J | 1 | 0.066 |
| 4141 | HAN SJ | 1 | 0.066 |
| 4142 | HAN XY | 1 | 0.066 |
| 4143 | HANAOKA K | 1 | 0.066 |
| 4144 | HANAOKA M | 1 | 0.066 |
| 4145 | HANDELSMAN S | 1 | 0.066 |
| 4146 | HANEFELD M | 1 | 0.066 |
| 4147 | HANF A | 1 | 0.066 |
| 4148 | HANNUKAINEN JC | 1 | 0.066 |
| 4149 | HANSEN J | 1 | 0.066 |
| 4150 | HANSEN L | 1 | 0.066 |
| 4151 | HANSSEN NMJ | 1 | 0.066 |
| 4152 | HANSSEN R | 1 | 0.066 |
| 4153 | HAO ZR | 1 | 0.066 |
| 4154 | HAR R | 1 | 0.066 |
| 4155 | HARA K | 1 | 0.066 |
| 4156 | HARA T | 1 | 0.066 |
| 4157 | HARAGUCHI A | 1 | 0.066 |
| 4158 | HARDTNER C | 1 | 0.066 |
| 4159 | HARE MJL | 1 | 0.066 |
| 4160 | HARING HU | 1 | 0.066 |
| 4161 | HARKNESS K | 1 | 0.066 |
| 4162 | HARMS HJ | 1 | 0.066 |
| 4163 | HARRIS KB | 1 | 0.066 |
| 4164 | HARRIS R | 1 | 0.066 |
| 4165 | HARRISON D | 1 | 0.066 |
| 4166 | HART L | 1 | 0.066 |
| 4167 | HART R | 1 | 0.066 |
| 4168 | HART SE | 1 | 0.066 |
| 4169 | HARTMAN I | 1 | 0.066 |
| 4170 | HARTMANN A | 1 | 0.066 |
| 4171 | HARTMANN B | 1 | 0.066 |
| 4172 | HARTUPEE J | 1 | 0.066 |
| 4173 | HARUHARA K | 1 | 0.066 |
| 4174 | HARVIE BM | 1 | 0.066 |
| 4175 | HASBAK P | 1 | 0.066 |
| 4176 | HASHEMI M | 1 | 0.066 |
| 4177 | HASHIMOTO N | 1 | 0.066 |
| 4178 | HASHIMOTO T | 1 | 0.066 |
| 4179 | HASHIMURA K | 1 | 0.066 |
| 4180 | HASHIZUME K | 1 | 0.066 |
| 4181 | HASS MDS | 1 | 0.066 |
| 4182 | HASSANABAD AF | 1 | 0.066 |
| 4183 | HASUNUMA T | 1 | 0.066 |
| 4184 | HATANO M | 1 | 0.066 |
| 4185 | HATTA Y | 1 | 0.066 |
| 4186 | HATTORI A | 1 | 0.066 |
| 4187 | HATTORI S | 1 | 0.066 |
| 4188 | HATTORI Y | 1 | 0.066 |
| 4189 | HATZIAGELAKI E | 1 | 0.066 |
| 4190 | HAU N | 1 | 0.066 |
| 4191 | HAUSKE SJ | 1 | 0.066 |
| 4192 | HAWLEY C | 1 | 0.066 |
| 4193 | HAWLEY CE | 1 | 0.066 |
| 4194 | HAWLEY CM | 1 | 0.066 |
| 4195 | HAYASHI I | 1 | 0.066 |
| 4196 | HAYASHI N | 1 | 0.066 |
| 4197 | HAYASHI T | 1 | 0.066 |
| 4198 | HAYASHIDA K | 1 | 0.066 |
| 4199 | HAZLETT I | 1 | 0.066 |
| 4200 | HE ZC | 1 | 0.066 |
| 4201 | HEAD GA | 1 | 0.066 |
| 4202 | HEALD AH | 1 | 0.066 |
| 4203 | HEATH K | 1 | 0.066 |
| 4204 | HECKMAN GA | 1 | 0.066 |
| 4205 | HEDRINGTON M | 1 | 0.066 |
| 4206 | HEDRINGTON MS | 1 | 0.066 |
| 4207 | HEER M | 1 | 0.066 |
| 4208 | HEERSPINK HJ | 1 | 0.066 |
| 4209 | HEIDENREICH PA | 1 | 0.066 |
| 4210 | HEISE T | 1 | 0.066 |
| 4211 | HEJLESEN O | 1 | 0.066 |
| 4212 | HELD C | 1 | 0.066 |
| 4213 | HELLER SR | 1 | 0.066 |
| 4214 | HELMSTADTER J | 1 | 0.066 |
| 4215 | HEMINGWAY H | 1 | 0.066 |
| 4216 | HEMMINGSEN B | 1 | 0.066 |
| 4217 | HENRY RR | 1 | 0.066 |
| 4218 | HENSON J | 1 | 0.066 |
| 4219 | HEO CU | 1 | 0.066 |
| 4220 | HERAT L | 1 | 0.066 |
| 4221 | HERBST R | 1 | 0.066 |
| 4222 | HERINGS RMC | 1 | 0.066 |
| 4223 | HERNANDEZ-MIJARES A | 1 | 0.066 |
| 4224 | HERRERA M | 1 | 0.066 |
| 4225 | HERSHON K | 1 | 0.066 |
| 4226 | HERZYK D | 1 | 0.066 |
| 4227 | HESS DA | 1 | 0.066 |
| 4228 | HETTIARACHCHI M | 1 | 0.066 |
| 4229 | HETTIGE TS | 1 | 0.066 |
| 4230 | HEUSSER K | 1 | 0.066 |
| 4231 | HEYWOOD S | 1 | 0.066 |
| 4232 | HIATT WR | 1 | 0.066 |
| 4233 | HIERRO-BUJALANCE C | 1 | 0.066 |
| 4234 | HIGA M | 1 | 0.066 |
| 4235 | HIGUCHI S | 1 | 0.066 |
| 4236 | HIKI M | 1 | 0.066 |
| 4237 | HILGENDORF I | 1 | 0.066 |
| 4238 | HILKER M | 1 | 0.066 |
| 4239 | HILL CL | 1 | 0.066 |
| 4240 | HILLIS G | 1 | 0.066 |
| 4241 | HINATA T | 1 | 0.066 |
| 4242 | HIRAI T | 1 | 0.066 |
| 4243 | HIRAIDE S | 1 | 0.066 |
| 4244 | HIRAKAWA Y | 1 | 0.066 |
| 4245 | HIRASE T | 1 | 0.066 |
| 4246 | HIRATA Y | 1 | 0.066 |
| 4247 | HIRAYAMA BA | 1 | 0.066 |
| 4248 | HIRD TR | 1 | 0.066 |
| 4249 | HIROMURA M | 1 | 0.066 |
| 4250 | HIROOKA Y | 1 | 0.066 |
| 4251 | HIROSE M | 1 | 0.066 |
| 4252 | HIROSE S | 1 | 0.066 |
| 4253 | HIROTA SA | 1 | 0.066 |
| 4254 | HIRSCH IB | 1 | 0.066 |
| 4255 | HIRUKAWA H | 1 | 0.066 |
| 4256 | HIRUMA S | 1 | 0.066 |
| 4257 | HISATAKE S | 1 | 0.066 |
| 4258 | HISAUCHI I | 1 | 0.066 |
| 4259 | HISER D | 1 | 0.066 |
| 4260 | HISHIDA A | 1 | 0.066 |
| 4261 | HITTLE L | 1 | 0.066 |
| 4262 | HIYOSHI T | 1 | 0.066 |
| 4263 | HNG TM | 1 | 0.066 |
| 4264 | HO A | 1 | 0.066 |
| 4265 | HOANG T | 1 | 0.066 |
| 4266 | HOBBS TM | 1 | 0.066 |
| 4267 | HOCHER B | 1 | 0.066 |
| 4268 | HOCHHAUSER E | 1 | 0.066 |
| 4269 | HODISH I | 1 | 0.066 |
| 4270 | HODOROGEA AS | 1 | 0.066 |
| 4271 | HOES AW | 1 | 0.066 |
| 4272 | HOHENSTEIN P | 1 | 0.066 |
| 4273 | HOJS R | 1 | 0.066 |
| 4274 | HOLLANDER K | 1 | 0.066 |
| 4275 | HOLLENBERG MD | 1 | 0.066 |
| 4276 | HOLMAN R | 1 | 0.066 |
| 4277 | HOLMES-WALKER DJ | 1 | 0.066 |
| 4278 | HOLT T | 1 | 0.066 |
| 4279 | HOME PD | 1 | 0.066 |
| 4280 | HOMPESCH M | 1 | 0.066 |
| 4281 | HON G | 1 | 0.066 |
| 4282 | HONDA A | 1 | 0.066 |
| 4283 | HONJO J | 1 | 0.066 |
| 4284 | HONKA MJ | 1 | 0.066 |
| 4285 | HONMA T | 1 | 0.066 |
| 4286 | HOPPE N | 1 | 0.066 |
| 4287 | HORBLITT A | 1 | 0.066 |
| 4288 | HORIE I | 1 | 0.066 |
| 4289 | HORIKAWA T | 1 | 0.066 |
| 4290 | HORIKOSHI T | 1 | 0.066 |
| 4291 | HORIUCHI R | 1 | 0.066 |
| 4292 | HORMAN S | 1 | 0.066 |
| 4293 | HORNUM M | 1 | 0.066 |
| 4294 | HORSWELL R | 1 | 0.066 |
| 4295 | HORVATH EM | 1 | 0.066 |
| 4296 | HOSHIDE S | 1 | 0.066 |
| 4297 | HOSOKAWA S | 1 | 0.066 |
| 4298 | HOU FF | 1 | 0.066 |
| 4299 | HOU SF | 1 | 0.066 |
| 4300 | HOUGH TA | 1 | 0.066 |
| 4301 | HOUSER SR | 1 | 0.066 |
| 4302 | HRICOVA J | 1 | 0.066 |
| 4303 | HRISTOVA M | 1 | 0.066 |
| 4304 | HSIA DS | 1 | 0.066 |
| 4305 | HSU JC | 1 | 0.066 |
| 4306 | HSU LC | 1 | 0.066 |
| 4307 | HTIKE ZZ | 1 | 0.066 |
| 4308 | HTUN W | 1 | 0.066 |
| 4309 | HU G | 1 | 0.066 |
| 4310 | HU MD | 1 | 0.066 |
| 4311 | HU SY | 1 | 0.066 |
| 4312 | HU Y | 1 | 0.066 |
| 4313 | HU YM | 1 | 0.066 |
| 4314 | HU YS | 1 | 0.066 |
| 4315 | HUANG CC | 1 | 0.066 |
| 4316 | HUANG CJ | 1 | 0.066 |
| 4317 | HUANG F | 1 | 0.066 |
| 4318 | HUANG JA | 1 | 0.066 |
| 4319 | HUANG JY | 1 | 0.066 |
| 4320 | HUANG KC | 1 | 0.066 |
| 4321 | HUANG SC | 1 | 0.066 |
| 4322 | HUANG XN | 1 | 0.066 |
| 4323 | HUANG Y | 1 | 0.066 |
| 4324 | HUANG YF | 1 | 0.066 |
| 4325 | HUANG YL | 1 | 0.066 |
| 4326 | HUCKER W | 1 | 0.066 |
| 4327 | HUDSON M | 1 | 0.066 |
| 4328 | HUE L | 1 | 0.066 |
| 4329 | HUELSMANN M | 1 | 0.066 |
| 4330 | HUFERT K | 1 | 0.066 |
| 4331 | HULOT JS | 1 | 0.066 |
| 4332 | HUMMEL M | 1 | 0.066 |
| 4333 | HUMPHREY LL | 1 | 0.066 |
| 4334 | HUNG A | 1 | 0.066 |
| 4335 | HUNG CC | 1 | 0.066 |
| 4336 | HUNG J | 1 | 0.066 |
| 4337 | HUNT B | 1 | 0.066 |
| 4338 | HUNTSBERRY AM | 1 | 0.066 |
| 4339 | HUPFELD C | 1 | 0.066 |
| 4340 | HURREN KM | 1 | 0.066 |
| 4341 | HUSAIN I | 1 | 0.066 |
| 4342 | HUSKAMP HA | 1 | 0.066 |
| 4343 | HUSSAIN MA | 1 | 0.066 |
| 4344 | HUSSAIN Q | 1 | 0.066 |
| 4345 | HUSSAIN S | 1 | 0.066 |
| 4346 | HUSSEIN AM | 1 | 0.066 |
| 4347 | HUTFLESS S | 1 | 0.066 |
| 4348 | HUYGHE I | 1 | 0.066 |
| 4349 | HWANG I | 1 | 0.066 |
| 4350 | HWANG IC | 1 | 0.066 |
| 4351 | HWANG SS | 1 | 0.066 |
| 4352 | HYDERY T | 1 | 0.066 |
| 4353 | HYODO T | 1 | 0.066 |
| 4354 | HYOGO H | 1 | 0.066 |
| 4355 | IACONETTI C | 1 | 0.066 |
| 4356 | IANNANTUONI F | 1 | 0.066 |
| 4357 | IANNIRUBERTO M | 1 | 0.066 |
| 4358 | IBI M | 1 | 0.066 |
| 4359 | IBRAHIM M | 1 | 0.066 |
| 4360 | ICHIJO T | 1 | 0.066 |
| 4361 | ICHINOSE F | 1 | 0.066 |
| 4362 | IDA T | 1 | 0.066 |
| 4363 | IDE K | 1 | 0.066 |
| 4364 | IDE S | 1 | 0.066 |
| 4365 | IDORN T | 1 | 0.066 |
| 4366 | IDRIS-KHODJA N | 1 | 0.066 |
| 4367 | IGAKI Y | 1 | 0.066 |
| 4368 | IGATA S | 1 | 0.066 |
| 4369 | IGNJATOVIC J | 1 | 0.066 |
| 4370 | IJZERMAN RG | 1 | 0.066 |
| 4371 | IKEDA K | 1 | 0.066 |
| 4372 | IKEDA T | 1 | 0.066 |
| 4373 | IKEDA Y | 1 | 0.066 |
| 4374 | IKEHARA K | 1 | 0.066 |
| 4375 | IKEHARA Y | 1 | 0.066 |
| 4376 | ILATOVSKAYA DV | 1 | 0.066 |
| 4377 | ILIESIU AM | 1 | 0.066 |
| 4378 | ILIODROMITIS E | 1 | 0.066 |
| 4379 | ILIODROMITIS EK | 1 | 0.066 |
| 4380 | ILOEJE U | 1 | 0.066 |
| 4381 | ILOMAKI J | 1 | 0.066 |
| 4382 | IMAJO K | 1 | 0.066 |
| 4383 | IMAZEKI H | 1 | 0.066 |
| 4384 | IMAZU M | 1 | 0.066 |
| 4385 | IMRAN H | 1 | 0.066 |
| 4386 | INADA C | 1 | 0.066 |
| 4387 | INAZAWA T | 1 | 0.066 |
| 4388 | INAZUMI K | 1 | 0.066 |
| 4389 | INDOLFI C | 1 | 0.066 |
| 4390 | INFANTE-GARCIA C | 1 | 0.066 |
| 4391 | INGHAM M | 1 | 0.066 |
| 4392 | INMAN D | 1 | 0.066 |
| 4393 | INOGUCHI T | 1 | 0.066 |
| 4394 | INOKUCHI T | 1 | 0.066 |
| 4395 | INOUE I | 1 | 0.066 |
| 4396 | INOUE M | 1 | 0.066 |
| 4397 | INOUE MK | 1 | 0.066 |
| 4398 | INOUE Y | 1 | 0.066 |
| 4399 | IOANNIDIS I | 1 | 0.066 |
| 4400 | IOANNIDOU E | 1 | 0.066 |
| 4401 | IORI E | 1 | 0.066 |
| 4402 | IOZZO P | 1 | 0.066 |
| 4403 | IQBAL A | 1 | 0.066 |
| 4404 | IQBAL N | 1 | 0.066 |
| 4405 | ISAJI M | 1 | 0.066 |
| 4406 | ISARANUWATCHAI S | 1 | 0.066 |
| 4407 | ISE T | 1 | 0.066 |
| 4408 | ISHIBASHI R | 1 | 0.066 |
| 4409 | ISHIDA N | 1 | 0.066 |
| 4410 | ISHIHARA H | 1 | 0.066 |
| 4411 | ISHII M | 1 | 0.066 |
| 4412 | ISHIKAWA T | 1 | 0.066 |
| 4413 | ISHIKAWA-TAKEMURA Y | 1 | 0.066 |
| 4414 | ISHIZAWA K | 1 | 0.066 |
| 4415 | ISKAKOVA S | 1 | 0.066 |
| 4416 | ISLAM MN | 1 | 0.066 |
| 4417 | ISO K | 1 | 0.066 |
| 4418 | ISSIKI M | 1 | 0.066 |
| 4419 | ITABASHI N | 1 | 0.066 |
| 4420 | ITANO S | 1 | 0.066 |
| 4421 | ITO A | 1 | 0.066 |
| 4422 | ITO T | 1 | 0.066 |
| 4423 | ITOH F | 1 | 0.066 |
| 4424 | IVEY-MIRANDA J | 1 | 0.066 |
| 4425 | IVKIN D | 1 | 0.066 |
| 4426 | IWAMOTO T | 1 | 0.066 |
| 4427 | IWASAKI K | 1 | 0.066 |
| 4428 | IWASAKI Y | 1 | 0.066 |
| 4429 | IWATA H | 1 | 0.066 |
| 4430 | IYOHA E | 1 | 0.066 |
| 4431 | IZQUIERDO MC | 1 | 0.066 |
| 4432 | IZUTSU T | 1 | 0.066 |
| 4433 | IZZO JL | 1 | 0.066 |
| 4434 | JAARSMALL T | 1 | 0.066 |
| 4435 | JABIR NR | 1 | 0.066 |
| 4436 | JACKSON KL | 1 | 0.066 |
| 4437 | JACOB R | 1 | 0.066 |
| 4438 | JACOB S | 1 | 0.066 |
| 4439 | JADRIJEVIC S | 1 | 0.066 |
| 4440 | JAFFER S | 1 | 0.066 |
| 4441 | JAGHUTRIZ BA | 1 | 0.066 |
| 4442 | JAGU B | 1 | 0.066 |
| 4443 | JAHANGIR E | 1 | 0.066 |
| 4444 | JAHNG JWS | 1 | 0.066 |
| 4445 | JAIKUMKAO K | 1 | 0.066 |
| 4446 | JAIME M | 1 | 0.066 |
| 4447 | JAIN R | 1 | 0.066 |
| 4448 | JAIWONGKAM T | 1 | 0.066 |
| 4449 | JAKHER H | 1 | 0.066 |
| 4450 | JAKOBSEN PE | 1 | 0.066 |
| 4451 | JAKUBIAK M | 1 | 0.066 |
| 4452 | JAKUPOVIC L | 1 | 0.066 |
| 4453 | JAMAL A | 1 | 0.066 |
| 4454 | JAMEEL A | 1 | 0.066 |
| 4455 | JAMIALAHMADI T | 1 | 0.066 |
| 4456 | JAN S | 1 | 0.066 |
| 4457 | JANCEV M | 1 | 0.066 |
| 4458 | JANDELEIT-DAHM KAM | 1 | 0.066 |
| 4459 | JANG JY | 1 | 0.066 |
| 4460 | JANISTA C | 1 | 0.066 |
| 4461 | JANKOWSKA EA | 1 | 0.066 |
| 4462 | JANSEN H | 1 | 0.066 |
| 4463 | JANSKY P | 1 | 0.066 |
| 4464 | JANSSENS J | 1 | 0.066 |
| 4465 | JANSSENS S | 1 | 0.066 |
| 4466 | JAPELJ M | 1 | 0.066 |
| 4467 | JAROLIM P | 1 | 0.066 |
| 4468 | JARRELL DK | 1 | 0.066 |
| 4469 | JARVIS S | 1 | 0.066 |
| 4470 | JAVED Z | 1 | 0.066 |
| 4471 | JAYABALLA R | 1 | 0.066 |
| 4472 | JAYAWARDENE D | 1 | 0.066 |
| 4473 | JAZI M | 1 | 0.066 |
| 4474 | JE NK | 1 | 0.066 |
| 4475 | JEAN HJ | 1 | 0.066 |
| 4476 | JELAKOVIC B | 1 | 0.066 |
| 4477 | JENEY V | 1 | 0.066 |
| 4478 | JENKINS A | 1 | 0.066 |
| 4479 | JENKINS B | 1 | 0.066 |
| 4480 | JENSEN J | 1 | 0.066 |
| 4481 | JENSEN MH | 1 | 0.066 |
| 4482 | JENSEN MT | 1 | 0.066 |
| 4483 | JENSSEN T | 1 | 0.066 |
| 4484 | JEON HJ | 1 | 0.066 |
| 4485 | JEON JY | 1 | 0.066 |
| 4486 | JEONG D | 1 | 0.066 |
| 4487 | JERMENDY G | 1 | 0.066 |
| 4488 | JERMYN R | 1 | 0.066 |
| 4489 | JESSEN N | 1 | 0.066 |
| 4490 | JESSUP M | 1 | 0.066 |
| 4491 | JEYAPRAKASH M | 1 | 0.066 |
| 4492 | JHA JC | 1 | 0.066 |
| 4493 | JHA V | 1 | 0.066 |
| 4494 | JHUANG WJ | 1 | 0.066 |
| 4495 | JHUO SJ | 1 | 0.066 |
| 4496 | JI QH | 1 | 0.066 |
| 4497 | JI XG | 1 | 0.066 |
| 4498 | JIAN Z | 1 | 0.066 |
| 4499 | JIANG AX | 1 | 0.066 |
| 4500 | JIANG BP | 1 | 0.066 |
| 4501 | JIANG F | 1 | 0.066 |
| 4502 | JIANG HC | 1 | 0.066 |
| 4503 | JIANG WJ | 1 | 0.066 |
| 4504 | JIANG X | 1 | 0.066 |
| 4505 | JIANG YH | 1 | 0.066 |
| 4506 | JIANG ZZ | 1 | 0.066 |
| 4507 | JIMENEZ DL | 1 | 0.066 |
| 4508 | JIMENEZ MTB | 1 | 0.066 |
| 4509 | JIMENEZ R | 1 | 0.066 |
| 4510 | JINNOUCHI K | 1 | 0.066 |
| 4511 | JINNOUCHI T | 1 | 0.066 |
| 4512 | JOH HK | 1 | 0.066 |
| 4513 | JOHANSSON I | 1 | 0.066 |
| 4514 | JOHANSSON P | 1 | 0.066 |
| 4515 | JOHANSSON U | 1 | 0.066 |
| 4516 | JOHNSON JT | 1 | 0.066 |
| 4517 | JOHNSON ME | 1 | 0.066 |
| 4518 | JOHNSSON K | 1 | 0.066 |
| 4519 | JOHNSTON R | 1 | 0.066 |
| 4520 | JOHO S | 1 | 0.066 |
| 4521 | JOIS B | 1 | 0.066 |
| 4522 | JOLLY G | 1 | 0.066 |
| 4523 | JONES PN | 1 | 0.066 |
| 4524 | JONG GP | 1 | 0.066 |
| 4525 | JONSSON-RYLANDER AC | 1 | 0.066 |
| 4526 | JORGENSEN NR | 1 | 0.066 |
| 4527 | JOSEPH S | 1 | 0.066 |
| 4528 | JOY M | 1 | 0.066 |
| 4529 | JUANATEY JRG | 1 | 0.066 |
| 4530 | JUDGE PK | 1 | 0.066 |
| 4531 | JULIO SMC | 1 | 0.066 |
| 4532 | JUNG CH | 1 | 0.066 |
| 4533 | JUNG DW | 1 | 0.066 |
| 4534 | JUNG HS | 1 | 0.066 |
| 4535 | JUNG S | 1 | 0.066 |
| 4536 | JURETIC A | 1 | 0.066 |
| 4537 | JURGENS M | 1 | 0.066 |
| 4538 | KABIR G | 1 | 0.066 |
| 4539 | KABIR MG | 1 | 0.066 |
| 4540 | KACZYNSKI J | 1 | 0.066 |
| 4541 | KADAM P | 1 | 0.066 |
| 4542 | KADOGLOU NPE | 1 | 0.066 |
| 4543 | KADOGUCHI T | 1 | 0.066 |
| 4544 | KADOKAMI T | 1 | 0.066 |
| 4545 | KADOKURA T | 1 | 0.066 |
| 4546 | KAGAWA T | 1 | 0.066 |
| 4547 | KAHLER LKA | 1 | 0.066 |
| 4548 | KAHLER P | 1 | 0.066 |
| 4549 | KAI S | 1 | 0.066 |
| 4550 | KAJIWARA K | 1 | 0.066 |
| 4551 | KAKIUCHI S | 1 | 0.066 |
| 4552 | KAKUTANI N | 1 | 0.066 |
| 4553 | KALASZ H | 1 | 0.066 |
| 4554 | KALINOVIC S | 1 | 0.066 |
| 4555 | KALOGERIS A | 1 | 0.066 |
| 4556 | KALOGIROU MS | 1 | 0.066 |
| 4557 | KALRA J | 1 | 0.066 |
| 4558 | KALTHEUNER M | 1 | 0.066 |
| 4559 | KALUS JS | 1 | 0.066 |
| 4560 | KALYANARAMAN N | 1 | 0.066 |
| 4561 | KAM G | 1 | 0.066 |
| 4562 | KAMADA N | 1 | 0.066 |
| 4563 | KAMADA Y | 1 | 0.066 |
| 4564 | KAMEDA H | 1 | 0.066 |
| 4565 | KAMEDA R | 1 | 0.066 |
| 4566 | KAMEDA T | 1 | 0.066 |
| 4567 | KAMEI S | 1 | 0.066 |
| 4568 | KAMEL H | 1 | 0.066 |
| 4569 | KAMMOUN HL | 1 | 0.066 |
| 4570 | KAMOUCHI M | 1 | 0.066 |
| 4571 | KAMSTRA R | 1 | 0.066 |
| 4572 | KANAI Y | 1 | 0.066 |
| 4573 | KANDEEL F | 1 | 0.066 |
| 4574 | KANE MP | 1 | 0.066 |
| 4575 | KANEKI M | 1 | 0.066 |
| 4576 | KANG A | 1 | 0.066 |
| 4577 | KANG RH | 1 | 0.066 |
| 4578 | KANG SH | 1 | 0.066 |
| 4579 | KANG SK | 1 | 0.066 |
| 4580 | KANG WS | 1 | 0.066 |
| 4581 | KANJANAHATTAKIJ N | 1 | 0.066 |
| 4582 | KANT R | 1 | 0.066 |
| 4583 | KANTHARIDIS P | 1 | 0.066 |
| 4584 | KANUMILLI N | 1 | 0.066 |
| 4585 | KAO YH | 1 | 0.066 |
| 4586 | KAPLAN A | 1 | 0.066 |
| 4587 | KAPOOR K | 1 | 0.066 |
| 4588 | KARACA-MANDIC P | 1 | 0.066 |
| 4589 | KARAGIANNIS AI | 1 | 0.066 |
| 4590 | KARALLIEDDE J | 1 | 0.066 |
| 4591 | KARAMOUZIS I | 1 | 0.066 |
| 4592 | KARANATSIS N | 1 | 0.066 |
| 4593 | KARASHIMA S | 1 | 0.066 |
| 4594 | KARCK M | 1 | 0.066 |
| 4595 | KARLSSON C | 1 | 0.066 |
| 4596 | KAROPOULOS P | 1 | 0.066 |
| 4597 | KARPOV A | 1 | 0.066 |
| 4598 | KARSENTY G | 1 | 0.066 |
| 4599 | KASAHARA M | 1 | 0.066 |
| 4600 | KASAI T | 1 | 0.066 |
| 4601 | KASAMA S | 1 | 0.066 |
| 4602 | KASCHINA E | 1 | 0.066 |
| 4603 | KASETTY M | 1 | 0.066 |
| 4604 | KASHANI F | 1 | 0.066 |
| 4605 | KASHIHARA N | 1 | 0.066 |
| 4606 | KASHIWAGI Y | 1 | 0.066 |
| 4607 | KASHYAP S | 1 | 0.066 |
| 4608 | KASICHAYANULA S | 1 | 0.066 |
| 4609 | KASNER S | 1 | 0.066 |
| 4610 | KATAOKA H | 1 | 0.066 |
| 4611 | KATARE S | 1 | 0.066 |
| 4612 | KATAVETIN P | 1 | 0.066 |
| 4613 | KATAYAMA S | 1 | 0.066 |
| 4614 | KATAYAMA T | 1 | 0.066 |
| 4615 | KATO M | 1 | 0.066 |
| 4616 | KATO T | 1 | 0.066 |
| 4617 | KATOGIANNIS K | 1 | 0.066 |
| 4618 | KATSI V | 1 | 0.066 |
| 4619 | KATSOGIANNOS P | 1 | 0.066 |
| 4620 | KATSUMATA Y | 1 | 0.066 |
| 4621 | KATSUYA T | 1 | 0.066 |
| 4622 | KATTAN MW | 1 | 0.066 |
| 4623 | KATUS HA | 1 | 0.066 |
| 4624 | KATWAN OJ | 1 | 0.066 |
| 4625 | KATZ SD | 1 | 0.066 |
| 4626 | KATZMARZYK PT | 1 | 0.066 |
| 4627 | KAULFERSCH C | 1 | 0.066 |
| 4628 | KAUPKE C | 1 | 0.066 |
| 4629 | KAUR A | 1 | 0.066 |
| 4630 | KAUR P | 1 | 0.066 |
| 4631 | KAVALAM M | 1 | 0.066 |
| 4632 | KAVATI A | 1 | 0.066 |
| 4633 | KAWAGUCHI A | 1 | 0.066 |
| 4634 | KAWAGUCHI Y | 1 | 0.066 |
| 4635 | KAWAKAMI A | 1 | 0.066 |
| 4636 | KAWANAKA M | 1 | 0.066 |
| 4637 | KAWASAKI E | 1 | 0.066 |
| 4638 | KAWASHIMA M | 1 | 0.066 |
| 4639 | KAWASHIMA S | 1 | 0.066 |
| 4640 | KAWATA S | 1 | 0.066 |
| 4641 | KAWAZOE Y | 1 | 0.066 |
| 4642 | KAYAMA Y | 1 | 0.066 |
| 4643 | KAYPAKLI O | 1 | 0.066 |
| 4644 | KAZUTA K | 1 | 0.066 |
| 4645 | KEDIA R | 1 | 0.066 |
| 4646 | KEIGHTLEY G | 1 | 0.066 |
| 4647 | KEINAN-BOKER L | 1 | 0.066 |
| 4648 | KEIRNS J | 1 | 0.066 |
| 4649 | KELLERER M | 1 | 0.066 |
| 4650 | KELLY DP | 1 | 0.066 |
| 4651 | KELLY JL | 1 | 0.066 |
| 4652 | KELLY T | 1 | 0.066 |
| 4653 | KELTAI M | 1 | 0.066 |
| 4654 | KEMPLER P | 1 | 0.066 |
| 4655 | KEMPTHORNE-RAWSON J | 1 | 0.066 |
| 4656 | KENGNE AP | 1 | 0.066 |
| 4657 | KENNEDY S | 1 | 0.066 |
| 4658 | KEOGH JB | 1 | 0.066 |
| 4659 | KEPE V | 1 | 0.066 |
| 4660 | KERN E | 1 | 0.066 |
| 4661 | KERRIDGE RK | 1 | 0.066 |
| 4662 | KESHAVAMURTHY C | 1 | 0.066 |
| 4663 | KESHAVAMURTHY CB | 1 | 0.066 |
| 4664 | KESSLER L | 1 | 0.066 |
| 4665 | KHALIL AB | 1 | 0.066 |
| 4666 | KHAMAISI M | 1 | 0.066 |
| 4667 | KHAN AA | 1 | 0.066 |
| 4668 | KHAN AY | 1 | 0.066 |
| 4669 | KHAN F | 1 | 0.066 |
| 4670 | KHAN G | 1 | 0.066 |
| 4671 | KHAN MS | 1 | 0.066 |
| 4672 | KHAN RS | 1 | 0.066 |
| 4673 | KHAN S | 1 | 0.066 |
| 4674 | KHANDELWAL S | 1 | 0.066 |
| 4675 | KHANGURA D | 1 | 0.066 |
| 4676 | KHANNA R | 1 | 0.066 |
| 4677 | KHANNA YP | 1 | 0.066 |
| 4678 | KHARAT A | 1 | 0.066 |
| 4679 | KHARITON Y | 1 | 0.066 |
| 4680 | KHAT DZ | 1 | 0.066 |
| 4681 | KHATIK GL | 1 | 0.066 |
| 4682 | KHAZIM K | 1 | 0.066 |
| 4683 | KHEIRANDISH M | 1 | 0.066 |
| 4684 | KHEMAIS-BENKHIAT S | 1 | 0.066 |
| 4685 | KHERAD B | 1 | 0.066 |
| 4686 | KHITAN Z | 1 | 0.066 |
| 4687 | KHOTSKINA AS | 1 | 0.066 |
| 4688 | KHRAISHAH H | 1 | 0.066 |
| 4689 | KIDOKORO K | 1 | 0.066 |
| 4690 | KIEL AM | 1 | 0.066 |
| 4691 | KIKUCHI S | 1 | 0.066 |
| 4692 | KILOV G | 1 | 0.066 |
| 4693 | KILPATRICK ES | 1 | 0.066 |
| 4694 | KIM CH | 1 | 0.066 |
| 4695 | KIM DH | 1 | 0.066 |
| 4696 | KIM ES | 1 | 0.066 |
| 4697 | KIM GS | 1 | 0.066 |
| 4698 | KIM GW | 1 | 0.066 |
| 4699 | KIM HK | 1 | 0.066 |
| 4700 | KIM I | 1 | 0.066 |
| 4701 | KIM JS | 1 | 0.066 |
| 4702 | KIM K | 1 | 0.066 |
| 4703 | KIM KM | 1 | 0.066 |
| 4704 | KIM MK | 1 | 0.066 |
| 4705 | KIM SC | 1 | 0.066 |
| 4706 | KIM SK | 1 | 0.066 |
| 4707 | KIM SR | 1 | 0.066 |
| 4708 | KIM SY | 1 | 0.066 |
| 4709 | KIM TK | 1 | 0.066 |
| 4710 | KIM TN | 1 | 0.066 |
| 4711 | KIM WH | 1 | 0.066 |
| 4712 | KIM YB | 1 | 0.066 |
| 4713 | KIM YC | 1 | 0.066 |
| 4714 | KIM YG | 1 | 0.066 |
| 4715 | KIM YJ | 1 | 0.066 |
| 4716 | KIMURA H | 1 | 0.066 |
| 4717 | KIMURA-MEDORIMA ST | 1 | 0.066 |
| 4718 | KIN H | 1 | 0.066 |
| 4719 | KINDERMANN I | 1 | 0.066 |
| 4720 | KING J | 1 | 0.066 |
| 4721 | KING JA | 1 | 0.066 |
| 4722 | KINGUCHI S | 1 | 0.066 |
| 4723 | KINNEALLY TL | 1 | 0.066 |
| 4724 | KINOSHITA T | 1 | 0.066 |
| 4725 | KINUGAWA K | 1 | 0.066 |
| 4726 | KINUGAWA S | 1 | 0.066 |
| 4727 | KIRJAVAINEN AK | 1 | 0.066 |
| 4728 | KIS O | 1 | 0.066 |
| 4729 | KIS SG | 1 | 0.066 |
| 4730 | KISANUKI K | 1 | 0.066 |
| 4731 | KISHI S | 1 | 0.066 |
| 4732 | KISHI T | 1 | 0.066 |
| 4733 | KISHOR K | 1 | 0.066 |
| 4734 | KISS RG | 1 | 0.066 |
| 4735 | KISSELA B | 1 | 0.066 |
| 4736 | KISTLER P | 1 | 0.066 |
| 4737 | KISTORP C | 1 | 0.066 |
| 4738 | KISTORP CM | 1 | 0.066 |
| 4739 | KITADA K | 1 | 0.066 |
| 4740 | KITAMOTO T | 1 | 0.066 |
| 4741 | KITAMURA K | 1 | 0.066 |
| 4742 | KITAZAWA H | 1 | 0.066 |
| 4743 | KITAZONO T | 1 | 0.066 |
| 4744 | KITSIOS K | 1 | 0.066 |
| 4745 | KITZMAN DW | 1 | 0.066 |
| 4746 | KIUCHI MG | 1 | 0.066 |
| 4747 | KJAER A | 1 | 0.066 |
| 4748 | KJAER LK | 1 | 0.066 |
| 4749 | KJOLBY M | 1 | 0.066 |
| 4750 | KLEIN T | 1 | 0.066 |
| 4751 | KLEMENS CA | 1 | 0.066 |
| 4752 | KLESSEN D | 1 | 0.066 |
| 4753 | KLIMONTOV VV | 1 | 0.066 |
| 4754 | KLOECKER DE | 1 | 0.066 |
| 4755 | KLUGER A | 1 | 0.066 |
| 4756 | KLUGER SL | 1 | 0.066 |
| 4757 | KNIGHT B | 1 | 0.066 |
| 4758 | KO SF | 1 | 0.066 |
| 4759 | KO YG | 1 | 0.066 |
| 4760 | KOBALAVA ZD | 1 | 0.066 |
| 4761 | KOBAYASHI A | 1 | 0.066 |
| 4762 | KOBAYASHI R | 1 | 0.066 |
| 4763 | KOBAYASHI S | 1 | 0.066 |
| 4764 | KOBAYASHI Y | 1 | 0.066 |
| 4765 | KOBYLIAK N | 1 | 0.066 |
| 4766 | KOCYIGIT D | 1 | 0.066 |
| 4767 | KODANI E | 1 | 0.066 |
| 4768 | KODERA S | 1 | 0.066 |
| 4769 | KODGULE R | 1 | 0.066 |
| 4770 | KOEMAN A | 1 | 0.066 |
| 4771 | KOEZUKA R | 1 | 0.066 |
| 4772 | KOGAWA K | 1 | 0.066 |
| 4773 | KOH ES | 1 | 0.066 |
| 4774 | KOH KK | 1 | 0.066 |
| 4775 | KOH SK | 1 | 0.066 |
| 4776 | KOHARA K | 1 | 0.066 |
| 4777 | KOHASHI K | 1 | 0.066 |
| 4778 | KOHN CG | 1 | 0.066 |
| 4779 | KOIWAI K | 1 | 0.066 |
| 4780 | KOIZUMI M | 1 | 0.066 |
| 4781 | KOKKINOS A | 1 | 0.066 |
| 4782 | KOKUBO H | 1 | 0.066 |
| 4783 | KOLANSKY D | 1 | 0.066 |
| 4784 | KOLBER MR | 1 | 0.066 |
| 4785 | KOLESNYK I | 1 | 0.066 |
| 4786 | KOMALA MG | 1 | 0.066 |
| 4787 | KOMATSU S | 1 | 0.066 |
| 4788 | KOMETANI M | 1 | 0.066 |
| 4789 | KOMURO I | 1 | 0.066 |
| 4790 | KONARI N | 1 | 0.066 |
| 4791 | KONDO M | 1 | 0.066 |
| 4792 | KONENKOV VI | 1 | 0.066 |
| 4793 | KONG APS | 1 | 0.066 |
| 4794 | KONG SX | 1 | 0.066 |
| 4795 | KONISHI H | 1 | 0.066 |
| 4796 | KONKALMATT PR | 1 | 0.066 |
| 4797 | KONSTAM M | 1 | 0.066 |
| 4798 | KONSTAM MA | 1 | 0.066 |
| 4799 | KOOMEN JV | 1 | 0.066 |
| 4800 | KOPP M | 1 | 0.066 |
| 4801 | KOPPOLU D | 1 | 0.066 |
| 4802 | KOPYT N | 1 | 0.066 |
| 4803 | KORBUT AI | 1 | 0.066 |
| 4804 | KORDONOURI O | 1 | 0.066 |
| 4805 | KORKMAZ-ICOZ S | 1 | 0.066 |
| 4806 | KORNOWSKI R | 1 | 0.066 |
| 4807 | KOROTKYI O | 1 | 0.066 |
| 4808 | KOROWNYK C | 1 | 0.066 |
| 4809 | KORZH S | 1 | 0.066 |
| 4810 | KOSAKAI Y | 1 | 0.066 |
| 4811 | KOSEKI M | 1 | 0.066 |
| 4812 | KOSHIZAKA M | 1 | 0.066 |
| 4813 | KOSIOR DA | 1 | 0.066 |
| 4814 | KOSMIDOU N | 1 | 0.066 |
| 4815 | KOSTOMITSOPOULOS N | 1 | 0.066 |
| 4816 | KOSUGI K | 1 | 0.066 |
| 4817 | KOSUGI S | 1 | 0.066 |
| 4818 | KOTSEVA K | 1 | 0.066 |
| 4819 | KOTSIS V | 1 | 0.066 |
| 4820 | KOTTRA G | 1 | 0.066 |
| 4821 | KOUFAKIS T | 1 | 0.066 |
| 4822 | KOUNTOURAS J | 1 | 0.066 |
| 4823 | KOUNTOURI A | 1 | 0.066 |
| 4824 | KOUNTZ DS | 1 | 0.066 |
| 4825 | KOUSATHANA F | 1 | 0.066 |
| 4826 | KOUTSAMPASOPOULOS K | 1 | 0.066 |
| 4827 | KOUZ S | 1 | 0.066 |
| 4828 | KOVACS A | 1 | 0.066 |
| 4829 | KOVESDY C | 1 | 0.066 |
| 4830 | KOZUKA C | 1 | 0.066 |
| 4831 | KRAAKMAN MJ | 1 | 0.066 |
| 4832 | KRAENZLIN ME | 1 | 0.066 |
| 4833 | KRAMER MHH | 1 | 0.066 |
| 4834 | KRAMERS BJ | 1 | 0.066 |
| 4835 | KRANE V | 1 | 0.066 |
| 4836 | KRASNOVA M | 1 | 0.066 |
| 4837 | KRAUS O | 1 | 0.066 |
| 4838 | KRAUWINKEL W | 1 | 0.066 |
| 4839 | KRENZ JR | 1 | 0.066 |
| 4840 | KRETZLER M | 1 | 0.066 |
| 4841 | KREUTZ R | 1 | 0.066 |
| 4842 | KRISHNA G | 1 | 0.066 |
| 4843 | KRISTENSEN SL | 1 | 0.066 |
| 4844 | KROPP J | 1 | 0.066 |
| 4845 | KRUGER D | 1 | 0.066 |
| 4846 | KSHIRSAGAR RP | 1 | 0.066 |
| 4847 | KU W | 1 | 0.066 |
| 4848 | KUBO H | 1 | 0.066 |
| 4849 | KUBOTA Y | 1 | 0.066 |
| 4850 | KUECKER CM | 1 | 0.066 |
| 4851 | KUHN A | 1 | 0.066 |
| 4852 | KUIPER BD | 1 | 0.066 |
| 4853 | KUIPER JG | 1 | 0.066 |
| 4854 | KUIPERS DP | 1 | 0.066 |
| 4855 | KUKA J | 1 | 0.066 |
| 4856 | KULIKOV A | 1 | 0.066 |
| 4857 | KULKARNI AA | 1 | 0.066 |
| 4858 | KULKARNI S | 1 | 0.066 |
| 4859 | KULLBERG J | 1 | 0.066 |
| 4860 | KULLDORFF M | 1 | 0.066 |
| 4861 | KUMAGAI Y | 1 | 0.066 |
| 4862 | KUMAMARU H | 1 | 0.066 |
| 4863 | KUMAR J | 1 | 0.066 |
| 4864 | KUMARENDRAN B | 1 | 0.066 |
| 4865 | KUMASHIRO Y | 1 | 0.066 |
| 4866 | KUMBHANI DJ | 1 | 0.066 |
| 4867 | KUMFU S | 1 | 0.066 |
| 4868 | KUNTIC M | 1 | 0.066 |
| 4869 | KUO SC | 1 | 0.066 |
| 4870 | KUOKKANEN M | 1 | 0.066 |
| 4871 | KURDI M | 1 | 0.066 |
| 4872 | KURIBAYASHI N | 1 | 0.066 |
| 4873 | KURINAMI N | 1 | 0.066 |
| 4874 | KURIYAMA C | 1 | 0.066 |
| 4875 | KURLYANDSKAYA R | 1 | 0.066 |
| 4876 | KURODA H | 1 | 0.066 |
| 4877 | KUROSAKI E | 1 | 0.066 |
| 4878 | KURUKULASURIYA LR | 1 | 0.066 |
| 4879 | KUSANO E | 1 | 0.066 |
| 4880 | KUSCHMA MC | 1 | 0.066 |
| 4881 | KUSHIMA H | 1 | 0.066 |
| 4882 | KUSHIYAMA A | 1 | 0.066 |
| 4883 | KUSHWAHA SS | 1 | 0.066 |
| 4884 | KUSNIR J | 1 | 0.066 |
| 4885 | KUSTER GM | 1 | 0.066 |
| 4886 | KUTSCHKE WJ | 1 | 0.066 |
| 4887 | KUWABARA M | 1 | 0.066 |
| 4888 | KUZULUGIL D | 1 | 0.066 |
| 4889 | KVITNE KE | 1 | 0.066 |
| 4890 | KWON H | 1 | 0.066 |
| 4891 | KYOHARA M | 1 | 0.066 |
| 4892 | KYRIACHENKO Y | 1 | 0.066 |
| 4893 | LA ROCCA HPB | 1 | 0.066 |
| 4894 | LA VERDE A | 1 | 0.066 |
| 4895 | LACHEV V | 1 | 0.066 |
| 4896 | LACKNER KJ | 1 | 0.066 |
| 4897 | LACRETA F | 1 | 0.066 |
| 4898 | LAGO F | 1 | 0.066 |
| 4899 | LAHNWONG S | 1 | 0.066 |
| 4900 | LAI D | 1 | 0.066 |
| 4901 | LAI V | 1 | 0.066 |
| 4902 | LAI WH | 1 | 0.066 |
| 4903 | LAJOINIE A | 1 | 0.066 |
| 4904 | LAKSHMANAN M | 1 | 0.066 |
| 4905 | LALA A | 1 | 0.066 |
| 4906 | LALIC J | 1 | 0.066 |
| 4907 | LALIC S | 1 | 0.066 |
| 4908 | LALLY J | 1 | 0.066 |
| 4909 | LAM A | 1 | 0.066 |
| 4910 | LAM KSL | 1 | 0.066 |
| 4911 | LAMACCHIA O | 1 | 0.066 |
| 4912 | LAMAS PA | 1 | 0.066 |
| 4913 | LAMBA S | 1 | 0.066 |
| 4914 | LAMBADIARI VA | 1 | 0.066 |
| 4915 | LAMBERT GW | 1 | 0.066 |
| 4916 | LAMBRINOU E | 1 | 0.066 |
| 4917 | LAMBRINOUDAKI I | 1 | 0.066 |
| 4918 | LAMOTTE M | 1 | 0.066 |
| 4919 | LANAS F | 1 | 0.066 |
| 4920 | LANCKI N | 1 | 0.066 |
| 4921 | LANDA N | 1 | 0.066 |
| 4922 | LANDRAY MJ | 1 | 0.066 |
| 4923 | LANE W | 1 | 0.066 |
| 4924 | LANFEAR DE | 1 | 0.066 |
| 4925 | LANG B | 1 | 0.066 |
| 4926 | LANG C | 1 | 0.066 |
| 4927 | LANGERMAN H | 1 | 0.066 |
| 4928 | LANSANG MC | 1 | 0.066 |
| 4929 | LANSKE B | 1 | 0.066 |
| 4930 | LANZINGER S | 1 | 0.066 |
| 4931 | LAPOLLA A | 1 | 0.066 |
| 4932 | LARSEN EL | 1 | 0.066 |
| 4933 | LARSEN JR | 1 | 0.066 |
| 4934 | LARUE SJ | 1 | 0.066 |
| 4935 | LASHIN LS | 1 | 0.066 |
| 4936 | LATHAM K | 1 | 0.066 |
| 4937 | LATT TS | 1 | 0.066 |
| 4938 | LAU A | 1 | 0.066 |
| 4939 | LAU YM | 1 | 0.066 |
| 4940 | LAURENT I | 1 | 0.066 |
| 4941 | LAURING B | 1 | 0.066 |
| 4942 | LAUSVIG NL | 1 | 0.066 |
| 4943 | LAUTSCH D | 1 | 0.066 |
| 4944 | LAVIOLA L | 1 | 0.066 |
| 4945 | LAWRENCE L | 1 | 0.066 |
| 4946 | LAWRENCE M | 1 | 0.066 |
| 4947 | LAZAR J | 1 | 0.066 |
| 4948 | LAZAREV PV | 1 | 0.066 |
| 4949 | LAZARIDIS AA | 1 | 0.066 |
| 4950 | LE L | 1 | 0.066 |
| 4951 | LE MAY C | 1 | 0.066 |
| 4952 | LE P | 1 | 0.066 |
| 4953 | LE ROUX CW | 1 | 0.066 |
| 4954 | LEASK RL | 1 | 0.066 |
| 4955 | LEBECHE D | 1 | 0.066 |
| 4956 | LEBEK S | 1 | 0.066 |
| 4957 | LEBLANC MH | 1 | 0.066 |
| 4958 | LEBRAS MH | 1 | 0.066 |
| 4959 | LECUBE A | 1 | 0.066 |
| 4960 | LEE AS | 1 | 0.066 |
| 4961 | LEE CJ | 1 | 0.066 |
| 4962 | LEE DM | 1 | 0.066 |
| 4963 | LEE E | 1 | 0.066 |
| 4964 | LEE EJ | 1 | 0.066 |
| 4965 | LEE HA | 1 | 0.066 |
| 4966 | LEE HC | 1 | 0.066 |
| 4967 | LEE HH | 1 | 0.066 |
| 4968 | LEE JE | 1 | 0.066 |
| 4969 | LEE JH | 1 | 0.066 |
| 4970 | LEE JJ | 1 | 0.066 |
| 4971 | LEE K | 1 | 0.066 |
| 4972 | LEE MKS | 1 | 0.066 |
| 4973 | LEE MMY | 1 | 0.066 |
| 4974 | LEE MS | 1 | 0.066 |
| 4975 | LEE PC | 1 | 0.066 |
| 4976 | LEE PTH | 1 | 0.066 |
| 4977 | LEE SG | 1 | 0.066 |
| 4978 | LEE SP | 1 | 0.066 |
| 4979 | LEE TI | 1 | 0.066 |
| 4980 | LEE TM | 1 | 0.066 |
| 4981 | LEE YH | 1 | 0.066 |
| 4982 | LEE YK | 1 | 0.066 |
| 4983 | LEES K | 1 | 0.066 |
| 4984 | LEES KR | 1 | 0.066 |
| 4985 | LEESE GP | 1 | 0.066 |
| 4986 | LEHMANN R | 1 | 0.066 |
| 4987 | LEHRNER L | 1 | 0.066 |
| 4988 | LEHTIMAKI T | 1 | 0.066 |
| 4989 | LEIA C | 1 | 0.066 |
| 4990 | LEIBA A | 1 | 0.066 |
| 4991 | LEIGH P | 1 | 0.066 |
| 4992 | LEIHERER A | 1 | 0.066 |
| 4993 | LEITNER K | 1 | 0.066 |
| 4994 | LEKAKIS J | 1 | 0.066 |
| 4995 | LEMIEUX S | 1 | 0.066 |
| 4996 | LENIHAN-GEELS GN | 1 | 0.066 |
| 4997 | LENTZ J | 1 | 0.066 |
| 4998 | LEONARD CE | 1 | 0.066 |
| 4999 | LEOR J | 1 | 0.066 |
| 5000 | LEPAGE S | 1 | 0.066 |
| 5001 | LEROITH D | 1 | 0.066 |
| 5002 | LESLIE RD | 1 | 0.066 |
| 5003 | LESSINGER JM | 1 | 0.066 |
| 5004 | LETO G | 1 | 0.066 |
| 5005 | LEUNG J | 1 | 0.066 |
| 5006 | LEVASSEUR JL | 1 | 0.066 |
| 5007 | LEVCHENKO V | 1 | 0.066 |
| 5008 | LEVELT E | 1 | 0.066 |
| 5009 | LEVI M | 1 | 0.066 |
| 5010 | LEVINE JA | 1 | 0.066 |
| 5011 | LEVINSON D | 1 | 0.066 |
| 5012 | LEWIS BS | 1 | 0.066 |
| 5013 | LEWIS T | 1 | 0.066 |
| 5014 | LI B | 1 | 0.066 |
| 5015 | LI CG | 1 | 0.066 |
| 5016 | LI DD | 1 | 0.066 |
| 5017 | LI F | 1 | 0.066 |
| 5018 | LI FF | 1 | 0.066 |
| 5019 | LI GX | 1 | 0.066 |
| 5020 | LI GY | 1 | 0.066 |
| 5021 | LI H | 1 | 0.066 |
| 5022 | LI HG | 1 | 0.066 |
| 5023 | LI JF | 1 | 0.066 |
| 5024 | LI JH | 1 | 0.066 |
| 5025 | LI JP | 1 | 0.066 |
| 5026 | LI JW | 1 | 0.066 |
| 5027 | LI JY | 1 | 0.066 |
| 5028 | LI KX | 1 | 0.066 |
| 5029 | LI L | 1 | 0.066 |
| 5030 | LI M | 1 | 0.066 |
| 5031 | LI N | 1 | 0.066 |
| 5032 | LI WD | 1 | 0.066 |
| 5033 | LI XL | 1 | 0.066 |
| 5034 | LI XT | 1 | 0.066 |
| 5035 | LI YC | 1 | 0.066 |
| 5036 | LIAKOPOULOS V | 1 | 0.066 |
| 5037 | LIAMIS G | 1 | 0.066 |
| 5038 | LIANG HW | 1 | 0.066 |
| 5039 | LIANG PH | 1 | 0.066 |
| 5040 | LIANG RY | 1 | 0.066 |
| 5041 | LIANG ZR | 1 | 0.066 |
| 5042 | LIAO IC | 1 | 0.066 |
| 5043 | LIAO RX | 1 | 0.066 |
| 5044 | LIAO YN | 1 | 0.066 |
| 5045 | LIATIS S | 1 | 0.066 |
| 5046 | LIBEROPOULOS E | 1 | 0.066 |
| 5047 | LIBEROPOULOS EN | 1 | 0.066 |
| 5048 | LIBERTI ME | 1 | 0.066 |
| 5049 | LICHTENAUER M | 1 | 0.066 |
| 5050 | LIEPINSH E | 1 | 0.066 |
| 5051 | LIGHT PE | 1 | 0.066 |
| 5052 | LIGI D | 1 | 0.066 |
| 5053 | LIM K | 1 | 0.066 |
| 5054 | LIM LL | 1 | 0.066 |
| 5055 | LIM LM | 1 | 0.066 |
| 5056 | LIM TS | 1 | 0.066 |
| 5057 | LIM-ABRAHAN MA | 1 | 0.066 |
| 5058 | LIMA JC | 1 | 0.066 |
| 5059 | LIMOS A | 1 | 0.066 |
| 5060 | LIN CJ | 1 | 0.066 |
| 5061 | LIN FJ | 1 | 0.066 |
| 5062 | LIN G | 1 | 0.066 |
| 5063 | LIN HW | 1 | 0.066 |
| 5064 | LIN HYH | 1 | 0.066 |
| 5065 | LIN J | 1 | 0.066 |
| 5066 | LIN LY | 1 | 0.066 |
| 5067 | LIN SJ | 1 | 0.066 |
| 5068 | LIN SZ | 1 | 0.066 |
| 5069 | LIN TS | 1 | 0.066 |
| 5070 | LIN Y | 1 | 0.066 |
| 5071 | LIN YH | 1 | 0.066 |
| 5072 | LIN YK | 1 | 0.066 |
| 5073 | LINDBERG M | 1 | 0.066 |
| 5074 | LINDBERG SO | 1 | 0.066 |
| 5075 | LINDBLAD AJ | 1 | 0.066 |
| 5076 | LINDHOLM D | 1 | 0.066 |
| 5077 | LINDLEY R | 1 | 0.066 |
| 5078 | LINDLEY RI | 1 | 0.066 |
| 5079 | LINDSAY MC | 1 | 0.066 |
| 5080 | LINDSAY TF | 1 | 0.066 |
| 5081 | LINET T | 1 | 0.066 |
| 5082 | LING J | 1 | 0.066 |
| 5083 | LINSSEN GCM | 1 | 0.066 |
| 5084 | LINZ P | 1 | 0.066 |
| 5085 | LIONTOS A | 1 | 0.066 |
| 5086 | LIOUDAKI E | 1 | 0.066 |
| 5087 | LIP G | 1 | 0.066 |
| 5088 | LIPMAN ML | 1 | 0.066 |
| 5089 | LISOVSKAJA V | 1 | 0.066 |
| 5090 | LISS DT | 1 | 0.066 |
| 5091 | LISS K | 1 | 0.066 |
| 5092 | LIU C | 1 | 0.066 |
| 5093 | LIU DY | 1 | 0.066 |
| 5094 | LIU G | 1 | 0.066 |
| 5095 | LIU HB | 1 | 0.066 |
| 5096 | LIU MZ | 1 | 0.066 |
| 5097 | LIU PL | 1 | 0.066 |
| 5098 | LIU SB | 1 | 0.066 |
| 5099 | LIU SQ | 1 | 0.066 |
| 5100 | LIU T | 1 | 0.066 |
| 5101 | LIU XD | 1 | 0.066 |
| 5102 | LIU XK | 1 | 0.066 |
| 5103 | LIU XN | 1 | 0.066 |
| 5104 | LIU Y | 1 | 0.066 |
| 5105 | LIU YX | 1 | 0.066 |
| 5106 | LIU ZQ | 1 | 0.066 |
| 5107 | LIVINGSTON M | 1 | 0.066 |
| 5108 | LO C | 1 | 0.066 |
| 5109 | LO KB | 1 | 0.066 |
| 5110 | LOCKE A | 1 | 0.066 |
| 5111 | LOFEUDO JM | 1 | 0.066 |
| 5112 | LOGANATHAN S | 1 | 0.066 |
| 5113 | LOGUE J | 1 | 0.066 |
| 5114 | LONDON GM | 1 | 0.066 |
| 5115 | LONG Y | 1 | 0.066 |
| 5116 | LOPEZ A | 1 | 0.066 |
| 5117 | LOPEZ-JARAMILLO P | 1 | 0.066 |
| 5118 | LOSCO T | 1 | 0.066 |
| 5119 | LOU Y | 1 | 0.066 |
| 5120 | LOUGHLIN AO | 1 | 0.066 |
| 5121 | LOUTERS LL | 1 | 0.066 |
| 5122 | LOUTRADIS C | 1 | 0.066 |
| 5123 | LOVBLOM LE | 1 | 0.066 |
| 5124 | LOVRE D | 1 | 0.066 |
| 5125 | LOW S | 1 | 0.066 |
| 5126 | LU K | 1 | 0.066 |
| 5127 | LU L | 1 | 0.066 |
| 5128 | LU YP | 1 | 0.066 |
| 5129 | LU YY | 1 | 0.066 |
| 5130 | LU ZS | 1 | 0.066 |
| 5131 | LUBIAN-LOPEZ S | 1 | 0.066 |
| 5132 | LUCHNER A | 1 | 0.066 |
| 5133 | LUCHT CM | 1 | 0.066 |
| 5134 | LUCIJANIC M | 1 | 0.066 |
| 5135 | LUCISANO G | 1 | 0.066 |
| 5136 | LUEBBE ST | 1 | 0.066 |
| 5137 | LUGO A | 1 | 0.066 |
| 5138 | LUNDER M | 1 | 0.066 |
| 5139 | LUNDKVIST P | 1 | 0.066 |
| 5140 | LUNGKAPHIN A | 1 | 0.066 |
| 5141 | LUNT M | 1 | 0.066 |
| 5142 | LUO CF | 1 | 0.066 |
| 5143 | LUO Y | 1 | 0.066 |
| 5144 | LUO YH | 1 | 0.066 |
| 5145 | LUO YY | 1 | 0.066 |
| 5146 | LUO ZC | 1 | 0.066 |
| 5147 | LUPUSORU R | 1 | 0.066 |
| 5148 | LUQUE-RAMIREZ M | 1 | 0.066 |
| 5149 | LUU AZ | 1 | 0.066 |
| 5150 | LUU J | 1 | 0.066 |
| 5151 | LUU VZ | 1 | 0.066 |
| 5152 | LV QG | 1 | 0.066 |
| 5153 | LV QY | 1 | 0.066 |
| 5154 | LV WS | 1 | 0.066 |
| 5155 | LYON A | 1 | 0.066 |
| 5156 | MA JH | 1 | 0.066 |
| 5157 | MA XB | 1 | 0.066 |
| 5158 | MA XM | 1 | 0.066 |
| 5159 | MA XX | 1 | 0.066 |
| 5160 | MA YM | 1 | 0.066 |
| 5161 | MAAYAH ZH | 1 | 0.066 |
| 5162 | MABERLY GF | 1 | 0.066 |
| 5163 | MABILLARD H | 1 | 0.066 |
| 5164 | MACCHI C | 1 | 0.066 |
| 5165 | MACDONALD PS | 1 | 0.066 |
| 5166 | MACESIC H | 1 | 0.066 |
| 5167 | MACGREGOR GG | 1 | 0.066 |
| 5168 | MACHII N | 1 | 0.066 |
| 5169 | MACHINO-OHTSUKA T | 1 | 0.066 |
| 5170 | MACISAAC AI | 1 | 0.066 |
| 5171 | MACRAE CA | 1 | 0.066 |
| 5172 | MACRAE VE | 1 | 0.066 |
| 5173 | MADDUX A | 1 | 0.066 |
| 5174 | MADER A | 1 | 0.066 |
| 5175 | MADSBAD S | 1 | 0.066 |
| 5176 | MADSEN KS | 1 | 0.066 |
| 5177 | MADSEN PL | 1 | 0.066 |
| 5178 | MAEDA K | 1 | 0.066 |
| 5179 | MAEDERA S | 1 | 0.066 |
| 5180 | MAEKAWA S | 1 | 0.066 |
| 5181 | MAEMURA K | 1 | 0.066 |
| 5182 | MAENG H | 1 | 0.066 |
| 5183 | MAESHIMA A | 1 | 0.066 |
| 5184 | MAEZAWA Y | 1 | 0.066 |
| 5185 | MAFFIOLI P | 1 | 0.066 |
| 5186 | MAGNO AL | 1 | 0.066 |
| 5187 | MAHAJAN UB | 1 | 0.066 |
| 5188 | MAHBOOBI H | 1 | 0.066 |
| 5189 | MAHESHWARI H | 1 | 0.066 |
| 5190 | MAHLING M | 1 | 0.066 |
| 5191 | MAHMOUD AN | 1 | 0.066 |
| 5192 | MAHMOUD I | 1 | 0.066 |
| 5193 | MAIER L | 1 | 0.066 |
| 5194 | MAILEY J | 1 | 0.066 |
| 5195 | MAIMAITUXUN G | 1 | 0.066 |
| 5196 | MAJUMDAR U | 1 | 0.066 |
| 5197 | MAKINO A | 1 | 0.066 |
| 5198 | MAKINO H | 1 | 0.066 |
| 5199 | MAKINO Y | 1 | 0.066 |
| 5200 | MAKRECKA-KUKA M | 1 | 0.066 |
| 5201 | MALDONADO M | 1 | 0.066 |
| 5202 | MALECKI P | 1 | 0.066 |
| 5203 | MALEK R | 1 | 0.066 |
| 5204 | MALIHA G | 1 | 0.066 |
| 5205 | MALIK AH | 1 | 0.066 |
| 5206 | MALIK AO | 1 | 0.066 |
| 5207 | MALKIN SJP | 1 | 0.066 |
| 5208 | MALKOVA D | 1 | 0.066 |
| 5209 | MALLAMACI F | 1 | 0.066 |
| 5210 | MALONE M | 1 | 0.066 |
| 5211 | MALONEY A | 1 | 0.066 |
| 5212 | MALTEZOS E | 1 | 0.066 |
| 5213 | MALYSZKO J | 1 | 0.066 |
| 5214 | MAMAS MA | 1 | 0.066 |
| 5215 | MAMZA JB | 1 | 0.066 |
| 5216 | MAN NH | 1 | 0.066 |
| 5217 | MANCIA G | 1 | 0.066 |
| 5218 | MANCINI D | 1 | 0.066 |
| 5219 | MANCINI SJ | 1 | 0.066 |
| 5220 | MANCUSO J | 1 | 0.066 |
| 5221 | MANDA N | 1 | 0.066 |
| 5222 | MANDAYAM S | 1 | 0.066 |
| 5223 | MANGALI SB | 1 | 0.066 |
| 5224 | MANI H | 1 | 0.066 |
| 5225 | MANICARDI V | 1 | 0.066 |
| 5226 | MANN JFE | 1 | 0.066 |
| 5227 | MANNELLO F | 1 | 0.066 |
| 5228 | MANNINA C | 1 | 0.066 |
| 5229 | MANOLIS AA | 1 | 0.066 |
| 5230 | MANOLIS AS | 1 | 0.066 |
| 5231 | MANOLIS TA | 1 | 0.066 |
| 5232 | MANRIQUE-ACEVEDO C | 1 | 0.066 |
| 5233 | MANRIQUE-ACEVEDO CM | 1 | 0.066 |
| 5234 | MANSKI-NANKERVIS JA | 1 | 0.066 |
| 5235 | MANTZOROS CS | 1 | 0.066 |
| 5236 | MANU P | 1 | 0.066 |
| 5237 | MAR JY | 1 | 0.066 |
| 5238 | MARANGHI M | 1 | 0.066 |
| 5239 | MARAR I | 1 | 0.066 |
| 5240 | MARATHIAS KP | 1 | 0.066 |
| 5241 | MARATOU E | 1 | 0.066 |
| 5242 | MARCUS Y | 1 | 0.066 |
| 5243 | MARECHAL X | 1 | 0.066 |
| 5244 | MARESCOTTI MC | 1 | 0.066 |
| 5245 | MARGULIES K | 1 | 0.066 |
| 5246 | MARGULIES KB | 1 | 0.066 |
| 5247 | MARINA LV | 1 | 0.066 |
| 5248 | MARINE JE | 1 | 0.066 |
| 5249 | MARK M | 1 | 0.066 |
| 5250 | MARKAKIS KP | 1 | 0.066 |
| 5251 | MARKERT M | 1 | 0.066 |
| 5252 | MARRE M | 1 | 0.066 |
| 5253 | MARSHALL T | 1 | 0.066 |
| 5254 | MARTENS P | 1 | 0.066 |
| 5255 | MARTIN SS | 1 | 0.066 |
| 5256 | MARTIN V | 1 | 0.066 |
| 5257 | MARTINEZ-CEREIJO JM | 1 | 0.066 |
| 5258 | MARTINEZ-LEMUS L | 1 | 0.066 |
| 5259 | MARTINEZ-LEMUS LA | 1 | 0.066 |
| 5260 | MARTINEZ-SELLES M | 1 | 0.066 |
| 5261 | MARTINS A | 1 | 0.066 |
| 5262 | MARUHASHI T | 1 | 0.066 |
| 5263 | MARUTANI E | 1 | 0.066 |
| 5264 | MARUTHUR NM | 1 | 0.066 |
| 5265 | MARUYAMA S | 1 | 0.066 |
| 5266 | MASAJTIS-ZAGAJEWSKA A | 1 | 0.066 |
| 5267 | MASCARO D | 1 | 0.066 |
| 5268 | MASIUKIEWICZ U | 1 | 0.066 |
| 5269 | MASON T | 1 | 0.066 |
| 5270 | MASSELLI E | 1 | 0.066 |
| 5271 | MASSY Z | 1 | 0.066 |
| 5272 | MASTROCOLA R | 1 | 0.066 |
| 5273 | MATA-CASES M | 1 | 0.066 |
| 5274 | MATEOS IC | 1 | 0.066 |
| 5275 | MATHER A | 1 | 0.066 |
| 5276 | MATHER KJ | 1 | 0.066 |
| 5277 | MATOS-SOUZA JR | 1 | 0.066 |
| 5278 | MATSCHKE K | 1 | 0.066 |
| 5279 | MATSUHISA M | 1 | 0.066 |
| 5280 | MATSUKAWA M | 1 | 0.066 |
| 5281 | MATSUMOTO H | 1 | 0.066 |
| 5282 | MATSUMOTO J | 1 | 0.066 |
| 5283 | MATSUMOTO S | 1 | 0.066 |
| 5284 | MATSUMOTO Y | 1 | 0.066 |
| 5285 | MATSUMURA N | 1 | 0.066 |
| 5286 | MATSUNAGA Y | 1 | 0.066 |
| 5287 | MATSUO M | 1 | 0.066 |
| 5288 | MATSUSHITA N | 1 | 0.066 |
| 5289 | MATSUTANI D | 1 | 0.066 |
| 5290 | MATSUURA T | 1 | 0.066 |
| 5291 | MATSUZAWA Y | 1 | 0.066 |
| 5292 | MATTEAU S | 1 | 0.066 |
| 5293 | MATYJASZEK-MATUSZEK B | 1 | 0.066 |
| 5294 | MAUCK KF | 1 | 0.066 |
| 5295 | MAULION C | 1 | 0.066 |
| 5296 | MAVRAKANAS TA | 1 | 0.066 |
| 5297 | MAYER C | 1 | 0.066 |
| 5298 | MAYER GJ | 1 | 0.066 |
| 5299 | MAYER P | 1 | 0.066 |
| 5300 | MAZANOWSKA O | 1 | 0.066 |
| 5301 | MAZER-AMIRSHAHI M | 1 | 0.066 |
| 5302 | MAZIARSKA K | 1 | 0.066 |
| 5303 | MAZON P | 1 | 0.066 |
| 5304 | MAZZETTI S | 1 | 0.066 |
| 5305 | MAZZOTTI A | 1 | 0.066 |
| 5306 | MAZZUCATO M | 1 | 0.066 |
| 5307 | MBANYA JC | 1 | 0.066 |
| 5308 | MBAYE MN | 1 | 0.066 |
| 5309 | MCADAM-MARX C | 1 | 0.066 |
| 5310 | MCCALEB R | 1 | 0.066 |
| 5311 | MCCALLUM W | 1 | 0.066 |
| 5312 | MCCANN GP | 1 | 0.066 |
| 5313 | MCCARTHY DA | 1 | 0.066 |
| 5314 | MCCOY RG | 1 | 0.066 |
| 5315 | MCCRIMMON R | 1 | 0.066 |
| 5316 | MCCULLOUG PA | 1 | 0.066 |
| 5317 | MCDERMOTT MT | 1 | 0.066 |
| 5318 | MCDONALD CJ | 1 | 0.066 |
| 5319 | MCDONALD M | 1 | 0.066 |
| 5320 | MCFARLANE P | 1 | 0.066 |
| 5321 | MCFARLANE PA | 1 | 0.066 |
| 5322 | MCGRANE D | 1 | 0.066 |
| 5323 | MCGRATH K | 1 | 0.066 |
| 5324 | MCGURNAGHAN SJ | 1 | 0.066 |
| 5325 | MCHUGH KR | 1 | 0.066 |
| 5326 | MCKEIGUE PM | 1 | 0.066 |
| 5327 | MCKELVIE RS | 1 | 0.066 |
| 5328 | MCKENNA MJ | 1 | 0.066 |
| 5329 | MCKNIGHT JA | 1 | 0.066 |
| 5330 | MCLAREN J | 1 | 0.066 |
| 5331 | MCLEAN M | 1 | 0.066 |
| 5332 | MCMURRAY J | 1 | 0.066 |
| 5333 | MCMURRAY JJ | 1 | 0.066 |
| 5334 | MCMURRAYL JJV | 1 | 0.066 |
| 5335 | MEARNS ES | 1 | 0.066 |
| 5336 | MEBAZAA A | 1 | 0.066 |
| 5337 | MEERAN K | 1 | 0.066 |
| 5338 | MEGAPANOU M | 1 | 0.066 |
| 5339 | MEHANNA A | 1 | 0.066 |
| 5340 | MEHES G | 1 | 0.066 |
| 5341 | MEHTA A | 1 | 0.066 |
| 5342 | MEHTA B | 1 | 0.066 |
| 5343 | MEHTA C | 1 | 0.066 |
| 5344 | MEHTA CR | 1 | 0.066 |
| 5345 | MEHTA NN | 1 | 0.066 |
| 5346 | MEHTA PB | 1 | 0.066 |
| 5347 | MEIER C | 1 | 0.066 |
| 5348 | MELLBIN LG | 1 | 0.066 |
| 5349 | MELZER-COHEN C | 1 | 0.066 |
| 5350 | MEMON MM | 1 | 0.066 |
| 5351 | MEN P | 1 | 0.066 |
| 5352 | MENDEZ Y | 1 | 0.066 |
| 5353 | MENDOZA M | 1 | 0.066 |
| 5354 | MENDOZA ML | 1 | 0.066 |
| 5355 | MENDOZA-ZUBIETA V | 1 | 0.066 |
| 5356 | MENEGAZZO L | 1 | 0.066 |
| 5357 | MENG LP | 1 | 0.066 |
| 5358 | MENNE J | 1 | 0.066 |
| 5359 | MENNELLA R | 1 | 0.066 |
| 5360 | MENON V | 1 | 0.066 |
| 5361 | MENOWN I | 1 | 0.066 |
| 5362 | MENTIAS A | 1 | 0.066 |
| 5363 | MENZIES DJ | 1 | 0.066 |
| 5364 | MERCADO M | 1 | 0.066 |
| 5365 | MEREDITH AH | 1 | 0.066 |
| 5366 | MERKEL T | 1 | 0.066 |
| 5367 | MERKER L | 1 | 0.066 |
| 5368 | MEROVCI A | 1 | 0.066 |
| 5369 | MERTON KW | 1 | 0.066 |
| 5370 | METGE A | 1 | 0.066 |
| 5371 | METZENDORF MI | 1 | 0.066 |
| 5372 | MEYEROWITZ-KATZ G | 1 | 0.066 |
| 5373 | MIAN MR | 1 | 0.066 |
| 5374 | MICCICHE A | 1 | 0.066 |
| 5375 | MICHAILIDIS T | 1 | 0.066 |
| 5376 | MICHEL JB | 1 | 0.066 |
| 5377 | MICHEL MC | 1 | 0.066 |
| 5378 | MICHELONI S | 1 | 0.066 |
| 5379 | MICIC D | 1 | 0.066 |
| 5380 | MIDDLETON J | 1 | 0.066 |
| 5381 | MIDTVEDT K | 1 | 0.066 |
| 5382 | MIEDEMA MD | 1 | 0.066 |
| 5383 | MIELNICZUK LM | 1 | 0.066 |
| 5384 | MIFTARAJ M | 1 | 0.066 |
| 5385 | MIGNOGNA C | 1 | 0.066 |
| 5386 | MIHALICK VL | 1 | 0.066 |
| 5387 | MIHARA K | 1 | 0.066 |
| 5388 | MIKI A | 1 | 0.066 |
| 5389 | MIKLICH MA | 1 | 0.066 |
| 5390 | MILDER TY | 1 | 0.066 |
| 5391 | MILES JM | 1 | 0.066 |
| 5392 | MILICIC D | 1 | 0.066 |
| 5393 | MILINKOVIC I | 1 | 0.066 |
| 5394 | MILINOVICH A | 1 | 0.066 |
| 5395 | MILIOTIS T | 1 | 0.066 |
| 5396 | MILLER A | 1 | 0.066 |
| 5397 | MILLER EM | 1 | 0.066 |
| 5398 | MILLER M | 1 | 0.066 |
| 5399 | MILLER S | 1 | 0.066 |
| 5400 | MILLS EJ | 1 | 0.066 |
| 5401 | MIMA A | 1 | 0.066 |
| 5402 | MIN TZ | 1 | 0.066 |
| 5403 | MINANA G | 1 | 0.066 |
| 5404 | MINE T | 1 | 0.066 |
| 5405 | MINEZAKI M | 1 | 0.066 |
| 5406 | MINTZ ML | 1 | 0.066 |
| 5407 | MINUTOLO R | 1 | 0.066 |
| 5408 | MIR MU | 1 | 0.066 |
| 5409 | MIRIJELLO A | 1 | 0.066 |
| 5410 | MISHRA V | 1 | 0.066 |
| 5411 | MISTAKIDI CV | 1 | 0.066 |
| 5412 | MISUMI T | 1 | 0.066 |
| 5413 | MITTAL A | 1 | 0.066 |
| 5414 | MIURA D | 1 | 0.066 |
| 5415 | MIYA A | 1 | 0.066 |
| 5416 | MIYACHI Y | 1 | 0.066 |
| 5417 | MIYAGI M | 1 | 0.066 |
| 5418 | MIYAGI T | 1 | 0.066 |
| 5419 | MIYAMOTO F | 1 | 0.066 |
| 5420 | MIYAMOTO J | 1 | 0.066 |
| 5421 | MIYAMOTO M | 1 | 0.066 |
| 5422 | MIYATA H | 1 | 0.066 |
| 5423 | MIYAUCHI Y | 1 | 0.066 |
| 5424 | MIYAZAKI T | 1 | 0.066 |
| 5425 | MIYAZAWA Y | 1 | 0.066 |
| 5426 | MIZUMOTO K | 1 | 0.066 |
| 5427 | MIZUNO M | 1 | 0.066 |
| 5428 | MODOLO R | 1 | 0.066 |
| 5429 | MOE GW | 1 | 0.066 |
| 5430 | MOERTL D | 1 | 0.066 |
| 5431 | MOGHISSI E | 1 | 0.066 |
| 5432 | MOHAN B | 1 | 0.066 |
| 5433 | MOHSIN S | 1 | 0.066 |
| 5434 | MOK JO | 1 | 0.066 |
| 5435 | MOLINA P | 1 | 0.066 |
| 5436 | MOLINA-AYALA MA | 1 | 0.066 |
| 5437 | MOLINA-GUERRERO D | 1 | 0.066 |
| 5438 | MOLINA-VEGA M | 1 | 0.066 |
| 5439 | MOLINER P | 1 | 0.066 |
| 5440 | MOLLAR A | 1 | 0.066 |
| 5441 | MOLLER JE | 1 | 0.066 |
| 5442 | MOLLER N | 1 | 0.066 |
| 5443 | MOLOCHEK N | 1 | 0.066 |
| 5444 | MONONEN N | 1 | 0.066 |
| 5445 | MONTAIGNE D | 1 | 0.066 |
| 5446 | MONTANYA E | 1 | 0.066 |
| 5447 | MONTECUCCO F | 1 | 0.066 |
| 5448 | MONTEIRO-SOARES M | 1 | 0.066 |
| 5449 | MONTENEGRO LD | 1 | 0.066 |
| 5450 | MONTENONT E | 1 | 0.066 |
| 5451 | MONTERO M | 1 | 0.066 |
| 5452 | MONTORO-MOLINA S | 1 | 0.066 |
| 5453 | MOON EK | 1 | 0.066 |
| 5454 | MOORADIAN AD | 1 | 0.066 |
| 5455 | MORALES C | 1 | 0.066 |
| 5456 | MORALES E | 1 | 0.066 |
| 5457 | MORAN MR | 1 | 0.066 |
| 5458 | MORANO S | 1 | 0.066 |
| 5459 | MORDI I | 1 | 0.066 |
| 5460 | MORDI NA | 1 | 0.066 |
| 5461 | MORDUJOVICH J | 1 | 0.066 |
| 5462 | MOREDA R | 1 | 0.066 |
| 5463 | MOREIRA C | 1 | 0.066 |
| 5464 | MOREL O | 1 | 0.066 |
| 5465 | MORENO GYC | 1 | 0.066 |
| 5466 | MORENO P | 1 | 0.066 |
| 5467 | MORENO PJR | 1 | 0.066 |
| 5468 | MORENO-FERNANDEZ J | 1 | 0.066 |
| 5469 | MORETTI U | 1 | 0.066 |
| 5470 | MORGAN AR | 1 | 0.066 |
| 5471 | MORGAN H | 1 | 0.066 |
| 5472 | MORI T | 1 | 0.066 |
| 5473 | MORI Y | 1 | 0.066 |
| 5474 | MORIARTY PM | 1 | 0.066 |
| 5475 | MORILLAS C | 1 | 0.066 |
| 5476 | MORIMOTO C | 1 | 0.066 |
| 5477 | MORIMOTO J | 1 | 0.066 |
| 5478 | MORIMOTO S | 1 | 0.066 |
| 5479 | MORINARI M | 1 | 0.066 |
| 5480 | MORINO Y | 1 | 0.066 |
| 5481 | MORNINGSTAR J | 1 | 0.066 |
| 5482 | MORPURGO PS | 1 | 0.066 |
| 5483 | MORREN F | 1 | 0.066 |
| 5484 | MORRIS PB | 1 | 0.066 |
| 5485 | MORRISON DJ | 1 | 0.066 |
| 5486 | MORROW L | 1 | 0.066 |
| 5487 | MORTARA A | 1 | 0.066 |
| 5488 | MORTON NM | 1 | 0.066 |
| 5489 | MOSCOVITZ JE | 1 | 0.066 |
| 5490 | MOSES AC | 1 | 0.066 |
| 5491 | MOSES K | 1 | 0.066 |
| 5492 | MOSLEH W | 1 | 0.066 |
| 5493 | MOSS AS | 1 | 0.066 |
| 5494 | MOTONAGA R | 1 | 0.066 |
| 5495 | MOTTALIB A | 1 | 0.066 |
| 5496 | MOULIN P | 1 | 0.066 |
| 5497 | MOURA B | 1 | 0.066 |
| 5498 | MOURA FA | 1 | 0.066 |
| 5499 | MOURA-ASSIS A | 1 | 0.066 |
| 5500 | MOUSTAFA M | 1 | 0.066 |
| 5501 | MRZLJAK A | 1 | 0.066 |
| 5502 | MU YM | 1 | 0.066 |
| 5503 | MUELLER-WIELAND D | 1 | 0.066 |
| 5504 | MUENDLEIN A | 1 | 0.066 |
| 5505 | MUKAE T | 1 | 0.066 |
| 5506 | MUKHERJEE D | 1 | 0.066 |
| 5507 | MULLER ME | 1 | 0.066 |
| 5508 | MULLICK A | 1 | 0.066 |
| 5509 | MULLICK AE | 1 | 0.066 |
| 5510 | MUNE T | 1 | 0.066 |
| 5511 | MUNEMASA M | 1 | 0.066 |
| 5512 | MUNHOZ DB | 1 | 0.066 |
| 5513 | MUNOZ EGC | 1 | 0.066 |
| 5514 | MUNOZ-GARACH A | 1 | 0.066 |
| 5515 | MURAD MH | 1 | 0.066 |
| 5516 | MURAKAMI T | 1 | 0.066 |
| 5517 | MURALEVA NA | 1 | 0.066 |
| 5518 | MURASE K | 1 | 0.066 |
| 5519 | MURATA A | 1 | 0.066 |
| 5520 | MURATA T | 1 | 0.066 |
| 5521 | MURK W | 1 | 0.066 |
| 5522 | MURPHY B | 1 | 0.066 |
| 5523 | MUSIC ML | 1 | 0.066 |
| 5524 | MUSTAFA OG | 1 | 0.066 |
| 5525 | MUTA K | 1 | 0.066 |
| 5526 | MUTHARASAN RK | 1 | 0.066 |
| 5527 | MYAKALA K | 1 | 0.066 |
| 5528 | MYERS A | 1 | 0.066 |
| 5529 | NACI H | 1 | 0.066 |
| 5530 | NACIU AM | 1 | 0.066 |
| 5531 | NADALIN S | 1 | 0.066 |
| 5532 | NADEAU DA | 1 | 0.066 |
| 5533 | NADKARNI GN | 1 | 0.066 |
| 5534 | NADRUZ W | 1 | 0.066 |
| 5535 | NAFTALI-SHANI N | 1 | 0.066 |
| 5536 | NAGAHISA T | 1 | 0.066 |
| 5537 | NAGAI S | 1 | 0.066 |
| 5538 | NAGAISHI K | 1 | 0.066 |
| 5539 | NAGAMORI S | 1 | 0.066 |
| 5540 | NAGARAJAN VB | 1 | 0.066 |
| 5541 | NAGASHIMA K | 1 | 0.066 |
| 5542 | NAGASHIMA M | 1 | 0.066 |
| 5543 | NAGASU H | 1 | 0.066 |
| 5544 | NAGEL AM | 1 | 0.066 |
| 5545 | NAGOSHI T | 1 | 0.066 |
| 5546 | NAIDOO P | 1 | 0.066 |
| 5547 | NAING S | 1 | 0.066 |
| 5548 | NAIR A | 1 | 0.066 |
| 5549 | NAIR S | 1 | 0.066 |
| 5550 | NAIROOZ R | 1 | 0.066 |
| 5551 | NAITO R | 1 | 0.066 |
| 5552 | NAJAFZADEH M | 1 | 0.066 |
| 5553 | NAKABAYASHI T | 1 | 0.066 |
| 5554 | NAKAGAITO M | 1 | 0.066 |
| 5555 | NAKAGAWA S | 1 | 0.066 |
| 5556 | NAKAGUCHI H | 1 | 0.066 |
| 5557 | NAKAJIMA A | 1 | 0.066 |
| 5558 | NAKAJIMA K | 1 | 0.066 |
| 5559 | NAKAMURA M | 1 | 0.066 |
| 5560 | NAKANISHI N | 1 | 0.066 |
| 5561 | NAKANISHI S | 1 | 0.066 |
| 5562 | NAKANO I | 1 | 0.066 |
| 5563 | NAKATSU Y | 1 | 0.066 |
| 5564 | NAMBA S | 1 | 0.066 |
| 5565 | NAMBU H | 1 | 0.066 |
| 5566 | NANA M | 1 | 0.066 |
| 5567 | NANASATO M | 1 | 0.066 |
| 5568 | NANGAKU M | 1 | 0.066 |
| 5569 | NAPPI F | 1 | 0.066 |
| 5570 | NARANJO M | 1 | 0.066 |
| 5571 | NARAYANAN M | 1 | 0.066 |
| 5572 | NARENDRAN P | 1 | 0.066 |
| 5573 | NARKO K | 1 | 0.066 |
| 5574 | NARVARTE J | 1 | 0.066 |
| 5575 | NASHAWI M | 1 | 0.066 |
| 5576 | NASS CM | 1 | 0.066 |
| 5577 | NASSAR T | 1 | 0.066 |
| 5578 | NASSIF M | 1 | 0.066 |
| 5579 | NATALI A | 1 | 0.066 |
| 5580 | NATALICCHIO A | 1 | 0.066 |
| 5581 | NATTERO-CHAVEZ L | 1 | 0.066 |
| 5582 | NAUMAN E | 1 | 0.066 |
| 5583 | NAVANEETHAN SD | 1 | 0.066 |
| 5584 | NAVIGLIO S | 1 | 0.066 |
| 5585 | NAWATA H | 1 | 0.066 |
| 5586 | NAWAZ A | 1 | 0.066 |
| 5587 | NAYAK SK | 1 | 0.066 |
| 5588 | NEDERLOF R | 1 | 0.066 |
| 5589 | NEGISHI K | 1 | 0.066 |
| 5590 | NEHLER M | 1 | 0.066 |
| 5591 | NEHMIZ G | 1 | 0.066 |
| 5592 | NEMOTO Y | 1 | 0.066 |
| 5593 | NESLUSAN C | 1 | 0.066 |
| 5594 | NESTER W | 1 | 0.066 |
| 5595 | NESTI L | 1 | 0.066 |
| 5596 | NEVORET ML | 1 | 0.066 |
| 5597 | NEWBY DE | 1 | 0.066 |
| 5598 | NEWBY LK | 1 | 0.066 |
| 5599 | NEWLAND-JONES P | 1 | 0.066 |
| 5600 | NEWMAN G | 1 | 0.066 |
| 5601 | NEWSOME PN | 1 | 0.066 |
| 5602 | NEWTON PJ | 1 | 0.066 |
| 5603 | NG KM | 1 | 0.066 |
| 5604 | NGHIEM L | 1 | 0.066 |
| 5605 | NGUYEN E | 1 | 0.066 |
| 5606 | NGUYEN GB | 1 | 0.066 |
| 5607 | NGUYEN HM | 1 | 0.066 |
| 5608 | NICHOL B | 1 | 0.066 |
| 5609 | NICHOLLS S | 1 | 0.066 |
| 5610 | NICHOLLS SJ | 1 | 0.066 |
| 5611 | NICODEMUS NA | 1 | 0.066 |
| 5612 | NICOL P | 1 | 0.066 |
| 5613 | NICOLLE LE | 1 | 0.066 |
| 5614 | NIELSEN R | 1 | 0.066 |
| 5615 | NIETO J | 1 | 0.066 |
| 5616 | NIGAM S | 1 | 0.066 |
| 5617 | NIGRO D | 1 | 0.066 |
| 5618 | NIGRO SC | 1 | 0.066 |
| 5619 | NIHOYANNOPOULOS P | 1 | 0.066 |
| 5620 | NIIJIMA Y | 1 | 0.066 |
| 5621 | NIKITEAS N | 1 | 0.066 |
| 5622 | NIKLASSON A | 1 | 0.066 |
| 5623 | NIKOLAOU PE | 1 | 0.066 |
| 5624 | NILSSON A | 1 | 0.066 |
| 5625 | NIMAN S | 1 | 0.066 |
| 5626 | NINOMIYA T | 1 | 0.066 |
| 5627 | NIRANTHARAKUMAR K | 1 | 0.066 |
| 5628 | NISHIMURA H | 1 | 0.066 |
| 5629 | NISHIMURA K | 1 | 0.066 |
| 5630 | NISHIMURA S | 1 | 0.066 |
| 5631 | NISHIO Y | 1 | 0.066 |
| 5632 | NISHIOKA M | 1 | 0.066 |
| 5633 | NISHIZAWA M | 1 | 0.066 |
| 5634 | NISNISAN J | 1 | 0.066 |
| 5635 | NISSEN SE | 1 | 0.066 |
| 5636 | NISTALA R | 1 | 0.066 |
| 5637 | NODA M | 1 | 0.066 |
| 5638 | NODA T | 1 | 0.066 |
| 5639 | NOGUCHI M | 1 | 0.066 |
| 5640 | NOH J | 1 | 0.066 |
| 5641 | NOMIYAMA T | 1 | 0.066 |
| 5642 | NORDEN ES | 1 | 0.066 |
| 5643 | NORMAND SL | 1 | 0.066 |
| 5644 | NORONHA L | 1 | 0.066 |
| 5645 | NORTH EJ | 1 | 0.066 |
| 5646 | NOSSULI AK | 1 | 0.066 |
| 5647 | NOUTSOU M | 1 | 0.066 |
| 5648 | NOVODVORSKY P | 1 | 0.066 |
| 5649 | NOZAKI Y | 1 | 0.066 |
| 5650 | NREU B | 1 | 0.066 |
| 5651 | NUDELMAN V | 1 | 0.066 |
| 5652 | NUNEZ E | 1 | 0.066 |
| 5653 | NUNOI K | 1 | 0.066 |
| 5654 | NUTI R | 1 | 0.066 |
| 5655 | NYIRJESY P | 1 | 0.066 |
| 5656 | NYLANDER S | 1 | 0.066 |
| 5657 | NYMAN JS | 1 | 0.066 |
| 5658 | O'BRIEN MJ | 1 | 0.066 |
| 5659 | O'CONNOR C | 1 | 0.066 |
| 5660 | O'CONNOR CM | 1 | 0.066 |
| 5661 | O'HARE JP | 1 | 0.066 |
| 5662 | O'KEEFE EL | 1 | 0.066 |
| 5663 | O'KEEFE JH | 1 | 0.066 |
| 5664 | O'LOUGHLIN J | 1 | 0.066 |
| 5665 | O'NEAL D | 1 | 0.066 |
| 5666 | O'NEAL DN | 1 | 0.066 |
| 5667 | O'SHAUGHNESSY A | 1 | 0.066 |
| 5668 | O'SHEA D | 1 | 0.066 |
| 5669 | O'SHEA PM | 1 | 0.066 |
| 5670 | OBA K | 1 | 0.066 |
| 5671 | OBATA A | 1 | 0.066 |
| 5672 | OBATA Y | 1 | 0.066 |
| 5673 | OBIALO C | 1 | 0.066 |
| 5674 | OBRADOR G | 1 | 0.066 |
| 5675 | OBUNIKE J | 1 | 0.066 |
| 5676 | OCHOA-MARTINEZ FE | 1 | 0.066 |
| 5677 | ODORI S | 1 | 0.066 |
| 5678 | ODUTAYO A | 1 | 0.066 |
| 5679 | OGAWA H | 1 | 0.066 |
| 5680 | OGINO J | 1 | 0.066 |
| 5681 | OGUNNIYI M | 1 | 0.066 |
| 5682 | OH SW | 1 | 0.066 |
| 5683 | OH T | 1 | 0.066 |
| 5684 | OH TJ | 1 | 0.066 |
| 5685 | OHARA E | 1 | 0.066 |
| 5686 | OHARA K | 1 | 0.066 |
| 5687 | OHARA M | 1 | 0.066 |
| 5688 | OHASHI H | 1 | 0.066 |
| 5689 | OHASHI M | 1 | 0.066 |
| 5690 | OHATA Y | 1 | 0.066 |
| 5691 | OHBAYASHI H | 1 | 0.066 |
| 5692 | OHGAKI R | 1 | 0.066 |
| 5693 | OHISHI M | 1 | 0.066 |
| 5694 | OHKI K | 1 | 0.066 |
| 5695 | OHLMANN P | 1 | 0.066 |
| 5696 | OHNEBERG K | 1 | 0.066 |
| 5697 | OHNISHI H | 1 | 0.066 |
| 5698 | OHSAKO T | 1 | 0.066 |
| 5699 | OHTOSHI K | 1 | 0.066 |
| 5700 | OHWADA W | 1 | 0.066 |
| 5701 | OI Y | 1 | 0.066 |
| 5702 | OIKONOMOU D | 1 | 0.066 |
| 5703 | OIKONOMOU E | 1 | 0.066 |
| 5704 | OITA M | 1 | 0.066 |
| 5705 | OIWA K | 1 | 0.066 |
| 5706 | OJHA S | 1 | 0.066 |
| 5707 | OKA K | 1 | 0.066 |
| 5708 | OKA R | 1 | 0.066 |
| 5709 | OKABE M | 1 | 0.066 |
| 5710 | OKADA K | 1 | 0.066 |
| 5711 | OKADA M | 1 | 0.066 |
| 5712 | OKAHARA F | 1 | 0.066 |
| 5713 | OKAMI S | 1 | 0.066 |
| 5714 | OKANOUE T | 1 | 0.066 |
| 5715 | OKAUCHI S | 1 | 0.066 |
| 5716 | OKAWARA Y | 1 | 0.066 |
| 5717 | OKERE AN | 1 | 0.066 |
| 5718 | OKOVITYI S | 1 | 0.066 |
| 5719 | OKTAY AA | 1 | 0.066 |
| 5720 | OKUDA S | 1 | 0.066 |
| 5721 | OLAH A | 1 | 0.066 |
| 5722 | OLDGREN J | 1 | 0.066 |
| 5723 | OLELEWE S | 1 | 0.066 |
| 5724 | OLINGER AS | 1 | 0.066 |
| 5725 | OLIPHANT CS | 1 | 0.066 |
| 5726 | OLIVA RV | 1 | 0.066 |
| 5727 | OLIVER M | 1 | 0.066 |
| 5728 | OLIVER N | 1 | 0.066 |
| 5729 | OLIVERIO R | 1 | 0.066 |
| 5730 | OLIVEROS R | 1 | 0.066 |
| 5731 | OLSEN FJ | 1 | 0.066 |
| 5732 | OLSZEWSKI A | 1 | 0.066 |
| 5733 | ONISHI A | 1 | 0.066 |
| 5734 | ONISHI S | 1 | 0.066 |
| 5735 | ONMEZ A | 1 | 0.066 |
| 5736 | ONO H | 1 | 0.066 |
| 5737 | ONO J | 1 | 0.066 |
| 5738 | ONO K | 1 | 0.066 |
| 5739 | ONO M | 1 | 0.066 |
| 5740 | ONO Y | 1 | 0.066 |
| 5741 | OOHIRA T | 1 | 0.066 |
| 5742 | OPINGARI E | 1 | 0.066 |
| 5743 | ORABY MA | 1 | 0.066 |
| 5744 | ORIME K | 1 | 0.066 |
| 5745 | ORLICKY DJ | 1 | 0.066 |
| 5746 | ORLOV NB | 1 | 0.066 |
| 5747 | ORTEGA R | 1 | 0.066 |
| 5748 | ORTIZ AJS | 1 | 0.066 |
| 5749 | OSADA U | 1 | 0.066 |
| 5750 | OSAKA S | 1 | 0.066 |
| 5751 | OSAKI M | 1 | 0.066 |
| 5752 | OSAKI N | 1 | 0.066 |
| 5753 | OSAMURA A | 1 | 0.066 |
| 5754 | OSATAPHAN S | 1 | 0.066 |
| 5755 | OSBAKKEN MD | 1 | 0.066 |
| 5756 | OSHER E | 1 | 0.066 |
| 5757 | OSHIMA H | 1 | 0.066 |
| 5758 | OSHIMA M | 1 | 0.066 |
| 5759 | OSONOI T | 1 | 0.066 |
| 5760 | OSONOI Y | 1 | 0.066 |
| 5761 | OSUGA J | 1 | 0.066 |
| 5762 | OTAGAKI M | 1 | 0.066 |
| 5763 | OUCHI S | 1 | 0.066 |
| 5764 | OULHAJ A | 1 | 0.066 |
| 5765 | OUNADJELA S | 1 | 0.066 |
| 5766 | OVERBEEK J | 1 | 0.066 |
| 5767 | OZDEMIR DD | 1 | 0.066 |
| 5768 | PADILLA F | 1 | 0.066 |
| 5769 | PADILLA J | 1 | 0.066 |
| 5770 | PADRON J | 1 | 0.066 |
| 5771 | PAGE B | 1 | 0.066 |
| 5772 | PAGE SO | 1 | 0.066 |
| 5773 | PAIK JM | 1 | 0.066 |
| 5774 | PALANDRI C | 1 | 0.066 |
| 5775 | PALANIYANDI SS | 1 | 0.066 |
| 5776 | PALAU P | 1 | 0.066 |
| 5777 | PALLARDO LM | 1 | 0.066 |
| 5778 | PALMER TM | 1 | 0.066 |
| 5779 | PALOMARES R | 1 | 0.066 |
| 5780 | PALYGIN O | 1 | 0.066 |
| 5781 | PAN GD | 1 | 0.066 |
| 5782 | PAN H | 1 | 0.066 |
| 5783 | PAN HY | 1 | 0.066 |
| 5784 | PAN ML | 1 | 0.066 |
| 5785 | PAN Q | 1 | 0.066 |
| 5786 | PAN Y | 1 | 0.066 |
| 5787 | PANCER J | 1 | 0.066 |
| 5788 | PANDEY J | 1 | 0.066 |
| 5789 | PANKHANIYA R | 1 | 0.066 |
| 5790 | PANTALONE KM | 1 | 0.066 |
| 5791 | PANTANETTI P | 1 | 0.066 |
| 5792 | PAOLILLO R | 1 | 0.066 |
| 5793 | PAPADEMETRIOU M | 1 | 0.066 |
| 5794 | PAPADOPOULOS DP | 1 | 0.066 |
| 5795 | PAPADOPOULOU E | 1 | 0.066 |
| 5796 | PAPAGEORGIOU G | 1 | 0.066 |
| 5797 | PAPAGEORGIOU M | 1 | 0.066 |
| 5798 | PAPAKITSOU I | 1 | 0.066 |
| 5799 | PAPATHEODOROU K | 1 | 0.066 |
| 5800 | PAPEIX G | 1 | 0.066 |
| 5801 | PAPPACHAN JM | 1 | 0.066 |
| 5802 | PAQUOT N | 1 | 0.066 |
| 5803 | PARAJULI N | 1 | 0.066 |
| 5804 | PARAMASIVAN AM | 1 | 0.066 |
| 5805 | PARAMESH S | 1 | 0.066 |
| 5806 | PAREEK M | 1 | 0.066 |
| 5807 | PARENT MC | 1 | 0.066 |
| 5808 | PARFREY P | 1 | 0.066 |
| 5809 | PARHOFER K | 1 | 0.066 |
| 5810 | PARHOFER KG | 1 | 0.066 |
| 5811 | PARIENTE A | 1 | 0.066 |
| 5812 | PARIKH V | 1 | 0.066 |
| 5813 | PARIM B | 1 | 0.066 |
| 5814 | PARISE M | 1 | 0.066 |
| 5815 | PARK CW | 1 | 0.066 |
| 5816 | PARK D | 1 | 0.066 |
| 5817 | PARK DSJ | 1 | 0.066 |
| 5818 | PARK EG | 1 | 0.066 |
| 5819 | PARK J | 1 | 0.066 |
| 5820 | PARK JB | 1 | 0.066 |
| 5821 | PARK JJ | 1 | 0.066 |
| 5822 | PARK K | 1 | 0.066 |
| 5823 | PARK KS | 1 | 0.066 |
| 5824 | PARK YM | 1 | 0.066 |
| 5825 | PARKER JD | 1 | 0.066 |
| 5826 | PARKER R | 1 | 0.066 |
| 5827 | PARKINSON J | 1 | 0.066 |
| 5828 | PAROD ED | 1 | 0.066 |
| 5829 | PAROLINI F | 1 | 0.066 |
| 5830 | PARSON HK | 1 | 0.066 |
| 5831 | PARTRIDGE ACR | 1 | 0.066 |
| 5832 | PARVAR SL | 1 | 0.066 |
| 5833 | PASCHETTA E | 1 | 0.066 |
| 5834 | PATEL DC | 1 | 0.066 |
| 5835 | PATEL DM | 1 | 0.066 |
| 5836 | PATEL G | 1 | 0.066 |
| 5837 | PATEL K | 1 | 0.066 |
| 5838 | PATEL RS | 1 | 0.066 |
| 5839 | PATEL VH | 1 | 0.066 |
| 5840 | PATHAN SK | 1 | 0.066 |
| 5841 | PATIL CR | 1 | 0.066 |
| 5842 | PATIL KR | 1 | 0.066 |
| 5843 | PATIL PD | 1 | 0.066 |
| 5844 | PATIL SP | 1 | 0.066 |
| 5845 | PATSKO E | 1 | 0.066 |
| 5846 | PATTAR SS | 1 | 0.066 |
| 5847 | PATTERSON JH | 1 | 0.066 |
| 5848 | PATTI ME | 1 | 0.066 |
| 5849 | PATTOU F | 1 | 0.066 |
| 5850 | PAULUS WJ | 1 | 0.066 |
| 5851 | PAVLIDIS G | 1 | 0.066 |
| 5852 | PAVON H | 1 | 0.066 |
| 5853 | PAWAR A | 1 | 0.066 |
| 5854 | PEDERSEN JS | 1 | 0.066 |
| 5855 | PEDERSEN T | 1 | 0.066 |
| 5856 | PEDERSEN TR | 1 | 0.066 |
| 5857 | PEHRSON S | 1 | 0.066 |
| 5858 | PEI XB | 1 | 0.066 |
| 5859 | PEIL B | 1 | 0.066 |
| 5860 | PENCE L | 1 | 0.066 |
| 5861 | PENDSE A | 1 | 0.066 |
| 5862 | PENG XR | 1 | 0.066 |
| 5863 | PENNIG J | 1 | 0.066 |
| 5864 | PENNINGS JS | 1 | 0.066 |
| 5865 | PENNO G | 1 | 0.066 |
| 5866 | PERAZELLA MA | 1 | 0.066 |
| 5867 | PERBELLINI F | 1 | 0.066 |
| 5868 | PEREIRA TMC | 1 | 0.066 |
| 5869 | PEREZ C | 1 | 0.066 |
| 5870 | PEREZ-GOMEZ MV | 1 | 0.066 |
| 5871 | PEREZ-POLO JR | 1 | 0.066 |
| 5872 | PERI-OKONNY PA | 1 | 0.066 |
| 5873 | PERICONE CD | 1 | 0.066 |
| 5874 | PERLSTEIN I | 1 | 0.066 |
| 5875 | PEROLA M | 1 | 0.066 |
| 5876 | PERREA D | 1 | 0.066 |
| 5877 | PERRINI S | 1 | 0.066 |
| 5878 | PERRONE S | 1 | 0.066 |
| 5879 | PERROT N | 1 | 0.066 |
| 5880 | PERRY D | 1 | 0.066 |
| 5881 | PETER A | 1 | 0.066 |
| 5882 | PETKOVA R | 1 | 0.066 |
| 5883 | PETRELLI M | 1 | 0.066 |
| 5884 | PETRIE J | 1 | 0.066 |
| 5885 | PETRIE M | 1 | 0.066 |
| 5886 | PETRIZZO M | 1 | 0.066 |
| 5887 | PETRUSKI-IVLEVA N | 1 | 0.066 |
| 5888 | PETRYKIV SI | 1 | 0.066 |
| 5889 | PETTIS K | 1 | 0.066 |
| 5890 | PETTUS J | 1 | 0.066 |
| 5891 | PFEIFER M | 1 | 0.066 |
| 5892 | PFEIFER V | 1 | 0.066 |
| 5893 | PFERSCHY PN | 1 | 0.066 |
| 5894 | PFISTER O | 1 | 0.066 |
| 5895 | PHADWAL K | 1 | 0.066 |
| 5896 | PHAM D | 1 | 0.066 |
| 5897 | PHARR W | 1 | 0.066 |
| 5898 | PHENG M | 1 | 0.066 |
| 5899 | PHILLIP M | 1 | 0.066 |
| 5900 | PHILLIPS A | 1 | 0.066 |
| 5901 | PHROMMINTIKUL A | 1 | 0.066 |
| 5902 | PIAGGI P | 1 | 0.066 |
| 5903 | PIANA RN | 1 | 0.066 |
| 5904 | PICATOSTE B | 1 | 0.066 |
| 5905 | PIEBER TR | 1 | 0.066 |
| 5906 | PIEPOLI M | 1 | 0.066 |
| 5907 | PIERALICE S | 1 | 0.066 |
| 5908 | PIGNOT M | 1 | 0.066 |
| 5909 | PILMORE H | 1 | 0.066 |
| 5910 | PINA I | 1 | 0.066 |
| 5911 | PINACH S | 1 | 0.066 |
| 5912 | PINEDA ED | 1 | 0.066 |
| 5913 | PING CLS | 1 | 0.066 |
| 5914 | PINNEY SP | 1 | 0.066 |
| 5915 | PINTAT S | 1 | 0.066 |
| 5916 | PIOLI MR | 1 | 0.066 |
| 5917 | PIQUERAS L | 1 | 0.066 |
| 5918 | PIRO S | 1 | 0.066 |
| 5919 | PISCITELLI P | 1 | 0.066 |
| 5920 | PITT B | 1 | 0.066 |
| 5921 | PLEIN S | 1 | 0.066 |
| 5922 | PLOSKER G | 1 | 0.066 |
| 5923 | PLOSKER GL | 1 | 0.066 |
| 5924 | PLUTZKY J | 1 | 0.066 |
| 5925 | POGOSOVA N | 1 | 0.066 |
| 5926 | POHL EE | 1 | 0.066 |
| 5927 | POLI M | 1 | 0.066 |
| 5928 | POLIDORI D | 1 | 0.066 |
| 5929 | POLIDORO JZ | 1 | 0.066 |
| 5930 | POLIMENI A | 1 | 0.066 |
| 5931 | POLIYEDATH A | 1 | 0.066 |
| 5932 | POLLET B | 1 | 0.066 |
| 5933 | POLOVINA M | 1 | 0.066 |
| 5934 | POLOVINA MM | 1 | 0.066 |
| 5935 | POLSKY S | 1 | 0.066 |
| 5936 | POLYCHRONOPOULOS G | 1 | 0.066 |
| 5937 | POLYVIOU T | 1 | 0.066 |
| 5938 | POLYZOS SA | 1 | 0.066 |
| 5939 | POMIECZYNSKA K | 1 | 0.066 |
| 5940 | POMPEN M | 1 | 0.066 |
| 5941 | PONGCHAIDECHA A | 1 | 0.066 |
| 5942 | PONTE CD | 1 | 0.066 |
| 5943 | PONTECORVI A | 1 | 0.066 |
| 5944 | PONTOPPIDAN JRN | 1 | 0.066 |
| 5945 | POOJARY P | 1 | 0.066 |
| 5946 | POON JL | 1 | 0.066 |
| 5947 | POP LM | 1 | 0.066 |
| 5948 | POPE JE | 1 | 0.066 |
| 5949 | POPESCU I | 1 | 0.066 |
| 5950 | PORADA D | 1 | 0.066 |
| 5951 | PORFIRIS G | 1 | 0.066 |
| 5952 | PORTIANSKY EL | 1 | 0.066 |
| 5953 | PORTILLO I | 1 | 0.066 |
| 5954 | PORTOLES M | 1 | 0.066 |
| 5955 | POST A | 1 | 0.066 |
| 5956 | POSTULA M | 1 | 0.066 |
| 5957 | POULSEN HE | 1 | 0.066 |
| 5958 | POULSEN MK | 1 | 0.066 |
| 5959 | POULTER NR | 1 | 0.066 |
| 5960 | POURMAND A | 1 | 0.066 |
| 5961 | POWER T | 1 | 0.066 |
| 5962 | POZO L | 1 | 0.066 |
| 5963 | POZZI G | 1 | 0.066 |
| 5964 | PRADITPORNSILPA K | 1 | 0.066 |
| 5965 | PRASAD K | 1 | 0.066 |
| 5966 | PRASTARO M | 1 | 0.066 |
| 5967 | PRESTON T | 1 | 0.066 |
| 5968 | PREVOST G | 1 | 0.066 |
| 5969 | PRICE-HAYWOOD EG | 1 | 0.066 |
| 5970 | PROBSTFIELD J | 1 | 0.066 |
| 5971 | PROKOP LJ | 1 | 0.066 |
| 5972 | PROSKOROVSKY I | 1 | 0.066 |
| 5973 | PROSPECT TA | 1 | 0.066 |
| 5974 | PROSPERO NA | 1 | 0.066 |
| 5975 | PRUIJM M | 1 | 0.066 |
| 5976 | PRUTSKY G | 1 | 0.066 |
| 5977 | PU YL | 1 | 0.066 |
| 5978 | PUCCI A | 1 | 0.066 |
| 5979 | PUCKRIN R | 1 | 0.066 |
| 5980 | PUGSLEY MK | 1 | 0.066 |
| 5981 | PUJADES-RODRIGUEZ M | 1 | 0.066 |
| 5982 | PUN N | 1 | 0.066 |
| 5983 | PUNCHIHEWA D | 1 | 0.066 |
| 5984 | PUOLIJOKI H | 1 | 0.066 |
| 5985 | PURGA SL | 1 | 0.066 |
| 5986 | PURI R | 1 | 0.066 |
| 5987 | PURIGHALLA R | 1 | 0.066 |
| 5988 | PURRELLO F | 1 | 0.066 |
| 5989 | PURSLEY M | 1 | 0.066 |
| 5990 | PURUSHOTHAMAN A | 1 | 0.066 |
| 5991 | PYLE L | 1 | 0.066 |
| 5992 | QAMAR U | 1 | 0.066 |
| 5993 | QASEEM A | 1 | 0.066 |
| 5994 | QIAN D | 1 | 0.066 |
| 5995 | QIU HY | 1 | 0.066 |
| 5996 | QIU KF | 1 | 0.066 |
| 5997 | QUAN XC | 1 | 0.066 |
| 5998 | QUERCI F | 1 | 0.066 |
| 5999 | QUESADA-SUAREZ L | 1 | 0.066 |
| 6000 | QURESHI AW | 1 | 0.066 |
| 6001 | RABAGO G | 1 | 0.066 |
| 6002 | RABINSTEIN A | 1 | 0.066 |
| 6003 | RACKETA J | 1 | 0.066 |
| 6004 | RADDATZ D | 1 | 0.066 |
| 6005 | RADOVIC N | 1 | 0.066 |
| 6006 | RADOVITS T | 1 | 0.066 |
| 6007 | RAHMAN HU | 1 | 0.066 |
| 6008 | RAHMAN W | 1 | 0.066 |
| 6009 | RAI P | 1 | 0.066 |
| 6010 | RAJ S | 1 | 0.066 |
| 6011 | RAJADHYAKSHA V | 1 | 0.066 |
| 6012 | RAJAGOPALAN S | 1 | 0.066 |
| 6013 | RAJAKUMAR L | 1 | 0.066 |
| 6014 | RAJAN A | 1 | 0.066 |
| 6015 | RAJARATNAM R | 1 | 0.066 |
| 6016 | RAJASEKERAN H | 1 | 0.066 |
| 6017 | RAJDA M | 1 | 0.066 |
| 6018 | RAJPATHAK SN | 1 | 0.066 |
| 6019 | RAJPUT SK | 1 | 0.066 |
| 6020 | RAKOCZY PE | 1 | 0.066 |
| 6021 | RAM P | 1 | 0.066 |
| 6022 | RAMAEKERS J | 1 | 0.066 |
| 6023 | RAMAIYA K | 1 | 0.066 |
| 6024 | RAMBIRITCH V | 1 | 0.066 |
| 6025 | RAMIREZ AJ | 1 | 0.066 |
| 6026 | RAMIREZ-PEREZ FI | 1 | 0.066 |
| 6027 | RAMIREZ-RENTERIA C | 1 | 0.066 |
| 6028 | RAMJI J | 1 | 0.066 |
| 6029 | RAMON JM | 1 | 0.066 |
| 6030 | RAMOS AM | 1 | 0.066 |
| 6031 | RAMOS M | 1 | 0.066 |
| 6032 | RAMRATNAM M | 1 | 0.066 |
| 6033 | RANA K | 1 | 0.066 |
| 6034 | RANDEVA MS | 1 | 0.066 |
| 6035 | RANJAN R | 1 | 0.066 |
| 6036 | RANJBAR G | 1 | 0.066 |
| 6037 | RANKIN JM | 1 | 0.066 |
| 6038 | RAO K | 1 | 0.066 |
| 6039 | RAO SV | 1 | 0.066 |
| 6040 | RAO VS | 1 | 0.066 |
| 6041 | RAO XQ | 1 | 0.066 |
| 6042 | RAPATTONI W | 1 | 0.066 |
| 6043 | RAPTIS AE | 1 | 0.066 |
| 6044 | RASCATI KL | 1 | 0.066 |
| 6045 | RASHDAN NA | 1 | 0.066 |
| 6046 | RASKIN P | 1 | 0.066 |
| 6047 | RASSEN JA | 1 | 0.066 |
| 6048 | RASTOGI A | 1 | 0.066 |
| 6049 | RASTOGI S | 1 | 0.066 |
| 6050 | RATH L | 1 | 0.066 |
| 6051 | RATHMANN W | 1 | 0.066 |
| 6052 | RAUBENHEIMER PJ | 1 | 0.066 |
| 6053 | RAVARANI L | 1 | 0.066 |
| 6054 | RAVERDY V | 1 | 0.066 |
| 6055 | RAWASIA WF | 1 | 0.066 |
| 6056 | RAY P | 1 | 0.066 |
| 6057 | RAZAVI-NEMATOLLAHI L | 1 | 0.066 |
| 6058 | REAL J | 1 | 0.066 |
| 6059 | REAL JT | 1 | 0.066 |
| 6060 | RECTOR RS | 1 | 0.066 |
| 6061 | REDDY GB | 1 | 0.066 |
| 6062 | REDDY RPM | 1 | 0.066 |
| 6063 | REE R | 1 | 0.066 |
| 6064 | REGMI A | 1 | 0.066 |
| 6065 | REID CM | 1 | 0.066 |
| 6066 | REID J | 1 | 0.066 |
| 6067 | REIFSNIDER OS | 1 | 0.066 |
| 6068 | REINDERS J | 1 | 0.066 |
| 6069 | REITER C | 1 | 0.066 |
| 6070 | REKLOU A | 1 | 0.066 |
| 6071 | REN BL | 1 | 0.066 |
| 6072 | REN DY | 1 | 0.066 |
| 6073 | REN JH | 1 | 0.066 |
| 6074 | REN SY | 1 | 0.066 |
| 6075 | REN ZN | 1 | 0.066 |
| 6076 | RENNER A | 1 | 0.066 |
| 6077 | REQUENA-IBANEZ JA | 1 | 0.066 |
| 6078 | RESL M | 1 | 0.066 |
| 6079 | RETNAKARAN R | 1 | 0.066 |
| 6080 | REUTER HM | 1 | 0.066 |
| 6081 | REYNIER P | 1 | 0.066 |
| 6082 | REZAIE P | 1 | 0.066 |
| 6083 | RHEE BD | 1 | 0.066 |
| 6084 | RHEE EJ | 1 | 0.066 |
| 6085 | RHEE JJ | 1 | 0.066 |
| 6086 | RHOADES LD | 1 | 0.066 |
| 6087 | RIACHY R | 1 | 0.066 |
| 6088 | RIBAS HT | 1 | 0.066 |
| 6089 | RIBAULT S | 1 | 0.066 |
| 6090 | RIBEIRO AB | 1 | 0.066 |
| 6091 | RIBEIRO-VAZ I | 1 | 0.066 |
| 6092 | RICH L | 1 | 0.066 |
| 6093 | RICHELSEN B | 1 | 0.066 |
| 6094 | RICHTER B | 1 | 0.066 |
| 6095 | RIEDER T | 1 | 0.066 |
| 6096 | RIEG JAD | 1 | 0.066 |
| 6097 | RIELLO R | 1 | 0.066 |
| 6098 | RIGBY AS | 1 | 0.066 |
| 6099 | RIGBY SP | 1 | 0.066 |
| 6100 | RIGGS K | 1 | 0.066 |
| 6101 | RIKNER K | 1 | 0.066 |
| 6102 | RILEY JP | 1 | 0.066 |
| 6103 | RIM JH | 1 | 0.066 |
| 6104 | RING A | 1 | 0.066 |
| 6105 | RISKALLAH J | 1 | 0.066 |
| 6106 | RITCHIE RH | 1 | 0.066 |
| 6107 | RITTER AMV | 1 | 0.066 |
| 6108 | RIX I | 1 | 0.066 |
| 6109 | RIZOS C | 1 | 0.066 |
| 6110 | RIZOS CV | 1 | 0.066 |
| 6111 | RIZOU E | 1 | 0.066 |
| 6112 | ROBERTS FL | 1 | 0.066 |
| 6113 | ROBERTSEN I | 1 | 0.066 |
| 6114 | ROBERTSON AAB | 1 | 0.066 |
| 6115 | ROBEVA R | 1 | 0.066 |
| 6116 | ROBINSON M | 1 | 0.066 |
| 6117 | ROCCA A | 1 | 0.066 |
| 6118 | RODBARD HW | 1 | 0.066 |
| 6119 | RODDICK AJ | 1 | 0.066 |
| 6120 | RODEN M | 1 | 0.066 |
| 6121 | RODER ME | 1 | 0.066 |
| 6122 | RODGERS A | 1 | 0.066 |
| 6123 | RODRIGUEZ CJ | 1 | 0.066 |
| 6124 | RODRIGUEZ H | 1 | 0.066 |
| 6125 | RODRIGUEZ J | 1 | 0.066 |
| 6126 | RODRIGUEZ LAG | 1 | 0.066 |
| 6127 | RODRIGUEZ R | 1 | 0.066 |
| 6128 | RODRIGUEZ V | 1 | 0.066 |
| 6129 | RODRIGUEZ-MANERO M | 1 | 0.066 |
| 6130 | RODRIGUEZ-SEGADE S | 1 | 0.066 |
| 6131 | ROELL WC | 1 | 0.066 |
| 6132 | ROEVER L | 1 | 0.066 |
| 6133 | ROHWEDDER K | 1 | 0.066 |
| 6134 | ROITMAN E | 1 | 0.066 |
| 6135 | ROMA LP | 1 | 0.066 |
| 6136 | ROMERA I | 1 | 0.066 |
| 6137 | ROMERO M | 1 | 0.066 |
| 6138 | RONCO C | 1 | 0.066 |
| 6139 | RONDI K | 1 | 0.066 |
| 6140 | ROOHANI S | 1 | 0.066 |
| 6141 | RORIZ J | 1 | 0.066 |
| 6142 | ROSAS S | 1 | 0.066 |
| 6143 | ROSE JB | 1 | 0.066 |
| 6144 | ROSE P | 1 | 0.066 |
| 6145 | ROSELLO-LLETI E | 1 | 0.066 |
| 6146 | ROSENTHAL T | 1 | 0.066 |
| 6147 | ROSIAK M | 1 | 0.066 |
| 6148 | ROSIVALL L | 1 | 0.066 |
| 6149 | ROSS HJ | 1 | 0.066 |
| 6150 | ROSS JS | 1 | 0.066 |
| 6151 | ROSS M | 1 | 0.066 |
| 6152 | ROSSATO M | 1 | 0.066 |
| 6153 | ROSSI C | 1 | 0.066 |
| 6154 | ROSSI F | 1 | 0.066 |
| 6155 | ROSSI MC | 1 | 0.066 |
| 6156 | ROSSIGNOL P | 1 | 0.066 |
| 6157 | ROSSING PR | 1 | 0.066 |
| 6158 | ROTA M | 1 | 0.066 |
| 6159 | ROTELLI B | 1 | 0.066 |
| 6160 | ROTHBERG MB | 1 | 0.066 |
| 6161 | ROTHENBERG F | 1 | 0.066 |
| 6162 | ROTHENBERG P | 1 | 0.066 |
| 6163 | ROTHENBERG PL | 1 | 0.066 |
| 6164 | ROTKVIC L | 1 | 0.066 |
| 6165 | ROTKVIC PG | 1 | 0.066 |
| 6166 | ROUSSELLE T | 1 | 0.066 |
| 6167 | ROUX F | 1 | 0.066 |
| 6168 | ROVIRA-LLOPIS S | 1 | 0.066 |
| 6169 | ROYLE P | 1 | 0.066 |
| 6170 | ROZADOS-LUIS A | 1 | 0.066 |
| 6171 | RUBIN A | 1 | 0.066 |
| 6172 | RUBIN E | 1 | 0.066 |
| 6173 | RUDNICKA C | 1 | 0.066 |
| 6174 | RUED BE | 1 | 0.066 |
| 6175 | RUFFOLO A | 1 | 0.066 |
| 6176 | RUGGENENTI P | 1 | 0.066 |
| 6177 | RUGGLES JA | 1 | 0.066 |
| 6178 | RUIZ-HURTADO G | 1 | 0.066 |
| 6179 | RUIZ-ORTEGA M | 1 | 0.066 |
| 6180 | RUPPERT M | 1 | 0.066 |
| 6181 | RUSH T | 1 | 0.066 |
| 6182 | RUSSO GT | 1 | 0.066 |
| 6183 | RYAN ML | 1 | 0.066 |
| 6184 | RYAN PB | 1 | 0.066 |
| 6185 | RYAN SPP | 1 | 0.066 |
| 6186 | SA-NGUANMOO P | 1 | 0.066 |
| 6187 | SABALE U | 1 | 0.066 |
| 6188 | SABATINO J | 1 | 0.066 |
| 6189 | SABBOUR H | 1 | 0.066 |
| 6190 | SABOLIC I | 1 | 0.066 |
| 6191 | SABOO B | 1 | 0.066 |
| 6192 | SAFAR MM | 1 | 0.066 |
| 6193 | SAFHI MM | 1 | 0.066 |
| 6194 | SAG AA | 1 | 0.066 |
| 6195 | SAG CM | 1 | 0.066 |
| 6196 | SAGLIETTI G | 1 | 0.066 |
| 6197 | SAHA A | 1 | 0.066 |
| 6198 | SAHASRABUDHE V | 1 | 0.066 |
| 6199 | SAI E | 1 | 0.066 |
| 6200 | SAIFEDDINE M | 1 | 0.066 |
| 6201 | SAILER S | 1 | 0.066 |
| 6202 | SAISHO Y | 1 | 0.066 |
| 6203 | SAITO A | 1 | 0.066 |
| 6204 | SAITO M | 1 | 0.066 |
| 6205 | SAITO O | 1 | 0.066 |
| 6206 | SAKAMAKI H | 1 | 0.066 |
| 6207 | SAKAMOTO K | 1 | 0.066 |
| 6208 | SAKAMOTO M | 1 | 0.066 |
| 6209 | SAKATA Y | 1 | 0.066 |
| 6210 | SAKATANI T | 1 | 0.066 |
| 6211 | SAKODA H | 1 | 0.066 |
| 6212 | SAKUMA M | 1 | 0.066 |
| 6213 | SAKURAGI S | 1 | 0.066 |
| 6214 | SALAHUDDIN U | 1 | 0.066 |
| 6215 | SALAMUCHA I | 1 | 0.066 |
| 6216 | SALATA K | 1 | 0.066 |
| 6217 | SALES V | 1 | 0.066 |
| 6218 | SALIM HM | 1 | 0.066 |
| 6219 | SALLES JEN | 1 | 0.066 |
| 6220 | SALOMAA V | 1 | 0.066 |
| 6221 | SALT IP | 1 | 0.066 |
| 6222 | SALTEVO J | 1 | 0.066 |
| 6223 | SALTIEL MP | 1 | 0.066 |
| 6224 | SALVO F | 1 | 0.066 |
| 6225 | SAMAN S | 1 | 0.066 |
| 6226 | SAMIA EL HAYEK MM | 1 | 0.066 |
| 6227 | SAMOCHA-BONET D | 1 | 0.066 |
| 6228 | SANAANI A | 1 | 0.066 |
| 6229 | SANADA J | 1 | 0.066 |
| 6230 | SANAGORSKI R | 1 | 0.066 |
| 6231 | SANCHEZ MJ | 1 | 0.066 |
| 6232 | SANCHEZ RA | 1 | 0.066 |
| 6233 | SANCHEZ-COVISA MA | 1 | 0.066 |
| 6234 | SANCHEZ-NINO MD | 1 | 0.066 |
| 6235 | SANCHIS J | 1 | 0.066 |
| 6236 | SANDESARA PB | 1 | 0.066 |
| 6237 | SANG YQ | 1 | 0.066 |
| 6238 | SANGARALINGHAM L | 1 | 0.066 |
| 6239 | SANGO K | 1 | 0.066 |
| 6240 | SANGSHETTI JN | 1 | 0.066 |
| 6241 | SANIDAS EA | 1 | 0.066 |
| 6242 | SANKARAM R | 1 | 0.066 |
| 6243 | SANON S | 1 | 0.066 |
| 6244 | SANON VP | 1 | 0.066 |
| 6245 | SANTAMARINA M | 1 | 0.066 |
| 6246 | SANTAMARINA ML | 1 | 0.066 |
| 6247 | SANTAS E | 1 | 0.066 |
| 6248 | SANTIAGO MO | 1 | 0.066 |
| 6249 | SANTINI L | 1 | 0.066 |
| 6250 | SANTOS LL | 1 | 0.066 |
| 6251 | SANTOS-FERREIRA D | 1 | 0.066 |
| 6252 | SANZ A | 1 | 0.066 |
| 6253 | SANZ MJ | 1 | 0.066 |
| 6254 | SARAVANAN G | 1 | 0.066 |
| 6255 | SARI I | 1 | 0.066 |
| 6256 | SARIN R | 1 | 0.066 |
| 6257 | SARKAR S | 1 | 0.066 |
| 6258 | SARNAK M | 1 | 0.066 |
| 6259 | SASAKI H | 1 | 0.066 |
| 6260 | SASAKI K | 1 | 0.066 |
| 6261 | SATA Y | 1 | 0.066 |
| 6262 | SATHYAPALAN T | 1 | 0.066 |
| 6263 | SATMAN I | 1 | 0.066 |
| 6264 | SATO A | 1 | 0.066 |
| 6265 | SATO H | 1 | 0.066 |
| 6266 | SATO M | 1 | 0.066 |
| 6267 | SATO N | 1 | 0.066 |
| 6268 | SATO S | 1 | 0.066 |
| 6269 | SATOH M | 1 | 0.066 |
| 6270 | SATYAMURTHY N | 1 | 0.066 |
| 6271 | SAULSBERRY WJ | 1 | 0.066 |
| 6272 | SAUNAVAARA V | 1 | 0.066 |
| 6273 | SAUNDERS KH | 1 | 0.066 |
| 6274 | SAUR D | 1 | 0.066 |
| 6275 | SAUVANT C | 1 | 0.066 |
| 6276 | SAVARESE G | 1 | 0.066 |
| 6277 | SAWA Y | 1 | 0.066 |
| 6278 | SAWADA K | 1 | 0.066 |
| 6279 | SAWAMURA T | 1 | 0.066 |
| 6280 | SAYER JA | 1 | 0.066 |
| 6281 | SAYNISCH J | 1 | 0.066 |
| 6282 | SAYOUR AA | 1 | 0.066 |
| 6283 | SAYOUR VN | 1 | 0.066 |
| 6284 | SCAFOGLIO C | 1 | 0.066 |
| 6285 | SCAVELLI FB | 1 | 0.066 |
| 6286 | SCAVONE C | 1 | 0.066 |
| 6287 | SCERBO D | 1 | 0.066 |
| 6288 | SCHACH C | 1 | 0.066 |
| 6289 | SCHACHT S | 1 | 0.066 |
| 6290 | SCHAEFFER SE | 1 | 0.066 |
| 6291 | SCHARFSTEIN D | 1 | 0.066 |
| 6292 | SCHERNTHANER GH | 1 | 0.066 |
| 6293 | SCHERRER P | 1 | 0.066 |
| 6294 | SCHERRER-CROSBIE M | 1 | 0.066 |
| 6295 | SCHIERBEEK H | 1 | 0.066 |
| 6296 | SCHILLER A | 1 | 0.066 |
| 6297 | SCHIMMEL AWM | 1 | 0.066 |
| 6298 | SCHLAICH M | 1 | 0.066 |
| 6299 | SCHMID C | 1 | 0.066 |
| 6300 | SCHMIDT AM | 1 | 0.066 |
| 6301 | SCHMIDT BMW | 1 | 0.066 |
| 6302 | SCHMIDT M | 1 | 0.066 |
| 6303 | SCHMOOR C | 1 | 0.066 |
| 6304 | SCHNEIDER MP | 1 | 0.066 |
| 6305 | SCHOENE K | 1 | 0.066 |
| 6306 | SCHOPKA S | 1 | 0.066 |
| 6307 | SCHOUTEN EM | 1 | 0.066 |
| 6308 | SCHREIMAN R | 1 | 0.066 |
| 6309 | SCHUEMIE MJ | 1 | 0.066 |
| 6310 | SCHULMAN-MARCUS J | 1 | 0.066 |
| 6311 | SCHULZ E | 1 | 0.066 |
| 6312 | SCHUMACHER C | 1 | 0.066 |
| 6313 | SCHUMACHER M | 1 | 0.066 |
| 6314 | SCHWARTZBARD A | 1 | 0.066 |
| 6315 | SCHWEDA F | 1 | 0.066 |
| 6316 | SCIANNAMEO V | 1 | 0.066 |
| 6317 | SCIATTI E | 1 | 0.066 |
| 6318 | SCIRICA B | 1 | 0.066 |
| 6319 | SCOTT D | 1 | 0.066 |
| 6320 | SCRIPTURE JP | 1 | 0.066 |
| 6321 | SECREST MH | 1 | 0.066 |
| 6322 | SEFEROVIC J | 1 | 0.066 |
| 6323 | SEFEROVIC JP | 1 | 0.066 |
| 6324 | SEGAL JB | 1 | 0.066 |
| 6325 | SEGGELKE SA | 1 | 0.066 |
| 6326 | SEGURA AMS | 1 | 0.066 |
| 6327 | SEIDELMANN SB | 1 | 0.066 |
| 6328 | SEINO H | 1 | 0.066 |
| 6329 | SEKI T | 1 | 0.066 |
| 6330 | SEKINO H | 1 | 0.066 |
| 6331 | SEKKARIE M | 1 | 0.066 |
| 6332 | SEKO Y | 1 | 0.066 |
| 6333 | SELEJAN S | 1 | 0.066 |
| 6334 | SELLES F | 1 | 0.066 |
| 6335 | SELVARAJ S | 1 | 0.066 |
| 6336 | SELVIN E | 1 | 0.066 |
| 6337 | SEN S | 1 | 0.066 |
| 6338 | SENDA M | 1 | 0.066 |
| 6339 | SENIOR P | 1 | 0.066 |
| 6340 | SENIOR PA | 1 | 0.066 |
| 6341 | SENNEWALD R | 1 | 0.066 |
| 6342 | SENNI M | 1 | 0.066 |
| 6343 | SENSENBRENNER J | 1 | 0.066 |
| 6344 | SEO Y | 1 | 0.066 |
| 6345 | SERENELLI M | 1 | 0.066 |
| 6346 | SERLIE MJ | 1 | 0.066 |
| 6347 | SERON D | 1 | 0.066 |
| 6348 | SERONDE MF | 1 | 0.066 |
| 6349 | SERRATRICE J | 1 | 0.066 |
| 6350 | SERUSCLAT P | 1 | 0.066 |
| 6351 | SEUBERT JM | 1 | 0.066 |
| 6352 | SEWELL K | 1 | 0.066 |
| 6353 | SHAEFER CF | 1 | 0.066 |
| 6354 | SHAH A | 1 | 0.066 |
| 6355 | SHAH KB | 1 | 0.066 |
| 6356 | SHAH ND | 1 | 0.066 |
| 6357 | SHAH SJ | 1 | 0.066 |
| 6358 | SHAHINFAR S | 1 | 0.066 |
| 6359 | SHAIKH A | 1 | 0.066 |
| 6360 | SHAINBERG A | 1 | 0.066 |
| 6361 | SHAKEEL M | 1 | 0.066 |
| 6362 | SHAKIL S | 1 | 0.066 |
| 6363 | SHALHOUB V | 1 | 0.066 |
| 6364 | SHAMLIYAN TA | 1 | 0.066 |
| 6365 | SHANG QH | 1 | 0.066 |
| 6366 | SHANTIKUMAR S | 1 | 0.066 |
| 6367 | SHAO H | 1 | 0.066 |
| 6368 | SHAO QM | 1 | 0.066 |
| 6369 | SHAO YL | 1 | 0.066 |
| 6370 | SHARIFF A | 1 | 0.066 |
| 6371 | SHARMA AK | 1 | 0.066 |
| 6372 | SHARMA RK | 1 | 0.066 |
| 6373 | SHARMA SK | 1 | 0.066 |
| 6374 | SHARMA UC | 1 | 0.066 |
| 6375 | SHARP DE | 1 | 0.066 |
| 6376 | SHAW J | 1 | 0.066 |
| 6377 | SHAW JE | 1 | 0.066 |
| 6378 | SHAW S | 1 | 0.066 |
| 6379 | SHEIKH O | 1 | 0.066 |
| 6380 | SHEIKH-AHMAD M | 1 | 0.066 |
| 6381 | SHEIKH-ALI M | 1 | 0.066 |
| 6382 | SHEMBALKAR J | 1 | 0.066 |
| 6383 | SHEN HP | 1 | 0.066 |
| 6384 | SHEN L | 1 | 0.066 |
| 6385 | SHEN W | 1 | 0.066 |
| 6386 | SHEN WF | 1 | 0.066 |
| 6387 | SHENK M | 1 | 0.066 |
| 6388 | SHERMAN SE | 1 | 0.066 |
| 6389 | SHETH S | 1 | 0.066 |
| 6390 | SHETTY KK | 1 | 0.066 |
| 6391 | SHI FH | 1 | 0.066 |
| 6392 | SHI H | 1 | 0.066 |
| 6393 | SHI L | 1 | 0.066 |
| 6394 | SHI LW | 1 | 0.066 |
| 6395 | SHI Q | 1 | 0.066 |
| 6396 | SHI XJ | 1 | 0.066 |
| 6397 | SHIBA K | 1 | 0.066 |
| 6398 | SHIBAHARA T | 1 | 0.066 |
| 6399 | SHIBASAKI-KURITA T | 1 | 0.066 |
| 6400 | SHIBATA M | 1 | 0.066 |
| 6401 | SHIBATA R | 1 | 0.066 |
| 6402 | SHIBAZAKI T | 1 | 0.066 |
| 6403 | SHIBUTANI H | 1 | 0.066 |
| 6404 | SHIGENO R | 1 | 0.066 |
| 6405 | SHIH WJ | 1 | 0.066 |
| 6406 | SHIKI K | 1 | 0.066 |
| 6407 | SHIMA KR | 1 | 0.066 |
| 6408 | SHIMADA F | 1 | 0.066 |
| 6409 | SHIMADA K | 1 | 0.066 |
| 6410 | SHIMATSU A | 1 | 0.066 |
| 6411 | SHIMIZU K | 1 | 0.066 |
| 6412 | SHIMIZU M | 1 | 0.066 |
| 6413 | SHIMODA M | 1 | 0.066 |
| 6414 | SHIMOFUSA R | 1 | 0.066 |
| 6415 | SHIMONO D | 1 | 0.066 |
| 6416 | SHIMOTOYODOME A | 1 | 0.066 |
| 6417 | SHIN HM | 1 | 0.066 |
| 6418 | SHIN JI | 1 | 0.066 |
| 6419 | SHIN SJ | 1 | 0.066 |
| 6420 | SHINOHARA K | 1 | 0.066 |
| 6421 | SHINOHARA M | 1 | 0.066 |
| 6422 | SHINOZAKI M | 1 | 0.066 |
| 6423 | SHIOJIMA I | 1 | 0.066 |
| 6424 | SHIOTANI M | 1 | 0.066 |
| 6425 | SHIOU YL | 1 | 0.066 |
| 6426 | SHIRAGA N | 1 | 0.066 |
| 6427 | SHIRAISHI K | 1 | 0.066 |
| 6428 | SHIRAIWA T | 1 | 0.066 |
| 6429 | SHIRAKAWA K | 1 | 0.066 |
| 6430 | SHIRAKAWA R | 1 | 0.066 |
| 6431 | SHIRALI R | 1 | 0.066 |
| 6432 | SHIRLEY DW | 1 | 0.066 |
| 6433 | SHIROZU K | 1 | 0.066 |
| 6434 | SHIVAKUMAR O | 1 | 0.066 |
| 6435 | SHIVASWAMY V | 1 | 0.066 |
| 6436 | SHLOMAI G | 1 | 0.066 |
| 6437 | SHOJI M | 1 | 0.066 |
| 6438 | SHUKLA AP | 1 | 0.066 |
| 6439 | SHUN-SHIN MJ | 1 | 0.066 |
| 6440 | SI S | 1 | 0.066 |
| 6441 | SIAO WZ | 1 | 0.066 |
| 6442 | SICIGNANO NM | 1 | 0.066 |
| 6443 | SIDDIQI TJ | 1 | 0.066 |
| 6444 | SIDDIQUI R | 1 | 0.066 |
| 6445 | SIDHU M | 1 | 0.066 |
| 6446 | SIDHU MS | 1 | 0.066 |
| 6447 | SIEBENLIST U | 1 | 0.066 |
| 6448 | SIEGMUND T | 1 | 0.066 |
| 6449 | SIERRA J | 1 | 0.066 |
| 6450 | SIGALA T | 1 | 0.066 |
| 6451 | SIHABOUT A | 1 | 0.066 |
| 6452 | SIKIRICA S | 1 | 0.066 |
| 6453 | SILIMAN G | 1 | 0.066 |
| 6454 | SILLER-MATULA J | 1 | 0.066 |
| 6455 | SILVA-CARDOSO JC | 1 | 0.066 |
| 6456 | SILVERII GA | 1 | 0.066 |
| 6457 | SIM AM | 1 | 0.066 |
| 6458 | SIMA A | 1 | 0.066 |
| 6459 | SIMENTAL-MENDIA LE | 1 | 0.066 |
| 6460 | SIMES BC | 1 | 0.066 |
| 6461 | SIMIONI N | 1 | 0.066 |
| 6462 | SIMON BR | 1 | 0.066 |
| 6463 | SIMPSON KN | 1 | 0.066 |
| 6464 | SIMPSON RW | 1 | 0.066 |
| 6465 | SINCOULAR A | 1 | 0.066 |
| 6466 | SINDONE A | 1 | 0.066 |
| 6467 | SINGH JS | 1 | 0.066 |
| 6468 | SINGH KK | 1 | 0.066 |
| 6469 | SINGH SK | 1 | 0.066 |
| 6470 | SINGHAL G | 1 | 0.066 |
| 6471 | SINHA AD | 1 | 0.066 |
| 6472 | SIOLOS P | 1 | 0.066 |
| 6473 | SIRI-ANGKUL N | 1 | 0.066 |
| 6474 | SIRIOPOL D | 1 | 0.066 |
| 6475 | SITU B | 1 | 0.066 |
| 6476 | SIU CW | 1 | 0.066 |
| 6477 | SIVAKUMAR SM | 1 | 0.066 |
| 6478 | SIVASINPRASASN S | 1 | 0.066 |
| 6479 | SJ STR M CD | 1 | 0.066 |
| 6480 | SKOLNIK N | 1 | 0.066 |
| 6481 | SKOUMAS J | 1 | 0.066 |
| 6482 | SKRIPOVA D | 1 | 0.066 |
| 6483 | SKRTIC M | 1 | 0.066 |
| 6484 | SLAVIC S | 1 | 0.066 |
| 6485 | SLAWIK J | 1 | 0.066 |
| 6486 | SLEJKO JF | 1 | 0.066 |
| 6487 | SLUGGETT JK | 1 | 0.066 |
| 6488 | SMILDE TDJ | 1 | 0.066 |
| 6489 | SMIRNOV A | 1 | 0.066 |
| 6490 | SMITH DM | 1 | 0.066 |
| 6491 | SMITH JD | 1 | 0.066 |
| 6492 | SMITH S | 1 | 0.066 |
| 6493 | SMORODCHENKO A | 1 | 0.066 |
| 6494 | SMULDERS R | 1 | 0.066 |
| 6495 | SMYTH LR | 1 | 0.066 |
| 6496 | SNAITH J | 1 | 0.066 |
| 6497 | SNAITH JR | 1 | 0.066 |
| 6498 | SO WY | 1 | 0.066 |
| 6499 | SOARES AAS | 1 | 0.066 |
| 6500 | SOBEL JD | 1 | 0.066 |
| 6501 | SOFER Y | 1 | 0.066 |
| 6502 | SOFIA C | 1 | 0.066 |
| 6503 | SOHN DW | 1 | 0.066 |
| 6504 | SOKOLOV V | 1 | 0.066 |
| 6505 | SOLERTE SB | 1 | 0.066 |
| 6506 | SOLINSKY PJ | 1 | 0.066 |
| 6507 | SOLIS-HERRERA C | 1 | 0.066 |
| 6508 | SOLOMON R | 1 | 0.066 |
| 6509 | SON M | 1 | 0.066 |
| 6510 | SONDERGAARD AL | 1 | 0.066 |
| 6511 | SONDERGAARD E | 1 | 0.066 |
| 6512 | SONG KH | 1 | 0.066 |
| 6513 | SONG P | 1 | 0.066 |
| 6514 | SONG XH | 1 | 0.066 |
| 6515 | SONG YQ | 1 | 0.066 |
| 6516 | SONI H | 1 | 0.066 |
| 6517 | SONODA N | 1 | 0.066 |
| 6518 | SOOD V | 1 | 0.066 |
| 6519 | SORENSEN AMS | 1 | 0.066 |
| 6520 | SORENSEN MH | 1 | 0.066 |
| 6521 | SORRENTINO S | 1 | 0.066 |
| 6522 | SOSSALLA S | 1 | 0.066 |
| 6523 | SOSSALLA ST | 1 | 0.066 |
| 6524 | SOTAK M | 1 | 0.066 |
| 6525 | SOTO A | 1 | 0.066 |
| 6526 | SOTO-PEREZ M | 1 | 0.066 |
| 6527 | SPACCAROTELLA C | 1 | 0.066 |
| 6528 | SPALLONE V | 1 | 0.066 |
| 6529 | SPARTANS E | 1 | 0.066 |
| 6530 | SPERTUS JA | 1 | 0.066 |
| 6531 | SPIGONI V | 1 | 0.066 |
| 6532 | SPILLER HA | 1 | 0.066 |
| 6533 | SPINAR J | 1 | 0.066 |
| 6534 | SPINLER SA | 1 | 0.066 |
| 6535 | SPIRES D | 1 | 0.066 |
| 6536 | SPITZER H | 1 | 0.066 |
| 6537 | SPIZZO I | 1 | 0.066 |
| 6538 | SPRAGUE A | 1 | 0.066 |
| 6539 | SPRY L | 1 | 0.066 |
| 6540 | SQUIRE I | 1 | 0.066 |
| 6541 | SREENIVASAN J | 1 | 0.066 |
| 6542 | SRIDHAR VS | 1 | 0.066 |
| 6543 | SRIRAM D | 1 | 0.066 |
| 6544 | SRIRAM U | 1 | 0.066 |
| 6545 | ST ONGE E | 1 | 0.066 |
| 6546 | STABOULI S | 1 | 0.066 |
| 6547 | STACHLEWITZ RF | 1 | 0.066 |
| 6548 | STACHON P | 1 | 0.066 |
| 6549 | STAHLI BE | 1 | 0.066 |
| 6550 | STAMATOULI AM | 1 | 0.066 |
| 6551 | STAMPOULOGLOU PK | 1 | 0.066 |
| 6552 | STANG PE | 1 | 0.066 |
| 6553 | STAPFF MP | 1 | 0.066 |
| 6554 | STAPLIN N | 1 | 0.066 |
| 6555 | STARCEVIC JN | 1 | 0.066 |
| 6556 | STARUSCHENKO A | 1 | 0.066 |
| 6557 | STARZEC A | 1 | 0.066 |
| 6558 | STEEN O | 1 | 0.066 |
| 6559 | STEF NSSON BV | 1 | 0.066 |
| 6560 | STEFAN N | 1 | 0.066 |
| 6561 | STEIN P | 1 | 0.066 |
| 6562 | STEINBERG HO | 1 | 0.066 |
| 6563 | STEINER T | 1 | 0.066 |
| 6564 | STELLA P | 1 | 0.066 |
| 6565 | STELMASZYK A | 1 | 0.066 |
| 6566 | STENLOF K | 1 | 0.066 |
| 6567 | STEPHAN D | 1 | 0.066 |
| 6568 | STEPHENS JW | 1 | 0.066 |
| 6569 | STERN N | 1 | 0.066 |
| 6570 | STERNLICHT H | 1 | 0.066 |
| 6571 | STEVENS J | 1 | 0.066 |
| 6572 | STIENEN GJM | 1 | 0.066 |
| 6573 | STIRNADEL-FARRANT H | 1 | 0.066 |
| 6574 | STIRNADEL-FARRANT HA | 1 | 0.066 |
| 6575 | STIRNEMANN JM | 1 | 0.066 |
| 6576 | STOCKBRIDGE N | 1 | 0.066 |
| 6577 | STOCKER SL | 1 | 0.066 |
| 6578 | STOIAN AP | 1 | 0.066 |
| 6579 | STOKAR J | 1 | 0.066 |
| 6580 | STOMPOR T | 1 | 0.066 |
| 6581 | STORK S | 1 | 0.066 |
| 6582 | STRAIN WD | 1 | 0.066 |
| 6583 | STRECKFUSS-BOMEKE K | 1 | 0.066 |
| 6584 | STREMBITSKA A | 1 | 0.066 |
| 6585 | STRONG J | 1 | 0.066 |
| 6586 | STRUTHERS A | 1 | 0.066 |
| 6587 | STUBBS B | 1 | 0.066 |
| 6588 | STYLIANOU KG | 1 | 0.066 |
| 6589 | SU MYM | 1 | 0.066 |
| 6590 | SU XF | 1 | 0.066 |
| 6591 | SU YW | 1 | 0.066 |
| 6592 | SUAREZ A | 1 | 0.066 |
| 6593 | SUAREZ J | 1 | 0.066 |
| 6594 | SUAREZ-CUERVO C | 1 | 0.066 |
| 6595 | SUCHINDA P | 1 | 0.066 |
| 6596 | SUDA S | 1 | 0.066 |
| 6597 | SUETA D | 1 | 0.066 |
| 6598 | SUGAMORI H | 1 | 0.066 |
| 6599 | SUGANAMI H | 1 | 0.066 |
| 6600 | SUGITA Y | 1 | 0.066 |
| 6601 | SUGIYAMA Y | 1 | 0.066 |
| 6602 | SULLIVAN MA | 1 | 0.066 |
| 6603 | SULTAN S | 1 | 0.066 |
| 6604 | SUM CF | 1 | 0.066 |
| 6605 | SUMITANI S | 1 | 0.066 |
| 6606 | SUN KY | 1 | 0.066 |
| 6607 | SUN RX | 1 | 0.066 |
| 6608 | SUN VA | 1 | 0.066 |
| 6609 | SUN XY | 1 | 0.066 |
| 6610 | SUN Y | 1 | 0.066 |
| 6611 | SUNAGAWA K | 1 | 0.066 |
| 6612 | SUNDARESH V | 1 | 0.066 |
| 6613 | SUNDSTROM J | 1 | 0.066 |
| 6614 | SUNG PH | 1 | 0.066 |
| 6615 | SURAMPUDI P | 1 | 0.066 |
| 6616 | SURANI S | 1 | 0.066 |
| 6617 | SURAPANENI A | 1 | 0.066 |
| 6618 | SURYAWANSHI S | 1 | 0.066 |
| 6619 | SUSANTITAPHONG P | 1 | 0.066 |
| 6620 | SUSSEX B | 1 | 0.066 |
| 6621 | SUSSMAN S | 1 | 0.066 |
| 6622 | SUZUKI H | 1 | 0.066 |
| 6623 | SUZUKI J | 1 | 0.066 |
| 6624 | SUZUKI T | 1 | 0.066 |
| 6625 | SVAERD R | 1 | 0.066 |
| 6626 | SVART M | 1 | 0.066 |
| 6627 | SVENSON KL | 1 | 0.066 |
| 6628 | SVYSTONYUK DA | 1 | 0.066 |
| 6629 | SWART HP | 1 | 0.066 |
| 6630 | SWEENEY G | 1 | 0.066 |
| 6631 | SWIGGUM E | 1 | 0.066 |
| 6632 | SYDORENKO V | 1 | 0.066 |
| 6633 | SYKES O | 1 | 0.066 |
| 6634 | SZABO G | 1 | 0.066 |
| 6635 | SZAFRANIEC A | 1 | 0.066 |
| 6636 | TAAL MW | 1 | 0.066 |
| 6637 | TABREZ S | 1 | 0.066 |
| 6638 | TABUCHI H | 1 | 0.066 |
| 6639 | TADA H | 1 | 0.066 |
| 6640 | TAEGTMEYER H | 1 | 0.066 |
| 6641 | TAGER T | 1 | 0.066 |
| 6642 | TAHA M | 1 | 0.066 |
| 6643 | TAHARA N | 1 | 0.066 |
| 6644 | TAIRA E | 1 | 0.066 |
| 6645 | TAJEDDINE N | 1 | 0.066 |
| 6646 | TAKADA A | 1 | 0.066 |
| 6647 | TAKADA S | 1 | 0.066 |
| 6648 | TAKAGAWA Y | 1 | 0.066 |
| 6649 | TAKAHARA M | 1 | 0.066 |
| 6650 | TAKAHARA S | 1 | 0.066 |
| 6651 | TAKAISHI A | 1 | 0.066 |
| 6652 | TAKAMURA T | 1 | 0.066 |
| 6653 | TAKANO H | 1 | 0.066 |
| 6654 | TAKANO K | 1 | 0.066 |
| 6655 | TAKASE T | 1 | 0.066 |
| 6656 | TAKEDA N | 1 | 0.066 |
| 6657 | TAKEMOTO M | 1 | 0.066 |
| 6658 | TAKESHIMA T | 1 | 0.066 |
| 6659 | TAKESUE K | 1 | 0.066 |
| 6660 | TAKEUCHI J | 1 | 0.066 |
| 6661 | TAKI H | 1 | 0.066 |
| 6662 | TAKIYAMA Y | 1 | 0.066 |
| 6663 | TAKKAVATAKARN K | 1 | 0.066 |
| 6664 | TALWALKAR PG | 1 | 0.066 |
| 6665 | TAMANAHA T | 1 | 0.066 |
| 6666 | TAMBASCIA M | 1 | 0.066 |
| 6667 | TAMLER R | 1 | 0.066 |
| 6668 | TAMME L | 1 | 0.066 |
| 6669 | TAMRAKAR AK | 1 | 0.066 |
| 6670 | TAMURA K | 1 | 0.066 |
| 6671 | TAMURA M | 1 | 0.066 |
| 6672 | TAMURA Y | 1 | 0.066 |
| 6673 | TAN A | 1 | 0.066 |
| 6674 | TAN ATB | 1 | 0.066 |
| 6675 | TAN BK | 1 | 0.066 |
| 6676 | TAN KCB | 1 | 0.066 |
| 6677 | TAN M | 1 | 0.066 |
| 6678 | TAN X | 1 | 0.066 |
| 6679 | TAN XY | 1 | 0.066 |
| 6680 | TANABE J | 1 | 0.066 |
| 6681 | TANABE M | 1 | 0.066 |
| 6682 | TANAJAK P | 1 | 0.066 |
| 6683 | TANAKA TD | 1 | 0.066 |
| 6684 | TANDON M | 1 | 0.066 |
| 6685 | TANDON N | 1 | 0.066 |
| 6686 | TANENBERG RJ | 1 | 0.066 |
| 6687 | TANG F | 1 | 0.066 |
| 6688 | TANG L | 1 | 0.066 |
| 6689 | TANG SCW | 1 | 0.066 |
| 6690 | TANG W | 1 | 0.066 |
| 6691 | TANGCHAROENPAISAN Y | 1 | 0.066 |
| 6692 | TANI A | 1 | 0.066 |
| 6693 | TANK A | 1 | 0.066 |
| 6694 | TANK J | 1 | 0.066 |
| 6695 | TAOKA M | 1 | 0.066 |
| 6696 | TARA S | 1 | 0.066 |
| 6697 | TARASENKO L | 1 | 0.066 |
| 6698 | TARAZON E | 1 | 0.066 |
| 6699 | TARDIF JC | 1 | 0.066 |
| 6700 | TARNGD DC | 1 | 0.066 |
| 6701 | TARNOWSKI D | 1 | 0.066 |
| 6702 | TARQUINI G | 1 | 0.066 |
| 6703 | TASHIRO S | 1 | 0.066 |
| 6704 | TASKAEVA IS | 1 | 0.066 |
| 6705 | TAT V | 1 | 0.066 |
| 6706 | TATE M | 1 | 0.066 |
| 6707 | TATSUMI F | 1 | 0.066 |
| 6708 | TAYLOR AJ | 1 | 0.066 |
| 6709 | TAYLOR SR | 1 | 0.066 |
| 6710 | TECSON K | 1 | 0.066 |
| 6711 | TEDFORD MC | 1 | 0.066 |
| 6712 | TEERLINK J | 1 | 0.066 |
| 6713 | TEERLINK JR | 1 | 0.066 |
| 6714 | TEKES K | 1 | 0.066 |
| 6715 | TEMELKOVA-KURKTSCHIEV T | 1 | 0.066 |
| 6716 | TENENBAUM A | 1 | 0.066 |
| 6717 | TENG FY | 1 | 0.066 |
| 6718 | TENG GQ | 1 | 0.066 |
| 6719 | TEO XQ | 1 | 0.066 |
| 6720 | TERAMURA E | 1 | 0.066 |
| 6721 | TERANISHI H | 1 | 0.066 |
| 6722 | TERASAKI M | 1 | 0.066 |
| 6723 | TERAWAKI Y | 1 | 0.066 |
| 6724 | TERRACCIANO C | 1 | 0.066 |
| 6725 | TERRELONGE A | 1 | 0.066 |
| 6726 | TESSE A | 1 | 0.066 |
| 6727 | TESTANI JM | 1 | 0.066 |
| 6728 | THACKER H | 1 | 0.066 |
| 6729 | THAKKINSTIAN A | 1 | 0.066 |
| 6730 | THANIKACHALAM PV | 1 | 0.066 |
| 6731 | THANOPOULOU A | 1 | 0.066 |
| 6732 | THEODORAKOPOULOU M | 1 | 0.066 |
| 6733 | THETHI TK | 1 | 0.066 |
| 6734 | THEVERKALAM G | 1 | 0.066 |
| 6735 | THIELKE D | 1 | 0.066 |
| 6736 | THIMMAIAH R | 1 | 0.066 |
| 6737 | THOBANI A | 1 | 0.066 |
| 6738 | THOMAS GN | 1 | 0.066 |
| 6739 | THOMAS L | 1 | 0.066 |
| 6740 | THOMAS LE | 1 | 0.066 |
| 6741 | THOMAS S | 1 | 0.066 |
| 6742 | THOMOPOULOS C | 1 | 0.066 |
| 6743 | THOMPSON J | 1 | 0.066 |
| 6744 | THOMPSON M | 1 | 0.066 |
| 6745 | THOMPSON PL | 1 | 0.066 |
| 6746 | THOMSEN HH | 1 | 0.066 |
| 6747 | THONGNAK LO | 1 | 0.066 |
| 6748 | THOREN F | 1 | 0.066 |
| 6749 | THORLUND K | 1 | 0.066 |
| 6750 | THORPE KE | 1 | 0.066 |
| 6751 | THRAILKILL KM | 1 | 0.066 |
| 6752 | THUM T | 1 | 0.066 |
| 6753 | THUMMASORN S | 1 | 0.066 |
| 6754 | THURAISINGAM S | 1 | 0.066 |
| 6755 | THURBER TK | 1 | 0.066 |
| 6756 | THYMIS J | 1 | 0.066 |
| 6757 | TIAN M | 1 | 0.066 |
| 6758 | TIBALDI J | 1 | 0.066 |
| 6759 | TIBBLES LA | 1 | 0.066 |
| 6760 | TIGAS S | 1 | 0.066 |
| 6761 | TIGGELAAR S | 1 | 0.066 |
| 6762 | TIMAR B | 1 | 0.066 |
| 6763 | TIMAR R | 1 | 0.066 |
| 6764 | TIMMERMANS AD | 1 | 0.066 |
| 6765 | TINAHONES FJ | 1 | 0.066 |
| 6766 | TIPPINS F | 1 | 0.066 |
| 6767 | TIRILOMIS P | 1 | 0.066 |
| 6768 | TISEO G | 1 | 0.066 |
| 6769 | TITZE J | 1 | 0.066 |
| 6770 | TITZE JM | 1 | 0.066 |
| 6771 | TOBE K | 1 | 0.066 |
| 6772 | TOBIUME T | 1 | 0.066 |
| 6773 | TOCHIYA M | 1 | 0.066 |
| 6774 | TOGANE M | 1 | 0.066 |
| 6775 | TOGLIATTO G | 1 | 0.066 |
| 6776 | TOGO M | 1 | 0.066 |
| 6777 | TOKGOZOGLU L | 1 | 0.066 |
| 6778 | TOKITA Y | 1 | 0.066 |
| 6779 | TOKUDA K | 1 | 0.066 |
| 6780 | TOKUSHIGE K | 1 | 0.066 |
| 6781 | TOKUYAMA H | 1 | 0.066 |
| 6782 | TOLBOD LP | 1 | 0.066 |
| 6783 | TOLEDANO Y | 1 | 0.066 |
| 6784 | TOMA M | 1 | 0.066 |
| 6785 | TOMAE M | 1 | 0.066 |
| 6786 | TOMASONI D | 1 | 0.066 |
| 6787 | TOMBERLI B | 1 | 0.066 |
| 6788 | TOMITA T | 1 | 0.066 |
| 6789 | TOMOYASU M | 1 | 0.066 |
| 6790 | TON J | 1 | 0.066 |
| 6791 | TONG LL | 1 | 0.066 |
| 6792 | TONG PCY | 1 | 0.066 |
| 6793 | TONNEIJCK L | 1 | 0.066 |
| 6794 | TOOR K | 1 | 0.066 |
| 6795 | TORAL M | 1 | 0.066 |
| 6796 | TORIMURA T | 1 | 0.066 |
| 6797 | TOTH A | 1 | 0.066 |
| 6798 | TOTI F | 1 | 0.066 |
| 6799 | TOTO R | 1 | 0.066 |
| 6800 | TOULIS KA | 1 | 0.066 |
| 6801 | TOUMANIANTZ G | 1 | 0.066 |
| 6802 | TOYODA S | 1 | 0.066 |
| 6803 | TRAINA S | 1 | 0.066 |
| 6804 | TRAN D | 1 | 0.066 |
| 6805 | TRAN NQ | 1 | 0.066 |
| 6806 | TRAVIS K | 1 | 0.066 |
| 6807 | TRENCHE SM | 1 | 0.066 |
| 6808 | TRENCSENYI G | 1 | 0.066 |
| 6809 | TREPPENDAHL MB | 1 | 0.066 |
| 6810 | TRESCOI C | 1 | 0.066 |
| 6811 | TRESPALACIOS F | 1 | 0.066 |
| 6812 | TRIBOLETTI JS | 1 | 0.066 |
| 6813 | TRIKHA J | 1 | 0.066 |
| 6814 | TRIKHA SRJ | 1 | 0.066 |
| 6815 | TRIMARCO B | 1 | 0.066 |
| 6816 | TRIPATHY D | 1 | 0.066 |
| 6817 | TRIPOLT NJ | 1 | 0.066 |
| 6818 | TRIPOSKIADIS F | 1 | 0.066 |
| 6819 | TRIPPE B | 1 | 0.066 |
| 6820 | TRUEBA P | 1 | 0.066 |
| 6821 | TRUJILLO A | 1 | 0.066 |
| 6822 | TSCHOPE D | 1 | 0.066 |
| 6823 | TSE G | 1 | 0.066 |
| 6824 | TSE HF | 1 | 0.066 |
| 6825 | TSENG E | 1 | 0.066 |
| 6826 | TSILINGIRIS D | 1 | 0.066 |
| 6827 | TSIVGOULIS G | 1 | 0.066 |
| 6828 | TSOI MF | 1 | 0.066 |
| 6829 | TSOUKAS MA | 1 | 0.066 |
| 6830 | TSUCHIDA A | 1 | 0.066 |
| 6831 | TSUCHIYA K | 1 | 0.066 |
| 6832 | TSUGAMI E | 1 | 0.066 |
| 6833 | TSUGAWA M | 1 | 0.066 |
| 6834 | TSUI HCT | 1 | 0.066 |
| 6835 | TSUKADA Y | 1 | 0.066 |
| 6836 | TSUKAMOTO K | 1 | 0.066 |
| 6837 | TSUMURA N | 1 | 0.066 |
| 6838 | TSURUYA K | 1 | 0.066 |
| 6839 | TSUTSUMI Y | 1 | 0.066 |
| 6840 | TUCCINARDI D | 1 | 0.066 |
| 6841 | TUCCINARDI F | 1 | 0.066 |
| 6842 | TUNCAY E | 1 | 0.066 |
| 6843 | TUNCELI O | 1 | 0.066 |
| 6844 | TUNE JD | 1 | 0.066 |
| 6845 | TUOMILEHTO J | 1 | 0.066 |
| 6846 | TURCHIN A | 1 | 0.066 |
| 6847 | TURNBULL JD | 1 | 0.066 |
| 6848 | TURNER JR | 1 | 0.066 |
| 6849 | TUXEN C | 1 | 0.066 |
| 6850 | TWAHIRWA M | 1 | 0.066 |
| 6851 | TWIG G | 1 | 0.066 |
| 6852 | TWILLA JD | 1 | 0.066 |
| 6853 | TZAVELA E | 1 | 0.066 |
| 6854 | TZAVELLA E | 1 | 0.066 |
| 6855 | UBUKATA M | 1 | 0.066 |
| 6856 | UCHIDA D | 1 | 0.066 |
| 6857 | UCHIDA S | 1 | 0.066 |
| 6858 | UCHINO H | 1 | 0.066 |
| 6859 | UCHIYAMA K | 1 | 0.066 |
| 6860 | UDDANDRAO VVS | 1 | 0.066 |
| 6861 | UDER M | 1 | 0.066 |
| 6862 | UEDA K | 1 | 0.066 |
| 6863 | UEMATSU M | 1 | 0.066 |
| 6864 | UENO M | 1 | 0.066 |
| 6865 | UETA K | 1 | 0.066 |
| 6866 | UEYAMA E | 1 | 0.066 |
| 6867 | UHRIG JL | 1 | 0.066 |
| 6868 | UKENA C | 1 | 0.066 |
| 6869 | UKENA JK | 1 | 0.066 |
| 6870 | UM J | 1 | 0.066 |
| 6871 | UMAYAHARA Y | 1 | 0.066 |
| 6872 | UMEI T | 1 | 0.066 |
| 6873 | UMPIERREZ GE | 1 | 0.066 |
| 6874 | UNDAVALLI C | 1 | 0.066 |
| 6875 | UNDERBERG JA | 1 | 0.066 |
| 6876 | UNE HD | 1 | 0.066 |
| 6877 | UNGER J | 1 | 0.066 |
| 6878 | UNNIKRISHNAN AG | 1 | 0.066 |
| 6879 | UNO S | 1 | 0.066 |
| 6880 | UNOSAWA S | 1 | 0.066 |
| 6881 | UNWIN D | 1 | 0.066 |
| 6882 | UPDEGROVE J | 1 | 0.066 |
| 6883 | UPPUGANTI S | 1 | 0.066 |
| 6884 | URATA H | 1 | 0.066 |
| 6885 | URBANEK K | 1 | 0.066 |
| 6886 | USHIJIMA R | 1 | 0.066 |
| 6887 | USMAN MS | 1 | 0.066 |
| 6888 | USUKURA M | 1 | 0.066 |
| 6889 | UTHMAN O | 1 | 0.066 |
| 6890 | UZUI H | 1 | 0.066 |
| 6891 | VAAG A | 1 | 0.066 |
| 6892 | VACCARELLA M | 1 | 0.066 |
| 6893 | VAIDYANATHAN S | 1 | 0.066 |
| 6894 | VALDIVIELSO J | 1 | 0.066 |
| 6895 | VALENCIA WM | 1 | 0.066 |
| 6896 | VALENTINE V | 1 | 0.066 |
| 6897 | VAN BLOEMENDAAL L | 1 | 0.066 |
| 6898 | VAN BUREN P | 1 | 0.066 |
| 6899 | VAN ECK JWM | 1 | 0.066 |
| 6900 | VAN GAAL LF | 1 | 0.066 |
| 6901 | VAN HAALEN HGM | 1 | 0.066 |
| 6902 | VAN HARSKAMP D | 1 | 0.066 |
| 6903 | VAN LINTHOUT S | 1 | 0.066 |
| 6904 | VAN RUITEN CC | 1 | 0.066 |
| 6905 | VAN STEENBERGEN A | 1 | 0.066 |
| 6906 | VAN TONGEREN S | 1 | 0.066 |
| 6907 | VANI AK | 1 | 0.066 |
| 6908 | VANNORMAN C | 1 | 0.066 |
| 6909 | VANNORSDALL M | 1 | 0.066 |
| 6910 | VANOVERSCHELDE JL | 1 | 0.066 |
| 6911 | VARELA A | 1 | 0.066 |
| 6912 | VARGAS F | 1 | 0.066 |
| 6913 | VARGAS-DELGADO AP | 1 | 0.066 |
| 6914 | VARGHESE F | 1 | 0.066 |
| 6915 | VASILAKOU D | 1 | 0.066 |
| 6916 | VASILUTA L | 1 | 0.066 |
| 6917 | VAZELOV E | 1 | 0.066 |
| 6918 | VECCHIE A | 1 | 0.066 |
| 6919 | VED JK | 1 | 0.066 |
| 6920 | VEDIN O | 1 | 0.066 |
| 6921 | VELASQUEZ FC | 1 | 0.066 |
| 6922 | VELASQUEZ-MIEYER P | 1 | 0.066 |
| 6923 | VELLANKI P | 1 | 0.066 |
| 6924 | VELLIOU M | 1 | 0.066 |
| 6925 | VELLOSO LA | 1 | 0.066 |
| 6926 | VENDELBO MH | 1 | 0.066 |
| 6927 | VENTRAPRAGADA S | 1 | 0.066 |
| 6928 | VENU VKP | 1 | 0.066 |
| 6929 | VERA-NUNEZ M | 1 | 0.066 |
| 6930 | VERGES B | 1 | 0.066 |
| 6931 | VERHEYEN N | 1 | 0.066 |
| 6932 | VERMA V | 1 | 0.066 |
| 6933 | VESTERGAARD P | 1 | 0.066 |
| 6934 | VICTOR VM | 1 | 0.066 |
| 6935 | VIDEBAEK L | 1 | 0.066 |
| 6936 | VIDEJA M | 1 | 0.066 |
| 6937 | VILKS K | 1 | 0.066 |
| 6938 | VILLEVALDE SV | 1 | 0.066 |
| 6939 | VINCI C | 1 | 0.066 |
| 6940 | VINIK AI | 1 | 0.066 |
| 6941 | VINKE JSJ | 1 | 0.066 |
| 6942 | VINOVSKIS CL | 1 | 0.066 |
| 6943 | VIRANI SA | 1 | 0.066 |
| 6944 | VIRGINIO VW | 1 | 0.066 |
| 6945 | VIROVIC-JUKIC L | 1 | 0.066 |
| 6946 | VISRAM A | 1 | 0.066 |
| 6947 | VISWANATH AK | 1 | 0.066 |
| 6948 | VITALE C | 1 | 0.066 |
| 6949 | VITARELLI G | 1 | 0.066 |
| 6950 | VITOLO E | 1 | 0.066 |
| 6951 | VITTURI N | 1 | 0.066 |
| 6952 | VLACHAKIS P | 1 | 0.066 |
| 6953 | VLACHOPOULOS C | 1 | 0.066 |
| 6954 | VLACO B | 1 | 0.066 |
| 6955 | VLAD A | 1 | 0.066 |
| 6956 | VLAHAKOS DV | 1 | 0.066 |
| 6957 | VLAHAKOS VD | 1 | 0.066 |
| 6958 | VLOTIDES G | 1 | 0.066 |
| 6959 | VO C | 1 | 0.066 |
| 6960 | VOELKER J | 1 | 0.066 |
| 6961 | VOGIATZI G | 1 | 0.066 |
| 6962 | VOLPE M | 1 | 0.066 |
| 6963 | VOLSI PL | 1 | 0.066 |
| 6964 | VON INGERSLEBEN G | 1 | 0.066 |
| 6965 | VON LUEDER TG | 1 | 0.066 |
| 6966 | VON SCHOLTEN BJ | 1 | 0.066 |
| 6967 | VONBANK A | 1 | 0.066 |
| 6968 | VOS D | 1 | 0.066 |
| 6969 | VOSSELER A | 1 | 0.066 |
| 6970 | VOURI SM | 1 | 0.066 |
| 6971 | VRHOVAC I | 1 | 0.066 |
| 6972 | VUJACIC-MIRSKI K | 1 | 0.066 |
| 6973 | VUKOTIC G | 1 | 0.066 |
| 6974 | VYAS M | 1 | 0.066 |
| 6975 | WADA F | 1 | 0.066 |
| 6976 | WADA J | 1 | 0.066 |
| 6977 | WADUD K | 1 | 0.066 |
| 6978 | WAGEMANN O | 1 | 0.066 |
| 6979 | WAI M | 1 | 0.066 |
| 6980 | WAKATSUKI T | 1 | 0.066 |
| 6981 | WAKAYAMA A | 1 | 0.066 |
| 6982 | WAKISAKA M | 1 | 0.066 |
| 6983 | WAKUI H | 1 | 0.066 |
| 6984 | WALDMAN M | 1 | 0.066 |
| 6985 | WALLACE CG | 1 | 0.066 |
| 6986 | WALLES T | 1 | 0.066 |
| 6987 | WALLIA A | 1 | 0.066 |
| 6988 | WALROTH T | 1 | 0.066 |
| 6989 | WALTER B | 1 | 0.066 |
| 6990 | WALTER MF | 1 | 0.066 |
| 6991 | WALTON MK | 1 | 0.066 |
| 6992 | WANCHAI K | 1 | 0.066 |
| 6993 | WANG A | 1 | 0.066 |
| 6994 | WANG BH | 1 | 0.066 |
| 6995 | WANG CCL | 1 | 0.066 |
| 6996 | WANG D | 1 | 0.066 |
| 6997 | WANG DF | 1 | 0.066 |
| 6998 | WANG E | 1 | 0.066 |
| 6999 | WANG F | 1 | 0.066 |
| 7000 | WANG H | 1 | 0.066 |
| 7001 | WANG HJ | 1 | 0.066 |
| 7002 | WANG HL | 1 | 0.066 |
| 7003 | WANG JB | 1 | 0.066 |
| 7004 | WANG KL | 1 | 0.066 |
| 7005 | WANG L | 1 | 0.066 |
| 7006 | WANG LF | 1 | 0.066 |
| 7007 | WANG P | 1 | 0.066 |
| 7008 | WANG PS | 1 | 0.066 |
| 7009 | WANG Q | 1 | 0.066 |
| 7010 | WANG S | 1 | 0.066 |
| 7011 | WANG SB | 1 | 0.066 |
| 7012 | WANG SY | 1 | 0.066 |
| 7013 | WANG TT | 1 | 0.066 |
| 7014 | WANG WH | 1 | 0.066 |
| 7015 | WANG WT | 1 | 0.066 |
| 7016 | WANG WX | 1 | 0.066 |
| 7017 | WANG XQ | 1 | 0.066 |
| 7018 | WANG XX | 1 | 0.066 |
| 7019 | WANG YB | 1 | 0.066 |
| 7020 | WANG YF | 1 | 0.066 |
| 7021 | WANG YL | 1 | 0.066 |
| 7022 | WANG Z | 1 | 0.066 |
| 7023 | WANG ZJ | 1 | 0.066 |
| 7024 | WANGENSTEEN R | 1 | 0.066 |
| 7025 | WARD GM | 1 | 0.066 |
| 7026 | WARD MS | 1 | 0.066 |
| 7027 | WARDEN BA | 1 | 0.066 |
| 7028 | WARNOCK A | 1 | 0.066 |
| 7029 | WASCHER TC | 1 | 0.066 |
| 7030 | WATANABE M | 1 | 0.066 |
| 7031 | WATANABE S | 1 | 0.066 |
| 7032 | WATSON AMD | 1 | 0.066 |
| 7033 | WATSON H | 1 | 0.066 |
| 7034 | WATSON J | 1 | 0.066 |
| 7035 | WATSON K | 1 | 0.066 |
| 7036 | WATSON KE | 1 | 0.066 |
| 7037 | WATTS GF | 1 | 0.066 |
| 7038 | WATTS R | 1 | 0.066 |
| 7039 | WAUGH N | 1 | 0.066 |
| 7040 | WAZIR A | 1 | 0.066 |
| 7041 | WEBER MA | 1 | 0.066 |
| 7042 | WEBER SL | 1 | 0.066 |
| 7043 | WEI L | 1 | 0.066 |
| 7044 | WEI X | 1 | 0.066 |
| 7045 | WEIDINGER F | 1 | 0.066 |
| 7046 | WEIDNER-WELLS M | 1 | 0.066 |
| 7047 | WEINER D | 1 | 0.066 |
| 7048 | WEINSTEIN JJ | 1 | 0.066 |
| 7049 | WEINTRAUB H | 1 | 0.066 |
| 7050 | WEINTRAUB HS | 1 | 0.066 |
| 7051 | WEIR TL | 1 | 0.066 |
| 7052 | WEISMAN H | 1 | 0.066 |
| 7053 | WEISS MC | 1 | 0.066 |
| 7054 | WELKER J | 1 | 0.066 |
| 7055 | WELLS S | 1 | 0.066 |
| 7056 | WELSCHOF P | 1 | 0.066 |
| 7057 | WELSH J | 1 | 0.066 |
| 7058 | WELTY F | 1 | 0.066 |
| 7059 | WELTY FK | 1 | 0.066 |
| 7060 | WEN XY | 1 | 0.066 |
| 7061 | WENG W | 1 | 0.066 |
| 7062 | WENGER N | 1 | 0.066 |
| 7063 | WENZEL P | 1 | 0.066 |
| 7064 | WERNER C | 1 | 0.066 |
| 7065 | WESOLOWSKA A | 1 | 0.066 |
| 7066 | WESTEIN E | 1 | 0.066 |
| 7067 | WETTERGREEN SA | 1 | 0.066 |
| 7068 | WHALEN K | 1 | 0.066 |
| 7069 | WHILLAS A | 1 | 0.066 |
| 7070 | WHITE CM | 1 | 0.066 |
| 7071 | WHITE J | 1 | 0.066 |
| 7072 | WHITTAKER CF | 1 | 0.066 |
| 7073 | WHYTE M | 1 | 0.066 |
| 7074 | WIBERG M | 1 | 0.066 |
| 7075 | WIDDOP RE | 1 | 0.066 |
| 7076 | WIGGINS BS | 1 | 0.066 |
| 7077 | WILCOX T | 1 | 0.066 |
| 7078 | WILD SH | 1 | 0.066 |
| 7079 | WILLECKE F | 1 | 0.066 |
| 7080 | WILLIAMS DM | 1 | 0.066 |
| 7081 | WILLIAMS DR | 1 | 0.066 |
| 7082 | WILLIAMS FB | 1 | 0.066 |
| 7083 | WILLIAMS S | 1 | 0.066 |
| 7084 | WILLIAMS SM | 1 | 0.066 |
| 7085 | WILLIS BH | 1 | 0.066 |
| 7086 | WILLIS M | 1 | 0.066 |
| 7087 | WILPSHAAR W | 1 | 0.066 |
| 7088 | WILSON LM | 1 | 0.066 |
| 7089 | WINDSOR SL | 1 | 0.066 |
| 7090 | WINKLER ME | 1 | 0.066 |
| 7091 | WINTRICH J | 1 | 0.066 |
| 7092 | WISSMAN NK | 1 | 0.066 |
| 7093 | WITKOWSKA A | 1 | 0.066 |
| 7094 | WITTWER JA | 1 | 0.066 |
| 7095 | WOJCIK C | 1 | 0.066 |
| 7096 | WOLF D | 1 | 0.066 |
| 7097 | WOLF VLW | 1 | 0.066 |
| 7098 | WOLSK E | 1 | 0.066 |
| 7099 | WON JC | 1 | 0.066 |
| 7100 | WONG J | 1 | 0.066 |
| 7101 | WONG ND | 1 | 0.066 |
| 7102 | WONG YF | 1 | 0.066 |
| 7103 | WONGCHAROEN W | 1 | 0.066 |
| 7104 | WOO CC | 1 | 0.066 |
| 7105 | WOO VC | 1 | 0.066 |
| 7106 | WOOD D | 1 | 0.066 |
| 7107 | WRIGHT EC | 1 | 0.066 |
| 7108 | WRIGHT EE | 1 | 0.066 |
| 7109 | WRIGHT LA | 1 | 0.066 |
| 7110 | WU BS | 1 | 0.066 |
| 7111 | WU J | 1 | 0.066 |
| 7112 | WU JD | 1 | 0.066 |
| 7113 | WU JJ | 1 | 0.066 |
| 7114 | WU JY | 1 | 0.066 |
| 7115 | WU L | 1 | 0.066 |
| 7116 | WU LD | 1 | 0.066 |
| 7117 | WU N | 1 | 0.066 |
| 7118 | WU Q | 1 | 0.066 |
| 7119 | WU SJ | 1 | 0.066 |
| 7120 | WU SS | 1 | 0.066 |
| 7121 | WU W | 1 | 0.066 |
| 7122 | WU YY | 1 | 0.066 |
| 7123 | WUST RCI | 1 | 0.066 |
| 7124 | WYSHAM CH | 1 | 0.066 |
| 7125 | XANTHOPOULOS A | 1 | 0.066 |
| 7126 | XIA C | 1 | 0.066 |
| 7127 | XIA CM | 1 | 0.066 |
| 7128 | XIA N | 1 | 0.066 |
| 7129 | XIANG JM | 1 | 0.066 |
| 7130 | XIANG YK | 1 | 0.066 |
| 7131 | XIAO HP | 1 | 0.066 |
| 7132 | XIAO PG | 1 | 0.066 |
| 7133 | XIARCHOU A | 1 | 0.066 |
| 7134 | XIE JM | 1 | 0.066 |
| 7135 | XING BD | 1 | 0.066 |
| 7136 | XIONG J | 1 | 0.066 |
| 7137 | XIONG SQ | 1 | 0.066 |
| 7138 | XIU P | 1 | 0.066 |
| 7139 | XU AM | 1 | 0.066 |
| 7140 | XU B | 1 | 0.066 |
| 7141 | XU GD | 1 | 0.066 |
| 7142 | XU N | 1 | 0.066 |
| 7143 | XU X | 1 | 0.066 |
| 7144 | XU ZJ | 1 | 0.066 |
| 7145 | YACOUB T | 1 | 0.066 |
| 7146 | YADIN D | 1 | 0.066 |
| 7147 | YAGI K | 1 | 0.066 |
| 7148 | YAHYA A | 1 | 0.066 |
| 7149 | YAKOVLEVA T | 1 | 0.066 |
| 7150 | YALCIN MU | 1 | 0.066 |
| 7151 | YALE JF | 1 | 0.066 |
| 7152 | YAMADA K | 1 | 0.066 |
| 7153 | YAMAGA M | 1 | 0.066 |
| 7154 | YAMAGISHI S | 1 | 0.066 |
| 7155 | YAMAGISHI SI | 1 | 0.066 |
| 7156 | YAMAGUCHI K | 1 | 0.066 |
| 7157 | YAMAKAGE H | 1 | 0.066 |
| 7158 | YAMAKAWA T | 1 | 0.066 |
| 7159 | YAMAMOTO M | 1 | 0.066 |
| 7160 | YAMAMOTO S | 1 | 0.066 |
| 7161 | YAMAMOTO Y | 1 | 0.066 |
| 7162 | YAMAMOTOYA T | 1 | 0.066 |
| 7163 | YAMANAKA T | 1 | 0.066 |
| 7164 | YAMANASHI K | 1 | 0.066 |
| 7165 | YAMAOKA-TOJO M | 1 | 0.066 |
| 7166 | YAMASAKI T | 1 | 0.066 |
| 7167 | YAMASHINA A | 1 | 0.066 |
| 7168 | YAMASHITA T | 1 | 0.066 |
| 7169 | YAMAUCHI K | 1 | 0.066 |
| 7170 | YAMAUCHI T | 1 | 0.066 |
| 7171 | YAMAUCHI Y | 1 | 0.066 |
| 7172 | YAMAZAKI S | 1 | 0.066 |
| 7173 | YAN AT | 1 | 0.066 |
| 7174 | YAN B | 1 | 0.066 |
| 7175 | YAN D | 1 | 0.066 |
| 7176 | YANAGIDA I | 1 | 0.066 |
| 7177 | YANAGIMACHI T | 1 | 0.066 |
| 7178 | YANAGIYA S | 1 | 0.066 |
| 7179 | YANCY CW | 1 | 0.066 |
| 7180 | YANG AL | 1 | 0.066 |
| 7181 | YANG BC | 1 | 0.066 |
| 7182 | YANG CC | 1 | 0.066 |
| 7183 | YANG GK | 1 | 0.066 |
| 7184 | YANG JY | 1 | 0.066 |
| 7185 | YANG LF | 1 | 0.066 |
| 7186 | YANG NI | 1 | 0.066 |
| 7187 | YANG PY | 1 | 0.066 |
| 7188 | YANG SF | 1 | 0.066 |
| 7189 | YANG SP | 1 | 0.066 |
| 7190 | YANG WS | 1 | 0.066 |
| 7191 | YANG XP | 1 | 0.066 |
| 7192 | YANG ZR | 1 | 0.066 |
| 7193 | YANO W | 1 | 0.066 |
| 7194 | YASSIN SA | 1 | 0.066 |
| 7195 | YASUDA G | 1 | 0.066 |
| 7196 | YASUDA H | 1 | 0.066 |
| 7197 | YASUDA M | 1 | 0.066 |
| 7198 | YASUDA T | 1 | 0.066 |
| 7199 | YASUMURA Y | 1 | 0.066 |
| 7200 | YATABE S | 1 | 0.066 |
| 7201 | YATES T | 1 | 0.066 |
| 7202 | YAZDANPARAST M | 1 | 0.066 |
| 7203 | YE C | 1 | 0.066 |
| 7204 | YE K | 1 | 0.066 |
| 7205 | YE L | 1 | 0.066 |
| 7206 | YEE KH | 1 | 0.066 |
| 7207 | YEH SM | 1 | 0.066 |
| 7208 | YELLON DM | 1 | 0.066 |
| 7209 | YEOH LY | 1 | 0.066 |
| 7210 | YERRA VG | 1 | 0.066 |
| 7211 | YIM HW | 1 | 0.066 |
| 7212 | YIN LH | 1 | 0.066 |
| 7213 | YIN WH | 1 | 0.066 |
| 7214 | YIP HK | 1 | 0.066 |
| 7215 | YIP PM | 1 | 0.066 |
| 7216 | YODOGAWA K | 1 | 0.066 |
| 7217 | YOKOH H | 1 | 0.066 |
| 7218 | YOKONO M | 1 | 0.066 |
| 7219 | YOKOYAMA H | 1 | 0.066 |
| 7220 | YONEDA T | 1 | 0.066 |
| 7221 | YONEKUBO S | 1 | 0.066 |
| 7222 | YOO KD | 1 | 0.066 |
| 7223 | YOON JW | 1 | 0.066 |
| 7224 | YOON YE | 1 | 0.066 |
| 7225 | YOSHIDA H | 1 | 0.066 |
| 7226 | YOSHIDA R | 1 | 0.066 |
| 7227 | YOSHIDA S | 1 | 0.066 |
| 7228 | YOSHIDA T | 1 | 0.066 |
| 7229 | YOSHIHARA F | 1 | 0.066 |
| 7230 | YOSHII A | 1 | 0.066 |
| 7231 | YOSHII T | 1 | 0.066 |
| 7232 | YOSHIKAWA T | 1 | 0.066 |
| 7233 | YOSHIMURA M | 1 | 0.066 |
| 7234 | YOSHINO T | 1 | 0.066 |
| 7235 | YOSHIZUMI M | 1 | 0.066 |
| 7236 | YOST L | 1 | 0.066 |
| 7237 | YOUNG ME | 1 | 0.066 |
| 7238 | YOUNG RJ | 1 | 0.066 |
| 7239 | YOUNG T | 1 | 0.066 |
| 7240 | YOUNGKONG S | 1 | 0.066 |
| 7241 | YOUNIS F | 1 | 0.066 |
| 7242 | YOUSEF Z | 1 | 0.066 |
| 7243 | YOUSSEF D | 1 | 0.066 |
| 7244 | YOUSSEF KD | 1 | 0.066 |
| 7245 | YU AS | 1 | 0.066 |
| 7246 | YU B | 1 | 0.066 |
| 7247 | YU CG | 1 | 0.066 |
| 7248 | YU H | 1 | 0.066 |
| 7249 | YU JW | 1 | 0.066 |
| 7250 | YU OHY | 1 | 0.066 |
| 7251 | YU P | 1 | 0.066 |
| 7252 | YU RN | 1 | 0.066 |
| 7253 | YU SH | 1 | 0.066 |
| 7254 | YU XX | 1 | 0.066 |
| 7255 | YUAN JXJ | 1 | 0.066 |
| 7256 | YUAN L | 1 | 0.066 |
| 7257 | YUASA S | 1 | 0.066 |
| 7258 | YUDKIN JS | 1 | 0.066 |
| 7259 | YUN J | 1 | 0.066 |
| 7260 | YUN JS | 1 | 0.066 |
| 7261 | YUTOH J | 1 | 0.066 |
| 7262 | ZAC-VARGHESE S | 1 | 0.066 |
| 7263 | ZAFAR MU | 1 | 0.066 |
| 7264 | ZAIDI SK | 1 | 0.066 |
| 7265 | ZAMBON A | 1 | 0.066 |
| 7266 | ZANCHETTA R | 1 | 0.066 |
| 7267 | ZANCHI A | 1 | 0.066 |
| 7268 | ZARGAR AH | 1 | 0.066 |
| 7269 | ZATTI G | 1 | 0.066 |
| 7270 | ZAUSIG Y | 1 | 0.066 |
| 7271 | ZAVARONI I | 1 | 0.066 |
| 7272 | ZAVIRIUKHA V | 1 | 0.066 |
| 7273 | ZAVYALOV EL | 1 | 0.066 |
| 7274 | ZAWADZKI NK | 1 | 0.066 |
| 7275 | ZEBEKAKIS P | 1 | 0.066 |
| 7276 | ZECCHI-ORLANDINI S | 1 | 0.066 |
| 7277 | ZECHNER PM | 1 | 0.066 |
| 7278 | ZEITZ U | 1 | 0.066 |
| 7279 | ZELEVINSKY K | 1 | 0.066 |
| 7280 | ZENG YH | 1 | 0.066 |
| 7281 | ZERAHN B | 1 | 0.066 |
| 7282 | ZHAI L | 1 | 0.066 |
| 7283 | ZHAI S | 1 | 0.066 |
| 7284 | ZHAN SY | 1 | 0.066 |
| 7285 | ZHANG B | 1 | 0.066 |
| 7286 | ZHANG BY | 1 | 0.066 |
| 7287 | ZHANG DP | 1 | 0.066 |
| 7288 | ZHANG DY | 1 | 0.066 |
| 7289 | ZHANG J | 1 | 0.066 |
| 7290 | ZHANG L | 1 | 0.066 |
| 7291 | ZHANG LA | 1 | 0.066 |
| 7292 | ZHANG LY | 1 | 0.066 |
| 7293 | ZHANG M | 1 | 0.066 |
| 7294 | ZHANG N | 1 | 0.066 |
| 7295 | ZHANG NN | 1 | 0.066 |
| 7296 | ZHANG RQ | 1 | 0.066 |
| 7297 | ZHANG RY | 1 | 0.066 |
| 7298 | ZHANG SZ | 1 | 0.066 |
| 7299 | ZHANG T | 1 | 0.066 |
| 7300 | ZHANG TT | 1 | 0.066 |
| 7301 | ZHANG WB | 1 | 0.066 |
| 7302 | ZHANG WH | 1 | 0.066 |
| 7303 | ZHANG XL | 1 | 0.066 |
| 7304 | ZHANG YJ | 1 | 0.066 |
| 7305 | ZHANG YM | 1 | 0.066 |
| 7306 | ZHANG YY | 1 | 0.066 |
| 7307 | ZHANG YZ | 1 | 0.066 |
| 7308 | ZHANG Z | 1 | 0.066 |
| 7309 | ZHANG ZW | 1 | 0.066 |
| 7310 | ZHAO JC | 1 | 0.066 |
| 7311 | ZHAO LN | 1 | 0.066 |
| 7312 | ZHAO WJ | 1 | 0.066 |
| 7313 | ZHAO WP | 1 | 0.066 |
| 7314 | ZHAO WX | 1 | 0.066 |
| 7315 | ZHAO YF | 1 | 0.066 |
| 7316 | ZHAO YG | 1 | 0.066 |
| 7317 | ZHAO YH | 1 | 0.066 |
| 7318 | ZHEN XM | 1 | 0.066 |
| 7319 | ZHENG HZ | 1 | 0.066 |
| 7320 | ZHENG PY | 1 | 0.066 |
| 7321 | ZHENG R | 1 | 0.066 |
| 7322 | ZHENG SL | 1 | 0.066 |
| 7323 | ZHENG TN | 1 | 0.066 |
| 7324 | ZHENG XX | 1 | 0.066 |
| 7325 | ZHENG Y | 1 | 0.066 |
| 7326 | ZHENG YY | 1 | 0.066 |
| 7327 | ZHONG JX | 1 | 0.066 |
| 7328 | ZHONG XB | 1 | 0.066 |
| 7329 | ZHONG XY | 1 | 0.066 |
| 7330 | ZHOU BQ | 1 | 0.066 |
| 7331 | ZHOU BY | 1 | 0.066 |
| 7332 | ZHOU H | 1 | 0.066 |
| 7333 | ZHOU J | 1 | 0.066 |
| 7334 | ZHOU LL | 1 | 0.066 |
| 7335 | ZHOU WJ | 1 | 0.066 |
| 7336 | ZHOU XH | 1 | 0.066 |
| 7337 | ZHOU XL | 1 | 0.066 |
| 7338 | ZHOU YF | 1 | 0.066 |
| 7339 | ZHU D | 1 | 0.066 |
| 7340 | ZHU DQ | 1 | 0.066 |
| 7341 | ZHU HH | 1 | 0.066 |
| 7342 | ZHU JH | 1 | 0.066 |
| 7343 | ZHU PJ | 1 | 0.066 |
| 7344 | ZHU QQ | 1 | 0.066 |
| 7345 | ZHU Y | 1 | 0.066 |
| 7346 | ZHU ZM | 1 | 0.066 |
| 7347 | ZHUANG AW | 1 | 0.066 |
| 7348 | ZHUO M | 1 | 0.066 |
| 7349 | ZIMMERMAN RS | 1 | 0.066 |
| 7350 | ZIRLIK A | 1 | 0.066 |
| 7351 | ZOCCALI C | 1 | 0.066 |
| 7352 | ZONSZEIN J | 1 | 0.066 |
| 7353 | ZOU CY | 1 | 0.066 |
| 7354 | ZOU DJ | 1 | 0.066 |
| 7355 | ZOU HH | 1 | 0.066 |
| 7356 | ZOUEIN FA | 1 | 0.066 |
| 7357 | ZUO F | 1 | 0.066 |

**Note:** SGLT2: Sodium Glucose Cotransporter 2. CV: cardiovascular
