# Supplementary material for: Bibliometric Study of Sodium Glucose Cotransporter 2 Inhibitors in Cardiovascular Research
Source: Front Pharmacol. 2020 Sep 15;11:561494. doi: 10.3389/fphar.2020.561494 (PMC7522576; doi:10.3389/fphar.2020.561494)
Supplement: Supplementary file 8 [file Table_8.docx]

Supplementary Material

**
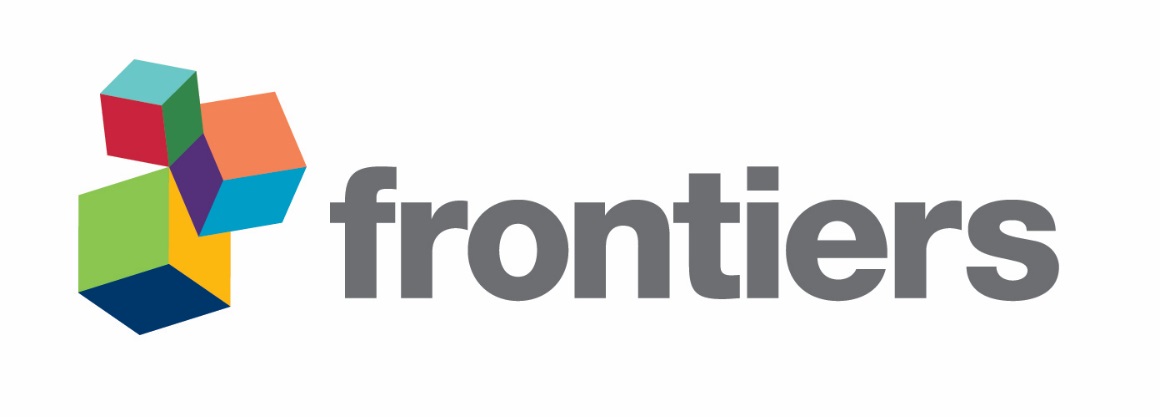
**

**Supplementary Table 8.** The cocited authors (at least 100 citations) of articles of SGLT2 inhibitors in CV research.

| **Rank** | **Cocited author** | **Citations** | **Total link strength** |
| --- | --- | --- | --- |
| 1 | neal, b | 907 | 16907 |
| 2 | zinman, b | 882 | 16699 |
| 3 | ferrannini, e | 861 | 21798 |
| 4 | rosenstock, j | 787 | 22862 |
| 5 | scheen, aj | 683 | 19906 |
| 6 | heerspink, hjl | 610 | 14352 |
| 7 | wanner, c | 593 | 13383 |
| 8 | inzucchi, se | 582 | 13569 |
| 9 | cherney, dzi | 569 | 13598 |
| 10 | defronzo, ra | 528 | 14760 |
| 11 | marso, sp | 505 | 10813 |
| 12 | gerstein, hc | 504 | 12527 |
| 13 | scirica, bm | 457 | 10727 |
| 14 | holman, rr | 439 | 11328 |
| 15 | sarafidis, pa | 431 | 8471 |
| 16 | turner, rc | 406 | 10460 |
| 17 | vallon, v | 404 | 8213 |
| 18 | wiviott, sd | 392 | 6940 |
| 19 | cefalu, wt | 352 | 9027 |
| 20 | white, wb | 342 | 8522 |
| 21 | fitchett, d | 332 | 7468 |
| 22 | wilding, jph | 332 | 10096 |
| 23 | bailey, cj | 328 | 9841 |
| 24 | kosiborod, m | 318 | 6714 |
| 25 | green, jb | 317 | 7762 |
| 26 | nauck, ma | 315 | 9643 |
| 27 | perkovic, v | 309 | 6740 |
| 28 | verma, s | 298 | 6185 |
| 29 | pfeffer, ma | 287 | 6676 |
| 30 | zelniker, ta | 269 | 5165 |
| 31 | bolinder, j | 262 | 6687 |
| 32 | mcmurray, jjv | 255 | 4289 |
| 33 | davies, mj | 249 | 5609 |
| 34 | patel, a | 241 | 6396 |
| 35 | monami, m | 238 | 5937 |
| 36 | packer, m | 233 | 4251 |
| 37 | nissen, se | 217 | 5278 |
| 38 | amer diabet, assoc | 209 | 5053 |
| 39 | mudaliar, s | 204 | 4904 |
| 40 | abdul-ghani, ma | 194 | 5430 |
| 41 | barnett, ah | 194 | 6444 |
| 42 | wright, em | 193 | 5054 |
| 43 | yale, jf | 184 | 5616 |
| 44 | vasilakou, d | 179 | 4704 |
| 45 | haring, hu | 177 | 5572 |
| 46 | henry, rr | 177 | 4934 |
| 47 | kohan, de | 173 | 5011 |
| 48 | schernthaner, g | 173 | 5494 |
| 49 | fadini, gp | 167 | 3644 |
| 50 | leiter, la | 164 | 5285 |
| 51 | kaku, k | 160 | 4913 |
| 52 | baker, wl | 157 | 4183 |
| 53 | tikkanen, i | 156 | 4284 |
| 54 | garber, aj | 155 | 3817 |
| 55 | zannad, f | 154 | 3742 |
| 56 | heise, t | 152 | 3434 |
| 57 | dormandy, ja | 148 | 3788 |
| 58 | american diabetes association | 145 | 2862 |
| 59 | duckworth, w | 143 | 3910 |
| 60 | chilton, r | 142 | 3490 |
| 61 | merovci, a | 141 | 3829 |
| 62 | sha, s | 140 | 3741 |
| 63 | stenlof, k | 139 | 4115 |
| 64 | drucker, dj | 138 | 3999 |
| 65 | roden, m | 138 | 4385 |
| 66 | us food and drug, administration | 138 | 3697 |
| 67 | fioretto, p | 137 | 3492 |
| 68 | sattar, n | 136 | 3079 |
| 69 | lytvyn, y | 131 | 3046 |
| 70 | european medicines agency | 129 | 3337 |
| 71 | abdul-ghani, m | 126 | 2856 |
| 72 | list, jf | 125 | 3168 |
| 73 | baartscheer, a | 122 | 2248 |
| 74 | gaede, p | 122 | 3347 |
| 75 | home, pd | 122 | 3271 |
| 76 | inagaki, n | 120 | 3929 |
| 77 | nathan, dm | 120 | 2844 |
| 78 | weber, ma | 118 | 3173 |
| 79 | kasichayanula, s | 117 | 3044 |
| 80 | kashiwagi, a | 115 | 3289 |
| 81 | muskiet, mha | 114 | 3281 |
| 82 | mahaffey, kw | 113 | 2839 |
| 83 | tahara, a | 113 | 1877 |
| 84 | taylor, si | 113 | 3277 |
| 85 | kovacs, cs | 112 | 3785 |
| 86 | ridderstrale, m | 112 | 3769 |
| 87 | wu, jhy | 110 | 2753 |
| 88 | devineni, d | 109 | 2733 |
| 89 | gilbert, re | 108 | 2595 |
| 90 | rahmoune, h | 108 | 2898 |
| 91 | seino, y | 106 | 3183 |
| 92 | strojek, k | 105 | 3587 |
| 93 | thomas, mc | 105 | 2503 |
| 94 | udell, ja | 103 | 2573 |
| 95 | buse, jb | 102 | 3851 |
| 96 | zoungas, s | 102 | 2853 |
| 97 | grempler, r | 101 | 2173 |
| 98 | mann, jfe | 101 | 2363 |
| 99 | tang, hl | 100 | 2665 |

**Note:** SGLT2: Sodium Glucose Cotransporter 2. CV: cardiovascular
