# Supplementary material for: Bibliometric Study of Sodium Glucose Cotransporter 2 Inhibitors in Cardiovascular Research
Source: Front Pharmacol. 2020 Sep 15;11:561494. doi: 10.3389/fphar.2020.561494 (PMC7522576; doi:10.3389/fphar.2020.561494)
Supplement: Supplementary file 9 [file Table_9.docx]

Supplementary Material

**
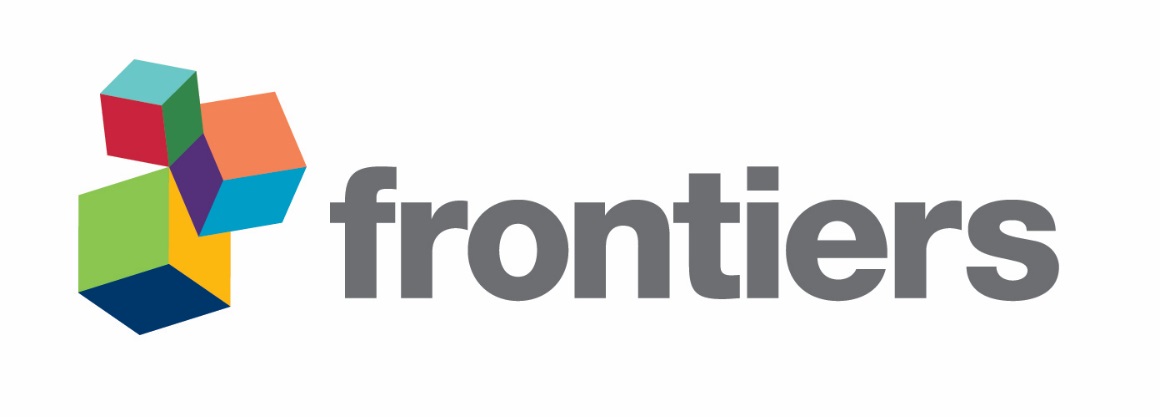
**

**Supplementary Table 9.** The cocited references related to SGLT2 inhibitors in CV research.

| **Rank** | **Count** | **Year** | **Cocited references** |
| --- | --- | --- | --- |
| 1 | 665 | 2015 | Zinman B, 2015, NEW ENGL J MED, V373, P2117, DOI 10.1056/NEJMoa1504720 |
| 2 | 622 | 2017 | Neal B, 2017, NEW ENGL J MED, V377, P644, DOI 10.1056/NEJMoa1611925 |
| 3 | 437 | 2016 | Wanner C, 2016, NEW ENGL J MED, V375, P1801 |
| 4 | 416 | 2016 | Marso SP, 2016, NEW ENGL J MED, V375, P311, DOI 10.1056/NEJMoa1603827 |
| 5 | 351 | 2016 | Sarafidis PA, 2016, NEW ENGL J MED, V374, P1092, DOI 10.1056/NEJMc1600827 |
| 6 | 345 | 2013 | Scirica BM, 2013, NEW ENGL J MED, V369, P1317, DOI 10.1056/NEJMoa1307684 |
| 7 | 310 | 2019 | Wiviott SD, 2019, NEW ENGL J MED, V380, P347, DOI 10.1056/NEJMoa1812389 |
| 8 | 293 | 2015 | Green JB, 2015, NEW ENGL J MED, V373, P232, DOI 10.1056/NEJMoa1501352 |
| 9 | 277 | 2013 | White WB, 2013, NEW ENGL J MED, V369, P1327, DOI 10.1056/NEJMoa1305889 |
| 10 | 249 | 2015 | Pfeffer MA, 2015, NEW ENGL J MED, V373, P2247, DOI 10.1056/NEJMoa1509225 |
| 11 | 221 | 2016 | Fitchett D, 2016, EUR HEART J, V37, P1526, DOI 10.1093/eurheartj/ehv728 |
| 12 | 221 | 2014 | Cherney DZI, 2014, CIRCULATION, V129, P587, DOI 10.1161/CIRCULATIONAHA.113.005081 |
| 13 | 192 | 2016 | Heerspink HJL, 2016, CIRCULATION, V134, P752, DOI 10.1161/CIRCULATIONAHA.116.021887 |
| 14 | 192 | 2016 | Ferrannini E, 2016, DIABETES CARE, V39, P1108, DOI 10.2337/dc16-0330 |
| 15 | 180 | 2013 | Heerspink HJL, 2013, DIABETES OBES METAB, V15, P853, DOI 10.1111/dom.12127 |
| 16 | 174 | 2013 | Vasilakou D, 2013, ANN INTERN MED, V159, P262, DOI 10.7326/0003-4819-159-4-201308200-00007 |
| 17 | 172 | 2017 | Kosiborod M, 2017, CIRCULATION, V136, P249, DOI 10.1161/CIRCULATIONAHA.117.029190 |
| 18 | 169 | 2019 | Zelniker TA, 2019, LANCET, V393, P31, DOI 10.1016/S0140-6736(18)32590-X |
| 19 | 168 | 2019 | Perkovic V, 2019, NEW ENGL J MED, V380, P2295, DOI 10.1056/NEJMoa1811744 |
| 20 | 151 | 2017 | Holman RR, 2017, NEW ENGL J MED, V377, P1228, DOI 10.1056/NEJMoa1612917 |
| 21 | 150 | 2014 | Ferrannini E, 2014, J CLIN INVEST, V124, P499, DOI 10.1172/JCI72227 |
| 22 | 144 | 2014 | Cherney DZI, 2014, CARDIOVASC DIABETOL, V13, P0, DOI 10.1186/1475-2840-13-28 |
| 23 | 142 | 2012 | Bolinder J, 2012, J CLIN ENDOCR METAB, V97, P1020, DOI 10.1210/jc.2011-2260 |
| 24 | 141 | 2013 | Cefalu WT, 2013, LANCET, V382, P941, DOI 10.1016/S0140-6736(13)60683-2 |
| 25 | 137 | 2016 | Mudaliar S, 2016, DIABETES CARE, V39, P1115, DOI 10.2337/dc16-0542 |
| 26 | 134 | 2014 | Kohan DE, 2014, KIDNEY INT, V85, P962, DOI 10.1038/ki.2013.356 |
| 27 | 133 | 2018 | Inzucchi SE, 2018, DIABETES CARE, V41, P0, DOI 10.2337/dc17-1551 |
| 28 | 129 | 2015 | Tikkanen I, 2015, DIABETES CARE, V38, P420, DOI 10.2337/dc14-1096 |
| 29 | 129 | 2014 | Barnett AH, 2014, LANCET DIABETES ENDO, V2, P369, DOI 10.1016/S2213-8587(13)70208-0 |
| 30 | 123 | 2014 | Baker WL, 2014, J AM SOC HYPERTENS, V8, P262, DOI 10.1016/j.jash.2014.01.007 |
| 31 | 121 | 2015 | Inzucchi SE, 2015, DIABETES VASC DIS RE, V12, P90, DOI 10.1177/1479164114559852 |
| 32 | 117 | 2015 | Chilton R, 2015, DIABETES OBES METAB, V17, P1180, DOI 10.1111/dom.12572 |
| 33 | 116 | 2015 | Zannad F, 2015, LANCET, V385, P2067, DOI 10.1016/S0140-6736(14)62225-X |
| 34 | 111 | 2014 | Merovci A, 2014, J CLIN INVEST, V124, P509, DOI 10.1172/JCI70704 |
| 35 | 111 | 2013 | Yale JF, 2013, DIABETES OBES METAB, V15, P463, DOI 10.1111/dom.12090 |
| 36 | 108 | 2016 | Wu JHY, 2016, LANCET DIABETES ENDO, V4, P411, DOI 10.1016/S2213-8587(16)00052-8 |
| 37 | 107 | 2013 | Roden M, 2013, LANCET DIABETES ENDO, V1, P208, DOI 10.1016/S2213-8587(13)70084-6 |
| 38 | 106 | 2018 | Davies MJ, 2018, DIABETES CARE, V41, P2669, DOI 10.2337/dci18-0033 |
| 39 | 103 | 2015 | Inzucchi SE, 2015, DIABETES CARE, V38, P140, DOI 10.2337/dc14-2441 |
| 40 | 103 | 2014 | Bolinder J, 2014, DIABETES OBES METAB, V16, P159, DOI 10.1111/dom.12189 |
| 41 | 100 | 2016 | Abdul-Ghani M, 2016, DIABETES CARE, V39, P717, DOI 10.2337/dc16-0041 |
| 42 | 99 | 2013 | Stenlof K, 2013, DIABETES OBES METAB, V15, P372, DOI 10.1111/dom.12054 |
| 43 | 98 | 2013 | Neal B, 2013, AM HEART J, V166, P217, DOI 10.1016/j.ahj.2013.05.007 |
| 44 | 95 | 2012 | Grempler R, 2012, DIABETES OBES METAB, V14, P83, DOI 10.1111/j.1463-1326.2011.01517.x |
| 45 | 94 | 2010 | Ferrannini E, 2010, DIABETES CARE, V33, P2217, DOI 10.2337/dc10-0612 |
| 46 | 93 | 2014 | Ridderstrale M, 2014, LANCET DIABETES ENDO, V2, P691, DOI 10.1016/S2213-8587(14)70120-2 |
| 47 | 93 | 2011 | Nauck MA, 2011, DIABETES CARE, V34, P2015, DOI 10.2337/dc11-0606 |
| 48 | 92 | 2017 | Baartscheer A, 2017, DIABETOLOGIA, V60, P568, DOI 10.1007/s00125-016-4134-x |
| 49 | 91 | 2012 | Gerstein HC, 2012, NEW ENGL J MED, V367, P319, DOI 10.1056/NEJMoa1203858 |
| 50 | 90 | 2010 | Bailey CJ, 2010, LANCET, V375, P2223, DOI 10.1016/S0140-6736(10)60407-2 |
| 51 | 85 | 2015 | Rosenstock J, 2015, DIABETES CARE, V38, P1638, DOI 10.2337/dc15-1380 |
| 52 | 83 | 2018 | Kosiborod M, 2018, J AM COLL CARDIOL, V71, P2628, DOI 10.1016/j.jacc.2018.03.009 |
| 53 | 83 | 2013 | Schernthaner G, 2013, DIABETES CARE, V36, P2508, DOI 10.2337/dc12-2491 |
| 54 | 82 | 2016 | Sattar N, 2016, DIABETOLOGIA, V59, P1333, DOI 10.1007/s00125-016-3956-x |
| 55 | 82 | 2014 | Scirica BM, 2014, CIRCULATION, V130, P1579, DOI 10.1161/CIRCULATIONAHA.114.010389 |
| 56 | 81 | 2015 | Bonner C, 2015, NAT MED, V21, P512, DOI 10.1038/nm.3828 |
| 57 | 81 | 2013 | Haring HU, 2013, DIABETES CARE, V36, P3396, DOI 10.2337/dc12-2673 |
| 58 | 80 | 2015 | Peters AL, 2015, DIABETES CARE, V38, P1687, DOI 10.2337/dc15-0843 |
| 59 | 79 | 2014 | Kovacs CS, 2014, DIABETES OBES METAB, V16, P147, DOI 10.1111/dom.12188 |
| 60 | 79 | 2014 | Vallon V, 2014, AM J PHYSIOL-RENAL, V306, P0, DOI 10.1152/ajprenal.00520.2013 |
| 61 | 79 | 2012 | Rosenstock J, 2012, DIABETES CARE, V35, P1473, DOI 10.2337/dc11-1693 |
| 62 | 79 | 2011 | Wright EM, 2011, PHYSIOL REV, V91, P733, DOI 10.1152/physrev.00055.2009 |
| 63 | 78 | 2015 | Scheen AJ, 2015, DRUGS, V75, P33, DOI 10.1007/s40265-014-0337-y |
| 64 | 76 | 2018 | Hernandez AF, 2018, LANCET, V392, P1519, DOI 10.1016/S0140-6736(18)32261-X |
| 65 | 76 | 2014 | Lin B, 2014, CARDIOVASC DIABETOL, V13, P0, DOI 10.1186/s12933-014-0148-1 |
| 66 | 75 | 2016 | Watts NB, 2016, J CLIN ENDOCR METAB, V101, P156, DOI 10.1210/jc.2015-3167 |
| 67 | 75 | 2016 | Ponikowski P, 2016, EUR HEART J, V37, P2129, DOI 10.1093/eurheartj/ehw128 |
| 68 | 75 | 2016 | Ferrannini E, 2016, DIABETES, V65, P1190, DOI 10.2337/db15-1356 |
| 69 | 75 | 2014 | Haring HU, 2014, DIABETES CARE, V37, P1650, DOI 10.2337/dc13-2105 |
| 70 | 73 | 2019 | McMurray JJV, 2019, NEW ENGL J MED, V381, P1995, DOI 10.1056/NEJMoa1911303 |
| 71 | 73 | 2017 | Lytvyn Y, 2017, CIRCULATION, V136, P1643, DOI 10.1161/CIRCULATIONAHA.117.030012 |
| 72 | 73 | 2016 | Kernan WN, 2016, NEW ENGL J MED, V374, P1321, DOI 10.1056/NEJMoa1506930 |
| 73 | 73 | 2009 | Duckworth W, 2009, NEW ENGL J MED, V360, P129, DOI 10.1056/NEJMoa0808431 |
| 74 | 72 | 2012 | Wilding JPH, 2012, ANN INTERN MED, V156, P405, DOI 10.7326/0003-4819-156-6-201203200-00003 |
| 75 | 72 | 2011 | Abdul-Ghani MA, 2011, ENDOCR REV, V32, P515, DOI 10.1210/er.2010-0029 |
| 76 | 71 | 2018 | Wanner C, 2018, CIRCULATION, V137, P119, DOI 10.1161/CIRCULATIONAHA.117.028268 |
| 77 | 71 | 2017 | Heerspink HJL, 2017, J AM SOC NEPHROL, V28, P368, DOI 10.1681/ASN.2016030278 |
| 78 | 71 | 2014 | Rosenstock J, 2014, DIABETES CARE, V37, P1815, DOI 10.2337/dc13-3055 |
| 79 | 70 | 2013 | Lavalle-Gonzalez FJ, 2013, DIABETOLOGIA, V56, P2582, DOI 10.1007/s00125-013-3039-1 |
| 80 | 70 | 2012 | Henry RR, 2012, INT J CLIN PRACT, V66, P446, DOI 10.1111/j.1742-1241.2012.02911.x |
| 81 | 70 | 2011 | Strojek K, 2011, DIABETES OBES METAB, V13, P928, DOI 10.1111/j.1463-1326.2011.01434.x |
| 82 | 68 | 2016 | Sonesson C, 2016, CARDIOVASC DIABETOL, V15, P0, DOI 10.1186/s12933-016-0356-y |
| 83 | 67 | 2016 | Zaccardi F, 2016, DIABETES OBES METAB, V18, P783, DOI 10.1111/dom.12670 |
| 84 | 66 | 2017 | Birkeland KI, 2017, LANCET DIABETES ENDO, V5, P709, DOI 10.1016/S2213-8587(17)30258-9 |
| 85 | 66 | 2017 | Jardine MJ, 2017, AM J NEPHROL, V46, P462, DOI 10.1159/000484633 |
| 86 | 65 | 2018 | Verma S, 2018, DIABETOLOGIA, V61, P2108, DOI 10.1007/s00125-018-4670-7 |
| 87 | 65 | 2018 | Mahaffey KW, 2018, CIRCULATION, V137, P323, DOI 10.1161/CIRCULATIONAHA.117.032038 |
| 88 | 65 | 2016 | Margulies KB, 2016, JAMA-J AM MED ASSOC, V316, P500, DOI 10.1001/jama.2016.10260 |
| 89 | 65 | 2012 | Rosenstock J, 2012, DIABETES CARE, V35, P1232, DOI 10.2337/dc11-1926 |
| 90 | 64 | 2018 | Radholm K, 2018, CIRCULATION, V138, P458, DOI 10.1161/CIRCULATIONAHA.118.034222 |
| 91 | 64 | 2017 | Vallon V, 2017, DIABETOLOGIA, V60, P215, DOI 10.1007/s00125-016-4157-3 |
| 92 | 64 | 2009 | List JF, 2009, DIABETES CARE, V32, P650, DOI 10.2337/dc08-1863 |
| 93 | 63 | 2019 | Rosenstock J, 2019, JAMA-J AM MED ASSOC, V321, P69, DOI 10.1001/jama.2018.18269 |
| 94 | 63 | 2017 | Mann JFE, 2017, NEW ENGL J MED, V377, P839, DOI 10.1056/NEJMoa1616011 |
| 95 | 62 | 2016 | Weber MA, 2016, LANCET DIABETES ENDO, V4, P211, DOI 10.1016/S2213-8587(15)00417-9 |
| 96 | 62 | 2012 | Inzucchi SE, 2012, DIABETES CARE, V35, P1364, DOI 10.2337/dc12-0413 |
| 97 | 61 | 2017 | Cherney DZI, 2017, LANCET DIABETES ENDO, V5, P610, DOI 10.1016/S2213-8587(17)30182-1 |
| 98 | 61 | 2012 | Ferrannini E, 2012, NAT REV ENDOCRINOL, V8, P495, DOI 10.1038/nrendo.2011.243 |
| 99 | 60 | 2018 | Perkovic V, 2018, LANCET DIABETES ENDO, V6, P691, DOI 10.1016/S2213-8587(18)30141-4 |
| 100 | 60 | 2015 | Taylor SI, 2015, J CLIN ENDOCR METAB, V100, P2849, DOI 10.1210/jc.2015-1884 |
| 101 | 60 | 2014 | Jabbour SA, 2014, DIABETES CARE, V37, P740, DOI 10.2337/dc13-0467 |
| 102 | 60 | 2012 | DeFronzo RA, 2012, DIABETES OBES METAB, V14, P5, DOI 10.1111/j.1463-1326.2011.01511.x |
| 103 | 59 | 2010 | Sarwar N, 2010, LANCET, V375, P2215, DOI 10.1016/S0140-6736(10)60484-9 |
| 104 | 58 | 2013 | Bailey CJ, 2013, BMC MED, V11, P0, DOI 10.1186/1741-7015-11-43 |
| 105 | 57 | 2016 | Frias JP, 2016, LANCET DIABETES ENDO, V4, P1004, DOI 10.1016/S2213-8587(16)30267-4 |
| 106 | 57 | 2013 | Wilding JPH, 2013, INT J CLIN PRACT, V67, P1267, DOI 10.1111/ijcp.12322 |
| 107 | 56 | 2018 | Uthman L, 2018, DIABETOLOGIA, V61, P722, DOI 10.1007/s00125-017-4509-7 |
| 108 | 56 | 2016 | Verma S, 2016, DIABETES CARE, V39, P0, DOI 10.2337/dc16-1312 |
| 109 | 56 | 2014 | Zinman B, 2014, CARDIOVASC DIABETOL, V13, P0, DOI 10.1186/1475-2840-13-102 |
| 110 | 56 | 2014 | Oliva RV, 2014, J AM SOC HYPERTENS, V8, P330, DOI 10.1016/j.jash.2014.02.003 |
| 111 | 55 | 2017 | Packer M, 2017, JAMA CARDIOL, V2, P1025, DOI 10.1001/jamacardio.2017.2275 |
| 112 | 55 | 2015 | DeFronzo RA, 2015, DIABETES CARE, V38, P384, DOI 10.2337/dc14-2364 |
| 113 | 54 | 2014 | Sha S, 2014, DIABETES OBES METAB, V16, P1087, DOI 10.1111/dom.12322 |
| 114 | 53 | 2015 | Leiter LA, 2015, DIABETES CARE, V38, P355, DOI 10.2337/dc13-2762 |
| 115 | 53 | 2015 | Ferrannini G, 2015, DIABETES CARE, V38, P1730, DOI 10.2337/dc15-0355 |
| 116 | 53 | 2014 | Yale JF, 2014, DIABETES OBES METAB, V16, P1016, DOI 10.1111/dom.12348 |
| 117 | 53 | 2014 | Chino Y, 2014, BIOPHARM DRUG DISPOS, V35, P391, DOI 10.1002/bdd.1909 |
| 118 | 53 | 2010 | Gerich JE, 2010, DIABETIC MED, V27, P136, DOI 10.1111/j.1464-5491.2009.02894.x |
| 119 | 53 | 2008 | Holman RR, 2008, NEW ENGL J MED, V359, P1577, DOI 10.1056/NEJMoa0806470 |
| 120 | 52 | 2017 | Solini A, 2017, CARDIOVASC DIABETOL, V16, P0, DOI 10.1186/s12933-017-0621-8 |
| 121 | 52 | 2017 | Lee TM, 2017, FREE RADICAL BIO MED, V104, P298, DOI 10.1016/j.freeradbiomed.2017.01.035 |
| 122 | 52 | 2014 | Liakos A, 2014, DIABETES OBES METAB, V16, P984, DOI 10.1111/dom.12307 |
| 123 | 52 | 2012 | Thomson SC, 2012, AM J PHYSIOL-REG I, V302, P0, DOI 10.1152/ajpregu.00357.2011 |
| 124 | 51 | 2015 | Rosenstock J, 2015, DIABETES OBES METAB, V17, P0, DOI 10.1111/dom.12503 |
| 125 | 51 | 2015 | Skrtic M, 2015, CURR OPIN NEPHROL HY, V24, P96, DOI 10.1097/MNH.0000000000000084 |
| 126 | 51 | 2014 | Nauck MA, 2014, DRUG DES DEV THER, V8, P1335, DOI 10.2147/DDDT.S50773 |
| 127 | 50 | 2014 | Terami N, 2014, PLOS ONE, V9, P0, DOI 10.1371/journal.pone.0100777 |
| 128 | 49 | 2018 | Davies MJ, 2018, DIABETOLOGIA, V61, P2461, DOI 10.1007/s00125-018-4729-5 |
| 129 | 49 | 2016 | Bilezikian JP, 2016, J CLIN ENDOCR METAB, V101, P43, DOI 10.1210/jc.2015-1860 |
| 130 | 49 | 2013 | Ferrannini E, 2013, DIABETES CARE, V36, P4015, DOI 10.2337/dc13-0663 |
| 131 | 49 | 2011 | Vallon V, 2011, J AM SOC NEPHROL, V22, P104, DOI 10.1681/ASN.2010030246 |
| 132 | 48 | 2018 | Hallow KM, 2018, DIABETES OBES METAB, V20, P479, DOI 10.1111/dom.13126 |
| 133 | 48 | 2018 | Amer Diabet Assoc, 2018, DIABETES CARE, V41, P0, DOI 10.2337/dc18-S008 |
| 134 | 48 | 2017 | Habibi J, 2017, CARDIOVASC DIABETOL, V16, P0, DOI 10.1186/s12933-016-0489-z |
| 135 | 48 | 2016 | Rajasekeran H, 2016, KIDNEY INT, V89, P524, DOI 10.1016/j.kint.2015.12.038 |
| 136 | 47 | 2017 | Jorsal A, 2017, EUR J HEART FAIL, V19, P69, DOI 10.1002/ejhf.657 |
| 137 | 47 | 2016 | Sano Motoaki, 2016, J Clin Med Res, V8, P844 |
| 138 | 47 | 2016 | Heerspink HJL, 2016, DIABETES OBES METAB, V18, P590, DOI 10.1111/dom.12654 |
| 139 | 47 | 2014 | Leiter LA, 2014, J AM GERIATR SOC, V62, P1252, DOI 10.1111/jgs.12881 |
| 140 | 47 | 2013 | DeFronzo RA, 2013, DIABETES CARE, V36, P3169, DOI 10.2337/dc13-0387 |
| 141 | 46 | 2019 | Kato ET, 2019, CIRCULATION, V139, P2528, DOI 10.1161/CIRCULATIONAHA.119.040130 |
| 142 | 46 | 2016 | Marx N, 2016, EUR HEART J, V37, P3192, DOI 10.1093/eurheartj/ehw110 |
| 143 | 46 | 2015 | Del Prato S, 2015, DIABETES OBES METAB, V17, P581, DOI 10.1111/dom.12459 |
| 144 | 46 | 2013 | Bode Bruce, 2013, HOSP PRACT (1995), V41, P72, DOI 10.3810/hp.2013.04.1020 |
| 145 | 45 | 2017 | [Anonymous], 2017, DIABETES CARE, V40, P0, DOI 10.2337/dc17-S001 |
| 146 | 45 | 2017 | DeFronzo RA, 2017, NAT REV NEPHROL, V13, P11, DOI 10.1038/nrneph.2016.170 |
| 147 | 45 | 2015 | Gallo LA, 2015, DIABETES VASC DIS RE, V12, P78, DOI 10.1177/1479164114561992 |
| 148 | 45 | 2013 | Heise T, 2013, DIABETES OBES METAB, V15, P613, DOI 10.1111/dom.12073 |
| 149 | 45 | 2011 | Sha S, 2011, DIABETES OBES METAB, V13, P669, DOI 10.1111/j.1463-1326.2011.01406.x |
| 150 | 44 | 2019 | Gerstein HC, 2019, LANCET, V394, P121, DOI 10.1016/S0140-6736(19)31149-3 |
| 151 | 43 | 2018 | Udell JA, 2018, CIRCULATION, V137, P1450, DOI 10.1161/CIRCULATIONAHA.117.031227 |
| 152 | 43 | 2016 | Fioretto P, 2016, DIABETES CARE, V39, P0, DOI 10.2337/dcS15-3006 |
| 153 | 43 | 2014 | Oelze M, 2014, PLOS ONE, V9, P0, DOI 10.1371/journal.pone.0112394 |
| 154 | 43 | 2009 | DeFronzo RA, 2009, DIABETES, V58, P773, DOI 10.2337/db09-9028 |
| 155 | 43 | 2008 | Patel A, 2008, NEW ENGL J MED, V358, P2560, DOI 10.1056/NEJMoa0802987 |
| 156 | 42 | 2018 | Fitchett D, 2018, EUR HEART J, V39, P363, DOI 10.1093/eurheartj/ehx511 |
| 157 | 42 | 2015 | Erondu N, 2015, DIABETES CARE, V38, P1680, DOI 10.2337/dc15-1251 |
| 158 | 42 | 2014 | Forst T, 2014, DIABETES OBES METAB, V16, P467, DOI 10.1111/dom.12273 |
| 159 | 42 | 2013 | Tahara A, 2013, EUR J PHARMACOL, V715, P246, DOI 10.1016/j.ejphar.2013.05.014 |
| 160 | 41 | 2014 | McMurray JJV, 2014, LANCET DIABETES ENDO, V2, P843, DOI 10.1016/S2213-8587(14)70031-2 |
| 161 | 41 | 2014 | Wilding JPH, 2014, DIABETES OBES METAB, V16, P124, DOI 10.1111/dom.12187 |
| 162 | 41 | 2013 | Vallon V, 2013, AM J PHYSIOL-RENAL, V304, P0, DOI 10.1152/ajprenal.00409.2012 |
| 163 | 41 | 2008 | Gerstein HC, 2008, NEW ENGL J MED, V358, P2545, DOI 10.1056/NEJMoa0802743 |
| 164 | 40 | 2016 | Cherney D, 2016, DIABETOLOGIA, V59, P1860, DOI 10.1007/s00125-016-4008-2 |
| 165 | 40 | 2015 | Bode B, 2015, DIABETES OBES METAB, V17, P294, DOI 10.1111/dom.12428 |
| 166 | 40 | 2015 | Rosenstock J, 2015, DIABETES CARE, V38, P376, DOI 10.2337/dc14-1142 |
| 167 | 40 | 2014 | Monami M, 2014, DIABETES OBES METAB, V16, P457 |
| 168 | 40 | 2014 | Rieg T, 2014, AM J PHYSIOL-RENAL, V306, P0, DOI 10.1152/ajprenal.00518.2013 |
| 169 | 39 | 2018 | Neuen BL, 2018, CIRCULATION, V138, P1537, DOI 10.1161/CIRCULATIONAHA.118.035901 |
| 170 | 39 | 2016 | Kusaka H, 2016, CARDIOVASC DIABETOL, V15, P0, DOI 10.1186/s12933-016-0473-7 |
| 171 | 39 | 2015 | Mudaliar S, 2015, DIABETES CARE, V38, P2344, DOI 10.2337/dc15-0642 |
| 172 | 39 | 2014 | Perkins BA, 2014, DIABETES CARE, V37, P1480, DOI 10.2337/dc13-2338 |
| 173 | 39 | 2013 | Panchapakesan U, 2013, PLOS ONE, V8, P0, DOI 10.1371/journal.pone.0054442 |
| 174 | 39 | 2013 | Abdul-Ghani MA, 2013, DIABETES, V62, P3324, DOI 10.2337/db13-0604 |
| 175 | 39 | 2012 | Musso G, 2012, ANN MED, V44, P375, DOI 10.3109/07853890.2011.560181 |
| 176 | 39 | 2009 | Home PD, 2009, LANCET, V373, P2125, DOI 10.1016/S0140-6736(09)60953-3 |
| 177 | 39 | 2009 | Wilding JPH, 2009, DIABETES CARE, V32, P1656, DOI 10.2337/dc09-0517 |
| 178 | 38 | 2018 | Bethel MA, 2018, LANCET DIABETES ENDO, V6, P105, DOI 10.1016/S2213-8587(17)30412-6 |
| 179 | 38 | 2017 | Shigiyama F, 2017, CARDIOVASC DIABETOL, V16, P0, DOI 10.1186/s12933-017-0564-0 |
| 180 | 38 | 2011 | Seshasai SRK, 2011, NEW ENGL J MED, V364, P829, DOI 10.1056/NEJMoa1008862 |
| 181 | 37 | 2018 | Cefalu WT, 2018, DIABETES CARE, V41, P14, DOI 10.2337/dci17-0057 |
| 182 | 37 | 2018 | Verma Subodh, 2018, JACC BASIC TRANSL SCI, V3, P575, DOI 10.1016/j.jacbts.2018.07.006 |
| 183 | 37 | 2017 | Steven S, 2017, REDOX BIOL, V13, P370, DOI 10.1016/j.redox.2017.06.009 |
| 184 | 37 | 2015 | Taylor SI, 2015, LANCET DIABETES ENDO, V3, P8, DOI 10.1016/S2213-8587(14)70227-X |
| 185 | 37 | 2014 | Pessoa TD, 2014, J AM SOC NEPHROL, V25, P2028, DOI 10.1681/ASN.2013060588 |
| 186 | 37 | 2010 | Chao EC, 2010, NAT REV DRUG DISCOV, V9, P551, DOI 10.1038/nrd3180 |
| 187 | 36 | 2018 | Raz I, 2018, DIABETES OBES METAB, V20, P1102, DOI 10.1111/dom.13217 |
| 188 | 36 | 2017 | Garg SK, 2017, NEW ENGL J MED, V377, P2337, DOI 10.1056/NEJMoa1708337 |
| 189 | 36 | 2016 | Hawley SA, 2016, DIABETES, V65, P2784, DOI 10.2337/db16-0058 |
| 190 | 36 | 2016 | Neeland IJ, 2016, DIABETES VASC DIS RE, V13, P119, DOI 10.1177/1479164115616901 |
| 191 | 36 | 2015 | Neal B, 2015, DIABETES CARE, V38, P403, DOI 10.2337/dc14-1237 |
| 192 | 36 | 2014 | Gembardt F, 2014, AM J PHYSIOL-RENAL, V307, P0, DOI 10.1152/ajprenal.00145.2014 |
| 193 | 35 | 2018 | Das SR, 2018, J AM COLL CARDIOL, V72, P3200, DOI 10.1016/j.jacc.2018.09.020 |
| 194 | 35 | 2017 | Packer M, 2017, CIRCULATION, V136, P1548, DOI 10.1161/CIRCULATIONAHA.117.030418 |
| 195 | 35 | 2017 | Butler J, 2017, EUR J HEART FAIL, V19, P1390, DOI 10.1002/ejhf.933 |
| 196 | 35 | 2013 | Ryden L, 2013, EUR HEART J, V34, P3035, DOI 10.1093/eurheartj/eht108 |
| 197 | 34 | 2019 | Mosenzon O, 2019, LANCET DIABETES ENDO, V7, P606, DOI 10.1016/S2213-8587(19)30180-9 |
| 198 | 34 | 2018 | Persson F, 2018, DIABETES OBES METAB, V20, P344, DOI 10.1111/dom.13077 |
| 199 | 34 | 2017 | Mazidi M, 2017, J AM HEART ASSOC, V6, P0, DOI 10.1161/JAHA.116.004007 |
| 200 | 34 | 2016 | Aubert G, 2016, CIRCULATION, V133, P698, DOI 10.1161/CIRCULATIONAHA.115.017355 |
| 201 | 34 | 2013 | Rosenstock J, 2013, DIABETES OBES METAB, V15, P1154, DOI 10.1111/dom.12185 |
| 202 | 33 | 2019 | Cefalu WT, 2019, DIABETES CARE, V42, P0, DOI 10.2337/dc19-S009 |
| 203 | 33 | 2019 | Zelniker TA, 2019, CIRCULATION, V139, P2022, DOI 10.1161/CIRCULATIONAHA.118.038868 |
| 204 | 33 | 2018 | Cherney DZI, 2018, KIDNEY INT, V93, P231, DOI 10.1016/j.kint.2017.06.017 |
| 205 | 33 | 2017 | Nauck MA, 2017, CIRCULATION, V136, P849, DOI 10.1161/CIRCULATIONAHA.117.028136 |
| 206 | 33 | 2017 | Xu L, 2017, EBIOMEDICINE, V20, P137, DOI 10.1016/j.ebiom.2017.05.028 |
| 207 | 33 | 2017 | Verma S, 2017, JAMA CARDIOL, V2, P939, DOI 10.1001/jamacardio.2017.1891 |
| 208 | 33 | 2016 | Storgaard H, 2016, PLOS ONE, V11, P0, DOI 10.1371/journal.pone.0166125 |
| 209 | 33 | 2015 | Cefalu WT, 2015, DIABETES CARE, V38, P1218, DOI 10.2337/dc14-0315 |
| 210 | 33 | 2012 | Ljunggren O, 2012, DIABETES OBES METAB, V14, P990, DOI 10.1111/j.1463-1326.2012.01630.x |
| 211 | 32 | 2017 | Neal B, 2017, DIABETES OBES METAB, V19, P926, DOI 10.1111/dom.12924 |
| 212 | 32 | 2017 | Ye YM, 2017, CARDIOVASC DRUG THER, V31, P119, DOI 10.1007/s10557-017-6725-2 |
| 213 | 32 | 2017 | **AmericanDiabetesAssociation, 2017, Diabetes Care, V40, P0 |
| 214 | 32 | 2016 | Lopaschuk GD, 2016, CELL METAB, V24, P200, DOI 10.1016/j.cmet.2016.07.018 |
| 215 | 32 | 2014 | Kalra S, 2014, DIABETES THER, V5, P355, DOI 10.1007/s13300-014-0089-4 |
| 216 | 32 | 2014 | Cherney DZI, 2014, KIDNEY INT, V86, P1057, DOI 10.1038/ki.2014.246 |
| 217 | 32 | 2012 | Devineni D, 2012, DIABETES OBES METAB, V14, P539, DOI 10.1111/j.1463-1326.2012.01558.x |
| 218 | 31 | 2018 | Heerspink HJL, 2018, KIDNEY INT, V94, P26, DOI 10.1016/j.kint.2017.12.027 |
| 219 | 31 | 2017 | Fadini GP, 2017, LANCET DIABETES ENDO, V5, P680, DOI 10.1016/S2213-8587(17)30257-7 |
| 220 | 31 | 2017 | Petrykiv S, 2017, CLIN J AM SOC NEPHRO, V12, P751, DOI 10.2215/CJN.10180916 |
| 221 | 31 | 2017 | Kohler S, 2017, ADV THER, V34, P1707, DOI 10.1007/s12325-017-0573-0 |
| 222 | 31 | 2017 | Byrne Nikole J, 2017, JACC BASIC TRANSL SCI, V2, P347, DOI 10.1016/j.jacbts.2017.07.003 |
| 223 | 31 | 2016 | Palmer SC, 2016, JAMA-J AM MED ASSOC, V316, P313, DOI 10.1001/jama.2016.9400 |
| 224 | 31 | 2016 | Zinman B, 2016, NEW ENGL J MED, V374, P1094, DOI 10.1056/NEJMc1600827 |
| 225 | 31 | 2013 | Johnsson KM, 2013, J DIABETES COMPLICAT, V27, P473, DOI 10.1016/j.jdiacomp.2013.05.004 |
| 226 | 30 | 2018 | Wiviott SD, 2018, AM HEART J, V200, P83, DOI 10.1016/j.ahj.2018.01.012 |
| 227 | 30 | 2018 | Zheng SL, 2018, JAMA-J AM MED ASSOC, V319, P1580, DOI 10.1001/jama.2018.3024 |
| 228 | 30 | 2017 | Baker WL, 2017, J AM HEART ASSOC, V6, P0, DOI 10.1161/JAHA.117.005686 |
| 229 | 30 | 2017 | Mosenzon O, 2017, DIABETES CARE, V40, P69, DOI 10.2337/dc16-0621 |
| 230 | 30 | 2016 | Briand F, 2016, DIABETES, V65, P2032, DOI 10.2337/db16-0049 |
| 231 | 30 | 2015 | Di Angelantonio E, 2015, JAMA-J AM MED ASSOC, V314, P52, DOI 10.1001/jama.2015.7008 |
| 232 | 30 | 2015 | Hayward RA, 2015, NEW ENGL J MED, V372, P2197, DOI 10.1056/NEJMoa1414266 |
| 233 | 30 | 2015 | Marx N, 2015, DIABETES VASC DIS RE, V12, P164, DOI 10.1177/1479164115570301 |
| 234 | 30 | 2014 | McMurray JJV, 2014, NEW ENGL J MED, V371, P993, DOI 10.1056/NEJMoa1409077 |
| 235 | 30 | 2013 | Johnsson KM, 2013, J DIABETES COMPLICAT, V27, P479, DOI 10.1016/j.jdiacomp.2013.04.012 |
| 236 | 29 | 2019 | Furtado RHM, 2019, CIRCULATION, V139, P2516, DOI 10.1161/CIRCULATIONAHA.119.039996 |
| 237 | 29 | 2018 | Rawshani A, 2018, NEW ENGL J MED, V379, P633, DOI 10.1056/NEJMoa1800256 |
| 238 | 29 | 2018 | Seferovic PM, 2018, EUR J HEART FAIL, V20, P853, DOI 10.1002/ejhf.1170 |
| 239 | 29 | 2018 | Cannon CP, 2018, AM HEART J, V206, P11, DOI 10.1016/j.ahj.2018.08.016 |
| 240 | 29 | 2017 | Januzzi JL, 2017, J AM COLL CARDIOL, V70, P704, DOI 10.1016/j.jacc.2017.06.016 |
| 241 | 29 | 2015 | Inzucchi SE, 2015, DIABETOLOGIA, V58, P429, DOI 10.1007/s00125-014-3460-0 |
| 242 | 29 | 2015 | Udell JA, 2015, LANCET DIABETES ENDO, V3, P356, DOI 10.1016/S2213-8587(15)00044-3 |
| 243 | 29 | 2015 | Lewin A, 2015, DIABETES CARE, V38, P394, DOI 10.2337/dc14-2365 |
| 244 | 28 | 2018 | Garber AJ, 2018, ENDOCR PRACT, V24, P91, DOI 10.4158/CS-2017-0153 |
| 245 | 28 | 2018 | Ryan PB, 2018, DIABETES OBES METAB, V20, P2585, DOI 10.1111/dom.13424 |
| 246 | 28 | 2017 | Dandona P, 2017, LANCET DIABETES ENDO, V5, P864, DOI 10.1016/S2213-8587(17)30308-X |
| 247 | 28 | 2016 | Li L, 2016, BMJ-BRIT MED J, V352, P0, DOI 10.1136/bmj.i610 |
| 248 | 28 | 2016 | McGuire DK, 2016, JAMA CARDIOL, V1, P126, DOI 10.1001/jamacardio.2016.0103 |
| 249 | 28 | 2015 | Shah AD, 2015, LANCET DIABETES ENDO, V3, P105, DOI 10.1016/S2213-8587(14)70219-0 |
| 250 | 28 | 2014 | Holman RR, 2014, LANCET, V383, P2008, DOI 10.1016/S0140-6736(14)60794-7 |
| 251 | 27 | 2018 | Zelniker TA, 2018, J AM COLL CARDIOL, V72, P1845, DOI 10.1016/j.jacc.2018.06.040 |
| 252 | 27 | 2017 | Wang XXX, 2017, J BIOL CHEM, V292, P5335, DOI 10.1074/jbc.M117.779520 |
| 253 | 27 | 2016 | McMurray J, 2016, J DIABETES COMPLICAT, V30, P3, DOI 10.1016/j.jdiacomp.2015.10.012 |
| 254 | 27 | 2016 | Townsend RR, 2016, J CLIN HYPERTENS, V18, P43, DOI 10.1111/jch.12747 |
| 255 | 27 | 2014 | Stenlof K, 2014, CURR MED RES OPIN, V30, P163, DOI 10.1185/03007995.2013.850066 |
| 256 | 27 | 2013 | Ptaszynska A, 2013, POSTGRAD MED, V125, P0, DOI 10.3810/pgm.2013.05.2667 |
| 257 | 27 | 2012 | Clar C, 2012, BMJ OPEN, V2, P0, DOI 10.1136/bmjopen-2012-001007 |
| 258 | 26 | 2018 | Verma S, 2018, CIRCULATION, V137, P405, DOI 10.1161/CIRCULATIONAHA.117.032031 |
| 259 | 26 | 2017 | Hammoudi N, 2017, CARDIOVASC DRUG THER, V31, P233, DOI 10.1007/s10557-017-6734-1 |
| 260 | 26 | 2017 | Fralick M, 2017, NEW ENGL J MED, V376, P2300, DOI 10.1056/NEJMc1701990 |
| 261 | 26 | 2016 | Shyangdan Deepson S, 2016, BMJ OPEN, V6, P0, DOI 10.1136/bmjopen-2015-009417 |
| 262 | 26 | 2016 | Daniele G, 2016, DIABETES CARE, V39, P2036, DOI 10.2337/dc15-2688 |
| 263 | 26 | 2015 | Vallon V, 2015, ANNU REV MED, V66, P255, DOI 10.1146/annurev-med-051013-110046 |
| 264 | 26 | 2015 | Ferrannini E, 2015, EUR HEART J, V36, P2288, DOI 10.1093/eurheartj/ehv239 |
| 265 | 26 | 2013 | Wing R, 2013, NEW ENGL J MED, V369, P145, DOI 10.1056/NEJMoa1212914 |
| 266 | 26 | 2013 | Polidori D, 2013, DIABETES CARE, V36, P2154, DOI 10.2337/dc12-2391 |
| 267 | 25 | 2020 | Cosentino F, 2020, EUR HEART J, V41, P255, DOI 10.1093/eurheartj/ehz486 |
| 268 | 25 | 2019 | Husain M, 2019, NEW ENGL J MED, V381, P841, DOI 10.1056/NEJMoa1901118 |
| 269 | 25 | 2018 | Amer Diabet Assoc, 2018, DIABETES CARE, V41, P0, DOI 10.2337/dc18-S009 |
| 270 | 25 | 2017 | Pfeifer M, 2017, CARDIOVASC DIABETOL, V16, P0, DOI 10.1186/s12933-017-0511-0 |
| 271 | 25 | 2017 | Neal B, 2017, DIABETES OBES METAB, V19, P387, DOI 10.1111/dom.12829 |
| 272 | 25 | 2017 | Ferrannini E, 2017, CELL METAB, V26, P27, DOI 10.1016/j.cmet.2017.04.011 |
| 273 | 25 | 2017 | Joubert M, 2017, DIABETES, V66, P1030, DOI 10.2337/db16-0733 |
| 274 | 25 | 2017 | Zinman B, 2017, STROKE, V48, P1218, DOI 10.1161/STROKEAHA.116.015756 |
| 275 | 25 | 2015 | Ojima A, 2015, HORM METAB RES, V47, P686, DOI 10.1055/s-0034-1395609 |
| 276 | 25 | 2015 | Cavender MA, 2015, CIRCULATION, V132, P923, DOI 10.1161/CIRCULATIONAHA.114.014796 |
| 277 | 25 | 2014 | Skrtic M, 2014, DIABETOLOGIA, V57, P2599, DOI 10.1007/s00125-014-3396-4 |
| 278 | 25 | 2012 | Bailey CJ, 2012, DIABETES OBES METAB, V14, P951, DOI 10.1111/j.1463-1326.2012.01659.x |
| 279 | 25 | 2010 | Cushman WC, 2010, NEW ENGL J MED, V362, P1575, DOI 10.1056/NEJMoa1001286 |
| 280 | 24 | 2017 | Kaku K, 2017, CIRC J, V81, P227, DOI 10.1253/circj.CJ-16-1148 |
| 281 | 24 | 2016 | Garber AJ, 2016, ENDOCR PRACT, V22, P84, DOI 10.4158/EP151126.CS |
| 282 | 24 | 2016 | Scheen AJ, 2016, DIABETES METAB, V42, P224, DOI 10.1016/j.diabet.2016.05.006 |
| 283 | 24 | 2016 | Maruthur NM, 2016, ANN INTERN MED, V164, P740, DOI 10.7326/M15-2650 |
| 284 | 24 | 2015 | Liu XY, 2015, J DIABETES COMPLICAT, V29, P1295, DOI 10.1016/j.jdiacomp.2015.07.011 |
| 285 | 24 | 2015 | Majewski C, 2015, DIABETES CARE, V38, P429, DOI 10.2337/dc14-1596 |
| 286 | 24 | 2014 | Inagaki N, 2014, EXPERT OPIN PHARMACO, V15, P1501, DOI 10.1517/14656566.2014.935764 |
| 287 | 24 | 2014 | Nyirjesy P, 2014, CURR MED RES OPIN, V30, P1109, DOI 10.1185/03007995.2014.890925 |
| 288 | 24 | 2012 | Zambrowicz B, 2012, CLIN PHARMACOL THER, V92, P158, DOI 10.1038/clpt.2012.58 |
| 289 | 24 | 2010 | Santer R, 2010, CLIN J AM SOC NEPHRO, V5, P133, DOI 10.2215/CJN.04010609 |
| 290 | 24 | 2009 | Turnbull FM, 2009, DIABETOLOGIA, V52, P2288, DOI 10.1007/s00125-009-1470-0 |
| 291 | 23 | 2018 | Soga F, 2018, CARDIOVASC DIABETOL, V17, P0, DOI 10.1186/s12933-018-0775-z |
| 292 | 23 | 2017 | Ott C, 2017, CARDIOVASC DIABETOL, V16, P0, DOI 10.1186/s12933-017-0510-1 |
| 293 | 23 | 2017 | Neal B, 2017, NEW ENGL J MED, V377, P2099, DOI 10.1056/NEJMc1712572 |
| 294 | 23 | 2016 | Gde P, 2016, DIABETOLOGIA, V59, P2298, DOI 10.1007/s00125-016-4065-6 |
| 295 | 23 | 2016 | Scheen AJ, 2016, DIABETES METAB, V42, P71, DOI 10.1016/j.diabet.2015.12.005 |
| 296 | 23 | 2016 | Cornel JH, 2016, DIABETES CARE, V39, P2304, DOI 10.2337/dc16-1415 |
| 297 | 23 | 2015 | Bailey CJ, 2015, DIABETIC MED, V32, P531, DOI 10.1111/dme.12624 |
| 298 | 23 | 2015 | Davies MJ, 2015, DIABETES OBES METAB, V17, P426, DOI 10.1111/dom.12439 |
| 299 | 23 | 2015 | Emdin CA, 2015, JAMA-J AM MED ASSOC, V313, P603, DOI 10.1001/jama.2014.18574 |
| 300 | 23 | 2014 | Yokono M, 2014, EUR J PHARMACOL, V727, P66, DOI 10.1016/j.ejphar.2014.01.040 |
| 301 | 22 | 2018 | Karg MV, 2018, CARDIOVASC DIABETOL, V17, P0, DOI 10.1186/s12933-017-0654-z |
| 302 | 22 | 2018 | Gerstein HC, 2018, DIABETES OBES METAB, V20, P42, DOI 10.1111/dom.13028 |
| 303 | 22 | 2018 | Patorno E, 2018, BMJ-BRIT MED J, V360, P0, DOI 10.1136/bmj.k119 |
| 304 | 22 | 2017 | Garber AJ, 2017, ENDOCR PRACT, V23, P207, DOI 10.4158/EP161682.CS |
| 305 | 22 | 2017 | Di Franco A, 2017, INT J CARDIOL, V243, P86, DOI 10.1016/j.ijcard.2017.05.032 |
| 306 | 22 | 2016 | Weber MA, 2016, BLOOD PRESSURE, V25, P93, DOI 10.3109/08037051.2015.1116258 |
| 307 | 22 | 2016 | Drucker DJ, 2016, CELL METAB, V24, P15, DOI 10.1016/j.cmet.2016.06.009 |
| 308 | 22 | 2015 | Pi-Sunyer X, 2015, NEW ENGL J MED, V373, P11, DOI 10.1056/NEJMoa1411892 |
| 309 | 22 | 2015 | Henry RR, 2015, DIABETES CARE, V38, P412, DOI 10.2337/dc13-2955 |
| 310 | 22 | 2014 | Hasan FM, 2014, DIABETES RES CLIN PR, V104, P297, DOI 10.1016/j.diabres.2014.02.014 |
| 311 | 22 | 2014 | Zhang M, 2014, DIABETES-METAB RES, V30, P204, DOI 10.1002/dmrr.2479 |
| 312 | 22 | 2014 | Gregg EW, 2014, NEW ENGL J MED, V370, P1514, DOI 10.1056/NEJMoa1310799 |
| 313 | 22 | 2014 | Kaku K, 2014, CARDIOVASC DIABETOL, V13, P0, DOI 10.1186/1475-2840-13-65 |
| 314 | 22 | 2013 | Afkarian M, 2013, J AM SOC NEPHROL, V24, P302, DOI 10.1681/ASN.2012070718 |
| 315 | 21 | 2018 | Sato T, 2018, CARDIOVASC DIABETOL, V17, P0, DOI 10.1186/s12933-017-0658-8 |
| 316 | 21 | 2018 | Scheen AJ, 2018, CIRC RES, V122, P1439, DOI 10.1161/CIRCRESAHA.117.311588 |
| 317 | 21 | 2018 | Lahnwong S, 2018, CARDIOVASC DIABETOL, V17, P0, DOI 10.1186/s12933-018-0745-5 |
| 318 | 21 | 2017 | Marso SP, 2017, NEW ENGL J MED, V377, P723, DOI 10.1056/NEJMoa1615692 |
| 319 | 21 | 2017 | Hayashi T, 2017, CARDIOVASC DIABETOL, V16, P0, DOI 10.1186/s12933-016-0491-5 |
| 320 | 21 | 2017 | van Bommel EJM, 2017, CLIN J AM SOC NEPHRO, V12, P700, DOI 10.2215/CJN.06080616 |
| 321 | 21 | 2017 | Han J, 2017, DIABETOLOGIA, V60, P364, DOI 10.1007/s00125-016-4158-2 |
| 322 | 21 | 2016 | Filion KB, 2016, NEW ENGL J MED, V374, P1145, DOI 10.1056/NEJMoa1506115 |
| 323 | 21 | 2015 | ONeill J, 2015, AM J PHYSIOL-RENAL, V309, P0, DOI 10.1152/ajprenal.00689.2014 |
| 324 | 21 | 2015 | Gilbert RE, 2015, LANCET, V385, P2107, DOI 10.1016/S0140-6736(14)61402-1 |
| 325 | 21 | 2015 | Scheen AJ, 2015, CLIN PHARMACOKINET, V54, P691, DOI 10.1007/s40262-015-0264-4 |
| 326 | 21 | 2015 | Kashiwagi Y, 2015, PLOS ONE, V10, P0, DOI 10.1371/journal.pone.0130605 |
| 327 | 21 | 2014 | Zoungas S, 2014, NEW ENGL J MED, V371, P1392, DOI 10.1056/NEJMoa1407963 |
| 328 | 21 | 2014 | Geerlings S, 2014, DIABETES RES CLIN PR, V103, P373, DOI 10.1016/j.diabres.2013.12.052 |
| 329 | 21 | 2013 | Kojima N, 2013, J PHARMACOL EXP THER, V345, P464, DOI 10.1124/jpet.113.203869 |
| 330 | 20 | 2019 | Figtree GA, 2019, CIRCULATION, V139, P2591, DOI 10.1161/CIRCULATIONAHA.119.040057 |
| 331 | 20 | 2019 | Santos-Gallego CG, 2019, J AM COLL CARDIOL, V73, P1931, DOI 10.1016/j.jacc.2019.01.056 |
| 332 | 20 | 2018 | Tuttle KR, 2018, LANCET DIABETES ENDO, V6, P605, DOI 10.1016/S2213-8587(18)30104-9 |
| 333 | 20 | 2018 | Jabbour S, 2018, DIABETES OBES METAB, V20, P620, DOI 10.1111/dom.13124 |
| 334 | 20 | 2018 | Thomas MC, 2018, DIABETOLOGIA, V61, P2098, DOI 10.1007/s00125-018-4669-0 |
| 335 | 20 | 2018 | Mathieu C, 2018, DIABETES CARE, V41, P1938, DOI 10.2337/dc18-0623 |
| 336 | 20 | 2017 | Nadkarni GN, 2017, DIABETES CARE, V40, P1479, DOI 10.2337/dc17-1011 |
| 337 | 20 | 2017 | Bouchi R, 2017, CARDIOVASC DIABETOL, V16, P0, DOI 10.1186/s12933-017-0516-8 |
| 338 | 20 | 2017 | Rawshani A, 2017, NEW ENGL J MED, V376, P1407, DOI 10.1056/NEJMoa1608664 |
| 339 | 20 | 2016 | Shin SJ, 2016, PLOS ONE, V11, P0, DOI 10.1371/journal.pone.0165703 |
| 340 | 20 | 2016 | **OrganizationWH, 2016, GLOB REP DIAB, V0, P0 |
| 341 | 20 | 2016 | Fioretto P, 2016, DIABETOLOGIA, V59, P2036, DOI 10.1007/s00125-016-4017-1 |
| 342 | 20 | 2015 | Lytvyn Y, 2015, AM J PHYSIOL-RENAL, V308, P0, DOI 10.1152/ajprenal.00555.2014 |
| 343 | 20 | 2015 | Nishimura R, 2015, CARDIOVASC DIABETOL, V14, P0, DOI 10.1186/s12933-014-0169-9 |
| 344 | 20 | 2014 | Nauck MA, 2014, DIABETES OBES METAB, V16, P1111, DOI 10.1111/dom.12327 |
| 345 | 20 | 2014 | Ji LN, 2014, CLIN THER, V36, P84, DOI 10.1016/j.clinthera.2013.11.002 |
| 346 | 20 | 2014 | Yang XP, 2014, EUR J CLIN PHARMACOL, V70, P1149, DOI 10.1007/s00228-014-1730-x |
| 347 | 20 | 2013 | Kaku K, 2013, DIABETES OBES METAB, V15, P432, DOI 10.1111/dom.12047 |
| 348 | 19 | 2019 | Li CG, 2019, CARDIOVASC DIABETOL, V18, P0, DOI 10.1186/s12933-019-0816-2 |
| 349 | 19 | 2019 | McMurray JJV, 2019, EUR J HEART FAIL, V21, P665, DOI 10.1002/ejhf.1432 |
| 350 | 19 | 2018 | Herrington WG, 2018, CLIN KIDNEY J, V11, P749, DOI 10.1093/ckj/sfy090 |
| 351 | 19 | 2018 | Kuchay MS, 2018, DIABETES CARE, V41, P1801, DOI 10.2337/dc18-0165 |
| 352 | 19 | 2018 | Suissa S, 2018, DIABETES CARE, V41, P6, DOI 10.2337/dc17-1223 |
| 353 | 19 | 2018 | Ueda P, 2018, BMJ-BRIT MED J, V363, P0, DOI 10.1136/bmj.k4365 |
| 354 | 19 | 2017 | Fitchett DH, 2017, EUR J HEART FAIL, V19, P43, DOI 10.1002/ejhf.633 |
| 355 | 19 | 2017 | Alicic RZ, 2017, CLIN J AM SOC NEPHRO, V12, P2032, DOI 10.2215/CJN.11491116 |
| 356 | 19 | 2016 | Heise T, 2016, CLIN THER, V38, P2265, DOI 10.1016/j.clinthera.2016.09.001 |
| 357 | 19 | 2016 | Mancia G, 2016, HYPERTENSION, V68, P1355, DOI 10.1161/HYPERTENSIONAHA.116.07703 |
| 358 | 19 | 2015 | Seferovic PM, 2015, EUR HEART J, V36, P1718, DOI 10.1093/eurheartj/ehv134 |
| 359 | 19 | 2015 | Henry RR, 2015, DIABETES CARE, V38, P2258, DOI 10.2337/dc15-1730 |
| 360 | 19 | 2015 | Scheen AJ, 2015, EXPERT OPIN DRUG SAF, V14, P505, DOI 10.1517/14740338.2015.1006625 |
| 361 | 19 | 2014 | Ptaszynska A, 2014, DRUG SAFETY, V37, P815, DOI 10.1007/s40264-014-0213-4 |
| 362 | 19 | 2014 | Weir MR, 2014, CURR MED RES OPIN, V30, P1759, DOI 10.1185/03007995.2014.919907 |
| 363 | 19 | 2014 | Kasichayanula S, 2014, CLIN PHARMACOKINET, V53, P17, DOI 10.1007/s40262-013-0104-3 |
| 364 | 19 | 2013 | Ferrannini E, 2013, DIABETES OBES METAB, V15, P721, DOI 10.1111/dom.12081 |
| 365 | 19 | 2011 | Boussageon R, 2011, BMJ-BRIT MED J, V343, P0, DOI 10.1136/bmj.d4169 |
| 366 | 18 | 2019 | Kario K, 2019, CIRCULATION, V139, P2089, DOI 10.1161/CIRCULATIONAHA.118.037076 |
| 367 | 18 | 2018 | Ludvik B, 2018, LANCET DIABETES ENDO, V6, P370, DOI 10.1016/S2213-8587(18)30023-8 |
| 368 | 18 | 2018 | Rosenstock J, 2018, DIABETES CARE, V41, P2560, DOI 10.2337/dc18-1749 |
| 369 | 18 | 2018 | Mancini SJ, 2018, SCI REP-UK, V8, P0, DOI 10.1038/s41598-018-23420-4 |
| 370 | 18 | 2017 | Ito D, 2017, DIABETES CARE, V40, P1364, DOI 10.2337/dc17-0518 |
| 371 | 18 | 2017 | Ferrannini E, 2017, DIABETES CARE, V40, P771, DOI 10.2337/dc16-2724 |
| 372 | 18 | 2016 | Wang CCL, 2016, CIRCULATION, V133, P2459, DOI 10.1161/CIRCULATIONAHA.116.022194 |
| 373 | 18 | 2015 | Muskiet MHA, 2015, LANCET DIABETES ENDO, V3, P928, DOI 10.1016/S2213-8587(15)00424-6 |
| 374 | 18 | 2014 | Yamout H, 2014, AM J NEPHROL, V40, P64, DOI 10.1159/000364909 |
| 375 | 18 | 2014 | Amer Diabet Assoc, 2014, DIABETES CARE, V37, P0, DOI 10.2337/dc14-S014 |
| 376 | 18 | 2014 | Kaku K, 2014, DIABETES OBES METAB, V16, P1102, DOI 10.1111/dom.12325 |
| 377 | 18 | 2014 | Gilbert RE, 2014, KIDNEY INT, V86, P693, DOI 10.1038/ki.2013.451 |
| 378 | 18 | 2013 | Eurich DT, 2013, CIRC-HEART FAIL, V6, P395, DOI 10.1161/CIRCHEARTFAILURE.112.000162 |
| 379 | 18 | 2013 | Shimazu T, 2013, SCIENCE, V339, P211, DOI 10.1126/science.1227166 |
| 380 | 18 | 2009 | Bakris GL, 2009, KIDNEY INT, V75, P1272, DOI 10.1038/ki.2009.87 |
| 381 | 17 | 2018 | Pabel S, 2018, EUR J HEART FAIL, V20, P1690, DOI 10.1002/ejhf.1328 |
| 382 | 17 | 2018 | Matsutani D, 2018, CARDIOVASC DIABETOL, V17, P0, DOI 10.1186/s12933-018-0717-9 |
| 383 | 17 | 2017 | Vaccaro O, 2017, LANCET DIABETES ENDO, V5, P887, DOI 10.1016/S2213-8587(17)30317-0 |
| 384 | 17 | 2017 | Tanaka H, 2017, ADV THER, V34, P436, DOI 10.1007/s12325-016-0457-8 |
| 385 | 17 | 2016 | Piepoli MF, 2016, EUR HEART J, V37, P2315, DOI 10.1093/eurheartj/ehw106 |
| 386 | 17 | 2016 | DeFronzo RA, 2016, J DIABETES COMPLICAT, V30, P1, DOI 10.1016/j.jdiacomp.2015.10.013 |
| 387 | 17 | 2015 | Wright JT, 2015, NEW ENGL J MED, V373, P2103, DOI 10.1056/NEJMoa1511939 |
| 388 | 17 | 2014 | De Nicola L, 2014, AM J KIDNEY DIS, V64, P16, DOI 10.1053/j.ajkd.2014.02.010 |
| 389 | 17 | 2012 | Meier JJ, 2012, NAT REV ENDOCRINOL, V8, P728, DOI 10.1038/nrendo.2012.140 |
| 390 | 17 | 2012 | Inzucchi SE, 2012, DIABETOLOGIA, V55, P1577, DOI 10.1007/s00125-012-2534-0 |
| 391 | 17 | 2009 | Ray KK, 2009, LANCET, V373, P1765, DOI 10.1016/S0140-6736(09)60697-8 |
| 392 | 17 | 2009 | Komoroski B, 2009, CLIN PHARMACOL THER, V85, P520, DOI 10.1038/clpt.2008.251 |
| 393 | 16 | 2019 | Patorno E, 2019, CIRCULATION, V139, P2822, DOI 10.1161/CIRCULATIONAHA.118.039177 |
| 394 | 16 | 2019 | Gerstein HC, 2019, LANCET, V394, P131, DOI 10.1016/S0140-6736(19)31150-X |
| 395 | 16 | 2019 | Verma S, 2019, LANCET, V393, P3, DOI 10.1016/S0140-6736(18)32824-1 |
| 396 | 16 | 2018 | Zhao YM, 2018, DIABETES OBES METAB, V20, P458, DOI 10.1111/dom.13101 |
| 397 | 16 | 2018 | Fioretto P, 2018, DIABETES OBES METAB, V20, P2532, DOI 10.1111/dom.13413 |
| 398 | 16 | 2018 | Cavender MA, 2018, J AM COLL CARDIOL, V71, P2497, DOI 10.1016/j.jacc.2018.01.085 |
| 399 | 16 | 2017 | Fadini GP, 2017, DIABETOLOGIA, V60, P1385, DOI 10.1007/s00125-017-4301-8 |
| 400 | 16 | 2017 | DeFronzo RA, 2017, DIABETES OBES METAB, V19, P1353, DOI 10.1111/dom.12982 |
| 401 | 16 | 2016 | Amer Diabet Assoc, 2016, DIABETES CARE, V39, P0, DOI 10.2337/dc16-S010 |
| 402 | 16 | 2015 | Vrhovac I, 2015, PFLUG ARCH EUR J PHY, V467, P1881, DOI 10.1007/s00424-014-1619-7 |
| 403 | 16 | 2015 | Matthaei S, 2015, DIABETES CARE, V38, P365, DOI 10.2337/dc14-0666 |
| 404 | 16 | 2014 | Dziuba J, 2014, DIABETES OBES METAB, V16, P628, DOI 10.1111/dom.12261 |
| 405 | 16 | 2014 | Goring S, 2014, DIABETES OBES METAB, V16, P433, DOI 10.1111/dom.12239 |
| 406 | 16 | 2012 | Gorboulev V, 2012, DIABETES, V61, P187, DOI 10.2337/db11-1029 |
| 407 | 16 | 2011 | Schramm TK, 2011, EUR HEART J, V32, P1900, DOI 10.1093/eurheartj/ehr077 |
| 408 | 16 | 2009 | Komoroski B, 2009, CLIN PHARMACOL THER, V85, P513, DOI 10.1038/clpt.2008.250 |
| 409 | 15 | 2019 | Pollock C, 2019, LANCET DIABETES ENDO, V7, P429, DOI 10.1016/S2213-8587(19)30086-5 |
| 410 | 15 | 2019 | Mahaffey KW, 2019, CIRCULATION, V140, P739, DOI 10.1161/CIRCULATIONAHA.119.042007 |
| 411 | 15 | 2018 | Wan NN, 2018, FRONT ENDOCRINOL, V9, P0, DOI 10.3389/fendo.2018.00421 |
| 412 | 15 | 2018 | Bonnet F, 2018, DIABETES METAB, V44, P457, DOI 10.1016/j.diabet.2018.09.005 |
| 413 | 15 | 2018 | Zhou H, 2018, REDOX BIOL, V15, P335, DOI 10.1016/j.redox.2017.12.019 |
| 414 | 15 | 2017 | Kappel BA, 2017, CIRCULATION, V136, P969, DOI 10.1161/CIRCULATIONAHA.117.029166 |
| 415 | 15 | 2017 | Tonneijck L, 2017, J AM SOC NEPHROL, V28, P1023, DOI 10.1681/ASN.2016060666 |
| 416 | 15 | 2017 | Saad M, 2017, INT J CARDIOL, V228, P352, DOI 10.1016/j.ijcard.2016.11.181 |
| 417 | 15 | 2016 | Singh JSS, 2016, CARDIOVASC DIABETOL, V15, P0, DOI 10.1186/s12933-016-0419-0 |
| 418 | 15 | 2016 | Armstrong MJ, 2016, LANCET, V387, P679, DOI 10.1016/S0140-6736(15)00803-X |
| 419 | 15 | 2015 | Tancredi M, 2015, NEW ENGL J MED, V373, P1720, DOI 10.1056/NEJMoa1504347 |
| 420 | 15 | 2014 | Scheen AJ, 2014, CLIN PHARMACOKINET, V53, P213, DOI 10.1007/s40262-013-0126-x |
| 421 | 15 | 2014 | Nicolle LE, 2014, POSTGRAD MED, V126, P7, DOI 10.3810/pgm.2014.01.2720 |
| 422 | 15 | 2014 | Usiskin K, 2014, POSTGRAD MED, V126, P16, DOI 10.3810/pgm.2014.05.2753 |
| 423 | 15 | 2011 | Gerstein HC, 2011, NEW ENGL J MED, V364, P818, DOI 10.1056/NEJMoa1006524 |
| 424 | 14 | 2019 | Kristensen SL, 2019, LANCET DIABETES ENDO, V7, P776, DOI 10.1016/S2213-8587(19)30249-9 |
| 425 | 14 | 2019 | Toyama T, 2019, DIABETES OBES METAB, V21, P1237, DOI 10.1111/dom.13648 |
| 426 | 14 | 2019 | Neuen BL, 2019, LANCET DIABETES ENDO, V7, P845, DOI 10.1016/S2213-8587(19)30256-6 |
| 427 | 14 | 2018 | Yuan Z, 2018, DIABETES OBES METAB, V20, P582, DOI 10.1111/dom.13115 |
| 428 | 14 | 2018 | Kosiborod M, 2018, DIABETES OBES METAB, V20, P1983, DOI 10.1111/dom.13299 |
| 429 | 14 | 2018 | Sano M, 2018, J CARDIOL, V71, P471, DOI 10.1016/j.jjcc.2017.12.004 |
| 430 | 14 | 2017 | Fadini GP, 2017, CARDIOVASC DIABETOL, V16, P0, DOI 10.1186/s12933-017-0529-3 |
| 431 | 14 | 2017 | Norton L, 2017, DIABETES OBES METAB, V19, P1322, DOI 10.1111/dom.13003 |
| 432 | 14 | 2017 | Yagi S, 2017, DIABETOL METAB SYNDR, V9, P0, DOI 10.1186/s13098-017-0275-4 |
| 433 | 14 | 2016 | Ghosh RK, 2016, INT J CARDIOL, V212, P29, DOI 10.1016/j.ijcard.2016.02.134 |
| 434 | 14 | 2016 | Bedi KC, 2016, CIRCULATION, V133, P706, DOI 10.1161/CIRCULATIONAHA.115.017545 |
| 435 | 14 | 2016 | Kohan DE, 2016, J NEPHROL, V29, P391, DOI 10.1007/s40620-016-0261-1 |
| 436 | 14 | 2015 | Mathieu C, 2015, DIABETES CARE, V38, P2009, DOI 10.2337/dc15-0779 |
| 437 | 14 | 2015 | Merovci A, 2015, J CLIN ENDOCR METAB, V100, P1927, DOI 10.1210/jc.2014-3472 |
| 438 | 14 | 2015 | Maliha G, 2015, J AM SOC HYPERTENS, V9, P48, DOI 10.1016/j.jash.2014.11.001 |
| 439 | 14 | 2014 | James PA, 2014, JAMA-J AM MED ASSOC, V311, P1809, DOI 10.1001/jama.2013.284427 |
| 440 | 14 | 2014 | Monami M, 2014, NUTR METAB CARDIOVAS, V24, P689, DOI 10.1016/j.numecd.2014.01.017 |
| 441 | 14 | 2014 | Macha S, 2014, DIABETES OBES METAB, V16, P215, DOI 10.1111/dom.12182 |
| 442 | 14 | 2013 | Fried LF, 2013, NEW ENGL J MED, V369, P1892, DOI 10.1056/NEJMoa1303154 |
| 443 | 14 | 2012 | Parving HH, 2012, NEW ENGL J MED, V367, P2204, DOI 10.1056/NEJMoa1208799 |
| 444 | 14 | 2011 | Higgins JPT, 2011, BMJ-BRIT MED J, V343, P0, DOI 10.1136/bmj.d5928 |
| 445 | 13 | 2019 | Sano M, 2019, CIRCULATION, V139, P1985, DOI 10.1161/CIRCULATIONAHA.118.038881 |
| 446 | 13 | 2019 | Verma S, 2019, CIRCULATION, V140, P1693, DOI 10.1161/CIRCULATIONAHA.119.042375 |
| 447 | 13 | 2018 | Khouri C, 2018, DIABETES OBES METAB, V20, P1531, DOI 10.1111/dom.13255 |
| 448 | 13 | 2018 | Garvey WT, 2018, METABOLISM, V85, P32, DOI 10.1016/j.metabol.2018.02.002 |
| 449 | 13 | 2018 | Pratley RE, 2018, DIABETES OBES METAB, V20, P1111, DOI 10.1111/dom.13194 |
| 450 | 13 | 2017 | Schneider MP, 2017, J AM SOC NEPHROL, V28, P1867, DOI 10.1681/ASN.2016060662 |
| 451 | 13 | 2017 | Staels B, 2017, AM J MED, V130, P0, DOI 10.1016/j.amjmed.2017.04.009 |
| 452 | 13 | 2017 | Ridker PM, 2017, NEW ENGL J MED, V377, P1119, DOI 10.1056/NEJMoa1707914 |
| 453 | 13 | 2016 | US Food and Drug Administration, 2016, FDA DRUG SAF COMM FD, V0, P0 |
| 454 | 13 | 2016 | Kohler S, 2016, CLIN THER, V38, P1299, DOI 10.1016/j.clinthera.2016.03.031 |
| 455 | 13 | 2016 | Tikkanen I, 2016, CURR OPIN NEPHROL HY, V25, P81, DOI 10.1097/MNH.0000000000000199 |
| 456 | 13 | 2016 | Savarese G, 2016, INT J CARDIOL, V220, P595, DOI 10.1016/j.ijcard.2016.06.208 |
| 457 | 13 | 2015 | Abdul-Ghani MA, 2015, AM J PHYSIOL-RENAL, V309, P0, DOI 10.1152/ajprenal.00267.2015 |
| 458 | 13 | 2015 | Ceriello A, 2015, LANCET DIABETES ENDO, V3, P929, DOI 10.1016/S2213-8587(15)00426-X |
| 459 | 13 | 2014 | Thomas MC, 2014, THER ADV ENDOCRINOL, V5, P0, DOI 10.1177/2042018814544153 |
| 460 | 13 | 2014 | Strojek K, 2014, DIABETES THER, V5, P267, DOI 10.1007/s13300-014-0072-0 |
| 461 | 13 | 2013 | Hong J, 2013, DIABETES CARE, V36, P1304, DOI 10.2337/dc12-0719 |
| 462 | 13 | 2012 | Lonborg J, 2012, EUR HEART J, V33, P1491, DOI 10.1093/eurheartj/ehr309 |
| 463 | 13 | 2012 | Vallon V, 2012, ANNU REV PHYSIOL, V74, P351, DOI 10.1146/annurev-physiol-020911-153333 |
| 464 | 13 | 2010 | Zhang L, 2010, DIABETES OBES METAB, V12, P510, DOI 10.1111/j.1463-1326.2010.01216.x |
| 465 | 13 | 2005 | Rahmoune H, 2005, DIABETES, V54, P3427, DOI 10.2337/diabetes.54.12.3427 |
| 466 | 12 | 2019 | Bersoff-Matcha SJ, 2019, ANN INTERN MED, V170, P764, DOI 10.7326/M19-0085 |
| 467 | 12 | 2019 | Kidokoro K, 2019, CIRCULATION, V140, P303, DOI 10.1161/CIRCULATIONAHA.118.037418 |
| 468 | 12 | 2019 | McMurray JJV, 2019, EUR J HEART FAIL, V0, P0, DOI 10.1002/ejhf.1548 |
| 469 | 12 | 2019 | Yurista SR, 2019, EUR J HEART FAIL, V21, P862, DOI 10.1002/ejhf.1473 |
| 470 | 12 | 2019 | Nassif ME, 2019, CIRCULATION, V140, P1463, DOI 10.1161/CIRCULATIONAHA.119.042929 |
| 471 | 12 | 2018 | Vilsboll T, 2018, DIABETES OBES METAB, V20, P889, DOI 10.1111/dom.13172 |
| 472 | 12 | 2018 | Wilcox CS, 2018, J AM HEART ASSOC, V7, P0, DOI 10.1161/JAHA.117.007046 |
| 473 | 12 | 2018 | Radholm K, 2018, DIABETES RES CLIN PR, V140, P118, DOI 10.1016/j.diabres.2018.03.027 |
| 474 | 12 | 2018 | Muskiet MHA, 2018, LANCET DIABETES ENDO, V6, P859, DOI 10.1016/S2213-8587(18)30268-7 |
| 475 | 12 | 2017 | Muskiet MHA, 2017, NAT REV NEPHROL, V13, P605, DOI 10.1038/nrneph.2017.123 |
| 476 | 12 | 2014 | Tuttle KR, 2014, DIABETES CARE, V37, P2864, DOI 10.2337/dc14-1296 |
| 477 | 12 | 2014 | Fujita Y, 2014, J DIABETES INVEST, V5, P265, DOI 10.1111/jdi.12214 |
| 478 | 12 | 2013 | Basile JN, 2013, J DIABETES COMPLICAT, V27, P280, DOI 10.1016/j.jdiacomp.2012.12.004 |
| 479 | 12 | 2012 | Foote C, 2012, DIABETES VASC DIS RE, V9, P117, DOI 10.1177/1479164112441190 |
| 480 | 12 | 2008 | Han SP, 2008, DIABETES, V57, P1723, DOI 10.2337/db07-1472 |
| 481 | 12 | 2008 | Gaede P, 2008, NEW ENGL J MED, V358, P580, DOI 10.1056/NEJMoa0706245 |
| 482 | 11 | 2018 | Bertero E, 2018, CARDIOVASC RES, V114, P12, DOI 10.1093/cvr/cvx149 |
| 483 | 11 | 2018 | Dekkers CCJ, 2018, DIABETES OBES METAB, V20, P1988, DOI 10.1111/dom.13301 |
| 484 | 11 | 2018 | Hong JL, 2018, DIABETES CARE, V41, P1196, DOI 10.2337/dc17-2212 |
| 485 | 11 | 2018 | Aroor AR, 2018, CARDIOVASC DIABETOL, V17, P0, DOI 10.1186/s12933-018-0750-8 |
| 486 | 11 | 2017 | Matthews VB, 2017, J HYPERTENS, V35, P2059, DOI 10.1097/HJH.0000000000001434 |
| 487 | 11 | 2017 | Tang HL, 2017, DIABETES OBES METAB, V19, P1106, DOI 10.1111/dom.12917 |
| 488 | 11 | 2017 | Ruanpeng D, 2017, DIABETES-METAB RES, V33, P0, DOI 10.1002/dmrr.2903 |
| 489 | 11 | 2017 | Nystrom T, 2017, DIABETES OBES METAB, V19, P831, DOI 10.1111/dom.12889 |
| 490 | 11 | 2017 | Groop PH, 2017, DIABETES OBES METAB, V19, P1610, DOI 10.1111/dom.13041 |
| 491 | 11 | 2015 | Fioretto P, 2015, CARDIOVASC DIABETOL, V14, P0, DOI 10.1186/s12933-015-0297-x |
| 492 | 11 | 2015 | Whalen K, 2015, CLIN THER, V37, P1150, DOI 10.1016/j.clinthera.2015.03.004 |
| 493 | 11 | 2015 | Amin NB, 2015, DIABETES OBES METAB, V17, P805, DOI 10.1111/dom.12486 |
| 494 | 11 | 2013 | Inagaki N, 2013, DIABETES OBES METAB, V15, P1136, DOI 10.1111/dom.12149 |
| 495 | 11 | 2013 | Tahrani AA, 2013, LANCET DIABETES ENDO, V1, P140, DOI 10.1016/S2213-8587(13)70050-0 |
| 496 | 11 | 2012 | Kasichayanula S, 2012, ADV THER, V29, P163, DOI 10.1007/s12325-011-0098-x |
| 497 | 11 | 2012 | **EuropeanMedicinesAgency, 2012, GUID CLIN INV MED PR, V0, P0 |
| 498 | 11 | 2011 | Bailey CJ, 2011, TRENDS PHARMACOL SCI, V32, P63, DOI 10.1016/j.tips.2010.11.011 |
| 499 | 11 | 2009 | Fonseca VA, 2009, DIABETES CARE, V32, P0, DOI 10.2337/dc09-S301 |
| 500 | 11 | 2007 | Wright EM, 2007, J INTERN MED, V261, P32, DOI 10.1111/j.1365-2796.2006.01746.x |
| 501 | 10 | 2019 | McGuire DK, 2019, CIRCULATION, V139, P351, DOI 10.1161/CIRCULATIONAHA.118.038352 |
| 502 | 10 | 2019 | Fralick M, 2019, ANN INTERN MED, V170, P155, DOI 10.7326/M18-0567 |
| 503 | 10 | 2019 | Adingupu DD, 2019, CARDIOVASC DIABETOL, V18, P0, DOI 10.1186/s12933-019-0820-6 |
| 504 | 10 | 2019 | Fitchett D, 2019, CIRCULATION, V139, P1384, DOI 10.1161/CIRCULATIONAHA.118.037778 |
| 505 | 10 | 2018 | Chang HY, 2018, JAMA INTERN MED, V178, P1190, DOI 10.1001/jamainternmed.2018.3034 |
| 506 | 10 | 2018 | */* S 73, 2018, DIABETES CARE S1, V41, P0, DOI DOI 10.2337/DC18-S008 |
| 507 | 10 | 2018 | Lee DM, 2018, CARDIOVASC DIABETOL, V17, P0, DOI 10.1186/s12933-018-0708-x |
| 508 | 10 | 2018 | Packer M, 2018, DIABETES OBES METAB, V20, P1361, DOI 10.1111/dom.13229 |
| 509 | 10 | 2018 | Whelton PK, 2018, HYPERTENSION, V71, P0 |
| 510 | 10 | 2017 | Perlman A, 2017, NUTR METAB CARDIOVAS, V27, P1108, DOI 10.1016/j.numecd.2017.10.011 |
| 511 | 10 | 2017 | Natali A, 2017, CARDIOVASC DIABETOL, V16, P0, DOI 10.1186/s12933-017-0615-6 |
| 512 | 10 | 2017 | Htike ZZ, 2017, DIABETES OBES METAB, V19, P524, DOI 10.1111/dom.12849 |
| 513 | 10 | 2016 | Ishibashi Y, 2016, HORM METAB RES, V48, P191, DOI 10.1055/s-0035-1555791 |
| 514 | 10 | 2016 | Handelsman Y, 2016, ENDOCR PRACT, V22, P753, DOI 10.4158/EP161292.PS |
| 515 | 10 | 2016 | Scheen AJ, 2016, EXPERT OPIN DRUG MET, V12, P1407, DOI 10.1080/17425255.2016.1215427 |
| 516 | 10 | 2016 | Salim HM, 2016, FRONT CARDIOVASC MED, V3, P0, DOI 10.3389/fcvm.2016.00043 |
| 517 | 10 | 2016 | Marso SP, 2016, NEW ENGL J MED, V375, P1834, DOI 10.1056/NEJMoa1607141 |
| 518 | 10 | 2015 | Cefalu WT, 2015, DIABETOLOGIA, V58, P1183, DOI 10.1007/s00125-015-3547-2 |
| 519 | 10 | 2014 | Kadowaki T, 2014, ADV THER, V31, P621, DOI 10.1007/s12325-014-0126-8 |
| 520 | 10 | 2013 | Kasichayanula S, 2013, BRIT J CLIN PHARMACO, V76, P432, DOI 10.1111/bcp.12056 |
| 521 | 10 | 2013 | Casagrande SS, 2013, DIABETES CARE, V36, P2271, DOI 10.2337/dc12-2258 |
| 522 | 10 | 2013 | Wilding JPH, 2013, DIABETES OBES METAB, V15, P403, DOI 10.1111/dom.12038 |
| 523 | 10 | 2013 | Cubbon RM, 2013, DIABETES VASC DIS RE, V10, P330, DOI 10.1177/1479164112471064 |
| 524 | 10 | 2013 | Rosenstock J, 2013, DIABETES VASC DIS RE, V10, P289, DOI 10.1177/1479164112475102 |
| 525 | 10 | 2013 | Yancy CW, 2013, J AM COLL CARDIOL, V62, P0, DOI 10.1016/j.jacc.2013.05.019 |
| 526 | 10 | 2013 | Monami M, 2013, DIABETES OBES METAB, V15, P112, DOI 10.1111/dom.12000 |
| 527 | 10 | 2013 | Mahaffey KW, 2013, AM HEART J, V166, P240, DOI 10.1016/j.ahj.2013.05.004 |
| 528 | 10 | 2011 | Lamanna C, 2011, DIABETES OBES METAB, V13, P221, DOI 10.1111/j.1463-1326.2010.01349.x |
| 529 | 10 | 2010 | Zoungas S, 2010, NEW ENGL J MED, V363, P1410, DOI 10.1056/NEJMoa1003795 |
| 530 | 10 | 2009 | Kooy A, 2009, ARCH INTERN MED, V169, P616, DOI 10.1001/archinternmed.2009.20 |
| 531 | 10 | 2009 | TZOULAKI I, 2009, BMJ-BRIT MED J, V339, P0, DOI DOI 10.1136/BMJ.B4731 |
| 532 | 10 | 2009 | Nathan DM, 2009, DIABETES CARE, V32, P193, DOI 10.2337/dc08-9025 |
| 533 | 10 | 2007 | Nissen SE, 2007, NEW ENGL J MED, V356, P2457, DOI 10.1056/NEJMoa072761 |
| 534 | 9 | 2019 | Garber AJ, 2019, ENDOCR PRACT, V25, P69, DOI 10.4158/CS-2018-0535 |
| 535 | 9 | 2019 | Giugliano D, 2019, DIABETES OBES METAB, V21, P1081, DOI 10.1111/dom.13629 |
| 536 | 9 | 2019 | Schork A, 2019, CARDIOVASC DIABETOL, V18, P0, DOI 10.1186/s12933-019-0852-y |
| 537 | 9 | 2018 | Wanner C, 2018, J AM SOC NEPHROL, V29, P2755, DOI 10.1681/ASN.2018010103 |
| 538 | 9 | 2018 | Danne T, 2018, DIABETES CARE, V41, P1981, DOI 10.2337/dc18-0342 |
| 539 | 9 | 2018 | [Anonymous], 2018, DIABETES CARE, V41, P0 |
| 540 | 9 | 2018 | Kaplan A, 2018, HEART FAIL REV, V23, P419, DOI 10.1007/s10741-017-9665-9 |
| 541 | 9 | 2018 | Uthman L, 2018, FRONT PHYSIOL, V9, P0, DOI 10.3389/fphys.2018.01575 |
| 542 | 9 | 2018 | Seidu S, 2018, PRIM CARE DIABETES, V12, P265, DOI 10.1016/j.pcd.2018.02.001 |
| 543 | 9 | 2017 | **InternationalDiabetesFederation, 2017, IDF DIABETES ATLAS, V0, P0 |
| 544 | 9 | 2017 | Andreadou I, 2017, FRONT PHYSIOL, V8, P0, DOI 10.3389/fphys.2017.01077 |
| 545 | 9 | 2017 | Zoungas S, 2017, LANCET DIABETES ENDO, V5, P431, DOI 10.1016/S2213-8587(17)30104-3 |
| 546 | 9 | 2017 | Petrykiv SI, 2017, DIABETES OBES METAB, V19, P1363, DOI 10.1111/dom.12936 |
| 547 | 9 | 2017 | Tang HL, 2017, DIABETOLOGIA, V60, P1862, DOI 10.1007/s00125-017-4370-8 |
| 548 | 9 | 2017 | Solini A, 2017, DIABETES OBES METAB, V19, P1289, DOI 10.1111/dom.12970 |
| 549 | 9 | 2017 | Terra SG, 2017, DIABETES OBES METAB, V19, P721, DOI 10.1111/dom.12888 |
| 550 | 9 | 2017 | Kawasoe S, 2017, BMC PHARMACOL TOXICO, V18, P0, DOI 10.1186/s40360-017-0125-x |
| 551 | 9 | 2017 | Zou HH, 2017, CARDIOVASC DIABETOL, V16, P0, DOI 10.1186/s12933-017-0547-1 |
| 552 | 9 | 2016 | Zhou B, 2016, LANCET, V387, P1513, DOI 10.1016/S0140-6736(16)00618-8 |
| 553 | 9 | 2015 | Rosenthal N, 2015, ANN NY ACAD SCI, V1358, P28, DOI 10.1111/nyas.12852 |
| 554 | 9 | 2015 | Imprialos KP, 2015, J HYPERTENS, V33, P2185, DOI 10.1097/HJH.0000000000000719 |
| 555 | 9 | 2015 | Pieber TR, 2015, DIABETES OBES METAB, V17, P0, DOI 10.1111/dom.12494 |
| 556 | 9 | 2014 | Plosker GL, 2014, DRUGS, V74, P807, DOI 10.1007/s40265-014-0225-5 |
| 557 | 9 | 2014 | Weir MR, 2014, J CLIN HYPERTENS, V16, P875, DOI 10.1111/jch.12425 |
| 558 | 9 | 2013 | Washburn WN, 2013, EXPERT OPIN INV DRUG, V22, P463, DOI 10.1517/13543784.2013.774372 |
| 559 | 9 | 2013 | Heise T, 2013, DIABETES THER, V4, P331, DOI 10.1007/s13300-013-0030-2 |
| 560 | 9 | 2013 | Devineni D, 2013, J CLIN PHARMACOL, V53, P601, DOI 10.1002/jcph.88 |
| 561 | 9 | 2012 | Nyirjesy P, 2012, CURR MED RES OPIN, V28, P1173, DOI 10.1185/03007995.2012.697053 |
| 562 | 9 | 2011 | List JF, 2011, KIDNEY INT, V79, P0, DOI 10.1038/ki.2010.512 |
| 563 | 9 | 2010 | Chen Jian, 2010, DIABETES THER, V1, P57, DOI 10.1007/s13300-010-0006-4 |
| 564 | 8 | 2019 | Mayer GJ, 2019, KIDNEY INT, V96, P489, DOI 10.1016/j.kint.2019.02.033 |
| 565 | 8 | 2019 | Cefalu WT, 2019, DIABETES CARE, V42, P0, DOI 10.2337/dc19S010 |
| 566 | 8 | 2019 | Lee HC, 2019, CARDIOVASC DIABETOL, V18, P0, DOI 10.1186/s12933-019-0849-6 |
| 567 | 8 | 2019 | Woods TC, 2019, AM J NEPHROL, V49, P331, DOI 10.1159/000499597 |
| 568 | 8 | 2018 | Williams B, 2018, EUR HEART J, V39, P3021, DOI 10.1093/eurheartj/ehy339 |
| 569 | 8 | 2018 | McMurray JJV, 2018, JACC-HEART FAIL, V6, P8, DOI 10.1016/j.jchf.2017.08.004 |
| 570 | 8 | 2018 | Mustroph J, 2018, ESC HEART FAIL, V5, P642, DOI 10.1002/ehf2.12336 |
| 571 | 8 | 2018 | Zhang XL, 2018, J AM HEART ASSOC, V7, P0, DOI 10.1161/JAHA.117.007165 |
| 572 | 8 | 2018 | Ghezzi C, 2018, DIABETOLOGIA, V61, P2087, DOI 10.1007/s00125-018-4656-5 |
| 573 | 8 | 2018 | Dagogo-Jack S, 2018, DIABETES OBES METAB, V20, P530, DOI 10.1111/dom.13116 |
| 574 | 8 | 2018 | Flores E, 2018, CARDIOVASC DRUG THER, V32, P213, DOI 10.1007/s10557-018-6786-x |
| 575 | 8 | 2018 | Rosenstock J, 2018, DIABETES OBES METAB, V20, P520, DOI 10.1111/dom.13103 |
| 576 | 8 | 2018 | Verma S, 2018, DIABETOLOGIA, V61, P1712, DOI 10.1007/s00125-018-4644-9 |
| 577 | 8 | 2017 | Li DD, 2017, DIABETES OBES METAB, V19, P348, DOI 10.1111/dom.12825 |
| 578 | 8 | 2017 | Sattar N, 2017, J AM COLL CARDIOL, V69, P2646, DOI 10.1016/j.jacc.2017.04.014 |
| 579 | 8 | 2017 | Arnold SV, 2017, EUR J PREV CARDIOL, V24, P1637, DOI 10.1177/2047487317729252 |
| 580 | 8 | 2017 | Holman RR, 2017, LANCET DIABETES ENDO, V5, P877, DOI 10.1016/S2213-8587(17)30309-1 |
| 581 | 8 | 2017 | Sabatine MS, 2017, NEW ENGL J MED, V376, P1713, DOI 10.1056/NEJMoa1615664 |
| 582 | 8 | 2017 | Liu JL, 2017, SCI REP-UK, V7, P0, DOI 10.1038/s41598-017-02733-w |
| 583 | 8 | 2017 | Griffin SJ, 2017, DIABETOLOGIA, V60, P1620, DOI 10.1007/s00125-017-4337-9 |
| 584 | 8 | 2016 | Scheen AJ, 2016, DIABETES RES CLIN PR, V121, P204, DOI 10.1016/j.diabres.2016.09.016 |
| 585 | 8 | 2016 | Hatanaka T, 2016, PHARMACOL RES PERSPE, V4, P0, DOI 10.1002/prp2.239 |
| 586 | 8 | 2016 | Toh S, 2016, ANN INTERN MED, V164, P705, DOI 10.7326/M15-2568 |
| 587 | 8 | 2016 | Davies MJ, 2016, DIABETES CARE, V39, P222, DOI 10.2337/dc14-2883 |
| 588 | 8 | 2016 | Alba M, 2016, CURR MED RES OPIN, V32, P1375, DOI 10.1080/03007995.2016.1174841 |
| 589 | 8 | 2016 | Gallo LA, 2016, SCI REP-UK, V6, P0, DOI 10.1038/srep26428 |
| 590 | 8 | 2015 | Qiang S, 2015, DIABETOL METAB SYNDR, V7, P0, DOI 10.1186/s13098-015-0102-8 |
| 591 | 8 | 2015 | Layton AT, 2015, AM J PHYSIOL-RENAL, V308, P0, DOI 10.1152/ajprenal.00007.2015 |
| 592 | 8 | 2015 | Simpson SH, 2015, LANCET DIABETES ENDO, V3, P43, DOI 10.1016/S2213-8587(14)70213-X |
| 593 | 8 | 2015 | Sands AT, 2015, DIABETES CARE, V38, P1181, DOI 10.2337/dc14-2806 |
| 594 | 8 | 2015 | Lambert R, 2015, J AM HEART ASSOC, V4, P0, DOI 10.1161/JAHA.115.002183 |
| 595 | 8 | 2015 | Han Y, 2015, AM J PHYSIOL-LUNG C, V309, P0, DOI 10.1152/ajplung.00167.2015 |
| 596 | 8 | 2013 | Rojas LBA, 2013, DIABETOL METAB SYNDR, V5, P0, DOI 10.1186/1758-5996-5-6 |
| 597 | 8 | 2013 | Berhan A, 2013, BMC ENDOCR DISORD, V13, P0, DOI 10.1186/1472-6823-13-58 |
| 598 | 8 | 2013 | Monami M, 2013, DIABETES OBES METAB, V15, P938, DOI 10.1111/dom.12116 |
| 599 | 8 | 2012 | Liu JW, 2012, DIABETES, V61, P2199, DOI 10.2337/db12-0052 |
| 600 | 8 | 2012 | Liang Y, 2012, PLOS ONE, V7, P0, DOI 10.1371/journal.pone.0030555 |
| 601 | 8 | 2012 | Boonman-de Winter LJM, 2012, DIABETOLOGIA, V55, P2154, DOI 10.1007/s00125-012-2579-0 |
| 602 | 8 | 2011 | Bennett WL, 2011, ANN INTERN MED, V154, P602, DOI 10.7326/0003-4819-154-9-201105030-00336 |
| 603 | 8 | 2011 | Kasichayanula S, 2011, DIABETES OBES METAB, V13, P47, DOI 10.1111/j.1463-1326.2010.01314.x |
| 604 | 8 | 2010 | Ismail-Beigi F, 2010, LANCET, V376, P419, DOI 10.1016/S0140-6736(10)60576-4 |
| 605 | 8 | 2009 | Nauck M, 2009, DIABETES CARE, V32, P84, DOI 10.2337/dc08-1355 |
| 606 | 8 | 2008 | Abdul-Ghani Muhammad A, 2008, Endocr Pract, V14, P782 |
| 607 | 8 | 2006 | Kahn SE, 2006, NEW ENGL J MED, V355, P2427, DOI 10.1056/NEJMoa066224 |
| 608 | 8 | 2005 | Ehrenkranz JRL, 2005, DIABETES-METAB RES, V21, P31, DOI 10.1002/dmrr.532 |
| 609 | 7 | 2020 | van Bommel EJM, 2020, KIDNEY INT, V97, P202, DOI 10.1016/j.kint.2019.09.013 |
| 610 | 7 | 2019 | Verma S, 2019, CIRCULATION, V139, P2537, DOI 10.1161/CIRCULATIONAHA.119.040514 |
| 611 | 7 | 2019 | Bhatt DL, 2019, NEW ENGL J MED, V380, P11, DOI 10.1056/NEJMoa1812792 |
| 612 | 7 | 2019 | Scheen AJ, 2019, EXPERT OPIN DRUG SAF, V18, P295, DOI 10.1080/14740338.2019.1602116 |
| 613 | 7 | 2019 | Santos-Gallego CG, 2019, CARDIOVASC DRUG THER, V33, P87, DOI 10.1007/s10557-018-06850-0 |
| 614 | 7 | 2019 | Dawwas GK, 2019, DIABETES OBES METAB, V21, P28, DOI 10.1111/dom.13477 |
| 615 | 7 | 2019 | Matthews DR, 2019, DIABETOLOGIA, V62, P926, DOI 10.1007/s00125-019-4839-8 |
| 616 | 7 | 2018 | Jia GH, 2018, CIRC RES, V122, P624, DOI 10.1161/CIRCRESAHA.117.311586 |
| 617 | 7 | 2018 | Fadini GP, 2018, DIABETES OBES METAB, V20, P740, DOI 10.1111/dom.13130 |
| 618 | 7 | 2018 | Rocha ND, 2018, DIABETES VASC DIS RE, V15, P375, DOI 10.1177/1479164118783756 |
| 619 | 7 | 2018 | Greene SJ, 2018, J AM COLL CARDIOL, V71, P1379, DOI 10.1016/j.jacc.2018.01.047 |
| 620 | 7 | 2018 | Packer M, 2018, JACC-HEART FAIL, V6, P445, DOI 10.1016/j.jchf.2017.12.016 |
| 621 | 7 | 2018 | Guo M, 2018, DIABETES OBES METAB, V20, P1977, DOI 10.1111/dom.13295 |
| 622 | 7 | 2018 | Einarson TR, 2018, CARDIOVASC DIABETOL, V17, P0, DOI 10.1186/s12933-018-0728-6 |
| 623 | 7 | 2018 | Lipscombe L, 2018, CAN J DIABETES, V42, P0, DOI 10.1016/j.jcjd.2017.10.034 |
| 624 | 7 | 2018 | Puckrin R, 2018, ACTA DIABETOL, V55, P503, DOI 10.1007/s00592-018-1116-0 |
| 625 | 7 | 2018 | Lee PC, 2018, OBES REV, V19, P1630, DOI 10.1111/obr.12755 |
| 626 | 7 | 2018 | Grunberger G, 2018, DIABETES THER, V9, P49, DOI 10.1007/s13300-017-0337-5 |
| 627 | 7 | 2018 | Wang ZY, 2018, DIABETES OBES METAB, V20, P113, DOI 10.1111/dom.13047 |
| 628 | 7 | 2018 | Li L, 2018, J PHARMACOL SCI, V137, P220, DOI 10.1016/j.jphs.2017.10.006 |
| 629 | 7 | 2018 | Scheen AJ, 2018, DIABETES RES CLIN PR, V143, P88, DOI 10.1016/j.diabres.2018.06.008 |
| 630 | 7 | 2018 | Li D, 2018, DIABETES METAB, V44, P410, DOI 10.1016/j.diabet.2018.02.001 |
| 631 | 7 | 2018 | Seino Y, 2018, J DIABETES INVEST, V9, P332, DOI 10.1111/jdi.12694 |
| 632 | 7 | 2018 | Wittbrodt ET, 2018, AM J MANAG CARE, V24, P0 |
| 633 | 7 | 2018 | Amer Diabet Assoc, 2018, DIABETES CARE, V41, P0, DOI 10.2337/dc18-S006 |
| 634 | 7 | 2017 | Burke KR, 2017, PHARMACOTHERAPY, V37, P187, DOI 10.1002/phar.1881 |
| 635 | 7 | 2017 | Softeland E, 2017, DIABETES CARE, V40, P201, DOI 10.2337/dc16-1347 |
| 636 | 7 | 2017 | Zhou Y, 2017, CELL PHYSIOL BIOCHEM, V41, P2503, DOI 10.1159/000475942 |
| 637 | 7 | 2017 | Azoulay L, 2017, DIABETES CARE, V40, P706, DOI 10.2337/dc16-1943 |
| 638 | 7 | 2017 | Wanner C, 2017, AM J CARDIOL, V120, P0, DOI 10.1016/j.amjcard.2017.05.012 |
| 639 | 7 | 2017 | Mizuno Y, 2017, METABOLISM, V77, P65, DOI 10.1016/j.metabol.2017.08.005 |
| 640 | 7 | 2017 | Sano Motoaki, 2017, J CLIN MED RES, V9, P457, DOI 10.14740/jocmr3011w |
| 641 | 7 | 2017 | Ogurtsova K, 2017, DIABETES RES CLIN PR, V128, P40, DOI 10.1016/j.diabres.2017.03.024 |
| 642 | 7 | 2017 | Al-Jobori H, 2017, DIABETES, V66, P1999, DOI 10.2337/db17-0100 |
| 643 | 7 | 2017 | Kawanami D, 2017, INT J MOL SCI, V18, P0, DOI 10.3390/ijms18051083 |
| 644 | 7 | 2017 | Lovshin JA, 2017, DIABETES CARE, V40, P1073, DOI 10.2337/dc17-0061 |
| 645 | 7 | 2017 | Tanaka A, 2017, CARDIOVASC DIABETOL, V16, P0, DOI 10.1186/s12933-017-0532-8 |
| 646 | 7 | 2016 | Tahrani AA, 2016, NAT REV ENDOCRINOL, V12, P566, DOI 10.1038/nrendo.2016.86 |
| 647 | 7 | 2016 | Tonneijck L, 2016, DIABETES CARE, V39, P2042, DOI 10.2337/dc16-1371 |
| 648 | 7 | 2016 | Tang HL, 2016, DIABETES OBES METAB, V18, P1199, DOI 10.1111/dom.12742 |
| 649 | 7 | 2016 | Heise T, 2016, CLIN THER, V38, P2248, DOI 10.1016/j.clinthera.2016.08.008 |
| 650 | 7 | 2016 | Nauck M, 2016, DIABETES OBES METAB, V18, P203, DOI 10.1111/dom.12591 |
| 651 | 7 | 2016 | Honda Y, 2016, PLOS ONE, V11, P0, DOI 10.1371/journal.pone.0146337 |
| 652 | 7 | 2016 | **CanadianDiabetesAssociationClinicalPracticeGuidelinesExpertCommittee, 2016, CAN J DIABETES, V40, P484, DOI 10.1016/j.jcjd.2016.09.003 |
| 653 | 7 | 2016 | Yavin Y, 2016, DIABETES THER, V7, P125, DOI 10.1007/s13300-015-0150-y |
| 654 | 7 | 2016 | Kern M, 2016, METABOLISM, V65, P114, DOI 10.1016/j.metabol.2015.10.010 |
| 655 | 7 | 2016 | Takashima S, 2016, KIDNEY INT, V90, P783, DOI 10.1016/j.kint.2016.06.012 |
| 656 | 7 | 2016 | Song P, 2016, EXPERT OPIN THER TAR, V20, P1109, DOI 10.1517/14728222.2016.1168808 |
| 657 | 7 | 2016 | Jojima T, 2016, DIABETOL METAB SYNDR, V8, P0, DOI 10.1186/s13098-016-0169-x |
| 658 | 7 | 2016 | Kristensen SL, 2016, CIRC-HEART FAIL, V9, P0, DOI 10.1161/CIRCHEARTFAILURE.115.002560 |
| 659 | 7 | 2016 | Hahn K, 2016, NAT REV NEPHROL, V12, P711, DOI 10.1038/nrneph.2016.159 |
| 660 | 7 | 2016 | Rahman A, 2016, J HYPERTENS, V34, P893, DOI 10.1097/HJH.0000000000000871 |
| 661 | 7 | 2015 | Sun F, 2015, DIABETES RES CLIN PR, V110, P26, DOI 10.1016/j.diabres.2015.07.015 |
| 662 | 7 | 2015 | Roden M, 2015, CARDIOVASC DIABETOL, V14, P0, DOI 10.1186/s12933-015-0314-0 |
| 663 | 7 | 2015 | Matthaei S, 2015, DIABETES CARE, V38, P2018, DOI 10.2337/dc15-0811 |
| 664 | 7 | 2015 | [Anonymous], 2015, DIABETES CARE, V38, P0, DOI 10.2337/dc15-S001 |
| 665 | 7 | 2015 | Kovacs CS, 2015, CLIN THER, V37, P1773, DOI 10.1016/j.clinthera.2015.05.511 |
| 666 | 7 | 2015 | [Anonymous], 2015, IDF DIABETES ATLAS, V0, P0 |
| 667 | 7 | 2015 | Abdul-Ghani MA, 2015, DIABETES OBES METAB, V17, P268, DOI 10.1111/dom.12417 |
| 668 | 7 | 2014 | Ussher JR, 2014, CIRC RES, V114, P1788, DOI 10.1161/CIRCRESAHA.114.301958 |
| 669 | 7 | 2014 | Mulvihill EE, 2014, ENDOCR REV, V35, P992, DOI 10.1210/er.2014-1035 |
| 670 | 7 | 2014 | Plosker G, 2014, DRUGS, V74, P2191, DOI 10.1007/s40265-014-0324-3 |
| 671 | 7 | 2014 | Seino Y, 2014, CURR MED RES OPIN, V30, P1245, DOI 10.1185/03007995.2014.912983 |
| 672 | 7 | 2014 | Fujita H, 2014, KIDNEY INT, V85, P579, DOI 10.1038/ki.2013.427 |
| 673 | 7 | 2014 | Suzuki M, 2014, NUTR DIABETES, V4, P0, DOI 10.1038/nutd.2014.20 |
| 674 | 7 | 2013 | Friedrich C, 2013, CLIN THER, V35, P0, DOI 10.1016/j.clinthera.2012.12.002 |
| 675 | 7 | 2012 | Devenny JJ, 2012, OBESITY, V20, P1645, DOI 10.1038/oby.2012.59 |
| 676 | 7 | 2011 | Schwartz SL, 2011, DIABETES TECHNOL THE, V13, P1219, DOI 10.1089/dia.2011.0012 |
| 677 | 7 | 2011 | Whiting DR, 2011, DIABETES RES CLIN PR, V94, P311, DOI 10.1016/j.diabres.2011.10.029 |
| 678 | 7 | 2008 | Nissen SE, 2008, JAMA-J AM MED ASSOC, V299, P1561, DOI 10.1001/jama.299.13.1561 |
| 679 | 7 | 2008 | Freitas HS, 2008, ENDOCRINOLOGY, V149, P717, DOI 10.1210/en.2007-1088 |
| 680 | 6 | 2020 | Mazer CD, 2020, CIRCULATION, V141, P704, DOI 10.1161/CIRCULATIONAHA.119.044235 |
| 681 | 6 | 2019 | Uthman L, 2019, CARDIOVASC RES, V115, P1533, DOI 10.1093/cvr/cvz004 |
| 682 | 6 | 2019 | Eickhoff MK, 2019, J CLIN MED, V8, P0, DOI 10.3390/jcm8060779 |
| 683 | 6 | 2019 | Butler J, 2019, CIRC-HEART FAIL, V12, P0, DOI 10.1161/CIRCHEARTFAILURE.118.005875 |
| 684 | 6 | 2019 | Rosenstock J, 2019, JAMA-J AM MED ASSOC, V322, P1155, DOI 10.1001/jama.2019.13772 |
| 685 | 6 | 2019 | Kelly MS, 2019, POSTGRAD MED, V131, P31, DOI 10.1080/00325481.2019.1549459 |
| 686 | 6 | 2019 | **InternationalDiabetesFederation, 2019, IDF DIABETES ATLAS, V0, P0 |
| 687 | 6 | 2019 | Kluger AY, 2019, CARDIOVASC DIABETOL, V18, P0, DOI 10.1186/s12933-019-0903-4 |
| 688 | 6 | 2019 | Horton JL, 2019, JCI INSIGHT, V4, P0, DOI 10.1172/jci.insight.124079 |
| 689 | 6 | 2019 | Connelly Kim A, 2019, JACC BASIC TRANSL SCI, V4, P27, DOI 10.1016/j.jacbts.2018.11.010 |
| 690 | 6 | 2019 | [Anonymous], 2019, DIABETES CARE, V42, P0, DOI 10.2337/DC19-S009] |
| 691 | 6 | 2019 | Heerspink HJL, 2019, DIABETOLOGIA, V62, P1154, DOI 10.1007/s00125-019-4859-4 |
| 692 | 6 | 2019 | Danne T, 2019, DIABETES CARE, V42, P1147, DOI 10.2337/dc18-2316 |
| 693 | 6 | 2018 | Dandona P, 2018, DIABETES CARE, V41, P2552, DOI 10.2337/dc18-1087 |
| 694 | 6 | 2018 | Inzucchi SE, 2018, CIRCULATION, V138, P1904, DOI 10.1161/CIRCULATIONAHA.118.035759 |
| 695 | 6 | 2018 | Mizuno M, 2018, PHYSIOL REP, V6, P0, DOI 10.14814/phy2.13741 |
| 696 | 6 | 2018 | Cai XL, 2018, OBESITY, V26, P70, DOI 10.1002/oby.22066 |
| 697 | 6 | 2018 | Basu D, 2018, ARTERIOSCL THROM VAS, V38, P2207, DOI 10.1161/ATVBAHA.118.311339 |
| 698 | 6 | 2017 | Rahman A, 2017, HYPERTENS RES, V40, P535, DOI 10.1038/hr.2016.193 |
| 699 | 6 | 2017 | Fukuda T, 2017, DIABETES THER, V8, P851, DOI 10.1007/s13300-017-0279-y |
| 700 | 6 | 2017 | Tkac I, 2017, DIABETES CARE, V40, P284, DOI 10.2337/dc15-1707 |
| 701 | 6 | 2017 | Martens Pieter, 2017, CURR TREAT OPTIONS CARDIOVASC MED, V19, P23, DOI 10.1007/s11936-017-0522-x |
| 702 | 6 | 2017 | Toulis KA, 2017, J CLIN ENDOCR METAB, V102, P1719, DOI 10.1210/jc.2016-3446 |
| 703 | 6 | 2017 | Neal B, 2017, N ENGL J MED, V0, P0 |
| 704 | 6 | 2017 | Tang L, 2017, AM J PHYSIOL-ENDOC M, V313, P0, DOI 10.1152/ajpendo.00086.2017 |
| 705 | 6 | 2017 | Verma Subodh, 2017, CMAJ OPEN, V5, P0, DOI 10.9778/cmajo.20160058 |
| 706 | 6 | 2017 | Shi XJ, 2017, MOL CELL BIOCHEM, V433, P97, DOI 10.1007/s11010-017-3018-9 |
| 707 | 6 | 2017 | Staels B, 2017, AM J CARDIOL, V120, P0, DOI 10.1016/j.amjcard.2017.05.013 |
| 708 | 6 | 2017 | Hsia DS, 2017, CURR OPIN ENDOCRINOL, V24, P73, DOI 10.1097/MED.0000000000000311 |
| 709 | 6 | 2016 | Blonde L, 2016, POSTGRAD MED, V128, P371, DOI 10.1080/00325481.2016.1169894 |
| 710 | 6 | 2016 | Cherney DZI, 2016, CIRCULATION, V134, P1915, DOI 10.1161/CIRCULATIONAHA.116.024764 |
| 711 | 6 | 2016 | Sinclair AJ, 2016, J AM GERIATR SOC, V64, P543, DOI 10.1111/jgs.14028 |
| 712 | 6 | 2016 | Mulvihill EE, 2016, DIABETES, V65, P742, DOI 10.2337/db15-1224 |
| 713 | 6 | 2016 | Goldenberg R, 2016, CAN J DIABETES, V40, P193, DOI 10.1016/j.jcjd.2016.02.006 |
| 714 | 6 | 2016 | Scheen AJ, 2016, CURR DIABETES REP, V16, P0, DOI 10.1007/s11892-016-0789-4 |
| 715 | 6 | 2016 | Nauck MA, 2016, LANCET DIABETES ENDO, V4, P525, DOI 10.1016/S2213-8587(15)00482-9 |
| 716 | 6 | 2016 | DeFronzo RA, 2016, DIABETES OBES METAB, V18, P454, DOI 10.1111/dom.12652 |
| 717 | 6 | 2016 | Rados DV, 2016, PLOS MED, V13, P0, DOI 10.1371/journal.pmed.1001992 |
| 718 | 6 | 2016 | Marso SP, 2016, N ENGL J MED, V0, P0 |
| 719 | 6 | 2016 | Layton AT, 2016, AM J PHYSIOL-RENAL, V310, P0, DOI 10.1152/ajprenal.00543.2015 |
| 720 | 6 | 2016 | Kimura G, 2016, J AM SOC HYPERTENS, V10, P271, DOI 10.1016/j.jash.2016.01.009 |
| 721 | 6 | 2016 | Tang HL, 2016, AM J CARDIOL, V118, P1774, DOI 10.1016/j.amjcard.2016.08.061 |
| 722 | 6 | 2016 | Fu AZ, 2016, DIABETES CARE, V39, P726, DOI 10.2337/dc15-0764 |
| 723 | 6 | 2016 | Zhang XD, 2016, J HYPERTENS, V34, P167, DOI 10.1097/HJH.0000000000000782 |
| 724 | 6 | 2016 | [Anonymous], 2016, DIABETES CARE, V39, P0, DOI 10.2337/dc16-S001 |
| 725 | 6 | 2016 | Komiya C, 2016, PLOS ONE, V11, P0, DOI 10.1371/journal.pone.0151511 |
| 726 | 6 | 2016 | Madsbad S, 2016, DIABETES OBES METAB, V18, P317, DOI 10.1111/dom.12596 |
| 727 | 6 | 2016 | Kimura G, 2016, CIRC J, V80, P2277, DOI 10.1253/circj.CJ-16-0780 |
| 728 | 6 | 2016 | Xie XF, 2016, LANCET, V387, P435, DOI 10.1016/S0140-6736(15)00805-3 |
| 729 | 6 | 2015 | Cooper ME, 2015, AM J KIDNEY DIS, V66, P441, DOI 10.1053/j.ajkd.2015.03.024 |
| 730 | 6 | 2015 | Handelsman Y, 2015, ENDOCR PRACT, V21, P1 |
| 731 | 6 | 2015 | Liakos A, 2015, THER ADV ENDOCRINOL, V6, P61, DOI 10.1177/2042018814560735 |
| 732 | 6 | 2015 | Scheen AJ, 2015, EXPERT OPIN PHARMACO, V16, P43, DOI 10.1517/14656566.2015.978289 |
| 733 | 6 | 2015 | Terasaki M, 2015, PLOS ONE, V10, P0, DOI 10.1371/journal.pone.0143396 |
| 734 | 6 | 2015 | Sjostrom CD, 2015, DIABETES VASC DIS RE, V12, P352, DOI 10.1177/1479164115585298 |
| 735 | 6 | 2015 | Ou SM, 2015, ANN INTERN MED, V163, P663, DOI 10.7326/M15-0308 |
| 736 | 6 | 2015 | Kashiwagi A, 2015, DIABETES OBES METAB, V17, P304, DOI 10.1111/dom.12331 |
| 737 | 6 | 2015 | Sjostrom CD, 2015, DIABETES OBES METAB, V17, P809, DOI 10.1111/dom.12500 |
| 738 | 6 | 2015 | Inagaki N, 2015, J DIABETES INVEST, V6, P210, DOI 10.1111/jdi.12266 |
| 739 | 6 | 2015 | Matthaei S, 2015, DIABETES OBES METAB, V17, P1075, DOI 10.1111/dom.12543 |
| 740 | 6 | 2015 | Rosenstock J, 2015, CARDIOVASC DIABETOL, V14, P0, DOI 10.1186/s12933-015-0215-2 |
| 741 | 6 | 2015 | Heerspink HJL, 2015, J AM SOC NEPHROL, V26, P2055, DOI 10.1681/ASN.2014070688 |
| 742 | 6 | 2015 | Khunti K, 2015, DIABETES CARE, V38, P316, DOI 10.2337/dc14-0920 |
| 743 | 6 | 2015 | Lovshin JA, 2015, DIABETES CARE, V38, P132, DOI 10.2337/dc14-1958 |
| 744 | 6 | 2014 | Kaku K, 2014, DIABETES THER, V5, P415, DOI 10.1007/s13300-014-0086-7 |
| 745 | 6 | 2014 | Newman JC, 2014, DIABETES RES CLIN PR, V106, P173, DOI 10.1016/j.diabres.2014.08.009 |
| 746 | 6 | 2014 | Wu SY, 2014, CARDIOVASC THER, V32, P147, DOI 10.1111/1755-5922.12075 |
| 747 | 6 | 2014 | Roumie CL, 2014, JAMA-J AM MED ASSOC, V311, P2288, DOI 10.1001/jama.2014.4312 |
| 748 | 6 | 2014 | Inzucchi SE, 2014, JAMA-J AM MED ASSOC, V312, P2668, DOI 10.1001/jama.2014.15298 |
| 749 | 6 | 2014 | Pitt B, 2014, NEW ENGL J MED, V370, P1383, DOI 10.1056/NEJMoa1313731 |
| 750 | 6 | 2014 | Scheen AJ, 2014, EXPERT OPIN DRUG MET, V10, P647, DOI 10.1517/17425255.2014.873788 |
| 751 | 6 | 2014 | Polidori D, 2014, DIABETOLOGIA, V57, P891, DOI 10.1007/s00125-014-3196-x |
| 752 | 6 | 2014 | Holman RR, 2014, AM HEART J, V168, P23, DOI 10.1016/j.ahj.2014.03.021 |
| 753 | 6 | 2014 | **CentersforDiseaseControlandPrevention, 2014, NAT DIAB STAT REP ES, V0, P0 |
| 754 | 6 | 2014 | Kahn SE, 2014, LANCET, V383, P1068, DOI 10.1016/S0140-6736(13)62154-6 |
| 755 | 6 | 2014 | Hansen L, 2014, ENDOCR PRACT, V20, P1187, DOI 10.4158/EP14489.OR |
| 756 | 6 | 2013 | Sarma S, 2013, EUR J HEART FAIL, V15, P194, DOI 10.1093/eurjhf/hfs153 |
| 757 | 6 | 2013 | Scheen AJ, 2013, NAT REV CARDIOL, V10, P73, DOI 10.1038/nrcardio.2012.183 |
| 758 | 6 | 2013 | Ferrannini E, 2013, DIABETES CARE, V36, P1260, DOI 10.2337/dc12-1503 |
| 759 | 6 | 2013 | Nathan DM, 2013, DIABETES CARE, V36, P2254, DOI 10.2337/dc13-0356 |
| 760 | 6 | 2013 | Elkinson S, 2013, DRUGS, V73, P979, DOI 10.1007/s40265-013-0064-9 |
| 761 | 6 | 2013 | Groop PH, 2013, DIABETES CARE, V36, P3460, DOI 10.2337/dc13-0323 |
| 762 | 6 | 2013 | Miao Z, 2013, DRUG METAB DISPOS, V41, P445, DOI 10.1124/dmd.112.049551 |
| 763 | 6 | 2013 | DeFronzo RA, 2013, DIABETES CARE, V36, P0, DOI 10.2337/dcS13-2011 |
| 764 | 6 | 2013 | Taylor SR, 2013, PHARMACOTHERAPY, V33, P984, DOI 10.1002/phar.1303 |
| 765 | 6 | 2012 | Roumie CL, 2012, ANN INTERN MED, V157, P601, DOI 10.7326/0003-4819-157-9-201211060-00003 |
| 766 | 6 | 2012 | Boussageon R, 2012, PLOS MED, V9, P0, DOI 10.1371/journal.pmed.1001204 |
| 767 | 6 | 2012 | Rieg T, 2012, AM J PHYSIOL-RENAL, V303, P0, DOI 10.1152/ajprenal.00259.2012 |
| 768 | 6 | 2012 | Abdul-Ghani MA, 2012, CURR DIABETES REP, V12, P230, DOI 10.1007/s11892-012-0275-6 |
| 769 | 6 | 2012 | Kim Y, 2012, DIABET METAB SYND OB, V5, P313, DOI 10.2147/DMSO.S22545 |
| 770 | 6 | 2011 | Mather A, 2011, KIDNEY INT, V79, P0, DOI 10.1038/ki.2010.509 |
| 771 | 6 | 2011 | Rosenstock J, 2011, DIABETES, V60, P0 |
| 772 | 6 | 2010 | From AM, 2010, J AM COLL CARDIOL, V55, P300, DOI 10.1016/j.jacc.2009.12.003 |
| 773 | 6 | 2010 | Graham DJ, 2010, JAMA-J AM MED ASSOC, V304, P411, DOI 10.1001/jama.2010.920 |
| 774 | 6 | 2009 | Banerjee SK, 2009, CARDIOVASC RES, V84, P111, DOI 10.1093/cvr/cvp190 |
| 775 | 5 | 2019 | Neuen BL, 2019, J AM SOC NEPHROL, V30, P2229, DOI 10.1681/ASN.2019010064 |
| 776 | 5 | 2019 | Nielsen R, 2019, CIRCULATION, V139, P2129, DOI 10.1161/CIRCULATIONAHA.118.036459 |
| 777 | 5 | 2019 | Packer M, 2019, CIRCULATION, V140, P443, DOI 10.1161/CIRCULATIONAHA.119.040909 |
| 778 | 5 | 2019 | Berg DD, 2019, CIRCULATION, V140, P1569, DOI 10.1161/CIRCULATIONAHA.119.042685 |
| 779 | 5 | 2019 | Lingvay I, 2019, LANCET DIABETES ENDO, V7, P834, DOI 10.1016/S2213-8587(19)30311-0 |
| 780 | 5 | 2019 | **AmericanDiabetesAssociation, 2019, DIABETES CARE, V42, P0, DOI 10.2337/dc19-S010 |
| 781 | 5 | 2019 | Oh CM, 2019, KOREAN CIRC J, V49, P1183, DOI 10.4070/kcj.2019.0180 |
| 782 | 5 | 2019 | Sarafidis P, 2019, NEPHROL DIAL TRANSPL, V34, P208, DOI 10.1093/ndt/gfy407 |
| 783 | 5 | 2019 | Arnett DK, 2019, J AM COLL CARDIOL, V74, P1376, DOI 10.1016/j.jacc.2019.03.009 |
| 784 | 5 | 2019 | Sternlicht H, 2019, CURR HYPERTENS REP, V21, P0, DOI 10.1007/s11906-019-0920-4 |
| 785 | 5 | 2019 | Donnan JR, 2019, BMJ OPEN, V9, P0, DOI 10.1136/bmjopen-2018-022577 |
| 786 | 5 | 2019 | Hess DA, 2019, CELL METAB, V30, P609, DOI 10.1016/j.cmet.2019.08.015 |
| 787 | 5 | 2019 | Cooper ME, 2019, AM J KIDNEY DIS, V74, P713, DOI 10.1053/j.ajkd.2019.03.432 |
| 788 | 5 | 2019 | Osataphan S, 2019, JCI INSIGHT, V4, P0, DOI 10.1172/jci.insight.123130 |
| 789 | 5 | 2019 | Abdurrachim D, 2019, DIABETES OBES METAB, V21, P357, DOI 10.1111/dom.13536 |
| 790 | 5 | 2019 | Benjamin EJ, 2019, CIRCULATION, V139, P0, DOI 10.1161/CIR.0000000000000659 |
| 791 | 5 | 2019 | Savarese G, 2019, CIRCULATION, V139, P1458, DOI 10.1161/CIRCULATIONAHA.118.038339 |
| 792 | 5 | 2018 | Umino H, 2018, SCI REP-UK, V8, P0, DOI 10.1038/s41598-018-25054-y |
| 793 | 5 | 2018 | Kluger AY, 2018, REV CARDIOVASC MED, V19, P41, DOI 10.31083/j.rcm.2018.02.907 |
| 794 | 5 | 2018 | Shibuya T, 2018, DIABETES OBES METAB, V20, P438, DOI 10.1111/dom.13061 |
| 795 | 5 | 2018 | Rieg T, 2018, DIABETOLOGIA, V61, P2079, DOI 10.1007/s00125-018-4654-7 |
| 796 | 5 | 2018 | McCrimmon RJ, 2018, DIABETOLOGIA, V61, P2126, DOI 10.1007/s00125-018-4671-6 |
| 797 | 5 | 2018 | Yang WY, 2018, DIABETES CARE, V41, P917, DOI 10.2337/dci18-0007 |
| 798 | 5 | 2018 | Nespoux J, 2018, CLIN SCI, V132, P1329, DOI 10.1042/CS20171298 |
| 799 | 5 | 2017 | Monami M, 2017, ACTA DIABETOL, V54, P19, DOI 10.1007/s00592-016-0892-7 |
| 800 | 5 | 2017 | Bain S, 2017, DIABETES OBES METAB, V19, P329, DOI 10.1111/dom.12821 |
| 801 | 5 | 2017 | Sorli C, 2017, LANCET DIABETES ENDO, V5, P251, DOI 10.1016/S2213-8587(17)30013-X |
| 802 | 5 | 2017 | Perrone-Filardi P, 2017, INT J CARDIOL, V241, P450, DOI 10.1016/j.ijcard.2017.03.089 |
| 803 | 5 | 2016 | Ceriello A, 2016, CARDIOVASC DIABETOL, V15, P0, DOI 10.1186/s12933-016-0440-3 |
| 804 | 5 | 2016 | Piepoli MF, 2016, ATHEROSCLEROSIS, V252, P207, DOI 10.1016/j.atherosclerosis.2016.05.037 |
| 805 | 5 | 2016 | Cheng STW, 2016, PLOS ONE, V11, P0, DOI 10.1371/journal.pone.0147391 |
| 806 | 5 | 2016 | Avogaro A, 2016, CARDIOVASC DIABETOL, V15, P0, DOI 10.1186/s12933-016-0431-4 |
| 807 | 5 | 2015 | Abdelmoneim AS, 2015, DIABETES OBES METAB, V17, P523, DOI 10.1111/dom.12456 |
| 808 | 5 | 2015 | Abdul-Ghani M, 2015, DIABETES CARE, V38, P373, DOI 10.2337/dc14-2517 |
| 809 | 5 | 2015 | **EuropeanMedicinesAgency, 2015, REV DIAB MED CALL SG, V0, P0 |
| 810 | 5 | 2015 | Drechsler C, 2015, J AM SOC NEPHROL, V26, P2213, DOI 10.1681/ASN.2014010093 |
| 811 | 5 | 2015 | Lapuerta P, 2015, DIABETES VASC DIS RE, V12, P101, DOI 10.1177/1479164114563304 |
| 812 | 5 | 2015 | Bakris GL, 2015, JAMA-J AM MED ASSOC, V314, P884, DOI 10.1001/jama.2015.10081 |
| 813 | 5 | 2015 | US Food and Drug Administration, 2015, FDA DRUG SAF COMM FD, V0, P0 |
| 814 | 5 | 2015 | Fadini GP, 2015, EUR HEART J, V36, P2454, DOI 10.1093/eurheartj/ehv301 |
| 815 | 5 | 2015 | Araki E, 2015, DIABETES OBES METAB, V17, P665, DOI 10.1111/dom.12464 |
| 816 | 5 | 2014 | Abdul-Ghani MA, 2014, J INTERN MED, V276, P352, DOI 10.1111/joim.12244 |
| 817 | 5 | 2014 | Jordan J, 2014, DIABETES, V63, P0 |
| 818 | 5 | 2014 | Wilding JPH, 2014, METABOLISM, V63, P1228, DOI 10.1016/j.metabol.2014.06.018 |
| 819 | 5 | 2014 | Scheen AJ, 2014, DIABETES METAB, V40, P176, DOI 10.1016/j.diabet.2014.03.004 |
| 820 | 5 | 2013 | Amer Diabet Assoc, 2013, DIABETES CARE, V36, P0, DOI 10.2337/dc13-S011 |
| 821 | 5 | 2013 | Lamos EM, 2013, EXPERT OPIN DRUG MET, V9, P763, DOI 10.1517/17425255.2013.791282 |
| 822 | 5 | 2013 | Bays H, 2013, DIABETES THER, V4, P195, DOI 10.1007/s13300-013-0042-y |
| 823 | 5 | 2013 | Fonseca VA, 2013, J DIABETES COMPLICAT, V27, P268, DOI 10.1016/j.jdiacomp.2012.11.005 |
| 824 | 5 | 2013 | Hirshberg B, 2013, DIABETES CARE, V36, P0, DOI 10.2337/dcS13-2041 |
| 825 | 5 | 2013 | Seman L, 2013, CLIN PHARM DRUG DEV, V2, P152, DOI 10.1002/cpdd.16 |
| 826 | 5 | 2013 | Nagata T, 2013, BRIT J PHARMACOL, V170, P519, DOI 10.1111/bph.12269 |
| 827 | 5 | 2013 | Kanada S, 2013, J DIABETES INVEST, V4, P613, DOI 10.1111/jdi.12110 |
| 828 | 5 | 2012 | Ruggenenti P, 2012, DIABETES CARE, V35, P2061, DOI 10.2337/dc11-2189 |
| 829 | 5 | 2012 | Plosker GL, 2012, DRUGS, V72, P2289, DOI 10.2165/11209910-000000000-00000 |
| 830 | 5 | 2012 | Woerle HJ, 2012, DIABETES, V61, P2349, DOI 10.2337/db11-1701 |
| 831 | 5 | 2011 | Danaei G, 2011, LANCET, V378, P31, DOI 10.1016/S0140-6736(11)60679-X |
| 832 | 5 | 2011 | Kashiwagi A, 2011, DIABETOLOGIA, V54, P0 |
| 833 | 5 | 2011 | Del Prato S, 2011, DIABETES OBES METAB, V13, P258, DOI 10.1111/j.1463-1326.2010.01350.x |
| 834 | 5 | 2011 | Bunck MC, 2011, DIABETES CARE, V34, P2041, DOI 10.2337/dc11-0291 |
| 835 | 5 | 2011 | Deacon CF, 2011, DIABETES OBES METAB, V13, P7, DOI 10.1111/j.1463-1326.2010.01306.x |
| 836 | 5 | 2010 | DeFronzo RA, 2010, DIABETOLOGIA, V53, P1270, DOI 10.1007/s00125-010-1684-1 |
| 837 | 5 | 2010 | Neumiller JJ, 2010, DRUGS, V70, P377, DOI 10.2165/11318680-000000000-00000 |
| 838 | 5 | 2009 | Buse JB, 2009, LANCET, V374, P39, DOI 10.1016/S0140-6736(09)60659-0 |
| 839 | 5 | 2006 | Drucker DJ, 2006, LANCET, V368, P1696, DOI 10.1016/S0140-6736(06)69705-5 |
| 840 | 4 | 2015 | Davies MJ, 2015, JAMA-J AM MED ASSOC, V314, P687, DOI 10.1001/jama.2015.9676 |
| 841 | 4 | 2015 | Hamouda NN, 2015, MOL CELL BIOCHEM, V400, P57, DOI 10.1007/s11010-014-2262-5 |
| 842 | 4 | 2014 | van Haalen HGM, 2014, CLIN DRUG INVEST, V34, P135, DOI 10.1007/s40261-013-0155-0 |
| 843 | 4 | 2014 | Egan AG, 2014, NEW ENGL J MED, V370, P794, DOI 10.1056/NEJMp1314078 |
| 844 | 4 | 2014 | Chao Edward C, 2014, CLIN DIABETES, V32, P4, DOI 10.2337/diaclin.32.1.4 |
| 845 | 4 | 2014 | Sinclair A, 2014, BMC ENDOCR DISORD, V14, P0, DOI 10.1186/1472-6823-14-37 |
| 846 | 4 | 2014 | Scheen AJ, 2014, CLIN PHARMACOKINET, V53, P295, DOI 10.1007/s40262-013-0128-8 |
| 847 | 4 | 2014 | Younis FM, 2014, DIABETES, V63, P0 |
| 848 | 4 | 2014 | Grandy S, 2014, DIABETES OBES METAB, V16, P645, DOI 10.1111/dom.12263 |
| 849 | 4 | 2013 | Barnett AH, 2013, POSTGRAD MED, V125, P92, DOI 10.3810/pgm.2013.09.2698 |
| 850 | 4 | 2013 | [Anonymous], 2013, INV CAN, V0, P0 |
| 851 | 4 | 2013 | Macha S, 2013, DIABETES OBES METAB, V15, P316, DOI 10.1111/dom.12028 |
| 852 | 4 | 2013 | Tikkanen I, 2013, DIABETOLOGIA, V56, P0 |
| 853 | 4 | 2013 | Ring A, 2013, CARDIOVASC DIABETOL, V12, P0, DOI 10.1186/1475-2840-12-70 |
| 854 | 4 | 2013 | Sarashina A, 2013, DRUG METAB PHARMACOK, V28, P213, DOI 10.2133/dmpk.DMPK-12-RG-082 |
| 855 | 4 | 2013 | Colosia AD, 2013, DIABETES METAB SYNDR, V6, P327, DOI 10.2147/DMSO.S51325 |
| 856 | 4 | 2013 | Powell DR, 2013, AM J PHYSIOL-ENDOC M, V304, P0, DOI 10.1152/ajpendo.00439.2012 |
| 857 | 4 | 2013 | Macha S, 2013, CLIN DRUG INVEST, V33, P351, DOI 10.1007/s40261-013-0068-y |
| 858 | 4 | 2013 | Ridderstrale M, 2013, CARDIOVASC DIABETOL, V12, P0, DOI 10.1186/1475-2840-12-129 |
| 859 | 4 | 2013 | **InternationalDiabetesFederation, 2013, IDF DIABETES ATLAS, V0, P0 |
| 860 | 4 | 2013 | Imamura A, 2013, DIABETES THER, V4, P41, DOI 10.1007/s13300-012-0016-5 |
| 861 | 4 | 2013 | Macha S, 2013, CLIN THER, V35, P226, DOI 10.1016/j.clinthera.2013.02.015 |
| 862 | 4 | 2012 | Whaley JM, 2012, DIABET METAB SYND OB, V5, P135, DOI 10.2147/DMSO.S22503 |
| 863 | 4 | 2012 | Nicolle LE, 2012, CURR MED RES OPIN, V28, P1167, DOI 10.1185/03007995.2012.689956 |
| 864 | 4 | 2011 | Tahrani AA, 2011, LANCET, V378, P182, DOI 10.1016/S0140-6736(11)60207-9 |
| 865 | 4 | 2010 | Macdonald FR, 2010, DIABETES OBES METAB, V12, P1004, DOI 10.1111/j.1463-1326.2010.01291.x |
| 866 | 4 | 2009 | Tzoulaki I, 2009, BMJ-BRIT MED J, V339, P0, DOI 10.1136/bmj.b4731 |
| 867 | 4 | 2009 | Garber A, 2009, LANCET, V373, P473, DOI 10.1016/S0140-6736(08)61246-5 |
| 868 | 4 | 2008 | Meng W, 2008, J MED CHEM, V51, P1145, DOI 10.1021/jm701272q |
| 869 | 4 | 2007 | Palmer AJ, 2007, DIABETES CARE, V30, P1638, DOI 10.2337/dc07-9919 |
| 870 | 4 | 2007 | Katsuno K, 2007, J PHARMACOL EXP THER, V320, P323, DOI 10.1124/jpet.106.110296 |
| 871 | 3 | 2014 | **CentersforDiseaseControlandPrevention, 2014, NAT DIAB STAT REP, V0, P0 |
| 872 | 3 | 2014 | Bell DSH, 2014, AM J CASE REP, V15, P152, DOI 10.12659/AJCR.890626 |
| 873 | 3 | 2014 | Lamos EM, 2014, EXPERT OPIN INV DRUG, V23, P875, DOI 10.1517/13543784.2014.909407 |
| 874 | 3 | 2013 | Forbes JM, 2013, PHYSIOL REV, V93, P137, DOI 10.1152/physrev.00045.2011 |
| 875 | 3 | 2013 | Garber AJ, 2013, ENDOCR PRACT, V19, P100, DOI 10.4158/EP12325.OR |
| 876 | 3 | 2013 | **NationalInstituteforHealthandCareExcellence, 2013, GUID METH TECHN APPR, V0, P0 |
| 877 | 3 | 2013 | Kasichayanula S, 2013, DIABETES OBES METAB, V15, P280, DOI 10.1111/dom.12024 |
| 878 | 3 | 2013 | Garber AJ, 2013, ENDOCR PRACT, V19, P536, DOI 10.4158/EP13176.CS |
| 879 | 3 | 2013 | Schernthaner G, 2013, DIABETES CARE, V0, P0 |
| 880 | 3 | 2012 | Wilding J, 2012, DIABETES, V61, P0 |
| 881 | 3 | 2012 | Brand T, 2012, ADV THER, V29, P889, DOI 10.1007/s12325-012-0055-3 |
| 882 | 3 | 2012 | Luippold G, 2012, DIABETES OBES METAB, V14, P601, DOI 10.1111/j.1463-1326.2012.01569.x |
| 883 | 3 | 2012 | Ohtake Y, 2012, J MED CHEM, V55, P7828, DOI 10.1021/jm300884k |
| 884 | 3 | 2012 | [Anonymous], 2012, DIABETES CARE, V35, P0 |
| 885 | 3 | 2012 | Goto K, 2012, DIABETES, V61, P0 |
| 886 | 3 | 2012 | Ptaszynska A, 2012, DIABETES, V61, P0 |
| 887 | 3 | 2012 | Osorio H, 2012, OXID MED CELL LONGEV, V2012, P0, DOI 10.1155/2012/542042 |
| 888 | 3 | 2012 | Burki TK, 2012, LANCET, V379, P507, DOI 10.1016/S0140-6736(12)60216-5 |
| 889 | 3 | 2011 | Veltkamp SA, 2011, CLIN DRUG INVEST, V31, P839, DOI 10.2165/11594330-000000000-00000 |
| 890 | 3 | 2011 | **CentersforDiseaseControlandPrevention, 2011, NAT DIAB FACT SHEET, V0, P0 |
| 891 | 3 | 2011 | Kasichayanula S, 2011, DIABETES OBES METAB, V13, P770, DOI 10.1111/j.1463-1326.2011.01397.x |
| 892 | 3 | 2011 | Kasichayanula S, 2011, DIABETES OBES METAB, V13, P357, DOI 10.1111/j.1463-1326.2011.01359.x |
| 893 | 3 | 2011 | Kohan D, 2011, J AM SOC NEPHROL S, V22, P0 |
| 894 | 3 | 2011 | Kasichayanula S, 2011, CLIN THER, V33, P1798, DOI 10.1016/j.clinthera.2011.09.011 |
| 895 | 3 | 2011 | Beloto-Silva O, 2011, J MEMBRANE BIOL, V239, P157, DOI 10.1007/s00232-010-9334-6 |
| 896 | 3 | 2010 | Schernthaner G, 2010, DIABETOLOGIA, V53, P1258, DOI 10.1007/s00125-010-1702-3 |
| 897 | 3 | 2010 | Obermeier M, 2010, DRUG METAB DISPOS, V38, P405, DOI 10.1124/dmd.109.029165 |
| 898 | 3 | 2010 | Ferrannini E, 2010, DIABETOLOGIA S1, V53, P0 |
| 899 | 3 | 2010 | Ferrannini E, 2010, NEPHROL DIAL TRANSPL, V25, P2041, DOI 10.1093/ndt/gfq249 |
| 900 | 3 | 2010 | DeFronzo RA, 2010, AM J MED, V123, P0, DOI 10.1016/j.amjmed.2009.12.008 |
| 901 | 3 | 2010 | Sha S, 2010, DIABETES S1, V59, P0 |
| 902 | 3 | 2010 | Garg R, 2010, DIABETES CARE, V33, P2349, DOI 10.2337/dc10-0482 |
| 903 | 3 | 2010 | Derosa G, 2010, METABOLISM, V59, P887, DOI 10.1016/j.metabol.2009.10.007 |
| 904 | 3 | 2010 | Nair S, 2010, J CLIN ENDOCR METAB, V95, P34, DOI 10.1210/jc.2009-0473 |
| 905 | 3 | 2009 | Rodbard HW, 2009, ENDOCR PRACT, V15, P540, DOI 10.4158/EP.15.6.540 |
| 906 | 3 | 2009 | Cheung BMY, 2009, AM J MED, V122, P443, DOI 10.1016/j.amjmed.2008.09.047 |
| 907 | 3 | 2009 | Marrett E, 2009, DIABETES OBES METAB, V11, P1138, DOI 10.1111/j.1463-1326.2009.01123.x |
| 908 | 3 | 2008 | Drucker DJ, 2008, LANCET, V372, P1240, DOI 10.1016/S0140-6736(08)61206-4 |
| 909 | 3 | 2008 | Baboolal K, 2008, NEPHROL DIAL TRANSPL, V23, P1982, DOI 10.1093/ndt/gfm870 |
| 910 | 3 | 2008 | Caulfield MJ, 2008, PLOS MED, V5, P1509, DOI 10.1371/journal.pmed.0050197 |
| 911 | 3 | 2008 | Fujimori Y, 2008, J PHARMACOL EXP THER, V327, P268, DOI 10.1124/jpet.108.140210 |
| 912 | 3 | 2008 | Moretto TJ, 2008, CLIN THER, V30, P1448, DOI 10.1016/j.clinthera.2008.08.006 |
| 913 | 3 | 2008 | Food and Drug Administration, 2008, GUID IND DIAB MELL E, V0, P0 |
| 914 | 3 | 2007 | Lee YJ, 2007, KIDNEY INT, V72, P0, DOI 10.1038/sj.ki.5002383 |
| 915 | 3 | 2006 | Evans JMM, 2006, DIABETOLOGIA, V49, P930, DOI 10.1007/s00125-006-0176-9 |
| 916 | 2 | 2013 | Barnett AH, 2013, DIABETES, V62, P0 |
| 917 | 2 | 2012 | Dobbins RL, 2012, DIABETES OBES METAB, V14, P15, DOI 10.1111/j.1463-1326.2011.01462.x |
| 918 | 2 | 2012 | Cefalu WT, 2012, DIABETES, V61, P0 |
| 919 | 2 | 2012 | Cefalu WT, 2012, DIABETES S1A, V61, P0 |
| 920 | 2 | 2012 | Devineni D MP, 2012, CLIN PHARM DRUG DEV, V1, P81 |
| 921 | 2 | 2012 | Currie CJ, 2012, DIABETES CARE, V35, P1279, DOI 10.2337/dc11-1277 |
| 922 | 2 | 2011 | Devineni D, 2011, AM DIAB ASS 71 SCI S, V0, P0 |
| 923 | 2 | 2011 | Carlson Glenn F, 2011, DIABETES THER, V2, P123, DOI 10.1007/s13300-011-0003-2 |
| 924 | 2 | 2011 | Calado J, 2011, KIDNEY INT, V79, P0, DOI 10.1038/ki.2010.510 |
| 925 | 2 | 2011 | Ferrannini E, 2011, DIABETES, V60, P695, DOI 10.2337/db10-1667 |
| 926 | 2 | 2010 | Cohen D, 2010, BRIT MED J, V341, P0, DOI 10.1136/bmj.c4848 |
| 927 | 2 | 2010 | Chen J, 2010, RADIAT PROT DOSIM, V142, P1, DOI 10.1093/rpd/ncq205 |
| 928 | 2 | 2010 | Alvarsson Michael, 2010, REV DIABET STUD, V7, P225, DOI 10.1900/RDS.2010.7.225 |
| 929 | 2 | 2009 | Eeg-Olofsson K, 2009, DIABETOLOGIA, V52, P65, DOI 10.1007/s00125-008-1190-x |
| 930 | 2 | 2009 | DeSouza C, 2009, NAT REV DRUG DISCOV, V8, P361, DOI 10.1038/nrd2872 |
| 931 | 2 | 2008 | Fox CS, 2008, DIABETES CARE, V31, P1582, DOI 10.2337/dc08-0025 |
| 932 | 2 | 2008 | Beest FJAP, 2008, CURR MED RES OPIN, V24, P2523 |
| 933 | 2 | 2008 | Amiel SA, 2008, DIABETIC MED, V25, P245, DOI 10.1111/j.1464-5491.2007.02341.x |
| 934 | 2 | 2007 | Fox CS, 2007, CIRCULATION, V115, P1544, DOI 10.1161/CIRCULATIONAHA.106.658948 |
| 935 | 2 | 2007 | Diamond GA, 2007, ANN INTERN MED, V147, P578, DOI 10.7326/0003-4819-147-8-200710160-00182 |
| 936 | 2 | 2007 | Gregg EW, 2007, PREV MED, V45, P348, DOI 10.1016/j.ypmed.2007.07.020 |
| 937 | 2 | 2007 | Foley RN, 2007, J AM SOC NEPHROL, V18, P2644, DOI 10.1681/ASN.2007020220 |
| 938 | 2 | 2007 | Gangji AS, 2007, DIABETES CARE, V30, P389, DOI 10.2337/dc06-1789 |

**Note:** SGLT2: Sodium Glucose Cotransporter 2. CV: cardiovascular
