# Supplementary material for: Bibliometric Study of Sodium Glucose Cotransporter 2 Inhibitors in Cardiovascular Research
Source: Front Pharmacol. 2020 Sep 15;11:561494. doi: 10.3389/fphar.2020.561494 (PMC7522576; doi:10.3389/fphar.2020.561494)
Supplement: Supplementary file 10 [file Table_10.docx]

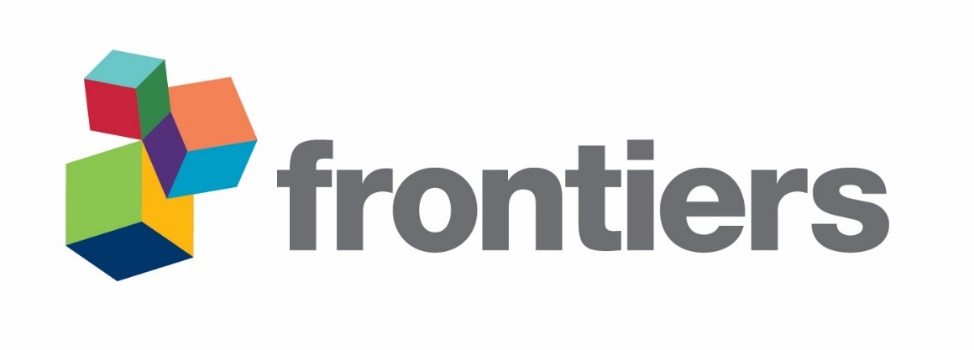
Supplementary Material

Supplementary Table 10. The cocited clusters of SGLT2 inhibitors in CV research.

| **Cluster ID** | **Size** | **Silhouette** | **Mean year** | **Top terms (log-likelihood ratio, p-level)** |
| --- | --- | --- | --- | --- |
| 0 | 91 | 0.692 | 2015 | atherosclerotic cardiovascular event (129.17, 1.0E-4); randomised cardiovascular outcome trial (129.17, 1.0E-4); stroke risk (125.58, 1.0E-4); diabetes drug (125.58, 1.0E-4); conventional glucose-lowering strategies (125.58, 1.0E-4); emerging glucose-lowering therapy (121.98, 1.0E-4); concomitant change (118.39, 1.0E-4); conventional cardiovascular risk factor level (118.39, 1.0E-4); receptor agonist (114.8, 1.0E-4); ongoing mechanistic trial (114.8, 1.0E-4); cardiac disease (107.61, 1.0E-4); risk stratification tool (104.02, 1.0E-4); diabetes clinic (104.02, 1.0E-4); acute decompensated heart failure (100.43, 1.0E-4); multicentre pilot study (100.43, 1.0E-4); pharmacological priorities (96.84, 1.0E-4); advanced type (96.84, 1.0E-4); diabetes treatment sequence (93.25, 1.0E-4); step therapy policies (93.25, 1.0E-4); contemporary choice (89.66, 1.0E-4); lowering agent (89.66, 1.0E-4); developing countries (86.07, 1.0E-4); danish user (82.48, 1.0E-4); severe hypoglycemia (82.48, 1.0E-4); triple therapy (82.48, 1.0E-4); major adverse cardiovascular event (82.48, 1.0E-4); metformin initiator (78.89, 1.0E-4); japanese working-age patient (78.89, 1.0E-4); dosing pattern (78.89, 1.0E-4); acute heart failure (75.3, 1.0E-4); bidirectional relationship (71.71, 1.0E-4); affecting treatment (71.71, 1.0E-4); post-transplant diabetes mellitus (71.71, 1.0E-4); preexisting liver disease-a (71.71, 1.0E-4); blinded endpoint parallel-group comparison study (68.12, 1.0E-4); nocturnal blood pressure (68.12, 1.0E-4); glucose-lowering medication (67.84, 1.0E-4); heart failure prevention (64.54, 1.0E-4); sweet spot (64.54, 1.0E-4); all-cause mortality (58.23, 1.0E-4); recent cardiovascular outcome trial (57.9, 1.0E-4); diabetic mellitus patient (57.36, 1.0E-4); left ventricular longitudinal function (57.36, 1.0E-4); positive effect (57.36, 1.0E-4); clinical outcome (56.12, 1.0E-4); oral antidiabetic drug (53.63, 1.0E-4); pharmacological management (53.3, 1.0E-4); heart failure patient (51.61, 1.0E-4); cardiovascular risk reduction (49.16, 1.0E-4); cardiometabolic benefit (46.6, 1.0E-4); prescribing glucose-lowering therapy (46.6, 1.0E-4); renal benefit (43.01, 1.0E-4); long-standing type (43.01, 1.0E-4); diabetes mellitus (42.42, 1.0E-4); glucose-lowering drug (40.86, 1.0E-4); cohort study (39.74, 1.0E-4); nationwide population-based longitudinal cohort study (35.84, 1.0E-4); new-onset arrhythmia (35.84, 1.0E-4); heart failure (35.37, 1.0E-4); new glp-1 receptor agonist (32.25, 1.0E-4); baseline body mass index (28.67, 1.0E-4); chronic heart failure (27.43, 1.0E-4); diabetes medication (25.64, 1.0E-4); evidence map (25.08, 1.0E-4); umbrella review (25.08, 1.0E-4); new treatment option (23.38, 1.0E-4); cardiometabolic risk factor (23.36, 1.0E-4); clinical practice (22.19, 1.0E-4); empagliflozin monotherapy (21.92, 1.0E-4); combination therapy (21.77, 1.0E-4); diabetes management (21.73, 1.0E-4); diabetes treatment (20.82, 1.0E-4); sglt2 inhibitor (20.22, 1.0E-4); cardiovascular outcome (19.66, 1.0E-4); diuretic effect (19.36, 1.0E-4); classic diuretics (19.36, 1.0E-4); empa-reg outcome (19.2, 1.0E-4); potential place (18.63, 1.0E-4); specific mechanism (17.91, 1.0E-4); glp-1 receptor agonist therapy (17.9, 1.0E-4); sglt1 inhibition boon (17.9, 1.0E-4); diabetes-associated cardiomyopathy (17.9, 1.0E-4); safety outcome (17.9, 1.0E-4); combination sglt2 inhibitor (17.9, 1.0E-4); complementary approach (17.9, 1.0E-4); antihyperglycemic agent therapy (17.17, 1.0E-4); adult patient (17.17, 1.0E-4); korean diabetes association (17.17, 1.0E-4); new oral glucose-lowering agent (16.8, 1.0E-4); metabolic border (16.44, 1.0E-4); new oral hypoglycemic agent (16.44, 1.0E-4); empa-reg outcome trial (16.21, 1.0E-4); fracture risk (16.12, 1.0E-4); evidence-based practice approach (15.71, 1.0E-4); natural history (15.71, 1.0E-4); placebo-controlled studies (15.34, 1.0E-4); bone mineral density (14.97, 0.001); glycemic control (14.95, 0.001); sglt2 inhibition (14.67, 0.001); true renoprotection (14.33, 0.001) |
| 1 | 88 | 0.717 | 2017 | diabetic cardiomyopathy (194.84, 1.0E-4); independent modulation (132.95, 1.0E-4); diabetic heart (132.95, 1.0E-4); organellar stress (132.95, 1.0E-4); doxorubicin-induced myocardial dysfunction (128.19, 1.0E-4); inhibitor dapagliflozin (123.44, 1.0E-4); coronary endothelium (118.69, 1.0E-4); diastolic dysfunction (118.69, 1.0E-4); non-diabetic model (118.69, 1.0E-4); different effect (114.12, 1.0E-4); epicardial fat (109.18, 1.0E-4); coronary artery disease patient (109.18, 1.0E-4); dapagliflozin treatment (109.18, 1.0E-4); signaling pathway (104.42, 1.0E-4); reperfusion injury (104.42, 1.0E-4); acute myocardial infarction-the emmy trial (99.67, 1.0E-4); left ventricular dysfunction (94.92, 1.0E-4); renal structure (94.92, 1.0E-4); non-diabetic rat (94.92, 1.0E-4); diabetic patient (88.1, 1.0E-4); cardiac fibrosis (80.67, 1.0E-4); ventricular hemodynamics (80.67, 1.0E-4); hypertensive heart failure rat (80.67, 1.0E-4); adp-dependent activation (75.91, 1.0E-4); human myeloid angiogenic cell (75.91, 1.0E-4); potential relevance (75.91, 1.0E-4); human platelet (75.91, 1.0E-4); diabetic rat (71.16, 1.0E-4); possible underlying mechanism (71.16, 1.0E-4); glp1 analog (71.16, 1.0E-4); comparative study (71.16, 1.0E-4); insulin resistance (66.42, 1.0E-4); ketone utilization (66.42, 1.0E-4); myocardial infarction (65.38, 1.0E-4); heart failure (63.81, 1.0E-4); cardiac function (63.68, 1.0E-4); antidiabetic medication (61.67, 1.0E-4); statin paradox (61.67, 1.0E-4); left ventricular function (56.92, 1.0E-4); atrial fibrillation (53.07, 1.0E-4); inhibitor empagliflozin (53.06, 1.0E-4); novel hypothesis (52.18, 1.0E-4); potential mechanism (48.96, 1.0E-4); inhibitor-related cardiovascular benefit (47.43, 1.0E-4); sodium-glucose co-transporter (46.64, 1.0E-4); glucose-lowering drug (42.8, 1.0E-4); current evidence (39.71, 1.0E-4); diabetes mellitus (38.86, 1.0E-4); protective effect (37.94, 1.0E-4); renal dysfunction (37.94, 1.0E-4); ejection fraction (36.75, 1.0E-4); 21-dependent traf3ip2 induction (33.19, 1.0E-4); to-mesenchymal transition (33.19, 1.0E-4); reck suppression (33.19, 1.0E-4); high glucose-induced oxidative stress (33.19, 1.0E-4); sglt2 inhibitor empagliflozin (28.52, 1.0E-4); discordant effect (28.45, 1.0E-4); epicardial adipose tissue inflammation (28.45, 1.0E-4); enhancing myocardial energetics (23.7, 1.0E-4); adverse left (23.7, 1.0E-4); nondiabetic heart failure (23.7, 1.0E-4); ventricular remodeling (23.7, 1.0E-4); load-independent effect (18.96, 1.0E-4); future direction (15.93, 1.0E-4); cardioprotective effect (15.69, 1.0E-4); sodium glucose (14.97, 0.001); adjunctive therapy (14.91, 0.001); blood pressure (14.24, 0.001); vascular endothelial function (14.22, 0.001); heart diseases (14.22, 0.001); reduced ejection fraction (14.16, 0.001); cardiovascular outcome (12.58, 0.001); sodium glucose co-transporter (12.4, 0.001); renal function (11.84, 0.001); empagliflozin monotherapy (11.77, 0.001); sglt-2 inhibitor (11.04, 0.001); cotransporter-2 inhibitor (10.78, 0.005); cardiovascular safety (10.37, 0.005); glp-1 receptor agonist (10, 0.005); murine model (9.48, 0.005); fatty acid oxidation (9.48, 0.005); exercise endurance capacity (9.48, 0.005); skeletal muscle (9.48, 0.005); empa-reg outcome (9.26, 0.005); drug classes (9.01, 0.005); new oral glucose-lowering agent (9.01, 0.005); pharmacological management (9.01, 0.005); clinical trial result (9.01, 0.005); cardiovascular risk reduction (8.66, 0.005); fracture resistance (8.43, 0.005); genetic ablation (8.43, 0.005); diabetic kidney disease risk (8.23, 0.005); sglt2 function (8.23, 0.005); pressure-dependent pathways-the progression (8.23, 0.005); diabetic kidney disease (8.15, 0.005); glucose-lowering agent (8.03, 0.005); tissue mineral density (8.03, 0.005); sodium-glucose co-transporter type (7.64, 0.01); renoprotective effect (7.64, 0.01); kidney disease (7.64, 0.01) |
| 2 | 75 | 0.634 | 2013 | clinical efficacy (175.75, 1.0E-4); 28-week extension (97.5, 1.0E-4); high baseline a1c (77.97, 1.0E-4); placebo-controlled study (72.75, 1.0E-4); preexisting cardiovascular disease (71.47, 1.0E-4); usual care (71.47, 1.0E-4); cotransporter-2 inhibitor (63.57, 1.0E-4); diabetes mellitus (52.08, 1.0E-4); current clinical evidence (51.96, 1.0E-4); renal safety (45.46, 1.0E-4); glucuretic effect (45.46, 1.0E-4); glycemic variation (38.96, 1.0E-4); blood pressure effect (32.46, 1.0E-4); sodium-glucose co-transport (32.46, 1.0E-4); sodium glucose (31.67, 1.0E-4); dapagliflozins effect (25.97, 1.0E-4); toxicological consideration (23.42, 1.0E-4); evaluating sglt2 inhibitor (21.96, 1.0E-4); sodium-glucose cotransporters type (21.4, 1.0E-4); drug-drug interaction (19.83, 1.0E-4); long-term maintenance (19.47, 1.0E-4); following co-administration (19.12, 1.0E-4); selective inhibitor (18.58, 1.0E-4); clinical pharmacokinetics (17.76, 1.0E-4); sodium-glucose co-transporter type (17.42, 1.0E-4); high-risk patient (16.72, 1.0E-4); healthy volunteer (15.22, 1.0E-4); glucose co-transporter (14.16, 0.001); peptidase-4 inhibitor (13.04, 0.001); complementing insulin therapy (12.1, 0.001); transporter-2 inhibitor (11.1, 0.001); sglt2 inhibitor (9.91, 0.005); sglt2 inhibition (9.83, 0.005); renal protection (9.62, 0.005); evidence-based practice approach (9.31, 0.005); natural history (9.31, 0.005); diabetes treatment (8.95, 0.005); new oral glucose-lowering agent (8.59, 0.005); sglt1 inhibition boon (7.94, 0.005); diabetes-associated cardiomyopathy (7.94, 0.005); sodium-glucose cotransporter inhibition (7.81, 0.01); new treatment option (7.62, 0.01); potential place (7.53, 0.01); extraglycemic effect (7.36, 0.01); glucose-lowering drug (6.92, 0.01); physiological responses (6.61, 0.05); sedentary overweight (6.61, 0.05); obese adult human (6.61, 0.05); cardiovascular safety (6.52, 0.05); new sodium-glucose cotransporter (6.49, 0.05); dietary counselling (6.36, 0.05); randomised controlled trial (6.27, 0.05); sustain-6 trial (6.22, 0.05); expert recommendation (6.13, 0.05); asian perspective (6.13, 0.05); abdominal obesity (6.13, 0.05); cardiovascular risk factor (6.13, 0.05); adjunctive therapy (6.04, 0.05); mitigating cardiovascular risk (5.93, 0.05); dapagliflozin combination therapy (5.93, 0.05); principal cardiovascular outcome result (5.93, 0.05); cardiovascular event (5.92, 0.05); cardiovascular outcome trial (5.64, 0.05); therapeutic ketosis (5.39, 0.05); cardiovascular protection (5.24, 0.05); therapeutic target (5.16, 0.05); altered ketone metabolism (4.9, 0.05); sglt2 inhibitor empagliflozin (4.68, 0.05); dedicated kidney (4.6, 0.05); disease-focused outcome trial (4.6, 0.05); clinical practice (4.59, 0.05); observational analyses (4.36, 0.05); renal function (4.21, 0.05); sodium-glucose cotransporter-2 (4.21, 0.05); thrifty substrate (4.17, 0.05); glp-1 receptor agonist (4.04, 0.05); new therapeutic target (4, 0.05); low renal risk (3.96, 0.05); renal outcome modification (3.96, 0.05); cohort study (3.96, 0.05); cardiovascular outcome (3.92, 0.05); low-grade inflammation (3.89, 0.05); potential contribution (3.89, 0.05); diabetes complication (3.89, 0.05); canvas program (3.89, 0.05); atrial fibrillation (3.81, 0.1); current cardiovascular outcome trial (3.81, 0.1); sodium-glucose cotransporter-2 inhibitor (3.78, 0.1); glycemic control (3.76, 0.1); sodium glucose cotransporter-2 (3.72, 0.1); cardiac function (3.72, 0.1); placebo-controlled trial (3.72, 0.1); new antihyperglycaemic agent (3.72, 0.1); cv protection (3.68, 0.1); sodium glucose transport modulation (3.68, 0.1); gastric bypass surgery (3.68, 0.1); repurposing potential (3.64, 0.1); drug classes (3.64, 0.1); clinical trial result (3.64, 0.1); exploring novel (3.64, 0.1) |
| 3 | 74 | 0.682 | 2014 | potential treatment (115.29, 1.0E-4); renin-angiotensin system inhibitor treatment (107.71, 1.0E-4); alport syndrome (107.36, 1.0E-4); basic physiology (101.54, 1.0E-4); uric acid level (97.97, 1.0E-4); cardio-renal risk factor (96.18, 1.0E-4); therapeutic potential (96.08, 1.0E-4); protective role (92.2, 1.0E-4); active-controlled trial (90.36, 1.0E-4); beneficial effect (90.36, 1.0E-4); pulmonary artery (80.66, 1.0E-4); coronary artery (80.66, 1.0E-4); no-dependent vascular relaxation (80.66, 1.0E-4); diabetic kidney (69.12, 1.0E-4); worsening heart failure (63.36, 1.0E-4); current status (57.59, 1.0E-4); cardiovascular death (56.59, 1.0E-4); sglt inhibitor (52.74, 1.0E-4); old agent (51.83, 1.0E-4); therapeutic target (50.76, 1.0E-4); sodium-glucose cotransporter (47.13, 1.0E-4); renal protection (46.61, 1.0E-4); clinical significance (46.06, 1.0E-4); serum chloride concentration (46.06, 1.0E-4); vascular complication (43.68, 1.0E-4); translational viewpoint (40.3, 1.0E-4); treating diabetes-a (40.3, 1.0E-4); potential salutary effect (40.3, 1.0E-4); supporting data (34.54, 1.0E-4); hypoglycemic agent (34.54, 1.0E-4); sglt2 inhibitor (33.93, 1.0E-4); pilot study (33.52, 1.0E-4); future perspective (31.02, 1.0E-4); kidney protection (28.78, 1.0E-4); renal effect (28.4, 1.0E-4); diabetic nephropathy (24.8, 1.0E-4); renal function (24.64, 1.0E-4); potential reduction (23.03, 1.0E-4); solute transport (17.27, 1.0E-4); reduced nephron number (17.27, 1.0E-4); japanese patient (16.58, 1.0E-4); cardiovascular outcome trial (15.78, 1.0E-4); sodium glucose (15.43, 1.0E-4); dedicated kidney (14.28, 0.001); disease-focused outcome trial (14.28, 0.001); observational analyses (12.44, 0.001); anticipated renoprotective effect (11.51, 0.001); chronic kidney disease (11.31, 0.001); low renal risk (11.22, 0.001); renal outcome modification (11.22, 0.001); cardiovascular event (10.38, 0.005); sglt2 inhibition (10.34, 0.005); glucose-lowering drug (10.08, 0.005); clinical trial (9.81, 0.005); cardiovascular safety (9.5, 0.005); systematic review (9.26, 0.005); adjunctive therapy (8.8, 0.005); non-diabetic kidney disease (8.79, 0.005); cardiovascular risk reduction (8.33, 0.005); inhibitor effect (8.22, 0.005); diabetic patient (8.22, 0.005); cardiovascular disease (7.79, 0.01); cardiovascular protection (7.64, 0.01); heart failure (7.44, 0.01); glucagon-like peptide-1 receptor agonist (6.91, 0.01); established nephropathy clinical evaluation trial result (6.42, 0.05); sodium-glucose cotransporter-2 (6.13, 0.05); glp-1 receptor agonist (5.9, 0.05); cohort study (5.78, 0.05); baseline kidney function (5.75, 0.05); canvas program (5.66, 0.05); atrial fibrillation (5.54, 0.05); sodium glucose cotransporter-2 (5.43, 0.05); cardiac function (5.43, 0.05); drug classes (5.32, 0.05); baseline characteristics (5.32, 0.05); clinical trial result (5.32, 0.05); sodium-glucose cotransporter-2 inhibitor (5.29, 0.05); glucose-lowering therapy (5.18, 0.05); pathophysiologic mechanism (5.12, 0.05); future therapeutic target (5.12, 0.05); cardioprotective effect (4.99, 0.05); renal outcome trial (4.9, 0.05); sglt2 function (4.85, 0.05); nonalcoholic fatty liver disease (4.85, 0.05); glucose-lowering agent (4.73, 0.05); underlying mechanism (4.73, 0.05); tissue mineral density (4.73, 0.05); empa-reg outcome (4.65, 0.05); renal event (4.4, 0.05); last decade (4.39, 0.05); diabetic cardiomyopathy (4.39, 0.05); anti-hyperglycemic agent (4.39, 0.05); future direction (4.39, 0.05); dpp-4 inhibitor (4.29, 0.05); balancing benefit (4.27, 0.05); antidiabetes drug (4.25, 0.05); european society (4.23, 0.05); atherosclerotic cardiovascular disease (4.16, 0.05); other antihyperglycemic agent (4.04, 0.05) |
| 4 | 52 | 0.796 | 2017 | other antihyperglycemic agent (126.47, 1.0E-4); diabetes mellitus patient (126.47, 1.0E-4); real-world comparison (126.47, 1.0E-4); established cardiovascular disease (126.47, 1.0E-4); hospitalization cost (126.47, 1.0E-4); real breakthrough (121.86, 1.0E-4); observational research (121.86, 1.0E-4); acc expert consensus decision (113.97, 1.0E-4); cardiovascular outcome studies (109.43, 1.0E-4); transporter-2 inhibitor (108.46, 1.0E-4); atherosclerotic cardiovascular disease (108.08, 1.0E-4); high risk (108.07, 1.0E-4); glp-1 receptor agonist (106.74, 1.0E-4); real-world clinical practice (103.51, 1.0E-4); multinational observational cohort study (103.51, 1.0E-4); sodium glucose (99.47, 1.0E-4); other antidiabetic medication (98.96, 1.0E-4); canvas program (98.58, 1.0E-4); key evidence (94.42, 1.0E-4); kidney outcome (92.32, 1.0E-4); spontaneous reporting system (89.89, 1.0E-4); disproportionality analysis (89.89, 1.0E-4); comparative effect (85.39, 1.0E-4); nephroprotective efficacy (83.95, 1.0E-4); side effect (83.95, 1.0E-4); sodium-glucose transporter (79.41, 1.0E-4); observational real-world data (79.41, 1.0E-4); reducing cardiovascular risk (74.89, 1.0E-4); dipeptidyl peptidase-4 inhibitor (71.3, 1.0E-4); clinical trial (65.1, 1.0E-4); other glucose-lowering drug (64.41, 1.0E-4); novel therapy (59.58, 1.0E-4); cardiovascular risk reduction (55.55, 1.0E-4); progenitor cell (55.39, 1.0E-4); circulating stem (52.63, 1.0E-4); drug interaction (50.93, 1.0E-4); pharmacovigilance evaluation (50.93, 1.0E-4); cohort study (46.61, 1.0E-4); amputation risk (43.95, 1.0E-4); ischaemic heart disease (39.58, 1.0E-4); cardiovascular risk factor (38.82, 1.0E-4); placebo-controlled crossover trial (37.74, 1.0E-4); diabetes management (35.85, 1.0E-4); cost model (34.63, 1.0E-4); practical consideration (33.41, 1.0E-4); glucagon-like peptide-1 receptor agonist (28.67, 1.0E-4); cardiovascular event (27.53, 1.0E-4); antihyperglycemic agent (25.76, 1.0E-4); real-world nationwide population-based cohort study (24.73, 1.0E-4); reduced risk (24.73, 1.0E-4); cotransporter-2 inhibitor (24.28, 1.0E-4); sodium-glucose cotransporter (23.23, 1.0E-4); clinical practice (21.08, 1.0E-4); chronic kidney disease (20.77, 1.0E-4); metabolic risk (19.78, 1.0E-4); diabetes mellitus (19.05, 1.0E-4); sodium glucose cotransporter (18.97, 1.0E-4); peptidase-4 inhibitor (15.69, 1.0E-4); glucose-lowering drug (15.34, 1.0E-4); cardiovascular safety (15.32, 1.0E-4); cardiovascular outcome (15.16, 1.0E-4); long-term post-marketing surveillance study (14.84, 0.001); real-world practice (14.84, 0.001); empa-reg outcome (14.73, 0.001); diabetic kidney disease (14.65, 0.001); potential mechanism (14.63, 0.001); clinical outcome (13.22, 0.001); cardiovascular risk (12.23, 0.001); empa-reg outcome trial (12.1, 0.001); systematic review (11.99, 0.001); european association (11.98, 0.001); american diabetes association (11.98, 0.001); consensus report (11.98, 0.001); renal effect (11.38, 0.001); second revolution (11.1, 0.001); cardiorenal protection (11.02, 0.001); heart failure patient (10.74, 0.005); empagliflozin monotherapy (10.57, 0.005); myocardial infarction risk score (10.57, 0.005); position statement (10.52, 0.005); peripheral artery disease (9.89, 0.005); statistical hoax (9.89, 0.005); oral antidiabetic drug (9.62, 0.005); recent cardiovascular outcome trial (9.52, 0.005); sglt2 inhibitor (9.26, 0.005); cardiac function (8.59, 0.005); peptide-1 receptor agonist (8.45, 0.005); reduced ejection fraction (8.27, 0.005); drug classes (8.09, 0.005); pharmacological management (8.09, 0.005); clinical trial result (8.09, 0.005); vascular complication (7.92, 0.005); glp1 receptor agonist (7.74, 0.01); working group (7.74, 0.01); jacc focus seminar (7.74, 0.01); european society (7.57, 0.01); diabetic patient (7.39, 0.01); cardiometabolic risk factor (7.17, 0.01); renal outcome (7.13, 0.01); sglt2 inhibition (7.03, 0.01) |
| 5 | 51 | 0.638 | 2013 | sodium glucose co-transporter (138, 1.0E-4); uric acid (89.94, 1.0E-4); pharmacodynamic effect (89.59, 1.0E-4); regulatory status (76.03, 1.0E-4); diabetes mellitus (73.43, 1.0E-4); safety evaluation (69.26, 1.0E-4); in-class treatment (69.26, 1.0E-4); current drug (62.52, 1.0E-4); urinary tract infection (55.31, 1.0E-4); randomized phase iii studies (49.1, 1.0E-4); potential role (42.36, 1.0E-4); clinical pharmacology (35.79, 1.0E-4); insulin-independent therapeutic approach (34.55, 1.0E-4); combination therapy (33.3, 1.0E-4); arterial stiffness (27.64, 1.0E-4); cardiovascular assessment study (20.73, 1.0E-4); renal effect (17.4, 1.0E-4); heart failure (15.69, 1.0E-4); risk factor (13.82, 0.001); the-art review (10.88, 0.001); transporter-2 inhibitor (9.63, 0.005); blood pressure (8.91, 0.005); selective sglt2 inhibitor (8.4, 0.005); sodium glucose co-transporter type (7.81, 0.01); baseline characteristics (7.79, 0.01); sodium-glucose cotransporter-2 inhibitor (7.34, 0.01); whole body (7.33, 0.01); dose-dependent glucosuria (7.28, 0.01); healthy subject (7.28, 0.01); sodium-glucose cotransporter-2 inhibition (7.01, 0.01); bone effect (6.91, 0.01); diabetic kidney disease (6.89, 0.01); relevant drug-drug interaction (6.43, 0.05); thorough qt (6.43, 0.05); phase iii trial (6.21, 0.05); common pathway (6.12, 0.05); dual burden (6.12, 0.05); controlled trial (6.11, 0.05); qt interval (5.76, 0.05); peptidase-4 inhibitor (5.72, 0.05); cardiorenal effect (5.69, 0.05); glucose-lowering drug (5.59, 0.05); chronic kidney disease (5.36, 0.05); cardiovascular safety (5.27, 0.05); cardiovascular disease (4.89, 0.05); renal outcome (4.79, 0.05); diabetes management (4.75, 0.05); insufficient glycemic control (4.73, 0.05); sodium-glucose cotransporter (4.71, 0.05); novel therapy (4.62, 0.05); type-2 diabete (4.49, 0.05); sglt2 inhibitor (4.28, 0.05); therapeutic target (4.17, 0.05); european society (4.03, 0.05); sglt2 inhibition (3.95, 0.05); clinical benefit (3.88, 0.05); antihyperglycemic agent (3.72, 0.1); diabetes treatment (3.66, 0.1); renal complication (3.63, 0.1); cardiovascular event (3.4, 0.1); glp-1 receptor agonist (3.27, 0.1); prevalent heart failure (3.24, 0.1); early treatment (3.22, 0.1); cohort study (3.2, 0.1); renal protection (3.19, 0.1); clinical practice (3.15, 0.1); low-grade inflammation (3.14, 0.1); potential contribution (3.14, 0.1); diabetes complication (3.14, 0.1); canvas program (3.14, 0.1); atrial fibrillation (3.08, 0.1); current cardiovascular outcome trial (3.08, 0.1); sodium glucose cotransporter-2 (3.01, 0.1); cardiac function (3.01, 0.1); new antihyperglycaemic agent (3.01, 0.1); available therapy (3, 0.1); pharmacologic management (3, 0.1); dpp-4 inhibitor (2.96, 0.1); new treatment option (2.96, 0.1); repurposing potential (2.95, 0.1); pharmacological management (2.95, 0.1); exploring novel (2.95, 0.1); pharmacotherapeutic application (2.95, 0.1); metabolic syndrome (2.93, 0.1); resting fat oxidation (2.9, 0.1); moderate intensity exercise training (2.9, 0.1); inulin-propionate ester supplementation (2.9, 0.1); overweight women (2.9, 0.1); prognostic value (2.89, 0.1); vascular complication (2.89, 0.1); arterial stiffness measurement (2.89, 0.1); working group (2.82, 0.1); recent cardiovascular outcome trial (2.72, 0.1); placebo-controlled trial (2.71, 0.1); diabetic patient (2.69, 0.5); nonalcoholic fatty liver disease (2.69, 0.5); pressure-dependent pathways-the progression (2.69, 0.5); sodium glucose (2.65, 0.5); underlying mechanism (2.62, 0.5); tissue mineral density (2.62, 0.5) |
| 6 | 43 | 0.751 | 2016 | adjunctive therapy (239.52, 1.0E-4); tissue mineral density (133.25, 1.0E-4); sglt2 function (130.51, 1.0E-4); fracture resistance (127.91, 1.0E-4); genetic ablation (127.91, 1.0E-4); reducing type (126.28, 1.0E-4); diabetes mortality (126.28, 1.0E-4); diabetic kidney disease risk (123.24, 1.0E-4); balancing benefit (121.79, 1.0E-4); dual inhibition (121.38, 1.0E-4); clinical trial data (116.49, 1.0E-4); potential therapeutic target (116.49, 1.0E-4); adjunctive use (111.62, 1.0E-4); clinical cases (111.62, 1.0E-4); sodium glucose co- transporter (106.76, 1.0E-4); inhibitor sotagliflozin (97.08, 1.0E-4); dual sglt (97.08, 1.0E-4); translational research (95.41, 1.0E-4); case study (92.27, 1.0E-4); mineral metabolism (85.7, 1.0E-4); treating kidney disease (84.11, 1.0E-4); surrogate endpoint (79.22, 1.0E-4); hard clinical renal outcome (76.06, 1.0E-4); us-the case (74.35, 1.0E-4); new glucose-lowering medication (71.28, 1.0E-4); nationwide cohort study (71.28, 1.0E-4); controlled trial (68.06, 1.0E-4); sodium-glucose co-transporter inhibitor (66.52, 1.0E-4); adjunctive treatment (66.52, 1.0E-4); cardioprotective effect (64.6, 1.0E-4); non-cardiovascular safety (61.78, 1.0E-4); study protocol (58.17, 1.0E-4); using second-generation (58.17, 1.0E-4); controlled pilot trial (55.1, 1.0E-4); bone microarchitecture (55.1, 1.0E-4); peripheral quantitative computed tomography (55.1, 1.0E-4); new oral glucose-lowering agent (52.28, 1.0E-4); adverse renal event (50.36, 1.0E-4); receiving sglt2-inhibitor (50.36, 1.0E-4); acute kidney injury (50.36, 1.0E-4); glucose-lowering agent (43.44, 1.0E-4); randomised controlled trial (39.12, 1.0E-4); first dual sglt inhibitor (35.89, 1.0E-4); heart failure (32.76, 1.0E-4); current outlook (32.31, 1.0E-4); diabetic kidney disease (30.95, 1.0E-4); treating hyperglycemia (26.25, 1.0E-4); machine learning (25.92, 1.0E-4); proportional hazards analysis (25.92, 1.0E-4); cardiovascular disease (24.95, 1.0E-4); sglt-2 inhibitor (23.81, 1.0E-4); deep neural network (22.81, 1.0E-4); sodium-glucose cotransporter-2 inhibitor (18.86, 1.0E-4); sodium glucose (18.25, 1.0E-4); cardiovascular outcome (16.79, 1.0E-4); sodium-glucose cotransporter (16.24, 1.0E-4); sodium-glucose co-transporter (16.17, 1.0E-4); electrolyte disorder (16.15, 1.0E-4); renal effect (15.7, 1.0E-4); sglt2 inhibitor (15.37, 1.0E-4); cardiovascular effect (15.32, 1.0E-4); peptidase-4 inhibitor (13.39, 0.001); glucose-lowering drug (13.09, 0.001); cardiovascular event (12.23, 0.001); chronic kidney disease (12.1, 0.001); clinical practice (12.02, 0.001); systematic review (11.57, 0.001); sglt inhibitor (11.49, 0.001); antidiabetic agent (11.49, 0.001); clinicians guide (11.4, 0.001); transporter-2 inhibitor (11.23, 0.001); diabetes management (11.13, 0.001); cardiovascular risk reduction (10.82, 0.005); novel therapy (10.82, 0.005); canagliflozin review (10.5, 0.005); efficacy profile (10.5, 0.005); japanese patient (10.2, 0.005); diabetes mellitus (10.05, 0.005); therapeutic target (9.77, 0.005); potential role (9.36, 0.005); blood pressure (9.13, 0.005); endothelial dysfunction (9.09, 0.005); renal function (8.96, 0.005); antihyperglycemic agent (8.71, 0.005); cardiometabolic risk factor (8.57, 0.005); renal protection (8.4, 0.005); cotransporter-2 inhibitor (8.26, 0.005); glp-1 receptor agonist (7.66, 0.01); cohort study (7.51, 0.01); practical consideration (7.46, 0.01); low-grade inflammation (7.36, 0.01); potential contribution (7.36, 0.01); diabetes complication (7.36, 0.01); canvas program (7.36, 0.01); atrial fibrillation (7.2, 0.01); current cardiovascular outcome trial (7.2, 0.01); potential mechanism (7.15, 0.01); sodium glucose cotransporter-2 (7.05, 0.01); cardiac function (7.05, 0.01); placebo-controlled trial (7.05, 0.01) |
| 7 | 41 | 0.752 | 2015 | empagliflozin monotherapy (110.82, 1.0E-4); kidney benefit (102.9, 1.0E-4); spanish setting (102.63, 1.0E-4); baseline glucose-lowering therapy (100.69, 1.0E-4); preclinical studies (100.53, 1.0E-4); once-weekly glp-1 analogue (99.41, 1.0E-4); once-weekly semaglutide (99.41, 1.0E-4); worsening cardiac dysfunction (97.65, 1.0E-4); using adult stem cell (95.93, 1.0E-4); kidney effect (95.85, 1.0E-4); sympathetic nerve activity (94.83, 1.0E-4); inflammasome activation (94.5, 1.0E-4); nucleotide-binding domain-like receptor protein (94.5, 1.0E-4); reduced nlrp3 (94.5, 1.0E-4); empagliflozin blunt (94.5, 1.0E-4); clinical application (93.63, 1.0E-4); salt sensitivity (87.74, 1.0E-4); nondiabetic rat model (87.74, 1.0E-4); drug-naive patient (73.02, 1.0E-4); phase iii (73.02, 1.0E-4); double-blind extension (73.02, 1.0E-4); blood pressure (70.31, 1.0E-4); 24-hour glucose variability (68.16, 1.0E-4); postprandial glucose (68.16, 1.0E-4); 4-week study (68.16, 1.0E-4); resistant hypertension (63.33, 1.0E-4); new-hope study (63.33, 1.0E-4); emerging therapy (63.33, 1.0E-4); hba1c reduction (53.76, 1.0E-4); contrasting influence (53.76, 1.0E-4); clinical studies (49.03, 1.0E-4); genital mycotic infection (49.03, 1.0E-4); hypertension receiving renin-angiotensin blocker (48, 1.0E-4); comprehensive review (47.96, 1.0E-4); off-label use protocol (46.81, 1.0E-4); placebo-controlled cardiovascular outcome trial (42.99, 1.0E-4); volume reduction (42.81, 1.0E-4); potential mechanism (42.41, 1.0E-4); phase iii trial (38.13, 1.0E-4); class effect (38.02, 1.0E-4); individual pattern (38.02, 1.0E-4); osmotic diuresis (33.09, 1.0E-4); endothelial dysfunction (31.68, 1.0E-4); sodium glucose cotransporter (30.1, 1.0E-4); adverse effect (28.21, 1.0E-4); coronary artery bypass graft surgery (26.5, 1.0E-4); potential role (25.16, 1.0E-4); body weight (24.17, 1.0E-4); diabetic kidney disease (23.98, 1.0E-4); heart failure hospitalization (21.49, 1.0E-4); differential volume regulation hypothesis (21.49, 1.0E-4); sodium glucose (19.03, 1.0E-4); diabetes mellitus (18.17, 1.0E-4); clinical outcome (17.91, 1.0E-4); sglt2 inhibitor (15.36, 1.0E-4); cardiometabolic risk factor (14.55, 0.001); transporter-2 inhibitor (13.29, 0.001); randomised trial (13.2, 0.001); peptidase-4 inhibitor (12.72, 0.001); glucose-lowering drug (12.43, 0.001); empa-reg outcomea (12.36, 0.001); adjunctive therapy (10.85, 0.001); chronic kidney disease (10.83, 0.001); renal effect (10.64, 0.005); diabetes management (10.57, 0.005); cardiovascular risk reduction (10.28, 0.005); novel therapy (10.28, 0.005); baseline characteristics (9.48, 0.005); therapeutic target (9.28, 0.005); sodium-glucose cotransporter (8.6, 0.005); combination therapy (8.57, 0.005); renal outcome (8.29, 0.005); antihyperglycemic agent (8.28, 0.005); glycemic control (7.95, 0.005); cotransporter-2 inhibitor (7.85, 0.01); renal event (7.76, 0.01); renal protection (7.68, 0.01); dpp-4 inhibitor (7.66, 0.01); position statement (7.35, 0.01); cohort study (7.13, 0.01); early treatment (7.02, 0.01); canvas program (6.99, 0.01); atrial fibrillation (6.84, 0.01); cardiac function (6.7, 0.01); renal function (6.66, 0.01); new oral glucose-lowering agent (6.56, 0.05); pharmacological management (6.56, 0.05); japanese patient (6.44, 0.05); metabolic syndrome (6.42, 0.05); vascular complication (6.42, 0.05); systematic review (6.23, 0.05); randomised controlled trial (6.13, 0.05); fracture resistance (6.13, 0.05); genetic ablation (6.13, 0.05); diabetic kidney disease risk (5.99, 0.05); sglt2 function (5.99, 0.05); diabetic patient (5.99, 0.05); nonalcoholic fatty liver disease (5.99, 0.05); pressure-dependent pathways-the progression (5.99, 0.05); cardiovascular outcome (5.94, 0.05) |
| 8 | 41 | 0.813 | 2016 | cardiovascular disease (297.79, 1.0E-4); low-grade inflammation (146.72, 1.0E-4); potential contribution (146.72, 1.0E-4); diabetes complication (146.72, 1.0E-4); sodium glucose cotransporter-2 (144.72, 1.0E-4); nonalcoholic fatty liver disease (143.88, 1.0E-4); current cardiovascular outcome trial (142.08, 1.0E-4); new antihyperglycaemic agent (137.45, 1.0E-4); repurposing potential (132.83, 1.0E-4); exploring novel (132.83, 1.0E-4); pharmacotherapeutic application (132.83, 1.0E-4); underlying mechanism (131.46, 1.0E-4); prognostic value (128.24, 1.0E-4); arterial stiffness measurement (128.24, 1.0E-4); non-alcoholic fatty liver disease (126.32, 1.0E-4); knowledge gap (121.25, 1.0E-4); diabetologists perspective (121.25, 1.0E-4); non-alcoholic steatohepatitis (121.25, 1.0E-4); renal mechanism (118.48, 1.0E-4); basic mechanism (117.41, 1.0E-4); low-density lipoprotein (112.76, 1.0E-4); tissue sodium content (111.13, 1.0E-4); systolic blood pressure (110.77, 1.0E-4); heart remodeling (108.13, 1.0E-4); multicenter community-based survey (106.08, 1.0E-4); various internal diseases (106.08, 1.0E-4); placebo-controlled trial (104.07, 1.0E-4); par2-mediated vasodilation (101.03, 1.0E-4); inflammation process (96, 1.0E-4); new possible indication (93.65, 1.0E-4); advanced hepatic fibrosis (91.06, 1.0E-4); large-scale cardiovascular outcome studies (90.97, 1.0E-4); possible mechanism (90.97, 1.0E-4); metabolic syndrome (89.46, 1.0E-4); future direction (83.8, 1.0E-4); nonalcoholic steatohepatitis (83.23, 1.0E-4); apoe knockout mice (81.6, 1.0E-4); translational clue (78.02, 1.0E-4); extracellular matrix (78.02, 1.0E-4); human cardiac myofibroblast (78.02, 1.0E-4); chronic liver disease (75.96, 1.0E-4); therapeutic target (73.36, 1.0E-4); high glucose-induced endothelial cell senescence (72.81, 1.0E-4); ii-induced redox-sensitive sglt1 (72.81, 1.0E-4); antidiabetic therapy (70.98, 1.0E-4); empa-reg outcome result (70.7, 1.0E-4); direct effect (70.7, 1.0E-4); cardiac allograft vasculopathy (70.33, 1.0E-4); future pharmacological therapy (67.61, 1.0E-4); sympathetic nervous activity (62.4, 1.0E-4); potential role (60.68, 1.0E-4); cardiometabolic risk factor (53.58, 1.0E-4); systematic review (52.7, 1.0E-4); fighting type-2 diabete (52, 1.0E-4); sodium-glucose co-transporter (51.97, 1.0E-4); common therapeutic option (50.47, 1.0E-4); chronic kidney disease boost (50.47, 1.0E-4); ejection fraction (49.1, 1.0E-4); renal protection (48.34, 1.0E-4); new kid (46.79, 1.0E-4); non-alcoholic fatty liver disease treatment (46.79, 1.0E-4); endothelial dysfunction (44.2, 1.0E-4); chronic kidney disease (41.71, 1.0E-4); liver fat (41.59, 1.0E-4); clinical practice (36.64, 1.0E-4); atrial fibrillation (36.42, 1.0E-4); cardiovascular event (34.97, 1.0E-4); randomised controlled trial (34.67, 1.0E-4); cardiovascular outcome (34.28, 1.0E-4); sodium-glucose cotransporter-2 inhibitor (33.06, 1.0E-4); japanese patient (33.04, 1.0E-4); key clinical trial (31.19, 1.0E-4); endothelial function (30.52, 1.0E-4); brachial artery shear stress (30.52, 1.0E-4); exploratory study (30.52, 1.0E-4); controlled trial (26.84, 1.0E-4); metabolic properties (25.99, 1.0E-4); future perspective (25.81, 1.0E-4); heart failure (25.8, 1.0E-4); clinical cardiology (25.6, 1.0E-4); diabetes mellitus (24.53, 1.0E-4); renal outcome (23.71, 1.0E-4); diabetic kidney disease (22.62, 1.0E-4); randomised trial (21.94, 1.0E-4); renoprotective properties (20.79, 1.0E-4); clinical potential relevance (20.74, 1.0E-4); empa-reg outcomea (20.64, 1.0E-4); sodium glucose (20.6, 1.0E-4); empa-reg outcome (19.71, 1.0E-4); renal function (17.64, 1.0E-4); dpp-4 inhibitor (17.51, 1.0E-4); renal effect (16.28, 1.0E-4); empa-reg outcome trial (15.61, 1.0E-4); cardiovascular effect (15.35, 1.0E-4); sodium-glucose cotransporter-2 inhibition (15.16, 1.0E-4); clinical trial (14.58, 0.001); peptidase-4 inhibitor (13.77, 0.001); glucose-lowering drug (13.46, 0.001); potential mechanism (12.83, 0.001); cardiovascular safety (12.68, 0.001) |
| 9 | 40 | 0.826 | 2015 | diabetic kidney disease (289.08, 1.0E-4); pressure-dependent pathways-the progression (135.54, 1.0E-4); kidney disease (135.22, 1.0E-4); glp-1 agonist (129.91, 1.0E-4); microvascular complication (129.91, 1.0E-4); renoprotective effect (127.48, 1.0E-4); sodium-hydrogen exchanger (124.13, 1.0E-4); natriuretic therapy (121.99, 1.0E-4); polish society (116.62, 1.0E-4); endocrine disorder (116.62, 1.0E-4); kidney diseases (116.62, 1.0E-4); antidiabetic drug (114.83, 1.0E-4); renal risk (112.96, 1.0E-4); managing glycaemic control (112.96, 1.0E-4); stem-cell chemokine (107.4, 1.0E-4); large-scale trial (107.4, 1.0E-4); potential confounding effect (107.4, 1.0E-4); peptidase-4 inhibitor (104.71, 1.0E-4); renal effect (97.14, 1.0E-4); diabetes therapy (94.43, 1.0E-4); cardiometabolic risk management (93.04, 1.0E-4); working group (90.51, 1.0E-4); pre-clinical prediction (88.85, 1.0E-4); incretin-based diabetes therapy (88.85, 1.0E-4); clinical trial outcome (85.32, 1.0E-4); clinical consideration (79.85, 1.0E-4); antihyperglycemic agent (78.66, 1.0E-4); renal outcome (74, 1.0E-4); new glucose-lowering agent (72.21, 1.0E-4); novel therapy (70.61, 1.0E-4); vascular complication (65.9, 1.0E-4); sodium-glucose cotransporter 2-inhibitor (64.77, 1.0E-4); oxygen deprivation (61.22, 1.0E-4); diabetic ckd development (61.22, 1.0E-4); deficient autophagic flux (61.22, 1.0E-4); real-world analysis (59.19, 1.0E-4); heart failure hospitalization risk (59.19, 1.0E-4); oral antidiabetic medication (59.19, 1.0E-4); antihyperglycemic drug (53.63, 1.0E-4); contrasting effect (53.63, 1.0E-4); sodium excretion (53.63, 1.0E-4); microvascular event (53.63, 1.0E-4); heart failure (51.71, 1.0E-4); chronic kidney disease (50.55, 1.0E-4); oral semaglutide (48.11, 1.0E-4); diabetic nephropathy (47.63, 1.0E-4); cardiovascular complication (42.62, 1.0E-4); potential beneficial effect (42.62, 1.0E-4); position statement (40.44, 1.0E-4); pharmacokinetic drug evaluation (37.16, 1.0E-4); nephrologists point (35.21, 1.0E-4); saxagliptin tablet (35.16, 1.0E-4); sodium-glucose co-transporter (33.68, 1.0E-4); sglt-2 inhibitor (32.16, 1.0E-4); glycemic control (30.29, 1.0E-4); pharmacological management (27.39, 1.0E-4); sodium glucose cotransporter (25.54, 1.0E-4); diabetes management (25.08, 1.0E-4); treating people (23.47, 1.0E-4); cardiovascular disease (23.38, 1.0E-4); dpp-4 inhibitor (21.7, 1.0E-4); critical comparison (17.6, 1.0E-4); diabetes mellitus (16.43, 1.0E-4); japanese patient (14.9, 0.001); clinical trial (14.79, 0.001); cardiovascular event (14.65, 0.001); sodium glucose (14.57, 0.001); transporter-2 inhibitor (13.94, 0.001); clinical practice (13.15, 0.001); sodium glucose co-transporter (13.12, 0.001); empa-reg outcome trial (12.83, 0.001); cardiovascular effect (12.63, 0.001); controlled trial (12.19, 0.001); cardiovascular outcome (11.88, 0.001); recent evidence (11.73, 0.001); glucagon-like peptide-1 receptor agonist (11.6, 0.001); combination therapy (10.96, 0.001); potential role (10.94, 0.001); endothelial dysfunction (10.73, 0.005); comprehensive review (10.73, 0.005); cardiometabolic risk factor (10.29, 0.005); sglt2 inhibition (10.17, 0.005); sodium-glucose cotransporter (9.97, 0.005); glucose-lowering drug (9.52, 0.005); cardioprotective effect (9.41, 0.005); sglt2 inhibitor (9.21, 0.005); cardiovascular risk (9.1, 0.005); potential mechanism (9.08, 0.005); cardiovascular safety (8.97, 0.005); adjunctive therapy (8.31, 0.005); clinical outcome (8.2, 0.005); cardiovascular risk reduction (7.87, 0.01); sodium-glucose cotransporter-2 inhibitor (7.8, 0.01); sglt2 inhibitor empagliflozin (7.54, 0.01); european association (7.43, 0.01); american diabetes association (7.43, 0.01); consensus report (7.43, 0.01); therapeutic target (7.1, 0.01); new treatment option (6.99, 0.01); heart failure patient (6.66, 0.01) |

**Note:** SGLT2: Sodium Glucose Cotransporter 2. CV: cardiovascular
