# Supplementary material for: Bibliometric Study of Sodium Glucose Cotransporter 2 Inhibitors in Cardiovascular Research
Source: Front Pharmacol. 2020 Sep 15;11:561494. doi: 10.3389/fphar.2020.561494 (PMC7522576; doi:10.3389/fphar.2020.561494)
Supplement: Supplementary file 11 [file Table_11.docx]

Supplementary Material

**
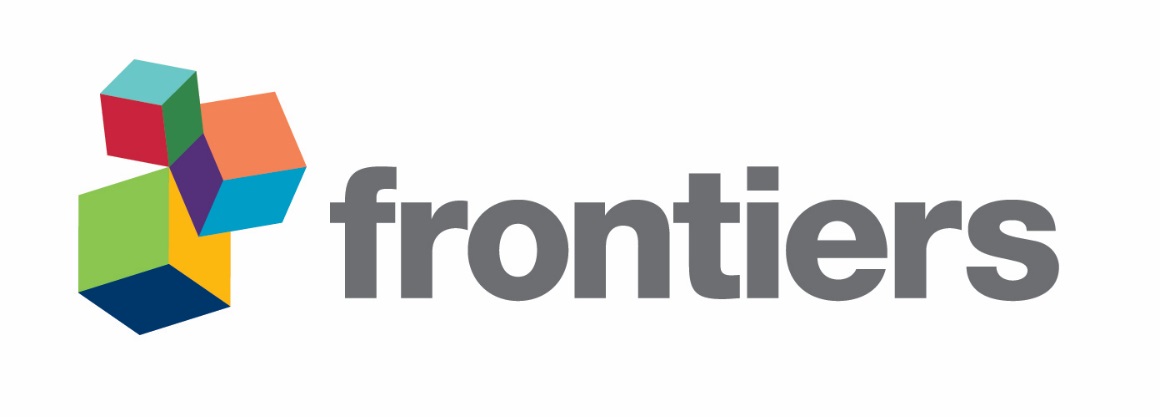
**

**Supplementary Table 11.** The author keywords co-occurring more than 5 times in articles of SGLT2 inhibitors in CV research.

| **Rank** | **Keyword** | **Occurrences** | **Total link strength** |
| --- | --- | --- | --- |
| 1 | sglt-2 inhibitors | 557 | 1663 |
| 2 | type 2 diabetes mellitus | 486 | 1437 |
| 3 | diabetes mellitus | 289 | 752 |
| 4 | heart failure | 206 | 581 |
| 5 | empagliflozin | 193 | 693 |
| 6 | cardiovascular diseases | 146 | 466 |
| 7 | dapagliflozin | 143 | 542 |
| 8 | canagliflozin | 117 | 474 |
| 9 | glp-1 receptor agonists | 92 | 349 |
| 10 | dipeptidyl peptidase-4 inhibitors | 86 | 336 |
| 11 | chronic kidney disease | 64 | 231 |
| 12 | sglt2 | 63 | 186 |
| 13 | antihyperglycemic agents | 60 | 183 |
| 14 | blood pressure | 55 | 195 |
| 15 | cardiovascular outcomes | 51 | 171 |
| 16 | hypertension | 47 | 157 |
| 17 | diabetic nephropathy | 43 | 128 |
| 18 | metformin | 42 | 164 |
| 19 | cardiovascular | 41 | 148 |
| 20 | diabetic kidney disease | 40 | 128 |
| 21 | meta-analysis | 38 | 122 |
| 22 | obesity | 36 | 124 |
| 23 | albuminuria | 34 | 136 |
| 24 | cardiovascular risk | 29 | 98 |
| 25 | inflammation | 29 | 92 |
| 26 | clinical-trials | 27 | 78 |
| 27 | safety | 27 | 95 |
| 28 | cardiovascular outcome trials | 23 | 92 |
| 29 | insulin | 23 | 89 |
| 30 | hyperglycemia | 22 | 71 |
| 31 | kidney | 22 | 73 |
| 32 | mortality | 22 | 80 |
| 33 | liraglutide | 21 | 108 |
| 34 | oxidative stress | 20 | 54 |
| 35 | type 1 diabetes mellitus | 20 | 62 |
| 36 | insulin resistance | 19 | 48 |
| 37 | glycemic control | 18 | 76 |
| 38 | sulfonylureas | 18 | 84 |
| 39 | ertugliflozin | 17 | 79 |
| 40 | body weight | 16 | 69 |
| 41 | review | 16 | 58 |
| 42 | atherosclerosis | 15 | 43 |
| 43 | diabetic cardiomyopathy | 15 | 33 |
| 44 | cardiovascular events | 14 | 50 |
| 45 | myocardial infarction | 14 | 48 |
| 46 | treatment | 14 | 39 |
| 47 | weight loss | 14 | 55 |
| 48 | cardiovascular safety | 13 | 42 |
| 49 | combination therapy | 13 | 48 |
| 50 | empa-reg outcome | 13 | 54 |
| 51 | glucose | 13 | 41 |
| 52 | stroke | 13 | 41 |
| 53 | cost-effectiveness | 12 | 38 |
| 54 | ipragliflozin | 12 | 37 |
| 55 | metabolic syndrome | 12 | 38 |
| 56 | atrial fibrillation | 11 | 38 |
| 57 | diabetes mellitus, type 2 | 11 | 27 |
| 58 | major adverse cardiovascular events | 11 | 53 |
| 59 | renoprotection | 11 | 36 |
| 60 | sitagliptin | 11 | 68 |
| 61 | network meta-analysis | 10 | 31 |
| 62 | renal function | 10 | 30 |
| 63 | risk-factors | 10 | 22 |
| 64 | sglt1 | 10 | 13 |
| 65 | thiazolidinediones | 10 | 45 |
| 66 | cardiac function | 9 | 19 |
| 67 | diastolic dysfunction | 9 | 22 |
| 68 | heart | 9 | 21 |
| 69 | hypoglycaemia | 9 | 37 |
| 70 | hypoglycemia | 9 | 26 |
| 71 | hypoglycemic agents | 9 | 19 |
| 72 | linagliptin | 9 | 64 |
| 73 | outcomes | 9 | 23 |
| 74 | pharmacokinetics | 9 | 40 |
| 75 | systematic review | 9 | 31 |
| 76 | endothelial function | 8 | 21 |
| 77 | fibrosis | 8 | 28 |
| 78 | glomerular filtration rate | 8 | 31 |
| 79 | guidelines | 8 | 42 |
| 80 | non-alcoholic fatty liver disease | 8 | 26 |
| 81 | pharmacotherapy | 8 | 26 |
| 82 | pioglitazone | 8 | 28 |
| 83 | prevention | 8 | 24 |
| 84 | saxagliptin | 8 | 58 |
| 85 | semaglutide | 8 | 60 |
| 86 | statins | 8 | 18 |
| 87 | tubuloglomerular feedback | 8 | 23 |
| 88 | uric acid | 8 | 31 |
| 89 | arterial stiffness | 7 | 21 |
| 90 | canvas | 7 | 33 |
| 91 | diabetic ketoacidosis | 7 | 24 |
| 92 | heart failure with preserved ejection fraction | 7 | 11 |
| 93 | lipids | 7 | 31 |
| 94 | observational study | 7 | 19 |
| 95 | sodium | 7 | 16 |
| 96 | aldosterone | 6 | 9 |
| 97 | body composition | 6 | 18 |
| 98 | cardioprotection | 6 | 15 |
| 99 | coronary artery disease | 6 | 15 |
| 100 | cvots | 6 | 30 |
| 101 | efficacy | 6 | 26 |
| 102 | exenatide | 6 | 50 |
| 103 | fracture | 6 | 20 |
| 104 | glimepiride | 6 | 29 |
| 105 | hba1c | 6 | 16 |
| 106 | heart function | 6 | 21 |
| 107 | kidney disease | 6 | 20 |
| 108 | lixisenatide | 6 | 52 |
| 109 | luseogliflozin | 6 | 18 |
| 110 | microvascular | 6 | 28 |
| 111 | natriuresis | 6 | 13 |
| 112 | prediabetes | 6 | 16 |
| 113 | renal outcomes | 6 | 21 |
| 114 | sodium glucose cotransporter | 6 | 16 |
| 115 | thiazolidinedione | 6 | 25 |
| 116 | tofogliflozin | 6 | 19 |
| 117 | acute coronary syndrome | 5 | 16 |
| 118 | adverse events | 5 | 13 |
| 119 | autophagy | 5 | 16 |
| 120 | biomarkers | 5 | 14 |
| 121 | cardiac metabolism | 5 | 10 |
| 122 | cardiomyopathy | 5 | 13 |
| 123 | cardiovascular mortality | 5 | 25 |
| 124 | diabetes complications | 5 | 15 |
| 125 | diabetic complications | 5 | 13 |
| 126 | diuretics | 5 | 10 |
| 127 | echocardiography | 5 | 15 |
| 128 | endothelium | 5 | 12 |
| 129 | epicardial adipose tissue | 5 | 9 |
| 130 | incretin | 5 | 18 |
| 131 | left ventricular diastolic function | 5 | 11 |
| 132 | mace | 5 | 15 |
| 133 | macrovascular | 5 | 27 |
| 134 | metabolism | 5 | 14 |
| 135 | mitochondria | 5 | 13 |
| 136 | personalized medicine | 5 | 16 |
| 137 | primary prevention | 5 | 21 |
| 138 | renal | 5 | 19 |
| 139 | renal insufficiency | 5 | 30 |
| 140 | renin-angiotensin-aldosterone system | 5 | 10 |
| 141 | secondary prevention | 5 | 21 |

**Note:** SGLT2: Sodium Glucose Cotransporter 2. CV: cardiovascular
