# Supplementary material for: Bibliometric Study of Sodium Glucose Cotransporter 2 Inhibitors in Cardiovascular Research
Source: Front Pharmacol. 2020 Sep 15;11:561494. doi: 10.3389/fphar.2020.561494 (PMC7522576; doi:10.3389/fphar.2020.561494)
Supplement: Supplementary file 12 [file Table_12.docx]

Supplementary Material

**
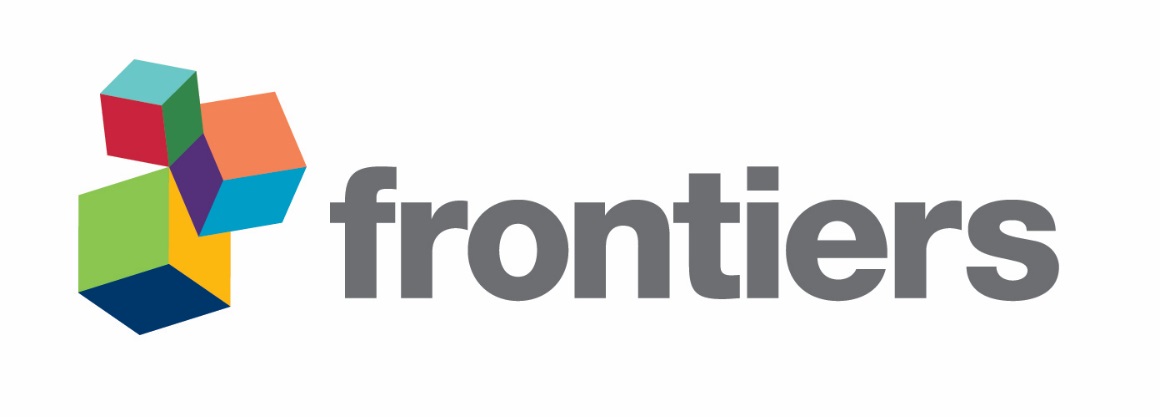
**

**Supplementary Table 12.** Replacement words unifying the similar words.

| **Label** | **Replace by** |
| --- | --- |
| cardiovascular disease | cardiovascular diseases |
| diabetes | diabetes mellitus |
| type 1 diabetes | type 1 diabetes mellitus |
| type 2 | type 2 diabetes mellitus |
| type 2 diabetes mellitus (t2dm) | type 2 diabetes mellitus |
| t2dm | type 2 diabetes mellitus |
| sglt-2 | sglt2 |
| sglt2i | sglt-2 inhibitors |
| sodium glucose co-transporter 2 (sglt2) inhibitor | sglt-2 inhibitors |
| sodium glucose co-transporter 2 inhibitor | sglt-2 inhibitors |
| sodium glucose co-transporter 2 inhibitors | sglt-2 inhibitors |
| sodium glucose cotransporter 2 | sglt2 |
| sodium glucose cotransporter 2 inhibitor | sglt-2 inhibitors |
| sodium glucose cotransporter 2 inhibitors | sglt-2 inhibitors |
| sodium-glucose co-transporter 2 inhibitor | sglt-2 inhibitors |
| sodium-glucose co-transporter-2 inhibitor | sglt-2 inhibitors |
| sodium-glucose co-transporter-2 inhibitors | sglt-2 inhibitors |
| sodium-glucose cotransporter | sglt2 |
| sodium-glucose cotransporter-2 inhibitor | sglt-2 inhibitors |
| sodium-glucose cotransporter-2 inhibitors | sglt-2 inhibitors |
| sodium-glucose transporter 2 | sglt-2 inhibitors |
| sglt-2 inhibitor | sglt-2 inhibitors |
| sglt2 inhibitor | sglt-2 inhibitors |
| sglt2 inhibitors | sglt-2 inhibitors |
| sodium-glucose co-transporter 2 inhibitors | sglt-2 inhibitors |
| sodium-glucose cotransporter 2 inhibitor | sglt-2 inhibitors |
| sodium-glucose cotransporter 2 inhibitors | sglt-2 inhibitors |
| sodium-glucose transporter 2 inhibitors | sglt-2 inhibitors |
| add-on | add-on therapy |
| blood-pressure | blood pressure |
| add-on | add-on therapy |
| cardiovascular-disease | cardiovascular diseases |
| clinical trial | clinical-trials |
| coronary-artery-disease | sglt-2 inhibitors |
| cotransporter 2 inhibition | sglt-2 inhibitors |
| cotransporter 2 inhibitors | sglt-2 inhibitors |
| cotransporter 2 inhibitor | sglt-2 inhibitors |
| diabetes-mellitus | diabetes mellitus |
| dipeptidyl peptidase-4 inhibitor | dipeptidyl peptidase-4 inhibitors |
| dpp-4 inhibitors | dipeptidyl peptidase-4 inhibitors |
| dipeptidyl peptidase 4 inhibitors | dipeptidyl peptidase-4 inhibitors |
| dpp-4 inhibitor | dipeptidyl peptidase-4 inhibitors |
| dpp4 | dipeptidyl peptidase-4 inhibitors |
| dpp4 inhibitors | dipeptidyl peptidase-4 inhibitors |
| glp-1 | glp-1 receptor agonists |
| glp-1 agonists | glp-1 receptor agonists |
| glp-1 analogue | glp-1 receptor agonists |
| glp-1 receptor agonist | glp-1 receptor agonists |
| glp-1 receptor agonists | glp-1 receptor agonists |
| glp-1ra | glp-1 receptor agonists |
| glucagon-like peptide-1 agonist | glp-1 receptor agonists |
| glucagon-like peptide-1 receptor agonist | glp-1 receptor agonists |
| glucagon-like peptide-1 receptor agonists | glp-1 receptor agonists |
| empa-reg | empa-reg outcome |
| heart-failure | heart failure |
| insulin-resistance | insulin resistance |
| metaanalysis | meta-analysis |
| placebo-controlled trial | placebo |
| risk factors | risk-factors |
| sglt2 inhibition | sglt-2 inhibitors |
| type 2 diabetes | type 2 diabetes mellitus |
| type-2 diabetes-mellitus | type 2 diabetes mellitus |
| antidiabetic drug | antihyperglycemic agents |
| antidiabetic drugs | antihyperglycemic agents |
| glucose control | antihyperglycemic agents |
| glucose-lowering drugs | antihyperglycemic agents |
| glucose-lowering therapy | antihyperglycemic agents |
| glycaemic control | antihyperglycemic agents |
| cardiovascular outcome | cardiovascular outcomes |
| cardiovascular outcome trial | cardiovascular outcome trials |
| clinical trials | clinical-trials |
| sulfonylurea | sulfonylureas |
