# Supplementary material for: Bibliometric Study of Sodium Glucose Cotransporter 2 Inhibitors in Cardiovascular Research
Source: Front Pharmacol. 2020 Sep 15;11:561494. doi: 10.3389/fphar.2020.561494 (PMC7522576; doi:10.3389/fphar.2020.561494)
Supplement: Supplementary file 13 [file Table_13.docx]

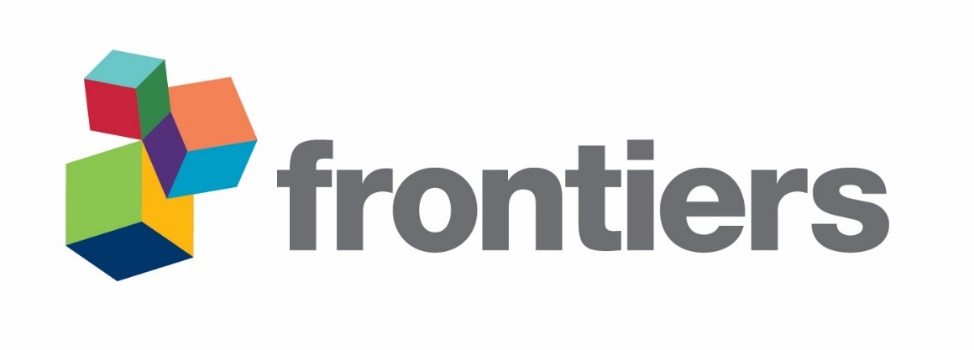
Supplementary Materia

A


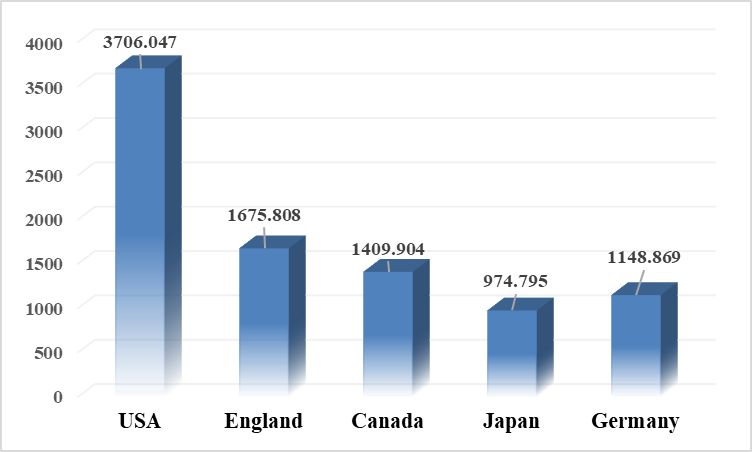


**Total IF in 2013-2019**

B


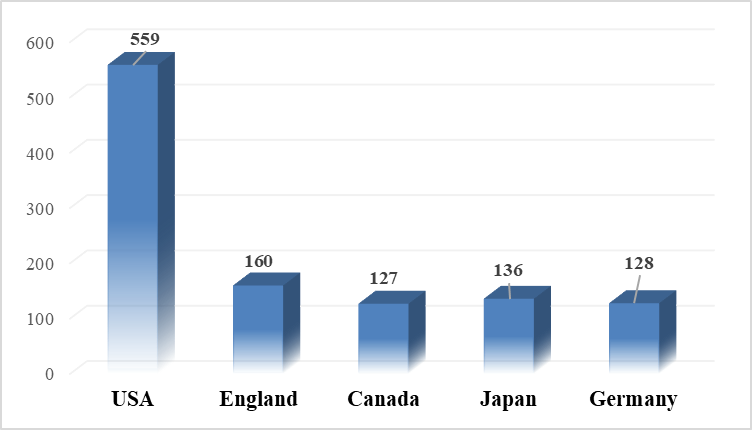


**Total publications in 2013-2019**

C

**Mean IF in 2013-2019**

**Supplementary Figure 1. Total number of publications, summarized and mean impact**

**factor for the leading nations of sodium glucose cotransporter 2 inhibitors in cardiovascular research.**
